
JOURNAL INFORMATION
==============================
NLM Title Abbreviation: J Physiol Sci
Journal ID: J Physiol Sci
NLM Journal ID: 101262417

The Journal of Physiological Sciences : JPS

ISSN: 1880-6546
EISSN: 1880-6562
Publisher: Elsevier

ARTICLE INFORMATION
==============================
PMCID: PMC11077711
DOI: 10.1186/s12576-024-00921-8
Article ID: 921
Article version: 1
Subjects: Meeting Abstracts

Proceedings of the 101st Annual Meeting of The Physiological Society of Japan

Electronic publication date: 2024 May 8
Publication date: 2024
Volume: 74
Issue: Suppl 2
Issue sponsor: Publication costs for this supplement were funded by The Physiological Society of Japan.
Electronic Location ID: 28
Copyright: © The Author(s) 2024
License: Open Access This article is licensed under a Creative Commons Attribution 4.0 International License, which permits use, sharing, adaptation, distribution and reproduction in any medium or format, as long as you give appropriate credit to the original author(s) and the source, provide a link to the Creative Commons licence, and indicate if changes were made. The images or other third party material in this article are included in the article's Creative Commons licence, unless indicated otherwise in a credit line to the material. If material is not included in the article's Creative Commons licence and your intended use is not permitted by statutory regulation or exceeds the permitted use, you will need to obtain permission directly from the copyright holder. To view a copy of this licence, visit http://creativecommons.org/licenses/by/4.0/.
License URL: https://creativecommons.org/licenses/by/4.0/

Conference name: The 101st Annual Meeting of The Physiological Society of Japan
Conference location: Kokura, Japan
Conference date: 28-30 March 2024
issue-copyright-statement: © The Physiological Society of Japan 2024

==============================

### Plenary Lecture

*J Physiol Sci* (2024) **74** (Suppl. 2):28
https://doi.org/10.1186/s12576-024-00921-8
Published: 8 May 2024

### Plenary Lecture 1

(March 28, 11:00 - 12:00, Room 1)

### 1PL01-01

### Cocaine- and Amphetamine-Regulated Transcript Peptide (CARTp) and GPR160: The Gateway to Understanding Appetite, Pain and Stress

Willis K. Samson *(Saint Louis University School of Medicine)*

The transcript identified to be upregulated by psychostimulants in rodent brain, cocaine- and amphetamine-regulated transcript (CART), was found to encode a large peptide, CARTp, which exerts multiple pharmacologic effects in rodents including in pancreas, as well as central and peripheral nervous systems. Central and peripheral sites of CARTp have been well characterized suggesting actions related to not only neuronal, but also endocrine function. Indeed, it appears that CARTp is involved in the control of glucose homeostasis both at the level of the pancreatic islet and as a mediator of gut-brain communication. While numerous, extensive pharmacologic characterizations have been reported, the physiological relevance of those actions has been difficult to establish due to the lack of an identified CARTp receptor. This has prevented the development of CARTp agonists and antagonists and thus the development of potential therapeutic strategies targeting CARTp's actions. We identified the previously orphaned, G protein-coupled receptor GPR160 to be required for the action of CARTp in a variety of tissue sites. In spinal cord, where CARTp has been demonstrated by us and others to induced pain responses, GPR160 compromise prevented the onset of spinal nerve injury pain and even was able to reverse established pain. Work by us and others has characterized the role of CARTp, produced in nodose ganglion neurons, in satiety signaling during food intake. Using passive immunoneutralization and shRNA approaches, we have demonstrated that CARTp signaling in the dorsal vagal complex is necessary for appropriate satiation cues. We now have developed two transgenic rodent models with which to further characterize the physiological relevance of CARTp signaling. A GPR160 knockout mouse line has further demonstrated the importance of CARTp-GPR160 signaling in models of neuropathic pain. Using our transgenic rat line in which the gene for GPR160 is flanked by loxP sites, we are establishing the importance of CARTp-GPR160 signaling in not only meal patterning, but also stress-induced behaviors and activation of the HPA axis. Most recently, we have developed small molecule antagonists of GPR160 that we are modifying in an attempt to produce safe and efficacious CARTp antagonists for clinical use.

### Plenary Lecture 2

(March 29, 11:00 - 12:00, Room 1)

### 2PL01-01

### Deciphering the mysteries of sleep: toward the molecular substrate for ″sleepiness”

Masashi Yanagisawa *(International Institute for Integrative Sleep Medicine (WPI-IIIS), The University of Tsukuba)*

Although sleep is a ubiquitous behavior in animal species with a nervous system, many aspects in the neurobiology of sleep remain mysterious. Our discovery of orexin, a hypothalamic neuropeptide involved in the maintenance of wakefulness, has triggered intensive research examining the exact role of the orexinergic and other neuronal pathways in the regulation of sleep/wakefulness. Orexin receptor antagonists, which specifically block the endogenous waking system, have been approved as a new drug to treat insomnia. Also, since the sleep disorder narcolepsy-cataplexy is caused by orexin deficiency, orexin receptor agonists are expected to provide mechanistic therapy for the disease; they will likely be also useful for treating excessive sleepiness due to other etiologies.

Even though the executive neurocircuitry and neurochemistry for sleep/wake switching, including the orexinergic system, has been increasingly revealed in recent years, the mechanism for homeostatic regulation of sleep, as well as the neural substrate for "sleepiness" (sleep pressure), remains unknown. To crack open this black box, we have initiated a large-scale forward genetic screen of sleep/wake phenotype in mice based on true somnographic (EEG/EMG) measurements. We have so far screened >10,000 heterozygous ENU-mutagenized founders and established several pedigrees exhibiting heritable and specific sleep/wake abnormalities. By combining linkage analysis and the next-generation whole exome sequencing, we have molecularly identified and verified the causal mutation in several of these pedigrees. Since these dominant mutations cause strong phenotypic traits, we expect that the mutated genes will provide new insights into the elusive pathway regulating sleep/wakefulness. Indeed, through a systematic cross-comparison of the SIK3 *Sleepy* mutants and sleep-deprived mice, we have found that the cumulative phosphorylation state of a specific set of mostly synaptic proteins may represent the molecular substrate of sleep pressure. We have also found that the neuronal molecular pathway LKB1-SIK3-HDAC4/5 may represent the level of sleep pressure, regulating the amount, depth, and timing of sleep by acting in different brain regions, respectively (Kim et al. Nature 612: 512-518, 2022; Zhou et al. Nature 612: 519-527, 2022).

### Plenary Lecture 3

(March 30, 11:00 - 12:00, Room 1)

### 3PL01-01

### ACE2-from fly hearts to the heart of a pandemic

Josef M Penninger *(Life Sciences Institute, University of British Columbia, Canada)*

With particular relevance to the COVID-19 pandemic, Josef Penninger will present how work on ACE2 and its role in lung failure, from the discovery in fly heart development to the first mutant mice and a fundamental understanding of SARS Coronavirus. This data provided the first molecular underpinning why the first SARS-CoV and now SARS-CoV2 causing COVID-19 became „dangerous viruses“. ACE2 is the critical receptor for SARS-Cov-2 and has taken center stage in global research and drug and vaccine development. This work has also been translated into ACE2-based drugs as rational and universal prevention and treatment strategies for COVID-19.

### Memorial Lecture

### S. Tawara Memorial Lecture

(March 28, 14:20 - 15:20, Room 1)

### 1ML01-01

### Physiological- and pathophysiological function of "pacemaker" channel in the heart

Makoto Takano *(Department of Physiology, School of Medicine, Kurume University)*

The mechanism of automaticity of the heart has been one of the central research topics in the field of Physiology. The histo-anatomical discovery of the atrioventricular (AV) node by Sunao Tawara in 1906 is a historical hallmark of the cardiac physiology. In his monograph in German entitled "The conduction system of mammalian heart", he predicted that signals from the atria could be conducted through AV node, and then distributed to the ventricles through His bundle and Purkinje fibers. The electrophysiological studies inspired by his pioneering prediction revealed that the myocytes of cardiac conduction system possess "pacemaker potential" that periodically depolarize the cell membrane to induce spontaneous action potential. They also possess unique inward cation current named "funny" current ($I_f$) or "pacemaker" current/channel. The Molecular entity of pacemaker channel is revealed to be hyperpolarization-activated, cyclic nucleotide sensitive (HCN1~4) channels. The functional significance of $I_f$ in the formation of pacemaker potential has been a subject of debate for long time. In the physiological condition, the expression of HCN channels are limited to the cardiac conduction system. However, in the cardiac failure, HCN channels are expressed in the ventricular myocytes, and suggested to induce ventricular arrhythmia. In this Tawara memorial lecture, I would like to review the findings of genetic manipulation of HCN channels, and discuss the physiological- and pathophysiological role of "pacemaker channel" in the heart.

### S. Hagiwara Memorial Lecture

(March 29, 9:50 - 10:50, Room 1)

### 2ML01-01

### Remodeling of Neuronal Circuits in vivo: Neuron-Glia Interaction

Junichi Nabekura *(National Institute for Physiological Sciences)*

Recent studies in the changes of neuronal circuits in development, learning and brain diseases focus on the contribution of glia cells to the remodeling of neuronal circuits, leading to the changes in various brain functions. In this talk, two topics regarding the interactions between glia (microglia and astrocyte) and synapses in an *in vivo* brain are introduced.

In intact cortex, microglia processes regularly contacted onto synapses (Wake et al. 2009). Microglia contact facilitated the excitatory synaptic transmission and promoted synchronous activity of cortical local circuits (Akiyoshi et al. 2018). In the damaged brain, their contact to neuronal elements became prolonged in duration, associated with the shift from "touch" to "wrapping" of the neuronal elements. To understand the functional relevance of microglial wrapping of damaged neuronal elements, epileptic action potentials were delivered in pyramidal neurons, which substantially induced axonal swelling and sustained pathological depolarization. Microglial processes migrated to and wrapped the swollen axons, followed by a recovery of neuronal membrane potential to the resting level. Thus, a contact of microglia on neuronal elements damaged could rescue neurons from excitotoxicity (Kato et al. 2016).

Second topic is the remodeling of cortical circuits by astrocyte in the chronic pain model. Peripheral nerve injury triggers maladaptive plastic changes along the somatosensory system so that altered nociceptive signal processing, represented by tactile allodynia, occurs. Indeed, remodeling of spines in the primary somatosensory cortex (S1) markedly increased during a developmental phase of exaggerated pain (Kim et al. 2011). During this developmental phase, enhanced expression of mGluR5 in astrocyte leads to an enhancement of their activity. Thrombospondin1 released from astrocyte facilitates circuits' remodeling, resulting in establishing exaggerated pain circuits in the S1. In the maintenance phase of chronic pain, the activity of astrocyte became less, leading to less plasticity of the circuits and the maintenance of pathological circuits, which could be the underlying mechanism of long-lasting exaggerated pain sensation (Kim et al. 2016). With an aim to treat the chronic pain, we attempted to relieve the affected circuits by manipulating astrocyte in the maintenance phase. Genetically manipulated and electrical re-activation of astrocyte with the manipulation of sensory afferents' activity (reduced sensation) eliminated dendritic spines related to allodynia and relived the exaggerated pain sensation. Thus, astrocyte could be the therapeutic target in chronic neuronal disorders (Takeda et al. 2022).

### Special Lecture

### Special Lecture 1

(March 28, 15:20 - 16:20, Room 1)

### 1SL01-1-01

**Biology of the Ryukyu Archipelago: a Strange World of diversity and Endemicity**

Masako Izawa *(Kitakyushu Museum of Natural History and Human History)*

The "Island" environment is a compact ecosystem within a limited space. Each island has a different environment and biota. In 2021, the islands in the Ryukyu Archipelago were registered as a World Natural Heritage site as "Amami-Oshima Island, Tokunoshima Island, Northern Part of Okinawa Island and Iriomote Island". Although the four islands were registered as representatives from the perspective of the World Natural Heritage system, the entire Ryukyu archipelago, stretching from Kyushu south to Taiwan, is of great biological interest. The biota of this region is characterized by "Diversity" and "Endemism". The most important characteristics that give rise to these characteristics are the fact that the Ryukyu Archipelago is an island group and have a complex geologic history. With the exception of the Daito Islands, which are oceanic islands, the island in the Ryukyu Archipelago have repeatedly undergone a complex process of isolation from the continent due to plate activity, re-division of islands due to sea-level changes, and connection by land bridges. In the process, organisms distributed on the continent were isolated on the fragmented islands, where they adapted to the restricted environment, acquired unique morphologies and life forms, and differentiated into diverse species and subspecies. As a result, the biota differed greatly among the three regional units of the Northern, Central, and Southern Ryukyus, which are separated by two biogeographic gaps. Furthermore, there are phenomena that are found only in this region or only on certain islands, such as animals that are divided into species on each island despite little morphological difference, species that have remained on only a few islands in the absence of neighboring species as relict endemic species, and carnivores that have survived on small islands that are uninhabitable. I would like to explain, using this region as an example, that endemic species have become what they are today after a long history of geological transition, and that we can learn about the geological history of the islands and the process of adaptation and evolution by looking at the organisms that currently inhabit them.

### Special Lecture 2-1

(March 28, 16:30 - 17:30, Room 1)

### 1SL01-2-01

**Mechanical brain: The force of synapses on the dendritic spines for memory and cognitive function**

Haruo Kasai *(WPI-IRCN, The University of Tokyo)*

Our study utilizes two-photon glutamate uncaging techniques, both *in vitro* and *in vivo*, to investigate the functions of dendritic spines within the hippocampus, cortex, and striatum. We have uncovered significant insights into the structure-function relationship of these dendritic spines, elucidating their enlargement associated with long-term potentiation (LTP), intrinsic dynamics, and the modulation of spine enlargement by dopamine in a time-sensitive manner. Moreover, our recent discoveries have revealed an intriguing phenomenon – the enlargement of these dendritic spines leads to the displacement of the presynaptic terminal, enhancing its functionality for a substantial period of 20-30 minutes. We have termed this phenomenon "Pressure-Sensation and Transduction Mechanisms" (PREST). Impressively, we estimate the force generated during spine enlargement to be approximately 0.5 kg f/cm², akin to the contractile force exhibited by smooth muscle. Notably, PREST operates independently of calcium signaling and facilitates the assembly of SNARE proteins over the same 20-30 minute timeframe. As a result, we have identified a third mode of synaptic transmission, "mechanical transmission," alongside chemical and electrical transmission. Mechanical transmission operates in conjunction with chemical transmission in spine synapses and could be the major reason for the existence of dendritic spines. Our current investigations are dedicated to unraveling the underlying mechanisms and potential functional roles of mechanical transmission. One intriguing hypothesis is its involvement in short-term potentiation (STP), a process initiated by postsynaptic NMDA receptors but with presynaptic expression. This mechanism may have implications in working memory and other cognitive functions. Our findings collectively suggest a profound concept: the brain harnesses mechanical forces to establish lasting structural imprints, contributing to both short-term and long-term memory formation, ultimately shaping cognitive function.

### Special Lecture 2-2

(March 28, 16:30 - 17:30, Room 2)

### 1SL02-01

**Spatial molecular imaging of the human islet**

Gina L. C. Yosten *(Saint Louis University School of Medicine)*

The Islets of Langerhans are complex, heterogenous micro-organs dispersed amongst the exocrine tissue of the pancreas. The function of these endocrine cell clusters is fully dependent upon their structure and composition; removal of any of the cell subtypes results in secretory failure of the other cell types. For example, loss of glucagon-producing alpha cells leads to an inability of the remaining beta cells to secrete insulin. However, the mechanisms underlying the intimate structure-function relationships between the multiple cell types within the islets remains incompletely understood. To fill gaps in our understanding of the interplay between cells of the islet neighborhood, we are employing a novel spatial omics technique, spatial molecular imaging (SMI). To this end, formalin-fixed, paraffin-embedded (FFPE) pancreas tissues from hundreds of human donors, collected at the time of organ donation, are sectioned and hybridized with probes linked to molecular barcodes representing 1000+ mRNA targets. Using a specialized cell segmentation technique designed for spatial omics data (InSituType), coupled with Leiden clustering-based cell identification methods, individual cells within each field of view are categorized (e.g., beta, delta, acinar, macrophage, etc). Differences in gene expression of each cell can then be assessed, from within its native anatomical niche. Through this work, we anticipate that novel intercellular associations will be revealed, which could lead to more effective methods for the diagnosis, treatment, and prevention of diseases of the pancreas.

### Special Lecture 3

(March 28, 17:30 - 18:30, Room 1)

### 1SL01-3-01

**Direction of international space exploration and JAXA's activities**

Naoki Nagai *(Space Exploration Center, Japan Aerospace Exploration Agency (JAXA))*

The 2nd International Space Exploration Forum (ISEF 2) was held in Tokyo in March 2018. More than 45 representatives from nations and organizations participated in discussions about international cooperation for future space exploration. ISEF2 has sparked increased interest in space exploration among participants, and many nations and organizations have planned their own space exploration scenarios. In 2019, the United States announced the Artemis program, which is comprehensive program encompassing all programs related to crewed lunar exploration and demonstrate technologies necessary for future crewed Mars Mission through Sustained Activity on the Moon. and has invited international partners to join the program. NASA, the United States' space agency, is developing the Space Launch System (SLS), the Orion spacecraft, the Civil Lunar Gateway which is the humanity's first space station around the Moon, and a Lunar lander. The Government of Japan decided on a policy for participation in international space exploration in 2019. Based on the government's policy, the JAXA Space Exploration Center (JSEC) was organized with the objective of expanding the area for human activities and initiating human lunar exploration towards Mars. The 3rd edition of the International Space Exploration Scenario has described these considerations and studies and has been released on our website.

JAXA's international contributions to the Gateway will provide an Environmental Control and Life Support System (ECLSS) with CO2 and toxic gas removal functions and the new Cargo Transfer Vehicle HTV-X. JAXA is studying and designing many elements and an integrated system for these items. For lunar exploration, the Smart Lander for Investigating the Moon (SLIM) was launched from the Tanegashima Space Center in September 2023. In addition, JAXA is developing a Lunar Polar Exploration Mission (LUPEX), which includes a Lunar Robotic Rover to investigate resources, and we are studying a pressurized rover that will be driven on the lunar surface by astronauts in the future.

JAXA will introduce some projects for future space exploration in this meeting.

### Special Lecture 4

(March 29, 8:50 - 9:50, Room 1)

### 2SL01-1-01

#### Physiological chemistry of odorant and pheromone in animals

Kazushige Touhara *(The University of Tokyo)*

The sense of olfaction plays a major role in regulating various behaviors including feeding behavior and socio-sexual interaction in many animals. Identification of specific chemical cues including pheromones and their receptors has provided a useful model to study how sensory inputs are converted into certain behavioral outputs. In this talk, I will show our work regarding intra-species chemical communication in mouse, primate, and human; 1) pheromones, receptors, and neural circuits that elicit sexual and social behavior in mice) the first pheromone candidates in a primate species, and 3) the body odorants that are involved in baby-mother and male-female communications with positive physiological and psychological effects in humans. Finally, I will describe our recent progress in decoding olfactory signals and visualizing multisensory interaction in the human brain using EEG and fMRI.

### Special Lecture 5

(March 29, 14:20 - 15:20, Room 1)

### 2SL01-2-01

#### Regulation of cell fate choices

Yukiko Gotoh[1,2] *([1]Graduate School of Pharmaceutical Sciences, The University of Tokyo, [2]International Research Center for Neurointelligence (WPI-IRCN), The University of Tokyo)*

A fundamental question in understanding tissue development is how resident stem cells or multipotent progenitors give rise to the various cell types in appropriate numbers and at the right locations to achieve tissue organization. Neural stem/progenitor cells (NPCs) in the mammalian neocortex initially divide symmetrically to increase their pool size (expansion phase). They then start to divide asymmetrically and give rise to neuronal and glial cell types in a region- and developmental stage–dependent manner. In this talk, I will present data regarding the mechanisms underlying the transition from the expansion phase to the neurogenic phase and discuss their potential role in psychiatric diseases such as autism spectrum disorder. I will then talk about the transition from the neurogenic phase to gliogenic phase. If I have time, I would also like to present some data regarding cell fate decision in response to viral infection as a separate topic.

### Special Lecture 6

(March 29, 15:20 - 16:20, Room 1)

### 2SL01-3-01

#### Next Generation Proteomics x AI Brings Revolution in Cancer Therapy

Keiichi Nakayama *(Tokyo Medical and Dental University)*

While many researchers are focused on elucidating the properties of proteins, they tend to ignore their "quantity," yet precise protein quantitation is essential for the introduction of mathematical science. To achieve absolute quantitation of all proteins, we have synthesized ~25,000 human recombinant proteins in vitro and used this information to develop a technique for absolute quantitation of a large number of proteins in a short time by rapid targeted proteomics (in vitro proteome-assisted MRM for Protein Absolute QuanTification (iMPAQT) (Patent No. 5468073). Using this iMPAQT method, we succeeded in drawing a complete picture of cancer metabolism by comparing normal cells with cancer cells, and identified key enzymes that are responsible for the big shifts in their metabolic states. These results revealed that the metabolic shift in cancer is a large-scale adaptive strategy to remodel carbon source utilization from energy production to macromolecular synthesis, and that the Warburg effect (= aerobic glycolytic shift), discovered about 100 years ago, is only a partial view of the big metabolic shifts in cancer. We also found that glutamine metabolism, a major source of nitrogen, is also greatly shifted in cancer, which we call the "second" Warburg effect. We succeeded in identifying the key enzyme PPAT that causes this shift in nitrogen metabolism. Meta-analysis of 11,000 patients with various cancers revealed that PPAT expression strongly correlated with mortality risk in most cancers, especially in small cell lung cancer. We have developed an artificial intelligence system, LIGHTHOUSE, to develop PPAT inhibitors, which actually succeeded in suppressing the progression of small cell lung cancer in mice.

### Special Lecture 7

(March 30, 8:50 - 9:50, Room 1)

### 3SL01-01

#### Molecular oxygen can be an awkward demand of us absolutely aerobic organisms

Yasuo Mori *(Kyoto University Graduate School of Engineering, Department of Synthetic Chemistry & Biological Chemistry)*

A strict demand for molecular oxygen ($O_2$) is imposed on us absolutely aerobic organisms to maintain metabolic activity through mitochondrial ATP production, while depletion of $O_2$ (hypoxia) promptly endangers our survival. Although this recognition concerning biological significance of $O_2$ may appear obligatory and undeniable, it is in fact malleable and ambiguous from the perspective of *in vivo* significance of $O_2$ in multicellular organisms. Recently, evidence has accumulated that hypoxia underlies various physiological responses in cells and organs. In addition, within the body, $O_2$ has been shown to be endogenously converted to the reactive molecular species responsible for oxidative stress that influences and often dysregulates downstream cellular signals. In this lecture, it is highlighted that a $Ca^{2+}$-permeable cation channel TRPA1 constitutes the sensors of $O_2$ and its related derivatives to activate downstream electrical/chemical signals in the peripheral and central nervous system. Also, by discussing how the TRPA1-mediated mechanism contributes to the setting of hypoxic level of $O_2$, I will attempt to robustify and conceptualize physiological aspects of hypoxia as 'physioxia'.

### Symposium

### Symposium

[1S01m]

**Dawn and Progress in Occupational Science of Japan-The Origin of Occupational Physiology and Hygiene**

March 28, 8:50 - 10:50, Room 1

### [1S01m-1]

### Significance of medical examination data in occupational physiology

*Yoshinori Marunaka[1,2,3] ([1]*Kyoto Industrial Health Association,* [2]*Ritsumeikan University,* [3]*Kyoto Prefectural University of Medicine*)

The importance of health management in occupational physiology and occupational health is widely recognized in society. In particular, Article 66 of Japan's Occupational Safety and Health Law stipulates: Employers shall provide workers with medical examinations by a physician as prescribed by Ordinance of the Ministry of Health, Labour and Welfare. Japan's health examination system is unique in the world and plays an important role in managing and promoting workers' health. Therefore, blood data obtained from health examinations in Japan are very valuable in human physiology research, both quantitatively and qualitatively. Since 2007, the Kyoto Industrial Health Association has digitized medical examination data, allowing the same person to be tracked over time, making it possible to predict future blood test data over time from the accumulated digital data of blood test results. In this presentation, I would like to introduce the results of an analysis of the relationship between the onset of lifestyle-related diseases and aging, especially from the perspective of medical examination data, and propose the contribution of "medical examination" to preventive medicine in the future.

### [1S01m-2]

### Physiology for occupational physician

*Seiji Yamada[1] ([1]*yamada seiji office for occupational health*)

Importance of physiology for occupational physicians was verified. Purpose of physiology is to obtain the true nature of human body. For the sake of investigating the essence of the human body, according to the developed research apparatus, research target was focused smaller components of the body, from organs to cells, to organelle, finally to the compound materials, such as proteins & DNA. A lot of accumulated new findings were grouped by some kinds of new concepts, finally summarized to the essential concept. Occupational medicine is the practice of the medical finding to human body. At first, they practice to the individual person, next to small group, finally to large group in working setting. An example for workers to acquire a habit of physical exercise in daily life was presented. To do the practice, physiological concept is very useful for occupational physicians.

### [1S01m-3]

### Dr. Gito Teruoka and his human connection-Labour science and Physiology-

*Kazuhiro Sakai[1] ([1]*Ohara Memorial Institute for Science of Labour*)

Before and after the war, Dr. Gito Teruoka was active in a variety of fields as the director of the Institute for Science of Labor. In my presentation I will examine his connections and with people and focus on the physiological research of Ama divers. Teruoka entered the Tokyo Imperial University School of Medicine in 1910. He studied under Professor Hisomu Nagai. At that time, Teruoka was interested in "physiology" and "labor science."At the recommendation of Professor Nagai, in 1918 he engaged in research into slums in downtown Tokyo. Later, he published it as "Social Hygiene". This is Teruoka's breakthrough work.Afterwards, at the request of Magosaburo Ohara (President of Kurashiki Spinning Co. Ltd) , Teruoka joined the Ohara Social Research Institute in 1919. Two years later, he established the Institute for Science of Labor(1921). He soon went to Germany to study. After returning to Japan, he worked hardly on labor scientific research at a spinning factory in Kurashiki. Teruoka and his fellows produced numerous results. At the same time, in response to the demands of society and his own interests, he conducted research on workers in various industries. In 1927 and 1928 he conducted physiological research on the diving of Ama divers, and in 1928, the results of his physiological research on the working conditions of Ama divers, published in an international academic journal, received great acclaim internationally.In 1936, the 15th General Meeting of the Japan Society of Physiological Association was held at the Institute of Labor Science (Kurashiki), with Dr. Teruoka serving as the president.At the International Congress of Physiological Sciences in 1965, a planned symposium was held to honor Dr.Teruoka 's world-leading research on Ama labor.

### Symposium

### [1S03m]
### Neuronal circuits mediating sensory and autonomic functions of visceral organs

March 28, 8:50 - 10:50, Room 3

### [1S03m-1]
### Mouse genetic approaches for the analysis and manipulation of the visceral sensory system

*Hideki Enomoto[1] ([1]Kobe University Graduate School of Medicine)

The luminal surface area of the gut represents the body's most extensive epithelial interface exposed to the external environment. Beyond its fundamental role in digesting and absorbing nutrients, the gut also functions as a sensory organ, gathering information from its vast luminal surroundings and communicating with the brain. Gut sensing serves as a crucial signal within the interconnected network of bodily functions, playing a pivotal role in maintaining overall homeostasis. But how exactly does the gut perceive its luminal environment? Recent research has unveiled the remarkable cellular machinery responsible for this luminal sensing. Much like neurons, enteroendocrine cells (EECs) within the gut's epithelial lining respond to luminal contents, releasing hormones and neurotransmitters that transmit signals via vagal sensory neurons to the brainstem. This revelation about the EEC-neural connection challenges the traditional notion that EECs exclusively mediate their function through hormones secreted into the bloodstream. This innovative model also offers fresh insights into the viscerosensory system and inter-organ communication, and it opens up possibilities for manipulating the endocrine system through nerve stimulation. Nonetheless, our current understanding remains incomplete. EECs and vagal sensory neurons comprise various cell subtypes, and we have limited knowledge regarding which neuronal subpopulation responds to a particular EEC group. Furthermore, the functions of individual EEC-neural connections and the mechanisms governing their development and maintenance remain largely elusive. In this presentation, I will introduce the mouse genetic approaches that we have developed to gain a deeper understanding of the anatomy and physiology of EEC-neural connections.

### [1S03m-2]
### Elucidating the mechanisms of gut osmolality detection using in vivo imaging of gut-innervating neurons

*Takako Ichiki[1] ([1]Division of Oral Biochemistry, Graduate School of Medical and Dental Sciences, Niigata University)

Fine balance between thirst and gain-of water is essential for maintaining body fluid homeostasis. Although emerging evidence suggests that gut osmolality sensing inhibits thirst neurons in brain, it remains unclear the mechanisms of how visceral sensory neurons sense gut osmolality changes. To observe the visceral osmolality responses, we established in vivo imaging from vagal and dorsal root ganglion (DRG) neurons combined with intestinal infusion. We demonstrated that the vagal but not the spinal pathway mediates visceral osmolality responses. Calcium imaging from individual vagal neurons reveals gut hypotonic stimuli activate a dedicated vagal population. We revealed that hypotonic responses are mediated by vagal afferents innervating the hepatic portal area (HPA). Eliminating sensory inputs from HPA selectively abolished hypotonic responses in vagal neurons. Recording from forebrain thirst neurons and behavioral analyses showed that HPA-derived osmolality signals are required for feed-forward thirst satiation and drinking termination. HPA-innervating vagal afferents do not sense osmolality itself but these responses are partly mediated by vasoactive intestinal peptide. Together, our results revealed gut hypoosmolality as an important vagal sensory modality to regulate thirst circuit activity through the HPA pathway.

### [1S03m-3]
### Multiplexed organ-scale mapping of axonal projection using fluorescent barcode vectors

*Daichi Moriyasu[1], Fuyuki Kamizono[1], BIswanath Saha[1], Hikari Takeshima[1], Yuki Ishida[1], Satoshi Fujimoto[1], Itaru Imayoshi[2], Takeshi Imai[1] ([1]Graduate School of medical science, Kyushu University, [2]Graduate School of Biostudies, Kyoto University, Kyoto, Japan)

Brain functions often involve computation in multiple brain areas. Some brain functions, such as sensory and motor processing, also require communication with other organs in the body. Therefore, we need to obtain a brain-wide and even a body-scale connectivity map to study brain functions. However, existing methods for circuit tracing have several limitations, hampering comprehensive analyses of the whole brain and body-scale connectivity map. For example, single-tracer injection visualizes projections from only one area per animal. Therefore, data obtained from multiple animals have to be registered to a standard brain atlas, making it challenging to evaluate fine topographic organization. Recently, RNA barcode-based tools (e.g., MAPseq) have been developed for multiplexed connectivity mapping. However, this strategy cannot provide detailed morphological information. To overcome these limitations, we sought to develop a multiplexed mesoscopic mapping tool named "fluorescent barcode vectors".

We constructed vectors expressing one or two out of the seven different fluorescent proteins (XFPs), allowing for multiplexed labeling with up to 28 combinations of XFPs. In HEK 293T cells transfected with these barcodes, each XFP signal was detected in an all-or-none fashion after linear unmixing of fluorescence signals. This allowed for the accurate identification of the barcodes in 95.0% of the cells using a simple linear classifier based on the color distance.

We then injected AAVs expressing different fluorescent barcodes into different cortical areas. The brain slices spanning the entire brain were imaged with confocal microscopy. The barcodes in neuronal somata were identified at 98.2% accuracy by the linear classifier based on the color distance. Axons were also clearly visualized with different fluorescent barcodes, demonstrating topographic organization in subcortical areas.

For the automated identification of neurites labeled with fluorescent barcodes, we developed a machine learning-based "barcode reader" that enables pixel classification based on both color and morphological information. This program distinguished even densely labeled fine axonal signals based on the limited number of training datasets.

Our fluorescent barcode vectors with automated barcode reader provide a powerful tool for a multiplexed mapping of organ-scale connectivity with rich morphological information.

### [1S03m-4]

### Autonomic responses via the vestibular system

*Chikara Abe[1] ([1]*Gifu University*)

The vestibular organs in the inner ear play an important role in postural control. The vestibular organs consist of the semicircular canals and otoliths, which are responsible for the sensation of rotation and linear acceleration, respectively. Input from the vestibular organs is integrated centrally with visual, bone, and muscle input for proper postural control. Interestingly, the vestibular system is involved not only in the sense of balance but also in sympathetic activity, one of the autonomic nervous systems (vestibular-sympathetic reflex). Recent studies have revealed that this reflex is involved in arterial blood pressure regulation, body temperature, feeding, immunity, and bone and muscle metabolism. The first half of this talk will focus on these physiological functions mediated by the vestibular system, and will present the peripheral and central mechanisms. In addition, daily gravity change input (change in the magnitude and direction of gravity due to body movement) is important for maintaining vestibular function, and the risk of falling is increased in the elderly (decrease in daily activity) and astronauts (weightless environment), in whom this input is reduced. In the latter half of this talk, I will present the mechanisms of vestibular dysfunction in the elderly and astronauts and countermeasures for deconditioning caused by the decline in vestibular function that have been revealed by previous studies.

### [1S03m-5]

### A Parallel Labeled-line Organization of Sympathetic Nervous System That Regulates Visceral Organs

Yukiko Harima[1], *Kazunari Miyamichi[1] ([1]*RIKEN BDR*)

The sympathetic nervous system exerts regulatory control over various organs. Previous research has often regarded the sympathetic nervous system as a homogenous entity, neglecting its multifaceted functional heterogeneity. Recent single-cell transcriptomic analyses on the adult mouse spinal cord have disclosed a dozen discrete subtypes among spinal sympathetic preganglionic neurons (preGNs). However, the functional relationship between these genetically defined preGN subtypes and the regulation of distinct organs remains unclear. Here we characterize two specific subtypes of sympathetic preGNs by viral genetic-based methods. Specifically, within the lower thoracic spinal cord, a preGN subtype expressing *cocaine- and amphetamine-regulated transcript prepropeptide* (*Cartpt+*) exclusively innervated the celiac/superior mesenteric ganglia (CG/SMG), whereas another subtype expressing *oxytocin receptor* (*Otr+*) selectively projects to the adrenal gland. Chemogenetic activation of either *Cartpt+* or *Otr+* preGNs selectively induced c-Fos, indicative of neural activation, in the postganglionic neurons in the CG/SMG or the adrenal medulla, respectively. The former suppressed intestinal motility, as assessed by intestinal transition time, whereas the latter specifically triggered the secretion of adrenaline, culminating in sex-dependent hyperglycemia. Collectively, our data demonstrate a parallel labeled-line organization from distinct preGN subtypes to regulate specific organ functions.

### Symposium

### [1S04m]
### Emotional influence on the autonomic nervous system

March 28, 8:50 - 10:50, Room 4

### [1S04m-1]
### Oxytocinergic nervous system that facilitates sympathetic thermogenesis

*Akihiro Fukushima[1], Kazuhiro Nakamura[1] ([1]*Department of Integrative Physiology, Nagoya University Graduate School of Medicine*)

Oxytocin (OXT), a hypothalamic neuropeptide produced in neurons of the paraventricular hypothalamic nucleus (PVH) and supraoptic nucleus (SON), is released in the central nervous system during various socio-emotional experiences. Besides the social effects, the OXT neural system has also been implicated in the regulation of body temperature and metabolism through the sympathetic nervous system. However, the central neuronal link between hypothalamic oxytocinergic neurons and the sympathetic nervous system remained unclear. To address this question, we developed an adeno-associated virus vector to selectively transduce oxytocinergic neurons with a gene of interest and conducted neural tract tracing. Many oxytocinergic, but not vasopressinergic, axons were distributed in the rostral medullary raphe region (rMR) and made close apposition to sympathetic premotor neurons that drive brown adipose tissue (BAT) thermogenesis. Retrograde tracing from the rMR revealed that the PVH, but not the SON, is the source of OXT fibers in the rMR. Therefore, we hypothesized that OXT neurons in the PVH regulate BAT thermogenesis via a monosynaptic pathway to sympathetic premotor neurons in the rMR. Consistent with this hypothesis, nanoinjection of OXT into the rMR elicited a remarkable increase in BAT thermogenic sympathetic activity, which was blocked by antagonizing OXT receptors in the rMR. This OXT-induced response persisted even under blockade of glutamate receptors in the rMR, suggesting a direct excitatory action of OXT on sympathetic premotor neurons. To investigate the role of endogenous OXT, we optogenetically stimulated axons and somata of OXT neurons. Photostimulation of PVH-derived oxytocinergic axon terminals in the rMR evoked BAT thermogenesis, similar to the response to the OXT nanoinjection. Moreover, photostimulation of oxytocinergic cell bodies in the PVH potentiated BAT thermogenesis and tachycardia evoked by an NMDA injection into the rMR. These results indicate that OXT released in the rMR not only drives BAT thermogenesis and tachycardia as an excitatory transmitter, but can also potentiates glutamatergic thermogenic transmission from other brain sites to sympathetic premotor neurons. Given that OXT can be released by socio-emotional contexts, the sympathoexcitatory action of OXT may be involved in emotion-related autonomic responses.

### [1S04m-2]
### Integration of interoception, decision-making, and affect: predictive processing of allostasis

*Hideki Ohira[1] ([1]*Nagoya University*)

Allostasis means the mechanism by which the brain changes physiological states including autonomic functions to adapt to the environment in order to achieve stability or homeostasis. This concept was originally proposed by Sterling and Eyer in 1988. However, recently, allostasis has been reconceptualized from the viewpoint of predictive processing, a theory proposed by Karl Friston arguing that the brain regulates perception and motor movement by generating predictions by inner models of the external world and self and minimizing prediction errors between the predictions and sensory signals. This idea provides integrated explanations of a wide range of phenomena, including interoception that is monitoring and regulation of bodily physiological states via autonomic functions, decision-making, and accompanying affects and consciousness. Although this theory has been a hypothesis, empirical evidence has been proposed in recent years. This talk introduces the theory of predictive processing of allostasis, recent related research findings both in animal studies and human neuroimaging stidues, and issues to be examined in the future.

### [1S04m-3]
### Pre-listening auditory-reward brain network decodes psychophysiological responses to music

*Kazuma Mori[1] ([1]*National Institutes for Quantum Science and Technology*)

Music can evoke pleasurable and rewarding experiences. Past studies that examined task-related brain activity revealed individual differences in musical reward sensitivity traits, and linked them to interactions between the auditory and reward systems. However, state-dependent fluctuations in spontaneous neural activity about music-driven rewarding experiences have not been studied. Here, we used functional MRI (N=49) to examine whether the coupling of auditory-reward networks during a silent period immediately before music listening can predict the degree of musical rewarding experience. We used machine learning models and showed that the functional connectivity between auditory and reward networks, but not others, could robustly predict subjective and physiological aspects of the strong musical reward of chills. Specifically, the broad auditory-reward connections predicted the reported duration of chills, whereas the auditory-amygdala connection was associated with physiological arousal measured by skin conductance responses. Most importantly, the predictive model derived from the first sample of individuals replicated in an independent dataset using different music samples. Since magnitude changes of the brain in the prelistening silent period did not predict the rewarding responses, the current study reveals the critical role of sensory-reward connectivity in the pre-task brain state in modulating subsequent rewarding experiences.

### [1S04m-4]

### Emotion and cognition in neurological disorders with autonomic dysregulation

*Masato Asahina[1] ([1]Department of Neurology, Kanazawa Medical University)

The mechanism that maintains a constant internal environment in humans is called “homeostasis”, in which the autonomic nervous and endocrine systems play important roles. In homeostasis, autonomic reflexes respond particularly to short-term environmental changes, while hormones are secreted in response to long-term changes. For example, the baroreflex responds to rapid blood pressure fall when standing up. The head-up tilt test (HUT), which evaluates the baroreflex, is used clinically as an autonomic function test. However, the reproducibility of the readings is not so high, and some experts consider that autonomic function tests including HUT have low reliability. The main cause of the poor reproducibility is the effect of cognitive and emotional processing. Emotion involves emotional behavior such as fight or flight, and the set points of autonomic reflexes are altered to adapt to the expected behavior. In this way, emotion and autonomic activities behave simultaneously.So, if an abnormality occurs in the autonomic nervous system, does it affect emotion? Interestingly, it was reported that in pure autonomic failure (PAF), in which only the autonomic nervous system is involved, the amygdala and insular cortex activities, which are important for emotional processing, were reduced. In addition, hypoperfusion and atrophy in the anterior cingulate gyrus were reported in PAF. In addition, autoimmune autonomic ganglionopathy, in which peripheral autonomic nerves are selectively damaged, was reported to present apathy or personal character change.
In patients with Parkinson's disease, a relationship between depression and autonomic failure was reported. On the other hand, it was reported that autonomic activity associated with emotion was increased, and the anxiety index was higher in patients with postural tachycardia syndrome, an autonomic disorder with sympathetic hyperactivity. William James (1890), famous for the James-Lange theory, hypothesized that physical changes in the body happen first, which then leads to the experience of emotion. The knowledge from patients with autonomic abnormalities in the previous reports may mean that changes in the internal environment caused by autonomic activities affect generating emotional feelings. Furthermore, the close relationship between cognitive dysfunction and autonomic failure is well-known in Lewy body disease, including dementia with Lewy bodies, Parkinson's disease, etc. This relationship may indicate the existence of a common pathology, although various hypotheses have been proposed to explain it.

### Symposium

### [1S05m]
### Physiology learned from fish

March 28, 8:50 - 10:50, Room 5

### [1S05m-1]
### Evolution and Diversity in the Reproductive Biology of Sharks and Rays: Revealing Mystery of Embryonic Diapause

*Atsuko Yamaguchi[1] ([1]*Nagasaki University*)

Cartilaginous fishes, belonging to the class Chondrichthyes, are the oldest living jawed vertebrates and include more than 1200 species of sharks, rays, and chimeras. Although the number of cartilaginous fish species is relatively lesser than that of other vertebrates, a wide diversity of reproductive modes and strategies have evolved throughout their existence, dating back at least 400 million years. They all exhibit internal fertilization and a relatively small number of large offsprings. The reproductive modes can be divided into two groups based on whether embryonic development occurs external to the mother's body (oviparity) or internally (viviparity). Viviparity generally includes four modes of reproduction: yolk-sac viviparity, histotrophy, oophagy, and placental viviparity. Basic knowledge of the reproductive biology and physiology of many cartilaginous fish species remains lacking, except for species that are established as experimental model organisms or are easily accessible. An intriguing but mysterious reproductive strategy sharks and rays use is embryonic diapause. Embryonic diapause is a regulatory phenomenon that halts embryonic development and prolongs gestation to produce offsprings at the optimal time. This reproductive strategy has evolved and adapted to maximize survival and reproductive success and has been reported in some vertebrates, including pandas and bats. The diapause period for sharks and rays is 1.5 to 10 months. Embryonic diapause was considered a rare strategy exhibited by only a few species of sharks and rays; however, our recent research suggests that this strategy is adopted by many species. In this symposium, I will discuss the evolution and extensive diversity in the reproductive biology of sharks and rays and the intriguing survival strategies of embryonic diapause revealed based on our field surveys.

### [1S05m-2]
### The sophisticated architecture of elasmobranch nephron brings amazing ability transporting ions, urea, and water

*Susumu Hyodo[1], Naotaka Aburatani[1], Wataru Takagi[1] ([1]*Laboratory of Physiology, Atmosphere and Ocean Research Institute, University of Tokyo*)

Body fluid regulation is essential for all organisms to survive in their respective habitats, including freshwater (FW), seawater (SW), and terrestrial environments. Elasmobranchs (sharks, rays and skates) have adopted a unique osmoregulatory strategy for the adaptation to a high-salinity marine environment. Elasmobranchs maintain plasma ion levels approximately one-half of the osmolality of surrounding SW and they fill the other half with urea as the osmolyte. As a result, the body fluids of elasmobranchs are slightly hyperosmotic to surrounding SW (approximately 1000 mOsm), and thus they do not suffer dehydration even in the SW environment. To maintain a high concentration of urea in the body, the nephron of elasmobranchs can reabsorb over 90% of filtered urea from the primary urine, making the kidney one of the most important urea-retaining organs. To achieve such unique function, the configuration of elasmobranch nephrons is extremely sophisticated, with more functionally distinct segments and tubules than the mammalian nephrons. Beginning at the renal corpuscle, each nephron makes four turns repeatedly between the two distinct zones: the sinus and bundle zones. With our molecular and histochemical investigations, we have revealed that the five tubular segments in the bundle zone, which are wrapped by a sac-like peritubular sheath, are coordinating intimately for the efficient urea reabsorption (Hyodo et al., 2014; 2023).

Most elasmobranchs live principally in SW, but a limited number of species including the bull shark and red stingray inhabit both SW and FW environments during their life cycles. These euryhaline elasmobranchs maintain high internal urea and ion levels (the resulting plasma osmolality is 600-700 mOsm) even in FW environments, which presents an enormous challenge as the constant osmotic gradient directs an inward movement of water. We recently found that red stingray acclimated to a FW environment excretes copious diluted urine with over 80 times increase in urine volume compared to those in SW environment. The huge increase in urine volume was due to not only an increase in glomerular filtration rate, but also a decrease in tubular water reabsorption.

### [1S05m-3]
### Exploring the Diversity of Sex in Teleost

*Emma Hinako Moritoshi[1], Midori Matsuoka[2], Gen Kume[2], Shinichi Dewa[3], Tomoki Sunobe[4] ([1]*Graduate School of Agriculture, Forestry and Fisheries, Kagoshima University,* [2]*Kagoshima University Faculty of Fisheries,* [3]*Diving Service Umi-annai,* [4]*Tokyo University of Marine Science and Technology*)

Gonochorism, a phenomenon in which an individual can function exclusively as male or female throughout life, is common in animals. Gonochoristic vertebrates have two major types of sex-determining mechanisms: (1) genotypic sex determination, where sex is decided at conception with genetic differences between sexes; (2) environmental sex determination, where sex is determined after fertilization without consistent genetic differences.

Teleost have the widest range of sexual patterns and mating systems among vertebrates. The sexual patterns can be categorized into gonochoric and hermaphrodite. The majority of fishes are gonochoristic, and their sex can be determined by genetics, environmental factors, or combination of both. For example, a sex-determining gene, that would be the equivalent of Sry of mammals, has been found in two species of the genus *Oryzias* and is named Dmy. Hermaphroditism, a phenomenon in which an individual can function as both sexes at the same time or different stages of their life history, is known in 481 species across 41 families in teleost. Simultaneous hermaphroditism is known in 57 species of 13 families, and among sequential hermaphroditism, female-to-male sex change is more common (314 species of 20 families) than male-to-female sex change (62 species of 14 families) or bidirectional sex change (69 species of seven families). The evolution of protogynous and protandrous sex change is explained by the size advantage model, where Individuals change their sex as they grow larger or older to have a better chance of reproductive success.

This symposium focuses on an intriguing example of a hermaphroditic species that has Intraspecific variation in sexual patterns. *Pseudanthias rubrizonatus* is known to be born and mature as a female before changing into a male. However, our recent research revealed that in the larger population, some individuals undergo bisexuality during the juvenile stage. These bisexual juveniles are regarded as primary males, individuals that differentiate directly to males without a female maturation process. The essential factor in the occurrence of primary males was considered as male and female interaction: the pre-courtship behaviour of male would have the role of suppressing sex differentiation in juveniles and sex change in females. The suppression seems effective in smaller groups with low female population density but not in the larger group with high female population density. Under the female-biased sex ratio, not every juvenile can receive an equal amount of pre-courtship; thus, primary males were only found in the large group. Therefore, *P. rubrizonatus* has an intraspecific variation in sexual patterns according to the group size.

### [1S05m-4]

### Physiological significance of tetrodotoxin in pufferfish of the genus *Takifugu*

*Shiro Itoi[1] ([1]*Nihon University*)

Pufferfish possess tetrodotoxin (TTX), also known as pufferfish toxin, in their bodies. Although pufferfish, classified in the genus *Takifugu*, accumulate TTX at least in the liver and ovaries, other tissues that accumulate TTX are species-specific. It has been proposed that pufferfish use it as a chemical substance for protection from predators, aggregation pheromone-like substance during spawning, or searching prey, but the details remained unclear. We have previously conducted studies on the localization of TTX in pufferfish of the genus *Takifugu* using liquid chromatography-mass spectrometry (LC-MS) analysis and fluorescent immunohistochemical staining, and found that TTX is arranged to cover the body surface of pufferfish larvae (*Takifugu rubripes* and *Takifugu alboplumbeus*). Predation experiments using pufferfish larvae from wild parents and predatory fish juveniles, such as the blackfish *Girella punctata*, sea bass *Lateolabrax* sp. and Japanese flounder *Paralichthys olivaceus*, demonstrated that predatory fish juveniles spat out pufferfish larvae just after ingestion. On the other hand, pufferfish larvae from cultured parents were fed by predatory fish juveniles, and they were not spat out. These results suggest that pufferfish accumulate TTX in order to protect their offspring. Pufferfish also possess several TTX analogs, which are thought to be biosynthetic precursors of TTX, beside TTX. It has been shown that pufferfish accumulate TTX by feeding on TTX-bearing organisms such as the flatworm *Planocera multitentaculata*, which possesses high levels of TTX and its major analog, 5,6,11-trideoxyTTX, in roughly equal amounts. Although TTX and 5,6,11-trideoxyTTX were found in large amounts in the body of wild pufferfish juveniles, the TTX/5,6,11-trideoxyTTX ratio was significantly higher, ranging from 2 to 5, suggesting that pufferfish have a mechanism to selectively accumulate TTX. On the other hand, behavioral experiments and electrophysiological studies have revealed that pufferfish do not respond to TTX, but only to and are attracted to 5,6,11-trideoxyTTX. Thus, it will be interesting to investigate how pufferfish utilize TTX and its analogues in order to elucidate the physiological significance of TTX. This presentation will include the latest findings on the significance of TTX and analogs in pufferfish.

### Symposium

### [1S06m]
### New physiological functions deciphered through behavioral analysis

March 28, 8:50 - 10:50, Room 6

### [1S06m-1]
### The physiological function of Neuromedin U system in fear memory

*Kenshiro Shikano[1], Reiko Hanada[1] ([1]*Department of Neurophysiology, Faculty of Medicine, Oita University*)

Neuromedin U (NMU) was isolated in the 1980s, and its specific receptors, NMUR1 and NMUR2, were defined in 2000. Another ligand of NMU receptors, Neuromedin S (NMS), was identified in 2005. Present studies have revealed several physiological roles of the NMU system, including in feeding behavior, energy expenditure, circadian rhythmicity, and inflammation. Recently, it has been reported that NMU system is involved in higher brain function, such as reward system, stress response and memory. In this study, to investigate the new physiological function of NMU system related to stress response, passive avoidance test (PAT) was performed in NMU/NMS double-knockout mice (dKO). Both wild type (WT) and dKO mice showed fear response one day after electric foot shock. Only dKO mice showed memory retention of fear conditioning 28 days after PAT. In addition to fear response, we examined whether nociceptive response could affect fear memory in deficit of NMU system or not. The von-Frey test indicated that pain perception of dKO mice was not increased compared to WT. These results showed fear memory induced by PAT has different mechanisms from nociceptive response. Previous studies have reported that fear memory is associated with neurogenesis in hippocampus. Then, to examine the hippocampal function in fear memory, we analyzed mature and immature neurons in hippocampus. Immunohistochemistry showed that immature neurons decreased in dKO mice. There is no difference in mature neurons between WT and dKO mice. Taken together, reduction of neurogenesis may affect the fear memory induced by deficiency of NMU system. Therefore, our study implies that NMU system has important roles in fear memory.

### [1S06m-2]
### Brain region-specific dopamine dynamics during fear conditioning in mice

*Takaaki Ozawa[1], Kazuhiro Umemoto[1], Moe Nakamura[1], Ryotaro Iwamoto[1], Yuma Matsumoto[1], Tomohiro Shibata[1], Yoshinobu Oyama[1], Tom Macpherson[1], Takatoshi Hikida[1] ([1]*Osaka University, Institute for Protein Research*)

Prediction and avoidance of future aversive events are vital abilities for survival in animals. The midbrain dopamine plays an important role in aversive learning such as fear conditioning. Previous studies found that striatal dopamine release is inhibited in response to aversive predictive cues and aversive stimuli themselves; however, little is known about how cortical and subcortical dopamine release dynamics change during learning of associations between cues and aversive events. To address this question, we conducted multi-site recording of dopamine release in the frontal cortex, the nucleus accumbens and the amygdala in mice during differential auditory fear conditioning. In the present study, we trained mice in an auditory fear conditioning paradigm where one auditory stimulus (CS+) is followed by a mild electrical shock (US) whereas the other is not (CS-). As a result, we found learning-dependent changes of dopamine release during CS+. In the nucleus accumbens, dopamine levels were significantly decreased during the CS+ following conditioning. On the other hand, dopamine releases in the frontal cortex and amygdala were increased during CS+ especially after several days of conditioning. These results suggest the possibility that experience-dependent coordinated activities of cortical, accumbal and amygdaloid dopamine releases are important for adaptive fear learning and prediction in mice.

### [1S06m-3]
### The Roles of Oxytocin and Vasopressin in Behavior

*Mitsuhiro Yoshimura[1] ([1]*Department of Physiology, School of medicine, UOEH, Japan*)

The hormones oxytocin (OXT) and vasopressin (AVP), produced in the hypothalamus and secreted into the systemic circulation from the posterior pituitary, have been identified for their diverse central physiological roles. To understand the direct roles of these hormones in behavior, chemogenetics, known as Designer Receptors Exclusively Activated by Designer Drugs (DREADD), have proven invaluable. Specifically, we generated genetically modified rats expressing hM3Dq receptors (excitatory DRAEDD) exclusively in endogenous OXT or AVP neurons, allowing us to meticulously investigate the impacts of OXT and AVP on diverse behaviors. Previous studies often employed supraphysiological doses of OXT and AVP, administered externally. In contrast, our approach involves a comprehensive analysis of endogenous effects, which has yielded results sometimes differing from these previous studies. Activation of endogenous AVP neurons resulted in significant reductions in food intake, water consumption, and urine output. In contrast, specific activation of OXT neurons did not affect these parameters. Furthermore, activation of AVP neurons markedly disrupted circadian rhythms, whereas OXT neuron activation had no effect on these rhythms. Activation of both AVP and OXT neurons demonstrated analgesic effects by activating descending pain inhibitory system. Additionally, activation of endogenous OXT neurons exhibited anti-inflammatory properties. These results enhance our understanding of the intrinsic roles of OXT and AVP in behavior, pointing toward potential novel therapeutic strategies for various pathological conditions. Currently, our research delves into the impact of OXT on anxiety and depression. Specific activation of OXT neurons in juvenile rats resulted in an increase in anxiety-like and anti-depressive behaviors in adulthood. These results are still preliminary; however, in this presentation, we will discuss the details while incorporating speculative discussion. By further investigating these hormonal functions, we aim to unravel deeper insights into the intricate interplay between hormones and behavior, shedding light on potential therapeutic interventions for a range of disorders.

### [1S06m-4]

### Pathophysiologic molecular crosstalk in the brain between obese and alcohol-dependent states

*Shiki Okamoto[1], Michio Shimabukuro[2], Yasuhiko Minokoshi[3], Hiroaki Masuzaki[1]
(*[1]Division of Endocrinology, Diabetes and Metabolism, Hematology, Rheumatology, Graduate School of Medicine, University of the Ryukyus, [2]Department of Diabetes, Endocrinology and Metabolism, School of Medicine, Fukushima Medical University, [3]Division of Endocrinology and Metabolism, Department of Homeostatic Regulation, National Institute for Physiological Sciences, National Institute of Natural Sciences*)

The McKinsey Global Institute reported that obesity and alcoholism ranked third and fourth in the "Factors Damaging the Global Economy," trailing war and terrorism in second place. Adult alcohol-related health problems affect approximately 300 million people worldwide. Despite various pharmaceutical and addiction-reversal approaches, there is still no definitive cure. Although moderate alcohol intake is positively correlated with body mass index, BMI (Puukka K et al., Am J Clin Nutr 2006), and patients requiring weight loss and metabolic surgery (Cerón-Solano G et al., Cir Esp 2021) have a higher prevalence of alcohol abuse disorders, the mechanical connection between alcoholism and obesity remains unclear. The molecular mechanisms linking food and alcohol preferences have attracted attention. Frequent heavy drinkers tend to prefer animal fat over carbohydrates (Cummings JR et al., Obes Rev 2020), while it has also been reported that alcohol-sensitive individuals have a high preference for sweet tastes. SNP analysis of loci involved in sweet-taste preference in Japanese people reveals that the alcohol-metabolizing enzyme ALDH2 is associated with such a high preference for sweet tastes (Kawafune K et al., J Hum Genet 2020). Numerous studies have also reported the importance of impaired dopamine action related to the dysregulation of GABA and opioids as a brain pathology in alcoholism (Abrahao KP et al., Neuron 2017). An FDG-PET study reveals that glucose uptake in the brain of alcohol-intoxicated patients is approximately 20% (Volkow ND et al., Neuroimage 2013), suggesting that the brain intoxicated with alcohol is in a state of energy starvation similar to that during fasting. We have identified hypothalamic neurons responsible for enhancing carbohydrate preference after fasting (Okamoto S et al., Cell Rep 2018) and recently explored the functional changes in a group of molecules involved in controlling food preference in the alcohol-dependent brain. In this talk, I will introduce our novel mechanism by which γ-oryzanol, a functional component of brown rice, acts as a modulator of the brain reward system to reduce alcohol dependence and discuss the possibility of crosstalk between the brain mechanisms for behavioral preference of alcohol and animal fats.

### [1S06m-5]

### The roles of blood cells in chronic stress-induced behavioral changes

*Shiho Kitaoka[1] (*[1]Hyogo Medical University*)

A Frightening or stressful event is a risk factor for mental illnesses and alters neuronal functions. To know how such stress develops and exacerbates mental illnesses such as depression, we employ a chronic stress model in which mice are repeatedly exposed to social defeat stress. This model induces cognitive impairment and behavioral changes, including social avoidance and elevated anxiety. We previously reported that repeated stress activates microglia in the medial prefrontal cortex (mPFC) through Toll-like receptors 2 and 4 and this microglial activation is required to induce social avoidance.

We also found that repeated stress increases the number of leukocytes in the anterior cingulate cortex, which is a dorsal area to the mPFC. As stress is known to activate sympathetic nervous and neuroendocrine systems, stress induces not only neuronal dysfunctions but also functional abnormalities in multiple organs. Therefore, we examined the dynamics of blood cells in the peripheral blood after repeated stress to know whether immune cells affect neuronal functions. We found that neutrophil is increased in the peripheral blood and is sustained after the cessation of stress. Moreover, the number of neutrophils positively correlates with the susceptibility to stress. Recently, we also found the specific type of blood cells is reduced in the peripheral blood and this reduction depends on interleukin-6. In this presentation, I will introduce our latest findings regarding micro-inflammation in various organs and its behavioral relevance.

### Symposium

[1S08m]
Young Physiologists Committee
**Bridging Fields through Techniques: The Tradition and Cutting-Edge of Autonomic Science**

March 28, 8:50 - 10:50, Room 8

### [1S08m-1]

### Dysregulation of the Autonomic Nervous System with Aging - An Approach from the Interaction with the Somatic Nervous System

*Harumi Hotta[1] ([1]*Department of Autonomic Neuroscience, Tokyo Metropolitan Institute of Gerontology*)

Our laboratory has long studied the effects of somatosensory stimulation on autonomic functions. For example, when stimulation is applied to the skin or muscles of anesthetized animals, the somatosensory information is transmitted to the central nervous system via somatic afferents, resulting in reflexive changes in autonomic nerve activity and changes in visceral functions such as heart, stomach, and bladder. Such somato-autonomic reflexes are one important base for the visceral regulating effects of physical therapies such as acupuncture and thermotherapy. Sympathetic nerves are distributed not only to visceral organs but also to skeletal muscles throughout the body. Sympathetic nerve fibers innervating skeletal muscles are distributed not only in vascular smooth muscle but also in skeletal muscle fibers and neuromuscular junctions. Recent studies have shown that most of the neuromuscular junctions, one for each skeletal muscle fiber, are innervated by sympathetic nerves. We have identified a feedback mechanism between skeletal muscle and sympathetic nerves in which reflex excitation of lumbar sympathetic nerves evoked by contraction of hindlimb muscles modulates their contractility. The amplitude of the tetanic force (TF) evoked by motor nerve stimulation was reduced by approximately 10% of the original force at 20 minutes after severing the lumbar sympathetic trunk (LST), cervical spinal cord, or lumbar dorsal roots. Because this feedback mechanism assists motor nerve function, reduced sympathetic function in the elderly may lead to reduced muscle strength. There is evidence of adrenergic receptors on both skeletal muscle fibers themselves and cholinergic motor nerve endings, and each target may alter the contractility of muscle fibers when activated. In this talk, I will present our recent work on somato-autonomic reflexes that may contribute to skeletal muscle homeostasis.

### [1S08m-2]

### *in vivo* electro-physiological study of feeding and autonomic regulation in animals.

*Mamoru Tanida[1] ([1]*Department of Physiology 2, Kanazawa Medical University*)

Feeding behavior is controlled by various hormones produced from abdominal organs such as white adipose tissue (WAT), small intestine, pancreas and stomach. Leptin, one of feeding inhibitors, is produced from WAT, and acts on the hypothalamus and induces efferent autonomic changes as sympathetic activation to maintain body function. To analyze autonomic nerve regulation mechanism by leptin, we recorded autonomic nerve activities in anesthetized rats and mice by electro-physiological recording system. Since the liver sympathetic nerve is involved in glucose metabolism, we previously cleared intracellular mechanism by which leptin acts on the hypothalamus to promote hepatic sympathetic nerve activation. In particular, central leptin stimulates liver sympathetic outflow through the PI3-kinase signal in the hypothalamic arcuate nucleus. On the other hand, it is well known that feeding regulators affect not only efferent autonomic nerves, but also afferent autonomic signals. After food ingestion, various feeding inhibitors such as, leptin, cholecystokinin (CCK), insulin and GLP-1 are secreted to blood for regulating feeding behavior. Afferent vagal nerve signal is critical role of feeding regulation, thus we checked which these hormones have a strong effect on the afferent vagus nerve in mice. Vagal afferent of nerve outflow was most stimulated by CCK. In addition, CCK stimulated efferent sympathetic signals to the brown adipose tissue and kidney, and vagotomy abolished CCK-induced sympathetic activation. Thus, our study suggest that CCK acts on the vagus nerve and is involved not only in the regulation of feeding, but also in the regulation of metabolism and circulatory function through activation of the efferent sympathetic nerve.

### [1S08m-3]

### Research of regulation of homeostasis via peripheral organ – brain interactions based on the $Ca^{2+}$ imaging analysis using single neurons derived from vagal afferent nerves

*Yusaku Iwasaki[1] ([1]*Kyoto Prefectural University*)

Vagal afferent nerves play a crucial role in regulating homeostasis by transmitting information from peripheral organs to the brain. Although this concept is now widely accepted, at that time, it was believed that vagal afferent neurons constituted a heterogeneous subclass with different expressed receptors, neurotransmitters, and innervating organs. However, when I began this research on vagal afferents about 15 years ago, it remained unclear which peripheral factors directly affected specific vagal afferent subclasses. Several research teams worldwide, including ours, established the method for measuring neural activity using $Ca^{2+}$ imaging and patch clamp techniques in single neurons derived from nodose ganglia of rodent models. In behavioral or metabolic studies using animal models, we have applied techniques for surgically or chemically denervating vagal afferents, as well as methods for specifically controlling the expression of target molecules in vagal afferents using AAV. Through these approaches, we have developed research on peripheral-brain interactions via vagal afferents. This presentation will highlight the utility of the $Ca^{2+}$ imaging method for single neurons of vagal afferents in the research of the autonomic nervous system and the results obtained using this technique.

### [1S08m-4]

### Physiological mechanisms underlying interoception by cortical neuronal populations

*Takuya Sasaki[1] ([1]*Graduate School of Pharmaceutical Sciences, Tohoku University*)

Cortical and subcortical brain areas bidirectionally connect with peripheral organs and these interactions have been shown to modulate emotional behavior both in humans and rodent models, including fear and facial expressions, anxiety, and depression. Especially, accumulating evidence demonstrates interoception, the sensation of internal states of the body, is crucial to modulate heartbeats, hunger, and blood pressure. However, neurophysiological mechanisms and insights supporting this hypothesis remain to be clarified. To address this issue, we performed simultaneous recordings of multiunit spike patterns and local field potential signals from the insular cortex, vagus nerve spikes, an electrocardiogram signal, and a peripheral blood glucose concentration from freely moving rats. Recordings were daily obtained for seven hours. At single-cell levels, a subset of insular cortical neurons increased or decreased their spike rates in response to changes in heart rates, blood glucose levels, and vagus nerve spikes. These results highlight insular cortical neurons as a detector of temporal changes in interoceptive signals, such as heart rates and blood glucose levels, which may cause various emotional valence and, in turn, regulate these organ signals through efferent controls in the brain-body axis.

### Symposium

### [1S09m]
### Neurobiology of projection axon: default and update

March 28, 8:50 - 10:50, Room 9

### [1S09m-1]
### Morphological and functional developments of neural circuits at the somatosensory thalamus

*Mitsuharu Midorikawa[1] ([1]*Kyoto University*)

During development, the neural circuit changes drastically before maturation. However, the developmental changes at the presynaptic side remain largely elusive at most of the central nerve systems. Here, we examined the morphology and synaptic function of the developing neural circuit at the mouse somatosensory thalamus. At the rodent somatosensory thalamus, the somatosensory information of the whisker is conveyed to the ventral posteromedial thalamic nucleus (VPM). We recently clarified the functional development of the single presynaptic terminal that innervates the VPN relay neuron via direct patch-clamp recordings (Midorikawa & Miyata, 2021, PNAS). Using the same technique, we labeled the entire projection of the single afferent fiber by injecting a neuro-tracer directly into the presynaptic terminal through the patch pipette. From the 3D confocal reconstruction, we examined the projection patterns of the axon arbors together with the number, size, and distribution of the terminals. With development, the number and the projection area of the terminals reduced drastically at the early stage of development, and the size of the terminals increased instead. The results suggest a smaller number of strong synapses is formed proximally to the single postsynaptic cell body after maturation. We also examined the developmental changes of supra-molecular complexes inside the presynaptic terminals. By applying two-color three-dimensional stochastic optical reconstruction microscopy (STORM), we visualized the nanoscale geometry of synaptic release sites and VGCCs. Our analysis quantitatively revealed the developmental tightening of the coupling distance, which is consistent with the developmental change of the transmitter release kinetics.

### [1S09m-2]
### Single axon morphology reveals the cerebellar functional organization

*Izumi Sugihara[1] ([1]*Tokyo Medical and Dental University*)

The cerebellum has a special organization of projection neurons. Except for local axonal projections of interneurons and granule cells, it lacks intercortical long axonal projections, different from the situation in the cerebral cortex. Therefore, branching projection patterns of input axons (mossy fibers and climbing fibers) mostly determine the interconnections among cerebellar cortical areas or compartments. In the course of long-lasting research of more than 20 years, we have been clarifying single axon morphology of climbing fiber axons, which originate from the inferior olive nucleus, and mossy fiber axons originating from major sources including the spinal cord and several brain stem nuclei. So far we obtained a total of more than 150 reconstructed single axons including preliminary ones which were labeled by extracellular localized injection of biotinylated dextran amine, a simple standard technique enough reliable for large/medium-sized axons.

Climbing fiber axons branch into seven climbing fibers on average usually within a longitudinal stripe in a single lobule or a combination of particular separate lobules. Mossy fiber axons have a larger number of branches and terminals (about 50-100) which are distributed in complicated patterns regarding transverse lobules and longitudinal stripes. Translobular branching and termination of climbing and mossy fiber axons are indicative of the functional relationship between separate lobules.

By summarizing projection patterns of all reconstructed axons, we have obtained these conclusions. (1) The cerebellar longitudinal striped pattern such as represented by the zebrin expression is linked more with climbing fiber axons than mossy fiber axons and also linked with the output topography. (2) The transversal lobular pattern is linked with projections of both climbing and mossy fiber axons. Lobular links and lobular involvement in different functions can be inferred by the axonal projection patterns. (3) Bilateral mossy fiber projection is frequently observed in many mossy fiber axons. Mossy fiber projections may be involved in bilateral coordination of motor and other activities.

In conclusion, variation of single axon morphology (branching and termination pattern) is linked with the functional organization of the CNS.

### [1S09m-3]
### Acquiring information of axonal activity in vivo with two-photon calcium imaging: study on thalamocortical axons during motor learning

*Yasuhiro R Tanaka[1], Yasuyo Tanaka[2], Masanori Matsuzaki[2] ([1]*Tamagawa Univ.*, [2]*U Tokyo*)

Motor learning enables animals to acquire the precise movements necessary for achieving their daily goals efficiently. Within the neural circuits responsible for motor control, the dynamics of neuronal ensembles in the primary motor cortex undergo complex changes during the learning process, requiring interactions with the basal ganglia and cerebellum. The thalamus serves as a critical hub through which neural signals from the basal ganglia and cerebellum are conveyed to the motor cortex. To elucidate the intricate dynamics of neuronal activity responsible for transmitting signals from subcortical structures to the primary motor cortex, we employed two-photon calcium imaging to observe thalamocortical axonal activity within the motor cortex of mice engaged in the acquisition of a self-initiated lever-pull task. As the animals progressed in their motor learning, the neural activity of thalamocortical axons stabilized, and their representation of lever trajectories improved. Axons in layer 1 (L1) exhibited activity both at the initiation and termination of lever pulls, while those in layer 3 (L3) displayed activity primarily at the initiation phase. Interestingly, we found that the length of sequences in the later stage of learning was more extended in L1 compared to L3. This extended sequence in the L1 thalamocortical population activity suggests a more prolonged engagement of this population during lever pulls. Furthermore, our experiments demonstrated that stimulation of the substantia nigra pars reticulata preferentially activated L1 axons, whereas deep cerebellar nuclei (DCN) stimulation had the opposite effect, preferentially activating L3 axons. Lesions to either the dorsal striatum or the deep cerebellar nuclei (DCN) impaired motor learning and disturbed the temporal patterns in both layers. Thus, layer-specific thalamocortical signals change with learning, which requires both basal ganglia and cerebellar activities.

### [1S09m-4]

### Stimulus-induced burst of hippocampal mossy fiber projection

*Haruyuki Kamiya[1] ([1]*Department of Neurobiology, Hokkaido University Graduate School of Medicine*)

Strong repetitive stimulus occasionally enhances the excitability of the axon and leads to the burst firings of axonal spikes either originating from the physiological spike initiation site mostly at the axon initial segment, or from the ectopic sites for the spike generation. For instance, the repetitive high-frequency stimulation of hippocampal mossy fibers was demonstrated to result in burst firings possibly originating ectopically from distal axons, although the underlying mechanisms are not yet well understood. In this study, I explored the mechanisms by computational approaches using a simple model of hippocampal mossy fibers implemented with the structure of *en passant* axons as well as experimentally obtained properties of ionic conductances. When slight depolarization of distal axons was given in conjunction with the high-frequency stimulus, afterdischarges were triggered after cessation of the repetitive stimulus and lasted for a prolonged period after the stimulus. Each spike during the burst recorded from distal axons precedes that recorded from the soma, suggesting that burst firings during afterdischarges were ectopically generated from distal axons and propagated antidromically to the soma. It should be noted that when the model of the axonal voltage-dependent potassium channel was exchanged with a model of a non-inactivating type in replacement of the original inactivating one, the burst firings were not induced by the repetitive stimuli. These results suggested that the inactivating property of axonal potassium channels plays a crucial role in generating the burst activity. Accumulated inactivation of potassium channels during strong repetitive stimulus may modify the excitability of mossy fibers and cause burst firings originating ectopically from different sites from physiological spike initiation.

### Symposium

[1S10m]

**The cochlea of the inner ear — from physiological architecture to pathological relevance to age-related hearing loss**

March 28, 8:50 - 10:50, Room 10

### [1S10m-1]

### Age-related hearing loss and cochlear sound detection based on the interaction between basilar membrane vibration and hair cells' function.

*Fumiaki Nin[1] ([1]*Gifu University*)

Sound evokes sub-nanoscale vibrations within the sensory epithelium in the mammalian cochlea. The epithelium includes not only immotile cells but also contractile outer hair cells that actively shrink and elongate in synchronization with sound frequency. These cells are exposed to continuous mechanical stress, causing damage over time. The maintenance of hair cells is challenged by damage from a variety of ototoxic factors, including loud noise, aging, genetic defects, and ototoxic drugs. This damage can manifest in many forms, from dysfunction of the hair cell mechano-transduction complex to loss of specialized ribbon synapses, and hair cell death. Because mammalian hair cells do not regenerate, the repair of hair cell damage is indispensable for auditory function

In this talk, we summarize how hair cells contribute to the hearing from the viewpoint of hair cell functions and structures. Sound stimulation elicits a wave of basilar-membrane motion that travels apically and peaks at a frequency-dependent position: high frequencies evoke a maximal response near the cochlear base and progressively lower frequencies at more apical positions. As a traveling wave advances on the basilar membrane, the active process of the hair cells adds energy to the vibration to amplify the trivial mechanical vibration induced by sound. The active process possesses two force-generating mechanisms. First, active hair-bundle motility elicited by Ca2+ influx and stems from the hair bundle and tip link complex located at the apical surface of the hair cells. Second, somatic motility mediated by the voltage-sensitive protein prestin at the basolateral membrane of the outer hair cells. These processes contribute to high sensitivity and broad intensity range of normal hearing. In addition, we discuss what is known about the damage of hair cell structures, and how the functions should be changed in aging.

### [1S10m-2]

### Physiological architecture of cochlear stria vascularis, an epithelial-like tissue with a biological battery and its relevance to age-related hearing loss.

*Hiroshi Hibino[1] ([1]*Graduate School of Medicine, Osaka University*)

The cochlea of the mammalian inner ear contains an endolymph that exhibits an endocochlear potential (EP) of +80 mV with a high [$K^+$] of 150 mM. This high potential is generated by a biological battery in the stria vascularis, which constructs a double-layered epithelial-like tissue together with the neighboring spiral ligament. In age-related hearing loss, the strial cells are degraded or partially lost with impairment of the battery and EP. In healthy cochlea, acoustic stimuli allow endolymphatic $K^+$ to enter sensory hair cells and excite them. The positive EP provided by the battery accelerates this $K^+$ influx, thereby sensitizing hearing. $K^+$ exits from hair cells and circulates back to the stria vascularis, which unidirectionally transports $K^+$ to the endolymph and likely maintains its high [$K^+$]. In vivo electrophysiological assays demonstrated that the strial battery stems primarily from two $K^+$ diffusion potentials yielded by [$K^+$] gradients between intracellular and extracellular compartments in the lateral wall. Such gradients seem to be controlled by ion channels and transporters expressed in particular membrane domains of the strial two layers. A computational model, which reconstitutes unidirectional $K^+$ transport by incorporating channels and transporters in stria vascularis and connects this transport to hair cell transcellular $K^+$ fluxes, simulates the circulation current flowing between the endolymph and the perilymph. In this model, modulation of the circulation current profile accounts for the processes leading to the battery and EP loss under pathological conditions including ischemia, hereditary deafness, and possibly age-related hearing loss. Additionally, our recent analysis of the endolymph with mass spectrometry identified several proteins that are more abundant in this fluid than perilymph, another cochlear extracellular solution whose ionic composition resembles blood plasma. The obtained protein profile of the endolymph may support a linkage between hearing loss and dementia. Further analyses of the stria vascularis, battery, and endolymph may more precisely reveal the pathological mechanisms underlying various hearing disorders including age-related hearing loss.

### [1S10m-3]

### Pathophysiological insight into cochlear synaptopathy in sensorineural hearing loss including age-related hearing loss

*Hideki Takago[1] ([1]*National Rehabilitation Center for Persons with Disabilities*)

Sound encoding depends upon $Ca^{2+}$-triggered glutamatergic neurotransmission at the afferent synapse between the inner hair cell (IHC) and type I spiral ganglion neurons (SGNs). The IHC contains a specialized structure called synaptic ribbon that tethers plenty of synaptic vesicles for securely transferring continuous auditory signals. Remarkably, a single IHC is equipped with 10-30 pairs of presynaptic ribbons and their postsynaptic partner SGNs, putatively enabling this synapse to cover a broad dynamic range of sound intensity, which is indispensable for proper speech discrimination. Moreover, *in vivo* recording from the postsynaptic SGNs showed different types of firing rate - sound intensity relationship: firstly highly sensitive SGNs with high spontaneous rate and low sound threshold, secondly lowly sensitive SGNs with low spontaneous rate and high sound threshold, and thirdly a type with an intermediate sensitivity. In accordance, *ex vivo* recordings from the IHCs and SGNs in the organ of Corti demonstrated that presynaptic $Ca^{2+}$ current and excitatory postsynaptic current (EPSC) are varied in their amplitudes. Thus, the functional diversity within individual IHC ribbon synapses is considered to underlie the wide dynamic range of auditory system. Intriguingly, accumulating evidence has been clarifying that synaptic disturbance may be one of major candidate mechanisms for the sensorineural hearing loss including age-related hearing loss. In the IHC active zones, the scaffold protein Bassoon and the hair cell-specific $Ca^{2+}$ sensor Otoferlin control exocytosis in the IHCs. Here I aim to answer a question whether these two key molecules (i.e., Bassoon and Otoferlin) may be involved in the generation of synaptic functional diversity, or more specifically, the broad variation of EPSC by taking advantage of *ex vivo* postsynaptic patch-clamp recording from mutant mice SGNs. The datasets showed that disruption of Bassoon or Otoferlin drastically reduced EPSC frequency during IHC depolarization. Furthermore, large amplitudes of EPSCs persisted in both mutants despite lowered EPSC frequency. These results support the hair cell-specific hypothetical release mechanism (i.e., univesicular release) that a single vesicle fusion with a dynamic fusion pore can produce EPSC size diversity, and suggest that Bassoon and Otoferlin are involved in this mechanism, potentially playing a role in acquiring wide dynamic range of hearing.

### [1S10m-4]

### Protective effects of pyrroloquinoline quinone (PQQ) in a cochlear cell line and in mouse models of noise-induced and age-related hearing loss

*Teru Kamogashira[1] ([1]*The University of Tokyo Hospital*)

We investigated the effects of pyrroloquinoline quinone (PQQ), an oxidoreductase cofactor, on the hydrogen peroxide-induced premature senescence model in the HEI-OC1 auditory cell line and on mouse models of noise-induced and age-related hearing loss (NIHL and ARHL).
Cells were treated with PQQ for 1 day before hydrogen peroxide (100 μM) exposure. Mitochondrial respiratory capacity was damaged in this premature senescence model but was restored in cells pretreated with PQQ (0.1 nM or 1.0 nM). A decrease in mitochondrial potential, the promotion of mitochondrial fusion and the accelerated movement of mitochondria were all observed in PQQ-pretreated cells. The protein expression of sirtuin 1 (SIRT1) and peroxisome proliferator-activated receptor gamma coactivator-1α (PGC-1α) were significantly decreased under hydrogen peroxide exposure while they were increased with PQQ pretreatment, and PGC-1α acetylation was significantly decreased.
To assess NIHL, 8 weeks-old mice with and without PQQ administration were exposed to noise for 4 h. PQQ was orally administered for one week before and after noise exposure and subcutaneously once before noise exposure. For ARHL evaluation, mice were given drinking water with or without PQQ starting at 2 months of age. In the NIHL model, PQQ-treated mice had auditory brainstem response (ABR) thresholds of significantly reduced elevation at 8 kHz, a significantly increased number of hair cells at the basal turn, and significantly better maintained synapses beneath the inner hair cells compared to controls. In the ARHL model, PQQ significantly attenuated the age-related increase in ABR thresholds at 8 and 32 kHz at 10 months of age compared to controls. In addition, the hair cells, spiral ganglion cells, ribbon synapses, stria vascularis and nerve fibers were all significantly better maintained in PQQ-treated animals compared to controls at 10 months of age.
In conclusion, PQQ has a protective effect on the premature senescence model of the HEI-OC1 auditory cell line and is associated with the SIRT1/PGC-1α signaling pathway, mitochondrial structure, and mitochondrial respiratory capacity. The physiological and histological results demonstrate that PQQ protects the auditory system from NIHL and ARHL in mice.

### [1S10m-5]

### Clinical evidence regarding age-related hearing loss

*Koichiro Wasano[1] ([1]*Tokai University School of Medicine*)

Hearing and speech perception deteriorate due to age-related changes in the inner ear and central nervous system. It has been shown that failure to provide appropriate intervention for age-related hearing loss age can lead to an increased risk of various diseases such as dementia, depression, and injury associated with falls, and the importance of diagnosis and intervention. is attracting attention. On the other hand, we currently do not have a sufficient answer to the question, “How and to what extent can we prevent the progression of hearing loss?"
Our large-scale study on age-related hearing loss revealed the average hearing change by age and gender. Hearing thresholds at higher frequencies (>1000 Hz) were significantly worse in men than in women. For participants ≥70 years, hearing thresholds at low frequencies were higher in women. (Wasano K. Lancet Reg Health West Pac. 2021) Factors associated with the progression of hearing loss include age, genetics, gender (male), noise exposure, and oto-toxic agents, as well as factors associated with arteriosclerosis, such as diabetes, hyperlipidemia, calorie intake, and smoking. Avoiding exposure to such risks can be expected to prevent the progression of age-related hearing loss. As a new topic, a retrospective large-scale cohort study reported that oral administration of metformin, a biguanide hypoglycemic drug, reduced the risk of developing hearing loss by 47%, and there was also a dose-dependent relationship. (Tseng CH. Otolaryngol Head Neck Surg. 2023)
Additionally, in 2023, some papers on the effectiveness of hearing interventions for cognitive decline were published in high-impact journals. The data from US National Health and Aging Trends Study (NHATS) showed that dementia prevalence among participants with moderate to severe hearing loss was higher than prevalence among participants with normal hearing. Among 853 participants with moderate to severe hearing loss, hearing aid use was associated with lower prevalence of dementia compared with no hearing aid use. (Huang AR. JAMA. 2023) The ACHIEVE study is a multicenter, parallel-group, unmasked, randomized controlled trial of adults aged 70–84 years with untreated hearing loss and without substantial cognitive impairment. Its results suggested that a hearing intervention might reduce cognitive change over 3 years in populations of older adults at increased risk for cognitive decline but not in populations at decreased risk for cognitive decline. (Frank R Lin. The Lancet. 2023)
In this symposium, I will show some clinical evidence regarding age-related hearing loss that might interest physiologists who are engaged in the basic research of auditory system.

### Symposium

[1S02a]
Committee for Promotion of Physiome and Systems Biology
**Deployment of the next generation medicine with informatics and engineering**

March 28, 14:20 - 16:20, Room 2

### [1S02a-1]

### Mechanisms of cardiac arrhythmias revealed by biomedical engineering and informatics

*Kunichika Tsumoto[1], Yasutaka Kurata[1] ([1]*Kanzawa Medical University*)

The virtual human project, which constructs humans virtually through medicine, engineering, and informatics alliances, has been promoted over about two decades. The remarkable advance in computer performance in recent years is making it possible to construct organs such as the heart virtually within cyberspace. Nowadays organs and human bodies reproduced with high precision in cyberspace are referred to as "*digital twins*". By storing large numbers of digital twins under various virtual conditions within cyberspace, a new era of medicine may arrive in which artificial intelligence (AI) will be able to predict future morbidity and even formulate treatment plans based on diagnostic information of individual patients. We have investigated electrophysiological mechanisms underlying electrical activities that trigger lethal arrhythmias, which are long-standing unanswered questions, from aspects of nonlinear mathematical sciences as well as biomedical engineering and informatics. In this symposium, we would like to introduce some of the electrophysiological mechanisms by which the development of early afterdepolarization leads to the triggered activity formation that initiates reentrant ventricular tachycardia. Incorporating our findings into a digital twin of the heart, which is currently under development, may enable us to predict the onset of fatal arrhythmias earlier and prevent sudden cardiac death.

### [1S02a-2]

### Image data analysis and medical applications using deep learning

*Hirohiko Niioka[1] ([1]*Kyushu University*)

With the recent dramatic improvements in image analysis technology based on deep learning, many reports have been published on diagnostic assistance systems using deep learning. The presentation will introduce these deep learning technologies and examples of medical applications.

### [1S02a-3]

### Functional restoration based on physiology of cerebral oscillation and medical engineering: implantable brain machine interfaces

*Masayuki Hirata[1] ([1]*Department of Neurological Diagnosis and Restoration, Osaka University Graduate School of Medicine*)

We have investigated brain functions using intracranial EEG for brain function mapping. Intracranial EEG can stably measure high frequency activities in a single trial, and responses in the high γ band are clearly observed regardless of the type of brain activity. This allows for accurate and immediate comprehensive evaluation of functional localization. Furthermore, since there is minimal noise contamination, amplitude components and activities in lower frequency bands such as δ and θ can also be measured stably. Recently, it has been found that the phase of low-frequency band cerebral oscillation synchronizes with the power of the high γ band, a phenomenon known as phase amplitude coupling (PAC), which plays an important role in controlling brain function. Modeling these findings from the perspective of neural networks gives comprehensive understanding of cerebral oscillation. Multiple large-scale neural networks such as sensory loops, motor loops, executive loops, and emotional/memory loops each have their own resonance characteristics in α, β, low γ, and θ bands respectively. When a part of the brain is activated, this resonance is reduced, and high γ activity occurs in the activated brain area. It is believed that PAC reflects the control state determining when and where to activate or inhibit such neural networks. By leveraging the immediate and accurate characteristics of high γ responses, we can apply them to BMI technology to operate robots using intracranial EEG. Currently, research and development is being conducted to reconstruct motor/communication functions using BMI. Instead of controlling everything solely with brain signals, we can achieve higher performance more efficiently by harmonizing it with the autonomous control of robots. In order to use BMIs for a long period at home, practical application of wireless implantable BMI devices is key. We need to develop an integrated amplifier that amplifies brain waves, along with wireless power supply and wireless data communication capabilities, all within a small, low-power electronic circuit that can be implanted. Implantable medical devices fall under Class 4, which is the most challenging category of medical device development. However, after persistently working on this for over 15 years, we are finally on the verge of starting clinical trials.

### [1S02a-4]

### Current status of mental disorder biomarker development by combining human brain imaging data and machine learning methods

*Okito Yamashita[1,2] ([1]ATR, [2]RIKEN-AIP)

The number of patients with mental illnesses such as depression, and schizophrenia is increasing every year. It has become such a serious social problem. However, the current diagnosis system, which is based on patients' self-reports, does not necessarily link the diagnosis to biological factors, and in some cases, treatment is not sufficiently effective. In order to improve treatment outcomes, objective diagnostic methods using biological data (biomarkers), such as blood, genes, brain structure, and brain function, are considered important. In the neuroimaging field, the research on human brain functions has been advancing rapidly since fMRI has been developed [1]. In particular, the resting-state functional connectivity (rsFC) method[2], which assess and visualize the functional brain network, is a powerful tool to characterize age, behavior and cognitive skills of each individual. With accumulating findings of the rsFC studies, the research for clinical application of mental disorder biomarkers has begun in the late 2000s. The individual-level classification of patients and healthy controls is possible by combining the machine learning algorithm with the rsFC. Recently, a joint research project involving ATR, Hiroshima University, the University of Tokyo, Kyoto University, Showa University, and RIKEN has succeeded in developing the harmonization method that compensate for differences in machines and measurement protocols between centers. Then the multi-center generalizable depression biomarker has been developed based on a total of 1300 depressed and normal subjects (AUC = 0.74, sensitivity 72%, and specificity 61%) [3]. Although significant progress has been made on the generalization issue, there is still room for improvement in terms of accuracy. Improvement of measurement, experiment and data analysis methods along with big-data accumulation are necessary. Currently, in the Brain-Mind/Beyond project supported by AMED, the research collaborations over Japan are running in order to develop more sophisticated biomarkers.

1. Ogawa, S., Lee, T. M., Kay, A. R. & Tank, D. W. *Proceedings of the National Academy of Sciences* **87**, 9868–72 (1990).
2. Fox, M. D. & Raichle, M. E. *Nat Rev Neurosci* **8**, 700–11 (2007).
3. Yamashita, A. *et al. PLoS Biol* **18**, e3000966 (2020).

### Symposium

[1S03a]

**Hormones and life stages: physiological changes and adaptive dynamics in organisms**

March 28, 14:20 - 16:20, Room 3

### [1S03a-1]

### The Role of Hormones in Shaping Life Stages

*Kazuya Hasegawa[1] ([1]*Teikyo Heisei University*)

Throughout their lifespan, organisms navigate stages of development, growth, reproduction, and aging, each accompanied by significant physiological transformations. The endocrine system plays a crucial role, utilizing bioactive substances to manage the physiological responses inherent to each stage and to facilitate smooth transitions between them. Endocrine regulation can also give rise to health complications, potentially stemming from discrepancies between fetal composition and postnatal environment, or from a declining endocrine regulatory capacity with age. The upcoming symposium will present research on oogenesis, aging, and hormonal regulation across generations. Additionally, methods for discovering unknown hormones and advancing research in this field will be discussed.

### [1S03a-2]

### Identification of spawning-inducing peptide in bivalves and application to fisheries research

*Shohei Funayama[1], Toshie Matsumoto[1], Yuki Hirano-Maeda[1], Yoshio Kodera[2]
([1]*Fisheries Technology Institute, Japan Fisheries Research and Education Agency,* [2]*Department of Physics, School of Science, Kitasato University*)

Bivalve aquaculture is widely spread in Japan and is produced on a large commercial scale, and it is centered on bivalves that can be cultivated in natural sea waters by collecting naturally growing larvae such as Pacific oysters and scallops. On the other hand, the abundance of bivalves inhabiting coastal areas has been declining, and research is being conducted to elucidate the causes and develop countermeasures. The pen shell (*Atrina pectinata*) is a large bivalve widely distributed in the sandy and muddy inner bays of the Ariake Sea, Seto Inland Sea, Ise Bay, and Mikawa Bay in Japan. It is one of the important fishery species traded at high prices due to its large adductor muscle and extremely delicious taste. However, the amount of resources has significantly decreased since the 2000s. The Ariake Sea is one of the main production areas of this species, and the situation has been severe, with fishing suspended for 10 consecutive years. As a countermeasure to this problem, the involving the technological development of aquaculture using artificially produced juveniles is underway. In general, bivalves induce spawning by raising water temperature, but the effect is uncertain. Therefore, many broodstocks are prepared, and eggs and sperm collected from individuals that respond to the stimulus are used to secure fertilized eggs. Accordingly, there is a need to develop more efficient technology to produce pen shell seedlings. It is thought that natural spawning in the pen shell involves the production of a bioactive substance that induces spawning, which acts on the gonads to release eggs and sperm. Utilizing this bioactive substance in the conventional method may induce spawning more reliably. We searched for a bioactive substance that induces spawning in the visceral ganglion of the pen shell and identified a novel peptide consisting of 26 amino acid residues. We named this spawning-inducing peptide (SIP) because it induces egg and sperm release when administered to the broodstocks. We confirmed that fertilized eggs obtained with SIP administration develop normally to larvae. This method of collecting eggs using SIP is now being used at several hatcheries and contributes to the systematic production of seedlings as a reliable method. Furthermore, we are working on developing a method to evaluate egg quality. The administration of SIP has enabled the collection of eggs from individual pen shells, which was previously impossible, and it has allowed us to count the number of eggs and calculate the hatching rate of larvae by each individual. The discovery of SIP is expected to provide the technology needed for stable bivalve hatcheries and lead to the development of new bivalve aquaculture.

### [1S03a-3]

### Age-related alternation in the intracellular localization of MC4R proteins: a novel mechanism of age-related obesity

*Manami Oya[1], Kazuhiro Nakamura[1] ([1]*Nagoya University*)

Obesity is a threat to global health, as it increases the risk of metabolic disorders, such as diabetes and cardiovascular diseases. Although it is well-known that susceptibility to obesity increases with age, the mechanism of age-related obesity is unknown. The melanocortin-4 receptor (MC4R) is a key molecule in the central neural circuit that regulates food intake and energy expenditure. Previous studies have reported that Mc4r mRNA is expressed in the dorsomedial hypothalamus (DMH) and paraventricular hypothalamic nucleus (PVH), which are important nuclei for the regulation of energy balance. Hypothalamic MC4Rs mediate anorexigenic leptin-melanocortin signaling, and mice and humans with MC4R gene mutations exhibit hyperphagia and severe obesity. However, alterations in the intracellular dynamics of MC4R proteins during aging have not been studied due to the unavailability of anti-MC4R antibodies applicable to immunocytochemical detection of endogenous MC4R proteins. Here, we generated an anti-MC4R antibody and performed immunostaining in rat brains of various ages. We discovered that MC4Rs are localized to immotile primary cilia, antenna-like organelles, of DMH and PVH neurons. Furthermore, MC4R-bearing primary cilia of these hypothalamic neurons were progressively shortened with age, in correlation with age-dependent metabolic decline and increased adiposity. In contrast, the length of MC4R-negative primary cilia did not change. Age-related shortening of MC4R-bearing primary cilia was promoted by overnutrition-induced upregulation of leptin–melanocortin signaling and inhibited by dietary restriction. Forced shortening of MC4R-bearing primary cilia of DMH and PVH neurons in young MC4R-Cre knock-in rats, which otherwise had long MC4R-bearing primary cilia, impaired neuronal sensitivity to melanocortin and resulted in decreased metabolism and increased appetite, leading to increases in body weight and body fat. These results indicate that age-related shortening of MC4R-bearing primary cilia impairs leptin–melanocortin signaling and thereby increases adiposity, providing a novel mechanism for age-related obesity.

### [1S03a-4]

### Hormonal abnormalities in the thrifty phenotype model rats due to fetal undernutrition

*Takahiro Nemoto[1] ([1]*Dept. Physiology, Nippon Medical School*)

Undernutrition during the embryonic period is thought to result in small body size and thrifty phenotype by altering the metabolic and endocrine systems. The thrifty phenotype is thought to be advantageous for survival if the environment after birth is poor nutrition status, but if exposed to overnutrition, it is thought to increase the risk of disease development. We have studied using offspring from dams fed a low-carbohydrate, calorie-restricted diet throughout pregnancy, because the mechanism is not clear. The offspring were born with low birthweight, and the model rats exhibited endocrinological abnormalities in decreased basal blood IGF-1 levels, and elevated corticosterone after restraint exposure, and these abnormalities were transgenerated to the next generation. When the model rat was exposed to starvation, the rate of weight loss after 24 hours was lower than controls, and the weight recovery rate after 48 hours of re-feeding was also lower than controls. Analysis of tissue weight suggested that the model rat have “Hard-to-Burn Fat” and “Easy-to-Lose & Hard-to-Gain Muscle”. We found that the expression of the IGF-1–miR-1–Pax7 axis, which is involved in skeletal muscle differentiation, is dysregulated in model rats, and that the expression of triglyceride-degrading enzymes such as ATGL and HSL is decreased in adipose tissues. Furthermore, nutritional intervention using a methyl modulator diet immediately after birth partially normalized glucocorticoid feedback dysregulation after exposure to restraint, and also restored changes in fat mass and muscle mass after fasting and refeeding. In this symposium, we will discuss “Hard-to-Burn fat” and “Easy-to-Lose & Hard-to-Gain Muscle” which are acquired through fetal undernutrition.

### [1S03a-5]

### Exploration of Unknown Peptide Hormones Acting on Stem Cells

*Masayasu Kojima[1] ([1]*Institute of Life Science, Kurume University*)

LGR4-6, which are stem cell markers, are essential for maintaining the normal function of the skin and gastrointestinal mucosa. They have also been implicated in the development of skin cancers such as basal cell carcinoma and squamous cell carcinoma, as well as their association with "cancer stem cells" in digestive tract cancers. While structurally belonging to the G-protein-coupled receptor (GPCR) family, the endogenous ligands that bind to LGR4-6 are still unknown, leaving the role of LGR4-6 in stem cells not fully understood. Previously, we discovered the hormone ghrelin as the endogenous ligand for GHSR, which was then an orphan GPCR. Subsequently, when searching for orphan GPCRs to target for the next ligand discovery, I came across a paper on LGR4-6 as stem cell markers. Learning that LGR4-6, despite being markers for stem cells, were orphan GPCRs with unknown ligands, piqued my interest in discovering their endogenous ligands. They attempted to search for endogenous ligands from tissue extracts but did not succeed in identification. LGR4-6 share similar structural characteristics, forming a family of three receptors. Their structure is similar to receptors for follicle-stimulating hormone (FSH), luteinizing hormone (LH), and thyroid-stimulating hormone (TSH) - LGR1-3, and also to the relaxin receptors LGR7-8. Given the high structural homology of receptors, it is likely that the as-yet unidentified endogenous ligands for LGR4-6 are also peptide hormones. Furthermore, since both LGR1-3 and LGR7-8 couple with Gs proteins, it is predicted that LGR4-6 also couple with Gs, leading to the production of cAMP as a second messenger upon receptor activation. In 2011, it was reported that R-spondin binds to LGR4-6. However, it was noted that R-spondin alone cannot stimulate cAMP production via LGR4-6, and it cannot structurally convert the receptor into its active form. Hence, it is believed that molecules other than R-spondin must bind to the receptor for LGR4-6 to be converted into their active form to transmit signals. In 2013, Norrin was reported to bind to LGR4-6, and in 2016, RANKL was also reported to bind to them. Nevertheless, neither Norrin nor RANKL alone can activate LGR4-6 and transmit signals, as cAMP levels do not change upon their binding. Thus, as of now, the endogenous ligand that can convert LGR4-6 into an active receptor is still unknown. This study aims to introduce our attempts to search for the endogenous ligand of LGR4-6, which has not been discovered yet.

### Symposium

[1S04a]
International Relations Committee
**Cutting-edge research of transporters and pumps: Structure, function and physiological significance**

March 28, 14:20 - 16:20, Room 4

### [1S04a-1]

### Physiological and Pathophysiological functions of intracellular ATPases

*Takuto Fujii[1], Takahiro Shimizu[1], Hideki Sakai[1] ([1]*Department of Pharmaceutical Physiology, Faculty of Pharmaceutical Sciences, University of Toyama*)

P-type ATPases, such as sodium pumps ($Na^+$,$K^+$-ATPases), gastric proton pump ($H^+$,$K^+$-ATPase), and $Ca^{2+}$ pumps ($Ca^{2+}$-ATPases), play crucial roles in the maintenance of electrochemical gradients and cellular homeostasis. Dysregulation in the expression and function of these ATPases has been reported to be associated with several diseases, including cancer and neurodegenerative disorders. Here, we report the physiological and pathophysiological functions of two intracellular P-type ATPases: $Na^+$,$K^+$-ATPase α3-isoform (α3NaK) in cancer cells and ATP13A2 (also called PARK9) in neurons. α3NaK is normally expressed in the plasma membrane of neurons, and skeletal and cardiac muscle. We found the aberrant expression of α3NaK in the intracellular vesicles of various types of human cancer cells. Interestingly, α3NaK is translocated to the plasma membrane upon cancer cell detachment. The dynamic translocation of α3NaK to the plasma membrane is implicated in the survival of detached (metastatic) cancer cells. Inhibition of intracellular α3NaK by cardiac glycosides, potent inhibitors of $Na^+$,$K^+$-ATPase, can suppress the cancer cell growth and metastasis. On the other hand, ATP13A2 is associated with an autosomal recessive early-onset form of Parkinson's disease known as Kufor-Rakeb syndrome. Our recent study revealed that ATP13A2 functions as a novel $H^+$,$K^+$-ATPase in the lysosomes of neurons. ATP13A2 exhibits unique pharmacological properties; its $H^+$/$K^+$ transport activity is inhibited by potassium competitive acid blockers (P-CABs; SCH28080 and vonoprazan), and SERCA $Ca^{2+}$-ATPase inhibitor (thapsigargin). Mutations identified in the patients of Parkinson's disease result in decreased expression and activity of ATP13A2. Dysfunction of ATP13A2 causes alkalinization and α-synuclein accumulation in the lysosomes of the neuronal cells, which are pathological hallmarks of Parkinson's disease. These our findings suggest new functions of intracellular P-type ATPase in refractory diseases.

### [1S04a-2]

### Deep learning-driven *de novo* drug design based on gastric proton pump structures

*Kazuhiro Abe[1] ([1]*Nagoya University*)

Existing drugs often suffer in their effectiveness due to side effects, low binding affinity or pharmacokinetic problems. This may be overcome by the development of novel compounds. Here, we exploit the rich structural basis of drug-bound gastric proton pump to develop novel compounds with strong inhibitory potency, employing a combinatorial approach utilizing deep generative models for *de novo* drug design with chemical synthesis and cryo-EM structural analysis. Candidate compounds that suffice pharmacophores defined in the drug-bound proton pump structures, were designed *in silico* using deep generative models, a workflow termed Deep Quartet. Several candidates were synthesized and screened according to their inhibition potencies *in vitro*, and their binding poses were in turn identified by cryo-EM. Structures reaching up to 2.10 Å resolution allowed us to evaluate and then fine-tune the compound structure, with subsequent re-design, heralding the most potent compound, DQ-18 (*N*-methyl-4-((2-(benzyloxy)-5-chlorobenzyl)oxy) benzylamine), which shows an $K_i$ value of 47.6 nM. Further high resolution cryo-EM analysis at 2.04 Å resolution unambiguously determined the DQ-18 binding pose. Our results showcase that machine learning based on the desired pharmacophores within the protein structure can overcome existing barriers to the development of novel drugs that are superior to existing ones.

### [1S04a-3]

### Structural Analysis Reveals the Biogenesis and Functions of a Heterodimeric Amino Acid Transporter

*Pattama Wiriyasermkul[1,2], Shushi Nagamori[1] ([1]*The Jikei University School of Medicine*, [2]*Iwate University*)

Amino acids enter the body through amino acid transporters. Heterodimeric amino acid transporters (HATs) exist at plasma membranes of cells and make a family of amino acid transporters which comprise two subunits: the amino acid transporters as the catalytic subunit and the glycoproteins for plasma membrane translocation. At present, two glycoproteins (rBAT and CD98hc) and 7 transporters have been characterized as HAT members. The rBAT-transporter complexes are located at the apical membrane while the CD98hc-transporter complexes are found at the basolateral side, suggesting the importance of glycoproteins for cell-polarized localization. In collaboration with structural biologists, we have solved and analyzed structures of CD98hc-LAT1 and rBAT-$b^{0,+}$AT. The rBAT-$b^{0,+}$AT structure, unlike CD98hc-LAT1, contains $Ca^{2+}$-binding sites on rBAT and forms super-dimer (dimer x dimer). We found that $Ca^{2+}$ in the endoplasmic reticulum stabilizes rBAT interface and mediates rBAT-$b^{0,+}$AT super-dimerization. This is a key step for protein quality control acquisition and protein trafficking to be functional at the plasma membrane. Regarding the transporter subunits, we analyzed the key substrate binding sites and substrate selectivity by using biochemical assays based on the structure information. Our studies light up the mechanisms of HATs biogenesis and functions and serve as a guide for translational medicine targeting the HATs.

### [1S04a-4]

### Structure and Function of the CNNM/CorC Family Magnesium Transporter

*Motoyuki Hattori[1] ([1]*School of Life Sciences, Fudan University*)

The CNNM/CorC family proteins are $Mg^{2+}$ transporters that are widely distributed in all domains of life. In bacteria, CorC has been implicated in the survival of pathogenic microorganisms in their host environment and in resistance to antibiotic exposure.
In humans, CNNM proteins are involved in a variety of biological events, including hypertension, various genetic disorders, and tumor progression. Accordingly, both CorC and CNNM have attracted interest as therapeutic targets. However, their $Mg^{2+}$ transport mechanism is unclear due to the lack of structural information. In this talk, I will present our recent structural and functional analyses of the CNNM/CorC family proteins and discuss the structure-function relationship between $Mg^{2+}$ transport and human genetic diseases. In addition, I will introduce the application of AlphaFold2 to the analysis of structural dynamics of membrane proteins.

### Symposium

[1S05a]
### Ultrasound interrogation and control physiological functions

March 28, 14:20 - 16:20, Room 5

### [1S05a-1]
### Recent advances in ultrasound technologies for biomedical research

*MASAFUMI SHIMOJO[1] ([1]*National Institutes for Quantum and Radiological Science and Technology*)

Ultrasound has become a fundamental biophysical tool for the visualization and manipulation of living organisms. Among a variety of modalities, ultrasound especially has superior advantages regarding its non-invasiveness, tissue permeability, and spatiotemporal resolution, offering multidisciplinary utilities of *in vitro* and *in vivo* systems for studying biology and medicine. In the last decade, ultrasound has also made innovative breakthroughs in the areas of neuroscience. For instance, current state-of-the-art functional ultrasound imaging enables to monitor whole-brain vasculature dynamics in response to brain activation. On the other hand, ultrasound neuromodulation technology in combination with genetics and/or drug engineering now provide a new possibility for controlling specific brain circuit of animal model and humans with excellent spatiotemporal resolution. In this session, we introduce recent advances in ultrasound technologies and discuss the potential applications for future biological research and biomedical engineering.

### [1S05a-2]
### Molecular mechanisms of intrinsic ultrasound-responsive ability in mouse cortical neurons

*Yumi Matsushita[1] ([1]*National Institutes for Quantum Science and Technology, Institute for Quantum Medical Science, Department of Functional Brain Imaging*)

Neuromodulation utilizing a variety of modalities is a fundamental biomedical approach for modulating the function and dysfunction of brain neuronal circuits in living laboratory animals and humans. Ultrasound has recently become an innovative tool for neuromodulation owing to non-invasiveness, superior tissue penetration, and high spatiotemporal resolution. These advantages of ultrasound offer a variety of potential applicability in translational neuroscience research and biomedical engineering.
Despite the benefit of ultrasound in neuromodulation being definitive, the molecular and cellular mechanisms by which ultrasound intervenes in the neuronal activity of the brain remain uncertain. To clarify this, we established a fluorescence microscopes-based live cell imaging system in combination with an ultrasound transducer and investigated how ultrasound irradiation affects intracellular calcium dynamics in neurons. Pulsed ultrasound irradiation (1 MHz, 500 msec) to cultured mouse cortical neurons expressing GCaMP6s, a genetically encoded fluorescent calcium indicator, rapidly induced increase of intracellular calcium concentration. Pharmacological experiments demonstrate that neuronal responses triggered by ultrasound irradiation depend on calcium influx through ion channels on the plasma membrane, followed by the generation of action potentials and synaptic transmission. Remarkably, inhibitors against mechanosensitive channels abolished the ultrasound-induced calcium responses in neurons, suggesting that the activation of mechanosensitive channels can be an initial trigger of neuronal ultrasound responses. Our findings indicate that mechanosensitive channels are predominantly involved in intrinsic ultrasound responses in neurons of mammalian brains, and further investigation to dissect detailed biological consequences underlying ultrasound neuromodulation is still in progress.

### [1S05a-3]
### A closed-loop sonogenetic control of absence epilepsy

*Kaede Yoshida[1], Nobuki Kudo[1], Masabumi Minami[1], Yuichi Takeuchi[1] ([1]*Hokkaido University*)

Epilepsy is a neurological disorder characterized by recurrent and unprovoked seizure occurrences with loss of consciousness and/or convulsions. It affects approximately one in every hundred individuals. Currently, 70% of patients can be controlled with pharmaceutical treatment; however, the remaining 30% are drug resistant. The refractory population may eventually undergo surgical resection of the seizure foci, which comes with some risk due to its invasiveness, irreversibility and possible side effects such as cognitive dysfunctions. Deep brain stimulation has been employed to the refractory population, in which stimulus electrodes go into deep brain regions; however, this is also invasive and may lead to complications such as bleeding and infections. Sonogenetics is a newly developed non-invasive brain stimulation technology that combines ultrasound and mechano-sensitive ion channels. The bio-permeability of ultrasound enables its transcranial irradiation, whereas the adeno-associated virus-mediated transduction of mechanosensitive channels allows cell-type specific control of neurons. Here we have transduced a sonogenetic actuator, a bacteria-derived mechano-sensitive ion channel, eMscL$^{G22S}$ to cerebral neurons of mice and rats via intravenous administration of an engineered serotype of AAV vector for highly efficient transduction to the central nervous system. First, we confirmed that eMscL$^{G22S}$ enhanced sensitivity of cerebral neurons to ultrasound using c-Fos immunohistochemistry and *in vivo* multi-unit electrophysiological recordings in anesthetized mice. We then implemented a closed-loop transcranial ultrasound irradiation system and found that time-targeted ultrasound irradiation immediately terminated seizures of eMscL$^{G22S}$ transduced rat model of absence epilepsy in an awake preparation. In this talk, recent development of sonogenetic technologies will also be refer to.

### [1S05a-4]

### Development of Ultrasound Control Method for Microbubble-Encapsulated Vesicles and Its Medical Applications

*Shu Takagi[1] ([1]*The University of Tokyo*)

The authors have been developing techniques for encapsulating microbubbles of about 2 µm in diameter inside vesicles of 5 µm or less in diameter, which can be used in microbubble drug delivery systems, and a method for controlling their position using ultrasound. In the present talk, the methods of generating Microbubble-Encapsulated Vesicle (MEV) are explained. As one of the methods, we used flow focusing type microchannels to generate MEVs. In this method, controlling of the flow rate of water and oil phases are important to have the small size vesicles with the microbubble encapsulated. Then, the generated MEVs are manipulated using the ultrasound array transducers. The phase control methods are employed, and the bubbles are successfully manipulated using ultrasound, which also makes the MEVs manipulated with bubble movement. Further analysis will be shown in the presentation.

### [1S05a-5]

### Therapeutic Prospects of Gene Delivery using Ultrasound and Nanobubbles

*Hiroshi Kida[1], Katsuro Tachibana[1] ([1]*Department of Anatomy, School of Medicine, Fukuoka University*)

Acoustic cavitation is a phenomenon in which a bubble nucleus in a liquid grows while vibrating in resonance with ultrasound irradiation and finally collapses. When combined with focused ultrasound surgery (FUS), the irradiation site can be precisely controlled in millimeters from outside the body, making it an excellent drug delivery system (DDS) with spatial selectivity. Traditionally, microbubbles (bubbles 1-100 µm in diameter) have been widely used in these studies, but recently even finer nanobubbles (bubbles less than 1 µm in diameter) have also been used. Compared to microbubbles, nanobubbles can reach deeper into living tissue, float extremely slowly, and stay in place for longer periods of time. We have established a method for generating high concentrations of albumin-shelled nanobubbles (A-NBs), clarified their stability, low cavitation threshold, and the relationship between particle size and ultrasound responsiveness, and reported selective and efficient transfer of DNA and mRNA into mammalian cell lines and animal tissues. Furthermore, we have demonstrated that gene transfer is possible with low-power and low-frequency ultrasound using A-NBs and are developing a device for application to mRNA vaccines. Currently, research is being conducted worldwide for therapeutic applications of ultrasound and nanobubbles for various malignant tumors and central nervous system diseases. In this presentation, we will introduce the trends of these studies as well as our research results.

### Symposium

[1S06a]
Editorial Board of the Journal of Physiological Sciences
**New insights into itch research**

March 28, 14:20 - 16:20, Room 6

### [1S06a-1]

### The contrasting roles of orexin neurons in itch and pain neural processing

*Tatsuroh Kaneko[1], Asuka Oura[1], Ikue Kusumoto-Yoshida[1], Takuro Kanekura[2], Hiroyuki Okuno[3], Tomoyuki Kuwaki[1], Hideki Kashiwadani[1] ([1]*Department of Physiology, Graduate School of Medical and Dental Sciences, Kagoshima University,* [2]*Department of Dermatology, Kagoshima University Graduate School of Medical and Dental Sciences,* [3]*Department of Biochemistry and Molecular Biology, Graduate School of Medical and Dental Sciences, Kagoshima University*)

Pain and itch are aversive sensations but induce different defense responses to protect our bodies from external stressors. Pain evokes a withdrawal response to avoid further damage, whereas itch causes a scratching behavior to remove irritants from the skin surface. These two sensations are recognized as antagonistic sensations; pain suppresses itch, whilst pain inhibition enhances itch.
While most previous studies have focused on the neural mechanisms at the spinal cord level underlying these pain-itch interactions, the role of supraspinal regions in processing these interactions still needs to be explored. Moreover, the question remains regarding why these two sensations are antagonistically regulated in relation to each other.
To pave the way for this research area, we have focused on orexin (ORX) producing neurons in the lateral hypothalamus (LH), which is known as a master switch that induces various “defense responses” when animals face a stressful environment.
Here, we revealed the contrasting role of ORX neurons, the inhibition of which suppressed itch while enhancing pain neural processing, by applying ORX-neuron-ablated (ORX-abl) mice and an optogenetic approach to the acute pruritus and pain model.
In addition, most ORX neurons responded to both pain and itch input, indicating that the same ORX neurons oppositely regulate pain and itch processing, which could be understood as defense responses against external stressors.
We also revealed that the circuit of ORX neurons from LH to periaqueductal gray regions served in the contrasting modulation of itch and pain processing using optogenetic terminal inhibition techniques.
Lastly, by applying ORX-abl mice to an atopic dermatitis model, we confirmed the involvement of ORX neurons in regulating chronic itch processing, which could lead to a novel therapeutic target for persistent pruritus in clinical settings.
Our findings provide a new angle to understanding the biological meaning of why these two sensations are antagonistically regulated to each other in the central nervous system, from the concept of defense responses triggered by ORX neurons.

### [1S06a-2]

### Possible involvement of central and peripheral PACAP-PAC1 receptor signaling pathways in itch transmission

*Takashi Kurihara[1] ([1]*Department of Pharmacology, Graduate School of Medical and Dental Sciences, Kagoshima University*)

Pituitary adenylate cyclase-activating polypeptide (PACAP) is a pleiotropic neuropeptide, belonging to the vasoactive intestinal polypeptide (VIP)/secretin/glucagon superfamily. PACAP receptor consists of three distinct G-protein-coupled receptors: the PACAP-specific (type 1: PAC1) receptor, and the PACAP/VIP-indifferent receptors, VPAC1 and VPAC2 receptors. Previously, we demonstrated that a single intrathecal (it) injection of PACAP or maxadilan, a PAC1 receptor specific agonist, induced transient aversive behaviors (licking/biting/scratching directed toward the caudal part of body for several hours) followed by a long-lasting mechanical allodynia in mice, suggesting that spinal PACAP-PAC1 receptor system plays an important role in the modulation of spinal nociceptive transmission and induction of chronic pain. However, involvement of PACAP-PAC1 receptor system in itch transmission is entirely unclear. In this study, we explored possible involvement of itch-like components in the PAC1 receptor-evoked aversive responses and evaluated the importance of PACAP-PAC1 receptor signaling in several mouse itch models.
Both intradermal (id) and it injection of PACAP (1 pmol–1 nmol) dose-dependently elicited licking/biting/scratching behaviors, and these behaviors were inhibited by subcutaneous pretreatment with the μ-opioid receptor antagonist naltrexone (1 mg/kg). The aversive behaviors induced by id and it PACAP were inhibited by id and it co-injection of recently discovered small-molecule PAC1 receptor antagonists (PA-8 and PA-9, 0.01-1 nmol), respectively. We further found that it, but not id, pretreatment of the PAC1 receptor antagonists (1 nmol) attenuated peripheral chloroquine-, compound 48/80- and 5-HT-induced itch-like behaviors. Single oral administration of the PAC1 receptor antagonists (3-30 mg/kg) dose-dependently suppressed itch-associated behaviors in dry skin, DNFB (dinitrofluorobenzene)-induced contact dermatitis and imiquimod-induced psoriasis-like model mice. In addition, the development of 5-HT and DNFB-induced itch-like behaviors was markedly suppressed in mice lacking PACAP.
These results suggest that PACAP-PAC1 receptor signaling in the spinal cord and skin is involved in an important mechanism underlying the itch-like behaviors, and blocking PAC1 receptor system may be a new strategy to manage itch sensation in skin diseases.

### [1S06a-3]

### Neuro-immune interaction shapes the pathophysiology of chronic inflammation - Pathogenic CD4$^+$ T (Tpath) cells induce pathological itch -

*Kiyoshi Hirahara[1] ([1]*Department of Immunology, Graduate School of Medicine, Chiba University*)

The interconnection of different biological systems, such as the immune and nervous systems, is involved in shaping the pathophysiology of various chronic inflammation. But the detailed molecular and cellular mechanisms remain largely unknown. In this lecture, I will introduce a new pathogenetic mechanism that induce chronic itch via "neuro-immune interaction" in chronic allergic inflammatory diseases. By analyzing a mouse model of chronic allergic conjunctivitis and patient samples of severe allergic conjunctivitis, we found that ectopic lymphoid tissue called conjunctiva-associated lymphoid tissue (CALT) was formed in the inflamed conjunctiva, and peripheral nerves were elongated around CALT structure. We identified pathogenic CD4$^+$ T (Tpath) cells that induce "pathological itch" by single cell RNA-sequencing using inflamed conjunctiva from both mouse and human. To develop new therapeutic strategies for intractable allergic diseases, it will become increasingly important to understand the precise features of neuro-immune interaction.
Reference: 1. Okano, M., Hirahara, K., Kiuchi, M., Onoue, M., Iwamura, C., Kokubo, K., Hishiya, T., Morimoto, Y., Ikehara, Y., Murakami, A., Ebihara, N., and Nakayama, T.: Interleukin-33-activated neuropeptide CGRP-producing memory Th2 cells cooperate with somatosensory neurons to induce conjunctival itch. **Immunity** 55(12):2352-2368.e7 (2022). 2. Sato, Y., Silina, K., van den Broek, M., Hirahara, K., and Yanagita, M.: The roles of tertiary lymphoid structures in chronic diseases. **Nat. Rev. Nephrol.** Aug;19(8):525-537 (2023).

### [1S06a-4]

### Itch transmission in the spinal cord and its alterations by chronic dermatitis

*Makoto Tsuda[1] ([1]*Department of Molecular and System Pharmacology, Graduate School of Pharmaceutical Sciences, Kyushu University*)

Itch is an unpleasant sensation that elicits the desire or reflex to scratch and normally serves as a self-protection mechanism from harmful external agents such as chemicals and parasites. The discovery of the itch-selective role of gastrin-releasing peptide receptor (GRPR)-expressing neurons in the spinal dorsal horn (SDH) revealed the presence of an itch-selective neural pathway and made great progress in our understanding of the neural circuits for itch transmission. Under pathological conditions such as atopic dermatitis, however, itch becomes more severe and chronic, which leads to excessive, repetitive scratching. By focusing on pathological changes in the nervous system, we identified two factors that enhance the excitability of GRPR+ neurons and play a critical role in chronic itch. First, we found that astrocytes, a type of glial cells, become activated in the SDH of mouse models of chronic dermatitis and that the astrocytic factor lipocalin-2 (LCN2) enhances the excitatory effect of GRP on activity of GRPR+ neurons. Second, we also found that expression of neuronal pentraxin 2 (NPTX2), an activity-dependent immediate early gene product, is upregulated in the dorsal root ganglion neurons of mice with chronic dermatitis and that DRG neuron-derived NPTX2 is necessary for a facilitation of glutamatergic inputs onto GRPR+ neurons. In my talk, I will show a current model of itch transmission and its alteration by chronic dermatitis, and will also discuss a drug discovery strategy for managing chronic itch.

### Symposium

[1S08a]

**Unraveling the Mechanisms of Electrical Signal Conversion in Ion Channels and Related Proteins**

March 28, 14:20 - 16:20, Room 8

### [1S08a-1]

### Analysis of the role of interactions between voltage-sensing phosphatase (VSP) and PI(4,5)$P_2$ in electro-chemical signal transduction of VSP

*Natsuki Mizutani[1], Yasushi Okamura[1] ([1]*Integrative Physiology, Graduate School of Medicine, Osaka University, Suita, Japan*)

Voltage-sensing phosphatase (VSP) is a unique membrane protein in which a voltage sensor domain (VSD) regulates an enzyme activity. Through tight coupling between the VSD and the cytoplasmic catalytic region (CCR), VSP exhibits the voltage-dependent phosphoinositide phosphatase activity against mainly PI(4,5)$P_2$. The CCR of VSP has a remarkable similarity to phosphatase and tensin homolog (PTEN). In PTEN, PI(4,5)$P_2$ is not a substrate but thought to bind to basic residues in the N-terminal PI(4,5)$P_2$-binding motif (PBM), enhancing phosphatase activity. Interestingly, PI(4,5)$P_2$ is also suggested to modulate VSD motion and coupling in VSP through the PBM-like region in the C-terminal half of the linker. However, it has not yet been elucidated whether PI(4,5)$P_2$ binds to this region. Our previous study using voltage clamp fluorometry reported that a type of fluorescent unnatural amino acid, called Anap, could detect interaction of *Ciona intestinalis* VSP (Ci-VSP) with PI(4,5)$P_2$ in *Xenopus* oocytes. In this study, we tested incorporation of Anap into the PBM-like region (K252, R253, and R254) and its vicinities of Ci-VSP to examine its PI(4,5)$P_2$ interaction, and compared Anap fluorescence signals evoked by membrane depolarization between in the presence and absence of pre-depolarization which mimic low and normal PI(4,5)$P_2$ levels on the plasma membrane. We found that only Y255 exhibited drastic kinetic changes in the fluorescence signals, suggesting that there is interaction between the linker and PI(4,5)$P_2$. As the Anap mutants of K252, R253, and R254 showed weak phosphatase activity toward PI(4,5)$P_2$, we are currently conducting experiments to analyze the signal kinetics of Anap in a reduced PI(4,5)$P_2$ environment induced by GPCRs and other methodologies. Our results might provide insight into molecular mechanisms of VSP where PI(4,5)$P_2$ is not only a substrate but a regulation factor.

### [1S08a-2]

### Identification of novel drugs targeting GIRK channel and the modulatory mechanism of drug action

*I-Shan Chen[1,2], Jumpei Yasuda[1], Yoshihiro Kubo[2], Tomoe Nakamura-Nishitani[1]
([1]*Wakayama Medical University,* [2]*National Institute for Physiological Sciences*)

G-protein-gated inwardly rectifying $K^+$ (GIRK) channel (GIRK1, GIRK2, GIRK3, GIRK4 subunits) are expressed in various tissues and are involved in the regulation of membrane excitability. We previously reported a novel GIRK channel activator, ivermectin (a well-known antiparasitic drug), and a novel inhibitor, terfenadine (an antihistamine). Site-directed mutagenesis analysis revealed that the side helix connecting transmembrane domain 1 (TM1) to N-terminus of GIRK2 is responsible for ivermectin-mediated activation, while the pore helix connected to the TM1 and selectivity filter of GIRK1 is responsible for terfenadine-mediated inhibition. Recently, we newly identified novel modulators of GIRK channels from ingredients of licorice, which is commonly used as a food additive and a Chinese medicine. We found that a major ingredient of licorice, glycyrrhizic acid (GA), inhibits the GIRK heterotetramers which contain GIRK1 subunits. On the other hand, the metabolite of GA, 18β-glycyrrhetinic acid (18β-GA), activates GIRK channels. Recent studies suggest that cardiac type GIRK channels (GIRK1-GIRK4 heterotetramers) are constitutively activated in patients with chronic atrial fibrillation. Since excess intake of licorice is also known to induce atrial fibrillation, we hypothesized that high concentrations of 18β-GA may affect atrial function via increasing GIRK channel activity. By motion analyses of myocyte contraction of rat atrial myocytes, we observed that 18β-GA decreases spontaneous beating of cardiomyocytes via activation of GIRK activity. We next investigated the modulatory mechanism of 18β-GA on GIRK channels and found that the Glu residue located at the cytoplasmic pore of GIRK channel is important for the interaction with 18β-GA. The pore helix residue Ser148 in GIRK2 also contributed to 18β-GA-mediated activation, and the corresponding Phe137 in GIRK1 was critical for GA-mediated inhibition. Furthermore, we found that $PIP_2$ is essential for 18β-GA-mediated GIRK activation, whereas the coupling of $G_{\beta\gamma}$ to the channel is not critical. In conclusion, we identified multiple drugs that activate or inhibit GIRK activity, but the structural features of the channels crucial for their modulatory mechanism differ. These results would provide clues to elucidate novel gating mechanisms of GIRK channels by drugs.

### [1S08a-3]

### Creation of Light-Driven Receptor Channels and Measurement of Structural Changes

*Yuichiro Fujiwara[1,2], Maiko Hirano[2], Kazuyo Kamitori[2] ([1]*Physiology and Biophysics, Graduate School of Biomedical and Health Sciences, Hiroshima University,* [2]*Molecular Physiology & Biophysics, Faculty of Medicine, Kagawa University*)

In recent years, light-driven channels such as algal channelrhodopsin have been studied as a tool for analyzing neural circuit function as optogenetics. Efforts have been made to find new light-driven rhodopsins from prokaryotes that are easier to use. On the other hand, there have been attempts to artificially create light-driven channels, but it is not easy. In this study, we attempted to create light-driven ion channels by cross-linking azobenzene derivatives, which are isomerized by light irradiation into cis- (5-12 Å) and trans- (19 Å) forms under UV and blue visible light, respectively. Cys-introduced insect fructose receptor channel mutants were expressed in *Xenopus* oocytes, cross-linked with azobenzene di-maleimide, and analyzed their ionic currents while being exposed to light. Several ion channel mutants that opened and closed in response to light irradiation were obtained. As the second objective of this study, an open/close structural model of the fructose receptor channel was created based on the positional information on the protein structure of the obtained Cys mutants and the distance information changed by azobenzene isomerization. In this presentation, we would like to discuss the conformational change by ligand binding and the dynamic conformational change of the channel where the ion permeation pathway opens.

### [1S08a-4]

### Structure-guided approach to elucidate modulation of voltage-gated potassium channels by auxiliary subunits

*Koichi Nakajo[1], Go Kasuya[1] ([1]*Division of Integrative Physiology, Department of Physiology, Jichi Medical University*)

Voltage-gated ion channels are essential membrane proteins for generating electrical signals in excitable cells. The activation mechanisms of voltage-gated potassium channels have been intensively studied since the first cloning of the Shaker $K^+$ (Kv1) channel in 1987, and it is now well understood that upward movement of the fourth segment (S4) of the voltage-sensing domain (VSD; S1-S4) triggers the opening of the pore domain. While the voltage-gated potassium channel genes represent one of the largest subfamilies of ion channels, their auxiliary subunits add to the diversity of their physiological functions and roles. Some auxiliary subunits are transmembrane proteins that bind directly to the VSD and alter its gating properties. For example, KCNE proteins bind directly to the VSD of KCNQ1 channels, altering their gating properties. Recent series of cryo-EM structures provide a clearer view of how these auxiliary subunits bind to the VSD. Taking advantage of the structure of these ion channel complexes, it is now possible to study how the binding of auxiliary subunits can modulate channel gating. Here, we will present our recent results on KCNQ1 and KCNE proteins. We noticed that KCNE3 binds to the S1 segment in the cryo-EM structure of the KCNQ1-KCNE3 complex. By introducing systematic mutations and applying voltage clamp fluorometry, we investigated the functional role of the interaction surface between S1 and KCNE3 proteins. We found that the interaction is tightly optimized, and that tight binding is necessary to stabilize the VSD in the intermediate (open) state. As a result, the KCNQ1-KCNE3 channel becomes a constitutively open channel. We also used the same strategy to study the KCNQ1-KCNE1 channel, which generates slowly activating potassium currents, also known as $I_{Ks}$. Interestingly, we found that KCNE1 also uses the S1 segment to stabilize the intermediate state. However, because the intermediate state in the KCNQ1-KCNE1 channel is non-conducting, it generates slowly activating currents as a result. Although the outputs of the KCNQ1-KCNE3 channel and the KCNQ1-KCNE1 channel appear to be opposite, they share a common mechanism for modulating VSD movement.

### [1S08a-5]

### Structural and functional bases of voltage dependent activation of KCNQ1 channels

*Jianmin Cui[1] ([1]*Washington University in St. Louis*)

The $I_{Ks}$ current controls action potential duration in the heart at various physiological conditions, and abnormal function of this current causes cardiac arrhythmias. $I_{Ks}$ is carried by the voltage activated KCNQ1 (Kv7.1) potassium channel associated with KCNE1 modulatory subunits. We found that in voltage dependent activation of KCNQ1 its voltage sensor domain (VSD) undergoes two steps of activation to the intermediate (I) and activated (A) states. The KCNQ1 pore opens when the VSD is at either the I or A state, and thus the channel has two open states, IO and AO. The IO and AO states show drastically different functional properties including voltage dependence, ion selectivity, pharmacology and regulation by KCNE1 β-subunits. This talk will present a structural and functional model of voltage dependent gating of KCNQ1 (Kv7.1) channels.

### Symposium

### [1S09a]
### Physiology-based understanding and overcome of congenital heart diseases

March 28, 14:20 - 16:20, Room 9

### [1S09a-1]
### Role of Physiological Assessment in the Treatment of Congenital Heart Disease

*Yasuhiro Kotani[1], Hiroaki Komatsu[1], Keiko Kaihara[2], Keiji Naruse[2], Gentaro Iribe[3], Shingo Kasahara[1] ([1]*Okayama University, Cardiovascular Surgery,* [2]*Okayama University, Cardiovascular Physiology,* [3]*Asahikawa Medical University, Physiology*)

Objective: Because of its unique anatomic and physiologic conditions, there are few evidence in the medical and surgical treatment for patients with congenital heart disease (CHD). Physiologic assessment may have a key role in understanding of changes during open heart surgery and postoperative course. We report our clinical and experimental experience of physiologic assessment in patients with CHD. Methods and Results: For clinical study, we reviewed 19 single ventricle patients who underwent a bidirectional cavopulmonary shunt (BCPS). SVC flow was measured at the time of BCPS by an ultrasonic flow probe. Mean indexed pre-BCPS SVC flow was 1.63±0.55L/min/m$^2$. In all but 1 patient, the SVC flow was increased to 1.99±0.57 L/min/m$^2$ after BCPS (p=0.005). There was a significant positive correlation between pre-BCPS and post-BCPS SVC flow (r=0.627, p=0.029). Pulmonary artery size correlated with post-BCPS SVC flow (r=0.560, p=0.016). Two patients with preoperative SVC flow of below 1.0 L/min/m$^2$ died or required BCPS takedown. SVC size did not correlate with BCPS flow (r=0.231, p=0.356). Univariate analysis indicated pre-BCPS pulmonary artery pressure was a risk factor for low arterial oxygen saturation (< 75%) immediately after BCPS (p=0.042) and at discharge (p=0.030).For experimental study, right ventricular myocardium resected from patients undergoing surgery for congenital heart disease was used. Single cardiomyocytes were enzymatically isolated from the tissue. To obtain force-length relationships for the cells, a pair of carbon fibers (CFs) were attached to each cell end and the cells were stretched in several steps under electrical stimulation of 0.5 Hz at 37°C. Cell length and tension were obtained from the distance between CFs and the amount of CF bending, respectively. The slope of the obtained end-systolic force-length relation and the end-diastolic force-length relation was used to evaluate cellular contractility and diastolic cellular stiffness, respectively.We performed 11 measurements and found that our method was reasonably accurate enough to extract expected mechanical property differences between mouse and human cardiomyocytes.Conclusions: Physiologic assessment in both clinical and experimental setting allows us to understand physiology in CHD patients undergoing surgical treatment and helps decision making process in the perioperative management.

### [1S09a-2]
### The molecular mechanisms of the ductus arteriosus-specific differentiation and transcriptional regulation

*Utako Yokoyama[1], Sayuki Oka[1], Kenta Kikuchi[2], Daisuke Kurotaki[2] ([1]*Tokyo Medical Univ.,* [2]*Kumamoto Univ.*)

The ductus arteriosus (DA) is essential for maintaining fetal circulation and immediately begins to close at birth when mammalian neonates transition from an aquatic to an atmospheric environment. This dynamic process involves DA-specific properties, including a highly differentiated phenotype of smooth muscle cells (SMCs), poorly formed elastic fibers in the tunica media, and prominent physiological intimal thickening. We and others have demonstrated that all these features are conferred by placenta-derived abundant prostaglandin $E_2$ ($PGE_2$) and play a coordinating role in the complete closure of the DA after birth. In contrast, it has been reported that patent ductus arteriosus (PDA) sometimes lacks these features in humans and other mammals. $PGE_2$ receptor EP4 is specifically expressed in DA SMCs and dilates the DA through the cAMP-protein kinase A pathway. We have shown that EP4 signaling contributes to intimal thickening formation by forming a hyaluronan, fibulin-1, and versican complex, and impairs elastic fiber formation via cAMP-independent pathways. Therefore, $PGE_2$-EP4 signaling plays critical roles in maintaining DA patency in utero and promoting phenotypic and structural remodeling of the DA for postnatal closure. However, the transcriptional regulation of EP4 during development is unknown. The DA originates from the 6$^{th}$ pharyngeal arch and is affected by cardiac neural crest cells. To investigate the spatiotemporal expression of EP4 during the fetal period, we generated EP4 reporter (*Ptger4*-IRES-nlsLacZ) mice. We found that EP4 began to be expressed specifically in the DA at embryonic day 14, but not in other parts of the cardiovascular system, and the expression levels of EP4 increased as development progresses. We performed RNA-seq, ATAC-seq, and CUT&Tag using the mouse DA and aorta and identified the candidate enhancer regions of *Ptger4*. The mutant mice, in which one of the candidate regions was deleted using CRISPR/Cas9, demonstrated a deficiency in EP4 expression in the DA and exhibited neonatal death due to PDA. These data indicate that there is the DA-specific transcriptional regulation independent of neural crest-derived cell-lineage.

### [1S09a-3]
### Osmotic pressure and patent arterial duct

*Yoshihiro Ishikawa[1], Utako Yokoyama[2] ([1]*Yokohama City University,* [2]*Tokyo Medical University*)

It's long been understood that significant changes occur in both serum components and haemodynamics at the moment of birth. These changes facilitate a rapid adaptation to life in an open-air environment. Among these changes is the closure of the ductus arteriosus, a vital shift that has been extensively studied. However, one intriguing aspect that hasn't been addressed is the transient decrease in serum osmolality observed in full-term human neonates after birth. This decrease in osmolality does recover over subsequent days, but the exact role of this phenomenon in relation to ductus arteriosus closure remains unknown. Therefore, the primary aim of our current study was to investigate the impact of these changes in serum osmolality on the closure of the ductus arteriosus. In our experimental setup, we observed that rats also experience a similar transient hypoosmolality following birth. When we subjected ductus arteriosus rings to hypotonic stimulation, there was a noticeable constriction, and an increase in calcium (Ca(2+)) transients was observed in ductus arteriosus smooth muscle cells—something not seen in the aorta. This led us to investigate the role of the transient receptor potential melastatin 3 (TRPM3), a known hypoosmotic sensor. We found that TRPM3 was highly expressed in rat ductus arteriosus tissue, and when silenced, the Ca(2+) response to hypoosmotic conditions was abolished. Stimulating TRPM3 with pregnenolone sulfate led to constriction of the rat ductus arteriosus both in ex vivo and in vivo conditions. Moreover, injecting hypertonic fluid disrupted the closure of the rat ductus arteriosus.Interestingly, in human subjects, we noticed that hypoosmolality was present in preterm infants who were relatively mature (≥28 weeks of gestational age). However, in extremely preterm infants (<28 weeks), this hypoosmolarity was absent. In these cases, a swift increase in osmolality was observed instead. Notably, this rise in osmolality was even more pronounced among patients with a patent ductus arteriosus. Our findings indicate that a transient decrease in serum osmolality may be an important factor that encourages ductus arteriosus closure during the early days of life. Therefore, regulating serum osmolality to appropriate levels could be a promising strategy to prevent the onset of patent ductus arteriosus, thus potentially improving therapeutic outcomes in extremely preterm infants.

### [1S09a-4]

### Understanding of Fontan circulation: the introduction of hemodynamic simulation

*Shuji Shimizu[1], Yasuhiro Kotani[2], Koji Uemura[1], Shingo Kasahara[2], Toshiaki Shishido[1]
([1]*National Cerebral and Cardiovascular Center,* [2]*Okayama University*)

**Objective:** Fontan operation is a surgical goal in the patients with single ventricular physiology. In the Fontan operation, both vena cava are anastomosed to the pulmonary artery. Therefore, the systemic circulation is serially connected to the pulmonary circulation and the whole circulation is maintained by a single ventricle. To understand this unique hemodynamics of Fontan circulation, a computational simulation may be helpful.
**Methods:** Our simulation model is based on the lumped parameter model. Cardiac chambers, a single atrium and a single ventricle, are described as the time-varying elastance model and systemic and pulmonary vasculatures are modeled as the modified Windkessel model.
**Results:** First, we investigated the effects of fenestration, an artificial shunt between a conduit for total cavopulmonary connection and a single atrium, on the failing Fontan circulation. Fenestration kept central venous pressure low even in the patients with high pulmonary vascular resistance index (PVRI). Next, we investigated the effects of systemic ventricular assist device (VAD) on the failing Fontan circulation. Systemic VAD improved the hemodynamics in the models with systolic/diastolic ventricular dysfunction or atrioventricular valve regurgitation. Finally, we examined the combined effects of systemic VAD and fenestration on the hemodynamics. The existence of fenestration reduced central venous pressure and stressed blood volume during systemic VAD support even in patients with elevated PVRI.
**Conclusions:** These findings suggests that computational simulation is helpful for the understanding of unique hemodynamics of Fontan patients. Furthermore, patients' specific simulation may be useful for the perioperative hemodynamic management.

### [1S09a-5]

### Ped-UT-Heart project supports precision medicine for congenital heart disease by providing detailed anatomical and functional information

*Seiryo Sugiura[1], Jun-ichi Okada[1,3], Takumi Washio[1,3], Isao Shiraishi[2], Toshiaki Hisada[1]
([1]*UT-Heart Inc.,* [2]*National Cerebral and Cardiovascular Center,* [3]*University of Tokyo*)

Congenital heart diseases (CHD) affecting approximately 1% of newborns constitutes a serious health problem. For cases of complex structural anomalies, surgical correction is the only cure, but the desirable outcomes can be achieved by not only the technical expertise but also the proper understanding of detailed anatomies and functional conditions of each patient. To facilitate the diagnosis and surgical planning by grasping the detailed anatomy, one of the authors (IS) has developed patient-specific heart replicas with real anatomy and texture by using 3D printing technology. To further empower this supporting system for heart surgery by adding functional information, we have launched a new project "ped-UT-Heart" using the UT-Heart technology. UT-Heart is a multi-scale, multi-physics heart simulator, in which electrophysiology, wall motion, and hemodynamics of the heart are reproduced based on the molecular function of cardiac excitation-contraction coupling process. In particular, we focus on the prediction of functional outcome after the treatment using the in virtual surgery. In each case, real (replica) and virtual (simulation) heart models in the pre-treatment state are created to accurately reproduce the anatomy and function. Then, under the guidance of cardiac surgeon, virtual surgery is performed to realize the post-operative state. By comparing the functional outcomes introduced by two to three alternative surgical plans, surgeons can choose the best strategy. The salient feature of multi-scale, multi-physics heart simulation is its ability to reveal the cellular and subcellular changes introduced by the surgical interventions. With those pieces of information, we hope we can support surgeons to further improve the treatment strategy, which ensures the life-long better QOL to the CHD patients.

### Symposium

### [1S10a]
**Behavioral and physiological regulations by the circadian clock**

March 28, 14:20 - 16:20, Room 10

### [1S10a-1]
**Neuropeptidergic inputs to the central circadian clock**

Chi Jung Hung[1], Tsai Chang-Ting[1], Sheikh Mizanur Rahaman[1], Akihiro Yamanaka[2], Wooseok Seo[3], Tatsushi Yokoyama[4], Masayuki Sakamoto[4], *Daisuke Ono[1] (*[1]Nagoya University, Research Institute of Environmental Medicine, [2]Chinese Institute for Brain Research, [3]Nagoya University, Graduate School of Medicine, [4]Kyoto University, Graduate School of Biostudies*)

Circadian rhythms are endogenous rhythms in physiology and behavior with a cycle length of approximately 24 hours. In mammals, the daily rhythms of these physiological functions are regulated by the central circadian clock located in the suprachiasmatic nucleus (SCN) of the hypothalamus. The SCN has retinal innervations via the retinohypothalamic tract to entrain circadian rhythms to environmental light-dark conditions. The circadian information in the SCN is then output to a variety of physiological functions such as sleep/wakefulness. Anatomically, it has been suggested that there are several neurons outside of the SCN that have inputs to the SCN, such as the intergeniculate leaflet, the median raphe nuclei, the lateral septal nucleus, the paraventricular thalamus, the preoptic areas, the ventromedial hypothalamus, the dorsomedial hypothalamus, and the lateral hypothalamus (LH). Although the multiple brain areas that innervate to the SCN have been anatomically reported, it is largely unknown which brain areas, which neuronal cell types, and which neurotransmitters influence circadian rhythms in the SCN. In this study, we identified that two neuronal populations, melanin-concentrating peptide (MCH)-producing and orexin-producing neurons, in the LH that have input to the SCN and regulate circadian periodicity in the SCN. When these neurons are ablated, the circadian behavioral rhythms are lengthened. Furthermore, when these peptides are applied to the culture medium, the circadian rhythms in the SCN slice are shortened and the levels of intracellular cAMP are decreased. We also found that these neurons innervate the SCN using anterograde and retrograde tracing. These results suggest that these neuropeptides in the LH play a role in the modulation of the circadian period in the SCN.

### [1S10a-2]
**The central circadian regulation of female reproductive functions**

*Takahiro J. Nakamura[1], Mizuki Sugiyama[1], Jiaxu Chen[1], Michihiro Mieda[2], Kazuto Watanabe[1], Wataru Nakamura[3] (*[1]Laboratory of Animal Physiology, School of Agriculture, Meiji University, [2]Department of Integrative Neurophysiology, Faculty of Medicine, Kanazawa University, [3]Department of Oral Chrono-Physiology, Graduate School of Biomedical Sciences, Nagasaki University*)

The central circadian clock of mammals is located in the suprachiasmatic nucleus (SCN) of the hypothalamus. The SCN regulates the circadian rhythms of various behavioral and physiological functions. In rodents, the luteinizing hormone (LH) surge that induces ovulation is time-dependent and occurs in the evening of proestrus. Additionally, SCN-lesioned rats and hamsters do not produce the LH surge. The projection pathway from the SCN to the gonadotropin-releasing hormone (GnRH) neurons that drive the LH surge in the hypothalamus has been studied. Still, the functional neural circuit that sends the timing information remains unclear. In the present study, we focused on the AVP, a neuropeptide abundant in the SCN, and conducted experiments using Avp-Cre mice. In mice where GABA release was specifically deficient in AVP neurons (*Avp-Cre; Vgat flox/flox* mice), disturbances in the estrous cycle, usually occurring for 4 or 5 days, were observed. Furthermore, the estrous cycle was disrupted when AVP neurons in the SCN were suppressed using optogenetic techniques just before the LH surge occurred during proestrus. However, this was not observed on days other than proestrus. Disturbances in the estrous cycle mean that regular ovulation is not occurring, and these results suggest that GABAergic transmission from AVP neurons in the SCN has important roles in female reproductive functions.

### [1S10a-3]
**Neural correlates of diurnal variation in the human suprachiasmatic nucleus**

*Akitoshi Ogawa[1], Satoshi Oka[1], Takahiro Osada[1], Masaki Tanaka[1], Koji Nakajima[2], Koji Kamagata[1], Shigeki Aoki[1], Yasushi Oshima[2], Sakae Tanaka[2], Eiji Kirino[1], Takahiro Nakamura[3], Seiki Konishi[1] (*[1]Juntendo University, [2]The University of Tokyo, [3]Meiji University*)

The suprachiasmatic nucleus (SCN) in the hypothalamus works as the central clock for circadian rhythms in mammals. While animal studies have revealed circadian neuronal activity in the SCN, the diurnal variation of the activity in the human SCN has remained elusive. In this study, the activity of the human SCN was examined every six hours within 24 hours in Experiment 1 (N = 27), and the human SCN activity in more detail during the night (every 30 min from midnight to 6:00) was examined in Experiment 2 (N = 20). The location of the SCN was identified using a boundary mapping technique applied to the resting-state images collected in our previous study. The SCN in each hemisphere was defined as the voxel above the optic chiasm and with the local minimum of the probabilistic boundary map. In this study, T1-weighted structural images and functional perfusion images were acquired using a 3-T MRI scanner at Juntendo University Hospital. The functional perfusion images were corrected for motion and distortion, and then the cerebral blood flow (CBF) maps in the standard MNI space were calculated. In Experiment 1, the SCN activity measured as the CBF was significantly modulated among the four scans (one-way repeated-measures ANOVA, $F(3,78) = 3.38$, $P = 0.022$). The activity at 12:00 was significantly higher than that at 6:00 (Tukey-Kramer test, $P < 0.05$). In Experiment 2, the SCN activity was measured in detail every 30 min from 24:00 to 6:00. A mixed-effects linear regression analysis indicates that the SCN activity gradually decreased from midnight to dawn (beta estimate = -1.40, $t(215) = -2.41$, $P = 0.017$). The SCN activity during the night better matched with the activity of the rodent SCN, synchronously with lights off. These results suggest that the diurnal variation of the SCN activity in humans was consistent with that in non-human mammals and could be influenced by ambient lights.

### [1S10a-4]

### Sex-dependent effect of chronic shifts of light-dark cycles on circadian clock and metabolism

*Shinobu Yasuo[1] ([1]*Faculty of Agriculture, Kyushu University*)

Chronic shifts of light-dark cycles or chronic jet lag (CJL) disrupts the circadian clock and increases the risks of metabolic disorders. Sex has been identified as an important factor influencing the circadian system, and sex differences have been reported on the response to circadian misalignment or association between rotating shift-works and metabolic diseases. However, results are inconsistent due to complex confounding factors. In animal experiments, previous studies mainly examined the effects of irregular light-dark cycles on health indicators in male animals, and sex differences of the effects are only partially understood. This study compared the effects of CJL on the circadian clock and metabolism using male and female C57BL/6N mice. For the CJL treatment, the lighting schedule was advanced by 6 hours every other day over 8 weeks. Following CJL, males exhibited significantly higher weight gain and glucose intolerance, while females experienced a slight decrease of weight gain and no significant changes of glucose tolerance. Food intake was not significantly altered by CJL in both sexes; the observed changes of body weight may be attributable to metabolic alteration. Animals of both sexes maintained activity and temperature rhythms after CJL. However, CJL females demonstrated a significantly weakened amplitude of core body temperature rhythms, compared to control females. CJL altered clock gene expression rhythms in the liver and adrenal gland in both sexes, with females experiencing a greater loss of rhythmicity. Notably, CJL elicited distinct effects on metabolic genes in the liver, such as increases of a lipase expression only in females. To determine if the distinct male and female responses to CJL are related to testosterone, we performed gonadectomy and testosterone replacement in males. Gonadectomized males under CJL exhibited female patterns of body weight gain, glucose tolerance, core body temperature rhythms, and expression of a lipase in the liver. Continuous testosterone treatment in gonadectomized males rescued male patterns of them. These findings suggest that CJL has sex-specific impacts on the circadian clock and metabolism, and testosterone is a major contributing factor of the sex differences.

### [1S10a-5]

### Temperature entrainment mechanism of peripheral clocks via circadian body temperature and its role in physiology

*Takahito Miyake[1], Masao Doi[1] ([1]*Kyoto University, Graduate School of Pharmaceutical Sciences*)

Body temperature in homeothermic animals does not remain constant but displays a regular circadian fluctuation within a physiological range (e.g., 35°C to 38.5°C in mice), constituting a fundamental systemic signal to harmonize circadian clock-regulated physiology. Recently, our lab identified a minimal upstream open reading frame (uORF) in the 5'UTR of the core clock gene *Per2* and assigned its role as a regulatory RNA module for temperature-dependent clock entrainment. A temperature shift within the physiological range does not affect transcription but instead increases translation of Per2 through its minimal uORF. Genetic ablation of the *Per2* minimal-uORF and inhibition of phosphoinositide-3-kinase (PI3K), lying upstream of temperature-dependent Per2 protein synthesis, compromised the entrainment of cells to simulated body-temperature cycles. At the organismal level, *Per2*-minimal-uORF-deficient skin showed delayed wound regeneration, indicating that uORF-mediated modulation of Per2 is crucial for optimal tissue homeostasis. Combined with transcriptional regulation, the uORF-mediated translational regulation of Per2 therefore helps enhance the fitness of circadian physiology. Further regulatory mechanisms underlying Per2 translation will be additionally discussed.

### Symposium

[1S02e]
Science and Research Committee / Committee for Research Ethics
**Educational Lecture 2**

March 28, 17:30 - 18:30, Room 2

**[1S02e-1]**

**Peer review of academic papers and research ethics: Teaching you how to peer review**

*Kiyoshi Kitamura[1] ([1]*Japan Association for Development of Community Medicine*)

### Symposium

[1S03e]

Cooperation with Other Societies Committee

### The physiological significance of ion-regulated membrane protein localization revealed by morphological and physiological approaches

March 28, 16:30 - 18:30, Room 3

### [1S03e-1]

### Cellular and subcellular distribution of Na/K-ATPase Alpha1 and 3 subunits on the plasma membrane of mouse hippocampal neurons

*Tatsuya Ishikawa[1,2], Kazuki Kuroda[2], Koshi Murata[2], Noriyuki Ozaki[1], Yugo Fukazawa[2]
([1]*Kanazawa Univ.*, [2]*Fukui Univ.*)

The asymmetrical distribution of $Na^+$ and $K^+$ in the cell is formed and maintained by an ion pump, sodium/potassium-ATPase (Na/K-ATPase: NKA), which is composed of two essential subunits, alpha and beta subunits. Four isoforms were identified for the alpha subunit (alpha1-4); alpha1 and 3 as neuronal isoforms in the brain with distinct affinities for ATP. Thus, expression of these isoforms across neuronal subpopulations, the subcellular distribution of the isoforms over distinct functional domains in the plasma membrane and their alteration, if any, in response to neuronal activity are essential to understanding the molecular basis of membrane excitability of these cells. On the other hand, previous studies indicated that the amylospheroids (Alzheimer's disease patient-derived 10-15 nm spherical amyloid β-protein oligomers) and α-synuclein specifically impaired the activity of alpha3 containing NKA and led to neuronal death. Accordingly, clarifying the distribution of alpha3 in each functional domain of neurons is also important to develop prophylactic or therapeutic methods for neurodegenerative diseases. However, cellular and subcellular expression patterns of the neuronal subunits in the brain remain largely elusive. We investigated mRNA and protein expression of these isoforms in the mouse brain at light and electron microscopic levels. This presentation presents the first quantitative picture of expression patterns of NKA subunits at cellular and subcellular levels. First, we show localization and co-localization of mRNAs for alpha1 and 3 isoforms in the hippocampus by single- and double-fluorescent *in situ* hybridization analysis. Next, we show the distribution of immunoreactivity for these subunits at a light microscopic level by specific antibodies for individual subunits, which is consistent with cellular expression patterns revealed by the *in situ* hybridization analysis. These analyses identify neuronal subpopulations in these regions expressing either of these isoforms together with those expressing both isoforms. Lastly, we show the subcellular distribution of these isoforms over the plasma membrane of various hippocampal and dentate gyrus neurons at an electron microscopic level by a quantitative and highly sensitive molecular localization technique: SDS-digested freeze-fracture replica labeling (SDS-FRL). The distribution of immunogolds for NKA alpha 3 was found in each functional subdomain such as hippocampal pyramidal cells or dentate gyrus granule cells by SDS-FRL. This analysis reveals cell-type dependent differential distribution of NKA alpha3 across distinct functional subdomains. These data provide fundamental information for cell type-dependent regulatory mechanisms for the membrane excitability of neurons.

### [1S03e-2]

### Mechanisms of regulating Nav channel distribution at the axon initial segment

*Ryota Adachi[1] ([1]*Department of Cell Physiology, Graduate School of Medicine, Nagoya University*)

The axon initial segment (AIS) is a highly excitable domain located near the soma and is involved in generating action potentials. At the AIS, voltage-gated $Na^+$ (Nav) channels are densely accumulated and tethered to the cytoskeletal proteins, actin-spectrin meshwork and microtubules, which is mediated by their interaction with a scaffold protein, ankyrinG. This accumulation of Nav channels lowers the threshold for action potential generation at the AIS. Length and position of the AIS varies among cell types and brain regions, and changes in a manner dependent on neuronal activity. This structural plasticity of the AIS contributes to the homeostatic control of neuronal activity and optimizes the function of neural circuits.Nucleus magnocellularis (NM) is an avian homolog of mammalian anteroventral cochlear nucleus and well known for such differentiations of the AIS. NM cells receive auditory input, while the AIS of these cells decreases its length as the input increases during development. To address the molecular basis of this AIS shortening, we prepared a slice culture containing NM that reproduces most features of AIS plasticity *in vivo*, and performed pharmacological analyses. Treating the culture with a high-$K^+$ medium shortened the AIS and reduced sodium current and membrane excitability. Pharmacological analyses revealed that this AIS shortening was driven by multiple $Ca^{2+}$ pathways and subsequent signaling molecules that converge on CDK5 via the activation of ERK1/2. AIS shortening was suppressed by overexpression of dominant-negative CDK5, whereas it was facilitated by the overexpression of p35, an activator of CDK5. Notably, p35(T138A), a phosphorylation-inactive mutant of p35, did not shorten the AIS. Moreover, microtubule stabilizers occluded AIS shortening during the p35 overexpression, indicating that CDK5/p35 mediated AIS shortening by promoting disassembly of microtubules at distal AIS. Disassembly of microtubules may alter the distribution of Nav channels by destabilizing the localization of ankyrinG. This study highlights the importance of microtubule reorganization and regulation of CDK5 activity in structural AIS plasticity and the tuning of AIS characteristics in neurons.

### [1S03e-3]

### Physiological impact of P/Q-type voltage-gated calcium channel distribution in the presynaptic active zone on synaptic transmission

*Kohgaku Eguchi[1,2], Ryuichi Shigemoto[2] ([1]*Okinawa Institute of Science and Technology*, [2]*Institute of Science and Technology Austria*)

When an action potential arrives the presynaptic terminal, a voltage-gated calcium channel (VGCC) opens and calcium ions enter in, which triggers neurotransmitter release. Synaptic vesicles (SVs) are tethered near VGCCs by protein complexes, including RIMs, Munc13-1, Rab3, etc, which maintain a tight coupling distance between the source of calcium ion influx and the calcium sensor on the SV, resulting in efficient synchronous neurotransmitter release. At many synapses in the mammalian central nervous system, P/Q-type voltage-gated calcium channels ($Ca_V2.1$) are the major VGCC α subunit responsible for neurotransmitter release. It is controversial how $Ca_V2.1$ is distributed in the presynaptic AZ and its spatial relation to docked SVs.
In this talk, I will introduce an immunoelectron microscopic technique called SDS-digested freeze-fracture replica labeling (SDS-FRL) to visualize the distribution pattern of $Ca_V2.1$ in the presynaptic terminal at the nanoscale and demonstrate the distribution pattern of $Ca_V2.1$ at parallel fiber-Purkinje cell (PF-PC) synapses in mouse cerebellar cortex. At PF-PC synapses, the coupling between $Ca^{2+}$ influx and the sensor on SVs is tightened during synaptic maturation, and in parallel, the alternative splicing isoform of $Ca_V2.1$ EF-hand switches from EFb to EFa. It is indicated that the developmental shift of the $Ca_V2.1$ EF-hand splicing isoforms may play an important role in tightening $Ca^{2+}$ influx/sensor coupling for neurotransmitter release at PF synapses. I will report the results of the study that examined differences in coupling distances between knock-in mice expressing only $Ca_V2.1$[EFb] and wild-type (WT) mice that predominantly express the EFa mutant at PF-PC synapses using electrophysiology and SDS-FRL and discuss the impact of the difference in the tightness of $Ca^{2+}$ influx-sensor coupling caused by the $Ca_V2.1$ EF-hand splicing variants to the synaptic transmission at PF-PC synapses in the mouse cerebellum.

### [1S03e-4]

### Physiological impacts of spatial distribution of mitochondrial $Ca^{2+}$ transporters in cardiomyocytes

*Ayako Takeuchi[1], Satoshi Matsuoka[1] ([1]*Department of Integrative and Systems Physiology, Faculty of Medical Sciences, University of Fukui and Life Science Innovation Center, University of Fukui*)

It is well accepted that communications between mitochondria and sarcoplasmic/endoplasmic reticulum (SR/ER) via $Ca^{2+}$ dynamics play important roles in regulating various cellular functions. In cardiomyocytes, mitochondrial $Ca^{2+}$ dynamics is strictly regulated by the influx via the mitochondrial $Ca^{2+}$ uniporter MCU and the efflux via the mitochondrial $Na^{+}$-$Ca^{2+}$ exchanger NCLX. We previously reported that NCLX is localized in close proximity of SR $Ca^{2+}$ pump SERCA in mouse ventricular myocytes (Takeuchi and Matsuoka, *Int J Mol Sci*, 2022). Considering that MCU is localized near SR ryanodine receptor RyR, and far from NCLX (De La Fuente et al., *J Biol Chem*, 2016; *Cell Rep*, 2018), it is strongly suggested that the $Ca^{2+}$ cycling between mitochondria and SR via MCU-RyR and NCLX-SERCA has important roles in cardiomyocyte functions.
In the present study, we performed a “physiome study”, a combination of experiments and mathematical model analyses to get insight into the physiological impacts of spatial distribution of mitochondrial $Ca^{2+}$ transporters in cardiomyocytes.
In HL-1 cardiomyocytes, a cell line derived from mouse atrial myocytes, $Ca^{2+}$ reuptake into SR after the caffeine application was significantly slowed down in the presence of an NCLX blocker, CGP-37157. Diminishing NCLX also slowed the firing rate of the cells. In order to reproduce these experimental results in the mathematical model of HL-1, the assumption of strong coupling between MCU-RyR and NCLX-SERCA, and the spatial separation of these couplings were required.
To further get insight into the physiological impacts, we newly developed a human ventricular myocyte model with excitation-contraction-energy metabolism, by incorporating the updated $Ca^{2+}$ couplings between mitochondria and SR, detailed mitochondrial metabolism and excitation-contraction coupling. We will discuss the roles of the couplings in cardiac energetics.

### Symposium

### [1S04e]

### Diurnal adaptation gear to shift from the resting phase to the active phase

March 28, 16:30 - 18:30, Room 4

### [1S04e-1]

### Clock Protein Signaling for Synchronized Oscillation of the Central Cellular Clock; Toward Jet Lag Drug Discovery

*Teruya Tamaru[1], Genki Kawamura[2], Mamoru Nagano[3], Hikari Yoshitane[4,5], Kimiko Shimizu[6], Yoshitaka Fukada[5], Takeaki Ozawa[2], Yasufumi Shigeyoshi[3], Ken Takamatsu[1]
(*[1]Department of Physiology, Toho University, School of Medicine, [2]Department of Chemistry, School of Science, The University of Tokyo, [3]Department of Anatomy, Kindai University, School of Medicine, [4]Circadian Clock Project, Tokyo Metropolitan Institute of Medical Science, [5]Department of Biological Sciences, School of Science, The University of Tokyo, [6]Department of Pathological Cell Biology, Medical Research Institute, Tokyo Medical and Dental University*)

It is becoming increasingly clear that the global network society and other factors have disconnected the internal clock of modern humans from the environment, weakening the adaptive vitality of the human body and causing physical and mental disorders and illnesses due to sleep disorders and weakened immune systems. As evidence, we previously showed that the synchronized response of the biological clock drives numerous adaptive systems, and that when the synchronized response is impaired, adaptive capacity is reduced. The biological (cellular) clock, which functions in tissues and cells throughout the body, is the temporal basis for physiological functions and environmental adaptation through an autonomous oscillation mechanism and environmental synchronization ability by clock protein BMAL1 and others. In addition, oscillation of peripheral clocks throughout the body in a regulated phase under the direction of the central clock (SCN) in the brain, which responds synchronously to light, is essential for the coordinated expression of various physiological functions. This study will (1) elucidate the early signals of clock synchronization via BMAL1 that drive synchronous oscillation of cellular clocks underlying environmental adaptation, and (2) develop novel clock inhibitors targeting BMAL1 to lay the foundation for overcoming jet lag health disorders and developing transient clock disruption models. Using genome editing and live imaging of peripheral and central clocks (SCN), we elucidated that the S region of BMAL1 (BMAL1-S) is an essential protein code for the synchronized oscillation of central and peripheral cellular clocks. In addition, we discovered a novel clock inhibitor targeting BMAL1 and showed that this inhibitor reversibly resynchronizes the arrested central clock to the environment and that administration of this inhibitor to mice alleviates jet lag.

### [1S04e-2]

### Circadian protection against bacterial over-proliferation in the skin by the CXCL14/bacterial DNA/TLR9 pathway.

*Kosuke Tanegashima[1], Kojiro Tsujihana[2,3], Kenji Kabashima[3], Hitoshi Okamura[2,4], Takahiko Hara[1,5,6] (*[1]Stem cell project, Tokyo Metropolitan Institute of Medical Science, [2]Graduate School of Pharmaceutical Sciences, Kyoto University, [3]Department of Dermatology, Graduate School of Medicine, Kyoto University, [4]Department of Neuroscience, Graduate School of Medicine, Kyoto University, [5]Graduate School of Medical and Dental Sciences, Tokyo Medical and Dental University, [6]Graduate School of Science, Department of Biological Science, Tokyo Metropolitan University*)

Circadian rhythms control many physiological processes, including immune responses. Several studies have shown that the numbers of immune cells in blood and lymphoid organ change during the day, suggesting that systemic immune responses are regulated by circadian rhythms. However, it is still unclear how local immune responses are regulated in a circadian manner. We have been focusing on the chemokine CXCL14, which is produced by epidermal keratinocytes, and we found that the expression of CXCL14 exhibits robust circadian fluctuations. The expression of *Cxcl14* mRNA was high during subjective daytime and low at night in mouse epidermis. In contrast, in marmosets, a diurnal primate, circadian *Cxcl14* expression was reversed. CHIP analysis revealed that RORα, one of the key activators of the diurnal rhythm during the resting phase, binds to the upstream binding site of the *CXCL14* gene and upregulated its expression in a human keratinocyte cell line. The clearance of the skin pathogen *Staphylococcus aureus* (*S. aureus*) in nocturnal mice coincided with *Cxcl14* expression: high during subjective daytime and low at night. *Cxcl14*-knockout mice lost the enhanced clearance of *S. aureus* during the resting phase, suggesting that CXCL14 is involved in circadian-regulated immune responses in the skin. Our study has revealed that CXCL14 binds to DNA and delivers it into endosomes and lysosomes, where the bacterial DNA sensor Toll-like receptor (TLR) 9 is present. We found that CXCL14 also bound to *S. aureus* DNA and induced the production of inflammatory cytokines through TLR9-signaling pathways in dendritic cells and macrophages. We also showed that *Tlr9*-knockout mice lost the enhanced clearance of *S. aureus* during the resting phase. These data indicate that the circadian production of the epidermal chemokine CXCL14 rhythmically suppresses skin bacterial proliferation in mammals by activating the innate immune system. Fighting off a pathogen and activating the immune system requires a lot of energy. We propose that the circadian regulation of the immune response against *S. aureus* using the CXCL14/bacterial DNA/TLR9 pathway is one of the effective immune responses during the resting phase when energy-demanding activity is suppressed.

### [1S04e-3]

### The transcription factor AP-2β regulates the amount of non-REM sleep.

*Ayaka Nakai[1], Mitsuaki Kashiwagi[1,2], Tomoyuki Fujiyama[1], Kanako Iwasaki[1], Arisa Hirano[1,3], Hiromasa Funato[1], Masashi Yanagisawa[1], Takeshi Sakurai[1,3], Yu Hayashi[1,2]
(*[1]International Institute for Integrative Sleep Medicine (WPI-IIIS), University of Tsukuba, [2]Department of Biological Sciences, Graduate School of Science, University of Tokyo, [3]Institute of Medicine, University of Tsukuba*)

The molecular mechanism of sleep remains elusive. Human short sleepers may offer insight into the molecular mechanisms of sleep. We focused on the transcription factor AP-2β (*TFAP2B*). Sleep abnormalities such as short sleep and sleep-walking have been self-reported in human families that carry mutations in *TFAP2B*. Furthermore, in invertebrate animal species, orthologous genes of *TFAP2B* play crucial roles in the regulation of sleep. Previously, we provided direct evidence that TFAP2B is involved in mammalian sleep. We showed that different mutations in TFAP2B have diverse effects on the mouse sleep architecture including the reduction or fragmentation of non-REM sleep (Nakai *et al.*, *Genetics* 2020). Thus, AP-2 transcription factors are crucial for sleep regulation across the animal phyla. However, it is unclear when and where TFAP2B functions. Therefore, we generated mice in which *Tfap2b* is deleted specifically in the nervous system, and mice in which *Tfap2b* can be postnatally deleted specifically in neurons. Both mice exhibited reduced non-REM sleep amount, but the nervous system-specific deletion of *Tfap2b* resulted in more severe sleep phenotypes that were accompanied by abnormal light entrainment of circadian clock and stereotypic jumping behavior. These results indicate that TFAP2B functions at least partly in postnatal neurons in sleep regulation and imply that TFAP2B also functions either at earlier stages or additional cell types within the nervous system.

### [1S04e-4]

### Analysis of circadian clock mechanism in pain hypersensitivity and identification of novel analgesic target molecules

*Sai Yasukochi[1], Satoru Koyanagi[2,3], Kimitaka Suetsugu[1], Takeshi Hirota[1], Shigehiro Ohdo[2], Ichiro Ieiri[1] ([1]*Department of Pharmacy, Kyushu University Hospital,* [2]*Faculty of pharmaceutical sciences, Kyushu university,* [3]*Department of Glocal Healthcare Science, Faculty of Pharmaceutical Sciences, Kyushu University*)

In mammals, diurnal rhythms in physiological functions are governed by an internal self-sustained molecular oscillator referred to as the circadian clock. The circadian timekeeping system enables organisms to adapt their physiological and behavioral functions to anticipatory changes in their environment. Several studies have investigated the relationship between pain and patients' circadian timing, but the underlying mechanism of diurnal exacerbation of pain hypersensitivity remains unknown.
Pain hypersensitivity is often caused by peripheral nerve injury, which is associated with the hyperexcitability of neurons in the dorsal horn of the spinal cord. Peripheral nociceptive inputs activate the primary afferents via release of various chemical mediators which mediate signals from neuron to glia in the dorsal root ganglion and subsequently in the dorsal horn of spinal cord. The subsequent interactions between neuron and glia contribute the development and maintenance of pain hypersensitivity.
We previously demonstrated that spinal expression of serum- and glucocorticoid-inducible kinase-1 (SGK-1) is associated with glucocorticoid- induced exacerbation of neuropathic pain hypersensitivity, but there are no available strategies to inhibit SGK-1 in the spinal cord. By screening a clinically approved drug library (more than 1,200 drugs), we found that sulfasalazine (SSZ) has inhibitory effects on SGK-1. Therefore, I first describe the analgesic effects of SSZ, which was found to target SGK-1, an exacerbating factor for neuropathic pain.
Functional changes in spinal glial cells are also involved in the maintenance of cancer-induced pain hypersensitivity. Although accumulating studies demonstrate the circadian physiology of glial cells, their contributions to diurnal alterations in cancer-associated pain have yet to be clarified. During the analysis for spinal gene expression of tumor-bearing mice, we found that microglial expression of Cancer-induced pain factor (CIPF; Tentative) in the spinal cord exhibited a diurnal oscillation, which was governed by the molecular components of circadian clock. Temporal elevation in CIPF levels decreased the threshold of pain hypersensitivity in tumor-bearing mice. Therefore, I next describe an analysis of the diurnal alterations of cancer-induced pain focusing on CIPF.

### [1S04e-5]

### The Role of Glucocorticoids in Regulating Circadian Rhythm Expression

*Masaaki Ikeda[1], Shinnosuke Yanagisawa[1,2], Megumi Kumagai[1], Yoshihiro Nakajima[3], Yasuhiro Takenaka[4] ([1]*Department of Physiology, Faculty ofMedicine, Saitama Medical University,* [2]*Department of Diabetes andEndocrinology, Saitama Medical University,* [3]*Health ResearchInstitute, National Institute of Advanced Industrial Science and Technology,* [4]*Department of Physiology,Graduate School of Medicine, Nippon Medical School*)

Circadian rhythms play a vital role in the regulation of gene expression in peripheral organs, with their phases synchronized by signals originating from the suprachiasmatic nucleus—the epicenter of circadian rhythms. The synchronization of peripheral circadian rhythms relies on signals transmitted through autonomic nerves and hormonal stimulation. Notably, glucocorticoids emerge as a significant hormonal signal influencing peripheral circadian rhythms. Glucocorticoids are secreted by the adrenal glands in response to adrenocorticotropic hormone (ACTH) secreted by the pituitary gland. Importantly, ACTH secretion exhibits circadian rhythmicity, peaking in the morning in humans. Some clock genes have been identified as being induced by glucocorticoids, and their induction mechanisms have been elucidated through promoter analysis. Experimental studies conducted in cell culture systems have shown that the circadian rhythms of cells can be synchronized through the addition of dexamethasone—a synthetic adrenal corticosteroid—to the culture medium. In this presentation, we aim to provide a comprehensive overview of the pivotal role that glucocorticoids play in the mechanism of circadian rhythm expression.

### Symposium

[1S05e]
### Diverse into CardioCytoZoom

March 28, 16:30 - 18:30, Room 5

### [1S05e-1]
### A highly talented but different fragment of a larger communication protein

Mario Maalouf[1], Vu Nguyen[1], Jennifer Hunter[1], Robin Shaw[1], *Daisuke Shimura[1,2]
([1]*The University of Utah,* [2]*Tokyo Medical and Dental University*)

For cell-cell communication, gap junctions are essential hexameric channels at the cellular membrane. Connexin43 (Cx43) protein is the most abundant gap junction protein in the cardiac ventricle and it is encoded by the *gja1* gene. Failure of gap junction formation, such as due to diminished transport of Cx43 to the cell membrane, causes disharmony of cardiac rhythm and dangerous cardiac arrhythmias. In genetic origin arrhythmogenic cardiomyopathy (AC), Cx43 also does not traffic to the cell membrane, and arrhythmias result. However, at present, we do not have a therapy that restores gap junction formation in AC. Our recent discovery highlights the internally translated N-terminus truncated small isoform of Cx43, GJA1-20k, as a crucial trafficking subunit for Cx43 to the cell membrane. Here, we show the functional details of GJA1-20k and the application capability of GJA1-20k for AC by promoting Cx43 transport and restoring normal heart rhythm. We generated a GJA1-20k mutant mouse model which has a point mutation of the start codon of GJA1-20k to suppress its expression, while still expressing the full-length of Cx43. The homozygous mutant heart showed significant abnormality of electrical physiological function largely due to lack of gap junction formation, resulting in arrhythmogenic sudden death in 2 - 4 weeks after birth. Our findings also revealed that most of the Cx43 in homozygous mutant hearts are stuck in the cytosol and rapidly degraded, indicating that GJA1-20k is necessary to maintain Cx43 stability and trafficking in vivo. We then investigated whether the exogenously overexpressed GJA1-20k ameliorates the pathogenesis of AC in several mouse models of AC. In the models, exogenous GJA1-20k effectively mitigates the reduction of Cx43 expression despite mutations in AC-related (non-Cx43) genes. Our data demonstrate that a small isoform of Cx43, GJA1-20k, essentially regulates full-length Cx43 trafficking and manages the connection between adjacent cells. We anticipate that GJA1-20k will be central to a new paradigm of treatment for AC and cardiac arrhythmias in general.

### [1S05e-2]
### Mechanistic analyses of gas exchange tissue formation through *in vivo* imaging of zebrafish

*Hiroyuki Nakajima[1], Naoki Mochizuki[1] ([1]*National Cerebral and Cardiovascular Center Research Institute*)

Beginning with fish, vertebrates have developed organized circulatory system throughout the body to distribute oxygen to every cell. Oxygen, the final acceptor of electrons in oxidative phosphorylation reactions, is essential to sustain life. In vertebrates, uptake of oxygen from the outside occurs through respiratory organs such as lungs and gills, where numerous alveoli and gill filaments maximize surface area, thereby allowing efficient uptake of oxygen from the air and water. Commonly in these gas exchange tissues, vascular endothelial cells (ECs) and flattened epithelial cells create a gas exchange niche by contacting each other, where morphological and functional specialization of ECs and epithelial cells allow for gas exchange by diffusion. However, it is unclear how ECs and epithelial cells cooperatively interact to form a functional gas exchange niche. In this study, we focus on the process of gas exchange tissue formation in zebrafish. We have established a unique system that enables simultaneous *in vivo* imaging of ECs, epithelial cells, and blood flow during gill development. We first found that gill vasculatures are formed by a novel type of angiogenesis, which is driven by the deformation of an EC sheet in response to blood flow. Furthermore, simultaneous imaging of ECs and polarized epithelial cells revealed that angiogenic ECs actively control the shape of the outer surface epithelial sheets through direct interaction to form a suitable gas exchange niche. In general tissue formation, the area of blood vessels is defined by the shape and size of the tissue. In contrast, we will present a novel mode of tissue morphogenesis, in which blood vessels actively control the formation of a functional gas exchange tissue.

### [1S05e-3]
### Multidimensional nano-scale analyses for *in vivo* vascular cells

*Naoki Honkura[1] ([1]*Hamamatsu University School of Medicine Dept. Med.Physiology*)

Organisms such as humans maintain all the cells in their body, supported by the accurate transportation of the absorbed nutrients via a blood vessel. The network of blood vessels must be responsible for substance transportation as the only way to provide tons of molecules to living cells in animal. We visualize blood vessels and tissues in the mouse skin surface and how nutrients are transported from the blood vessels to cells in the organ. Moreover, we have tried to visualize variable intravital metabolic activity in single vascular cells with intravital rapid FLIM imaging. Here, we would like to introduce and discuss our system to measure these activities and a part of the result in this meeting.

### [1S05e-4]

### Spatial transcriptomics of the heart, by the beginner, for the beginner.

*Masaki Miyasaka[1], Masahide Seki[2], Akinori Kanai[2], Yutaka Suzuki[2] ([1]*The Jikei University School of Medicine,* [2]*Graduate School of Frontier Sciences*)

Technological advancements have enabled single-cell analysis to capture gene expression information at the cellular level that couldn't be obtained through bulk-level analysis. However, an issue with the single-cell analysis methods was the loss of spatial information due to the isolation of cells. To address this, spatial transcriptomics technology was developed, allowing for the analysis of the relationship between gene expression patterns and their locations within tissues. However, when compared to single-cell analysis, there are still challenges in resolution and transcriptome coverage. In this presentation, we introduce spatial transcriptomics technology applied to mouse hearts.

### [1S05e-5]

### The mechanism of mitochondrial dynamics regulation via PIPs

*Sho Aki[1], Tsuyoshi Osawa[1] ([1]*Division of Integrative Nutriomics and Oncology RCAST, The University of Tokyo*)

The balance between mitochondrial fusion and fission (mitochondrial dynamics) is the cornerstone of maintaining the morphology and function of mitochondria, and in cancer, it is known that involved in motility and metastasis. While mitochondrial morphology changes dynamically moment by moment, the detailed mechanism of mitochondrial dynamics remains largely unknown. Phosphoinositides (PIPs) consist of the plasma membrane and organelle membranes, and changes in PIPs are important for intracellular membrane trafficking including organelles. PIPs that regulate the mitochondrial fusion process were explored to clarify the molecular mechanism of mitochondrial fusion. It was found that $PI(3,4)P_2$ promotes mitochondrial fusion by recruiting membrane fusion-related factors. Knockdown of $PI(3,4)P_2$ metabolic enzymes did not affect the expression levels of fusion factors (Mfn1/2) or fission factors, and although Mfn1 was properly recruited to the mitochondrial fusion site, significant mitochondrial fragmentation was observed. Furthermore, cardiac-specific $PI(3,4)P_2$ metabolic enzymes-knockout mice showed cardiac dysfunction owing to mitochondrial abnormalities. These observations indicate that $PI(3,4)P_2$ is a novel metabolite that promotes mitochondrial fusion and controls mitochondrial dynamics. Our findings may lead to the development of therapeutics for mitochondrial-dysregulated diseases, such as cardiomyopathy and neurodegenerative diseases.

### Symposium

[1S06e]
Cooperation with Other Societies Committee
**State-of-the-art muscle physiology research: from single molecules and cells to living organisms**

March 28, 16:30 - 18:30, Room 6

### [1S06e-1]

### Sarcomere synchronization regulates ventricular contractility in the *in vivo* beating mouse heart

*Fuyu Shimozawa Kobirumaki[1], Tomoko Oyama[2], Kotaro Oyama[2], Mitsumasa Taguchi[2], Norio Fukuda[1] ([1]*Jikei Univ. Sch. of Med.*, [2]*Nat. Ins. QST*)

In the present study, we expressed α-actinin-AcGFP in Z-disks to analyze sarcomeric movements along a myofibril in a left ventricular (LV) myocyte in anesthetized open-chest mice. For quantification of the magnitude of contribution of individual sarcomeres to myofibrillar dynamics, we introduced "contribution index" (CI) to quantify the synchrony in movements between a sarcomere and a myofibril (from -1 [complete asynchrony] to 1 [complete synchrony]). We found that: 1) CI varied markedly (i.e., -0.4 to 0.7) between sarcomeres along a myofibril, with an average of ~0.3 under normal contractile conditions, 2) when the movements between adjacent sarcomeres were asynchronous (CI < 0), a sarcomere and the ones next to the adjacent sarcomeres and farther away, moved in synchrony (CI > 0), and 3) under depressed contractile conditions (LV pressure < 10 mmHg), diastolic sarcomere length (SL) increased up to the upper limit (~2.2 µm), and the movements between adjacent sarcomeres became marked asynchrony (CI, -0.3 to -0.4). Next, when preload was reduced by clamping of the inferior vena cava, SL was shortened on average by ~0.2 µm, and the individual SL variance was increased by ~50%, both of which returned to the pre-clamping levels within 3 sec, demonstrating that an imbalance of active and passive force between sarcomeres is smoothed in response to a change in mechanical load along myofibrils. Furthermore, by culturing rat neonatal cardiomyocytes on micro-patterned gelatin hydrogels, we found that: 1) the orientation of Z-disks became improved along myofibrils, and 2) sarcomere shortening became greater in association with improved synchrony. Therefore, the following are suggested: 1) sarcomere movements are heterogeneous due to an imbalance of active and passive force between neighboring sarcomeres even under physiologic conditions, 2) the orientation of Z-disks is an important factor that regulates sarcomere dynamics, and 3) sarcomere synchrony generated by the distal inter-sarcomere interaction via titin's passive force along myofibrils regulates cardiac contractile function *in vivo*.

### [1S06e-2]

### Evaluation of skeketal myosin intermolecular cooperativity using information theory

*Motoshi Kaya[1] ([1]*University of Tokyo, Department of Physics*)

We have evaluated the mechanical properties of single-multiple molecules of skeletal and cardiac myosin, which have very similar amino acid compositions. From these results, we found that skeletal and cardiac myosin each have distinctive molecular properties, and that these properties exhibit characteristic functions in force generation in myosin ensembles. For example, skeletal myosins enhance the probability of synchronizing the timing of force generation between molecules at high loads, ensuring faster contractions than those achieved by random force generation. Such cooperative function is an essential property involved in the high contractile force and velocity output required for skeletal muscle. On the other hand, cardiac myosin can bind to actin for long periods of time at high loads, with less frequency of detachment from actin, by its repeated conformational changes in the direction of contraction and vice versa. As a result, a higher fraction of cardiac myosins can bind to actin than that of skeletal muscle myosin, thus maintaining nearly twice as much force for a longer period than skeletal myosin ensembles. This cooperative function is thought to be responsible for the cardiac contractions that produce a steady blood stroke volume in the heart. However, no physical quantity has been proposed as an indicator of the degree of such cooperative phenomena. In this study, we focus on information to quantify the changes in cooperative phenomena in myosin ensembles. In other words, we calculate the mutual information of myosin ensembles, assuming that the information transmission ability between molecules increases when cooperative phenomena occur between molecules. The mutual information can be calculated by finding the probability distribution of inputs and outputs. Hence in this study, the load acting on myosin was taken as input and the sliding velocity of actin as output, and these physical quantities were measured by optical tweezers to evaluate the mutual information and information efficiency, which indicates the ability of information transmission, from these input-output relationships. As a result, it was found that when the number of interacting molecules of skeletal myosin increased from 10 to 30, the information transmission efficiency increased from 20% to 30%. These results suggest that it may be possible to evaluate the degree of cooperative phenomena among myosin molecules by assessing the amount of information among myosin molecules. We believe that such an approach is also effective to evaluate cooperative phenomena among various proteins.

### [1S06e-3]

### On accuracy of macroscopic medical indicators obtained by multiscale heart simulator incorporating stochastic cooperativities of molecular motors

*Takumi Washio[1,4], Jinse Shimo[2], Isao Shiraishi[2,3], Toshiaki Hisada[1] ([1]*UT-Heart Inc.*, [2]*Japan Medical Device Corporation*, [3]*National Cerebral and Cardiovascular Center*, [4]*University of Tokyo*)

Changes in intracellular calcium concentrations regulate heartbeats. However, the rise in the left ventricular pressure during isovolumetric systole is gentler than that of the Ca2+ transient, and the decline in the left ventricular pressure during early diastole is much sharper than that of the Ca2+ transient. These mismatches between the input signal and the macroscopic outcomes are thought to be due to the cooperative properties of molecular motors. In the UT-Heart simulator, the heart pumping is driven by the stochastic molecular motor models arranged in the sarcomere models which are in turn imbedded into the heart wall. Therefore, the cooperative behaviors of molecular motors pulling the common thin filament are naturally incorporated into the numerical model. In this lecture, we introduce how the microscopic cooperativities affect the macroscopic medical indicators like blood pressure and cardiac outputs through the numerical simulation studies using UT-Heart. We also report how accurately UT-Heart reproduced the individual patient's hearts in ongoing project of the preoperative predictions of congenital heart diseases.

### [1S06e-4]

### Microscopic decoding of heat-activated and -releasing muscle contractions

*Madoka Suzuki[1] ([1]*Institute for Protein Research, Osaka University*)

Force and heat generations are major roles of muscles. Force generation is directly related to heat generation as a shivering thermogenesis. Understanding how heat affects contraction at the molecular scale could lead to new hyperthermia strategies to improve muscle performance. However, the fundamental mechanism of heat-contraction coupling remained largely unclarified. We have previously reported that heating induces contraction of both skeletal and cardiac muscle cells without intracellular $Ca^{2+}$ rises. This heat-induced contraction in cardiac cells was later reproduced at the molecular scale in the *in vitro* motility assay combined with the optical heating microscopy. In the *in vitro* motility assay, the sliding motion of fluorescently labeled actin filaments propelled by myosin molecules that are attached to a glass surface is examined under the fluorescence microscope. The optical heating microscopy creates a concentric temperature gradient around the focal point of a focused infrared laser beam that is well absorbed by water. Recently, we investigated the heat sensitivity of skeletal and cardiac contractile systems reconstituted from proper pairs of myosin and thin filament (actin filament and tropomyosin-troponin complex). We confirmed that heating induces sliding of both skeletal and cardiac systems under a muscle relaxation condition. These results indicate that the heat shifts the "*on–off*" equilibrium of muscle contractile systems to the "*on*" state within the body temperature range, even in the absence of $Ca^{2+}$. Further analysis by switching the combination of myosin and thin filament revealed that the lower temperature dependence of skeletal myosin compared to cardiac myosin is compensated by the higher temperature dependence of skeletal thin filament compared to cardiac thin filament. Overall, the temperature dependence of skeletal muscle is higher than that of the heart. The difference may represent the fine-tuned regulation of muscle contraction to match physiological demands. In this symposium, I will also introduce our attempts to use an *in situ* X-ray diffraction recording from insect flight muscles to reveal the molecular mechanisms of thermogenesis in a living beetle.

### [1S06e-5]

### Identifying the differentiation mechanism of tissue macrophages important for cardiac morphogenesis

*Norika Liu[1], Naofumi Kawahira[2], Yasuhiro Nakashima[3], Haruko Nakano[2], Akiyasu Iwase[4], Yasunobu Uchijima[4], Sean Wu[5], Susumu Minamisawa[1], Hiroki Kurihara[4], Atsushi Nakano[1,2] ([1]*The Jikei University School of Medicine,* [2]*University of California, Los Angeles,* [3]*Kyoto University,* [4]*University of Tokyo,* [5]*Stanford University*)

The cells that comprise the circulatory system not only share their origins but also mutually promote each other’s differentiation during the formation of functional circulatory system. We previously reported a subset of endocardial cells is hemogenic during early embryogenesis. They are enriched in the cushion region, the primordia of the cardiac valves and septa, where active remodeling via endothelial-mesenchymal transition takes place. Hemogenic endocardial cells undergo endocardial-hematopoietic transition (EHT) via Nkx2-5-dependent manner, suggesting that Drosophila tinman-dependent cardio-hematopoietic program is conserved in mammals. In this study, we analyzed the regulatory network of Nkx2-5-dependent endocardial hematopoiesis using a scRNA-seq data from wildtype and Nkx2-5-null embryonic hearts. As expected, Nkx2-5-null hearts were devoid of clusters for hemogenic endocardium and cushion endocardium. Interestingly, scRNA-seq analysis further revealed that genes related to Notch signaling pathway are significantly downregulated in Nkx2-5-null endocardium. A further gene network analysis identified that Dhrs3, an enzyme that attenuates retinoic acid (RA) signal by catalyzing the reduction of all-trans-retinaldehyde to all-trans-retinol, is a signature gene of the hemogenic endocardial cells downstream of Nkx2-5. Although RA signal is known to induce the formation of multipotent progenitor, our *ex vivo* hematopoietic colony forming assay revealed that macrophage formation is strongly inhibited by RA signal. Notch inhibition also suppressed the formation of macrophages. Consistently, *in vivo* forced activation of NICD drastically increased the number of $CD41^{+}$ hemogenic endocardial cells as well as macrophages in the cardiac cushion. Strikingly, impaired hematopoiesis and cushion defects in the Nkx2-5-null heart were both rescued by overexpression of NICD, suggesting that Notch signaling promotes endocardial hematopoiesis downstream of Nkx2-5.Taken together, our study demonstrated that the Nkx2-5/RA/Notch signaling axis plays a pivotal role in EHT during early embryogenesis, thereby facilitating local tissue remodeling by inducing macrophage differentiation.

### Symposium

[1S08e]
Cooperation with Other Societies Committee
**Novel Research Developments in Health Science Based on the evidence of Physical Inactivity Studies~The Latest Findings in Skeletal Muscle Research~**

March 28, 16:30 - 18:30, Room 8

### [1S08e-1]

### Development of physical inactivity model in animals, and muscle adaptations and its sex differences induced by physical inactivity in the growth process

*Toshinori Yoshihara[1] ([1]*Graduate School of Health and Sports Science*)

In the past decade, physical inactivity (sedentary lifestyle) has been recognized as a risk factor for morbidity and mortality due to cardiovascular diseases, cancer, chronic respiratory diseases, and diabetes all over the world. As skeletal muscle is the most common and widely distributed muscle tissue in the body, the loss of skeletal muscle mass and strength can result in a drastic reduction in an individual's quality of life and lead to an increased risk for the development of insulin resistance and various chronic health conditions. Thus, maintaining skeletal muscle mass is necessary to maintain our health. Indeed, aging per se is associated with a progressive loss of skeletal muscle mass and strength—so-called sarcopenia is associated with musculoskeletal frailty, impaired health span, and quality of life in older individuals; however, this reproducible syndrome also applies to the young and middle-aged, as well as the elderly people. Importantly, a sedentary lifestyle in childhood has a great impact on later life; thus, physical inactivity among children and adolescents is also a major problem in the maintenance of skeletal muscle and health.In the growth process, physical inactivity induces developmental disorder but, in most cases, we cannot notice the abnormality because the organ and skeletal muscle will grow with age even if the activity is not enough and it has a great impact on inter-individual variability in the growing process. Furthermore, the evidence has not been established due to the difficulty of research in children and adolescents; therefore, animal experiments have provided great insights into the investigation of the physical inactivity-induced phenomena and mechanisms in growing children. For example, our recent work reveals that 8 weeks of physical inactivity, induced by the cage volume reduction, exacerbates subsequent disuse-induced skeletal muscle atrophy without the alternations in metabolic and physiological properties in rat soleus muscle.Moreover, growing evidence indicates that there are sex-related differences in response to muscle inactivity and overload both in human and animal models. Although the precise mechanisms associated with sex-specific differences in muscle adaptation remain debatable, our data suggest that males and females show different responses to the FoxO3a/ubiquitin–proteasome pathway and canonical TGF-β signaling (Smad signaling) following disuse in rat soleus muscle.This symposium will introduce experimental animal models of physical inactivity and present recent research on the changes, adaptations, and sex differences in skeletal muscle induced by physical inactivity focusing on the growth process.

### [1S08e-2]

### The molecular mechanisms of skeletal muscle atrophy by physical inactivity

*Yuki Tomiga[1] ([1]*Faculty of Sports and health science, Fukuoka University*)

Physical inactivity is one of the most serious health problems worldwide. In fact, the global economic burden of healthcare was reported to be approximately $5.4 billion in 2013. Physical inactivity leads to skeletal muscle atrophy. Epidemiological studies have shown that skeletal muscle mass and strength are related to life expectancy. Given Japan's entry into an unprecedented era of super-aging, it is important to elucidate the molecular mechanisms that maintain skeletal muscle mass and function in order to create a healthy future and develop concrete solutions for the future.

The authors have focused on epigenetics, which is 'the study of how cells control gene activity without changing the DNA sequence,' of neuronal nitric oxide synthase (nNOS) as one of the regulatory mechanisms of skeletal muscle atrophy associated with muscle inactivity. nNOS is the major source of nitric oxide (NO) production in skeletal muscle and is known as one of the proteins constituting the skeletal muscle cell membrane. In skeletal muscle, nNOS plays an important role in both muscle hypertrophy and atrophy. When nNOS migrates into the cytoplasm, it produces NO gas and induces atrogin-1 and MuRF1, which are genes related to muscle atrophy, ultimately leading to skeletal muscle atrophy. Previously, the authors demonstrated that nNOS expression in atrophic skeletal muscle may be epigenetically regulated by DNA methylation, which is one of the major epigenetic modifications (Tomiga et al., 2019). Muscle inactivity using cast immobilization induced significant muscle atrophy in the soleus muscle. In the atrophied soleus muscle, total *Nos1* gene expression, including *Nos1α* and *Nos1β*, and nNOS protein levels were also greatly reduced. In addition, DNA methylation levels of the *Nos1* gene were markedly increased, showing a significant negative correlation with mRNA levels. These results suggest that nNOS expression during muscle inactivity may be epigenetically regulated by DNA methylation. In contrast, increased physical activity through exercise effectively combats skeletal muscle atrophy. Our unpublished findings suggest that adipose tissue may play a role in preventing skeletal muscle atrophy during exercise.

In this symposium, including our findings, we will discuss the molecular mechanisms of skeletal muscle atrophy caused by physical inactivity.

### [1S08e-3]

### Capillary regression of skeletal muscle associated with physical inactivity and the countermeasure.

*Hidemi Fujino[1] ([1]*Kobe University*)

Tissue microcirculation transports oxygen and nutrients, and disturbances in microcirculation affect the metabolic functions of cells. The network of capillaries involved in tissue microcirculation adapts and increases or decreases with cells and cross-talk, and skeletal muscle capillaries regress during physical inactivity. The capillaries form a ladder-like structure, and regression of anastomotic capillaries and TUNEL positivity of endothelial cells are especially observed during physical inactivity. In addition, temporal observation of changes in angiogenic and angiogenic inhibitory factors involved in capillary regulation shows that thrombospondin-1, an angiogenic inhibitory factor, increases rapidly in the early stage, and the expression balance between angiogenic and angiogenic inhibitory factors is disrupted. VEGF, an angiogenic factor, decreases after a while, and capillary regression progresses slowly during physical inactivity condition. Exercise can promote muscle hypertrophy, but it also induces excessive reactive oxygen species, and capillary regression progresses in the fragile state of skeletal muscle due to physical inactivity. Thus, capillary regression progresses in skeletal muscle overexpressing reactive oxygen species. Meanwhile, suppressing the excessive production of reactive oxygen species in skeletal muscle with antioxidant nutrients can optimize the expression balance of angiogenic factors and angiogenesis inhibitory factors and attenuate capillary regression. For example, astaxanthin, a carotenoid, propolis, a bee product, and chlorogenic acid, a coffee extract, are functional nutrients with antioxidant properties that attenuate capillary regression by suppressing excess reactive oxygen species. Thus, it is thought that microvessels and muscle cells cross-talk, and elucidation of the mechanism by which the oxygen environment in tissues regulates the increase or decrease of capillaries and cross-talk with skeletal muscle cells is expected to contribute to the improvement of muscle atrophy and sarcopenia.

### [1S08e-4]

### The Significance of Physical Inactivity in Non-obese Metabolic Diseases.

*Saori Kakehi[1], Yoshifumi Tamura[1], Takashi Funayama[1], Ryuzo Kawamori[1], Hirotaka Watada[1] ([1]*Juntendo University Graduate School*)

One of the most widely recognized health problems in the world today is metabolic syndrome. Much research has been conducted on the relationship between this condition and physical inactivity. The primary mechanism of skeletal muscle insulin resistance, a major cause of type 2 diabetes and metabolic syndrome, is obesity-mediated lipid accumulation in skeletal muscle cells, caused by factors such as obesity-induced fat cell dysfunction, inflammation, and elevated free fatty acids in the blood (the fat-centric hypothesis). However, this hypothesis applies primarily to obese individuals in the West and cannot fully explain the fact that non-obese people in Japanese and East Asian populations often develop type 2 diabetes. Furthermore, physical inactivity has been shown to be associated with mortality and the risk of type 2 diabetes independent of obesity, highlighting the need to understand this mechanism in order to prevent and treat metabolic diseases in non-obese individuals. In this context, our research has focused on the accumulation of intramyocellular lipid, known to be associated with a decrease in skeletal muscle insulin sensitivity caused by factors like a high-fat diet (HFD). In fact, a 3-day HFD was associated with increased IMCL levels and decreased insulin sensitivity in the tibialis anterior muscle in 50 non-obese Japanese men. However, large individual differences in fat-loading sensitivity to a HFD were observed, and fat-loading sensitivity was attributed to differences in the expression of lipid metabolism genes in skeletal muscle. On the other hand, these individual differences in fat-loading sensitivity were associated with the amount of daily physical activity, suggesting that the amount of physical activity may be related to intracellular lipid accumulation in skeletal muscle cells. At the animal experimental level, we also examined the combined effects of physical inactivity and HFD on skeletal muscle insulin sensitivity using a hindlimb cast immobilization (HCI) model of inactivity, and found that HCI increased intracellular diacylglycerol, which is associated with impaired insulin signaling and decreased insulin sensitivity, and that this phenomenon was further exacerbated when combined with HFD.

In conclusion, we emphasized the importance of physical inactivity as an independent risk factor for health problems such as type 2 diabetes and metabolic syndrome. We also showed that dietary changes affecting insulin sensitivity and intracellular lipids in skeletal muscle have a negative impact on them. Understanding these mechanisms may be crucial for the prevention and treatment of non-obese type 2 diabetes, especially in East Asians, including Japanese.

### Symposium

### [1S09e]

### Various possibilities of intracellular Ca2+ signaling in the field of medicine, pharmacology, dentistry, agriculture, and food sciences

March 28, 16:30 - 18:30, Room 9

### [1S09e-1]

### Doxorubicin-Induced Cardiotoxicity: Insights into $Ca^{2+}$ Signaling in Human Cardiac Fibroblasts

*Masanari Umemura[1], Soichiro Ishikawa[1], Masatoshi Narikawa[3], Hiroko Nemoto[2], Chihiro Hayashi[1], Yuto Mizuno[1], Yoshihiro Ishikawa[1] (*[1]Yokohama City University Graduate School of Medicine, CVRI, [2]Yokohama City University Graduate School of Medicine, Cardiovascular Surgery, [3]Yokohama City University, Graduate School of Medicine, Cardiovascular Medicine*)

Doxorubicin (DOX)-induced heart failure is associated with a poor prognosis, and early detection and effective treatment methods remain elusive. DOX induces oxidative stress in the cell membrane of cardiomyocytes. Several studies have identified various mechanisms of cardiotoxicity, including DNA intercalation, topoisomerase II inhibition, apoptosis, mitochondrial dysfunction, autophagy, ferroptosis, inflammatory cytokines, and calcium ($Ca^{2+}$) homeostasis. Up to 70% of all myocardial cells are non-myocyte cells, which include cardiac fibroblasts, endothelial cells, and vascular smooth muscle cells. Among these, fibroblasts play a crucial role in producing extracellular matrix (ECM) proteins and humoral factors such as transforming growth factor-β (TGF-β), interleukin (IL)-6, and platelet-derived growth factor (PDGF). However, the specific mechanism of DOX on cardiac fibroblasts has not been fully elucidated. In this study, we investigated the effect of DOX on cardiac fibroblasts and found that DOX induced their trans-differentiation into myofibroblasts. Animal studies demonstrated that a lower dose of DOX (4 mg/kg/week for 3 weeks, *i.p.*) induced perivascular fibrosis in mice without causing cell death in the heart. In human cardiac fibroblasts (HCFs) culture cells, DOX enhanced the expression of α-smooth muscle actin (α-SMA), a marker of trans-differentiation, indicating that DOX promoted the transformation of HCFs into myofibroblasts.Furthermore, DOX increased the mRNA and protein expression of matrix metalloproteinase (MMP)-1 at concentrations below 0.1 μM without inhibiting HCFs cell viability. This effect was mediated through the phosphatidylinositol-3 kinase (PI3K)/Protein Kinase B (Akt) pathway. DOX also elevated IL-6 levels via the TGF-β/Smad pathway and induced mitochondrial damage, leading to increased expression of IL-1 through stress-activated protein kinases (SAPK)/c-Jun NH-2 terminal kinase (JNK). A recent study found that ORAI1, a calcium channel protein on the cell membrane responsible for store-operated calcium entry (SOCE), is increased in cardiac fibroblasts from failing ventricles. Notably, we also uncovered that ORAI1, a critical component of SOCE, plays a role in regulating p53 expression, which leads to cell apoptosis in HCFs. ORAI1 was found to be highly expressed in HCFs. The SOCE inhibitor YM-58483 attenuated DOX (40 mg/kg)-induced apoptosis in murine models. In conclusion, our findings suggest that DOX promotes reactive fibrotic changes and induces cell apoptosis in HCFs. These observations shed light on potentially novel mechanisms underlying DOX-induced cardiotoxicity in clinical settings.

### [1S09e-2]

### Calcium homeostasis derangement by anticancer tyrosine kinase inhibitors

*Hiroko Izumi-Nakaseko[1], Ai Goto[1], Ryuichi Kambayashi[1], Koki Chiba[2], Yoshinori Takei[1], Yuko Sekino[3,4], Atsuhiko T. Naito[5], Yasunari Kanda[6], Atsushi Sugiyama[1] (*[1]Dept. Pharmacol., Fac. Med., Toho Univ., [2]Dept. Traditional Med., Fac. Med., Toho Univ., [3]Grad. Sch. Agricultural and Life Sci., Univ. Tokyo, [4]Inst. Drug Discovery Innovation, [5]Dept. Physiol., Div. Cell Physiol., Grad. Sch. Med., Toho Univ., [6]Div. Pharmacol., NIHS*)

Some anticancer drugs have been reported to induce various types of arrhythmias and ventricular dysfunction acutely as well as chronically. For example, tyrosine kinase inhibitors (TKIs): sunitinib, lapatinib, dasatinib and imatinib have been shown to induce several cardiovascular adverse events, such as hypertension, vascular impairment, left ventricular dysfunction and/or QT prolongation. We have assessed the cardiac effects of these TKIs using *in vivo* anesthetized canine models and *in vitro* hiPSC-derived cardiomyocytes sheets. In the *in vivo* study, each drug suppressed the ventricular active relaxation, whereas sunitinib and lapatinib enhanced the ventricular active contraction, but dasatinib and imatinib suppressed it. The impaired ventricular relaxation by the drugs was followed by the elevation in left ventricular end-diastolic pressure. They prolonged QT interval along with $J\text{-}T_{peak}$ or $J\text{-}T_{peak}c$ which is one of the surrogate markers of torsade de pointes. In the *in vitro* study, dasatinib and imatinib slowed the conduction, delayed the repolarization, obscured the positive contraction velocity-frequency relationship and slowed the early relaxation in a concentration-dependent manner. They reduced the time to peak of $Ca^{2+}$ transient, increasing the contraction velocity. They also decelerated decay in an early phase of Ca transients, decreasing the early-relaxation velocity. Imatinib induced early afterdepolarization and decreased synchrony in motion; the latter was correlated with conduction delay at >300 μm from the paced site. Thus, Each TKI impaired the relaxation *in vivo* and *in vitro*, indicating an occurrence of delayed $Ca^{2+}$ uptake via sarco/endoplasmic reticulum $Ca^{2+}$-ATPase, which would increase $Na^{+}/Ca^{2+}$ exchanger contribution to the elimination of cytosolic $Ca^{2+}$, leading to a delayed repolarization period.

### [1S09e-3]

### Occlusal disharmony causes cardiac dysfunction via dysregulation of calcium signaling in cardiomyocytes ~ Usefulness of pharmacological inhibition of cardiac adenylyl cyclase by vidarabine, an anti-herpes agent ~

*Kenji Suita[1], Yoshio Hayakawa[1,2], Yoshiki Onuki[1], Satoshi Okumura[1] (*[1]Department of Physiology, Tsurumi University School of Dental Medicine, [2]Department of Dental Anesthesiology, Tsurumi University School of Dental Medicine*)

Oral health decreases with age, and this is one of major risk factors for many diseases including cardiovascular disease (CVD). Occlusal disharmony (OD) is caused by various oral conditions such as loss of teeth and inappropriate vertical dimension of crowns, bridges, or dentures. We recently reported a positive relationship between OD and CVD via chronic activation of β-adrenergic receptor (β-AR) signaling in mice. In recent years, catecholamine-induced oxidative stress and hyperphosphorylation of proteins related to calcium handling such as phospholamban (PLN) and ryanodine receptor have been considered to cause intracellular calcium abnormalities in cardiomyocytes; and the calcium dysregulation is likely to play important roles in the pathogenesis of CVD. Although the usefulness of β-AR antagonists (β-blockers) for the treatment of CVD is established, the undesirable side effect suppressing basal cardiac function significantly limits their clinical usage. Adenylyl cyclase (AC) transduces the signal generated by the binding of catecholamines to β-AR, resulting in an increased production of cAMP in cardiomyocytes. Previously, we found that vidarabine, an anti-herpes agent, is a selective inhibitor of cardiac AC subtype. Here, we evaluated the effect of vidarabine on OD-induced cardiac dysfunction in mice.

OD was induced by bite-opening (BO) in mice with cementing a suitable appliance onto the mandibular incisors for a period of 2 weeks. Vidarabine (15 mg/kg/day) was administered into mice via subcutaneously implanted osmotic minipumps from 2 days before the start of BO treatment to the end. After 2 weeks, the cardiac function (left ventricular ejection fraction and fractional shortening) was significantly decreased, concomitantly with increased cardiac fibrosis and myocardial apoptosis, in BO mice as compared to control. In cardiomyocytes, oxidative DNA damage and protein oxidation was increased by BO. The BO-induced cardiac dysfunction was associated with increased PLN phosphorylation, as well as increased activation of calcium-calmodulin-dependent protein kinase II/ receptor-interacting protein 3 signaling pathway. Vidarabine significantly attenuated the BO-induced pathological cardiac changes without adverse effect on basal cardiac function. These results suggest that pharmacological inhibition of cardiac AC with vidarabine ameliorates the OD-induced cardiac dysfunction via reduction of oxidative stress and prevention of intracellular calcium dysregulation.

### [1S09e-4]

### CHERP and ALG-2 Regulate Calcium-Dependent Alternative Splicing via Interaction with Chromatin

*Ken-ichi Fujita[1,2], Takaki Ishizuka[3], Akila Mayeda[2], Seiji Masuda[3] ([1]*National Cancer Center Research Institute,* [2]*Fujita Health University,* [3]*Kindai University*)

Intracellular calcium signaling is important for the control of broad cellular processes, some of which are mediated by the regulation of alternative splicing. However, the mechanism of calcium-dependent alternative splicing is still poorly understood. The nuclear factor CHERP ($Ca^{2+}$ homeostasis endoplasmic reticulum protein) interacts with an essential splicing factor U2 snRNP and it functions in genome-wide splicing regulation. Interestingly, CHERP binds with the calcium-binding factor ALG-2, however, its physiological significance remains to be elucidated. Here, we hypothesize that CHERP functions cooperatively with ALG-2 to regulate calcium-dependent alternative splicing. Using whole transcriptome analysis of CHERP- and ALG-2-depleted cells, we found that both factors cooperatively regulate splicing. Next, we analyzed the whole transcriptome of CHERP- and ALG-2-depleted cells after treatment with intracellular calcium-upregulating reagents (Thapsigargin or A23187). Together, These results revealed that CHERP and ALG-2 mediate most of the alternative splicing changes that responded with intracellular calcium concentration. To investigate the mechanism of action, we characterized the calcium-dependent interactome of CHERP. Surprisingly, we observed that CHERP, together with ALG2, binds to the histone core in a calcium-dependent manner. Recently, it has been reported that splicing factors associated with chromatin determine alternative exon recognition. Therefore, we propose a novel model that the calcium-dependent binding of CHERP and ALG2 to chromatin regulates calcium-dependent alternative splicing.

### [1S09e-5]

### Effects of omega-3 polyunsaturated fatty acids on cardiac rhythm, electrical excitability, and $Ca^{2+}$ signaling

*Masaki Morishima[1] ([1]*Kindai University*)

Epidemiological studies have established the impact of obesity as an independent risk factor for atrial fibrillation (AF), which highlighted the role of high fat diet. Although many studies have shown that reduced cardiac L-type $Ca^{2+}$ channel expression is a known causal mechanism of AF, underlying pathophysiological mechanisms for obesity-mediated AF are not clarified. Cardiovascular benefits of dietary omega-3 polyunsaturated fatty acids, including eicosapentaenoic acid (EPA), have been actively investigated for many years. However, the mechanisms for these potential benefits on cardiac rhythm are complex and not well defined. The objective of this study was to review and evaluate effects of EPA on cardiomyocyte focusing on the L-type $Ca^{2+}$ channel and a transcription factor adenosine-3', 5'-cyclic monophosphate response element binding protein (CREB). Transesophageal burst pacing invariably induced AF (100%) in high fat diet (HFD)-induced obesity mice, whereas AF was induced less frequently (50%) in EPA+HFD mice. Masson's trichrome staining revealed interstitial fibrosis in the left atrium (LA) of HFD mice, which was not observed in EPA+HFD mice. Downregulation of the Cav1.2-L-type $Ca^{2+}$ channel and the Nav1.5-$Na^{+}$ channel mRNA levels was demonstrated in HFD mice LA, but not in EPA+HFD mice LA. In addition, EPA rescued a decrease of spontaneous beating rate, L-type $Ca^{2+}$ channel current, Cav1.2 mRNA, and protein expressions of the Cav1.2 caused by a mixture of oleic acid (OA) and palmitic acid (PA) or OAPA in isolated neonatal mice cardiomyocytes. Immunocytochemical analysis revealed a distinct downregulation of the Cav1.2 channel by OAPA with a concomitant decrease in the phosphorylated component of CREB in the nucleus, which was also rescued by EPA. Transcriptional regulation of Cav1.2 by EPA was blocked by a free fatty acid receptor 4 (FFAR4) antagonist AH7614. Furthermore, EPA shortened the time to the peak and accelerated the decay of the $Ca^{2+}$ transient in Fluo-4 loaded cardiomyocytes. These results suggest that EPA rescues $Ca^{2+}$ overload caused by OAPA lipotoxicity through the FFAR4/CREB/Cav1.2-mediated pathways, which might imply a novel promising target for the management of arrhythmias.

### Symposium

### [1S10e]
**Another face of PDGFRα positive cells -A novel smooth muscle pacemaker-**

March 28, 16:30 - 18:30, Room 10

### [1S10e-1]
**Overview ~Roles of PDGFRα(+) cells in smooth muscle spontaneous activity~**

*Hikaru Hashitani[1] ([1]*Dept. Cell. Physiol, Grad. Sch. Med. Sci., Nagoya City Univ*)

Smooth muscle tissues, particularly those in visceral organs and small vessels develop spontaneous contractions arising from transient depolarisations. In the gastrointestinal tract where Kit (+) interstitial cells of Cajal serve as pacemaker cells to electrically drive smooth muscle cells (SMCs), interstitial cells expressing PDGFRα, a marker of fibroblasts, appear to function as a counteracting system to stabilise SMC excitability. Thus, PDGFRα (+) interstitial cells generate hyperpolarising signals by the opening of SK3 potassium channels that transmit to SMCs. Despite the wide distribution of Kit(+) or PDGFRα (+) cells in a variety of smooth muscle tissues, their roles in the generation or modulation of spontaneous activity have been proven in only a limited number of tissues. Recently, PDGFRα (+) smooth muscle or interstitial cells were revealed to exert a role in pacemaking of several smooth muscle tissues. In the renal pelvis, atypical SMCs, known pacemaker cells for pyeloureteric peristalsis, express PDGFRα and develop spontaneous transient depolarisations, events known to underlie pacemaker potentials. In seminal vesicle, PDGFRα (+) subepithelial interstitial cells distributed in the mucosa generate electrical slow waves to drive SMCs. In rat caudal epididymis, an innermost layer of thin SMCs *co-express* α-SMA and PDGFRα as do atypical SMCs in the renal pelvis. Notably, isolated cells that may correspond to these α-SMA (+) PDGFRα (+) cells generate spontaneous transient inward currents. Interestingly, PDGFRα (+) cells in all three smooth muscle tissues share a common pacemaker mechanism, namely the expression of $Ca^{2+}$-activated $Cl^-$ channels (CaCCs), including TMEM16A/ANO1, the pacemaker channel of Kit (+) ICC. Thus, cytosolic $Ca^{2+}$ oscillator linking to CaCCs appears to be an ubiquitous mechanism underlying smooth muscle spontaneous activity. Up-to-date knowledge of the roles and mechanisms underlying PDGFRα (+) cell-driven smooth muscle spontaneous activity will be discussed.

### [1S10e-2]
**Roles of PDGFRα (+) cells in renal pelvis pacemaking**

*Nathan Grainger[1], Sei Kim[1], Emily Fox[1], Kenton Sanders[1] ([1]*University of Nevada, Reno School of Medicine*)

The upper urinary tract (UUT) plays a crucial role in removing urine from the kidneys. Besides aiding in waste removal, the UUT also helps filter solutes and prevent nephron damage by minimizing intratubular back pressure. Once the final urine is formed, it enters the UUT through renal papillae and calyces. As urine fills the calyces, the muscles contract, pushing it into the renal pelvis, from where it is distributed to the ureters and the bladder for storage and voiding. Specialized UUT pacemaker cells that express smooth muscle markers, known as atypical smooth muscle cells (ASMCs), provide a constant, depolarizing signal to the electrically coupled contractile typical SMCs to facilitate peristaltic contractions. ASMC density decreases from the top of the upper urinary tract to the ureter, ensuring that peristalsis occurs in an antegrade direction. Although the mechanisms of renal pacemakers are not entirely determined, transient inward currents generated by $Cl^-$ and non-selective cation channels are believed to evoke spontaneous depolarizations. Recent studies have identified ways to distinguish pacemaker cells from contractile typical SMCs that facilitate peristalsis, including co-expression of the cell surface tyrosine kinase receptor, PDGFRα, and smooth muscle myosin. Significantly, PDGFRα (+) ASMCs co-express the $Ca^{2+}$-activated $Cl^-$ channel, ANO1 (or TMEM16A), which is known to mediate gastrointestinal pacemaking. This talk will discuss the mechanisms of renal pelvis pacemaking, the functional expression of ANO1 in PDGFRα (+) ASMCs, findings from transgenic studies that manipulate UUT ANO1 expression, and recent advances in identifying pacemaker cells with RNA-sequencing technologies.

### [1S10e-3]
**Identification of PDGFRα$^+$ subepithelial interstitial cells as a pacemaker in the guinea pig seminal vesicles**

*Mitsue Takeya[1], Kei-ichiro Nakamura[2], Makoto Takano[1] ([1]*Division of Integrated Autonomic Function, Department of Physiology, Kurume University School of Medicine,* [2]*Cognitive and Molecular Research Institute of Brain Diseases, Kurume University School of Medicine*)

Seminal vesicles (SVs), a pair of male accessory glands, vigorously contract upon nerve excitation during ejaculation, while developing spontaneous phasic contractions (SPCs) during the inter-ejaculatory storage phase. In guinea pig SVs, stretch-induced SPCs resulting from electrical slow waves and corresponding $Ca^{2+}$ flashes are generated in a unique mucosa-dependent manner, as such spontaneous activities in the SV smooth muscle are abolished by the removal of mucosal layer (Takeya et al. 2017; PMID: 28421606). The pacemaker activity in the SVs appears to originate in subepithelial interstitial cells (SICs) expressing platelet-derived growth factor receptor α (PDGFRα). In the basal layer of mucosa separated from the SV muscular layer, PDGFRα$^+$ SICs are capable of developing synchronous spontaneous $Ca^{2+}$ oscillations within their network and corresponding electrical slow waves. Strikingly, spontaneous $Ca^{2+}$ transients in the SICs are synchronized with those in SV smooth muscle cells (SMCs). Dye-coupling between SICs and SMCs further suggests the notion that SICs electrically drive the SPCs of SV SMCs by sending slow waves via gap junctions (Takeya et al. 2022; PMID: 35081665). Electrical pacemaker activity in PDGFRα$^+$ SICs appears to arise from cyclic endoplasmic reticulum (ER) $Ca^{2+}$ release linked with the opening of $Ca^{2+}$-activated chloride channels, resulting in depolarizations to activate L-type voltage-dependent $Ca^{2+}$ channels (LVDCCs). Since PDGFRα$^+$ SICs are immunoreactive for $P2Y_1$ receptor and respond to $P2Y_1$ agonists by developing $Ca^{2+}$ transients, purinergic signaling may play a role in maintaining SV spontaneous activity. Involvement of TMEM16A/ANO1 in pacemaker currents of PDGFRα$^+$ SICs remains to be explored. In addition, ion channels that account for the mechano-sensitivity of spontaneous activity are of great interest.

### [1S10e-4]

### Pacemaker mechanisms in the epididymis

*Dirk Ferdinand van Helden[1], Retsu Mitsui[2], Hikaru Hashitani[2] ([1]*The University of Newcastle, Australia,* [2]*Nagoya City University*)

The epididymis is present in male mammals, birds and reptiles and underlies transportation and maturation of sperm. Here we consider pacemaker mechanisms that drive smooth muscle (SM) contractions in this organ. The primary components of the epididymis are the initial segment, caput, corpus, and cauda where the former three, henceforth referred to as the proximal duct, facilitate transport and maturation of sperm with the cauda providing temporary storage of sperm. An interesting feature in the epididymis is that the pacemaker mechanism in the proximal duct is different to that in the proximal cauda, the distal cauda being quiescent in the absence of stimulation. Pacemaking in the proximal duct is primarily dependent on L-type Ca and K channels in the cell membrane (Mewe et al. 2006; PMID: 16855213) and is thus considered a membrane clock-based pacemaker whereas pacemaking in the proximal cauda is generated by rhythmical release of $Ca^{2+}$ from intracellular stores and the opening of $Ca^{2+}$-activated $Cl^-$ channels ($Cl^-_{Ca}$) and hence is a calcium clock-based pacemaker (Mitsui et al. 2021; PMID: 34596752). The epididymis has interstitial cell networks (ICs) that are immunoreactive to platelet-derived growth factor receptor α (PDGFRα) which intertwine and/or run near to the contractile SM (Hiroshige et al. 2021; PMID: 34298316). These are present in both the proximal duct and cauda and represent candidate pacemaker cell networks. Should they subserve this role then the difference between the pacemaker mechanisms in the proximal duct and cauda of the epididymis remains to be explained. However, from an operational perspective, separate pacemaker mechanisms in these epididymal regions will allow functional control over the movement of sperm in regions that serve very different purposes.

### Symposium

[2S03m]
### Diversity and Prospect of Cardiac Physiology

March 29, 8:50 - 10:50, Room 3

### [2S03m-1]
### Pathophysiology of diabetic embryopathy

*Atsushi Nakano[1,2] ([1]*The Jikei University,* [2]*University of California Los Angeles*)

Mounting evidence supports an instructive role for metabolism in stem cell fate decisions. However, much is unknown about how fetal metabolism changes during mammalian development and how altered maternal metabolism shapes fetal metabolism. Here, we present a descriptive atlas of in vivo fetal murine metabolism during mid-to-late gestation in normal and diabetic pregnancies. Using 13C-glucose and LC-MS, we profiled the metabolism of fetal brains, hearts, livers, and placentas harvested from pregnant dams between embryonic days (E)10.5 and 18.5. Comparative analysis of our large metabolomics dataset revealed metabolic features specific to fetal tissues developed under a hyperglycemic environment and metabolic signatures that may denote developmental transitions during euglycemic development. We observed sorbitol accumulation in fetal tissues and altered neurotransmitter levels in fetal brains isolated from dams with maternal hyperglycemia. Tracing 13C-glucose revealed disparate nutrient sourcing in fetuses depending on maternal glycemic states. Regardless of glycemic state, histidine-derived metabolites accumulate in fetal tissues and maternal plasma during late development. Our rich dataset presents a comprehensive overview of in vivo fetal tissue metabolism and alterations occurring as a result of maternal hyperglycemia.

### [2S03m-2]
### Maturation of Pluripotent Stem Cell Derived Cardiomyocytes for Disease Modeling in vitro

*Hideki Uosaki[1] ([1]*Division of Regenerative Medicine, Center for Molecular Medicine, Jichi Medical University*)

Pluripotent stem cell-derived cardiomyocytes (PSC-CMs) show great potential for replicating disease phenotypes in vitro. However, due to their immaturity, PSC-CMs are not yet perfect disease models. In order to obtain mature PSC-CMs, we aimed to understand the molecular mechanism underlying the cardiomyocyte maturation process in vivo. First, we performed a transcriptional network analysis and identified transcriptional factors and nuclear receptors that are activated during maturation. Our overexpression screening revealed that PGC1α/β are two of the top regulators of CM maturation. Knock-out of both PGC1α/β resulted in immature CMs in vivo. As PGC1α/β are co-receptors for nuclear receptors, we further screened and identified the best combinations of nuclear receptor agonists to promote maturation of PSC-CMs. With these combinations, mouse PSC-CMs became more similar to adult cells, while human PSC-CMs became more mature but still juvenile. Finally, we tested if the agonist cocktail could exaggerate disease phenotypes of cardiomyopathy. To this end, we generated a large cohort of mitochondrial cardiomyopathy (MCM) iPSCs and demonstrated that the agonist cocktail induced massive hypertrophy in MCM-iPSC-CMs. This study suggests that the maturation of PSC-CMs can help replicate cardiomyopathy phenotypes in vitro.

### [2S03m-3]
### Intracellular Organelle Calcium Handling: Implications for Cardiac Arrhythmias

*Lai-Hua Xie[1] ([1]*Rutgers University-New Jersey Medical School*)

Calcium ions ($Ca^{2+}$) are precisely regulated within various intracellular compartments, including the sarcoplasmic reticulum (SR), mitochondria, and lysosomes under physiological condition. We aim to investigate the dysregulation of intracellular organelle $Ca^{2+}$ dynamics and its significance in relation to arrhythmogenesis. We observed that the protonophore FCCP depolarized the mitochondrial membrane potential ($\Delta\psi m$) and increased the frequency and amplitude of $Ca^{2+}$ waves (CaWs) in a dose-dependent manner. FCCP also increased the amplitude and frequency of delayed afterdepolarization and induced triggered action potentials. FCCP reversibly raised basal intracellular $Ca^{2+}$ levels even after depletion of SR $Ca^{2+}$ and in the absence of external $Ca^{2+}$, suggesting $Ca^{2+}$ release from mitochondria. The effects of FCCP on CaWs were counteracted by the mitochondrial permeability transition pore (mPTP) blocker cyclosporine A, or the mitochondrial $Ca^{2+}$ uniporter activator kaempferol. These results suggest that mitochondrial $Ca^{2+}$ release and uptake exquisitely control the local $Ca^{2+}$ level in the micro-domain near SR ryanodine receptors and play an important role in regulation of intracellular CaWs and arrhythmogenesis. This concept was validated using a cyclophilin D knockout (CypD KO) mouse model and a pathological iron-overload condition, in addition to computer simulation approaches. FCCP caused the $\Delta\psi m$ depolarization to the same extent in cardiomyocytes from both WT and CypD KO mice, however, CypD KO cardiomyocytes exhibited a lower level of mPTP opening than WT cardiomyocytes. FCCP caused significant increases in CaW frequency in WT cardiomyocytes but not in CypD KO cardiomyocytes. Consistently, WT hearts exhibited a significantly higher average arrhythmia score than CypD KO hearts subjected to FCCP treatment or chemical ischemia-reperfusion. We also demonstrated that iron overload induced mitochondrial ROS generation and $\Delta\psi m$ depolarization, thereby opening the mPTP and promoting CaWs and cardiac arrhythmias. Conversely, the inhibition of mPTP ameliorates the proarrhythmic effects of iron overload. Treatment with nicotinic acid dinucleotide phosphate (NAADP), an agonist of the two-pore channel in the lysosomal membrane, increased the amplitude of the $Ca_i$ transient and induced spontaneous CaWs. Lysosomes are also recognized as iron stores. We found that NAADP induced an increase in cytosolic and mitochondrial iron levels, suggesting a potential translocation. In conclusion, the intracellular organelles act as intricate reservoirs, precisely releasing and sequestering $Ca^{2+}$ to orchestrate the heart's function. Dysregulation of this balance can lead to abnormal $Ca^{2+}$ dynamics and electrical impulses, contributing to arrhythmias.

### [2S03m-4]

### Cardiac conduction system research: pathophysiology for future antiarrhythmic therapies

*Shu Nakao[1] ([1]*Dept of Physiology, Tokai University School of Meicine*)

The cardiac conduction system (CCS) is a specialized pacemaker tissue responsible for generating and propagating electrical impulses in the heart. Impulses are regularly generated in the sinus node, the primary pacemaking site located in the right atrium, and subsequently travel to the atrioventricular node through the atrial wall. Slowed conduction in the atrioventricular node rapidly conveys to the ventricular myocardium through the His-Purkinje network, enabling highly coordinated myocardial pumping motion for systemic blood supply. Although investigating the CCS is challenging due to its complex anatomy and function, accumulated evidence reveals that the CCS is highly heterogeneous in terms of electrophysiology, histology, and development. CCS electrophysiology, representing cardiac automaticity, is characterized by orchestration of pacemaking ion channels and transporters. The CCS is insulated within a dense collagen meshwork and is accompanied by nerve fibers, capillaries, and immune cells. In recent decades, extensive research efforts have revealed that pathological conditions cause transcriptional and functional ion channel remodeling in the CCS, resulting in various arrhythmias. miRNAs and inflammation can be involved in these CCS dysfunctions. Aging and heart disease also lead to structural CCS remodeling. Severe loss of pacemaker cells and conduction fibers with fibrofatty replacement is observed in the sinus node and atrioventricular node of elderly patients with bradycardia and heart block. Future studies will identify corresponding signal transduction pathways for these pathologies. The CCS initially develops with atrial tissue, and it is then specified with a pacemaker gene program that inhibits the atrial program. However, the underlying mechanisms of CCS development and pacemaker cell specification remain elusive. Next-generation sequencing analyses have identified several genes that are differentially expressed in the CCS. Future investigations into these gene functions for spontaneous action potential firing will provide insights into illustrating the transcriptional network of the pacemaker gene program. This knowledge can be applied to the development of a 'biological pacemaker' as a cell source for regenerative medicine.

### [2S03m-5]

### Explore the pathogenesis of inherited arrhythmia syndromes from the view of electrophysiology.

*Seiko Ohno[1], Keiko Sonoda[1], Koichi Kato[2], Takeru Makiyama[3] ([1]*National Cerebral and Cardiovascular Center,* [2]*Shiga University of Medical Science,* [3]*Kyoto University Graduate School of Medicine*)

IInherited arrhythmia syndromes (IAS) are mainly caused by pathogenic variants in genes related with ion channels, and some of them lead to sudden cardiac death due to lethal ventricular arrhythmias. Among IAS, Long QT syndrome (LQTS) is characterized by QT prolongation in the ECG and peculiar ventricular tachycardia called as torsade de pointes. Ventricular arrhythmias in catecholaminergic polymorphic ventricular tachycardia (CPVT) are induced by exercise or emotional stress, and no abnormal finding is observed in the ECG at rest. Among the causative genes of IAS, we here focus on genes encoding calmodulin and *RYR2* encoding cardiac ryanodine receptor channel (RyR2), both are related with calcium dynamics.Calmodulin is a $Ca^{2+}$ binding protein encoded by three genes, *CALM1*, *CALM2* and *CALM3* located on different chromosomes. Variants in *CALM1* were reported as the cause of CPVT in 2012, and *CALM1* and *CALM2* variants in LQTS in 2013. These variant calmodulins changed the $Ca^{2+}$ affinity and RyR2 binding affinity. These functional changes in variant calmodulins induce abnormal calcium dynamics and related with the lethal arrhythmias. Until now, more than 60 variants in *CALM1-3* were reported, and some of them were analyzed using stable cell lines, iPS derived cardiomyocytes and mouse models. RyR2 is located on the sarcoplasmic reticulum (SR) and release $Ca^{2+}$ from SR following the inflow of $Ca^{2+}$ from L type calcium channel in T tubules. Gain of function type variants in *RYR2* are the major cause for CPVT. Excessive release of $Ca^{2+}$ from SR induces the delayed after depolarization and leads to the lethal ventricular arrhythmia. Recently, loss of function type *RYR2* variants have been reported in the patients with lethal arrhythmia without exercise/emotional stress. The disease is called as cardiac ryanodine receptor calcium release deficiency syndrome (CRDS). In the functional analysis using HEK293 cells, variants identified in CRDS showed high activation threshold of store overload induced $Ca^{2+}$ release from SR and low open probability in single RyR2. We also identified two loss of function type variants in patients with mild phenotypes: LQTS and infantile bradycardia. Compared to the variants causative for lethal arrhythmias, our variant RyR2 showed complete loss of function and no $Ca^{2+}$ release from RyR2.In conclusion, variants in calmodulin and RyR2 affect the $Ca^{2+}$ dynamics and leads to lethal arrythmias. Genetic testing in patients with lethal ventricular arrhythmias and further functional analysis would lead to the effective treatment for IAS.

### Symposium

[2S04m]
International Relations Committee
**Old players take on new roles: various $Ca^{2+}$ signaling regulators provide novel mechanisms in cardiac metabolism and disease**

March 29, 8:50 - 10:50, Room 4

### [2S04m-1]

### Cereblon contributes to cardiac dysfunction by degrading $Ca_v1.2α$

*Jin Han[1] ([1]*Cardiovascular and Metabolic Disease Center, Inje University*)

Cereblon (CRBN) is a substrate receptor of the E3 ubiquitin ligase complex that was reported to target ion channel proteins. L-type voltage-dependent $Ca^{2+}$ channel (LTCC) density and dysfunction is a critical player in heart failure with reduced ejection fraction (HFrEF). However, the underlying cellular mechanisms by which CRBN regulates LTCC subtype $Ca_v1.2α$ during cardiac dysfunction remain unclear. Here, we explored the role of CRBN in HFrEF by investigating the direct regulatory role of CRBN in $Ca_v1.2α$ activity and examining how it can serve as a target to address myocardial dysfunction. Cardiac tissues from HFrEF patients exhibited increased levels of CRBN compared with controls. *In vivo* and *ex vivo* studies demonstrated that whole-body *CRBN* knockout ($CRBN^{-/-}$) and cardiac-specific knockout mice ($Crbn^{fl/fl}$/$Myh6^{Cre+}$) exhibited enhanced cardiac contractility with increased LTCC current ($I_{CaL}$) compared with their respective controls, which was modulated by the direct interaction of CRBN with $Ca_v1.2α$. Mechanistically, the Lon domain of CRBN directly interacted with the N-terminal of $Ca_v1.2α$. Increasing CRBN levels enhanced the ubiquitination and proteasomal degradation of $Ca_v1.2α$ and decreased $I_{CaL}$. In contrast, genetic or pharmacological depletion of CRBN via TD-165, a novel PROTAC-based CRBN degrader, increased surface expression of $Ca_v1.2α$ and enhanced $I_{CaL}$. Low CRBN levels protected the heart against cardiomyopathy *in vivo*. Cereblon selectively degrades $Ca_v1.2α$, which in turn facilitates cardiac dysfunction. A targeted approach or an efficient method of reducing CRBN levels could serve as a promising strategy for HFrEF therapeutics.

### [2S04m-2]

### Distinct alterations in local $Ca^{2+}$ signaling in right and left atrial myocytes by acute and chronic mechanical stress

*Sun-Hee Woo[1] ([1]*Chungnam National University*)

$Ca^{2+}$ releases in the interior of atrial myocytes, lacking t-tubules, govern their contractility, but regulatory mechanisms for central $Ca^{2+}$ releases are poorly understood. Loss of atrial contractility is associated with atrial blood stasis and decrease of ventricular filling at diastole. The conditions, such as atrial fibrillation (AF), can be caused by hemodynamic disturbances including shear stress and pressure overload. AF indeed occurs more often in left atrial (LA)- than right atrial (RA)-chamber. We investigated if and how $Ca^{2+}$ releases from central non-junctional versus peripheral junctional sites are altered by prolonged increase in afterload and by high fluid shear stress, and compared these responses between LA and RA myocytes. Rapid 2-D confocal $Ca^{2+}$ imaging was used to simultaneously measure peripheral and non-junctional (central) $Ca^{2+}$ releases in rat atrial myocytes. Transverse aortic constriction (TAC) for >20 weeks was used to induce left heart failure (HF) in rats. Shear stress was applied onto single cell by pressurized fluid-puffing. Shear stress transiently enhanced peripheral and central $Ca^{2+}$ releases on depolarization with much higher stimulation on non-junctional sites, which was followed by attenuations of $Ca^{2+}$ transients at both sites. High frequency of depolarizations (3 Hz) removed this shear-induced prolonged central $Ca^{2+}$ release in LA cells, but not in RA cells. During the HF development, $Ca^{2+}$ transients were reduced in LA cells but enhanced in RA cells with a similar decrease in their SR $Ca^{2+}$ stores. In addition, central release efficacy at a given peripheral release on depolarization was increased in both RA and LA cells. Peripheral release in LA cells from TAC rats was distinctly reduced more when the central release was enhanced in TAC RA myocytes. Decay and rise of $Ca^{2+}$ signals were somewhat accelerated in TAC RA cells, whereas they were slowed in TAC LA cells. Immunoblotting analyses revealed that RA and LA cells from TAC rats have differential profile of $Ca^{2+}$ signaling toolkit gene expressions, in particular, for ryanodine receptor, L-type $Ca^{2+}$ channel, $Na^+$-$Ca^{2+}$ exchanger and SR $Ca^{2+}$ ATPase, which was consistent with distinct alterations in local $Ca^{2+}$ signaling between RA and LA cells. Our data suggest that central $Ca^{2+}$ signaling of LA myocytes are highly vulnerable under shear stress with high stimulation frequency and prolonged pressure overload compared with RA cells mainly due to deterioration of peripheral $Ca^{2+}$ signaling and lower level of SR $Ca^{2+}$ ATPase.

### [2S04m-3]

### Heart failure controlled by isoform-specific functions of TRPC proteins

*Motohiro Nishida[1,2] ([1]*Department of Physiology, Graduate School of Pharmaceutical Sciences, Kyushu University,* [2]*Division of Cardiocirculatory Signaling, National Institute for Physiological Sciences (Exploratory Research Center on Life and Living Systems), National Institutes of Natural Sciences*)

Transient receptor potential (TRP) family proteins form membrane channels that sense a variety of extracellular physicochemical stimuli such as temperature, osmotic pressure, oxygen, and reactive oxygen species. TRP channel is also focused as an award-winning research theme in the 2021 Nobel Prize in Physiology or Medicine. TRP channels have also attracted attention as disease targets, but effective therapeutic agents have not yet been developed. Canonical TRP (TRPC) proteins are the molecular entity of a receptor-operated cation channel and activated downstream of all neurohumoral and growth factors that stimulate phospholipase C-linked receptors modulating cardiovascular functions.
We previously found that the upregulation of TRPC3 and TRPC6, both of which form diacylglycerol-activated cation channels, are involved in the hypertrophic growth of rat cardiomyocytes to physicochemical stress stimuli. After that, however, analysis using TRPC-deficient mice revealed that the pathophysiological functions of TRPC3 and TRPC6 were different. Especially, the protein scaffolding of TRPC3 protein with ROS-generating enzyme, NADPH oxidase (Nox) 2, rather than TRPC3 channel activity, is essential to develop myocardial atrophy and interstitial fibrosis. We also found that TRPC6-mediated $Zn^{2+}$ influx positively regulates baroreflex-dependent cardiac positive inotropy through potentiating bAR-Gs axis in cardiomyocytes. Pharmacological perturbation of TRPC3-Nox2 protein complex formation or activation of TRPC6-mediated $Zn^{2+}$ influx improves chronic heart failure and mortality in mice. These results strongly suggest that both TRPC3 and TRPC6 are undoubted therapeutic targets for the treatment of heart failure, but the underlying pathophysiological mechanisms are quite different.

### [2S04m-4]

### Various $Ca^{2+}$ binding proteins regulating cardiac hypertrophy: similar in structure but different in function

*Tomoe Y Nakamura-Nishitani[1], Shigeo Wakabayashi[2] ([1]*Wakayama Medical University,* [2]*Osaka Aoyama University*)

Cardiac hypertrophy is a major cause of various heart diseases, and intracellular $Ca^{2+}$ signaling plays an important role in this process. There are several $Ca^{2+}$-dependent and $Ca^{2+}$-independent hypertrophic pathways, including calcineurin/NFAT-, CaMKII/HDAC-pathways, and hypoxia-induced pathways. We have previously demonstrated that various $Ca^{2+}$-binding proteins regulate cardiac hypertrophy in different ways. For example, we found that neuronal $Ca^{2+}$ sensor 1 (NCS-1), an EF-hand $Ca^{2+}$-binding protein, is up-regulated by Gq-coupled receptor stimulation and promotes cardiac hypertrophy. NCS-1 binds to the $Ca^{2+}$ release channel $IP_3Rs$ on the SR and activates CaMKII signaling, thereby increasing both SR and nuclear $Ca^{2+}$ levels, promoting the hypertrophy-related gene expression. We also demonstrated that chronic activation of $Na^+/H^+$ exchanger 1 (NHE1) with its auxiliary subunit, calcineurin homologous protein 1 (CHP1), is sufficient to induce cardiac hypertrophy by increasing intracellular $Ca^{2+}$ levels. NHE1 regulates not only intracellular pH but also intracellular $Na^+$ and $Ca^{2+}$ levels, the latter regulated by the $Na^+/Ca^{2+}$ exchanger. Using biochemical techniques, we identified calcineurin as a novel binding partner of NHE1/CHP3 complex and showed that the increase in intracellular pH and $Ca^{2+}$ induced by NHE1 activation stimulates the calcineurin/NFAT pathway and promotes cardiac hypertrophy. CHP is an EF-hand $Ca^{2+}$-binding protein like NCS-1, with three homologues CHP1-3. CHP3 is predominantly expressed in the heart, but its role in cardiomyocytes was unknown. Knockdown of CHP3 resulted in hypertrophy of cardiomyocytes. Biochemical analysis revealed that CHP3 (but not CHP1) binds to glycogen synthase kinase 3β (GSK3β), which promotes cardiac hypertrophy when phosphorylated. CHP3 inhibits phosphorylation of GSK3β, thereby negatively regulating cardiac hypertrophy. Furthermore, analysis of CHP3 knockout mice indicates a potential link between CHP3 and hypoxia induced hypertrophy. In this symposium, we will discuss how several structurally similar proteins (e.g., CHP3, CHP1, NCS-1) regulate cardiac hypertrophy by different mechanisms. COI:No.

### Symposium

[2S05m]
International Relations Committee
**Secret lives of membrane lipids in physiology**

March 29, 8:50 - 10:50, Room 5

### [2S05m-1]

### The atomic structure of voltage sensing phosphatase reveals substrate recognition mechanism

*Atsushi Nakagawa[1], Hirotaka Narita[1], Makoto Matsuda[1], Yumiko Hara[1], Eiki Yamashita[1], Yasushi Okamura[2] ([1]*Institute for Protein Research, Osaka University,* [2]*Graduate School of Medicine*)

Voltage Sensing Phosphatase (VSP) was initially identified in the ascidian, *Ciona intestinalis*, by systematic genomic survey and has been found to be conserved in various animals, from sea squirt to human. VSP is a membrane-bound enzyme that dephosphorylates phosphatidyl inositol phosphates and its activity is regulated by membrane potential changes. VSP consists of two distinct regions; a voltage sensor domain (VSD) and a cytosolic catalytic region (CCR), which consists with phosphatase domain (PD) and C2 domain, and these two regions are connected by VSD-PD liner. A voltage sensor domain is embedded in a cell membrane and is consisted of four–helix bundle of which the 4th transmembrane helix (S4) of VSD senses membrane potential with several positively charged residues on the S4 as voltage sensing ion channels. CCR shares sequence and structural similarities with a tumor suppressor gene product, phosphatase and tensin homolog deleted on chromosome 10 (PTEN), which is a tumor suppressor gene product. It is known that its mutations of this gene cause the development of many cancers. Despite of high sequence identity between cytoplasmic catalytic region of VSP and PTEN, these enzymes show different substrate specificities. Since PTEN is one of the major proteins of which mutation(s) cause high risks of cancers in human, many atomic structures, including wild–type and mutants, have been determined. Atomic structures of the complexes of various substrates of VSP gives useful information on the substrate recognition of these enzymes.
We have succeeded in solving the atomic structures of VSP, which contains both VSD and CCR, and its substrate complexes. These structures suggested the molecular mechanism of membrane voltage-enzymatic activity coupling and substrate recognition.

### [2S05m-2]

### Physiological function of PI4P/PS exchange coupled with PI4P metabolism

*Asami Kawasaki[1], Fubito Nakatsu[1] ([1]*Department of Neurochemistry and Molecular Cell Biology, Niigata University*)

Phosophatidylinositol 4-phosphate (PI4P), the most abundant spieces of phosphoinositides, has been shown to regulate various cellular processes. Recent studies particularly shed light on an emerging role of this lipid as a master regulator of intracellular lipid transport process at membrane contact sites (MCSs). MCSs represent regions where cellular membrnaes come into close proximity without fusion. MCSs have been shown to serve as zones for transfer of lipids mediated by lipid transfer proteins. Oxysterol Binding Protein (OSBP)-Related Proteins (ORPs) are a family of lipid transfer proteins. A characteristic functional property of ORPs is their lipid countertransport activity, Several, but not all, members of ORPs have been demonstrated to exchange PI4P and other lipids between cellular membranes. In this study, we identified ORP10 as a lipid exchanger at ER–endosome MCSs. ORP10 localized at the MCSs between the ER and the PI4KIIα-positive endosomes where it mediates exchange of two different lipids, phosphatidylserine (PS) and PI4P. Cell biological analysis demonstrated that ORP10 supplied PS in exchange for PI4P to endosomes where the PS-binding protein EHD1 is recruited, thereby promoting the fission process of tubulovesicular carrier from endosomes in the retrograde trafficking pathway. Thus, ORP10 is a new lipid exchanger at ER–endosome MCSs.

### [2S05m-3]

### The role and control of membrane fusion factors in skeletal muscle differentiation

*Taichiro Tomida[1], Yoshinori Mikami[1], Daisuke Ohshima[1], Yuto Tei[1], Satomi Adachi-Akahane[1] ([1]*Dept. of Physiol., Fac. of Med., Toho Univ.*)

Skeletal muscle formation relies on the fusion of numerous myoblasts to form multinucleated muscle fibers. The molecular mechanisms controlling this cell fusion process have long remained elusive. However, recent advanced studies have revealed key molecular entities responsible for cell membrane fusion, shedding light on the intricate mechanism of cell fusion and its physiological significance in muscle cell differentiation. We have been investigating intracellular signaling pathways regulating the expression of the membrane-associated fusogenic factor Myomixer. Myomixer acts on the phospholipids of adjacent cell membranes, creating pores in the hemi-fused cell membrane where the phospholipid bilayers of two cells have partially merged. Our results demonstrated the involvement of the stress-activated kinase, p38MAPK, in the initiation of cell fusion. Indeed, in an in vitro muscle cell model, inhibition of p38MAPK resulted in reduced expression of Myomixer, which prevented cell fusion and led to the persistence of mononuclear cells during muscle differentiation. p38MAPK is known to be activated in response to inflammation and cellular stress, suggesting its potential relevance in muscle regeneration following muscle injury. Deleting the gene encoding Myomixer inhibited cell fusion while reducing the expression of genes related to sarcomere formation and $Ca^{2+}$ signaling, leading to significant inhibition of voltage-induced calcium release (VICR). Thus, p38MAPK-mediated control of cell fusion not only connects individual cells but also appears to facilitate certain aspects of muscle differentiation associated with the formation of excitation-contraction coupling. In this symposium, we will discuss the mechanisms leading from myoblast cell fusion to muscle differentiation. In addition, we will discuss a recently developed optical p38 MAPK activity assay that enables us to understand how the kinase activity in living cells is dynamically regulated during muscle cell differentiation. This research advances our understanding of how cell fusion events, originating from membrane phospholipid regulation, is involved in muscle development and regeneration.

### [2S05m-4]

### Control of $Ca^{2+}$ signaling and excitation-contraction coupling in skeletal muscle: the phosphoinositide connection

*Vincent Jacquemond[1] ([1]*Institut NeuroMyoGène - CNRS UMR5261 - Inserm U1315 – Université Claude Bernard Lyon 1 – Lyon, France*)

Proper function of excitation-contraction (EC) coupling in muscle fibers relies on instant control of cytosolic $Ca^{2+}$ concentration by plasma membrane voltage. This is made possible by intimate molecular interactions between the voltage-dependent $Ca^{2+}$ channel $Ca_V1.1$ in the transverse invaginations of the plasma membrane (t-tubules), and the type 1 ryanodine receptor $Ca^{2+}$ release channel (RyR1) in the junctional sarcoplasmic reticulum (SR) membrane. On this basis, electrical activity sensed by $Ca_V1.1$ is mechanically transduced into opening of RyR1, allowing SR $Ca^{2+}$ release and binding of $Ca^{2+}$ to troponin C. We have found that phosphoinositides (PtdIns*P*s) have the capability to affect EC coupling function. First, depletion of PtdIns(4,5)$P_2$ in the t-tubule membrane, by activation of a voltage-sensitive phosphatase (VSP), dampens RyR1-mediated SR $Ca^{2+}$ release in a reversible manner. The exact mechanism is incompletely pinpointed but could involve both a role for t-tubule PtdIns(4,5)$P_2$ in easing $Ca_V1.1$ transducing function and/or in stabilization of the EC coupling machinery by promoting t-tubule anchorage of a junctional protein. More unexpected is the role for 3-OH phosphorylated forms of PtdIns*P*s. This was revealed by a mouse model of genetic deficiency in the PtdIns*P* phosphatase MTM1. MTM1 dephosphorylates PtdIns(3,5)*P*2 and PtdIns(3)*P* at the D3 position. MTM1 deficiency has dramatic consequences on EC coupling characterized by reduced amplitude of RyR1-mediated $Ca^{2+}$ release and progressive destruction of the t-tubule network. Of striking interest is the rescuing effect by a PtdIns-3-kinase inhibitor, which enhances SR $Ca^{2+}$ release in MTM1-deficient muscle fibers while having no effect on WT fibers. This suggests that accumulation of MTM1 PtdIns*P*s substrates is detrimental to $Ca^{2+}$ release. Beyond the related potential therapeutic interest, this opens the prospect that 3-OH phosphorylated PtdIns*P*s in the SR membrane directly modulate RyR1 function. Overall, we now have clear functional evidence for a role of PtdIns*P*s in the control of $Ca^{2+}$ signaling and EC coupling in muscle. One issue that remains uncertain, but of critical interest, is whether there are specific physiological conditions as well as other pathological conditions which change the endogenous levels of t-tubule or SR PtdIns*P*s so as to modulate EC coupling function.

### Symposium

[2S06m]

**Maintenance and disruption of homeostasis~Challenging Research for healthy life expectancy~**

March 29, 8:50 - 10:50, Room 6

### [2S06m-1]

### Physiological roles of free fatty acid receptors in maintaining tissue homeostasis

*Takako Ikeda[1,2], Ikuo Kimura[1,2,3] ([1]*Laboratory of Molecular Neurobiology, Graduate School of Biostudies, Kyoto University,* [2]*Department of Molecular Endocrinology, Graduate School of Pharmaceutical Sciences, Kyoto University,* [3]*Department of Applied Biological Chemistry, Graduate School of Agriculture, Tokyo University of Agriculture and Technology*)

The composition and diversity of gut microbiota affect host physiology through the production of bioactive metabolites. Short-chain fatty acids (SCFAs) are the main metabolites produced by microbial fermentation of dietary fiber. SCFAs are organic fatty acids with fewer than six carbon atoms, which are mainly composed of acetate, propionate, and butyrate. Many lines of evidence suggest that SCFAs play a crucial role in the regulation of physiological processes such as metabolic and immune system through G-protein-coupled receptors (GPCRs), termed free fatty acid receptors. GPR43 and GPR41were initially identified as SCFA-activated GPCRs. Signaling through these receptors affects various physiological processes, such as insulin secretion and anti-inflammatory responses, suggesting that SCFAs not only serve as an energy source but also act as signaling molecules. Following the discovery of GPR43 and GPR41, three additional GPCRs, namely GPR109A, Olfr78, and Olfr558 (also known as GPR164), have been identified as receptors for SCFAs, and their ligands and physiological importance have been studied. The beneficial effects of SCFAs on intestinal homeostasis have been investigated. SCFAs, especially butyrate, are important for the barrier function of intestinal epithelial cells. Butyrate repairs and enhances barrier function by increasing the expression of tight junction protein and inducing the redistribution of tight junction proteins. Butyrate also increases the expression of mucin 2, the most abundant mucin on the intestinal surface, thus conferring protection against luminal pathogens. In addition, SCFAs regulate appetite through promoting the release of glucagon-like peptide-1 (GLP-1) and peptide YY (PYY) from enteroendocrine L cells. GLP-1 and PYY stimulate intestinal vagal afferent nerve fibers to transmit signals to the central nervous system, thereby suppressing appetite. Therefore, SCFAs play an important role in transmitting appetite-suppressing signals through the gut–brain axis. Here, we present new insights and recent advances about a novel SCFA receptor, GPR164. GPR164 is olfactory chemosensory receptor that is expressed in many tissues other than the olfactory epithelium. GPR164 is coupled with $G_{olf}$ proteins in the olfactory epithelium, or $G_s$ proteins in other tissues, and transduce their signals by increasing intracellular cAMP levels. Interestingly, GPR164 was also identified as a medium-chain fatty acid (MCFA) receptor, suggesting that GPR164 can sense the nutrient status by switching ligands from a SCFA to a MCFA. A better understanding of physiological functions of GPR164 may provide insight into the mechanisms underlying the maintenance of intestinal homeostasis through diet.

### [2S06m-2]

### Osteoporosis: Pathophysiology and Drug Treatment, Changes in Bone Microarchitecture with Aging and Menopause

*Ko Chiba[1] ([1]*Department of Orthopedic Surgery, Nagasaki University Graduate School of Biomedical Sciences*)

Osteoporosis is a common disease in women, and the main causes are aging and menopause. After menopause, estrogen levels decline, which leads to activation of osteoclasts and increased bone resorption. Bone formation by osteoblasts cannot keep up, and bone mineral density (BMD) decreases.

BMD is usually measured using a device called DXA (dual-energy x-ray absorptiometry), but this is a two-dimensional evaluation that does not reveal fine changes within the bone. We are using a high-resolution CT device called HR-pQCT (High-resolution peripheral quantitative computed tomography) to study bone microarchitecture in osteoporosis. Our research has shown that the number of trabeculae decreases with age, and that cortical bone becomes porous and thins after menopause.

Medical treatment for osteoporosis typically includes two types of drugs: bone resorption inhibitors and bone formation promoters. Vitamin D supplements are often also used in conjunction with these drugs.

### [2S06m-3]

### Development of Antisense Drugs That Target Apolipoprotein C3 for Treatment of Familial Chylomicronemia Syndrome

*Mariko Harada-Shiba[1], Fumito Wada[2], Tadayuki Kobayashi[3] ([1]*Osaka Medical and Pharmaceutical University,* [2]*Liid Pharmaceuticals Inc,* [3]*National Cerebral and Cardiovascular Center Research Institute*)

Primary hyperchylomicronemia (PHC) is a rare and intractable disease characterized by marked accumulation of plasma chylomicrons. Recurrent acute pancreatitis is sometimes seen in these patients due to severely elevated levels of triglyceride (TG). PHC is caused by defects in the lipoprotein lipase (LPL) pathway due to genetic mutations, autoantibodies and so on. Apolipoprotein C3 (ApoC3) has been shown to be a good therapeutic target against PHC, and a clinical trial of anti-ApoC3 antisense in patients with PHC reported a decrease in TG levels. However, there have been reported some side effects including thrombocytopenia.
To develop a new drug targeting human ApoC3 for the treatment of PHC.
Methods: Antisense sequence was selected in vitro by the calcium enriched method which can estimate in vivo activity. We synthesized our original GalNAc-conjugated antisense which makes it possible to target hepatocytes. The in vivo effect was tested in the liver of humanized mice (mice with human hepatocytes). Thus, we conducted a dosage and administration study using cynomolgus monkeys.
After administration of GalNAc conjugated antisense into humanized mice, human ApoC3 mRNA was suppressed in the liver. The single dose study was conducted using the doses of 0.5, 1, 3, 20 mg/kg of GalNAc conjugated antisense which was administered subcutaneously into cynomolgus monkeys. A dose dependent decrease in plasma TG and ApoC3 in blood was shown which lasted for more than 40 days. After administration of 3 mg/kg of GalNAc conjugated antisense, the liver was obtained after 3, 28, 56 and 91 days and subjected to measure the amount of ApoC3 mRNA. The decrease in ApoC3 mRNA expression in the liver was observed until the day 56th. For the repeated-dose studies, administration of 0.3, and 1.0 mg/kg biweekly and 1.0 and 3.0 mg/kg monthly showed sustained suppression of plasma TG and ApoC3 levels in the blood. Pathological and biochemical studies showed no toxicity findings in any of the subjects. We are now conducting pre-clinical studies using mice, rats and cynomolgus monkeys to prepare for a first-in-human study.
Our original GalNAc conjugated antisense had efficacy in reducing plasma TG by reducing ApoC3 mRNA without any toxicity in non-human primates.

### [2S06m-4]

### The effect of combined endurance exercise training and hypoxia exposure on oxidative capacity in skeletal muscles

*Hisashi Takakura[1], Kojiro Ishii[1] ([1]*Doshisha University, Faculty of Health & Sports Scicence*)

Hypoxia training is generally used to enhance cellular and whole-body adaptations and/or performance. However, hypoxia as a supplement to training is not consistently of advantage for endurance at sea level. One of the reasons is that the effect of hypoxia training to muscle oxidative metabolism is still unclear. Based on the differences in physiological function and molecular mechanisms, muscle oxidative metabolism would be divided roughly into three factors of extracellular oxygen supply capacity, intracellular oxygen supply capacity and intracellular oxygen utilization capacity. Although usual endurance training increased the above three factors, the effective training requires the training program causing the notable positive response and adaptation to the above three factors without negative effect, compared with the usual endurance training. Therefore, this study aimed to examine whether combined endurance exercise training and hypoxia exposure enhance the muscle oxidative metabolism-affecting factors to comprehensively improve oxidative metabolism in skeletal muscles. First, examining the effects of hypoxic condition on oxygen supply capacity via hypoxia inducible factor-1α (HIF-1α), including red blood cell count and capillary density, showed that exposure to at least $<12\%$ oxygen for $>3$ h affects the kidney, while no hypoxic response was detected in muscles. Further, whether hypoxia exposure for 3 h after endurance training enhanced both oxygen delivery to the muscles and oxygen utilization capacity in skeletal muscles was examined. Combined hypoxia exposure and endurance training did not significantly change blood component, myoglobin, and mitochondrial volume in skeletal muscles. In conclusion, stimulation with hypoxia exposure or endurance training alone enhanced different factors involved in oxidative capacity. Meanwhile, the combination of hypoxia exposure and training may offset their individual effects, such as increased blood component and mitochondrial protein levels.

### [2S06m-5]

### Involvement of food texture in life-related diseases

*Yukari Date[1] ([1]*University of Miyazaki*)

The texture of food has been known to affect energy metabolism and/or life-related diseases. Although there are some reports that feeding with soft pellets or via a tube can increase body weight, it’s unclear how food texture differences influence energy metabolism. Here, we investigated the effects of two different food textures on energy balance and glucose and lipid metabolism in male Wistar rats. We fed the rats on soft pellets (SP) or control pellets (CP) on a 3-h restricted feeding schedule for 14 weeks and examined their energy intake, body weight, and energy expenditure. The levels of gastrointestinal hormones, glucose, and insulin were investigated at pre-, mid, and post-feeding. Glucose tolerance test, insulin tolerance test, the expressions of molecules involved in insulin signaling system or lipogenesis in the livers were also examined. Pancreatic islets were histologically investigated using anti-insulin and anti-Ki-67 antibodies. There were no significant differences in energy intake and body weight or gastrointestinal hormone levels between the SP and CP rats; however, the SP rats showed glucose intolerance and insulin resistance with disruption of insulin signaling. Increases in lipogenic factors were also found in the SP rats. The numbers of insulin-positive areas and Ki-67-positive cells of SP rats significantly increased. In addition, we also investigated whether changing SPs into CPs could improve the dysfunctions of glucose and lipid metabolism. There were no significant differences in the glucose metabolism and insulin resistance between CP rats and rats that were fed an SP for 11 weeks and changed it into a CP diet. These findings indicate that a soft food texture could be one of the causes for type 2 diabetes. In Japan, the body mass index (BMI) of diabetic patients is approximately 23 kg/m$^2$. The cut-off value of BMI for development of diabetes of Asian American is also known to be drastically low compared to that of Westerners. As shown in this study, a part of characteristics of SP-induced type 2 diabetes could resemble that of diabetes of Asian American as well as Japanese. Taken together, this study suggests that investigation into eating habit involved in a food texture and intervention in it may be efficacious in the prevention of the development of Asian diabetes.

### Symposium

### [2S08m]

**Breathing and emotion-cognition: New Insights from neuroscience and psychology**

March 29, 8:50 - 10:50, Room 8

### [2S08m-1]

### A crosstalk interaction between the brain and breath: Timing-dependent effects of respiration on functional brain networks and beyond

*Nozomu Nakamura[1] ([1]*Department of Physiology, Faculty of Medicine, Hyogo Medical University*)

Breathing, a naturally fundamental action that we cannot do without, sensitively and intensely changes under a variety of situations in our daily life. What if this essential act of breathing can impact our overall well-being? Recent studies showed that respiration couples with higher brain functions, such as perception, cognition, and behavior. We recently demonstrated that the timing of breathing (i.e., exhalation-to-inhalation transition) modulates performance accuracy in cognitive tasks and alters cortical activity (e.g., right supramarginal gyrus and temporoparietal junction), which is measured by human functional MRI (Nakamura et al., Cereb Cortex Commun, 3:tgac038, 2022). Moreover, our interventional approach using genetically modified mice with optogenetics showed that activation of the primary respiratory rhythm generator PreBötzinger complex (PreBötC) contributes to shaping hippocampal ensemble dynamics and memory performance (Nakamura et al., Nat Commun 14:4391, 2023). Here we have discussed i) how respiration interacts with brain oscillations, perception (and dyspnea), motor actions, and cognitive processes; ii) how respiratory rhythms are generated in pontomedullary networks of the brainstem; and iii) whether breathing manipulation is potentially applied for mental health care via the cingulo-opercular salience networks. Then, we hypothesize that PreBötC activity, which is equivalent to inspiratory onset or exhalation-to-inhalation transition, might trigger dynamics in convergence-divergence cycles of functional brain networks. These outlines and considerations of brain-breath interactions lead to a better understanding of the interoceptive and cognitive mechanisms that underlie brain-body interactions in health conditions and in stress-related and neuropsychiatric disorders.

### [2S08m-2]

### Development of a Biomarker for Mindfulness Trait Using Respiratory Variability: Attending to Natural Breathing Alters Respiratory Patterns

*Masahiro Fujino[1], Yuuki Ooishi[1], Takashi Sato[1], Yoshiyuki Ueda[2] ([1]*NTT Communication Science Laboratories,* [2]*Kyoto Univ.*)

Many studies of mindfulness-based therapy have shown that individuals having symptoms of depression or anxiety can reduce their symptoms by simply being aware of negative experiences, such as uncomfortable sensations and emotions, without diverting their attention from them, suppressing them, or reacting to them. However, some studies have reported that individuals with symptoms of depression or anxiety, while trying to be mindfully aware of their negative experiences, may unconsciously suppress them. This unconscious suppression results in adverse effects such as increased tension or discomfort. Objective physiological indicators are needed to measure the ability of individuals to be mindfully aware of their experiences, including sensations and emotions, as they are.
In this study, we got an idea from the phenomenon where directing attention to natural breathing can alter respiratory patterns. Based on this idea, we attempted to develop a physiological indicator using the difference in respiratory fluctuations between intentionally attending to natural breathing and not attending. Using a respiration transducer belt, non-meditation practitioners' respirations were measured both during the rest and intentionally attending to natural breathing. Furthermore, mindfulness traits were also measured using a questionnaire. In the analysis, we calculated the difference in respiratory fluctuations between the resting period and the period of attending natural breathing and examined the correlation between such a difference and mindfulness traits. The results showed that respiratory fluctuations were greater during the attending period than the resting period. Furthermore, a negative correlation was observed between this difference and the non-reactivity aspect of mindfulness traits. This suggests that the difference in respiratory fluctuations between rest and the period of attending natural breathing has a potential to be used as a physiological indicator of being aware of experiences as they are.

### [2S08m-3]

### Mind anchoring: Exploring the synergy between breathing rhythm, emotions, and memories

*Yuri Masaoka[1] ([1]*Department of Physiology, Showa University School of Medicine*)

The rhythm of breathing repeats even without our conscious awareness. The sense that accompanies this breathing is the sense of smell. By inhaling, we can perceive odors, and it can alter the state within the brain while linking to various emotions and memories. During times of anxiety or stress, the number of breaths increases, but consciously taking slow, deep breaths reduces anxiety. However, repeating conscious breathing can actually make us feel breathless. That's where olfactory stimulation, which can naturally alter our breathing, comes in. Pleasant olfactory stimulation naturally reduces the number of breaths, making them deeper without making us feel breathless. Functional magnetic resonance imaging (fMRI) showed that this deep breathing was positively correlated with the activity of the medial frontal cortex. The medial frontal cortex may have a role as a hub for the link between slow breathing and olfactory input to control our emotions emerging from the limbic areas. It is interesting to observe that odors associated with autobiographical memories (odor memory) showed the most increase in tidal volume. This increase in tidal volume positively correlated with the level of emotional arousal and the feeling of being brought back. fMRI indicated that, in addition to the frontal cortex, the occipital-parietal cortex was activated during the odor memory. The activity of the occipital-parietal cortex enhanced connectivity with the frontal cortex, especially the orbitofrontal cortex, as the feeling of nostalgia and recall increased. Spatial and temporal visual images emerge through odor memory, activating the occipital-parietal cortex even without actual visual input. Why do past memories, revived by odor memory, strengthen the connection between the occipital-parietal and frontal cortex? Possible mechanisms may be discussed in view of the relationship between the visual image of the memory and future imagery.

### [2S08m-4]

### The neural basis of successful communication: A hyperscanning fMRI study

*Takahiko Koike[1], Kanae Ogasawara[1] ([1]*RIKEN Center for Brain Science*)

The term "synchronized respiration with a partner (Iki-no-atta)" in Japanese describes high-quality coordinated movements, collaboration, and communication that involve precise synchronization. This phrase goes beyond a mere metaphor; recent studies in psychology and neuroscience research suggest that effective communication is not just about synchronized physical body movements but also involves the synchronization of physiological indicators, such as respiration, and brain activation. Traditional research has focused on individual metrics, such as respiration, heartbeat, and brain activity, as reflections of an individual's behavior, cognitive state, and perceptual state. However, recent studies shift the focus to the nature of communication involving two or more individuals: there is a reciprocal information exchange, and individuals mutually predict their partner’s behavior as an outcome of their own behavior. One of the noteworthy methods focusing on the reciprocity of communication is "hyperscanning," recording brain activity and/or physiological indicators simultaneously from two or more individuals during communication and calculating correlations, synchronization, or resonance of these indices between them. This approach assesses brain activity and physiological indicators not only as fluctuations within an individual but also examines their relationship to the partner. We have used a hyperscanning fMRI setting to simultaneously record brain activity from two individuals during communication, aiming to investigate the neural basis of effective communication. In this presentation, firstly, we explain the theoretical background of the hyperscanning method, including why it is essential to simultaneously record the brain activity and physiological indicators of two individuals to examine the neural basis of communication. Next, through the results of our hyperscanning studies, we will discuss what neural basis is behind successful communication.

### Symposium

[2S09m]

### From educational fields to cutting-edge research - the potential of small fish models

March 29, 8:50 - 10:50, Room 9

### [2S09m-1]

### Small fish for wide application from education to the scientific research

*Genri Kawahara[1], Yoshihiko Shimizu[2], Yukiko Hayashi[1] ([1]*Tokyo Medical University,* [2]*Kouryo Junior High School*)

Small fish species such as medaka and zebrafish are used in the latest research. They are used not only for basic research in neuroscience and developmental biology, but also for research into diseases and the development of drugs that can lead to cures. Meanwhile, small fish also make a significant contribution to early science learning in primary and secondary schools. Medaka are used for observation of eggs and embryos in fifth-grade classes at primary schools, while at junior high schools they are used as experimental animals in biology classes. In junior high schools, they are used to observe blood vessels, blood flow and blood cells, which are also used to learn about genetics. Medaka appear in their textbooks and in high school entrance examination and are widely used for learning early biology. Medaka used as teaching materials are generally purchased from pet shops, but our laboratory volunteers to provide them to a primary school and a junior high school.

On the other hand, our laboratory uses medaka and zebrafish for research on human diseases. For example, abnormal skeletal muscle phenotype is clearly detectable in muscular dystrophy models and they are useful to elucidate pathophysiology. Clear phenotypes in skeletal muscle are also useful for drug screening to search for therapeutic agents. We used the Duchenne muscular dystrophy (DMD) model fish, *sapje*, to identify drug candidates that improve the abnormal skeletal muscle structure found in their skeletal muscle.

We believe that small fishes are excellent animal models for human diseases, and they are useful in providing an opportunity to experience basic science and in contributing to the development of science.

### [2S09m-2]

### Exploratory activities using fish

*kyuichi kanda[1] ([1]*Takatsuki high school*)

### [2S09m-3]

### Functional evolution of skeletal muscle revealed by zebrafish

*Fumihito Ono[1] ([1]*Osaka Medical and Pharmaceutical University*)

Acetylcholine receptors (AChRs) functioning in neuromuscular junctions are composed of five subunits. Among the subunits, gamma, the embryonic type, switches to epsilon, the adult type, as the animal develops. We previously proposed that zebrafish expresses AChRs lacking both epsilon and gamma subunits, which are specific to slow muscle fibers. We recently confirmed it by showing that a classical non-depolarizing muscle relaxant pancuronium inhibited fast muscle fibers at lower concentration compared to slow muscle fibers in zebrafish, which matched the difference arising from the different composition of AChRs recorded in Xenopus oocytes. We named the two types of AChRs in zebrafish as e/g(-)-and e/g(+)-type, based on the absence or inclusion of epsilon/gamma subunits. The former and the latter was specific to slow and fast muscles, respectively. These two types had contrasting channel characteristics, with regards to the calcium conductance, inward rectification and open time duration. Interestingly, protochordate *ciona intestinalis* before metamorphosis had AChRs in the neuromuscular junctions with characteristics compatible with the e/g(-)-type in zebrafish. Moreover, muscle fibers in *ciona* and those in zebrafish slow muscles had other common features such as the absence of sodium-channel-dependent action potentials and the lack to cell fusion. Phylogenetic analysis of subunit genes also support that AChRs in *ciona* are e/g(-)-type and epsilon/gammasubunits evolved later.In contrast to epsilon/gamma double knockout (KO) zebrafish in which slow muscles function normally, epsilon/gamma double knockout mice are totally paralyzed, suggesting that skeletal muscles in mammals contain mostly e/g(+)-type AChRs.Based on these findings, we propose that AChRs in chordates are divided into two types: e/g(-) and e/g(+). Primitive chordates had the former type. Teleost including zebrafish developed the latter, which is restricted to the fast muscle. Mammalian skeletal muscles are e/g(+)-type, phylogenetically derived from fast muscles of zebrafish.

### [2S09m-4]

### Pathophysiological role of liver extracellular ATP/ADO in MALFD/MASH using zebrafish model

*Reiko Hanada[1] ([1]*Oita University*)

Excessive energy intake accumulates in adipocytes and nonadipose tissue, including the liver. As fat accumulation in the liver progresses metabolic dysfunction associated with fatty liver disease (MAFLD) develops and, in some cases, progresses to irreversible metabolic dysfunction-associated steatohepatitis (MASH), which can lead to cirrhosis and liver cancer. The pathogenesis of MASH is thought to be based on a complex process involving oxidative stress, inflammation, and other factors. However, the detailed pathogenesis and molecular pathophysiological mechanisms of MAFLD/MASH have not been fully elucidated, and no effective treatment has yet been established.We have been investigating the pathophysiology of MAFLD/MASH using mouse models. In general, rodent models of MAFLD/MASH were less likely to develop liver fibrosis and required approximately one year of high-fat dietary treatment to develop MASH, which also limited their usefulness as models. Against this background, we currently focused on zebrafish as an alternative *in vivo* disease model to mice. Zebrafish possess approximately 70% of the orthologs of human genes and are a helpful model as a medical experimental animal due to their various other advantages. Furthermore, recent advances in gene modification technology have made zebrafish use in medical research is spreading rapidly.Hepatocytes damaged by fat accumulation release ATP extracellularly, together with adenosine (ADO) converted from ATP, as damage-associated molecular patterns (DAMPs) that promote further inflammation and fibrosis of the hepatocytes. Thus, the importance of ATP/ADP dynamics in the liver in the pathogenesis of MAFLD/MASH has been suggested. However, the correlation between ATP and ADO kinetics over time and disease progression in MAFLD/MASH, its pathophysiological significance, and its relationship with liver fibrosis and prognosis have yet to be investigated. Therefore, taking advantage of zebrafish, we have established sensor zebrafish ($GRAB_{ATP}$ and $GRAB_{ADO}$ fish) that can visualize ATP and ADO dynamics as fluorescent GFP intensity in the liver *in vivo* and are investigating the pathological progression of MAFLD/MASH related to ATP and ADO dynamics. In this talk, I will introduce our recent progress in elucidating the pathophysiological role of liver extracellular ATP/ADP dynamics in MAFLD/MASH using a zebrafish model.

### [2S09m-5]

### Discovery of novel regulatory mechanisms using an obesity and diabetes model zebrafish

*Yasuhito Shimada[1] ([1]*Mie University*)

Obesity is a condition where excessive fat accumulates in the body, and a Body Mass Index (BMI) of 30 or more (25 or more in Japan) is defined as obesity. According to a WHO report, about 35% of the population in the US and about 25% in European countries are obese. This paves the way for 'life-threatening' diseases that follow, such as type 2 diabetes, hypertension, dyslipidemia leading to arteriosclerosis, coronary artery disease, and it makes them a high-risk group for strokes. While excessive energy intake, genetics, and lack of exercise are the main causes of obesity, the therapeutic effects of dietary restrictions and exercise are limited, indicating a significant scope for improvement from a preventive medicine perspective.

In 2011, we developed the first dietary-induced obesity model of zebrafish (Oka T, *et al*. *BMC Physiol*. 2010; 10: 21) and have since conducted research on obesity and its related diseases using this model for over a decade. Throughout our studies, we uncovered novel onset mechanisms for fatty liver and dyslipidemia (Shimada Y, *et al*. *Nutr Metab*. 2015; 12: 17), and identified MXD3 as a critical molecule for visceral fat accumulation (Shimada Y, *et al*. *Int J Obes*. 2013; 38: 1053-1060). In 2017, we developed the first type 2 diabetes mellitus model of zebrafish (Zang L, *et al*. *Sci Rep*. 2017; 7: 1461) and identified the novel insulin regulatory gene, CENPX (Zang L, *et al*. F*ront Genet*. 2019; 10: 693). We are now working on creating a diabetic nephropathy model by introducing an igf1 receptor deficiency and EGFP labeling of urinary proteins. We will share our ongoing research into potential therapeutic genes for nephropathy, a condition currently incurable in clinical. Additionally, we will discuss our preliminary steps towards future drug development, which includes using a visceral fat visualization model, conducting screening studies, and transitioning from zebrafish to mouse models—eventually aiming for human clinical trials.

### Symposium

### [2S10m]
### Physiological and behaviorological approach for psychiatric phenotypes

March 29, 8:50 - 10:50, Room 10

### [2S10m-1]

### An approach for pathophysiology of mental disorders from neural circuit mechanisms of decision-making behavior

*Takatoshi Hikida[1] ([1]*Osaka University Institute for Protein Research*)

Decision-making behavior is important for animals to survive, and impairment in decision-making can be observed in various mental disorders. Nucleus accumbens (NAc) is one of key neural substrates for decision-making behavior in the cortico-basal ganglia circuit. Within the NAc, dopamine D1 and D2 receptor-expressing medium spiny neuron (D1-/D2-MSNs) in the direct and indirect pathways have been revealed to play important roles in controlling reward and aversive behavior, respectively. However, the collaborative role of NAc D1-/D2-MSNs in decision-making behavior has been remained. In this study, using a visual discrimination task in mice, we assessed the role of NAc D1-/D2-MSNs in cue-guided reward-based decision-making behavior. Cell-type specific neuronal silencing and in-vivo calcium imaging revealed NAc D1-/D2-MSNs to separately contribute to cue-guided reward-based decision-making behavior. Our findings indicate that neural circuit mechanisms within NAc underlies decision-making behavior and the pathophysiology of mental disorders.

### [2S10m-2]

### Filial attachment behavior in mammals: focus on primates' distress vocalizations

*Kumi O Kuroda[1,3], Saori Yano-Nashimoto[2], Takuma Kurachi[4], Kazutaka Shinozuka[3], Nami Ohmura-Tagane[5], Chihiro Yoshihara-Nemoto[1] ([1]*Tokyo Institute of Technology,* [2]*Hokkaido University,* [3]*RIKEN,* [4]*Tokyo University of Agriculture and Technology,* [5]*Hiroshima University*)

For mammalian offspring, the relationship with the parent (primary caregiver) is the sole lifeline that sustains survival. Therefore, the offspring remembers its parents, follows them, and contributes to the maintenance of the parental relationship by sending various signals. These behaviors are collectively called attachment behaviors. Among them, crying (distress vocalization) is one of the most potent reinforcing signals of the parental approach. We found that when parents carry their offspring, infants reduce their crying, voluntary movement and heart rate within a few seconds in human infants and mouse pups. The transport response is regarded as a primitive attachment behavior, as it facilitates the parental transport of the offspring. In family-living New World monkeys common marmosets, infants' vocalization has a vital function in soliciting the turn changes of carrying among family members. Infants not only modulate the amount of distress vocalizations, but also tune the quality of distress vocalizations, depending on the parenting style of each caregiver and their social context within the family. Such a flexible attachment system develops in the average-expectable rearing environment of this species; if the infants are separated from their family in early infancy, they cannot develop adaptive use of attachment behaviors both physically and vocally. Even infant crying has a vital function for infant survival among mammals, a significant proportion (20-30%) of human infants are known to exhibit unsoothable crying without obvious reasons. Excessive crying may stress parents and may induce impulsive maltreatment by the caregiver. To prevent such incidents, recent technological advances such as wearable sensors in combination with machine learning may be used to help parents. We would like to discuss these crying-related issues in this symposium.

### [2S10m-3]

### Repeated neural activations induce long-term structural plasticity of the nucleus

*Tsuyoshi Miyakawa[1] ([1]*Fujita Health University*)

Activation of neurons triggers plastic changes in neural circuits that are essential for brain development, learning and memory, but overactivation can lead to pathological alterations. Here, we show that repeated neural activation induces long-term changes in the nuclear structure of neurons, persisting for two weeks. These alterations involve disruption of nuclear lamina and an increase in heterochromatin domains and are accompanied by changes of transcriptome and chromatin accessibility, which partially resemble the $G_2$-M phase in cycling cells, and hyper-locomotor activity in mice. These cell cycle-like changes and hyper-locomotor activity were mitigated by *in vivo* gene knockout of cyclin B, a molecule essential for $G_2$-M phase transition. Our results demonstrate that subchronic neural activation reinstates a cell cycle-like process, leading to chronic changes in neuronal functions and behavior. Our findings provide novel insights into activity-dependent plastic changes in neuronal circuits, which may be relevant to the pathogenesis of neuropsychiatric disorders.

### [2S10m-4]

### Altered circadian rhythm of activity and heart rate variability in depression

*Hiroshi Kunugi[1,2] ([1]*Teikyo University,* [2]*National Center of Neurology and Psychiatry*)

Reduced activity and sleep-wake rhythm disturbances are essential features of depressive disorders. Although diagnosis of depressive disorder is currently made based on subjective symptoms of patients, these disturbances could be measured objectively by wearable devices such as actigraphy (accelerometer). Using wrist-worn actigraphy, we previously reported that fragmented sleep and altered circadian rest-activity rhythms in depressed patients (Hori et al, 2016; PMID: 26978182). Heart rate variability (HRV), another physiological measure that can be monitored by a wearable sensor on subtle changes in duration of successive heartbeats, has also been known to provide clinically useful information on autonomic nervous system functions. Accumulating evidence has suggested reduced HRV indices in patients with mood disorders. Surprisingly, there was no study that examined sleep-wake activity rhythm and HRV simultaneously. By using a wearable sensor on chest that monitors both 3-dimensional acceleration and HRV, we examined the activity and HRV indices in depressive episode of mood disorders for 3 consecutive days (Koga et al, 2022; PMID: 35906793). We found that activity magnitude was significantly reduced while lying/resting time was increased in depressed patients, compared with controls. HRV indices such as R-R interval and high-frequency power (a parameter for the parasympathetic system) were significantly decreased in patients than in controls. Importantly, sympathetic load during sleep significantly correlated with damped rest-activity rhythm in depressed patients, which might be a characteristic pathology of depression. The observed sympathetic overload in depressed patients could be modified through the gut-brain interaction, i.e., probiotics produce short chain fatty acids that stimulate the vagus nerve and mitigate the autonomic imbalance. This possibility accords with our observations that dysbiosis (reduced *Bifidobacterium*) was associated with depression and their poorer clinical outcomes (Aizawa et al, 2016; PMID: 27288567; Otaka et al, 2021; PMID: 34068832).

### Symposium

[2S03a]
Cooperation with Other Societies Committee
**Next Generation Researches Toward the Understanding of Dynamic Cellular Signaling**

March 29, 14:20 - 16:20, Room 3

### [2S03a-1]

### Neuronal-activity-dependent activation of ROS signal and its involvement in regulation of cerebellar functions

*Sho Kakizawa[1] ([1]*Grad. Sch. Pharmaceu. Sci., Kyoto University*)

Reactive oxygen species (ROS) is a redox-signaling molecules, and indicated to be involved in various pathophysiological events in organisms, such as aging and lifestyle-related diseases. In addition, recent studies suggest possible involvement of ROS in physiological events. For example, expression of ROS synthases (NADPH oxidase (Nox) and dual oxidase (Duox)) in various tissues are reported. These facts indicate that ROS has dual functions, physiological and pathophysiological functions, in organisms. However, physiological functions of endogenous ROS are yet to be determined, especially in brain systems.
Plasticity at cerebellar synapses are cellular basis for cerebellar-dependent motor learning. In parallel fiber (PF) to Purkinje cell synapse in the cerebellum, long-term depression (LTD) is identified as the cellular basis for acquisition of motor learning such as optokinetic response (OKR) and eyeblink conditioning. So far, many signaling molecules are indicated to be involved in the induction of cerebellar LTD. However, most of the molecules are very rapidly catalyzed or excluded, and molecular mechanisms underlying long-term memory are largely unknown. We focused on the persistent effects of ROS on other molecules through chemical modification, such as disulfidation.
In this symposium, I will introduce our recent studies indicating involvement of ROS and its downstream signal, 8-nitro-cGMP, in induction of cerebellar LTD and OKR. In addition, imaging study demonstrating production of ROS by neuronal activity will be introduced. These observations suggest that ROS is a physiological messenger produced by neuronal activity and involved in higher brain functions.

### [2S03a-2]

### Identification of mitochondrial K+ conductance that paradoxically benefits ATP synthesis

*Junji Suzuki[1,3,4], Kirichok Yuriy[2,3,4] ([1]*Equator Therapeutics Japan, G.K.*, [2]*Washington University in St. Louis*, [3]*University of California San Francisco*, [4]*Equator Therapeutics, Inc.*)

The chemiosmotic theory postulated that the inner mitochondrial membrane (IMM) is impermeable to $K^+$. This abundant cytosolic cation can be attracted into the organelle by the extremely negative voltage, causing uncontrolled mitochondrial swelling, depolarization, and disruption of ATP production. However, the IMM has since been proposed to have various mechanisms of $K^+$ permeability, ranging from a slow $K^+$ uniport to large-conductance $K^+$ channels. To resolve this controversy, we explored the $K^+$ permeability of the IMM using whole-IMM patch-clamp electrophysiology. Here we demonstrate that the IMM has a small $K^+$ conductance mediated by a previously unidentified uniporter for monovalent cations (UMC). UMC is almost equally permeable to $K^+$, $Na^+$ and $Cs^+$. Its unitary currents cannot be resolved and are compatible with either a very low-conductance channel or a transporter. UMC is inhibited by a wide variety of ion channel inhibitors and modulators. At the intact-mitochondria level, these inhibitors demonstrate that slow mitochondrial $K^+$ uptake via UMC does not cause measurable mitochondrial membrane potential depolarization. However, from these experiments, UMC emerges as crucial for regulation of mitochondrial volume, phosphate import, ATP production and respiration capacity. In conclusion, the IMM contains a $K^+$-permeable uniporter that paradoxically benefits oxidative phosphorylation and was an overlooked factor in the original formulation of the chemiosmotic theory.

### [2S03a-3]

### Lighting up extracellular signaling dynamics using new genetically-encoded fluorescent indicators

*Daisuke Ino[1] ([1]*Department of Pharmacology, Graduate School of Medicine, Osaka University*)

Hundreds of extracellular signaling molecules are supposed to facilitate intercellular communications in the brain. Nevertheless, the precise mechanisms by which these molecules operate within the living brain remain largely elusive, primarily attributed to the absence of techniques capable of capturing their spatiotemporal dynamics. Therefore, to address this issue, we have been developing genetically-encoded fluorescent sensors, designed to yield optical readouts upon binding with their designated ligands. In this presentation, we will introduce the new fluorescent sensors we have engineered and their applications to a variety of biological phenomena.

### [2S03a-4]

### In vivo intracellular calcium imaging analyses in peripheral organs and tissues to elucidate their physiological functions

*Kazunori Kanemaru[1] ([1]*Department of Physiology, Nihon University School of Medicine*)

Intracellular $Ca^{2+}$ signaling reflects ongoing cellular activity, since it is activated by a variety of extracellular bioactive substances including hormones and neurotransmitters, which in turn triggers cellular processes including secretion, contraction, and transcription. Most insights of spatiotemporal pattern and function of $Ca^{2+}$ dynamics have predominantly obtained by ex vivo experiments such as cultured cells and extracted organs. The ex vivo preparations lose physiological environments such as local circulations, hormones, nutrients, and neurotransmitters. Therefore, in vivo validation of these findings using is crucial to clarify physiological/pathophysiological function of $Ca^{2+}$ signals. However, many peripheral organs and tissues are still awaitied verification due to technical limitations. Compared to the central nervous system, where optical devices can be easily fixed on the skull, in vivo $Ca^{2+}$ imaging analysis of peripheral organs and tissues is more challenging, because of the difficulty in fixation of imaging optics and motion blur caused by heartbeat, respiration, and skeletal/smooth muscle contractions. To solve these problems, we generated transgenic mouse lines expressing yellow cameleon-Nano50 (YC-Nano50), a highly sensitive ratiometric $Ca^{2+}$ indicator protein, in the cells of peripheral organs and tissues. Using these mice, we have succeeded to analyze $Ca^{2+}$ dynamics in vivo. In this presentation, we will introduce the in vivo $Ca^{2+}$ dynamics of pancreatic β-cells, liver hepatocytes, and taste cells in tastebuds, and their unexpected controlling mechanisms and physiological roles.

### [2S03a-5]

### Functional analysis of $Ca^{2+}$ signaling in skeletal muscles from malignant hyperthermia model mice

*Toshiko Yamazawa[1] ([1]*Core Research Facilities, The Jikei University School of Medicine*)

Skeletal muscle contracts when depolarization is transmitted to the dihydropyridine receptor in the T-tubule, releasing $Ca^{2+}$ through the type 1 ryanodine receptor (RYR1) from the sarcoplasmic reticulum (SR). Mutations in RYR1 cause severe muscle diseases, such as malignant hyperthermia (MH), characterized by a disorder of $Ca^{2+}$-induced $Ca^{2+}$ release (CICR) through RYR1 from the SR. We recently reported that volatile anesthetics (e.g., isoflurane) induce MH-like episodes by enhancing CICR in heterozygous R2509C-RYR1 mice. We simultaneously measured intracellular $Ca^{2+}$ concentration ($[Ca^{2+}]_{cyt}$) and cellular temperature, finding that an increase in cellular temperature is associated with an increase in $[Ca^{2+}]_{cyt}$ upon the application of isoflurane. Furthermore, our progress extended to the successful expression of a genetically encoded $Ca^{2+}$ indicator within skeletal muscles. This method enabled us to monitor temperature changes and $[Ca^{2+}]_{cyt}$ alterations in real-time within living mice under MH conditions.

### Symposium

[2S04a]
### The cross-talk between physiology and exercise science

March 29, 14:20 - 16:20, Room 4

### [2S04a-1]
### Role of sensory ion channels in skeletal muscle afferents during exercise

*Rie Ishizawa[1] ([1]*National Institute of Fitness and Sports in KANOYA*)

The exercise pressor reflex (EPR) is an important neural mechanism that originates in working skeletal muscle that contributes to the control of cardiovascular function during physical activity. The EPR is mediated by the muscle mechanoreflex and metaboreflex which is predominantly activated via mechanically and metabolically sensitive group III and group IV muscle afferents during muscle contraction. Both group III and IV muscle afferent fibers synapse in the dorsal horn of the spinal cord and subsequently project to the brainstem (cardiovascular control area). Then, BP is elevated by increasing sympathetic nerve activity (SNA). Several ion channels in sensory afferents innervating skeletal muscle have been suggested to contribute to the EPR function. Transient receptor potential cation channel subfamily V member 1 (TRPV1) was expressed in sensory afferent fibers innervating skeletal muscle, and a recent study using a novel TRPV1 null mouse model to directly study the EPR demonstrated that the metaboreflex is mediated by activation of TRPV1 in skeletal muscle afferents. However, the mechano-gated channels responsible for mechanosensation in skeletal muscle afferents remain to be elucidated. Piezo channels are known to be mechanically-activated ion channels that play crucial roles in several mechanotransduction processes in various organs. We examined the impact of Yoda1, a specific Piezo channel activator, on neuronal responses in rat mechanosensitive group IV afferents innervating skeletal muscle using a muscle-nerve preparation. As a result, Piezo1 expressed and functioned in mechanosensitive-group IV fibers innervating skeletal muscle. These findings demonstrate an important role for Piezo1 and TRPV1 in the mechanoreflex and metaboreflex components of the EPR.

### [2S04a-2]
### Circulatory physiology and exercise science

*Shigehiko Ogoh[1] ([1]*Toyo University*)

There is not much research available regarding the interaction between the two physiological functions of cerebral circulation and respiratory regulation, particularly in the context of their influence during exercise. We investigated the interplay between these two systems from the perspective of cerebral circulation regulation during physical activity. In this symposium, we categorize the physiological factors of cerebral circulation regulation function into both direct and indirect categories and provide an overview of the correlation between these two physiological systems in relation to the interplay between the cerebral circulation regulation system and the respiratory regulation system. We also explain how cerebral circulation function is affected by alterations in the respiratory system induced by exercise. These discussions could potentially provide insights for clinical applications, such as understanding the mechanisms behind the increased risk of brain-related diseases in patients with respiratory disorders like chronic obstructive pulmonary disease (COPD) and identifying clues for optimizing exercise rehabilitation.

### [2S04a-3]
### Prospects of glucose and lactate metabolism in exercise science

*Takeshi Hashimoto[1] ([1]*Faculty of Sport and Health Science, Ritsumeikan University*)

All our living cells require glucose as a pivotal energy substrate. During exercise, contractile skeletal muscle activates glycolysis, which can anaerobically produce ATP, leading to lactate and pyruvate formation, that are the main fuels for oxidative metabolism. Given that lactate/pyruvate ratio increases many times during muscle contraction, lactate is considered as a major energy substrate in exercise (Hashimoto and Brooks, 2008).

In addition to the energy substrate, it is well worth to focus on an aspect of signaling molecule in the lactate metabolism. Actually, we found that it functions as an inducer of cell signaling in skeletal muscle cells (Hashimoto et al., *FASEB J* 2007) and adipocytes (Hashimoto et al., *J Appl Physiol* 2013), and elicits muscle hypertrophy in rats (Oishi et al., *J Appl Physiol* 2015).Additionally, we found that high-intensity interval exercise (HIIE), which increases lactate production, is beneficial for the improvement of cognitive executive function (Tsukamoto et al., *Physiol & Behav* 2016a; *MSSE* 2017). Further we found that HIIE-induced lactate might be a key factor for the improvement of cognitive executive function (Tsukamoto et al., *Physiol & Behav* 2016b; Hashimoto et al., *FASEB J* 2018).

In the sports and health science, we can utilize beneficial effects of lactate acutely and chronically (Hashimoto et al., *Metabolites* 2021). Here, I will try to uncover importance of glycolysis-induced lactate metabolism in the field of exercise physiology.

### [2S04a-4]

### Exercise and the Aging Brain: Findings from Masters Athletes and Randomized Controlled Trials

*Takashi Tarumi[1] ([1]*National Institute of Advanced Industrial Science and Technology*)

Physical activity and exercise demand cognition and stimulate its neural substrates through interactions with the environment. Visuosensory information from the surroundings, increased attention to compete and outperform opponents, verbal communications with teammates, and/or physiological responses (e.g., increased cerebral blood flow) to increased metabolic rate in athletic sports are all integrated to stimulate the brain during exercise. Having performed for years or a lifetime, exercise training may elicit significant brain adaptations through interactions with aging. In older adults, exercise is used as one of the therapeutic strategies to prevent cognitive impairment and dementia (e.g., Alzheimer's disease). Nevertheless, current evidence is inconsistent as to the preventive effects of exercise training on age-related cognitive decline and impairment, as revealed by the recent meta-analysis and systematic reviews. Our research group investigated whether aerobic exercise training would alter age-related reduction in neurocognitive function using cross-sectional and interventional designs. We observed that middle-aged and young athletes committed to long-term, high-intensity endurance training have significant alterations in regional cortical thickness and microstructural organization of the white matter fiber tracts, particularly the corpus callosum when compared with the sedentary control subjects. The cerebrovascular assessments further revealed increased regional cerebral perfusion and alterations in the dynamic pressure-flow relation in endurance athletes compared with sedentary adults. Conversely, one-year aerobic exercise showed small effects on neurocognitive function in cognitively normal older adults and patients with mild cognitive impairment. This session will summarize the key findings from the presenter's previous studies investigating the association between aerobic exercise and neurocognitive function in aging adults.

### Symposium

[2S05a]
### Multi-layered research to analysis of the molecular pathology of psychiatric disorders

March 29, 14:20 - 16:20, Room 5

### [2S05a-1]
### Serotonin - the neural mechanisms of optimism and pessimism

*Kayoko Miyazaki[1] ([1]*Okinawa Institute of Science and Technology*)

Waiting for future rewards is an adaptive behavior based on the anticipation of future rewards. We have previously reported the following results from studies in rats and mice that demonstrate a causal relationship between serotonergic neural activity in the dorsal raphe nucleus (DRN) and future reward anticipation behavior. (1) Serotonergic neurons in rats showed a sustained increase in activity during reward-waiting behavior and a decrease when waiting was abandoned (Miyazaki et al., J Neurosci 2011). (2) Local pharmacological inhibition of serotonergic neuronal activity in the DRN impaired the patience of rats to wait for delayed rewards (Miyazaki et al., J Neurosci 2012). (3) Optogenetic activation of serotonergic neurons in the DRN improved the patience of mice to wait for both conditioned reinforcer tone and food reward (Miyazaki et al., Curr Biol 2014). (4) Serotonin stimulation promoted waiting most effectively when the probability of reward delivery is high but the timing of delivery is uncertain (Miyazaki et al., Nat Commun 2018). (5) When the delay time is constant and the timing of reward delivery is predictable (low temporal uncertainty), waiting is only promoted by serotonin stimulation in the orbitofrontal cortex (OFC). On the other hand, when the timing of rewards is difficult to predict (high temporal uncertainty), serotonin stimulation not only in the OFC but also in the medial prefrontal cortex (mPFC) promotes waiting (Miyazaki et al., Sci Adv 2020). Furthermore, a recent fiber photometry study shows that dorsal raphe serotonin neurons encode probability, not value, of future rewards, which can support risk-sensitive behaviors. These findings suggest that the serotonergic system acts on higher brain regions to produce flexible adaptive behaviors in response to changes in the temporal uncertainty of future rewards.

### [2S05a-2]
### Animal models of Mendelian diseases that accompany mood disorders

*Kazuo Nakajima[1,2,3], Tadafumi Kato[2,3] ([1]*Department of Physiology, Teikyo University School of Medicine,* [2]*Laboratory for Molecular Dynamics of Mental Disorders, RIKEN Center for Brain Science,* [3]*Department of Psychiatry & Behavioral Science Juntendo University Graduate School of Medicine*)

Regarding the biological study of mood disorders such as depression and bipolar disorder, where causative genetic mutations have not been identified, it would be an effective approach to focus on Mendelian diseases that accompany mood disorders. In those Mendelian diseases, there are two possibilities: one is that mood disorders are caused by pleiotropic effects derived from causal genetic mutations, and the other is that other genetic mutation linked to causal genetic mutations becomes a risk factor for mood disorders. In the former case, we have focused on Darier disease, which causes skin keratosis, and the responsible gene is *ATP2A2* (or *SERCA2*), which encodes a $Ca^{2+}$ pump on the endoplasmic reticulum membrane and is important for intracellular $Ca^{2+}$ signaling. We analyzed *Atp2a2* heterozygous brain-specific conditional knockout (hetero cKO) mice. ATP2A2 was shown to be essential for neuronal $Ca^{2+}$ homeostasis, and the cKO mice showed behavioral phenotypes including abnormalities in novel environments and impaired fear memory. Enhanced dopamine neurotransmission was also detected in the nucleus accumbens. These findings may explain how *ATP2A2* mutation confers risk for mood disorders.
In the latter case on Mendelian diseases that accompany mood disorders, we have focused on a family of autosomal dominant tubulointerstitial kidney disease (ADTKD) comorbid with mood disorders. The causal renal gene for ADTKD was identified as *MUC1*, but it was not expressed in the brain. By linkage analysis and whole-genome analysis of this family, a E492K mutation in the NTRK1 gene was identified, that was approximately 1 Mb distal to *MUC1*. *NTRK1* codes for TrkA (Tropomyosin-related kinase A) which is a receptor for nerve growth factor and essential for development of the cholinergic neurons. We generated conditional knock-in mice carrying this mutation specifically in the brain. The mice showed depression-like behavior in response to physostigmine, which activates cholinergic signaling. While there were no detectable morphological abnormalities of cholinergic neurons in the mice, basal pERK levels were increased in the hippocampus, suggesting that the E492K mutation might cause altered downstream signaling of TrkA. These results imply that the *NTRK1* E492K mutation impairs cholinergic neurotransmission, and may convey susceptibility to mood disorders.

### [2S05a-3]
### Cellular phenotypes and causal candidate genes in iPSC-derived neurons of bipolar disorder family patients

*Gakuya Takamatsu[1,2], Yoko Manome[4], Kanako Toyama[1,9], Kumiko Yanagi[5], Dimitar Dimitrov[7], JunSeok Lee[1,9], Yuko Akamine[1], Kae Koganebuchi[3,6], Minami Hasegawa[4], Tomoko Hayakawa[1,8], Fuyuko Yoshida[10], Kotaro Hattori[10,11], Tomoyuki Takahashi[7], Hiroshi Kunugi[10,12], Tsuyoshi Kondo[2], Tadashi Kaname[5], Hirotaka Okano[4], Ryosuke Kimura[3], Masayuki Matsushita[1] ([1]*Dept. of Mol. Cel. Physiol., Grad. Sch. of Med., Univ. of the Ryukyus,* [2]*Dept. of Neuropsy., Grad. Sch. of Med., Univ. of the Ryukyus, Okinawa, Japan,* [3]*Dept. of Hum. Biol. Anat., Grad. Sch. of Med., Univ. of the Ryukyus, Okinawa, Japan,* [4]*Div. of Regen. Med., Jikei Univ. Sch. of Med., Tokyo, Japan,* [5]*Dept. of Gen. Med., Natl. Ctr. for Chd. Hlth. Dev., Tokyo, Japan,* [6]*Dept. of Biol. Sci., Grad. Sch. of Sci., Univ. of Tokyo, Tokyo, Japan,* [7]*Cel. Mol. Syn. Func. Unit, Okinawa Inst. of Sci. Tech. Grad. Univ., Okinawa, Japan,* [8]*Dept. of Phamc., Jichi Med. Univ., Tochigi, Japan,* [9]*Adv.Med. Res. Ctr., Fac. of Med, Univ. of the Ryukyus, Okinawa, Japan,* [10]*Dept. of Men. Dis. Res., Nat. Inst. of NeuSci., Natl. Ctr. of Neu. and Psy.,* [11]*Dept. of Biores., Med. Gen. Ctr., Natl. Ctr. of Neu. and Psy.,* [12]*Dept. of Psy., Teikyo Univ. Sch. of Med.*)

Patient-derived induced pluripotent stem cells (iPSCs) have the potential to be cellular models for neuropsychiatric diseases. Bipolar disorder (BD) is a major psychiatric disorder characterized by mood and activity dysregulation ranging from depression to mania.
BD is inheritable with an estimated heritability of 70–80%; however, genomic variants that strongly contribute to BD are almost unknown, and its molecular pathophysiology is uncovered because of the high clinical and genetical heterogeneity as well as the polygenicity. To overcome the limitation of heterogeneity in the study of pathogenesis of BD, we focused on rare familial patients and performed cellular phenotype analysis using patient iPSC-derived neurons.
First, we surveyed pedigrees with multiple BD patients in Okinawa Prefecture. We conducted a parametric linkage analysis on a three-generation pedigree with multiple patients with BD and recurrent depressive disorder. We detected a significant linkage peak in a chromosome 1p region which previous studies repeatedly reported positive links to bipolar disorder and depressive disorder. Subsequently, we determined the entire sequence of the 6.7 Mb haplotype segregating with all affected family members using whole genome sequencing. The three-generation familial patients might share the high-penetrance variants.
We generated iPSC lines from affected and unaffected individuals of the family. Notably, excitatory neurons directly reprogrammed from the affected members' iPSCs showed a significantly higher calcium transient frequency than neurons from healthy controls in Fluo4-AM calcium imaging.
Finally, to discover potential disease-causing genes, we performed RNA sequencing of patient-derived neurons and analyzed allelic imbalances of transcripts in the 1p-linkage haplotype. We found a reduced expression of a transcriptional variant of a nuclear-encoded mitochondrial gene in affected members of the family. It might contribute to mitochondrial dysfunction and the development of the disease in the family.
In conclusion, we observed that neurons derived from the familial patients exhibit a potential cellular phenotype. The patient-derived iPSCs would be valuable resources to elucidate the pathogenesis of BD. (COI: properly declared.)

### [2S05a-4]

### Generation and analysis of *MECP2* mutant marmosets

*Noriyuki Kishi[1,2], Junko Okahara[1,3], Kenya Sato[3], Daisuke Yoshimaru[1,4], Yoko Kurotaki[3], Kohei Onishi[1], Tsukasa Sanosaka[2], Rachel Henry[5], Taeko Ito[1], Misako Okuno[1], Edward Weinstein[5], Jill Crittenden[6], Hirotaka Okano[1,4], Jun-ichi Hata[1,2,7], Jun Kohyama[2], Erika Sasaki[1,3], Tomomi Shimogori[1], Hideyuki Okano[1,2] (*[1]RIKEN Center for Brain Science, [2]Keio University School of Medicine, [3]Central Institute for Experimental Animals, [4]Jikei University School of Medicine, [5]Horizon Discovery, [6]Massachusetts Institute of Technology, [7]Tokyo Metropolitan University*)

Rett syndrome (RTT) is a severe, progressive neurodevelopmental disorder that primarily affects girls. Its prevalence rate is one in 10,000-15,000. Children with RTT develop relatively normally for 6-18 months, after which they experience rapid regression, with loss of purposeful hand use, deceleration of head growth, and autistic behaviors. Mutations of the *MECP2* gene on the X chromosome are found in over 95% of cases of classic Rett syndrome. While the functions of *MECP2* in the central nervous system have been studied using *Mecp2* mutant mice, it is important to note that these mouse models do not always accurately replicate the symptoms observed in RTT patients due to the differences in brain structure and function between primates and rodents. The establishment of nonhuman primate (NHP) models that are similar to humans in many aspects is very important to understand the pathogenesis of RTT. The common marmoset (*Callithrix jacchus*) is a small New World primate that is native to the Atlantic coastal forests in northeastern Brazil. It offers several advantages as a model organism. In this study, we used genome editing to generate *MECP2* mutant marmosets and establish a new primate model for the neurodevelopmental disorder RTT. The marmosets lacking *MECP2* were born without complications, but during the weaning period, they exhibited impaired brain growth and reduced activity. MRI analysis in this model reveal diminished cortico-cortical connections, particularly among those originating from the prefrontal cortex. By conducting single-nucleus and spatial transcriptome analyses, we identified abnormal gene expression related to neuronal maturation in *MECP2*-deficient neurons, varying across different types of neurons and cortical layers. These findings demonstrate that the marmoset model faithfully mirrors the pathophysiology observed in RTT patients and has potential for advancing our understanding of the underlying mechanisms of RTT and for conducting preclinical research.

### Symposium

[2S06a]
**HFpEF -The Cold Case in Heart Failure Pathophysiology-**

March 29, 14:20 - 16:20, Room 6

### [2S06a-1]

### Cardiovascular Mechanics in HFpEF -From basic knowledge to a decision support system-

*Keita Saku[1] ([1]*National Cerabral and Cardiovasucular Center*)

The rapidly increasing number of heart failure patients, also known as the heart failure pandemic, is a critical issue that directly affects the sustainability of regional healthcare systems and medical costs. In an aging society, heart failure with preserved ejection fraction (HFpEF) is becoming increasingly common. The aging population is more likely to have other medical conditions that increase the risk of HFpEF, such as hypertension, diabetes, and chronic kidney disease. Additionally, ventricular hypertrophy, diastolic dysfunction, atherosclerosis, and loss of elasticity in the arteries become apparent with age. The autonomic nervous system, which strongly regulates cardiovascular function, is also known to decline with age. It is extremely difficult for Japan's 15,000 cardiologists to manage the enormous number of both outpatient and hospitalized heart failure patients. Additionally, the workload of non-specialists at all stages of care and the cost of inviting specialists, especially in underpopulated areas, are major problems. To address these issues, we are developing a cardiovascular bio-digital twin, a physiology-based simulator, that supports early diagnosis and optimal intervention for heart failure. In addition, we are also developing a D-to-D system that could aid regional heart failure care link. In this session, we will review the alteration of cardiovascular function in HFpEF. We will also introduce our ongoing development of a heart failure management system based on the knowledge of cardiovascular physiology.

### [2S06a-2]

### Exploration of therapies for HFpEF targeting age-related fibrotic protein

*Ippei Shimizu[1] ([1]*National Cerebral and Cardiovascular Center Research Institute*)

Therapy for heart failure with preserved ejection fraction (HFpEF) is extremely limited, and it is urgent to establish new therapies for this disorder. Recently, we identified a new senescence-associated secretory phenotype (SASP) factor. Testing more than 600 human samples, we found a secreted-type pro-fibrotic protein increased in circulation with age (Hereby described as "age-related fibrotic protein (AFP)"). This molecule also increased in the plasma of chronologically aged or dietary obese mice. We also found AFP increased in the circulation of patients with HFpEF, atrial fibrillation, sarcopenia, chronic kidney disease(CKD) and non-alcoholic steatohepatitis (NASH). The global population of patients suffering from these diseases is considered as follows; HFpEF(13million), NASH(3-5%), CKD(10-12%), and now we are trying to categorize them as "Age-related fibrotic disorders (A-FiD)". Studies with systemic as well as tissue-specific knockout models, and tissue-specific overexpression models indicated AFP would become a therapy for A-FiD including HFpEF, and now we are trying to generate a neutralizing antibody for AFP.

### [2S06a-3]

### Unraveling the pathogenesis of HFpEF through clonal hematopoiesis.

*Yoshimitsu Yura[1] ([1]*Nagoya University School of Medicine, Department of Cardiovascular Medicine*)

Clonal hematopoiesis, resulting from an array of nonmalignant driver gene mutations, can lead to altered immune cell function and chronic disease and has been associated with worse outcomes in patients with heart failure (HF) with reduced ejection fraction (HFrEF). However, the role of Clonal hematopoiesis in the prognosis of heart failure with preserved ejection fraction (HFpEF) has been understudied. To investigate the causal role of Clonal hematopoiesis in HFpEF, non-conditioned mice underwent adoptive transfer with *Tet2*-wildtype or *Tet2*-deficient bone marrow. They were subsequently subjected to a high-fat diet/L-NAME combination treatment to induce features of HFpEF. This model of *Tet2*-Clonal hematopoiesis exacerbated cardiac hypertrophy by heart weight to tibia length, diastolic dysfunction by E/e' and LV EDP, and cardiac fibrosis compared to the *Tet2*-wildtype condition. Furthermore, we characterize Clonal hematopoiesis in patients with HFpEF**.** Using a panel of 20 candidate Clonal hematopoiesis driver genes and a variant allele frequency cutoff of 0.5%, ultra-deep error-corrected sequencing identified Clonal hematopoiesis in a cohort of 81 patients with HFpEF (Age: 71 ± 6 years old, EF: 63% ± 5%) and 36 control individuals without a diagnosis of HFpEF (Age: 74 years old ±7 years old, EF: 61.5% ± 8%). Compared to control individuals, patients with HFpEF exhibited an increased prevalence of mutations in the Clonal hematopoiesis driver genes *TET2* alone (12% vs. 0%, respectively, p=0.02). Within the HFpEF cohort, patients with CH exhibited exacerbated diastolic dysfunction in terms of E/e' (14.9 vs. 11.7, respectively, p=0.0096) and E/A (1.69 vs. 0.86, respectively, p=0.0134) compared to those without Clonal hematopoiesis. Accordingly, HFpEF patients with Clonal hematopoiesis and an age ≥ 70 years old exhibited worse prognosis in terms of 5-year CV-related hospitalization rate (HR = 5.023, p= 0.0041) compared to HFpEF patients without Clonal hematopoiesis and an age ≥ 70 years old. Clonal hematopoiesis is associated with worse heart function and prognosis in patients with HFpEF, and a murine experimental model of *Tet2*-mediated Clonal hematopoiesis displays greater features of HFpEF.

### [2S06a-4]

### Mechanisms of diastolic dysfunction and HFpEF in diabetic cardiomyopathy

*Satomi Adachi-Akahane[1] ([1]*Toho Univ.*)

Since heart failure has a high incidence rate among the elderly and a very poor prognosis, the number of patients with heart failure is expected to explode in the future. Therefore, developing methods for diagnosing, treating, and preventing heart failure is an urgent issue. One of the risk factors for HFpEF is diabetes mellitus. Diabetic cardiomyopathy is known to present with left ventricular diastolic dysfunction prior to systolic dysfunction. The mechanisms of diabetes-induced left ventricular diastolic dysfunction include structural remodeling such as hypertrophy of ventricular myocytes and increased cardiac fibrosis, as well as glucotoxicity and lipotoxicity caused by metabolic abnormalities, mainly due to dysregulation of glucose-metabolizing hormones, that give rise to disruption of mitochondria function, intracellular calcium signal regulation, and sarcomere protein function in ventricular myocytes, resulting in diastolic dysfunction. In addition, the insulin receptor signaling pathway has been shown to play an important role in regulating intracellular calcium signaling in ventricular myocytes, and it has become clear that inadequate insulin action is also involved in diastolic dysfunction in the early stages of diabetic cardiomyopathy. On the other hand, ventricular contractile and diastolic function has turned out to be maintained by the compensatory action of intrinsic cardioprotective factors, suggesting that HFpEF develops as a result of a balance between exacerbating and protective factors. In this symposium, I will review and present the latest findings from basic research on the molecular mechanisms of left ventricular diastolic dysfunction in the early onset of diabetic cardiomyopathy and its progression to the advanced stage. I would like to further extend the discussion to challenges in elucidating the pathological mechanisms and novel therapeutic approaches.

### [2S06a-5]

### HEpEF Update -A Multifaceted Consequence in The Heart

*Yasuko K Bando[1] ([1]*Molecular Physiology and Cardiovascular Biology, Mie University Graduate School of Medicine*)

The pathophysiology of heart failure with preserved ejection fraction, HFpEF, is still halfway, presumably because HFpEF is a multifaceted consequence in the myocardium that progresses in a subclinical and asymptomatic fashion. More concretely, the risk factors for HFpEF, such as hypertension, aging, and diabetes, are often associated with chronic inflammation, leading to a “cardio-sclerotic” or “aging-of-heart” condition that causes hemodynamic and pathological abnormalities, namely diastolic left ventricular dysfunction with cardiac remodeling such as fibrosis and microvasculopathy. Importantly, recent clinical trials revealed that SGLT2 inhibitor is the first effective drug for ameliorating worsening heart failure in HFpEF via an unknown mechanism. To explore the mechanism underlying the pathophysiology of HFpEF, including the impact of SGLT2 inhibitor, simultaneous and multiple approaches using molecular and physiological research are essential and inseparable as a pair of wheels. I would like to overview the recent progress in the pathophysiology of HFpEF from the topic of left atrial myopathy to the comprehensive screening using single cell RNA sequence, to expecting a breakthrough in this unresolved problem in basic and clinical research.

### Symposium

### [2S08a]
### Cardiovascular Functions Regulated by the Sophisticated Structures and Interactions of Ion Channels

March 29, 14:20 - 16:20, Room 8

### [2S08a-1]
### Dynamic remodeling of TRPC5-caveolin-1-eNOS complexes regulates the precise signal transfer between $Ca^{2+}$ and NO

*Reiko Sakaguchi[1,2,3], Nobuaki Takahashi[2], Takashi Yoshida[4], Nozomi Ogawa[2], Yoshifumi Ueda[2], Satoshi Hamano[2], Kaori Yamaguchi[5], Seishiro Sawamura[2], Shinichiro Yamamoto[4], Yuji Hara[6], Tomoya Kawamoto[2], Akito Nakao[2], Masayuki X Mori[1], Shunichi Shimizu[4], Ryuji Inoue[7], Yasuo Mori[2] (*[1]School of Medicine, University of Occupational and Environmental Health, [2]Graduate School of Engineering, Kyoto University, [3]Institute for Integrated Cell-Material Sciences, Kyoto University, [4]Faculty of Pharmaceutical Sciences, Teikyo Heisei University, [5]Hall of Global Environmental Studies, Kyoto University, [6]School of Pharmaceutical Sciences, University of Shizuoka, [7]Department of Physiology, Fukuoka University*)

The cell signaling molecules nitric oxide (NO) and $Ca^{2+}$ regulate diverse biological processes through their closely coordinated activities directed by signaling protein complexes. However, it remains unclear how dynamically the multi-component protein assemblies behave within the signaling complexes upon the interplay between NO and $Ca^{2+}$ signals. Here we demonstrate that TRPC5 channels activated by stimulation of G-protein-coupled ATP receptors mediate $Ca^{2+}$ influx, which triggers NO production from endothelial NO synthase (eNOS), inducing secondary activation of TRPC5 *via* cysteine *S*-nitrosylation and eNOS in vascular endothelial cells. Mutations in the caveolin-1-binding domains of TRPC5 disrupt its association with caveolin-1 and impair $Ca^{2+}$ influx and NO production, suggesting that caveolin-1 serves primarily as the scaffold for TRPC5 and eNOS to assemble into the signal complex. Interestingly, during ATP receptor activation, eNOS dissociated from caveolin-1 in turn directly associates with TRPC5, which accumulates at the plasma membrane dependently on $Ca^{2+}$ influx and calmodulin (CaM). This protein reassembly likely results in a relief of eNOS from the inhibitory action of caveolin-1 and an enhanced TRPC5 *S*-nitrosylation by eNOS localized in the proximity, facilitating the secondary activation of $Ca^{2+}$ influx and NO production. In isolated rat aorta, vasodilation induced by acetylcholine was significantly suppressed by the TRPC5 inhibitor AC1903. Thus, our study provides evidence that dynamic remodeling of the protein assemblies among TRPC5, eNOS, caveolin, and CaM determines the ensemble of $Ca^{2+}$ mobilization and NO production in vascular endothelial cells.

### [2S08a-2]
### The role of TRPC6 channel in peripheral arterial disease

*Yuri Kato[1], Tsukasa Shimauchi[1,2], Takuro Tomita[2], Kazuhiro Nishiyama[1], Akiyuki Nishimura[2], Motohiro Nishida[1,2] (*[1]Kyushu Univ., [2]NIPS*)

Peripheral arterial disease (PAD) represents a condition in which peripheral blood vessels become occluded due to obstruction of blood flow caused by arteriosclerosis progression. After occlusion, angiogenesis, and vessel maturation are promoted to restore blood flow to the tissues. We have previously reported that activation of Transient receptor potential canonical (TRPC) 6, a receptor-activated cation channel on the plasma membrane, negatively regulates myogenesis of vascular smooth muscle cells (VSMC), which is required for vascular maturation after ischemia. In this study, we aimed to determine the involvement of TRPC6 channels in peripheral vascular injury in vivo.
The left femoral artery was ligated to induce a hind-limb ischemia as a mouse model of PAD. Vessel diameter and vessel maturation after hind-limb ischemia were clarified by immunohistochemical analysis for *a*-SMA and CD31, and peripheral blood flow was measured using a laser doppler flowmetry, which was accelerated blood flow recovery in TRPC6-deficient mice compared to wild-type mice. TRPC6 KO/ACTA2-C6 (WT) mice, which express TRPC6 specifically in VSMCs of TRPC6-deficient mice, were generated and treated with ischemia, TRPC6 KO/ACTA2-C6 (WT) mice showed significantly delay blood flow recovery after ischemia compared to TRPC6-deficient mice. Furthermore, TRPC6 inhibitor were screened for using vascular maturation, and 1-BP (1 -Benzyl-1 (11-hydroxyundecyl) piperidin-1 -ium chloride) was identified. Intraperitoneal implantation of 1-BP with an osmotic pump in WT mice enhanced blood flow recovery and increased the number of matured blood vessels after hind-limb ischemia.
These results suggest that inhibition of TRPC6 channel activity in VSMCs improves blood flow recovery after hind-limb ischemia in mice and that inhibition of TRPC6 channel activity may be a novel therapeutic target for peripheral arterial disease.

### [2S08a-3]
### Molecular identification of a new auxiliary subunit for the voltage-gated $K^+$ channel KCNQ1 expressed in hearts

*Go KASUYA[1], Koichi NAKAJO[1] (*[1]Division of Integrative Physiology, Department of Physiology Jichi Medical University School of Medicine*)

A certain number of ion channels form macromolecular complexes with their auxiliary subunits. Auxiliary subunits directly interact with ion channels, modulating various properties such as expression, localization, and activation/inactivation kinetics, thus diversifying the physiological functions of ion channels. KCNE1, also called MinK, is a single-transmembrane protein that was first identified in 1988 as a putative pore-forming channel subunit. A few years later, it was revealed that KCNE1 serves as an auxiliary subunit for the voltage-gated $K^+$ channel KCNQ1, forming the slowly activating $K^+$ currents ($I_{Ks}$), which plays a crucial role in action potential repolarization in humans. KCNE1 belongs to the KCNE protein family, comprising five isoforms (KCNE1-5), all capable of modulating KCNQ1 channel properties. However, since the discovery of KCNE5 in 2001, no new KCNE genes have been reported. Here, we identified a KCNE-like gene in the zebrafish genome, which we refer to as KCNE6 for clarity. Electrophysiological experiments revealed that, while the amino acid sequence of the zebrafish KCNE6 has the highest similarity to KCNE3 among the five KCNE isoforms, the modulational effect on KCNQ1 by the zebrafish KCNE6 resembles that of mammalian KCNE1 rather than KCNE3. Comparative sequence analysis across various species indicated that the genomic sequences around the *kcne6* open reading frame regions are conserved in vertebrates, ranging from fish to mammals. Co-expression of KCNE6 from various vertebrates with KCNQ1 showed that KCNE6 from lower vertebrates, including marsupials, can modulate the KCNQ1 currents, like the zebrafish KCNE6. Additionally, promoter analysis using a transgenic zebrafish line confirmed KCNE6 expression in zebrafish hearts. In summary, our study has led the identification of KCNE6, a new KCNE isoform, marking the first discovery in over two decades.

### [2S08a-4]

### Pathophysiological roles of cardiac KCNQ1 channels

*Junko Kurokawa[1], Masami Kodama[1], Satoshi Shimizu[1], Shushi Nagamori[2], Yasuhide Watanabe[1], Kazuho Sakamoto[1] ([1]*Faculty of Pharmaceutical Sciences, University of Shizuoka*, [2]*The Jikei University School of Medicine*)

The $I_{Ks}$ channel, a cardiac potassium channel, consists of the alpha subunit KCNQ1 and the beta subunit KCNE1, contributing to the repolarization phase of the cardiac action potential. Mutations in these genes are associated with the development of lethal arrhythmias due to congenital QT prolongation syndrome and are influenced by sympathetic nerve stimulation and sex hormones. We have demonstrated the involvement of a molecular complex of the KCNQ1 channel in the $I_{Ks}$ regulation by intracellular $Ca^{2+}$, cAMP, and NO. Our proteomic analysis reveals that the KCNQ1 molecular complex participates in $Ca^{2+}$ signaling, epithelial junction signaling, mitochondrial dysfunction, and more. However, we have yet to elucidate its specific pathophysiological role. Hence, our goal is to examine whether cardiac $I_{Ks}$ channels activated by pathological $Ca^{2+}$ overload could counterbalance arrhythmias using transgenic mice ($I_{Ks}$-Tg) expressing human $I_{Ks}$ channels. Using a sepsis model (Cecal Slurry injection), we induced excess $Ca^{2+}$. $I_{Ks}$-Tg mice showed significantly lower sepsis scores than wild-type mice. In this presentation, I would like to discuss the molecular mechanisms of sepsis-induced cardiac dysfunction development based on patch clamp data from isolated cardiomyocytes.

### [2S08a-5]

### Elaborate gating mechanism of cardiac ryanodine receptor

*Haruo Ogawa[1] ([1]*Graduate School of Pharmaceutical Sciences, Kyoto University*)

Ryanodine receptor 2 (RyR2) is a large $Ca^{2+}$ channel localized in the sarcoplasmic reticulum of the myocardium and plays a central role in muscle contraction. RyR2 mutations (>300 locations) are associated with several arrhythmogenic heart diseases, such as catecholaminergic polymorphic ventricular tachycardia (CPVT). The primary triggering for RyR2 opening is an elevation of cytoplasmic $Ca^{2+}$ concentration. When $Ca^{2+}$ binds to RyR2, the channel opens, which is known as $Ca^{2+}$-induced $Ca^{2+}$ release (CICR). However, the molecular mechanism by which a molecule as small as $Ca^{2+}$regulates the opening of RyR2, a supramolecular 60,000 times larger than $Ca^{2+}$, and the structural basis of disease-related mutations that cause abnormal channel activity have long been unclear. Recently, we have determined the cryo-EM structures of RyR2. By combining a series of functional analyses, we succeeded in resolving the gating mechanism of RyR2 by $Ca^{2+}$-binding. We also clarified the mechanism by which disease-related mutations cause channel abnormalities. In this symposium, we will present the RyR2 gating mechanism that we have elucidated so far and our recent results.

### Symposium

[2S09a]

Cooperation with Other Societies Committee

**Newly identified regulatory mechanisms to preserve the homeostasis and induction of diseases caused by their disruptions**

March 29, 14:20 - 16:20, Room 9

### [2S09a-1]

### Involvement of cellular senescence and enhanced growth signals in pulmonary hypertension

*Lin Hai Kurahara[1], Gaopeng Li[1], Xiaodong Li[1], Kaori Ishikawa[2], Ryo Ishikawa[3], Kazufumi Nakamura[4], Katsuya Hirano[1] ([1]*Department of Cardiovascular Physiology, School of medicine, Kagawa University,* [2]*Department of General Internal Medicine, Kagawa University Hospital,* [3]*Department of Diagnostic Pathology, Kagawa University Hospital,* [4]*Department of Cardiovascular Medicine, Faculty of Medicine, Okayama University*)

Pulmonary hypertension (PH) is characterized by pulmonary arterial remodeling or remodeling of pulmonary artery, attributable to enhanced proliferation of pulmonary arterial smooth mouse cells and endothelium dysfunction. Proliferating cells as well as senescent cells contain shorter telomere, the telomere length is maintained by the RNA-dependent DNA polymerase activity of telomerase reverse transcriptase (TERT), a catalytic subunit of telomerase complex. TERT also exhibits RNA-dependent RNA polymerase (RdRP) activity, which lease to abnormal cell proliferation. RdRP activity is increased by phosphorylation of T249 by cyclin-dependent kinase 1 (CDK1). However, the role of telomere length, TERT expression and cell senescence in pathogenesis of PH remains elusive. The patients with PH have more cells with shortened telomeres in the intra-vascular area. The CDK1/TERT-double positive cells increased in the extra-vascular area of pulmonary hypertension. The enhanced proliferation of pulmonary smooth muscle cells isolated from PH patients was significantly ameliorated by CDK1 inhibitor, Ro-3306. Most of the TERT-positive cells seen around pulmonary artery in PH were positive for CD44, a cancer stem cell marker. The cells positive for p16, a senescence marker, were observed mainly in the pulmonary arterial endothelium. The p16-positive smooth muscle cells were extensively found in the lung of PH associated with systemic scleroderma. Cordycepin, 3'-deoxyadenosine derived from *Cordyceps militaris*, is known to exert anti-RdRP activity and anti-senescence activity. Cordycepin significantly improved remodeling of pulmonary artery and right ventricles and prolonged the survival of PH rats. Taken together, accumulated senescence endothelial cells and proliferative smooth muscle cell are suggested to be a key biological process in vascular remodeling of pulmonary hypertension. These results invite consideration of the potential impact on PH of strategies aimed at controlling cell senescence and CDK1/TERT axis.

### [2S09a-2]

### Pathogenesis-based therapeutic approaches toward diabetic peripheral neuropathy

*Kazunori Sango[1], Hideji Yako[1], Mari Suzuki[1], Naoko Niimi[1], Shizuka Takaku[1] ([1]*Diabetic Neuropathy Project, Tokyo Metropolitan Institute of Medical Science*)

Diabetic peripheral neuropathy (DPN) is one of the most common chronic complications of *Diabetes Mellitus* (DM), and its prevalence correlates closely with the degree and duration of hyperglycemia caused by pancreatic ß cell dysfunction and impaired insulin actions. Although the detailed pathogenesis of DPN remains largely unknown, metabolic disorders of neurons, glial cells, and vascular endothelial cells could be deeply involved in progressive neural dysfunction and irreversible nerve fiber damage. Streptozotocin (STZ), a compound that has a preferential toxicity against pancreatic β cells, has been utilized to induce type 1 DM in rodents, and the STZ-diabetic mice exhibit DPN that partially mimics human disorders (hypoalgesia, reduced nerve conduction velocities, etc.). In addition, *Drosophila melanogaster* is a suitable animal model for genetic analyses and is also useful for the study of DM because the organs and molecular regulators of energy metabolism in *Drosophila* are mostly analogous to those in humans. Adult flies fed a high-sugar diet (HSD) develop human type 2 DM-like phenotypes, such as hyperglycemia and insulin resistance. However, whether dietary-induced diabetic conditions induce peripheral nerve disorders in the flies remains unknown. In this presentation, we introduce cutting-edge approaches toward DPN in close association with the disruption of metabolic regulatory mechanisms in STZ-diabetic mice and HSD-fed flies. In particular, a role of exogenous pyruvate in the maintenance of glycolysis-TCA cycle under diabetic conditions, and glial proteasome as one of the potential therapeutic targets for DPN will be discussed.

### [2S09a-3]

### Mechanisms of the Postnatal Neutrophil Surge and its Potential Roles in the Establishment of Postnatal Homeostasis.

*Ryo Ishiwata[1], Yuji Morimoto[1] ([1]*Dept. of Physiology, National Defense Medical College*)

Mammalian neonates experience an abrupt surge of blood neutrophil counts during the first 24 hours of life. The neutrophil surge is thought to be an adaptive response of neonates to the acute transition from a bacteria-free uterine environment to a bacteria-rich environment, but the mechanisms and the specific roles of the surge are unclear. In this study, we aimed to elucidate where the surge arises from and where the neutrophils are destined for.Full-term (embryonic day 21, e21) Wistar rat fetuses were delivered vaginally or by caesarean section. Flow cytometric analysis showed that the blood neutrophil count increased from 484 ± 70.5/mL at e21 to 1,109 ± 80.8/mL at 6h after birth ($n$ = 8, $P$ < 0.001), while the monocyte and lymphocyte counts did not change during this period. The proportion and the maturity of the bone marrow neutrophils did not change from e21 to 6h. Instead, we observed a significant increase in the BrdU$^+$ proliferating cells in the bone marrow from day 1 to day 3 of birth, suggesting that an activation of bone marrow haematopoiesis occurs after the neutrophil surge. Immunohistochemistry for myeloperoxidase showed that the spleen and liver of e21 fetuses contained a significant proportion of neutrophils. We then performed flow cytometric analysis of a whole organ. The number of neutrophils per liver decreased from e21 to day 1 ($1.05\times10^7 \pm 5.09\times10^5$ vs. $0.85\times10^7 \pm 5.03\times10^5$, $n$ = 8, $P$ < 0.05), while the number of neutrophils per spleen did not change significantly ($4.7\times10^5 \pm 7.23\times10^4$ vs. $7.4\times10^5 \pm 1.26\times10^5$, $n$ = 8, $P$ = 0.09). Hepatic neutrophils had higher expression of integrin α4 and β1 and lower expression of CXCR2 than blood neutrophils, indicating that the hepatic neutrophils are a less mature population. Administration of the NOS inhibitor, L-NAME, significantly inhibited the neutrophil surge in rats at 6h of birth and tended to maintain hepatic neutrophil counts. We also observed *Lys-EGFP* mice, in which EGFP is selectively expressed in neutrophils. Observation of *Lys-EGFP* neonates delivered by caesarean section using IVIS (*in vivo* imaging system) showed that EGFP signal intensity in the liver decreased over time up to 12 hours of birth, supporting the liver-origin of the neutrophil surge. Instead, the signal intensity increased siginificantly in the oral tissue. In oral lavage fluid, neutrophils were transiently detected at the time of the neutrophil surge. These results suggest that the neutrophil surge results from the release of hepatic neutrophils in NOS-dependent manner and the the the surge may contribute to the establishment of the postnatal oral homeostasis.

### [2S09a-4]

### Roles of Epac1 in the regulation of contractility and myosin regulatory light-chain phosphorylation in cardiac muscle

*Yoshiki Ohnuki[1], Kenji Suita[1], Okumura Satoshi[1] ([1]*Dept. of Physiol., Tsurumi Univ. Sch. of Dent. Med.*)

The second messenger, cyclic AMP (cAMP), is one of the most important signaling molecules responsible for the effects of β-adrenergic receptor (β-AR) stimulation on cardiac function. The major intracellular functions of cAMP are mediated not only by protein kinase A (PKA) but also by the more recently identified exchange protein directly activated by cAMP (Epac). However, in contrast to PKA, a well-described cAMP effector, the involvement of Epac in the contractility of cardiac myofilaments has not been established. To elucidate the contribution of Epac1 to cardiac myofilament response to β-AR stimulation, we examined the contractility as well as the phosphorylation status in skinned (demembranated) myocardium prepared from transgenic mice overexpressing Epac1 in the heart (Epac1TG). $Ca^{2+}$ sensitivities of force and ATPase activity as well as tension cost (ATPase activity/force) were significantly increased in cardiac myofilaments from Epac1TG, compared to non-transgenic mice (NTG). Phosphorylation level of myosin regulatory light chain (RLC) was significantly greater in Epac1TG than that in NTG without any changes in the phosphorylation level of other myofilament proteins, troponin I (TnI) and myosin binding protein C (MyBP-C). We also observed that pharmacological activation of Epac with 8-CPT-AM, an Epac-specific but not isoform-selective cAMP analogue, increased $Ca^{2+}$ sensitivities of force and ATPase activity as well as phosphorylation levels of RLC in skinned myocardium prepared from wild-type mice. In addition, the pharmacological activation of Epac increased phosphorylation level of myosin phosphatase target subunit (MYPT), a negative regulator of myosin light chain phosphatase (PP1cδ). However, the increase in the phosphorylation levels of RLC and MYPT was blunted by the addition of a phospholipase C (PLC) inhibitor (U73122) or a protein kinase C (PKC) inhibitor (Bisindolylmaleimide I). These results suggest that Epac1 activation by β-AR stimulation promotes RLC phosphorylation and subsequent increases in $Ca^{2+}$ sensitivity and tension cost in cardiac myofilaments through PLC/PKC/MYPT signaling pathways, independently of PKA.

### Symposium

### [2S10a]
### Understanding the inter-functional linkage between membrane lipid molecules and ion channels

March 29, 14:20 - 16:20, Room 10

### [2S10a-1]
### A switching mechanism between $PIP_2$-and voltage-dependence in two pore channels

*Takushi Shimomura[1,2], Yoshihiro Kubo[1,2] ([1]*National Institute for Physiological Sciences*, [2]*The Graduate University for Advanced Studies*)

Two-pore channels (TPCs) are a family of voltage-gated cation channels critical for $Ca^{2+}$ release from endosomes and lysosomes. They are composed of two pairs of homologous repeats, each with six transmembrane helices corresponding to the functional units of the superfamily of voltage-gated cation channels. The former four helices consist of voltage sensor domains and the latter two form pore domains in each domain. Domain I (DI) has a $PIP_2$-binding site and DII is responsible for voltage sensing. There are three types of TPCs that differ in $PIP_2$ and voltage sensitivity. It is still unclear how the TPC subtypes exhibit different sensitivities to the two stimuli.

We performed an electrophysiological analysis of TPCs in the *Xenopus laevis* oocyte expression system using a two-electrode voltage-clamp technique. We investigated whether two TPC subtypes, TPC3, which is primarily voltage-gated, and TPC2, which is $PIP_2$-gated and non-voltage-gated, share the underlying mechanisms for sensing the two stimuli. In particular, we applied voltage clamp fluorometry (VCF) to TPC3 to investigate the detailed dynamics of the S4 helix in DII (DII-S4), a core part of voltage sensing. VCF analysis of DII-S4 in TPC3 clearly showed that it has an intermediate state that opens in a strongly $PIP_2$-dependent manner, along with up and down conformations. Mutations that stabilize this intermediate state changed TPC3 from predominantly voltage-gated to strongly $PIP_2$-gated. In TPC2, the tricyclic antidepressant desipramine induces DII-S4-based voltage dependence. Mutational stabilization of the intermediate state corresponding to TPC3 impaired voltage-induced currents in TPC2 but did not affect $PIP_2$-induced currents. These results indicate a unique regulatory mechanism common to TPC subtypes, the ability to switch between $PIP_2$-gating and voltage-gating modes. Namely, in addition to a down ("resting") and an up ("activated") conformations that reflect its voltage-dependent movement of DII-S4, DII-S4 adopt an intermediate conformation, in which TPCs are more sensitive to $PIP_2$. This new mechanism in TPCs may provide a perspective for understanding voltage-gated ion channels in general.

### [2S10a-2]
### Voltage-sensing Phosphatase (VSP) Activity in Mammalian Epididymal Maturating Spermatozoa

*Takafumi Kawai[1], Yasushi Okamura[1] ([1]*Graduate School of Medicine, Osaka University*)

The voltage-sensing phosphatase (VSP) was first identified in sea squirt and shows voltage-dependent phosphoinositide phosphatase activity. We previously reported that VSP is functionally expressed in mammalian spermatozoa, regulating the spermatozoa motility. However, there is no detailed information about the voltage-sensing capability of mammalian VSP even in heterologous expression system. In the present study, we first detected the voltage-sensing phosphatase activity of mouse VSP (mVSP) in vitro by modifying its N-terminal and intracellular loop with their functional domains remaining intact. We examined the voltage-sensitivity of mVSP with $PI(4,5)P_2$ sensitive GIRK current as well as $PI(4,5)P_2$ probe. The threshold of voltage-sensitivity in mVSP is similar or even lower than that of other species; it starts to show the activity around -30mV. We also examined the time course of VSP activation in native spermatozoa by focusing on the sperm maturation process. Interestingly, we found that $PI(4)P/PI(4,5)P_2$ ratio, which is increased by VSP activity, gradually increases during the maturation process in normal spermatozoa, but not in VSP-deficient spermatozoa, indicating that VSP is constitutively activated during the maturation process. Then, based on the above-mentioned heterologous experiments results, we generated knock-in VSP mice in that the voltage-sensitivity or electrochemical coupling of mVSP was changed. We discuss how the membrane potential sensing in sperm maturation process is important for the proper phosphoinositides environment in spermatozoa flagellum

### [2S10a-3]
### A specific lipid species regulates thermal/mechanical receptors and sensory responses.

*Takaaki Sokabe[1], Takuto Suito[1], Xiangmei Deng[1], Kohjiro Nagao[2], Makoto Tominaga[1] ([1]*ExCELLS/NIPS*, [2]*Kyoto Pharmaceutical Univ*)

Membrane lipids are key elements to regulate membrane proteins and *Drosophila melanogaster* (fruit fly) is the great tool for comprehensive understanding of functional interactions of lipids and proteins. We sought for membrane lipids enriched in sensory neurons in *Drosophila* larvae and found ether phospholipids (ePLs) that possese an ether bond, instead of standard ester bond, at sn-1 position of glycerol backbone. When an ePL synthesis gene, alkylglycerone phosphate synthase (*AGPS*), was mutated in *Drosophila*, we confirmed an exclusive depletion of ePLs in the neurons.

*AGPS* mutated larvae displayed defects in responding and avoiding uncomfortable warm temperatures. Specific knockdown of *AGPS* in TRPA1-expressing neurons recapitulated the phenotype. In the presence of ePLs, TRPA1 was sensitized to temperature stimulation and showed a reduction in the thermal threshold for activation. We also found that *AGPS* mutation induced a defect in mechano-sensation in larvae. Escaping behaviors from von Frey filaments was attenuated when *AGPS* was knocked down in PIEZO-expressing neurons. When PIEZO was expressed in tissue culture cells, responses to chemical activator Yoda1 was augmented by supplementation of ePLs. We found that multiple physical properties of cell membranes were altered in the presence of ePLs, including membrane elasticity, tension and lipid order state, which could affect the responsiveness of TRPA1 and PIEZO ion channels.

We conclude that ePLs are novel lipid species that govern sensory responses to physical stimulation by tuning multiple receptor functions.

### [2S10a-4]

### The role of membrane cholesterol in modulating TRPM5 activity: Insights from an artificial reconstruction system

*Kunitoshi Uchida[1,2] ([1]*Laboratory of Functional Physiology, School of Food and Nutritional Sciences, University of Shizuoka,* [2]*Graduate School of Integrated Pharmaceutical and Nutritional Sciences, University of Shizuoka*)

TRPM5 is a monovalent cation-permeable channel that is activated by intracellular calcium, and its activity is enhanced by temperature increases. In this study, we analyzed the temperature dependence of TRPM5 using whole-cell patch-clamp recording and the planar lipid bilayer method. We observed that TRPM5 is activated and irreversibly inactivated upon exposure to heat stimulation. This temperature-dependent activation and inactivation were also observed in a POPC:POPE = 3:1 membrane incorporating purified TRPM5 protein. We then examined the effect of cholesterol on TRPM5 channel activity. Cholesterol is a major component of the cell membrane, which plays a crucial role in controlling membrane fluidity and flexibility. Interestingly, while the conductance of TRPM5 in a bilayer lipid membrane without cholesterol was 63.4 pS, the conductance of TRPM5 in a bilayer lipid membrane with cholesterol was 34.1 pS. Additionally, we found that cholesterol modulates the temperature-dependent activation of TRPM5. Consequently, membrane cholesterol could play a significant role in modulating TRPM5 channel activity.

### [2S10a-5]

### TRPV4-dependent $Ca^{2+}$ influx determines cholesterol dynamics at the plasma membrane

*Masashi Maekawa[1,2], Yutaro Kuwashima[1,3], Masataka Yanagawa[3,4], Mitsuhiro Abe[3], Yasushi Sako[3], Makoto Arita[1,2,5,6] ([1]*Division of Physiological Chemistry and Metabolism, Graduate School of Pharmaceutical Sciences, Keio University,* [2]*Laboratory for Metabolomics, RIKEN Center for Integrative Medical Sciences (IMS),* [3]*Cellular Informatics Laboratory, RIKEN Cluster for Pioneering Research (CPR),* [4]*Molecular and Cellular Biochemistry, Graduate School of Pharmaceutical Sciences, Tohoku University,* [5]*Cellular and Molecular Epigenetics Laboratory, Graduate School of Medical Life Science, Yokohama City University,* [6]*Human Biology-Microbiome-Quantum Research Center (WPI-Bio2Q), Keio University*)

The activities of the transient receptor potential vanilloid 4 (TRPV4), a $Ca^{2+}$-permeable non-selective cation channel, are controlled by its surrounding membrane lipids (*e.g.,* cholesterol, phosphoinositides). The transmembrane region of TRPV4 contains a cholesterol recognition amino acid consensus (CRAC) motif and its inverted (CARC) motif located in the plasmalemmal cytosolic leaflet. Despite the critical roles of plasmalemmal cholesterol in the regulation of TRPV4 activation, the molecular dynamics of cholesterol and TRPV4 at the plasma membrane remains unclear. In this study, we developed a single-molecule live cell imaging of plasmalemmal cholesterol by making use of a cholesterol biosensor, D4H. We also visualized the spatiotemporal interactions between cholesterol and TRPV4 at the plasma membrane in living cells by dual-color single-molecule imaging using total internal reflection fluorescence microscopy (TIRFM). Our single-molecule tracking analysis showed that the TRPV4 molecules interact with cholesterol molecules mainly in the low fluidity membrane domains in which both molecules are highly-clustered. Agonist-evoked TRPV4 activation remarkably decreased colocalization probability and association rate between TRPV4 and cholesterol molecules. Interestingly, upon TRPV4 activation, the density of cholesterol molecules was decreased and the cholesterol molecules in the fast-diffusing state were increased at the plasma membrane. The introduction of skeletal dysplasia-associated R616Q mutation into the CRAC/CARC motif of TRPV4, which reduced the interaction with cholesterol clusters, could not alter the cholesterol dynamics. Mechanistically, TRPV4-mediated $Ca^{2+}$ influx and the C-terminal calmodulin-binding site of TRPV4 are essential for modulating the plasmalemmal cholesterol dynamics. We propose that TRPV4 remodels its surrounding plasmalemmal environment by manipulating cholesterol dynamics through $Ca^{2+}$ influx.

### Symposium

[3S01m]
Research Ethics Committee
**Education Seminar**

March 30, 9:50 - 10:50, Room 1

### [3S01m-1]

### Humane care and use of laboratory animals in biomedical studies in Japan - The 2006 regime, and the future action plan

*Takehiko Bando[1] ([1]*Niigata University*)

Many efforts have been made for human health and wellness. Among them, the contributions made by biomedical studies are especially fruitful, in many of which animal experiments play a vital role. Furthermore, safety and effectiveness of newly developed drugs and medical techniques should be tested by using laboratory animals, in prior to the clinical application on patients. On the other hand, studies using laboratory animals have been criticized on the ethical aspects. In this lecture, ethical aspects of experiments using animals are firstly discussed, in comparison with experiments using human volunteers. Biomedical studies require justification to use laboratory animals both on the scientific and ethical aspects. The use of animals in scientific studies only allowed when potential benefits outweigh the expected harm. In addition, researchers should follow the high standards of humane care and treatment, such as the 3Rs principle, in their animal studies. Animals can only be used if there is no other method available. Animals are used for a limited number of the research purposes, and with refined techniques to prevent suffering. The 3Rs principle is adopted in many countries and organizations as the ethical standard for humane care and use of laboratory animals. Secondary, the systems to manage ethical aspects of studies using animals are argued. Two types of control systems are known in the world. One of them has been adopted by the EU, National license and registration are required for users of laboratory animals in European countries. Another one is developed in the US at 1985, and animal experiments were basically controlled through discussions in the Institutional Animal Care and Use Committee in each institute. The system is backed up by US law. A similar system to that in the US was introduced at 2006 in Japan. In this system, the studies using animals should be approved by the animal care and use committee in each institute, in compliance to the 3Rs principle. Animal researches in Japan are highly regulated in compliance with Japanese law, and with appropriate national and international guidelines. In addition, desirable action plan is discussed to improve Japanese system to manage animal experiments. Lastly, the efforts for social consensus on animal studies in life sciences in the EU, including the communication with public, are mentioned.

### Symposium

### [3S03m]
### RNA Modifications in Physiology and Pathophysiology

March 30, 8:50 - 10:50, Room 3

### [3S03m-1]
### tRNA modifications for optimal translation and proteostasis

*Takeo Suzuki[1] ([1]*Graduate School of Medicine, University of the Ryukyus*)

Of over 50 distinct forms of post-transcriptional modifications in human RNA, most of them are present in tRNAs. tRNA modifications confer diverse functions on tRNAs, including structural stabilization, resistance to degradation, and enhanced accuracy and efficiency of decoding. Recent findings on the metabolism-dependent regulation of tRNA modifications suggest their extended roles as a sensor of physiological conditions. Aberrant tRNA modifications disrupt cellular proteostasis and can cause human diseases such as mitochondrial disorders, neurodevelopmental disabilities, and tumor progression. We will talk about our recent progress in animal specific tRNA modifications and the relation to human diseases associated with the deficiencies in tRNA modifications, which we term "RNA modopathy".

### [3S03m-2]
### Mettl1-dependent m$^7$G tRNA modification is essential for spermatogenesis in *Drosophila*

*Kuniaki Saito[1,2] ([1]*National Institute of Genetics,* [2]*SOKENDAI*)

N$^7$-methylguanosine (m$^7$G) in the variable loop region of tRNA is catalyzed by METTL1/WDR4 heterodimer and stabilizes target tRNA. Here, we reveal essential functions of Mettl1 in *Drosophila* fertility. Knockout of Mettl1 (Mettl1-KO) loses the elongated spermatids and mature sperm, which is fully rescued by a Mettl1-transgene expression, but not a catalytic-dead Mettl1 transgene. This demonstrates that Mettl1-dependent m$^7$G is required for spermatogenesis. Mettl1-KO results in a loss of m$^7$G modification on a subset of tRNAs and a decreased level of tRNA expression. Strikingly, overexpression of the translational elongation factor, EF1α1, which can compete with the rapid tRNA decay (RTD) pathway in *S. cerevisiae*, significantly counteracted the sterility of Mettl1-KO males, supporting a critical role of m$^7$G modification of tRNAs in spermatogenesis. Ribo-seq analysis showed that Mettl1-KO increases the number of ribosome collisions at codons decoded by tRNAs that were reduced in expression. Mettl1-KO also significantly reduced the translation of genes involved in elongated spermatid formation and sperm stability. These findings reveal a developmental role for m7G tRNA modifications and indicate that m$^7$G modification-dependent tRNA stability differs among tissues.

### [3S03m-3]
### The importance of tRNA modifications in the brain

*Takeshi Chujo[1] ([1]*Faculty of Life Sciences, Kumamoto University*)

tRNA is the adaptor molecule that translates genetic information transcribed on mRNA to protein. tRNAs are posttranscriptionally decorated with a variety of chemical modifications that are incorporated by specific modifying enzymes. The tRNA modifications are important to maintain tRNA structural integrity, biochemical stability and/or appropriate codon-anticodon interactions. In humans, tRNAs collectively contain more than 40 different modifications at specific positions, and the importance of the modifications is emphasized by the existence of more than 50 human tRNA modification enzymes with pathogenic mutations. Among the 'tRNA modopathies', brain tRNA modopathies have the largest number (24) of tRNA modification enzyme genes with pathogenic mutations. However, the reason why the brain is especially susceptible to loss of tRNA modifications has been largely unknown, due to the need to generate animal models to study brain.

To elucidate how tRNA modifications support the brain, we generated whole-body knockout mice of two different tRNA methyltransferases, which are FTSJ1 that is responsible for methylations at tRNA 32nd and 34th nucleotides and TRMT10A that is responsible for methylation at tRNA 9th nucleotide. Interestingly, both of these tRNA methylase KO mice showed clear tissue dysfunctions only in the brain. Both the *Ftsj1* KO mice and *Trmt10a* KO mice showed aberrant synaptic structure, dysfunctional synaptic plasticity and impaired memory. *Ftsj1* KO brains and *Trmt10a* KO brains displayed reduced levels of specific tRNAs and slower translation of corresponding codons, that were associated with perturbed translation especially of neuron-related mRNAs. Our studies revealed how specific tRNA modifications support the brain, and I would like to discuss their commonalities, differences, and what needs to be further elucidated in this field.

### [3S03m-4]

### Regulation of hematopoietic cell differentiation and functions by RNA methylation

*Masanori Yoshinaga[1] ([1]*Department of Medical Chemistry, Graduate School of Medicine, Kyoto University*)

Hematopoiesis is a complex process that generates mature erythrocytes, leukocytes, and thrombocytes from hematopoietic stem cells (HSCs). Various mechanisms, including transcriptional, epigenetic, post-transcriptional, and post-translational regulations, govern this process. $N^6$-methyladenosine ($m^6A$) methylation, an internal RNA modification, has recently emerged as a key player in post-transcriptional regulation, impacting RNA processing, turnover, and translation. However, the specific roles of $m^6A$ modification in hematopoietic cell differentiation and functions are not yet fully understood.
Here, by conducting a genome-wide CRISPR screen, we discover that an RNA $m^6A$ methyltransferase, METTL16, plays an essential role in proper erythropoiesis. Our cellular and molecular investigations demonstrate that METTL16 controls the expression of DNA-repair-related genes via $m^6A$ modification, which is crucial for enabling erythroid differentiation. Moreover, we discover that METTL16 regulates the substrate mRNA expression in a manner dependent on the MTR4-nuclear RNA exosome complex in erythroblasts by performing a pairwise CRISPRi screen. Collectively, these findings establish that the METTL16-nuclear RNA exosome circuit acts as a novel regulatory machinery to maintain genome integrity and erythropoiesis. In this talk, I would like to introduce our recent findings and discuss the biological roles of RNA methylation.

### [3S03m-5]

### Modified adenosine metabolism safeguards energy balance

*Akiko Ogawa[1], Satoshi Watanabe[1], Kenji Inaba[1], Fan-Yan Wei[1] ([1]*IDAC*)

More than 150 types of RNA modifications have been identified in RNA species.
During RNA catabolism, most modified nucleosides derived from RNA are resistant to degradation and are released into the extracellular space. Previously, we reported that N6-methyladenosine (m6A), one of the most abundant modifications in mRNA, triggers pathophysiological immune responses by activating the G protein-coupled receptor[1)]. However, the fate of extracellularly released m6A was unknown. Recently, we identified the regulatory mechanism of m6A signalling. Using comprehensive approaches including in vitro kinetic assays, in silico modelling and knockout cell models, we show that the m6A pathway consists of multiple m6A-targeting enzymes that act in a sequential manner. Importantly, using knockout mouse models, we show that dysfunction of the m6A pathway is closely linked to imbalance in energy metabolism through impairment of the purinogenic pathway. Finally, we found a direct link between the defective metabolic processing of m6A and human disease. Taken together, our study shows that metabolic regulation of m6A is essential for cell signalling and energy balance, and that dysregulation of signalling can have catastrophic consequences.

### Symposium

### [3S04m]
### The Mystery of Deliciousness: Unraveling the Secrets of Eating through Neurophysiological Approaches

March 30, 8:50 - 10:50, Room 4

### [3S04m-1]
### Molecular mechanisms of sweet taste receptor activation/inactivation and species-specific sensitivity

*Keisuke Sanematsu[1,2,3], Yuki Nagasato[1], Masato Yamamoto[1], Yuko Kawabata[1], Shingo Takai[1], Noriatsu Shigemura[1,3] (*[1]Section of Oral Neuroscience, Graduate School of Dental Science, Kyushu University, [2]Oral Health/Brain Health/Total Health Research Center, Graduate School of Dental Science, Kyushu University, [3]Research and Development Center for Five-Sense Devices, Kyushu University*)

Taste G-protein-coupled receptor (GPCR), composed of TAS1R2 and TAS1R3 in a heterodimeric form, plays a role in mediating sweet taste. While recent studies have identified the binding sites of the sweet taste receptor for sweet substances and modulators, the mechanisms of the activation dynamics and species-specific sensitivity to ligands remain unknown. In this study, we employed molecular dynamics simulations and functional assays to clarify the activation mechanisms of TAS1R2 + TAS1R3.
Our simulations revealed dynamic allostery induced by artificial sweeteners and sweet inhibitors on the transmembrane domain of TAS1R3. Upon activation of the receptor, the allostery induced by artificial sweeteners resulted in the destabilization of the intracellular region of TAS1R3, which is the putative interface of the Gα subunit. This destabilization was accompanied by the opening of an ionic lock. The reverse was true for the effect of the sweet inhibitors. Our predictions were confirmed by functional assays involving mutations of TAS1R3. Furthermore, we identified that one residue mutation between humans and mice in the binding site of TAS1R2 determines the species-specific sensitivity of the sweet taste receptor to artificial sulfamate sweeteners. In this study, we provide not only further understanding of the sweet taste receptor function but also valuable insights into predicting dynamic activation for other G-protein-coupled receptors.

### [3S04m-2]
### Evaluation of food texture perception in rodents

*Chihiro Nakatomi[1] (*[1]Division of Physiology, Kyushu Dental University*)

Food texture is an important factor in swallowing, mastication, and food preference. However, due to the lack of established animal experimental systems, the details of the mechanism have not been elucidated and have lagged behind research on taste and smell. The difficulty in studying texture in laboratory animals is that rodents can detect the flavors of additives that impart physical properties to foods. We have investigated the evaluation system for food texture perception in rats under conditions in which the influence of flavors has been eliminated. We found that rats can detect a low viscosity of 3.6 mPa·s (equivalent to Worcestershire sauce) using the conditioned aversion tests, and that they can detect the presence of cellulose microparticles of approximately 1.5 µm in diameter using conditioned preference tests. The evaluation of texture perception using rodents is a novel approach on a global scale. By using these experimental systems in combination with the use of genetically modified animals and brain disruption experiments, it will be possible to elucidate the molecular and neurological mechanisms of texture perception.

### [3S04m-3]
### How is taste memory retrieved?

*Tadashi Inui[1] (*[1]Department of Oral Physiology, Graduate School of Dental Medicine, Hokkaido University*)

Taste memory plays a pivotal role in referring to whether food is safe. Acquisition of taste memory requires feeding followed by some postingestive consequences. For example, visceral malaise after food ingestion develops a memory of a conditioned aversive taste (CTA). Re-encountering the taste of food as a conditioned stimulus (CS) retrieves the CTA memory, resulting in a suppressed CS intake. We attempted to reveal the neural circuits underlying CTA memory retrieval. Pharmacological inhibition of neuronal activity in the basolateral amygdala (BLA) increased the CS intake in a single-bottle test. It decreased aversive responses to intra-orally infused CS in a taste reactivity (TR) test. We also found that chemogenetic inhibition of neuronal activity in the BLA increased the size of burst licking and decreased hesitation to approach the CS. These studies indicate that the BLA neurons are needed to express aversion and fear in CTA memory retrieval. An intraoral CS infusion retrieving CTA memory excited the projection neurons in the BLA, which send axons to the nucleus accumbens (NAc), the bed nucleus of the stria terminalis (BNST), and the central amygdala (CeA). NAc neurons densely project to the ventral pallidum (VP), most of which are GABAergic. An intraoral CS infusion activated the NAc-VP projective neurons and increased the extracellular release of GABA in the VP. When the $GABA_A$ receptors in the VP were blocked by microinjections of bicuculline, aversive responses in the TR test decreased, and CS intake increased in a single-bottle test. These findings imply that NAc-VP GABAergic transmission mediates aversion to CS in retrieving CTA memory. We also recently examined the effect of manipulating neurons in the CeA and BNST on the expression of behavioral responses to a CS. Inhibition of CeA neurons reduced bursting licking, whereas excitation increased it. Inhibition of BNST neurons decreased burst licking and increased the hesitating approach, whereas excitation did not alter these behaviors. Thus, it is likely that the CeA and BNST have discrete roles in retrieving CTA memory: the CeA may mediate aversion, whereas the BNST may mediate fear. These results prove that multiple brain regions and their connections play distinct roles in the expression of behavior during CTA memory retrieval.

### [3S04m-4]

### The effect of food odor and disease on proper feeding behavior

*Nao Horio[1], Stephen Liberles[1] ([1]*Harvard Medical School*)

Hunger is a powerful motivational state that intensely drives behaviors that are predictive of food consumption. Animal should prefer food cues when they are hungry in order to obtain food. For instance, we experience that the food odor is very attractive when we are hungry. However, neural mechanism by which internal state shapes preference behavior to sensory inputs remains poorly understood.

To reveal the brain mechanism for food odor preference behavior under fasted state, we conducted the behavior experiments with activation of brain neurons by optogenetic method in mice. First, with our behavior experiments called two choice odor preference test, we found that fasted mice preferred food odor over pheromone in fasted state, not in fed state. Then, we focused on the hunger-control neurons in the brain; hypothalamic agouti-related peptide (AGRP) neurons. With optogenetic activation of AGRP neurons, we found the activation of AGRP neurons and their specific projections to the paraventricular thalamus (PVT) enhanced attraction to food odors but not to pheromones. Moreover, by using knockout mice, we knew that a specific neurotransmitter, Neuropeptide Y (NPY), released by AGRP neurons and its receptor (NPY5R) are required for hunger-dependent food odor preference. Therefore, we identified a neuronal mechanism by which hunger selectively promotes attraction to food odors over other olfactory cues in mice.

Hunger is one of the physiological states to show the proper feeding behavior. Disease state is another state that changes many behaviors including feeding behavior. Sick mice ate less, and we found that this reduced amount of food is because of the decreased motivation to eat, rather than solely a physical inability to eat. We knew that in order to show the proper feeding behavior, to have a proper hungry feeling under healthy state is needed.

### Symposium

[3S05m]
Cooperation with Other Societies Committee
**Scientific understanding of the effects of acupuncture and moxibustion**

March 30, 8:50 - 10:50, Room 5

### [3S05m-1]

### Mechanisms of chronic muscle hyperalgesia -from a study using animal models-

*Asako Kubo[1] ([1]*Faculty of Rehabilitation, Niigata University of Health and Welfare*)

Tension-type headache (TTH) and fibromyalgia (FM) are representative chronic muscular pain disorders for which acupuncture is positively listed in the guidelines for the treatment of these diseases, but how the pathogenesis of these diseases remain unclear. In this symposium, I will present recent findings on the pathogenesis of them obtained from animal models of TTH and FM, respectively.
TTH and TMJ type I (myogenic TMD) are often associated with stiffness and soreness of head and neck muscles such as the trapezius muscle, which is thought to be caused by sensitization of the free nerve endings innervating the pericranial muscles. In this study, a masticatory muscle pain model was created in accordance with this pathophysiology. Ten days of myalgia induction in the trapezius muscle and tonic contraction of the masseter muscle by electrical stimulation induced mechanical hyperalgesia in the masseter muscle. Analysis of the projection pathways of primary afferents innervating the masseter muscle revealed that the primary afferents synapse with secondary neurons in the brainstem and then project to the lateral parabrachial nucleus, which projects to nuclei involved in the pain emotional system, such as the central nucleus of the amygdala. This suggests that not only sensitization of free nerve endings but also central sensitization may be involved in the onset of muscle mechanical hyperalgesia in this TTH model.
Fibromyalgia is classified as nociplastic pain because it presents various symptoms other than myalgia, such as mental distress, autonomic nervous system dysfunction and fatigue. Central sensitization is speculated to be the underlying cause. We used a rat repeated cold stress (RCS) model with many characteristics common to FM. Rats exposed to this RCS for 5 days develop persistent muscle mechanical hyperalgesia. In this model, the muscle pH under mechanical hyperalgesia was significantly decreased. Inhibition of this pH decrease reversed the mechanical hyperalgesia. Furthermore, inhibition of extracellular matrix components or acid-sensing ion channel 3, which have been implicated in mechanosensitization by low pH, also reversed the muscle mechanical hyperalgesia. These results indicate that the peripheral mechanism caused by the pH change in the muscle is also involved in the onset of muscle mechanical hyperalgesia in this FM model.

### [3S05m-2]

### Regulation of sympathetic nerve activities in the peripheral nervous system

*Kanda Hirosato[1] ([1]*Lab. of Anat., Sch. of Pharm., Hyogo Medical Univ.*)

The autonomic nervous system consists of the sympathetic and parasympathetic nervous systems, which dynamically controls homeostasis. In daily life, the body adapts to the external environment by modulating the tone of autonomic activity in response to somatosensory input. It has been known that acupuncture and moxibustion can improve various physical symptoms by regulating the activities of sympathetic and parasympathetic nervous systems. Regarding the sympathetic nervous system, the sympathetic nerve activities are modulated by sensory information primarily in the central nervous system such as paraventricular nucleus, spinal cord. While the central regulatory mechanisms of sympathetic activity have been well studied, the modification of nerve activity within the peripheral nervous system has so far remained unclear. The celiac ganglion (CG) is the site of neurotransmission between pre-sympathetic nerve terminals and post-sympathetic ganglionic neurons. Interestingly, the presence of small neural circuits in the CG has been suggested before. Here, we introduce the intra-ganglionic regulation of sympathetic activity in the peripheral nervous system. We have recently developed a rat whole-mount CG preparation, and established an *in situ* patch-clamp technique combined with high-speed pressure-clamp device. By applying this technique, we have successfully detected synaptic transmission within sympathetic ganglia. In this talk, I will introduce these projects and discuss the prospects of understanding the mechanism of acupuncture and moxibustion on autonomic nervous system.

### [3S05m-3]

### Roles of plasma membranes and mitochondria in blood flow sensing in vascular endothelium

*Kimiko Yamamoto[1], Joji Ando[2] ([1]*System Physiology, Graduate School of Medicine, The University of Tokyo,* [2]*Biomedical Engineering, School of Medicine, Dokkyo Medical University*)

Vascular endothelial cells (ECs) maintain circulatory system homeostasis by changing their functions in response to changes in hemodynamic forces, including shear stress and stretching. However, it is unclear how ECs sense changes in shear stress and stretching and transduce these changes into intracellular biochemical signals. The plasma membranes of ECs have recently been shown to respond to shear stress and stretching differently by rapidly changing their lipid order, fluidity, and cholesterol content. Such changes in the membranes' physical properties trigger the activation of membrane receptors and cell responses specific to each type of force. Artificial lipid-bilayer membranes show similar changes in lipid order in response to shear stress and stretching, indicating that they are physical phenomena rather than biological reactions. These findings suggest that the plasma membranes of ECs act as mechanosensors in response to mechanical forces. Mechanical forces first alter the physical properties of plasma membranes. Then, they modify the conformation and function of membrane proteins that activate downstream signaling pathways, including mitochondrial ATP production and purinergic $Ca^{2+}$ signaling. The latest imaging technology using cholesterol biosensors and a fluorescence resonance energy transfer-based ATP biosensor revealed that mitochondrial ATP production is mediated by plasma membrane cholesterol-dependent mitochondrial oxidative phosphorylation. Furthermore, increased mitochondrial ATP production led to ATP release from the endothelial cells, thereby activating purinoceptors in the plasma membrane and leading to purinergic $Ca^{2+}$ signaling in response to shear stress. This new appreciation of plasma membranes as mechanosensors could help to explain the distinctive features of mechanotransduction in ECs involving shear stress and stretching, which activate a variety of membrane proteins and multiple signal transduction pathways almost simultaneously.

### [3S05m-4]

### Changes in Cerebral Circulation in Patients with Major Depressive Disorder Due to Acupuncture Treatment: Evaluating Regional Cerebral Blood Flow Using Arterial Spin Labeling Magnetic Resonance Imaging

*Yuto Matsuura[1] ([1]*Department of Acupuncture and Moxibustion, Tokyo Ariake University of Medical and Health Sciences, Tokyo, Japan*)

In this symposium, we present the effect of acupuncture on patients with mood disorders, and its influence on regional cerebral blood flow (rCBF) in those with major depressive disorder (MDD), using arterial spin labeling (ASL) magnetic resonance imaging (MRI).

**Add-on Effects of Acupuncture to Standard Treatments for MDD and Bipolar Disorder**

We report a longitudinal study using a retrospective cohort on patients with MDD and depressive states of bipolar disorder (BD). In this study, 19 eligible cases were observed across three periods: before acupuncture treatment (run-in period: standard treatment only), during acupuncture (Period A: standard treatment plus acupuncture), and after treatment (Period B: standard treatment only), with each period lasting three months. Effects were assessed using the AB method. Significant reductions in depression and anxiety symptoms were observed based on the Himorogi Self-Rating Depression Scale (HSDS) and Himorogi Self-Rating Anxiety Scale (HSAS), which were measured two months after initiating acupuncture. These effects persisted for two months post-treatment for HSDS and one month for HSAS. Additionally, improvements in physical symptoms and quality of life were noted during the acupuncture treatment.

**Changes in rCBF Due to Acupuncture Stimulation in MDD Patients**

Patients with MDD exhibit either increased or decreased rCBF. We evaluated rCBF changes due to acupuncture stimulation using 3-Tesla ASL MRI to gain insights into the mechanism behind acupuncture treatment. The study included 11 MDD patients (6 men, 5 women) and 14 healthy controls (4 men, 10 women). Compared to the controls, areas like the somatosensory cortex during stimulation and the left prefrontal cortex post-stimulation were notably activated in the MDD group. Specific reductions in rCBF were observed in the bilateral temporal lobes and the right insular cortex during stimulation and in the bilateral hippocampus and amygdala post-stimulation. These localized rCBF changes in areas associated with MDD suggest that acupuncture could induce specific brain function modifications that contribute to clinical symptom improvement.

Combination standard treatments for MDD with acupuncture appears to be effective in reducing depressive symptoms. This efficacy is likely due to improvements in cerebral blood flow resulting from acupuncture stimulation.

### [3S05m-5]

### The Influence of Kampo Formula and Acupuncture Treatments on Oxytocin Secretion

*Masataka Sunagawa[1] ([1]*Dept. Physiol., Showa Univ. Grad. Sch. Med.*)

Oxytocin serves not only as a hormone but also influences the central nervous system, playing a crucial role in regulating various physiological functions, including anti-anxiety and anti-stress effects, trust and bonding formation, maternal behavior, and the amelioration of autism spectrum disorders, as well as exerting analgesic effects.
We employed a rat stress model to explore the anti-stress effects of a Kampo formula called "*Kamikihito* (KKT)" and its involvement in modulating oxytocin secretion as a mechanism of action. We randomly divided male Wistar rats into three groups: a Control group, a Restraint Stress (Stress) group, and a KKT pre-treatment followed by Restraint Stress (KKT+Stress) group. The stress load was induced by confining the subjects in a small acrylic box for 90 minutes. The KKT+Stress group exhibited a notable increase in oxytocin secretion compared to the other two groups. Subsequently, we utilized press tack needles (PTN) for acupuncture treatment on the same model animals to evaluate the anti-stress effects of acupuncture therapy and its impact on oxytocin secretion. We randomly divided male Wistar rats into three groups: a Control group, a Stress group, and a PTN treatment followed by Restraint Stress (PTN+Stress) group. We applied PTN needles (1.2mm in length) to the acupoint equivalent to the human Baihui point (GV20) and subjected them to stress 24 hours later. The PTN+Stress group displayed an increase in oxytocin secretion compared to the Stress group. These treatment methods are thought to increase resistance to stress and contribute to recovery from stress conditions by increasing the secretion of oxytocin.
Given that oxytocin has limited ability to cross the blood-brain barrier, central effects are unlikely when administered systemically. To enhance oxytocin secretion through pharmacotherapy, Kampo formula or acupuncture therapy is considered beneficial.
COI: This study was funded by TSUMURA & CO.

### Symposium

### [3S06m]
### Recent approaches to develop novel therapeutic strategies for cardiovascular diseases

March 30, 8:50 - 10:50, Room 6

### [3S06m-1]
### Systematical investigation on the role of TRPM4 channel in cardiac arrhythmogenicity

*Yaopeng Hu[1], Hiraishi Keizo[1], Jiehui Cang[1], Qin Li[2], Xin Zhu[2], Ryuji Inoue[1], Takayuki Fujita[1] ([1]*Dept. Physiol., Sch. Med., Fukuoka Univ.,* [2]*Dept. Biomed. Info. Technol., Aizu Univ.*)

The TRPM4 channel is a $Ca^{2+}$-activated monovalent cation channel abundantly expressed in cardiac Purkinje fibers, where it plays a crucial role in excitation conduction. The gating of this channel is profoundly influenced by the membrane potential, in coordination with other physiological modulators such as intracellular $Ca^{2+}$ and $PIP_2$. In this study, we systematically investigated the implications of this channel in cardiac arrhythmias, particularly in the context of its arrhythmogenic mutations.
We re-evaluated the voltage- and $Ca^{2+}$-dependent activation of TRPM4 channel using our own recording device utilizing ionomycin-mediated membrane permeabilization. Detailed analyses based on this method revealed that voltage- and $Ca^{2+}$-dependent gating of this channel is mutually interactive in both activation/deactivation processes. These results effectively explained why the overactivation of TRPM4 channel under remodeling conditions is arrhythmogenic, and also how two arrhythmic mutations of TRPM4 channel, E7K and Q854R, markedly facilitate voltage-dependent open-state transition with increased $Ca^{2+}$ sensitivity.
Numerical simulations using the most updated human Purkinje fiber single-cell action potential (AP) model (Trovato 2020) showed that facilitated opening by the above mutations significantly delays and instabilizes the repolarization of action potentials with a depolarized resting membrane potential, which eventually causes conduction blocks. Two-dimensional simulations introducing myocyte-fibroblast heterogeneities demonstrated that the increased density/activity of TRPM4 channels can generate various degrees of conduction blocks and complex patterns of propagation disturbances, indicating a significant predisposition to ventricular arrhythmias. Finally, in Langendorff-perfused ex vivo hearts, the TRPM4 selective blocker 9-phenanthrol strongly suppressed ventricular tachyarrhythmias induced by balloon inflation that mechanically stimulates endocardial Purkinje fibers.

### [3S06m-2]
### Lysine crotonylation modulation in cardiomyocytes represent a novel therapeutic target in heart disease

*Wenqian Cai[1,2] ([1]*Guangzhou Medical University,* [2]*Guangzhou Women and Children's Medical Center*)

BACKGROUND: Heart disease is still a leading cause of morbidity and mortality worldwide. Post-translational modification of protein lysine residues plays akey role in regulating cellular functions in both physiological and pathological states. Lysine crotonylation (Kcr) was recently identified as a post-translational histone modification that found to occur in a large number of histone proteins. However, the role of Kcr in myocardial injury has been rarely reported. Here, we performed proteomics analysis to determine the profiling and pathophysiological significance of Kcr modification following cardiac injury and explore the underlying mechanism.METHODS: We investigated the dynamic change of both the Kcr sites and protein level in left ventricular tissues following sham or cardiac injury (including ischemia-reperfusion and cardiac hypotrophy) followed by liquid chromatography-coupled tandem mass tag mass spectrometry. After validation of the enriched protein Kcr by immunoprecipitation and immunoeletron microscopy, the function and mechanism of specific Kcr sites were further investigated in vitro and in vivo by gain- or loss-of-function mutations targeting Kcr sites of selected proteins.RESULTS: We found that cardiac injury triggers Kcr of proteins required for cardiomyocyte contractility, including mitochondrial, cytoskeleton proteins and sarcoplasmic reticulum calcium channel, which occurs largely independently of protein-level changes in the same proteins, indication specific location of Kcr in cardiomyocytes. Modulating site-specific Kcr of selected mitochondrial protein IDH3a (isocitrate dehydrogenase 3 [NAD+] alpha) at K199 protects cardiomyocyte from apoptosis by inhibiting BNIP3 (Bcl-2 adenovirus E18 19-kDa–interacting protein 3)-mediated mitophagy. Cytoskeletal protein TPM1 (tropomyosin alpha-1 chain) at K28/29cr enhanced cytoskeleton structure remodelling but also preserves postinjury myocardial function by inhibiting fibrosis and apoptosis. However, K157cr of SERCA2a (sarcoplasmic reticulum calcium ATPase 2A) decreased cardiac function in mice hypotrophy. CONCLUSIONS: Our results indicate that Kcr modulation is a key response of cardiomyocytes to cardiac injury and may represent a novel therapeutic target in the context of heart disease.

### [3S06m-3]
### Hippo signal in pulmonary arterial hypertension

*Aya Yamamura[1], Alamgir Hossain[1], Motohiko Sato[1] ([1]*Department of Physiology Aichi Medical University*)

Pulmonary arterial hypertension (PAH) is a progressive and fatal disease in the cardiovascular system. The major pathogenesis is sustained vasoconstriction and vascular remodeling of the pulmonary artery, which is largely mediated by an increase in cytosolic $Ca^{2+}$ concentration in pulmonary arterial smooth muscle cells (PASMCs). We previously demonstrated that the expression of $Ca^{2+}$-sensing receptors (CaSRs) was upregulated in PASMCs from idiopathic pulmonary arterial hypertension (IPAH) patients, enhanced $Ca^{2+}$ signaling, and resulted in pulmonary vascular remodeling. The molecular mechanisms underlying the upregulation of CaSR expression were examined in PASMCs from normal subjects and IPAH patients. In normal-PASMCs, the expression of CaSRs was upregulated by platelet-derived growth factor (PDGF) stimulation, which is known as a pathological signal associated with PAH. The expression of PDGF receptors was higher in IPAH-PASMCs than in normal-PASMCs. In the present study, DNA microarray analysis using IPAH-PASMCs revealed Hippo signaling as a downstream pathway of PDGF receptors. Hippo signaling is known to be associated with cell life, proliferation, and differentiation. In IPAH-PASMCs, the expression of YAP (a central molecule of Hippo signaling) and TEAD (a transcription factor) was increased. In addition, the treatment with PDGF-BB on normal-PASMCs for 48 h increased the expression of YAP, TEAD, and CYR61 (a fibrosis marker). Their upregulations were suppressed by siRNA knockdown of PDGF receptor β. These results strongly suggest that Hippo signaling functions as a downstream pathway of PDGF receptors, contributing to the development of vascular remodeling in PAH.

### [3S06m-4]

### Ischemic tolerance of the heart based on sulfur metabolism

*Akiyuki Nishimura[1], Xialkang Tang[1], Yuri Kato[2], Motohiro Nishida[1,2] ([1]*Division of Cardiocirculatory Signaling, National Institute for Physiological Sciences,* [2]*Grad. Sch. Pharmaceut. Sci., Kyushu Univ.*)

The robustness of cardiomyocytes is supported by their superior redox homeostasis, and disruption of redox homeostasis leads to the onset and progression of cardiac disease. Redox research has so far focused on oxygen such as reactive oxygen species (ROS). On the other hand, supersulfides, which include catenated sulfur atoms (R-$S_n$SH) such as cysteine persulfide (Cys-SSH), have been recently identified as highly reactive sulfur metabolites and recognized as a key molecule to regulate redox homeostasis in both prokaryotes and eukaryotes. The aim of this study was to elucidate the role of sulfur metabolism in maintaining cardiac robustness, and the impact of abnormal sulfur metabolism on ischemic heart disease.
We established an imaging-based method to visualize supersulfides in a cell and tissue using chemical probes and found that supersulfides are reduced to hydrogen sulfides in the heart of myocardial infarction (MI) model mice. The depletion of supersulfides decreased the contractile function of cardiomyocytes through mitochondrial hyperfission. We also found that the depletion of supersulfides promotes mitochondrial fission by decreasing polysulfidation of mitochondrial fission factor Drp1 at Cys644.
Our results suggest that the redox modification of Drp1 Cys644 has a pivotal role in the ischemic tolerance of cardiomyocytes. We found that this polysulfidated Cys644 is modified by S-glutathionylation. Biochemical analysis revealed that Drp1 is glutathionylated by oxidized GSSG but not reduced GSH. GSSG-mediated Drp1 glutathionylation inhibited hypoxia-induced Drp1 activation, leading to myocardial dysfunctions. To evaluate cardioprotective effects of GSSG on MI-model mice, GSSG was administrated into mice one week after MI operation. Additionally, MD simulation of Drp1 structure revealed that bulky modification at Cys644 via polysulfidation and glutathionylation reduces Drp1 activity by disrupting Ser637-Glu640-Cys644 interaction. Our findings suggest a novel therapeutic potential of polysulfur-based Cys bulking on Drp1 for ischemic heart disease.

### Symposium

### [3S08m]
### Exploring the principle of the State-switching mechanism in physiological phenomena

March 30, 8:50 - 10:50, Room 8

### [3S08m-1]
### Mechanisms of experience-induced shift of aggression state in the mouse

*Aki Takahashi[1] ([1]*Institute of Human Sciences, University of Tsukuba*)

The male mouse shows territorial aggressive behavior toward a rival male. This behavior can be modified by previous winning or losing experiences with the other males, and the winner tends to increase aggressive behavior while the loser reduces their aggressive behavior in the following encounter. Previously, we have shown that the preceding indirect exposure to the rival male enhances aggressive behavior in the following aggressive encounter, named as social instigation procedure. In this talk, we will discuss how previous winning- and losing-experiences can modulate the effect of social instigation on aggressive behavior. Also, we have shown the involvement of the dorsal raphe nucleus (DRN) in this increase of aggressive behavior by social instigation. Especially, glutamatergic inputs from the lateral habenula (LHb) to the DRN (LHb-DRN projections) were increased during the social instigation-heightened aggression. Both optogenetic and chemogenetic inhibition of the LHb-DRN projection blocked instigation-heightened aggression, while optogenetic activation of this projection increased inter-male aggression. On the other hand, LHb-DRN inhibition did not affect baseline aggression without social instigation. Thus, these data indicate that LHb-DRN projection is specifically involved in the escalation of aggressive behavior from its baseline, but not required for the expression of species-typical aggressive behavior. Anatomical analysis showed that DRN neurons that receive input from the LHb project to the ventral tegmental area (VTA), and optogenetic activation of the DRN to VTA projecting neurons increased inter-male aggression. On the other hand, we found that optogenetic activation of 5-HT neurons blocked social instigation-heightened aggression without affecting species-typical aggressive behavior. These results indicate that the DRN contains neurons that have bidirectional effects on the escalation of aggression induced by social instigation.

### [3S08m-2]
### Optical measurements of brain environment shifts dependent on brain states

*Yoko Ikoma[1], Daichi Sasaki[1], Yusuke Takahashi[1], Ko Matsui[1] ([1]*Super-network Brain Physiology, Graduate School of Life Sciences, Tohoku University*)

To understand the role of astrocyte activities in controlling the state of the brain, fluorescent sensor proteins were genetically expressed in the astrocytes of mice. An optical fiber was implanted into the lateral hypothalamus of the mouse brain to send excitation light and record fluorescence signals. Such fiber photometry is widely used in current neuroscience research; however, many studies have ignored the effects of the changes in the local brain blood volume (BBV) and the cytosolic pH on the fluorescent signals detected. Here, rather than attempting to remove these troublesome effects from BBV and pH, we devised a method to read as much as possible of the local environmental parameters from the detected fluorescent signal. This newly devised evaluated astrocytes' activity and critical components of local brain environmental changes were dissected.Using epileptogenesis as an extreme form of plasticity, the present study examined plastic changes in astrocyte responses in the lateral hypothalamus. The lateral hypothalamus is a part of the brain known to be vital for whole-body metabolism and sleep/awake control. Fluorescent sensors of calcium or pH expressed in astrocytes were examined by in vivo fiber photometry using freely moving transgenic male mice for up to 1 week. We showed that exacerbation of epilepsy in a hippocampal stimulation kindling model results in the appearance of an acid response in astrocytes in the lateral hypothalamus. The astrocyte acid response leads to the amplification of excitatory neuronal signaling and may be the underlying driving force for plasticity in epileptogenesis. We also captured changes in the brain environment in sudden epilepsy death and found the boundary response that separates life and death during seizures.Such pH fluctuation in the cytosol may occur only in an extreme pathological situation such as epilepsy and ischemia. However, using the same optical analysis technology, we found that REM sleep can also produce a robust acid response in the astrocytes. In addition, glial acidification during REM sleep was enhanced in a state where epileptic seizures were likely to occur. Controlling the astrocyte pH could be a new therapeutic target for the treatment of epilepsy and the prevention of undesired plasticity associated with epileptogenesis.

### [3S08m-3]
### Elucidating the mechanism of action of Aripiprazole in regulating phase-shift of the circadian rhythms

*Arisa Hirano[1], Ruoshi Li[1], Takeshi Sakurai[1] ([1]*University of Tsukuba, Institute of Medicine/ IIIS*)

Aripiprazole has long been clinically used as a treatment for psychiatric disorders such as schizophrenia and bipolar disorder. Many patients with these psychiatric conditions frequently experience disruptions in their sleep–wake cycles called circadian rhythm sleep disorder. Several case studies and clinical trials have shown that the administration of aripiprazole for the treatment of bipolar disorder or major depression alleviates the symptoms of circadian sleep disorders in these patients. This improvement may be attributed to the direct effects of aripiprazole on the circadian central clock, specifically the hypothalamic suprachiasmatic nucleus (SCN), which regulates various circadian physiological rhythms, including the sleep–wake cycle, in mammals. To examine whether aripiprazole facilitates adaptation to changes in the light–dark cycle, we orally administered aripiprazole to mice and subjected them to jet-lag experiments. Mice receiving aripiprazole were more rapidly entrained to 6 h advanced light–dark cycles. Moreover, we examined the effect of aripiprazole on the cellular rhythms of SCN slice cultures and found that aripiprazole disrupted cellular synchronization among the SCN neurons, thereby accelerating the damping of the SCN rhythm at the population level. Adenosine 3'5' monophosphate (cAMP) assay using a bioluminescence indicator revealed that intracellular cAMP level in the SCN increased following aripiprazole treatment. However, this increase was blocked by pre-treatment with the serotonin 1A receptor ($5\text{-}HT_{1A}R$) antagonist. Based on these findings, we propose that aripiprazole modulates intracellular signaling, including $5\text{-}HT_{1A}R$-mediated cAMP signaling, and desynchronizes SCN neurons, ultimately leading to enhanced entrainment to phase advanced light–dark cycles in mice. These findings indicate that the improvement in sleep symptoms reported in patients with psychiatric disorders receiving aripiprazole may be due to modulation of the circadian clock. Our study provides novel insights into the potential clinical applications of aripiprazole in patients with various circadian sleep disorders.

### [3S08m-4]

### Dissecting the cortical dynamics underlying developmental disorders

*Aya Ito-Ishida[1] ([1]*RIKEN Center for Brain Science*)

Rett syndrome is a postnatal neurodevelopmental disorder caused by the loss of methyl-CpG binding protein 2 (MeCP2) and is characterized by multiple neurological symptoms such as autism, regression, and sensory-motor deficits. While previous studies have demonstrated the necessity of MeCP2 for normal neuronal function and identified abnormal excitatory synapses within local circuits, the broader impact on global connectivity remains elusive. To examine the global cortical connectivity changes in Rett syndrome, we conducted macroscopic calcium imaging in Mecp2-heterozygous female mice expressing GCaMP6f in forebrain excitatory cells. We obtained images from the entire dorsal cortex that spanned major cortical areas. Dual excitation illumination at 470 nm and 405 nm effectively removed calcium-independent signals. Analyzing data from over 15 mice, we constructed functional connectivity matrices based on correlations in calcium activity. We applied combinatorial techniques, including machine learning, graph theory, and principal component analysis, to examine the correlation matrix. Our results revealed altered cortical connectivity in Mecp2-heterozygous mice. Notably, we detected the most significant reduction in the corticocortical connection that is centered around midline association areas strongly linked to sensory and motor regions. Furthermore, the results from the principal component analysis emphasized changes in these midline cortical areas. In summary, our findings suggest selective impairment of cortical connectivity in Rett syndrome, with a focus on inter-regional connectivity centered around the midline association cortex. Given that impaired cortical connectivity is a crucial pathology in autism spectrum disorders, our study offers valuable insights into the mechanisms underlying cortical disturbances.

### Symposium

[3S10m]
**System of cells and organs for responses against environmental changes in epidermis**

March 30, 8:50 - 10:50, Room 10

### [3S10m-1]

### Regulation of human keratinocyte stem cells through thermoTRP channels – mTORC1 signaling axis

*Daisuke Nanba[1] ([1]*Division of Regenerative Medicine and Therapeutics, Department of Genomic Medicine and Regenerative Therapy, School of Medicine, Faculty of Medicine, Tottori University*)

Adult autologous human epidermal stem cells can be extensively expanded *ex vivo* for cell and gene therapy. Identifying the mechanisms involved in stem cell maintenance and defining culture conditions to maintain stemness is critical because an inadequate environment can result in the rapid conversion of stem cells into progenitors/transient amplifying cells (clonal conversion) with deleterious consequences on the quality of the transplants and their ability to engraft. Here we demonstrate that cultured human epidermal stem cells respond to a small drop in temperature through thermoTRP channels connected to mTOR signaling. Exposure of cells to rapamycin or a small drop in temperature also induces the nuclear translocation of mTOR with an impact on gene expression. We also demonstrate by single-cell analysis that long-term inhibition of mTORC1 reduces clonal conversion and favors the maintenance of stemness. Taken together our results demonstrate that human keratinocyte stem cells can adapt to environmental changes (e.g, small variations in temperature) through mTOR signaling, and constant inhibition of mTORC1 favors stem cell maintenance, a finding of paramount importance for regenerative medicine applications.

### [3S10m-2]

### Sweat and Skin: A Delicate Balance

*Hiroyuki Murota[1] ([1]*Department of Dermatology, Graduate School of Biomedical Sciences, Nagasaki University*)

Sweat plays a vital role in the body's ability to maintain a healthy state. It regulates body and skin temperature, provides biological defense against pathogens, and moisturizes the skin to maintain a healthy skin condition. Under certain circumstances, however, sweat can cause itching. For example, excess sweat on the skin surface in an occlusive condition for an extended period, skin problems may occur due to maceration of the horny layer. On the other hand, decreased sweating will make the skin dry, and increased skin temperature and homeostasis will be impaired. Physicians frequently hear complaints of skin diseases caused or aggravated by sweat in clinical practice. Atopic dermatitis is a typical example. In addition, abnormal sweating may be observed secondary to systemic diseases or rare intractable diseases. This lecturer reviews the relationship between sweating and skin diseases based on current evidence.

### [3S10m-3]

### Establishment of human eccrine sweat gland research and its application to cosmetics

*Hiroko Kato[1] ([1]*Osaka University*)

Human eccrine sweat glands exist almost throughout the body and play a major role in thermoregulation, while mouse sweat glands are found only in the palms and are thought to function differently because they function to prevent slipping. Therefore, we have been studying human eccrine sweat glands in order to understand their basic functions for developing antiperspirants. First, we clarified the three-dimensional coil structure of the human eccrine sweat gland and searched for expression markers of the secretory part and conduits, which are the constituent parts of the coil region of the sweat gland, as well as the orientation of blood vessels and nerves. Next, we attempted to identify stem cells and fractionate them using surface markers in order to set up a culture system for human eccrine sweat glands. In the secretory part of human eccrine sweat glands, myoepithelial cells are stem cell-like cells, and spheres with undifferentiated myoepithelial cells and differentiated luminal cells were formed when myoepithelial cells were cultured in spheroid. However, primary myoepithelial cells could not be cultured for a long period of time, so an immortalized cell line was established by introducing an immortalization gene. This immortalized cell line was shown to have the same differentiation potential as the primary cells. On the other hand, it had been unclear how sweat was secreted from the eccrine sweat gland, therefore, we attempted to clarify this by observing the dynamics of the excised sweat gland using live imaging. We have successfully observed the contraction of myoepithelial cells around the secretory ducts. We searched for a material that inhibited the contraction of sweat glands and confirmed that treatment by the material to sweat glands not only inhibited sweating movement *in vitro* but also inhibited human armpit sweat caused by exercise *in vivo*. By applying this new sweat suppression technology to products, we hope to improve the quality of life of people suffering from sweating.

### [3S10m-4]

### Percutaneous penetration of hydrophilic and lipophilic compounds through glycated skin and its mechanism

*Yoshihiro Tokudome[1] ([1]*Saga Univ.*)

The main body of intercellular lipids in the stratum corneum is composed of ceramide (CER), cholesterol (Chol), and fatty acids (FA) in approximately equal molar amounts, forming a lamellar structure. The stratum corneum, the outermost layer of the skin, provides a barrier to the penetration of compounds that is the regarded as the most significant obstacle to transdermal penetration. Advanced glycation end products (AGEs), which are linked to both aging and hyperglycemia, cause marked functional and structural alterations in human skin. AGEs are generated via the non-enzymatic Maillard reaction between reducing sugars and proteins, lipids or nucleic acids. Moreover, glycation of dermal collagen and elastic fibers contributes to stiffness and loss of elasticity, forming wrinkles. In addition to the modification of structural proteins, AGEs also induce biological reactions via binding to receptor for AGEs (RAGE). For these reasons, it is expected that alteration of the membrane properties of glycated skin causes changes in its permeability to compounds. We investigated the effect of glycation on lipid content in cultured reconstructed skin. In addition to transepidermal water loss, content of intercellular lipids in the reconstructed epidermal model were analyzed. Expression of genes related to ceramide metabolism was determined. It was found that FA was significantly increased by glycation. CER[NS], [AP], and cholesterol were decreased in glycated epidermis. Expression of ceramide synthase 3 (CERS3) was significantly decreased while fatty acid elongase 3 was increased by glyoxal in a dose dependent manner. The effects of glycation on skin permeation and accumulation of compounds were evaluated using an in vitro glycated skin model. Flux and accumulation in the skin were determined by applying hydrophilic and lipophilic molecules to this in vitro glycated skin model. Flux across glycated full-thickness skin was higher than that across normal skin, although there was no difference with lipophilic molecules. These results suggest that glycated SC and epidermis–dermis differentially regulate the permeability of hydrophilic molecules and highlight the importance of controlling drug delivery by modifying the formulation or method of application depending on skin condition. These results indicate that changes in intercellular lipid metabolism in the stratum corneum due to glycation disrupt the composition ratio of intercellular lipids in the epidermis. Furthermore, they show that the barrier function of the skin against water-soluble molecules is altered through structural changes caused by cross-linking denaturation, indicating the importance of preventing skin glycation in terms of barrier function.

### [3S10m-5]

### The roles of thermo-sensors in responses for embironmental changes

*Fumitaka Fujita[1,2] ([1]*Graduate School of Pharmaceutical Sciences, Osaka University,* [2]*Mandom corporation*)

Epithelial tissue, especially the skin, is constantly exposed to major environmental changes, pathogens and chemicals, but it has an elaborate mechanism for maintaining tissue through a flexible cell response and control system. Thirty years ago, we never imagined that one molecule could sense temperature and sensory irritations on the skin surface. In 1997, it was discovered that an ion channel called TRPV1 responded to not only high temperature but also capsaicin, which dramatically changed understanding of sensory perception on skin. We have clarified that TRPA1, which was found in 2003, is related to sensory irritation by various chemicals and environmental changes, such as anti-bacterial agents, alkali solution, hypotonic solution and fragrance chemicals. Recently, we found that TRPM4 act as an intrinsic immune regulatory molecule in keratinocytes and a new nature-derived TRPM4 agonist aluminum potassium sulfate. Moreover, TRPM4 activation influenced cell proliferation in various temperatures. In addition, we demonstrated that TRPV4 activation inhibits NFκB signaling, resulting in the suppression of IL-1β production in both monocytes and macrophages. A TRPV4 activator also inhibited the differentiation of monocytes into GM-CSF M1 macrophages but not M-CSF M2 macrophages. We also observed a significant increase in the number of iNOS-positive/TRPV4-negative dermal macrophages in AD compared to healthy skin specimens. These findings show thermosensitive TRP channels have important roles in regulation of immune responses in skin.

### Symposium

[3S01a]
Academic and Research Committee

**Joint Symposium of the Japanese Physiological Society, the Japanese Association of Anatomists, and the Japanese Pharmacological Society"Future society opened up through the diversity of sleep"**

March 30, 14:20 - 16:20, Room 1

### [3S01a-1]

### Evolution of Sleep and its Physiological Significance: From "Lower" Organisms (Hydra) to Mammals

*Taichi Q Itoh[1] ([1]*Faculty of Arts and Science, Kyushu University*)

Sleep is a widely observed physiological phenomenon in vertebrates, extending beyond mammals to various species. Recent research has confirmed the presence of non-REM sleep and REM sleep, which were long considered exclusive to mammals, in reptiles and fish. Studies on sleep in invertebrates, using model organisms such as *Caenorhabditis elegans* and *Drosophila melanogaster*, were reported in the early 2000s. These studies primarily rely on behavioral indicators to define sleep, often referred to as 'sleep-like states.' However, the sleep regulatory factors in these animals often exhibit similarities with those of other species, and research utilizing these organisms in the study of sleep remains active. A common feature among animals that exhibit sleep is the presence of a central nervous system, primarily located in the brain. The physiological significance of sleep is intricately linked to brain functions, such as memory consolidation, underlining the crucial relationship between sleep and the brain. Nevertheless, recent discoveries have revealed the presence of sleep phenomena in cnidarians like Jellyfish and Hydra, which lack a central nervous system. The shared characteristics of sleep-regulating factors in cnidarians and other species have raised the possibility that sleep in cnidarians might have primitive origins in the evolution of sleep. Hence, our research group aims to deepen our understanding of the fundamental significance of sleep in animals by exploring this primitive sleep phenomenon. Furthermore, the unique characteristics of Hydra, such as remarkable regenerative abilities, resistance to starvation, and potential immortality, prompt investigations into their relationship with sleep. This presentation will provide an overview of our research findings and discuss how these 'lower' and 'peculiar' organisms might contribute to the future of sleep research. Through these insights, we anticipate offering novel perspectives on the evolution and physiological significance of sleep, thereby enhancing our understanding of the future of sleep research.

### [3S01a-2]

### The Mechanism of Hibernation and its Clinical Application

*Genshiro A. Sunagawa[1] ([1]*RIKEN BDR*)

Hibernation is a natural process that occurs in some animals when food or energy shortages. During hibernation, the animals actively switch off their metabolism to lower their body temperature and reduce energy expenditure, which is of great interest owing to its potential medical benefits. However, the underlying mechanisms of hibernation are not known yet. One major obstacle in hibernation research is the inability to induce hibernation in animals instantly. Fortunately, we have demonstrated that the excitation of QRFP-containing neurons (Q neurons) at the mouse hypothalamus causes a long-lasting hypometabolic and hypothermic state that resembles hibernation (Takahashi TM et al., Nature, 2020). This breakthrough has transformed hibernation basic research into an experiment-testable field. We named this state QIH after Q neurons–induced hypometabolism, which can be induced on-demand by stimulating the genetically expressed receptors on the Q neurons pharmacologically or optogenetically. We are testing how the hibernation-like state affects physiological and pathophysiological conditions, fully taking advantage of QIH. Remarkably, inducing QIH has been found to suppress several acute diseases (Kyo S et al., JTCVS open, 2022), providing evidence of the potential utility of synthetic hibernation to humans. These findings open up new avenues for research into hibernation and the potential medical applications of this fascinating transition in metabolic state.

### [3S01a-3]

### Sensory medicine and drug discovery technology to induce artificial hibernation and life-protective state by innate fear odors

*Reiko Kobayakawa[1], Tomohiko Matsuo[1], Ko Kobayakawa[1] ([1]*Inst. Biomed. Sci., Kansai Medical Univ.*)

In the course of evolution, humans and animals have evolved potential protective abilities to survive in critical situations. However, at present, it is still unclear what kind of protective abilities exist, what kind of stimulation induces these abilities, and whether the stimulation method can be applied as a medical technology. It is also thought that some system in the brain is responsible for recognizing critical situations and inducing appropriate protective abilities. However, the whole picture of this system is also yet to be revealed. We have developed a series of odor molecules named "thiazoline-related fear odor (tFO)," which have activity to induce an innate fear response and have been working to elucidate the neural mechanisms that control the innate fear responses. In this series of studies, we discovered an intriguing link between innate fear and life-protective effects, specifically, the novel life phenomenon that tFO binds to TRPA1 receptors in the trigeminal/vagus nerves and transmits crisis information to the brainstem-midbrain pathway, thereby inducing a protective capability that enables survival in lethal environments and pathological conditions. Based on this concept, we named the technology to artificially induce desirable physiological responses and metabolic and gene expression that improve pathological conditions "sensory medicine and drug discovery." The latest findings on the characteristics and mechanisms of sensory medicine and drug discovery technology that induces artificial hibernation and a life-protecting state by innate fear odor stimuli will be introduced.

### [3S01a-4]

### SIK3 variants regulate sleep

*Hiromasa Funato[1] ([1]*Toho Univ*)

Sleep is homeostatically modulated, although the intrinsic biological mechanisms orchestrating sleep remain enigmatic. Previously, using a forward genetic approach, we identified the protein kinase SIK3 and HDAC4 as pivotal elements in NREM sleep regulation. Loss of a PKA phosphorylation site, serine551, of SIK3 resulted in increased sleep. Using a variety of Cre driver lines, we examined the sleep/wakefulness of mice that lack S551 in specific neuron groups, which shows that a specific set of neurons regulate sleep via the SIK3 pathway. There are several SIK3 variants. Genetic deletions of specific SIK3 variants indicate the differential roles of SIK3 variants in sleep and circadian behavior. Single nucleus RNA-seq exhibited several genes expressed in cortical excitatory neurons, related to sleep need encoding. I will discuss how an intracellular signaling pathway regulates time spent in NREM sleep and EEG delta power during NREM sleep.

### [3S01a-5]

### Toward the establishment of human systems biology using sleep-wake rhythm as a model.

*Yoichi Minami[1], Akifumi Kishi[1], Hiroki Ueda[1,2] ([1]*The University of Tokyo, Graduate School of Medicine, Department of Systems Pharmacology,* [2]*Laboratory for Synthetic Biology, RIKEN Center for Biosystems Dynamics Research*)

The ERATO Ueda Biological Timing Project, launched in 2020, aims to establish human systems biology by employing sleep-wake rhythms as a model system. The challenge at hand is to comprehend sleep and wakefulness' "biological timing" information from the molecular to individual levels. Over recent years, the significance of achieving "healthy sleep" has become more apparent. To enhance accessibility in sleep measurement, our laboratory is tackling the development of sleep analysis technology through wearable terminals.

Ode et al. developed ACCEL, an algorithm to define sleep and wakefulness from acceleration data (Ode et al., iScience, 2022). Katori et al. confirmed the feasibility of this algorithm using acceleration data from approximately 100,000 subjects living normal lives collected by the UK biobank (Katori et al. PNAS, 2022). Using this core technology, we are trying to conduct large-scale monitoring of sleep with wearable devices to assess people's sleep quality, identify problems, and ultimately lead to improve overall health through better sleep. To this end, we are proposing a "Sleep Health Checkup". Our project focuses specifically on the quantitative assessment of children's sleep on a large scale. At this symposium, I will present our project and introduce our work on children's sleep.

Minami & Kishi has no COI to disclose. Ueda is the founder of ACCELStars, Inc.

### Symposium

### [3S02a]
### Elucidation of the pathogenesis of heart diseases and development of new therapeutic strategies

March 30, 14:20 - 16:20, Room 2

### [3S02a-1]
### The role of the junctophilin in the pathophysiology of heart failure

*Tstuomu Nakada[1], Takuro Tomita[2], Mitsuhiko Yamada[2] ([1]*Shinshu Unieristy, Research Center for Advanced Science and Technology,* [2]*Shinshu Unieristy School of Medicine, Department of Molecular Pharmacology*)

To ensure normal contraction of cardiac and skeletal muscles, it is essential for the membrane of the cell and the sarcoplasmic reticulum membrane to be closely associated, with functional interaction between L-type calcium channels (LTCC) and ryanodine receptors. It is known that junctophilin (JP) plays a crucial role in maintaining this membrane association. Previous studies have revealed that in skeletal muscles, JP1 binds to LTCC and regulates its intracellular localization. However, there are many unknowns regarding the relationship between JP2 and LTCC in cardiac muscles. In this study, a C-terminal truncated mutant of JP2 (JP2Δ427) was expressed in mouse cardiac muscle cells using an adeno-associated virus vector, and its in vivo effects were examined. Expression was driven by the Troponin T promoter. Four weeks after viral administration, strong expression of JP2Δ427 in the cardiac muscles were confirmed by immunostaining and Western blotting. furthermore, a significant increase in heart weight was observed. However, there were no significant changes in lung weight, heart rate, or blood pressure. Analysis by echocardiogram showed that the %FS in the control group was 43%, while in the JP2Δ427 group, it was reduced to 31%. Calcium imaging of isolated cardiac muscle cells revealed a significant decrease in calcium transients induced by electrical stimulation in the JP2Δ427 group. Immunostaining results showed that compared to T-tubules, JP2Δ427 was more abundantly distributed in the surface cell membrane. Examination of LTCC in the same cells revealed a higher localization on the surface cell membrane compared to the control group. These results suggest that overexpression of JP2Δ427 induces alterations in the intracellular localization of LTCC, leading to disruptions in calcium metabolism and cardiac function.

### [3S02a-2]
### The potential of RyR2 inhibitors as a new class of antiarrhythmic drugs

*Nagomi Kurebayashi[1] ([1]*Dept Pharmacol, Juntendo Univ Fac Med*)

Hyperactivation of the type 2 ryanodine receptor (RyR2) is known to cause lethal arrhythmias such as catecholaminergic ventricular tachycardia (CPVT) and chronic heart failure, where spontaneous $Ca^{2+}$ release via overactivated RyR2 depolarizes diastolic membrane potential to induce triggered activity. In such diseases, reduction of RyR2 activity is thought to suppress arrhythmias, but there are no clinically available antiarrhythmic drugs with pure RyR2-specific inhibitory activity. We searched for RyR2 inhibitors by a high-throughput screening based on an ER $Ca^{2+}$-based assay and found several compounds that selectively suppress RyR2 activity. In isolated cardiomyocytes, these RyR2 inhibitors suppressed generation of arrhythmogenic $Ca^{2+}$ waves and sparks without affecting action potential-induced $Ca^{2+}$ transients. In addition, we further developed a high-affinity (IC50 of ~15 nM) and selective RyR2 inhibitor based on one of the hit compounds identified in the high-throughput screening. This compound, TMDJ-035 effectively suppressed arrhythmias in CPVT mouse models harboring mutant RyR2s. Unlike conventional antiarrhythmic drugs (i.e., Na channel inhibitors, Ca channel inhibitors, ß-blockers), TMDJ-035 did not affect ECG parameters or cardiac contractile function at the effective doses. These results suggest that RyR2 inhibitors can represent a promising new class of antiarrhythmic drugs.

### [3S02a-3]
### Modeling calmodulin-related life-threatening arrhythmias using iPS cells

*Takeru Makiyama[1,2], Jingshan Gao[2], Yuta Yamamoto[2], Takuya Kobayashi[3], Aizawa Takanori[2], Hai Huang[2], Asami Kasahiwa[2], Tomohiko Imamura[2], Hisaaki Aoki[4], Koichi Kato[5], Megumi Fukuyama[5], Futoshi Toyoda[6], Seiko Ohno[7], Naomasa Makita[8], Nagomi Kurebayashi[3], Takashi Murayama[3], Miinoru Horie[5], Takeshi Kimura[2], Koh Ono[2] ([1]*Department of Community Medicine Supporting System, Kyoto University Graduate School of Medicine,* [2]*Department of Cardiovascular Medicine, Kyoto University Graduate School of Medicine,* [3]*Department of Pharmacology, Juntendo University School of Medicine,* [4]*Department of Pediatric Cardiology, Osaka Women's and Children's Hospital,* [5]*Department of Cardiovascular Medicine, Shiga University of Medical Science,* [6]*Department of Physiology, Shiga University of Medical Science,* [7]*Medical Genome Center, National Cerebral and Cardiovascular Center,* [8]*Omics Research Center, National Cerebral and Cardiovascular Center*)

Calmodulin (CaM) is a ubiquitously expressed, multifunctional $Ca^{2+}$ sensor molecule that regulates numerous proteins. In humans, CaM is encoded by three different genes (*CALM1*, *CALM2*, and *CALM3*) which produce identical amino acid sequences. Recently, mutations in any of the three genes (*CALM 1–3*) have been identified in patients with life-threatening arrhythmias, such as long QT syndrome (LQTS) and catecholaminergic polymorphic ventricular tachycardia (CPVT). However, the exact mechanism of CaM-related arrhythmias in human cardiomyocytes (CMs) remains unclear. To investigate the underlying disease causing mechanisms, we established disease model associated with *CALM* mutations using human induced pluripotent stem (iPS) cells. In this session, we present our findings on *CALM*-related arrhythmias using iPS cell models.

We generated LQTS-iPSCs from a 12-year-old boy with LQTS carrying a *CALM2* p.N98S mutation and differentiated into cardiomyocytes (CMs). *CALM*-LQTS iPSC-CMs exhibited prolonged action potential (AP) durations and impaired inactivation of L-type Ca2+ channel (LTCC) currents compared with control cells, consistent with clinical phenotypes. Notably, ablation of the mutant allele by gene editing rescued the electrophysiological abnormalities of LQTS-iPSC-CMs, indicating that the mutant allele caused dominant-negative suppression of LTCC inactivation, resulting in prolonged AP duration.

Regarding *CALM*-related CPVT, we established iPSCs from a boy carrying a novel *de novo* heterozygous variant, *CALM2* p.E46K, who was diagnosed with CPVT accompanied by neurodevelopmental disorders. In *CALM*-CPVT iPSC-CMs, we found more frequent abnormal electrical excitations and $Ca^{2+}$ waves than control lines in association with increased $Ca^{2+}$ leakage from the sarcoplasmic reticulum via RyR2. Furthermore, the [$^{3}$H] ryanodine binding assay revealed that E46K-CaM facilitated RyR2 function especially by activating at low [$Ca^{2+}$] levels. The real-time CaM-RyR2 binding analysis demonstrated that E46K-CaM had a tenfold increased RyR2 binding affinity compared to wild-type CaM which may account for the dominant effect of the mutant CaM. Finally, antiarrhythmic agents, nadolol and flecainide, suppressed abnormal $Ca^{2+}$ waves in E46K-CMs. Our findings in iPSC-based drug testing will contribute to precision medicine.

### [3S02a-4]

### Elucidation of the mechanisms of trastuzumab-induced severe cardiotoxicity using patient-derived iPSCs cardiomyocyte model

*Ritsuko Sasaki[1], Hidetaka Eguchi[2], Wado Akamatsu[3], Sakiko Miyazaki[4], Mitsue Saito[1] ([1]*Department of Breast Oncology, Juntendo university,* [2]*Diagnostics and Therapeutics of Intractable Diseases, Intractable Disease Research Center,* [3]*Center for Genomic and Regenerative Medicine, Juntendo University Graduate School of Medicine,* [4]*Department of Cardiovascular Biology and Medicine, Juntendo University Graduate School of Medicine*)

**Introduction** Human epidermal growth factor receptor 2 (HER2/ErbB2) is overexpressed in approximately 20% of invasive breast cancer and is associated with poor prognosis. Trastuzumab (Tmab), an anti-ErbB2 humanized monoclonal antibody, dramatically reduces cancer recurrence and mortality in patients with ErbB2-positive breast cancer. However, Tmab-induced cardiotoxicity interferes with continued treatment in approximately 10% of patients, but its mechanism has not been fully elucidated because relevant patient characteristics are not well understood. **Methods** From 468 breast cancer patients treated with Tmab, we selected three ones with severe-cardiotoxicity of left ventricular ejection fraction reduced by ≥30% from baseline (**SP**), and three non-cardiotoxic ones (**NP**) matched with the SP by various clinical factors. The iPS cells established from their peripheral blood sample were induced to cardiomyocytes. Using these cell models, we evaluated cell function in terms of cardiac contractility and energy metabolism pathways and performed RNA-Seq. **Results** Reduced contraction and relaxation velocities with trastuzumab were more evident in SP than in NP, indicating that the cardiotoxicity phenotype could be replicated. Differences in ATP production, ROS and autophagy activity were observed between the two groups, while there was no difference in mitochondrial oxygen consumption rate following trastuzumab treatment. To further explore the factors contributing to these results, RNA-Seq was performed on 12 samples of cell models from both groups, Tmab-treated and untreated. RNA sequencing analysis followed by quantitative real-time PCR revealed that in SP rather than NP, enhanced kallikrein-5 gene expression in the untreated group and an increase in inflammatory signaling pathways, such as interleukin-1*B*, after trastuzumab treatment. The expression and functional analysis of proteins showed that the kallikrein5-protease-activated receptor 2 (PAR2)-MAPK pathway was more activated in SP. Furthermore, treatment with a PAR2-antagonist suppressed interleukin-1*B* mRNA expression. **Conclusions** The biological characteristics of SP indicate a drug-sensitive individual with increased inflammatory signaling and vulnerability. The anti-inflammatory pathway against Tmab cardiotoxicity could be a new therapeutic strategy.

### [3S02a-5]

### Device development for fighting against heart failure pandemic

*Keita Saku[1] ([1]*Department of cardiovascular dynamics, National Cerabral and Cardiovasucular Center*)

Heart failure is a major medical challenge, particularly in the elderly. Despite the growing burden of heart failure (heart failure pandemic), there are limited solutions to slow its progression. Minimally invasive therapies, such as catheterization and implantable cardiac devices, have made it possible to treat elderly heart failure patients and improve their quality of life and prognosis. We are developing an innovative catheter-based device to modulate the autonomic nervous system. The autonomic nervous system plays a vital role in regulating cardiovascular function, and its dysregulation contributes to heart failure progression. The vagal nerve, which directly connects the brainstem to the thorax and abdomen, plays a crucial role in regulating the functions of multiple organs. It has already been clinically used as a device to treat epilepsy and has shown effectiveness in other neurological disorders. Additionally, acute vagal nerve stimulation (VNS) targeting the efferent pathway has been investigated and shown beneficial effects for several cardiovascular diseases in animal studies. Excessive tachycardia leads to increased myocardial oxygen consumption (MVO2) and worsened cardiac function in heart failure. VNS elicits a significant physiological response, reducing heart rate. We have recently developed an intravenous VNS (iVNS) catheter called JOHAKU. In a canine model of acute heart failure, the iVNS catheter effectively attenuated heart rate and MVO2 without exacerbating hemodynamics. To develop these therapies for elderly heart failure patients, we need to consider the increased medical costs, comorbidities, frailty, and the patient's or family's will, as well as other social problems associated with the heart failure pandemic. In this session, we will review the autonomic characteristics of heart failure and new treatment strategies using medical devices.

### Symposium

[3S03a]

**Physiological action and clinical application of functional sugar “rare sugar Allulose"**

March 30, 14:20 - 16:20, Room 3

### [3S03a-1]

### Molecular and quantitative analysis of D-allulose transport by glucose transporters

*Kazuyo Kamitori[1,3], Susumu Mochizuki[2,3], Ken Izumori[2,3], Kazuya Akimitsu[2,3], Yuichiro Fujiwara[1,3,4] ([1]*Laboratory of Molecular Physiology and Biophysics, Faculty of Medicine, Kagawa University,* [2]*Faculty of Agriculture, Kagawa University,* [3]*International institute of Rare Sugar Research and Education, Kagawa University,* [4]*Physiology and Biophysics, Graduate School of Biomedical and Health Sciences, Hiroshima University*)

D-allulose is a ketohexose classified as rare sugars. Increasing reports have showed the usefulness of this sugar in the healthcare field. Its versatile physiological activities suggest that it is transported into cells in living organisms, and modify the dynamics of physiologically important sugars such as D-glucose and D-fructose. Meanwhile molecules responsible for D-allulose transport have not been clearly identified, because sugar transport analyses have largely depended on RI-labelled molecules, which is hard to obtain for rare sugars. In this presentation, we will introduce our recently developed non-RI method which quantitatively analyses the transport capability of each transporter for each sugar. Using this approach, we could analyze the transport profile of D-allulose as well as other rare sugars.

As a first step, transport profile of D- and L-ketohexoses by human GLUT2 and GLUT5. These transporters are expressed on the basolateral and apical sides of intestinal epithelial cells, respectively, and transport different sugars, attracting attention as drug targets for sugar metabolism. We expressed GLUT2 or GLUT5 in Xenopus laevis oocytes, then they were treated with each sugar and the transported sugar was quantitatively analyzed using HPLC. As a result, GLUT2 showed low selectivity, transporting several sugars, D-tagatose, D-fructose, L-allulose, L-sorbose, and D-allulose, with D-tagatose at the highest level. GLUT5 showed high specificity, transporting the most D-fructose, a known substrate, and also D-allulose. Next, we analyzed the competitive effect of D-allulose on the D-fructose transport by GLUT5. The result showed that D-fructose transport was inhibited by D-allulose in GLUT5-expressing oocytes. Other transporters examined in this study showed low transport capability for D-allulose. Overall, GLUT5 is one of the major transporter for D-allulose, and also a target molecule concerning the anti-hyperglycemic effects of this rare sugar.

Further application of this strategy for other transporters would lead to the comprehensive understandings of rare sugar dynamics in human body and in various environments.

### [3S03a-2]

### D-Allulose suppress feeding and body weight by reciprocally regulating satiety and appetite neurons in the hypothalamic arcuate nucleus in mice

*Yermek Rakhat[1,2], Daisuke Yabe[2,3,4], Yutaka Seino[3], Toshihiko Yada[1,2,4] ([1]*Center for Integrative Physiology Division of Integrative Physiology, Kansai Electric Power Medical Research Institute, Kyoto, Japan,* [2]*Department of Diabetes, Endocrinology and Metabolism/ Rheumatology and Clinical Immunology, Gifu University Graduate School of Medicine, Gifu, Japan,* [3]*Yutaka Seino Distinguished Center for Diabetes Research, Kansai Electric Power Medical Research Institute, Osaka, Japan,* [4]*Center for One Medicine Innovative Research, Gifu University Institute for Advanced Study, Gifu, Japan*)

D-Allulose, a zero-calorie rare sugar sweetener, decreases blood glucose, food intake and body weight, thereby counteracting type 2 diabetes and obesity. Regarding the underlying mechanism, we previously reported that D-Allulose stimulates secretion of glucagon like peptide-1 (GLP-1) from the intestine, which in turn activates vagal afferent nerves. D-Allulose has been considered not to pass through blood-brain-barrier in a substantial amount. Tanycytes constitute a sub-population of glial cells laying out the third ventricle. Recent findings revealed the new potency of Tanycytes to transport relatively large molecules such as leptin, insulin and GLP-1 receptor agonist from circulation to the brain. This prompted us to hypothesize that oral D-Allulose is transported to the hypothalamic arcuate nucleus (ARC) neurons and regulates feeding and metabolism. In this study, we examined the effect of D-Allulose on the ARC neurons that produce satiety or appetite, and the effect of intracerebroventricular injection (icv) of D-Allulose on feeding in mice. We found that, icv injected D-Allulose significantly reduced cumulative food intake for 1~4 hours after injection in mice. D-Allulose at doses around 1 mM directly interacted with neurons isolated from ARC and increased $[Ca^{2+}]_i$ in the anorectic neurons that express pro-opiomelanocortin (POMC) and that respond to GLP-1. Furthermore, D-Allulose attenuated $[Ca^{2+}]_i$ increases in the orexigenic ARC neurons that respond to ghrelin and low glucose and that express neuropeptide Y (NPY) . These results show that D-Allulose directly activates anorectic and inhibits orexigenic neurons in the ARC. This reciprocal regulation of satiety and appetite neurons may underly the outstanding ability of D-Allulose to regulate feeding, energy and glucose metabolism. The property of D-Allulose to evoke both the tanycyte-ARC pathway and GLP-1 release - vagal afferent pathway represents D-Allulose as a promising compound for regulating feeding and metabolism to ameliorate obesity and diabetes in humans.

### [3S03a-3]

### Secretory mechanisms of GLP-1 by D-Allulose

*Tohru Hira[1] ([1]*Hokkaido University*)

Enteroendocrine cells, scattered (approximately 1%) in the gastrointestinal epithelium, sense various information in the gastrointestinal lumen and release various gastrointestinal hormones to the basorateral side. Our research focuses on the mechanism by which enteroendocrine cells sense nutrients and food components. Glucagon-like peptide-1 (GLP-1) is a gastrointestinal hormone that enhances insulin secretion in a plasma glucose level-dependent manner. It is also called “incretin”. GLP-1 producing cells (so called L cells) are abundant in the ileum and large intestine. GLP-1 secretion increases after meals. Glucose, fructose, long-chain fatty acids, short-chain fatty acids, peptides, and some amino acids are known to promote GLP-1 secretion. D-allulose (allulose), an isomer of fructose, is one of the rare sugars that exists in small amounts in nature, and is known to lower plasma glucose levels and fat accumulation. In animal studies, we found that a single oral administration of allulose strongly stimulates GLP-1 secretion, but did not stimulate glucose-dependent insulinotropic polypeptide (GIP). An administration of allulose into the small intestine induced GLP-1 secretion, but intraperitoneal administration did not, suggesting that it stimulated GLP-1 secretion from the luminal side. We observed that the small intestine remained expanded after allulose administration. Since allulose is poorly absorbed (approximately 40% in rats and 60% in humans), it was thought that it remained along with water in the lumen to maintain osmotic pressure. It is possible that such stimulation of intestinal expansion is related to the GLP-1 secretion promoting effect of allulose.

### [3S03a-4]

### Multiple beneficial functions of D-allulose against hyperphagic obesity and diabetes via GLP-1 release and its activation of vagal afferents

*Yusaku Iwasaki[1] ([1]*Laboratory of Animal Science, Graduate School of Life and Environmental Sciences, Kyoto Prefectural University*)

Glucagon-like peptide-1 (GLP-1) is an intestinal hormone that has several beneficial effects such as regulating food intake and blood glucose levels through insulin secretion. The primary triggers for GLP-1 secretion are nutrients with calories, including carbohydrates, proteins, and fats. Interestingly, we have previously identified the rare sugar D-allulose (Allu) as a potent GLP-1 secretagogue, even though it is a zero-calorie sweetener. This discovery is significantly advancing our understanding of the physiological functions and mechanisms of endogenous GLP-1. Unlike the stable GLP-1 receptor agonists used in diabetes treatment, endogenous GLP-1 is unstable in vivo. However, when GLP-1 is released, it rapidly activates vagal afferent nerves distributed near the GLP-1-secreting cells. This neural information is then conveyed to the brain, thereby ameliorating hyperphagic obesity and diabetes. In this symposium, I will present the multiple beneficial functions of D-allulose, including its role in regulating feeding, glucose metabolism, and energy metabolism through GLP-1 release and its activation of vagal afferent nerves.

### [3S03a-5]

### What does allulose bring to diabetes treatment?

*Toshihiro Kobayashi[1], Kensaku Fukunaga[1], Koji Murao[1] ([1]*Department of Endocrinology and Metabolism, Faculty of Medicine, Kagawa University*)

Studies on human have revealed that D-allulose improves glucose metabolism and postprandial blood glucose in non-diabetic healthy people, and also has an anti-obesity effect by reducing body weight. These effects are also expected in diabetic patients, and attention has recently focused on effectively utilizing rare sugars in dietary therapy, which is considered to be the most basic and important part of diabetes treatment. In our study, we aimed to develop a diabetes therapeutic diet that better suppresses postprandial hyperglycemia for patients with type 2 diabetes, and examined whether a therapeutic diet containing D-allulose suppresses postprandial hyperglycemia. In type 2 diabetes patients between 20 and 80 years of age who were undergoing inpatient treatment at the Department of Endocrinology and Metabolism, Kagawa University Hospital, postprandial blood glucose changes were evaluated using an intermittently scanned continuous glucose monitoring (isCGM) during two days of consumption of a normal diabetes therapeutic diet and two days of consumption of a diabetes therapeutic diet containing D-allulose. The results showed that the diabetes therapeutic diet containing D-allulose tended to suppress postprandial hyperglycemia compared to the normal diabetes therapeutic diet. No adverse events, such as diarrhea, which are thought to be caused by D-allulose intake, were observed in this study. There has been no study on the effect of the diabetes therapeutic diet containing D-allulose on suppressing postprandial hyperglycemia in type 2 diabetes patients, and we believe that the results of this study may make rare sugars a new option for dietary therapy of diabetes patients.
Dietary therapy is an important part of treatment in early diabetes, but it requires patients to limit the amount of carbohydrate-rich foods and food intake, which is not an easy task to sustain over the long term. D-allulose is already used in commercial sweeteners and confectionery, and has the potential to be applied to a variety of foods in the future. If the use of seasonings and foods with D-allulose can change the "painful" dietary therapy to the "tasty and enjoyable" dietary therapy, diabetes patients may be able to engage in treatment more positively.

### Symposium

[3S04a]

**Physiology of neural organoids: How close to CNS**

March 30, 14:20 - 16:20, Room 4

### [3S04a-1]

### Recapitulation and investigation of human brain development with neural organoids

*Keiko Muguruma[1] ([1]*Kansai Medical University*)

### [3S04a-2]

### Emergence of neural region and function uncovered by cerebral and hippocampal organoids

*Hideya Sakaguchi[1] ([1]*RIKEN Center for Biosystems Dynamics Research*)

### [3S04a-3]

### Constructive understanding of cortical neural circuits using organoid technology

*Fumitaka Osakada[1,2] ([1]*Laboratory of Cellular Pharmacology, Graduate School of Pharmaceutical Sciences, Nagoya University*, [2]*Laboratory of Neural Information Processing, Institute for Advanced Research, Nagoya University*)

The function of the cerebral cortex is based on the integration of bottom-up inputs from lower-order areas, top-down inputs from higher-order areas, and local inputs within areas. Recent single-cell RNA-sequencing and connectivity analyses in the mouse cortex have revealed a diversity of cell types, each with unique connectivity. How do cortical neurons form long-range projections and local circuits to organize cortical functions? One potential approach to address this question is to construct cortical neural circuits *in vitro*. We directed pluripotent stem cells to generate neural organoids with different regional properties and fused them to recapitulate the interaction between regions. The fused organoids are called assembloids. I will talk about cortical assembloids with features of key cortical events and functions.

### [3S04a-4]

### Hypothalamic-Pituitary Axis in organoids

*Hidetaka Suga[1], Ryusaku Matsumoto[2], Hajime Ozaki[1], Tsutomu Miwata[1], Hiroshi Arima[1] ([1]*Nagoya University Graduate School of Meidicine,* [2]*Kyoto University*)

The hypothalamic-pituitary system is essential for maintaining homeostasis and life through the control of systemic hormones. We have established techniques to generate functional adenohypophysis and hypothalamus from human pluripotent stem cells. Pluripotent stem cells (PSC), such as embryonic stem (ES) cells and induced pluripotent stem (iPS) cells, differentiate into neuroectodermal progenitors when cultured as three-dimensional floating aggregates under serum-free conditions. Recent results have shown that strict depletion of exogenous patterning factors during the early differentiation period induces efficient generation of rostral hypothalamic-like progenitors, i.e. the most rostral and most ventral position in the cerebral nervous system. As a result of recapitulating such a condition in vitro, the PSC-derived hypothalamic-like progenitors generated rostral-dorsal hypothalamic neurons, in particular magnocellular vasopressinergic neurons, which release hormones upon stimulation.
We were then able to induce both ventral hypothalamic and oral ectodermal tissues simultaneously. Self-organisation of Rathke's pouch, the pituitary primordium, occurred at the interface of the two epithelia in vitro. After prolonged culture, the Rathke's pouch-like structures gave rise to various endocrine cells, including corticotrophs and somatotrophs. The induced corticotrophs efficiently secreted adrenocorticotropic hormone (ACTH) in response to corticotropin-releasing hormone (CRH). In addition, we found that in vitro generated corticotrophs were able to rescue hormone levels, physical activity and survival when transplanted into pituitary-resected hypopituitary mice. We have thus developed a useful method for the production of functional human pituitary tissue.
Our culture methods described above are characterised by the recapitulation of stepwise embryonic differentiation. Therefore, these methods could potentially be used as developmental and disease models, as well as for future regenerative medicine. We will present our recent efforts to establish an in vitro disease model using disease-specific iPS cells.

### [3S04a-5]

### Modeling hypothalamic development with neural organoids

*Yu Kodani[1] ([1]*Department of Physiology, Fujita Health University School of Medicine*)

The hypothalamus is an essential component of the endocrine and autonomic nervous system, and it also regulates motivated behaviors and the cognitive function. The multifunctional nature of the hypothalamus is conferred by diverse neuropeptidergic neurons localized in hypothalamic nuclei and subregions. Another key player is tanycytes, an ependymal cell population lining the third ventricle that modifies the functions of the hypothalamus through various means, including adult neurogenesis. Recent single-cell transcriptomic analyses have revealed molecular profiles of hypothalamic cell groups and gained insights into their developmental trajectories, but it remains largely unknown how specific neurons or tanycytes generate and mature in the developing hypothalamus. Neural organoid technology, utilizing embryonic stem cells (ESCs) or induced pluripotent stem cells (iPSCs), continues to advance as a versatile tool for modeling the development and pathology of different brain regions. Several studies have reported the generation of hypothalamic organoids (HOs) from ESCs/iPSCs; however, the usefulness of these organoids as a research model has not been well defined. To better understand the similarity of HOs to the native hypothalamus, we characterized several aspects of mouse ESC-derived HOs: (1) the temporal pattern of neuronal and tanycytic differentiation, (2) neurochemical and physiological properties of neuropeptidergic neurons, and (3) axonal projections of HO-derived neurons after transplantation into the rodent brain. We provide evidence that neurons and tanycytes generated in HOs exhibit developmental and phenotypic features comparable to their *in vivo* counterparts. Our results suggest that neural organoids offer a promising approach to study the ontogeny and physiology of hypothalamic cell types.

### Symposium

[3S05a]
Cooperation with Other Societies Committee
**Role of calcium signaling in cell proliferation and related diseases**

March 30, 14:20 - 16:20, Room 5

### [3S05a-1]

### Regulation of cancer cell proliferation by calcium signaling

*Midori Shimada[1], Yuki Sato[2], Makoto Habara[2], Shunsuke Hanaki[2], Takahiro Masaki[2], Haruki Tomiyasu[2], Yosei Miki[2] ([1]*Nagoya University,* [2]*Yamaguchi University*)

Intracellular calcium ions ($Ca^{2+}$) act as diverse second messengers in signal transduction pathways and are involved in various biological phenomena such as gene expression, cell motility, apoptosis, and the cell cycle. Intracellular $Ca^{2+}$ increases most during the G1/S phase of the cell cycle, but the significance of this increase has not been well understood. To elucidate the effect of intracellular $Ca^{2+}$ on cancer cell growth, RNA sequencing data were analyzed when the concentration of intracellular $Ca^{2+}$ was reduced by amlodipine treatment. We found that a decrease in intracellular $Ca^{2+}$ promotes proteolysis of E2F1 and c-Myc, which contribute to malignant transformation of cancer, and decreases expression of their target genes and inhibits cell proliferation. In a reciprocal manner, increasing intracellular $Ca^{2+}$ by calcium ionophore treatment led to stabilization of E2F1 and c-Myc proteins. We further found that calcineurin- a phosphatase that mediates $Ca^{2+}$ signaling-dephosphorylates E2F1 and c-Myc and decreases binding of these proteins to the FBXW7 ubiquitin ligase. Calcineurin, therefore, inhibits ubiquitination and stabilizes both E2F1 and c-Myc. These results are in line with our previous report that calcineurin stabilizes and activates estrogen receptor alpha in breast cancer and that elevated expression of calcineurin is associated with a higher recurrence rate after endocrine therapy and a poorer prognosis. Dysregulation of $Ca^{2+}$ signaling is thought to be involved in tumor development, progression, and metastasis. Degradation of E2F1 and c-Myc, two key players involved in malignant transformation of cancer, by $Ca^{2+}$ signaling therefore opens up the possibility that calcineurin inhibition as a therapeutic strategy for cancers with high expression of E2F1 and c-Myc.

### [3S05a-2]

### The significance of hypoxia-sensitive TRPA1 channel in life

*Nobuaki Takakahashi[1] ([1]*Kyoto University*)

The atmospheric partial pressure of $O_2$, which is vital for aerobic organisms, has fluctuated greatly during the 4.6-billion-year history of Earth. While it has long been recognized that the $O_2$ fluctuation imposed selective pressure on organisms and contributed greatly to major evolutionary events, little is known about the evolution of $O_2$ sensing mechanisms in life. Here, our molecular evolutionary study on TRPA1, a hypoxia-sensitive channel, reveals that the genetic fixation of the hypoxia sensitivity in TRPA1 arose with the emergence of advanced placenta. We found that TRPA1 plays an essential role in fetal survival in maternal anemia by ensuring $O_2$ supply to the fetus. Mechanistically, TRPA1 increases trophoblast giant cells (TGCs), which are central to vascular remodeling in placenta, in response to hypoxic stress by inducing the differentiation of trophoblast stem cells. TRPA1 activation by hypoxic stress induces $Ca^{2+}$ influx that activates ERK1/2-p53 signaling axis and in turn promotes the differentiation into TGC. Anemia represents an "Achilles heel" of viviparity as this system requires lots of blood and the $O_2$-transport protein hemoglobin. Our findings suggest that there was a selection of mammals that have the hypoxia sensitivity of TRPA1 in order to leave offspring even in anemia, and would provide critical and fundamental insights into the evolution of mammals and perinatology.

### [3S05a-3]

### $Ca^{2+}$-mediated ER-mitochondria crosstalk during plasma membrane damage-dependent senescence

Kojiro Suda[1], Yohsuke Moriyama[1], *Keiko Kono[1] ([1]*Okinawa Institute of Science and Technology Graduate University*)

Cellular senescence is a stable cell cycle arrest that contributes to a variety of physiological and pathological processes in vivo, including organismal aging, wound healing, and cancer. Accumulating evidence suggests that elimination of senescent cells ameliorates age-related pathologies. In vitro, various stresses, including oxidative stress, oncogene activation, telomere shortening, and DNA damage, induce cellular senescence via the DNA damage response. However, the physiological triggers of cellular senescence remain controversial. Here, we show that cellular senescence is induced by physiological plasma membrane damaging stimuli such as pore-forming toxins and mechanical injury in normal human fibroblasts in vitro. We found that $Ca^{2+}$ influx following plasma membrane damage is necessary and sufficient for the induction of plasma membrane damage-dependent senescence. Live cell imaging revealed that $Ca^{2+}$ entering the cytosol is immediately incorporated by the endoplasmic reticulum (ER). Subsequently, mitochondrial $Ca^{2+}$ levels rise steadily, suggesting $Ca^{2+}$ transport from the ER to the mitochondria via the contact sites. We observed an increase in mitochondrial oxidative stress, and attenuation of oxidative stress with antioxidants suppressed plasma membrane damage-dependent senescence. These results suggest that mitochondrial dysfunction due to $Ca^{2+}$ accumulation induces plasma membrane damage-dependent senescence. We also found that $Ca^{2+}$ transport from the ER to the mitochondria is necessary to maintain cytosolic $Ca^{2+}$ levels and cell survival after plasma membrane damage. Using a proteomic approach, we identified the proteins that mediate the ER-mitochondria contact. This study highlights an underappreciated subtype of cellular senescence, plasma membrane damage-dependent senescence, and provides mechanistic insights into $Ca^{2+}$-dependent senescence induction.

### [3S05a-4]

### Role of $Ca^{2+}$-permeable mechanosensitive ion channels in skeletal muscle regeneration

Kotaro Hirano[1], *Yuji Hara[1] ([1]*Univ. of Shizuoka*)

Skeletal muscle myofibers possess high regenerative capacity in response to muscle damages. Muscle-resident stem cells called muscle satellite cells (MuSCs) play a critical role in myofiber regeneration to maintain tissue homeostasis in skeletal muscle. Calcium ion ($Ca^{2+}$) has been long thought to be involved in MuSC functions, but the molecular entity of ion channels that conduct $Ca^{2+}$ required for myofiber regeneration remains to be elucidated. Here we identify PIEZO1, a $Ca^{2+}$-permeable mechanosensitive cation channel that is activated by membrane tension, as a critical determinant for muscle regeneration. Fluorometric $Ca^{2+}$ imaging detected PIEZO1-dependent $Ca^{2+}$ fluctuation in freshly isolated MuSCs. Using a series of genetic mouse models, we revealed that myofiber regeneration after muscle injury was significantly delayed in MuSC-specific *Piezo1*-deficient mice, at least partly because of mitotic defects of undifferentiated MuSCs including the presence of chromosomal bridges and micronuclei. Moreover, pharmacological studies showed that the cell division defects in *Piezo1*-deficient MuSCs could be restored by Rho activation. Collectively, PIEZO1 plays a role in muscle regeneration by controlling cell division of MuSCs in a Rho-dependent manner, suggesting that $Ca^{2+}$ influx through the mechanosensing machinery is central to the maintenance of muscle homeostasis.
In this session, we will also present our unpublished data showing that a series of mechanosensitive ion channels may orchestrate muscle regeneration.

### [3S05a-5]

### Cardiocirculatory regulation by receptor-operated $Ca^{2+}$ -permeable channel TRPC6

*Kazuhiro Nishiyama[1], Motohiro Nishida[2,3] ([1]*Osaka Metropolitan University Graduate School of Veterinary Science,* [2]*Graduate School of Pharmaceutical Sciences, Kyushu University,* [3]*National Institute for Physiological Sciences (NIPS), National Institutes of Natural Sciences (NINS)*)

Transient receptor potential canonical (TRPC) subfamily proteins are the molecular entity of receptor-activated $Ca^{2+}$-permeable channels in vertebrates. Especially, diacylglycerol-sensitive TRPC members, TRPC3 and TRPC6, reportedly contribute to receptor-stimulated cardiovascular remodeling. However, the physiological role of TRPC6 in blood vessels and the heart remains unclear. In this presentation, we would like to introduce two topics related to TRPC6 that we recently discovered.
1, We found that TRPC6-knockout mice have better blood flow recovery after hindlimb ischemia than wild type mice. Treatment of TRPC6 inhibitor improved post-ischemic peripheral blood circulation.
2, We found that TRPC6-mediated $Zn^{2+}$ influx with α1adrenergic receptor stimulation enhances baroreflex-induced positive inotropy. Treatment of TRPC6 activator prevented chronic heart failure progression in mice.
In these studies, we show that modulation of TRPC6 is an excellent therapeutic target for several diseases.

### Symposium

### [3S06a]
**Physiology Education Starting with "Why"- Education that changes in the era of post-corona and digital transformation-**

March 30, 14:20 - 16:20, Room 6

### [3S06a-1]
**Online physiology practical training using Zoom**

*Michio Shiibashi[1], Hiromasa Satoh[1] ([1]*Saitama Medical University*)

### [3S06a-2]
**Do you insist on continuing your own lectures?**

*SUSUMU MINAMISAWA[1] ([1]*The Jikei Univ. Cell Physiology*)

Our education is already undergoing major changes with the introduction of digital transformation, but COVID-19 spurred it on.Particularly in the field of higher education, where face-to-face teaching had been banned and alternatives had to be considered, almost all higher education institutions introduced online and/or on-demand education.Physiology education was no exception, and not only lectures but also practical training had to be conducted remotely.Now that COVID-19 has come to a close and face-to-face teaching is available, many higher education institutions appear to be returning to their previous teaching styles, although some universities are continuing to transform their teaching styles in the wake of COVID-19.To give one example, The Jikei University, to which I belong, has cancelled all face-to-face lectures in basic medicine and replaced them with asynchronous, on-demand lectures.In addition, the number of lecture sessions was reduced by 10-20% and new synchronous small group exercise courses were introduced instead. As a result, the students' assessment of their knowledge acquisition did not change after the cessation of face-to-face teaching, at least as judged by the examination results.A major change is that the educational content of each teacher is now easily visible among teachers. This is also the case in open education, which is now rapidly becoming popular.It would be a shame to keep excellent on-demand lectures within a single university.If there were on-demand lectures that promoted knowledge acquisition more effectively than I do, and students had free access to them, I have to wonder where the point is for me to provide on-demand content by myself.If lectures could be left to virtual 'best teachers', teachers could spend more time with their students and get them to think more deeply about life phenomena. Open education has such potential.

### [3S06a-3]
**Online-based TBL for Indonesian/Japanese medical students**

*Noriyuki Koibuchi[1] ([1]*Department of Integrative Physiology, Gunma University Graduate School of Medicine*)

Every year, we conduct online-based team-based learning (TBL) for medical students of Sriwijaya University, Indonesia, using clinical case scenario. Sometimes medical students at Gunma University are invited to attend the TBL. The exact topic of TBL is not announced. They are only informed that the topic is from a specific area (e.g., Endocrine Physiology). Before the TBL, a coordinator (Koibuchi) and teachers at Sriwijaya University usually have one or two online meetings to discuss how to manage the class. On the day of the TBL, students (approximately 100 students) are grouped 5-6 students/group). Japanese students are assigned to the group whose members can speak fluent English.
In the group whose members cannot speak or understand English very well, teachers are assigned to support them. Before the case presentation, approximately 10-15 multiple chose questions are given to confirm students' readiness (individual readiness assurance test. IRAT). Then, using breakout room, students can discuss the questions to find out the correct answer (team readiness assurance test, TRAT) followed by submission of the group answer using "Chat". Groups that submitted wrong answer should explain the reason why they selected the answer. After the IRAT and TRAT, clinical case scenario is presented with several questions. All questions are related to physiological phenomenon. For example, in case of "Type 1 diabetes mellitus" is used, representative questions are "Why did this patient urinate so frequently?" and "Why did ketone body level increase?". Students should discuss in the group to answer the question. Then, students get together and coordinator pick up students randomly and ask to answer questions, based on their group discussion. After the session, a summary and small lecture are given by coordinator and the session is closed. After the class, survey questions are given. At present the TBL has been conducted 3 consecutive years. Based on the answer of the survey question, students seem to be enjoying the TBL, mainly because they feel that knowledge retention is better in TBL class than in standard lectures. Another important point of this international TBL is the development of friendship between Japanese and Indonesian students, which can be constructed without visiting each country.

### [3S06a-4]

### The Physiology MOOC in China: Construction and Application

*Ziqiang Luo[1], Dandan Feng[1] ([1]*Dept of Physiology, Xiangya Medical School, Central South University, Changsha City, Hunan Province, China*)

Massive open online courses (MOOCs) are a novel and emerging mode of online learning which has the advantage of free access to the public without time and space limit. MOOCs are becoming the one of the main ways to implement the innovations of modern education. The construction of MOOCs in China began in 2013, and developed rapidly in 2015 with the encouragement of the Ministry of Education of China. The major MOOC platforms in earlier time including "China University MOOC" and "Xuetang X ", and in March 2022, the Official Public Service Platform named “Smart Education of China · Higher Education” was launched, 27,000 high-quality courses were selected from over 52,000 higher education MOOCs in China, and these courses are running with all resources free to the public.
The first MOOC of Physiology in Chinese Medical College was launched in 2016, and 45 MOOCs of Physiology from Medical Colleges have been running in China at present, while only 8 high-quality Physiology courses have been recognized as First-class Online Open Courses by Ministry of Education of China. To facilitate the development of higher education, Ministry of Education of China actively promotes Universities to integrate MOOC content into the regular curriculum creating the blended learning programs, and selects the First-class National Blended Teaching Courses to inspire the reforming and revolution of teaching and learning in China. The Physiology MOOC at Central South University (CSU) was firstly launched in April 2017 and has completed 14 rounds of online teaching. The number of registered social learners has reached more than 350,000, ranking first among similar courses in China, and it has been selected as SPOC applied in their teaching by more than 70 Medical Colleges in China. Aim to share the Chinese experience in Physiology MOOC construction and application to international colleagues, we take the MOOC of Physiology at CSU as an example to introduce the design and construction of Physiology MOOC in Chinese Medical Colleges, and the application of the MOOC in teaching, such as implement the flipped classroom by using MOOC.

### [3S06a-5]

### Digital innovations shape the activities of the IUPS and American Physiological Society

*Robert Graham Carroll[1] ([1]*Brody School of Medicine at East Carolina University*)

The educational landscape usually changes by evolution rather than revolution. The Covid-19 pandemic forced a revolutionary change in teaching, as an estimated decade of educational innovation was compressed into a few months. Innovative approaches to teaching and learning, such as remote laboratory sessions and remote assessment of learning, moved rapidly from the “pilot study” phase to global implementation. As the world emerges from Covid restrictions, professional societies resume their traditional roles of identifying the educational innovations, evaluating educational outcomes and training members to implement those approaches that have proven successful. The American Physiological Society launched the Center for Physiology Education in 2021, bringing together curriculum development, physiology education research and evidence-based teaching practice. As an example, the Core Concepts in Physiology project is redefining exactly what is physiology, guiding instruction at all levels. These activities augment the research base expanded by publications in the journal Advances in Physiology Education. The pandemic also normalized online instruction and meetings. The financial and time costs of meeting travel were diminished or eliminated by presenting meetings online. Global participation was as easy as participation from across town. Education conferences tied to the IUPS embraced these opportunities and regularly featured international speakers. The 2022 IUPS congress in Beijing 2022 was held online, as was the Education Workshop. The waning pandemic allows a deeper exploration of the cost/benefit ratio of live vs. virtual conference attendance. Contrasting the pandemic experience with the post-pandemic experience will allow a stronger appreciation of the value of interpersonal interactions. In summary, the post-pandemic educational environment needs to be shaped by the activities and events that have improved the teaching and learning of physiology. Professional societies need to play a central role in evaluating the outcomes of these changes, and prepare their members to adopt those shown to be successful.

### Symposium

[3S08a]
**Multidimensional monitoring of physiological function and behavior using virtual reality**

March 30, 14:20 - 16:20, Room 8

### [3S08a-1]

### A distributed and efficient population code of mixed selectivity neurons for flexible navigation decisions

*Shinichiro Kira[1], Houman Safaai[1,2], Ari Morcos[1], Stefano Panzeri[2,3], Christopher Harvey[1] ([1]*Harvard Medical School,* [2]*Istituto Italiano di Tecnologia,* [3]*University Medical Center Hamburg-Eppendorf (UKE)*)

Flexible behavior often requires rapid switching of associations between sensory cues and actions based on behavioral objectives stored in memory. A key computation for such rapid switching is the integration of sensory signals with short-term memory. Here, we aimed to reveal the cortical areas and neural mechanisms central to this integration, and thus flexible decision-making, during spatial navigation. We trained mice to make flexible decisions in a delayed match-to-sample (DMS) task in a virtual reality T-maze. As a mouse navigated through the maze, it sequentially observed two cues separated by a short maze segment, which created 1-2 s of delay between the cues. Mice switched, on a trial-to-trial basis, their navigation toward or away from a second visual cue depending on its match to the remembered first cue. To systematically screen for cortical areas that are involved in flexible navigation decisions, we bilaterally inhibited different sites across the dorsal cortical surface by optogenetically activating inhibitory neurons in VGAT-ChR2 mice. Inhibition of V1, posterior parietal cortex (PPC), or retrosplenial cortex (RSC) induced a large decrease in the task performance. Two-photon calcium imaging revealed neurons that can mediate rapid sensorimotor switching by encoding a conjunction of a current and remembered visual cue that predicted the mouse's navigational choice from trial to trial. These mixed selectivity neurons formed efficient population codes that appeared to guide accurate decision-making because they were informative before correct choices but degenerated during errors. Surprisingly, these neurons were distributed across posterior cortex, even V1, but were densest in RSC and sparsest in PPC. The mixed selectivity neurons were rare in naïve mice that ran through the identical maze, indicating that these neurons emerged through learning of the DMS task. Together, we propose that the flexibility of navigation decisions arises from neurons that develop mixed selectivity over learning to integrate visual and memory information within a visual-parietal-retrosplenial network, centered in RSC.

### [3S08a-2]

### Mechanisms of hippocampal cognitive map formation and plasticity

*Masaaki Sato[1] ([1]*Department of Neuropharmacology, Hokkaido University Graduate School of Medicine*)

Virtual reality (VR) is a computer-generated environment that can reproduce a “world” to be experienced interactively. Head-fixed VR environments can be combined with large-scale, high-resolution brain activity measurements such as multiphoton calcium imaging, offering the advantage of precise and flexible control of experimental conditions. VR is thus becoming increasingly important in behavioral experiments using mice. Hippocampal pyramidal neurons are involved in a dynamic cellular ensemble code for space and memory, and salient features of the environment are represented by an increased density of relevant place cells. However, the cellular mechanisms for the establishment and reorganization of such disproportionated maps remained unclear. By combining VR with two-photon calcium imaging, we have examined the formation and plasticity of hippocampal CA1 place cell maps in mice undergoing spatial learning. We chronically imaged the deep CA1 sublayer of Thy1-G-CaMP7 transgenic mice during training on a virtual linear track. In this task, landmark and reward were associated with two distinct locations on the track created in the virtual environment. Representations of landmark and reward emerged with experience over rapid and delayed time courses, respectively. While the formation of overrepresented maps was dominated by the selective stabilization of place fields encoding salient locations, the reorganization of pre-established maps by the relocation of reward occurred through the cooperation of de novo formation, lateral shifts, and selective stabilization of place fields. The overrepresentation of landmark was dependent on Shank2, a postsynaptic scaffold protein encoded by an autism spectrum disorder-associated gene, but that of reward was not. Collectively, these findings reveal that multiple distinct dynamics of place fields are involved in the formation and plasticity of hippocampal cognitive maps and provide a mechanism by which the experience of salient environmental features can form lasting yet adaptive memory traces.

### [3S08a-3]

### A mouse VR system for monitoring cortical functional network dynamics during behavior

*Nobuhiro Nakai[1] ([1]*Kobe University*)

The cerebral cortex is an important brain region that plays a crucial role in integrating sensory information from the external environment and the body, as well as in generating appropriate behavioral outputs. In the cerebral cortex, complex information processing is carried out through the cooperative activity of various functional areas. However, the overall brain network of this process remains unclear. To analyze the wide-ranging network activity of the cerebral cortex during spontaneous behavior in mice, we developed an experimental platform that combines wide-field calcium imaging with virtual reality (VR). In this presentation, I will visualize the cooperative activity between cortical areas as a functional network and introduce how network patterns are rapidly reorganized in response to changes in behavioral states, as well as differences in cortical network activity depending on visual information. Furthermore, in the analysis using a mouse model of autism, I revealed autism-specific abnormalities in the cortical network during the initiation and cessation of locomotion. For future research directions, I would like to discuss the potential of using the VR system to study brain functional network dynamics related to social behavior, the possibility of predicting behavior from brain information using machine learning, and the development of new approaches for the diagnosis and treatment of autism.

### [3S08a-4]

### Measurement and manipulation of the "forward-looking" mind using multidimensional assessments of the body during walking in a virtual reality environment

*Takahiro Hirao[1,6], Mitsuhiro Miyamae[1], Kazuma Mori[1,8], Yoshiko Yabe[1,9], Kokono Terada[1,4], Hiroaki Hamada[3,1,7], Natsuki Sado[2], Tempei Tominaga[2], Makiko Yamada[1,4,5]
(*[1]National Institutes for Quantum Science and Technology, [2]University of Tsukuba, [3]Araya Inc., [4]Chiba university, [5]Tohoku university, [6]Waseda university, [7]Okinawa Institute of Science and Technology Graduate University, [8]Osaka university, [9]Kochi University of Technology*)

While there have been research reports on the relationship between dysphoric mood and gait, such as depressed individuals having a characteristic gait (e.g., Michalak et al., 2009; Adolph et al., 2021), there are few research findings on positive mood and gait. In particular, it is not well known how individual differences in positive personality traits and state changes in positive mood possessed by healthy individuals are manifested in the body during walking. The relationship between gait and the forward-looking mind itself, as well as other mentally positive states of mind generated by the forward-looking mind, is the focus of our research.
To obtain research findings that can be applied to the real world, it is necessary to conduct gait experiments under conditions that are as close as possible to those experienced in daily life. However, gait measurements in the laboratory are often far removed from the real-world environment. For example, physical gait measurement experiments often use a treadmill set up in the laboratory, but this does not include visual changes associated with walking, which occur in a daily life situation. Therefore, virtual reality (VR) technology for human research may be important to bridge the gap between the laboratory and the real-world environment.
Our aim is to create an environment in the laboratory that is simulating a walk in a forest by projecting VR images on a large dome screen linked to the movement of a treadmill. Using this environment, we are currently investigating the relationship between the forward-looking mind and the body. In addition to biomechanical data, such as walking posture, psychophysiological data, such as electroencephalogram and electrocardiogram recordings, respiration, and gaze behavior are being acquired to comprehensively understand the body and determine the relationship between the body and the mind. In this presentation, we will introduce the VR environment for walking constructed for this research, present the current analysis results using multidimensional data on walking in the VR environment, and suggest an intervention experiment that takes advantage of the strengths of the VR environment, such as the function to add audio-visual stimuli as desired.

### Symposium

[3S09a]
**New perspectives on physiological functions and control mechanisms of energy sources:from feeding to pathological condition**

March 30, 14:20 - 16:20, Room 9

### [3S09a-1]

### Sweet taste memory changes peripheral glucose metabolism via the basolateral amygdala

*Chitoku Toda[1] ([1]*Kumamoto University, Faculty of Life Sciences, Department of Neuroscience for Metabolic Control*)

Anticipatory physiological responses to food were first reported by Ivan Pavlov a century ago. Pavlov showed that when food-independent sound cues are associated with food, a representation of the cue increases the secretion of saliva, gastric acid, pancreatic enzymes, etc. Similar effects also occur when food is perceived through sight, smell, and taste. These reactions are called cephalic responses and enable efficient digestion, absorption, and nutritional regulation before the start of food intake. Cephalic responses play an essential role in the brain's regulation of systemic energy metabolism, including glucose metabolism. When sugar is expected to be ingested, the cephalic phase insulin release increases to prepare for the control of blood glucose levels before food intake. However, the associated neural mechanism is still ill-defined. Here, we identified two types of neurons in the basolateral amygdala (BLA), which are activated by sweetener (saccharin) or water after sucrose conditioning, representing expected sweet taste and unmet expectation, respectively. Saccharin-induced met-expectation of sweet taste enhances, while $H_2O$-induced unmet-expectation deteriorates, glucose metabolism in peripheral tissues. Deletion of saccharin-responsive neurons in BLA impaired saccharin-induced increase in insulin sensitivity. Deletion of $H_2O$-responsive neurons in BLA improved glucose intolerance by unmet-expectation. Saccharin- and $H_2O$-responsive neurons had different gene expressions. Our data suggest that distinct BLA neurons evaluate the gap between the expected incoming sugar and sweet taste to control peripheral glucose metabolism.

### [3S09a-2]

### Mechanisms underlying metabolic adaptations in response to dietary protein deprivation

*Yuka Toyoshima[1] ([1]*Utsunomiya University*)

Protein deprivation suppresses protein synthesis and causes growth retardation. The reduction in the circulating insulin-like growth factor-I is associated with these events. In addition, protein deprivation is known to affect glucose and lipid metabolism, leading to fatty liver in humans and animals. Previous studies including ours have shown that protein deprivation suppresses insulin secretion in response to glucose but does not impair glucose tolerance. The insulin tolerance test confirmed that the low-protein diet increased insulin sensitivity. To determine in which tissues insulin sensitivity is increased upon protein deprivation, we analyzed the amount and phosphorylation of insulin signaling molecules in the liver, skeletal muscle, and adipose tissues. The low-protein diet increased the amounts of insulin receptor substrate (IRS)-2 and eukaryotic translation initiation factor 4E-binding protein 1 (4E-BP1) in the liver. IRS-2 is a major substrate of insulin receptor and mediates insulin action to stimulate lipid synthesis. 4E-BP1, a substrate of mechanistic target of rapamycin complex 1 and a translation initiation repressor, has shown to regulate lipid metabolism. Therefore, we examined whether IRS-2 and 4E-BP1 are involved in the increased insulin sensitivity and the development of fatty liver under protein deprivation using gene knock-out and knock-down techniques. The results indicate that both signaling molecules are required for the development of fatty liver and IRS-2 plays important roles in the enhancement of insulin sensitivity. Protein deprivation is increasingly considered as a dietary approach to improve metabolic health since it has a variety of beneficial effects, such as the extension of life span and the increased energy expenditure. Fibroblast growth factor 21 (FGF21) is known to be essential to mediate these beneficial effects of protein deprivation. In addition, protein deprivation has shown to increase FGF21 synthesis in the liver and its circulating level, which is likely to prevent the amelioration of fatty liver. These changes of metabolism could be considered as a metabolic adaptation in response to protein deprivation. Here, I introduce our studies and others and discuss the mechanisms by which animals regulate metabolism to adapt to protein deprivation.

### [3S09a-3]

### Effect of exogenous acute β-hydroxybutyrate administration on skeletal and cardiac muscle energy metabolism during exercise and rest in rats

*Motoyuki Iemitsu[1] ([1]*Ritsumeikan University*)

A ketone body (β-hydroxybutyrate [β-HB]) is synthesized by β-oxidation of fatty acids in the liver and used as an energy source in peripheral tissues, such as skeletal muscle, heart, and nervous system via blood circulation. It is oxidized as an alternative to pyruvate and enter the mitochondria to provide acetyl-CoA for the tricarboxylic acid (TCA) cycle, resulting in a greater adenosine triphosphate (ATP) supply. Therefore, effective utilization of β-HB during exercise may accelerate exercise performance through improved energy supply. In several previous studies, exogenous acute β-HB ingestion before exercise enhanced endurance exercise performance. As the mechanisms, exogenous acute β-HB ingestion reduces the energy supply from glycolysis and increases oxidative metabolism in skeletal muscles by preferentially using β-HB. However, the effects of exogenous acute β-HB ingestion on exercise mode with different energy supply pathways to skeletal and cardiac muscle remains unclear. Recently we investigated the effect of exogenous acute β-hydroxybutyrate administration on skeletal and cardiac muscle energy metabolism during exercise and rest in rats (Med Sci Sports Exerc 55: 1184-1194. 2023). This study examined the effects of exogenous acute β-HB administration on exercise performance (endurance, resistance, and high-intensity intermittent exercise [HIIE]) in healthy Sprague Dawley (SD) rats (Study 1). Additionally, metabolome analysis was performed to profile the effects of exogenous β-HB administration on acute HIIE-induced metabolic responses in the skeletal and heart muscles (Study 2). In this symposium, my presentation would like to focus on the effect of β-HB on energy metabolism in skeletal and cardiac muscle in different exercise mode.

### [3S09a-4]

### Alterations in nutrient metabolism and its control signaling and cognitive dysfunction

*Akiko Taguchi[1] ([1]*National Center for Geriatrics and Gerontology*)

High-fat- diet intake in middle-aged and its -associated disease, type 2 diabetes, are known as risk factors for dementia, and it has recently known that malnutrition due to insufficient protein intake in old age is also a risk factor for cognitive frailty. However, the molecular mechanism by which malnutrition induces cognitive decline is not clear. In our studies, chronic low-protein diet mice in old age revealed decreased body weight, reduction in muscle mass and strength, abnormal gluconeogenesis, impaired thermoregulation, and increased expression of orexigenic neuropeptides, AgRP and NPY, in the hypothalamus. Moreover, decreased spontaneous activity and reduction in the hippocampus and forebrain -associated spatial memory function occurred accompanied by a significant decline in neuronal activity in CA1 and dentate gyrus. In the hippocampus, we observed decrease in tyrosine phosphorylation of insulin receptor substrate 1 (IRS1), a key regulator of insulin signaling and in phosphorylation of Akt, which reflect signaling activation. Additionally, we found that inactivation of IRS1 in astrocytes, which are responsible for nutrient energy metabolism in the brain, leads to impairment of systemic nutrient energy metabolism and decreased spatial memory functions. These results suggested that long-term protein deprivation which causes sarcopenia and systemic abnormalities in nutrient energy metabolism impairs cognitive function, and its mechanism by which cognitive dysfunction occurs may be involved in altered glial insulin signaling.

### [3S09a-5]

### Role of dead cell clearance by macrophages as a molecular mechanism of chronic inflammation

*Miyako Tanaka[1], Takayoshi Suganami[1] ([1]*Dep. Molecular Medicine and Metabolism, RIeM, Nagoya Univ.*)

Substantial attention has been paid to dead cells and their clearance as a molecular mechanism of non-resolving inflammation. It is also elucidated that damage-associated molecular patterns (DAMPs) released from dead cells act on surrounding cells and are involved in the formation of various pathological conditions. So, it is important to identify the specific DAMPs to further understand the molecular mechanism of the pathological condition. In 2005, the existence of crown-like structure (CLS) was reported, where macrophages surround and engulf dead adipocytes in obese adipose tissue. We and others have shown that CLS triggers obesity-induced adipose tissue inflammation and fibrosis, suggesting the significance of dead cells in chronic inflammation. On the other hand, we found that CLS formation is observed in experimental models of acute kidney injury (AKI) and nonalcoholic steatohepatitis (NASH). In AKI, bone marrow-derived macrophages form CLS in close proximity to necrotic tubules, and in NASH, resident macrophages (Kupffer cells) constitute CLS surrounding dead hepatocytes with excessive lipid accumulation.
By interacting with dead cells, these CLS-forming macrophages acquire proinflammatory and profibrotic properties with altered intracellular metabolism, and then give rise to acceleration of pathological conditions and sustained inflammation. In this session, I would like to review the molecular mechanisms of chronic inflammation caused by the interaction between macrophages and dead cells.

### Symposium

[3S10a]
Cooperation with Other Societies Committee
**Lifelong development, adaptation and deterioration in oral physiology**

March 30, 14:20 - 16:20, Room 10

### [3S10a-1]

### Mechanism of feeding behavior change associated with nausea induction.

*Makoto Funahashi[1] ([1]*Oral Physiology, Graduate school of dental medicine, Hokkaido University*)

The oral physiological functions that are expected to develop throughout life are the learning and memory of eating experiences. Feeling the taste, flavor and texture and maintaining the function of eating deliciously enriches life. Memories of delicious food experiences are constantly updated. In other words, the importance of eating behavior for humans is not only from the viewpoint of nutritional intake, but also to improve the quality of life by memorizing and learning about food experiences. Anorexia occurs with various diseases and their treatment. This not only leads to nutritional deficiencies, but also reduces the quality of life and happiness due to memorization and learning of food experiences. Indeed, it can be said that maintaining the healthy oral physiological functions is essential for improving happiness. However, much is still unclear about the neural mechanisms of memory and learning about food experiences. Therefore, we would like to clarify the neural mechanism of behavioral change caused by memory and learning about eating experiences by investigating conditioning behavior that causes rats to dislike the sweetness of saccharin, which is originally pleasant information, by inducing nausea with various drugs. So far, we have investigated the chemoreceptivity of area postrema neurons, which are well known as chemoreceptor trigger zones that induce vomiting, and clarified the characteristics of neurons that are likely to be involved in nausea induction. We also used behavioral experiments to investigate how H-channel inhibition, area postrema lesions (APX) and the vagal afferent vagotomy (VX) affect the acquisition of conditioned taste aversion (CTA) in rats. Administration of H-channel antagonist attenuates CTA. Also, the effects of APX and VX vary depending on the type of nausea-inducing substance used for conditioning. In this talk, we present the experimental results comparing the conditioning by emetine and cisplatin. Emetine is an alkaloid contained in the root of the vomit root and induces acute nausea and vomiting. Cisplatin is an antineoplastic drug that induces acute, delayed and anticipatory nausea and vomiting as its side effects. Emetine-induced CTA disappears with APX. Cisplatin-induced CTA does not disappear with both APX and VX, but CTA disappears with the addition of pre-administration of dexamethasone to APX and VX. Recently, we have started experiments to observe the gaping response of rats as an indicator of conditioned nausea and to investigate the effect of conditioned taste aversion to saccharin on the intake of sweet feed, so we will introduce the pilot data that we have obtained so far.

### [3S10a-2]

### Physiological research for elucidating the pathophysiology of dental sleep-related conditions

*Takafumi Kato[1,2,3], Ayano Katagiri[1], Hiroki Toyoda[1] ([1]*Osaka University Graduate School of Dentistry, Department of Oral Physiology,* [2]*Osaka University Medical Hospital Sleep Medicine Center,* [3]*Osaka University United Graduate School of Child Development*)

There is growing recognition that dentists can have a role in both diagnosing and managing certain sleep disorders, along with the associated orodental issues. Nevertheless, there is a limited body of physiological research on dental sleep-related conditions. One common condition is sleep bruxism (SB), which affects approximately 20% of children and 10% of adults. Polysomnographic studies have indicated that rhythmic masticatory muscle activity occurs more frequently in individuals with SB during non-rapid eye movement (NREM) sleep, in association with transient arousals and cyclic sleep patterns. To delve deeper into the neurophysiological mechanisms of SB, studies have been conducted for developing an animal model. In naturally sleeping animals, rhythmic activity of masticatory muscles can be found to occur. The occurrence of rhythmic activity of masticatory muscles coincides with cortical and cardiac activations during NREM sleep while repetitive phasic bursts of masticatory muscles during rapid eye movement sleep mimic the patterns observed during chewing. Additionally, experimental stimulation to pyramidal tract induced rhythmic contractions of the masseter muscles during sleep. Another prevalent sleep disorder is obstructive sleep apnea (OSA), which is characterized by intermittent hypoxic conditions due to the occurrence of apnea or hypopnea. OSA has been linked to a heightened risk of chronic orofacial pain and SB. To explore the pathophysiological relationship between orofacial pain and OSA, experiments were conducted using a model that subjected animals to chronic intermittent hypoxia (CIH). The findings revealed that CIH-exposed animals displayed increased intraoral sensitivity to capsaicin solution, accompanied by an increase in transient receptor potential vanilloid 1(TRPV1)-positive neurons in the trigeminal ganglion. The results indicate that CIH could induce hyperalgesic condition in orofacial region. Further physiological studies are required to investigate the pathophysiology of dental sleep-related conditions through the utilization of animal models, enabling the exploration of causal or modulatory impacts of risk factors as proposed in clinical studies.
COI: Properly Declared.

### [3S10a-3]

### Defective neural circuit formation and brain function due to oral system failure

*Naofumi Uesaka[1], Moe Tanigawa[1], Midori Wada[1], Chiho Kato[1], Takashi Ono[1] ([1]*Tokyo Medical and Dental University*)

Deficiencies in the oral system, including food softening, tooth loss, dental caries, periodontal disease, and mouth breathing, have been linked to diminished brain function. This suggests that the oral system has implications beyond oral health, extending to overall brain functionality. Prior research has identified tooth loss, compromised chewing ability, and mouth breathing as risk factors for cognitive impairments, including deficits in memory and learning. Despite these findings, several questions remain unanswered. Specifically, it is yet to be determined whether the oral system influences other aspects of brain function and the underlying mechanisms remain elusive. To address these gaps, we employ three distinct mouse models that exhibit oral system deficiencies. Our research focuses on the cerebellum, a region known for its role in sensory-motor functions and its contributions to cognitive, social, and emotional coordination. We particularly examine the process of synapse elimination, a critical phase in brain development. Here we would like to show our resent results about brain function and synapse elimination in mouse models of deficiencies in the oral system and discuss roles of oral system in development and function of brain.

### [3S10a-4]

### Response properties and morphological organizations of trigeminal mechanoreceptors

*Takahiro Furuta[1] ([1]*Graduate school of dentistry, Osaka University*)

The trigeminal nervous system is heavily involved in somatosensory mechanism of the head region and is essential for the realization of advanced functions such as chewing, swallowing, breathing, and speech. Of the trigeminal sensation, information obtained from mechanoreceptors is known to contribute not only to precise tactile perception but also to the refinement of motor control. In this study, in order to deepen our understanding of the mechanisms of trigeminal tactile reception, we investigated the response characteristics of mechanoreceptors to experimental stimuli and also morphologically analyzed the receptors and their surrounding structures using the rodent whisker system as a subject. Intra-axonal recordings were made in the spinal trigeminal tract of the brainstem to investigate response properties in single primary afferent fibers to whisker stimulus of square-wave shape or high frequency sine-wave shape. After activity recording, the peripheral endings (mechanoreceptors) of the recorded axons were visualized morphologically by intra-axonal injection of biotinylated dextran amine. Mechanoreceptors have been known to be divided into several types according to their morphological characteristics. Here, we found that each type had different activity characteristics. By integrating the results of simulation analysis and analysis of the relationship with the surrounding structures, it was shown that the multiple mechanoreceptor types play different roles. In this presentation, we will focus on recently obtained data on Ruffini mechanoreceptor.

### [3S10a-6]

### Excitatory and inhibitory synaptic organization in development of jaw movement-related motoneurons

*Shiro Nakamura[1] ([1]*Department of Oral Physiology, Showa University School of Dentistry*)

Feeding behaviors such as suckling and mastication, essential for survival of mammals, undergo changes during the early period of postnatal development. The properties of neural circuits associated with orofacial motor activity also show advances with maturity during this period, accompanied by tooth eruption and growth of orofacial musculoskeletal structures. In the present study, electrophysiological properties of glutamatergic, GABAergic, and glycinergic transmissions from premotor neurons to jaw-closing and -opening motoneurons during early postnatal development in rats were analyzed. Postnatal day (P)2-5, P9-12, and P14-17 age groups, corresponding to the suckling period before tooth eruption, post eruption, and immature chewing, respectively, were examined, and showed that the properties of non-NMDA receptor-mediated miniature excitatory postsynaptic currents (mEPSCs) in both motoneurons were not different among them. However, those of NMDA mEPSCs were more active in P2-5 masseter motoneurons, followed by a decrease in association with neuron maturity. As for inhibitory inputs, the amplitude and frequency of glycinergic miniature inhibitory postsynaptic currents showed dramatic increases with age in both the masseter and digastric motoneurons. In contrast, the frequency of GABAergic components in masseter motoneurons was higher at P2-5 than at P14-17, while that in digastric motoneurons remained constant throughout the postnatal period. Neurons expressing transcription factor Phox2b were also found densely distributed in the reticular formation surrounding the trigeminal motor nucleus, which were predominantly glutamatergic premotor neurons with low-frequency firing characteristics, whereas the majority of Phox2b-negative neurons were GABAergic and glycinergic premotor neurons with high-frequency firing patterns. These findings suggest different age-related characteristics of excitatory and inhibitory inputs between jaw-closing and -opening motoneurons, and among glutamatergic, GABAergic and glycinergic currents, thus indicating distinct features that possibly reflect feeding behavior development.

### [3S10a-5]

### Circadian rhythms in trigeminal innervated regions and expression of clock genes in the trigeminal ganglion of mice

*Sachi N Ohno[1,2], Mitsutaka Sugimura[1] ([1]*Dental Anesthesiology, Graduate School of Medical and Dental Sciences, Kagoshima University,* [2]*Anesthesiology, Aso Iizuka Hospital*)

The intensity of pain in the orofacial region, including caries, is known to vary throughout the day. This has been thought to be influenced by various factors such as blood pressure and autonomic nervous system activity. However, it is possible that diurnal variations exist in the activity of the trigeminal nerve itself, which is responsive to orofacial pain. The purpose of this study was to investigate circadian rhythms in the trigeminal innervated area and to elucidate the rhythm of expression of clock genes in the trigeminal ganglion (TG) of mice to understand the mechanism of circadian regulation in the same area.

Ten-week-old male mice were maintained under a 12/12-hour light/dark cycle for at least 10 days. Subsequently, formalin was injected into the second branch region of the trigeminal nerve and the duration of pain-related behaviors (PRBs) was assessed. Immunohistochemical staining was then performed, and the number of c-Fos-immunopositive cells in the trigeminal spinal tract subnucleus caudalis (Sp5C) was quantified. Additionally, the TGs were extracted from the mice and examined using quantitative real-time PCR to evaluate the daytime and nighttime expression of nociceptive receptors. The results indicated that the duration of PRBs was longer and the number of c-Fos immunopositive cells in the Sp5C was higher at nighttime compared to daytime. Furthermore, the mRNA expression of transient receptor potential ankyrin 1 in the TG was significantly higher at night than during the day. These results suggest that pain in the trigeminal nerve region is more intense at nighttime, when rodents are active, partly due to differences in nociceptor expression.

Next, to measure gene expression as bioluminescence, PERIOD2::LUCIFERASE knock-in (PER2::LUC) mice were utilized. Unilateral TG and brain sections, including the suprachiasmatic nucleus (SCN), were incubated *ex vivo*. Bioluminescence levels were measured using a highly sensitive photodetector. Similar experiments were conducted with *Cry1*-gene deficient (*Cry1*-/-) or *Cry2*-gene deficient (*Cry2*-/-) mice. Additionally, immunohistochemistry examined the expression of the PER2 protein in the SCN and TG of wild-type mice. Mouse TG *ex vivo* tissue exhibited distinct circadian oscillations in PER2::LUC levels in all genotypes. The period was shorter in the TG than in the SCN; it was shorter in *Cry1*-/- and longer in *Cry2*-/- mice than in the wild-type mice. In the TG, immunohistochemistry localized PER2 protein expression within the neuronal cell body. The expression of *Per2* in neurons of the TG in *ex vivo* culture and the oscillation in a distinct circadian rhythm suggest that the TG is responsible for the relay of sensory inputs and temporal gating through autonomous circadian oscillations.

### Educational Program

### Educational Lecture 1

[1EL02m]

March 28, 8:50 - 10:50, Room 2

### [1EL02m-1]

### A physiological approach to understanding and utilizing brain information

Kenji Kansaku *(Dokkyo Medical University School of Medicine)*

Technological advances have enabled researchers to obtain physiological signals from the living brain either invasively or non-invasively. Our research group has used the acquired brain signals to pursue research towards understanding brain function based on brain information and utilization of brain information for medical practice.

We are more specifically focused on the neural mechanisms of body image and sense of self, as well as practical neuroprosthetics. For example, in one study, we developed a robotic arm with myoelectric control, in which the user continuously varied the joint position, in a robot hand illusion task, and showed that the 3 transradial amputees experienced sense of ownership and agency over the robotic arm (Sato, et al., 2018). In addition, we created a rubber tail illusion task in mice, and suggested that mice can experience body ownership of their tails (Wada, et al., 2016; 2019). The establishment of animal experimental models of body image and sense of self is expected to contribute to the understanding of the neural basis, and to the development of advanced neuroprosthetics (Kansaku, 2021).

In another study, we collected brain information from clinically diagnosed patients with unresponsive wakefulness syndrome (UWS) by using an fMRI language task (Kansaku, et al., 2000), and 2 out of 10 patients showed activation in the posterior language areas while listening to spoken narratives. We then used a steady state visual evoked potential (SSVEP)-based brain-computer interface (BCI) task and showed that the 2 patients successfully modulated their focal attention to the visual target to operate the in-house BCI device, suggesting that they maintained a minimally conscious state (Okahara, et al., 2023).

Further studies along these lines are underway, with the ultimate aim of contributing to the well-being of patients with impaired communication and control.

### [1EL02m-2]

### Discovery of a novel intracellular degradation system: solving the long-standing mystery of the lens

Hideaki Morishita *(Kyushu Univ.)*

All intracellular organelles such as mitochondria, endoplasmic reticulum, and lysosomes are completely degraded during the differentiation process of lens cells of the eye, but its molecular mechanism and physiological significance have been unknown over 100 years. To solve this long-standing question, we constructed an in vivo screening system using zebrafish, and identified PLAAT (phospholipase A/acyltransferase), which is highly conserved in vertebrates, as an essential factor for lens organelle degradation. We further demonstrated that PLAAT-mediated organelle degradation is essential for lens transparency in both mice and zebrafish. This study revealed for the first time the existence of an organelle degradation mechanism that does not depend on autophagy.

In this lecture, I will introduce the process and results of research that took 12 years to solve the long-standing question.

### [1EL02m-3]

### Cardiovascular Developmental Physiology

SUSUMU MINAMISAWA *(The Jikei Univ. Dept. of Cell Physiology)*

### Model Lecture

[2EL02m]

March 29, 8:50 - 10:50, Room 2

### [2EL02m-1]

**What is "Pain"? -No pain, no gain-**

Daisuke Uta *(Department of Applied Pharmacology, Faculty of Pharmaceutical Sciences, University of Toyama)*

### [2EL02m-2]

**Environmental Physiology - Physiological responses to space environment**

Tomomi Watanabe-Asaka *(Tohoku Medical and Pharmaceutical University)*

### [2EL02m-3]

**Homeostatic control of blood glucose**

Michiko Tanaka *(Miyazaki Prefectural Nursing University)*

### Education Workshop

[2WS02a]

March 29, 14:20 - 16:20, Room 2

**[2WS02a-1]**

Kayoko Matsushima *(Medical Education Development Center, Nagasaki University Hospital)*

### Educational Lecture 2

[3EL02m]

March 30, 8:50 - 10:50, Room 2

### [3EL02m-1]

### Quantification and visualization of brain and body dynamics through data-driven approaches

Keiichi Kitajo[1,2] *([1]National Institute for Physiological Sciences, [2]The Graduate University for Advanced Studies (SOKENDAI))*

Our research employs data-driven approaches for the quantification and visualization of brain and body dynamics, with a particular emphasis on synchronous oscillatory activities. Initially, I outline the theoretical underpinnings of neural oscillations and synchrony through the lens of dynamical systems theory. Subsequently, I detail our scalp electroencephalography (EEG) studies, which link EEG synchrony with individual psychological traits and brain disorders, including stroke. Furthermore, I highlight our investigations into state-dependent synchrony between EEG activity and physiological signals from the body, such as those from electrocardiography and respirometry. Finally, I introduce our data-driven methodologies which monitor dynamic shifts in the excitation/inhibition (E/I) balance, derived from scalp EEG, through the application of data assimilation techniques.

### [3EL02m-2]

### Social behavior and oxytocin

Yuki Takayanagi *(Division of Brain and Neurophysiology, Department of Physiology, Jichi Medical University)*

The nona-peptide hormone oxytocin is produced in the hypothalamus and released into the circulation from the posterior pituitary. Oxytocin is also released from the axon terminals and the dendrites of oxytocin-synthesizing neurons in the brain. Its peripheral functions include muscle contractions associated with reproduction, such as milk ejection during lactation and uterine contractions during parturition. Recently, central oxytocin has been shown to play a crucial role in various prosocial behaviors, including social recognition, social bonding, and parental care. However, depending on the situation, oxytocin can also contribute to anti-social behaviors like envy, gloating, and aggression. In addition, it has been shown that oxytocin activity in the early-life stage may influence social behavior in adulthood. The effects of the oxytocin system on the regulation of social behavior appear to vary considerably depending on changing environments and to be more complex than initially thought.

### [3EL02m-3]

### Cardiorespiratory exercise physiology: Basic and applied

Hidefumi Waki *(Juntendo University)*

Physical fitness (PF) is the power that humans require to lead a healthy and fulfilling life. PF comprises physiological, anatomical, and psychological components that support physical and mental activities. PF is a fundamental factor in exercise and sports. Exercise training aimed at extending healthy life expectancy (HLE), maintaining and improving the quality of life, and improving athletic performance is necessary for enhancing PF. The content of an exercise training program depends on individual health conditions, PF levels, and training objectives. To provide proper exercise training instructions, it is necessary to understand the physiological components that constitute PF and the plasticity of each component induced by exercise. One of the most important components of PF, particularly exercises for extending HLE, is cardiorespiratory fitness (CRF), which is also known as aerobic capacity. CRF is the ability to perform sustained activities, such as running or cycling, which require several skeletal muscle groups to work in tandem. Skeletal muscles recruited during exercise absorb the required oxygen and energy substrates from the bloodstream and rapidly expel the metabolites produced by the active skeletal muscle into the blood. The lungs take in sufficient oxygen from the air for this activity and release carbon dioxide. Therefore, enhanced lung ventilation is essential to increase gas exchange, and cardiac function is necessary to increase blood flow in the lungs and active muscles during exercise. The cardiorespiratory system is strengthened by repeated stimulation by exercise training, thereby improving the CRF, PF, and health. This presentation provides an overview of the basic knowledge and recent findings on respiratory and circulatory responses observed during exercise, their regulatory mechanisms, and the effects of exercise training on CRF. We hope this lecture serves as an opportunity for members of the Physiological Society to reaffirm the importance of exercise for better health and will help them create better exercise programs in a medical setting.

### Oral Presentation

### Oral

[1O07-01]
**Endocrine**

March 28, 8:50 - 9:50, Room 7

### [1O07-01-01]

### Remission of social behavior impairment by oral administration of a precursor of NAD in CD157, but not in CD38, knockout mice by elevating NAD and oxytocin by NAD-boosting nicotinamide riboside

*Haruhiro Higashida[1] ([1]*Kanazawa University Research Center for Child Mental Development*)

Nicotinamide adenine dinucleotide (NAD) is a substrate of adenosine diphosphate (ADP)-ribosyl cyclase and is catalyzed to cyclic ADP-ribose (cADPR) by CD38 and/or CD157. cADPR, a $Ca^{2+}$ mobilizing second messenger, is critical in releasing oxytocin from the hypothalamus into the brain. Although NAD precursors effectively play a role in neurodegenerative disorders, muscular dystrophy, and senescence, the beneficial effects of elevating NAD by NAD precursor supplementation on brain function, especially social interaction, and whether CD38 is required in this response, has not been intensely studied. Here, we report that oral gavage administration of nicotinamide riboside, a perspective NAD precursor with high bioavailability, for 12 days did not show any suppressive or increasing effects on sociability (mouse's interest in social targets compared to non-social targets) in both CD157KO and CD38KO male mice models in a three-chamber test. CD157KO and CD38KO mice displayed no social preference (that is, more interest towards a novel mouse than a familiar one) behavior. This defect was rescued after oral gavage administration of nicotinamide riboside for 12 days in CD157KO mice, but not in CD38KO mice. Social memory was not observed in CD157KO and CD38KO mice; subsequently, nicotinamide riboside administration had no effect on social memory. Elevating NAD by NAD-boosting nicotinamide riboside may allow animals with cADPR and oxytocin-forming deficits to overcome these deficits and function more normally. Together with the results that nicotinamide riboside had essentially no or little effect on body weight during treatment in CD157KO mice, nicotinamide riboside is less harmful and has beneficial effect on defects in recovery from social behavioral, for which CD38 is required in mice.

### [1O07-01-02]

### Tmem119 is crucial for bone formation activity of PTH in mice

*Naoyuki Kawao[1], Daichi Matsumura[1,2], Ayaka Yamada[1], Katsumi Okumoto[3], Takashi Ohira[1], Yuya Mizukami[1], Daiki Hashimoto[1], Hiroshi Kaji[1] ([1]*Department of Physiology and Regenerative Medicine, Kindai University Faculty of Medicine,* [2]*Department of Orthopaedic Surgery, Kindai University Faculty of Medicine,* [3]*Life Science Research Institute, Kindai University*)

We previously identified Transmembrane protein 119 (Tmem119) as a TGF-β- and Smad3-related factor closely linked to osteoblastic bone formation in *in vitro* study. Moreover, it was selected as a crucial gene linked to the osteoblast phenotype and bone mass in the gene-linkage analyses, and Tmem119 expression is enhanced by PTH in mouse osteoblastic cells. Although PTH has been used as a potent drug which enhances bone formation for osteoporotic patients, roles of Tmem119 in PTH-induced bone formation *in vivo* have remained unknown. In the present study, we investigated the involvement of Tmem119 in bone anabolic actions enhanced by the intermittent PTH administration using Tmem119-deficient mice. Twelve-week-old Tmem119-deficient and wild-type mice were injected intraperitoneally 80 µg/kg PTH-(1-34) for 6 weeks. Bone mineral density (BMD) and bone volume at the femurs were measured using micro-computed tomography. Tmem119 deficiency significantly blunted trabecular bone volume, cortical BMD and bone microarchitecture change enhanced by PTH in the femurs of mice. Histomorphometric analyses with calcein labeling revealed that Tmem119 deficiency significantly blunted the rates of bone formation and mineralization as well as numbers of osteoblasts, but not osteoclasts, enhanced by PTH in mice. Moreover, Tmem119 deficiency significantly blunted β-catenin level and alkaline phosphatase (ALP) activity enhanced by PTH in mouse osteoblasts, although its effects on osteoblast apoptosis and early-stage osteoblastic differentiation suppressed by PTH were not significant. In conclusion, we showed that Tmem119 is involved in the bone anabolic actions of PTH partly through canonical Wnt/β-catenin signaling and ALP activity in mice. COI: NO.

### [1O07-01-03]

### Developmental exposure to perfluorooctane sulfonate leaves a potential risk in brain function of middle-aged male mice

*Ayane Kate Ninomiya[1], Izuki Amano[1], Hiraku Suzuki[1], Yuki Fujiwara[1], Asahi Haijima[2], Noriyuki Koibuchi[1] ([1]*Dept. Integrative Physiology, Gunma Univ. Grad. Sch. Med.,* [2]*Dept. Environmental Brain Sciences, Faculty of Human Sciences, Waseda Univ.*)

Perfluorooctane sulfonate (PFOS) exerts an adverse effect on neuronal development. We previously reported that the lactational PFOS exposure causes the retardation of cognitive and motor development in mouse offspring in young adulthood. Whereas health outcomes following developmental PFOS exposure in young population have been widely reported in both epidemiological and experimental studies, little is known about PFOS toxicity in aging population. Limited evidences have shown that early-life PFOS exposure holds a potential risk for developing age-related neurodegenerative diseases such as Alzheimer's disease later in life. However, the mechanism of PFOS-induced neurodegeneration is unclear. The present study investigated the effects of lactational PFOS exposure on cognitive function using one-year-old aged mice. Dams were exposed to PFOS (1 mg/kg body weight) through lactation (postnatal day 0 – 14) by gavage. One-year-old male offspring were used for the behavior test battery to assess cognitive function and then sacrificed to extract the hippocampal tissues. Western blot analysis was conducted to measure the levels of proteins related to the pathogenesis of Alzheimer's disease. There was no drastic change in cognitive behavior in PFOS-exposed mice, excluding a mild deficiency in social recognition. In the hippocampus, the expression of tau protein was significantly increased. However, proteins related to tau hyperphosphorylation were not changed. These results underline a mild effect of developing PFOS exposure on cognitive function and neurodegeneration. Yet, the present study at least presents the long-lasting effects of PFOS even in middle-aged period and warrants a potential aftermath.

### [1O07-01-04]

### Lability of oxytocin receptor expression in the cerebellum and its modulation

*Ayumu Inutsuka[1], Aisa Hattori[1], Masahide Yoshida[1], Yuki Takayanagi[1], Yukiko U. Inoue[2], Tatsushi Onaka[1] ([1]*Jichi Medical University,* [2]*National Institute of Neuroscience, National Center of Neurology and Psychiatry*)

The cerebellum regulates not only body movement, but also cognitive and social function. Pediatric cerebellar injury increases the risk of autism spectrum disorder, and cerebellar inflammation induces depression-like behaviors and social avoidance in mice. Oxytocin is involved in social relationships and the oxytocin receptor mediates its role in social behavior. However, the expression pattern of the oxytocin receptor in the cerebellum is controversial, and the physiological role of oxytocin in the cerebellum is still unknown. Here, we report that the expression pattern of the oxytocin receptor in the cerebellum is highly variable among transgenic lines. We used an Oxtr-Cre knock-in mouse combined with a fluorescent reporter line and found that oxytocin receptor expression is much more variable in Bergmann glia than in Purkinje cells during development. In these mice, the number of fluorescent protein-expressing cells in Purkinje cells remained almost constant throughout development, whereas the number of fluorescent protein-expressing cells in Bergmann glia increased significantly. We also found that physical damage to the cerebellum selectively activates oxytocin receptor expression in Bergmann glia. Our findings highlight the importance of the high variability of oxytocin receptor expression in the cerebellum and provide a possibility that the oxytocin receptor may influence neural processing in pathological conditions such as inflammation.

### [1O07-01-05]

### Elucidation of metabolic abnormalities in vasopressin receptor-deficient mice

*Kazuki Harada[1], Eiji Wada[2], Kie Shimizu[3,4], Masao Miyazaki[4,5], Yukiko Hayashi[2], Kauzaki Nakamura[3], Takashi Tsuboi[1] ([1]*Department of Life Sciences, Graduate School of Arts and Sciences, The University of Tokyo,* [2]*Department of Pathophysiology, Tokyo Medical University,* [3]*Department of Pharmacology, National Research Institute for Child Health and Development,* [4]*Division of Life Science, Graduate School of Science and Engineering, Saitama University,* [5]*Department of Biological Chemistry, School of Agriculture, University of Iwate*)

Arginine vasopressin (AVP) is an antidiuretic hormone secreted by the pituitary gland and involved in a various physiological functions including pair bonding, circadian rhythm, and metabolic homeostasis. AVP V1a receptor (*V1aR*)- and/or V1b receptor (*V1bR*)-deficient mice show altered glucose and lipid metabolism. However, the precise mechanisms of this pathophysiology remain unknown. We focused on the role of gut microbiota and gut hormones, specifically glucagon-like peptide-1 (GLP-1) and peptide YY (PYY) hormones derived from enteroendocrine L cells, in AVP receptor-deficient mice. Our results show that GLP-1 and PYY secretion was impaired in V1aR-deficient ($V1aR^{-/-}$), V1bR-deficient ($V1bR^{-/-}$), and V1aR/V1bR double-deficient ($V1aR^{-/-}$ $V1bR^{-/-}$) mice. $V1aR^{-/-}$ $V1bR^{-/-}$ mice also showed increased expression of Paneth cell-related genes in the small intestinal epithelia. In addition, we observed an increase in fecal butyrate levels and butyrate-producing *Clostridium* IV bacteria in these mice. When treated with butyric acid, mouse L-cells showed inhibited GLP-1 secretion. However, glucose intolerance was not observed in $V1aR^{-/-}$, $V1bR^{-/-}$ or $V1aR^{-/-}$ $V1bR^{-/-}$ mice. In contrast, $V1aR^{-/-}$ $V1bR^{-/-}$ mice showed increased lipid accumulation in their slow muscle fibers and increased lipid droplets in their brown adipose tissue. Thus, enteroendocrine L-cell functions and lipid metabolism in both skeletal muscle and brown adipose tissue are impaired by the overproduction of intestinal butyrate in AVP receptor-deficient mice.

### Oral

[1O07-02]
### Neurochemistry, Glia, Higher brain function

March 28, 9:50 - 10:50, Room 7

### [1O07-02-01]

### Developing multiplex imaging for deciphering $Ca^{2+}$-dependent biochemical signaling under physiological and pathological conditions in the brain

*Hajime Fujii[1], Yayoi Kondo[1], Keisuke Ota[1], George Cai[2], Richard Song[3], Haobo Song[1], Shin-ichiro Horigane[4], Sayaka Takemoto-Kimura[4,5], Haruhiko Bito[1] ([1]*Deptartment of Neurochemistry, Graduate School of Medicine, The University of Tokyo, [2]Department of Physics, Harvard University, [3]Department of Neuroscience, Vanderbilt University, [4]Res. Inst. of Envrn. Med. (RIEM), Nagoya Univ., Nagoya, [5]Dept. of Molecular/Cellular Neurosci., Nagoya University Graduate School of Medicine*)

$Ca^{2+}$ transients are triggered by various neuronal events and their precise measurements are essential to investigate synaptic transmission, local dendritic spikes and action potential firing. Among downstream $Ca^{2+}$-dependent effectors, CaMKIIalpha and calcineurin stand out as they are critical for regulating neuronal plasticity, learning and memory. Thus, better understanding of how $Ca^{2+}$ and the downstream kinase and phosphatase signals are activated during cognitive processes, and deciphering the dynamics of their spatial and temporal codes, are fundamental, yet unanswered, questions in neuroscience. To begin to address this issue, we previously developed multi probe imaging, namely dFOMA (dual FRET imaging with Optical Manipulation) imaging method for simultaneous measurements of two distinct biochemical signals, and four color, fast, sensitive and liner genetically encoded $Ca^{2+}$ indicator XCaMPs to measure neuronal activities of different cell types or different intracellular domains. dFOMA imaging demonstrated that CaMKIIalpha and calcineurin activations operated as distinct chemical decoding readouts of different parameters contained in the patterned neuronal input. To further explore this, an updated dFOMA2.0 method was generated by integrating brighter and more selective donor/acceptor FRET pairs, while also developing new/improved fluorescent probes for $Ca^{2+}$, CaMKIIalpha and calcineurin signaling. The new dual FRET imaging of CaMKII and calcineurin clearly demonstrated spatio-temporal activity difference between the kinase and phosphatase. In order to expand multiplicity of neuronal activity imaging, we expanded the color pallet of XCaMPs and the new $Ca^{2+}$ sensor permitted 6 color $Ca^{2+}$ imaging as well as more quantitative readout of intracellular $Ca^{2+}$ in the neurons. Finally, by combining a CaMKIIalpha FRET probe, a linearly performing red-color $Ca^{2+}$ indicator and pharmacological knockout approach, we developed disease phenotyping system that is selective, sensitive, quantitative platform for gaining functional insights into disease-causing rare gene mutations. This approach revealed the downstream biochemical activities are more relevant for abnormalities underlying the pathogenesis of neurodevelopmental diseases, and illustrates the power of advancing multiplex imaging of biochemical signaling to deciphering the molecular mechanisms underlying brain functions as well as the etiology of the neuropathological diseases.

### [1O07-02-02]

### Sustained ameliorative effects of levodopa on 6-OHDA-treated hemi-parkinsonism model rats by inhibiting neuroinflammation

*Mohammed E Choudhury[1], Noriyuki Miyaue[2], Masahiro Nagai[2], Junya Tanaka1[1] (*[1]Department of Molecular and Cellular Physiology, Ehime University Graduate School of Medicine, [2]Clinical Pharmacology and Therapeutics, Ehime University Graduate School of Medicine*)

We have lately reported that primary cultured rat microglia express dopamine (DA) receptors and that DA inhibits their LPS-induced inflammatory activation via D1R by elevating intracellular cAMP levels. In the present study, levodopa, a precursor of DA, was administered to 6-OHDA-induced hemi-Parkinson's disease (PD) model Wistar rats twice per day for 7 days. Levodopa suppressed microglial somata enlargement both in the striatum and the substantia nigra pars compacta of the PD model rats. Flow cytometry analyses revealed that levodopa abolished 6-OHDA-induced elevated CD11b and CD45 expression. Levodopa increased the expression of tissue repairing microglial factors, TGF-β1, bFGF, Arg1, and Ym1. Conversely, the expression of microglial proinflammatory factors, IL-1β, TNFα, and iNOS decreased even after levodopa treatment was discontinued. DA decreased the expression of iNOS by primary cultured microglia and increased their cAMP contents. Improved motor functions of 6-OHDA-treated rats was continuously observed even after levodopa administration was discontinued as revealed by forepaw adjustment steps (FAS) test and cylinder test, the most suitable tests for the hemi-Parkinsonism model. Furthermore, levodopa-treated rats showed improved social and cognitive functions as revealed by three-chamber test and Morris water maze (MWM) test. The results of this study indicate that the ameliorative actions of levodopa on PD are not solely attributed to DA replacement effects but also to anti-inflammatory effects on microglia that lead to prevention of DArgic neurons in the SNc. Thus, earlier administration of levodopa to PD patients may have better effects to prevent progressive loss of DArgic neurons, for which activated microglia should be responsible.

### [1O07-02-03]

### Behavioral task related information encoded in frontal cortico-striatal ensembles while a task progresses

*Takashi Handa[1,3], Tomoki Kurikawa[2,3] (*[1]Hiroshima University, [2]Future University of Hakodate, [3]RIKEN Center for Brain Science*)

The frontal cortex-basal ganglia network plays a pivotal role in adaptive goal-directed behaviors such as decision -making based on the outcome after its own action. However, it remains to be clarified what neural mechanisms across frontal cortex-basal ganglia circuit underlie the outcome-based decision making. In particular, little is known about how the neural ensembles in frontal cortex-basal-ganglia circuit encoded task-related information at single-trial level while adaptive behavior is ongoing. We previously demonstrated that neural ensembles in the medial frontal cortex (MFC) and dorsal striatum (DS), which is a main input nucleus of basal ganglia, of rats performing an outcome-based alternative choice task were temporally correlated. More synchronous activities between MFC and DS were represented in the rats proficiently performing task. However, it remains unclear how frontal-striatal ensembles encode task-related information along the progress of task, in other words at single-trial level. To address this question, we analyzed high dimensional ensemble activity consisting of both MFC and DS ensemble spiking activity during task through tensor component analysis (TCA). TCA is an unsupervised method to provide lower dimensional activity patterns and to enable us analyze dynamics of the ensemble activities at single-trial level. We found that the selectivity of outcome-based choices dynamically changed across trials and the selectivity correlated with behavioral variables such as reaction times and motivation-related behavior. TCA analysis unveiled differential ensemble activity between switching choice and repetitive choice. TCA components revealing outcome information, differences between reward and non-reward, were more strongly encoded in MFC-DS ensembles than in MFC or DS ensembles alone. Our results suggest that the MFC-DS ensembles reflect information related to outcome-based decision making along changes in behavioral variables along task progress.

### [1O07-02-04]

### Cross-species high-resolution cortical mapping of the face perception network in humans and macaques

*Takuro Ikeda[1], Takayuki Ose[1], Masahiro Ohno[1], Yuki Matsumoto[1], Takuya Hayashi[1,2] ([1]*RIKEN Center for Biosystems Dynaimc Research,* [2]*Kyoto University Graduate School of Medicine*)

Primates have evolved complex societies. Face perception is one of the most important elements of social interaction. Recent technical advances in neuroimaging have allowed the localization of functional areas associated with face perception. Previous studies have identified 6 separate areas along the superior temporal sulcus (STS) called face patches in macaques, whereas two areas in the occipito-temporal cortex in humans (fusiform face area [FFA] and occipital face areas [OFA]). However, it still remains unclear how macaque face patches correspond to human's FFA/OFA, primarily due to insufficient harmonization. To answer this question, we investigated the neural basis of social perception in two macaque monkeys (*Macaca mulatta*) using high-resolution fMRI during face perception. Four types of visual stimuli, comparable to those used in the Human Connectome Project (HCP), were presented to the animals: PLACE, TOOL, FACE, and BODY. Macaque MRI data were acquired using a 3T MRI scanner (MAGNETOM Prisma, Siemens, Germany) and 24ch RF coil designed for non-human primates (NHP) (Takashima Seisakusho, Japan) with a cortical thickness-based spatial resolution and high temporal resolution, as was adopted in HCP. The fMRI data was precisely mapped onto the cortical surface, thoroughly 'denoised' based on spatiotemporal features, and statistically analyzed for functional areas using an NHP-HCP pipeline. The same preprocessing was applied to HCP fMRI data to obtain functional maps (n=997). In macaques, we found distinct signals related to face perception in the dorsal and ventral banks of the STS, and signals related to TOOL in the inferior temporal cortex (IT). The human HCP data, signals related to face perception were found in the FFA, OFA, and STS. These results suggest correspondences between the macaque dorsal STS and the human STS, and between the macaque ventral STS and the human IT. No signals related to face perception were found in the macaque fusiform gyrus. These findings suggest that the area between the dorsal and ventral face areas (i.e., middle temporal area) is enlarged in humans compared to macaques, raising the possibility of a major functional reorganization of this area. Cross-species harmonized high-resolution functional imaging would be a critical method for understanding the neural basis of social perception and its evolution in primates.

### [1O07-02-05]

### Depression-like behaviors in mice underexpressing transforming growth factor-β1

*Masao Kakoki[1], Kensaku Nomoto[1], Kenji Kansaku[1] ([1]*Department of Physiology, Faculty of Medicine, Dokkyo Medical University*)

Loss-of-function mutations in the component genes of transforming growth factor-β (TGF-β) signaling lead to fatal aortic aneurysm syndromes (Loeys-Dietz Syndrome, LDS). Recent studies have suggested that patients with LDS have greater anxiety than controls (Heidi J et al. Am J Med Genet 2022). We previously noted elevated plasma levels of corticosterone in C57BL6/J mice with homozygous hypomorphic alleles (*Tgfb1L/L*), which express approximately 20 % amount of TGF-β1 protein of that in wildtype (WT)(Kakoki M et al. Proc Natl Acad Sci USA 2013). Glucocorticoid is a component of the stress response by hypothalamic-pituitary-adrenal axis and a pathogenic factor of depression. Indeed, chronic corticosterone (glucocorticoid in rodents) administration has been commonly used to experimentally induce depression-like phenotype. In the present study, we have performed two sorts of well-established behavioral tests for evaluating depression including sucrose preference test (SPT) and tail suspension test (TST) in the mice expressing genetically altered levels of TGF-β1. In SPT, *Tgfb1L/L* mice had markedly reduced sucrose preference as compared with WT (Preference index:*Tgfb1L/L* [n = 20] $0.67 \pm 0.02$ vs. WT [n = 22] $0.78 \pm 0.02$, $p < 0.001$ by Tukey's HSD test, $p < 0.001$ by 1-way ANOVA), whereas the sucrose preference of the other genotypes of mice was not significantly different from that of WT (Preference index:*Tgfb1L/+* [n = 14] $0.78 \pm 0.02$, *Tgfb1H/+* [n = 14] $0.78 \pm 0.02$, and *Tgfb1H/H* [n = 8] $0.79 \pm 0.03$). Likewise, *Tgfb1L/L* mice had significantly elongated immobility time in TST as compared with WT (Immobility time [seconds]; *Tgfb1L/L* [n = 7] $178 \pm 23$ vs. WT [n = 7] $104 \pm 22$, $p < 0.05$ by Student's t-test). Thus, we have found evidence that genetic insufficiency of TGF-β1 causes depression-like phenotype in C57BL/6 mice. The elevated corticosterone levels may play a pathogenic role in the development of the depression-like behavior in *Tgfb1L/L* mice. Our findings suggest that TGF-β1 and/or its downstream signaling could be the therapeutic target for treating depression.

### Oral

### [1O07-03]
### Higher brain function, Motor function

March 28, 14:20 - 15:20, Room 7

### [1O07-03-01]

### Emotional behavior in the kinase-dead knock-in mouse of calmodulin kinase IIα

*Yoko Yamagata[1], Yuchio Yanagawa[2] ([1]*National Institute for Physiological Sciences,* [2]*Gunma Univ. Grad. Sch. of Med*)

$Ca^{2+}$/calmodulin-dependent protein kinase IIα (CaMKIIα) is a key mediator of activity-dependent neuronal modification and involved in the molecular mechanisms of learning and memory. It is also implicated in the regulation of emotional behavior. Indeed, we revealed severe deficits in spatial memory in the Morris water maze tasks in the kinase-dead CaMKIIα (K42R)-KI mouse, and have been examining its emotional behavior as well. We found that the kinase-dead CaMKIIα-KI mouse showed decreased time spent in the center area in the open field locomotion test, indicating the avoidance of an open space. On the other hand, the KI mouse showed increased time spent in the open arms in the elevated plus maze test, indicating the preference for an open space. We additionally performed the light-dark transition test, and found that the KI mouse spent longer time in the light compartment compared to the wild-type (WT) mouse, indicating less avoidance of the light environment. Furthermore, in the Porsolt forced swim test, both genotypes showed increased immobility along the time course on the 1st day, and on the 2nd day, the WT mouse showed increased immobility from the beginning, whereas the KI mouse showed similar pattern of immobility as on the 1st day, indicating that the KI mouse did not remember the experience of the day before. These results suggest that in the absence of kinase activity of CaMKIIα, the expression of anxiety- and/or fear-related behavior is reduced, and severe deficits in learning and memory affects the outcome of emotional behavior.

### [1O07-03-02]

### Hippocampal CA1 neurons represent preceding experience: temporal dynamics of multiple-unit firings and rapid increase in information entropy per single ripple

*Dai Mitsushima[1,2], Junko Ishikawa[1], Takuto Tomokage[1] ([1]*Department of Physiology, Yamaguchi University Graduate School of Medicine,* [2]*The Research Institute for Time Studies, Yamaguchi University*)

Although the hippocampus processes spatiotemporal information and temporally encodes specific episodic experiences, the representation of episode-specificity and the temporal dynamics are unknown. We captured multi-neuronal spontaneous firings within hippocampal CA1 from freely behaving male rats which experienced various episodes for 10 min and continued recording for an additional 40 min. Spike width measurements suggested that the recorded data contained approximately 77% pyramidal cells and 13% interneurons. During the emotional experience, high-frequency firings that exceeded 3 SD of the pre-experience firings occurred repeatedly, followed by the alternating silent periods with no firings and ripple firings (short duration synchronous firings lasting 20 to 100 msec). The diversity of ripple firings expanded significantly in an episode-dependent and specific manner, increasing the information entropy per single ripple-firings. Moreover, the occurrence and the multiple features of ripple firings were significantly correlated with the occurrence of super bursts. Finally, analysis of synaptic strength using hippocampal slices showed significant episode specificity in synaptic diversity mediated by AMPA and $GABA_A$ receptors, suggesting episode-dependent and specific synaptic plasticity. These results provide evidence that the most recent prior experience is represented by the multi-neuronal firings and synaptic diversity of hippocampal CA1 neurons. Using deep learning with artificial intelligence, it may be possible to decipher the encoded representation of past experience contained in the diversity of ripple firings.

### [1O07-03-03]

### The Synthetic Synaptic Organizer CPTX Enables Rapid Recovery of Dexterous Hand Function after Spinal Cord Injury in Monkeys

*Stefan Peyda[1], Reona Yamaguchi[1,2], Satoko Ueno[1,3], Erika Omae[1], Kunimichi Suzuki[3,4], Veronica Chang[4], Hiroyuki Sasakura[5], Keiko Matsuda[3], Kousei Takeuchi[5], Radu Aricescu[4], Hirotaka Onoe[6], Michisuke Yuzaki[3], Tadashi Isa[1,2,6] ([1]*Department of Neuroscience, Graduate School of Medicine, Kyoto University, Japan,* [2]*Institute for the Advanced Study of Human Biology (WPI-ASHBi), Kyoto University, Japan,* [3]*Department of Neurophysiology, School of Medicine, Keio University, Japan,* [4]*MRC Laboratory of Molecular Biology, United Kingdom,* [5]*Department of Medical Cell Biology, School of Medicine, Aichi Medical University, Japan,* [6]*Human Brain Research Center, Graduate School of Medicine, Kyoto University, Japan*)

Hand movements are controlled by signals from the brain's primary motor cortex (M1) sent to motoneurons in the cervical spinal cord via the corticospinal tract (CST) and then relayed to the muscles. Physiologically, accurate neuronal circuit formation and function relies on "synaptic organizing proteins" (SOPs). The architecture of neuronal circuits is compromised by spinal cord injuries (SCI) which damage the CST and leads to impaired motor function. While currently SCI cannot be healed, a recent study by Suzuki et al. (*Science*, 2020) reported that CPTX, a synthetic SOP connecting pre-synaptic nerve terminals and post-synaptic excitatory AMPA-type glutamate receptors, can restore the communication between central neurons in mice. Promisingly, mice treated with CPTX after SCI regained the ability to walk. Whether CPTX can also restore hand function following SCI in primates is unknown.

To investigate this, we trained macaque monkeys to perform a reach-and-grasp task in which they retrieve small cubes of sweet potato from inside a slit by pinching it between the thumb and index finger. This “dexterous” type of grasping indicates very precise finger control. Also, using a modified Brinkman board task, monkeys were allowed to pick up as many potato cubes placed inside 25 vertical and 25 horizontal slots as possible within two minutes. Next, the monkeys were deprived of their hand function by sub-hemisection spinal cord injury surgery targeting the CST between cervical segments C6 and C7. One week later the monkeys were randomized to receive injection of either CPTX (total dose 25 μg) or buffer solution into the spinal cord at two depths in each of five sites surrounding the lesion. Surgeons as well as experimenters evaluating the tasks were blinded to the injected substance. After injection the tasks were re-started and the recovery was recorded until day 120 after SCI.

At the end of the experiment, unblinding revealed that monkeys treated with CPTX could recover the dexterous grasp earlier compared to the control monkeys. Further experiments with neuroanatomical analysis by tracing the CST axons would clarify the mechanism underlying the different phenotype between CPTX treated and control monkeys. To conclude, CPTX may be a potential treatment to faster recover hand function in spinal cord injury patients, reducing their suffering and rehabilitation time.

### [1O07-03-04]

### Dynamics of synaptic plasticity and acetylcholine secretion in layer V neurons of primary motor cortex by motor learning

*Hiroyuki Kida[1], Kawakami Ryousuke[2], Tsukamoto Fumimaro[1], Toyoshima Shotaro[1], Sakimoto Yuya[1], Mitsushima Dai[1] ([1]*Department of Physiology Yamaguchi University Graduate School of Medicine,* [2]*Department of Molecular Medicine for Pathology Ehime University Graduate School of Medicine*)

Layer V neurons in primary motor cortex (M1) are important for motor skill learning. Since pretreatment of layer V of M1 in rats with either CNQX or APV inhibits rotor rod learning, we analyzed training-induced synaptic plasticity by whole-cell patch-clamp technique using acute brain sections. 1-day trained rats showed a decrease in small inhibitory postsynaptic current (mIPSC) frequency and an increase in the evoked IPSC-to-pulse ratio, suggesting a transient decrease in presynaptic GABA release in the early phase. 2-day trained rats showed an increase in the amplitude/frequency of small excitatory postsynaptic currents (mEPSCs) and an increase in the AMPA/NMDA ratio, suggesting that AMPA receptors mediated excitatory synapses, suggesting a long-term enhancement of excitatory synapses. Importantly, rotor rod performance in trained rats correlated with the mean mEPSC amplitude and frequency obtained from those animals. Furthermore, live imaging analysis of Thy1-YFP transgenic mice showed that training rapidly recruited a substantial number of spines for long-term plasticity of M1 layer V neurons. Finally, we quantified acetylcholine secretion during motor learning in M1 by microdialysis and found that it is increased especially in the early learning period. Taken together, these results indicate that motor training induces complex and diverse plasticity in layer V pyramidal neurons in M1.

### [1O07-03-05]

### The latest in vivo technologies ensure the refinement of the principles of the 3Rs

*Shinichi Sato[1], Takashi Kanbayashi[3], Kyoichi Ono[2], Tsuguo Nishijima[1], Seiji Nishino[4]
([1]*Iwate Medical Univ.,* [2]*Akita Univ.,* [3]*Tsukuba Univ.,* [4]*Stanford Univ.*)

An animal experiment is a prerequisite in physiological education and research, which plays an essential role in understanding and elucidating the life phenomenon that contributes significantly to maintaining human health. However, we should keep the principles of the 3Rs in mind while planning and conducting animal experiments and be aware of the methods to ensure the refinement, i.e., minimizing pain, suffering, and distress. In this presentation, we list several noninvasive vital monitor systems for in vivo experiments, with some findings in the past two decades. The systems equipped with piezoelectric transducer or stripe electrode plate sensors can monitor heartbeat, electrocardiogram, breathing, or activity in freely behaving mice without causing pain and suffering. This nature of the systems is essential because pain and suffering can affect behavior, physiology, and immunology and can alter experimental results, which impairs both the reliability and reproducibility of studies.
By broadening the latest in vivo technologies, we hope the young physiologists feel free to do in vivo experiments due to the 3Rs.

### Oral

### [1O07-04]
### Membrane transport, Ion channels, Receptors

March 28, 15:20 - 16:20, Room 7

### [1O07-04-01]
### Advanced Analysis of Insulin Secretory Granule Dynamics in Response to Calcium Signaling

*Daisuke Ohshima[1], Yoshinori Mikami[1], Taichiro Tomida[1], Yuto Tei[1], Satomi Adachi-Akahane[1] ([1]*Department of Physiolosy, Faculty of Medicine, Toho University*)

**Background:** The insulin secretion from pancreatic β cells is precisely regulated by calcium signaling, mediated by voltage-dependent calcium channels (VDCCs). Precise modulation of calcium dynamics is crucial for optimal insulin release. However, the molecular mechanisms underlying these processes have not been fully understood.
**Objective:** This research aimed to elucidate the intricate interplay between calcium signaling and insulin secretory granule dynamics using a synergistic approach that combines molecular biology techniques with machine learning-based image segmentation and data-driven analysis.
**Methods:** We developed MIN6 cell lines engineered to express mCherry-labeled insulin together with the calcium indicator GCamp7. Using total internal reflection fluorescence microscopy (TIRF), we captured the detailed dynamics of labeled insulin in regions close to the cell membrane and the concomitant calcium elevation. Advanced machine learning algorithms were subsequently employed to accurately segment the insulin secretory granules. Our methodology allowed us to segment unlabeled granules using high-resolution time-lapse data from confocal microscopy.
**Results:** Our advanced approach effectively segmented insulin-secreting granules in both mCherry-labeled fluorescence images and newly acquired transmitted light images. Our analysis of the tracking data revealed a significant increase in the Max Speed and Distance Traveled of the secretory granules upon activation of calcium signaling. These findings revealed a part of the granule dynamics that lead to secretion. In addition, our methodology enabled accurate and high-throughput quantification of the dynamics of insulin-containing granules in response to calcium signaling, providing critical insight into the dynamics of insulin secretory granules.
**Conclusion:** By integrating advanced microscopy techniques with machine learning, our research has unveiled a novel method that delves into the subcellular dynamics of native insulin secretory granules. The underlying mechanism of the dynamics of insulin secretory granules in response to calcium signaling will be discussed.
**COI: NO**

### [1O07-04-02]
### Phospholipase D1 is essential for neutrophil extracellular trap formation.

*Takehito Uruno[1], Ryosuke Aihara[2] ([1]*Kyushu Univ.*, [2]*Kyushu Dental Univ.*)

Neutrophil extracellular trap (NET) is a fibrous net-like scaffold of decondensed chromatin released from activated neutrophils to trap and kill bacteria. Although NETs are important for host integrity, dysregulated NETs lead to pathological thrombosis associated with sepsis and autoimmune diseases. However, the molecular mechanism fine tuning NETs remains to be elucidated. Here, we show that phospholipase D1 (PLD1) and its product phosphatidic acid (PA) are crucial mediator of NET formation. In PLD1 deficient murine neutrophils, reactive oxygen species (ROS) production and NET formation induced by phorbol-12-myristate-13-acetate are greatly suppressed. A pharmacological inhibitor of PLD1 suppressed lipopolysaccharide (LPS)-induced NET formation in both murine and human neutrophils. In vivo, PLD1 deficiency greatly suppressed ROS production in the lung after intratracheal injection of LPS. Moreover, PLD1 deficient mice were protected from thrombus formation in an established model of deep vein thrombosis. These results indicate that PLD1 is essential for NET formation and serves as a potential therapeutic target for NET-related pathological thrombosis. (COI: NO)

### [1O07-04-03]
### Anomalous motions of soluble molecules in olfactory cilia

*Hiroko Takeuchi[1], Takashi Kurahashi[1] ([1]*Osaka University*)

Chemical information of odorants is converted into the electrical signal within the 100 nm diameter olfactory cilia. In the cilia, [cAMP]i is increased upon odor stimulation, which opens CNG channels, and, resultant $Ca^{2+}$ influx in turn activates excitatory Cl channels. During several decades of researches, physiologists observed current responses that were caused by the opening of those ion channels in the olfactory receptor cells. At the same time, it has long been whispered in the research filed that the time course of cell responses cannot be explained by a standard activity of second messenger molecules distributing in 10-μm-long cilia. In this study, we built a digital model that mimics the activities and macroscopic movement of molecules within the cilium. First, by using the model, the time course of the current was derived from standard activities of second messenger molecules, and was compared with that of the native cell response. After a transient increase in cytoplasmic cAMP in the digital model, the molecule in the cilia rapidly decayed to near zero levels within a few hundred milliseconds, whereas in native cilia the response lasted for more than a few seconds even with short pulses. The discrepancy of time courses has to be resolved because the response time is related to the signal amplification when we consider about the time integral of odor information; the number of action potentials is determined by the amplitude and duration of the odorant-induced depolarization. Next, in cell experiments, the diffusion coefficient of molecules in the cilium was measured using caged cAMP and Ca dye, and was shown to be about 1,000 times smaller than the value known for the free diffusion in solution (500 $\mu m^2$/s). In the digital model, we found that the binding between the molecule and cytoplasmic surface of the plasma membrane slows down diffusion considerably. This phenomenon became more pronounced when the diameter of the cilia model is reduced down to the nano-level. We verified that the long-lasting response time course found in the native cell was explained by a restriction in the diffusion of molecules that was caused by bindings of molecules to the cytoplasmic membrane surface when the parameters were set to physiologically reliable conditions.

### [1O07-04-04]

### Dual role of transmembrane S5 segment in the channel gating of type 1 ryanodine receptor (RyR1)

*Takashi Murayama[1], Nagomi Kurebayashi[1], Toshiko Yamazawa[2], Hideto Oyamada[3], Takashi Sakurai[1], Haruo Ogawa[4] (*[1]Juntendo University, [2]The Jikei University, [3]Showa University, [4]Kyoto University*)

The ryanodine receptor type 1 (RyR1) is a critical $Ca^{2+}$ release channel in the sarcoplasmic reticulum of skeletal muscle. RyR1 is a huge homotetrameric channel composed of six transmembrane segments (S1-S6). Among these segments, S6 forms the gate and pore, and the S4-S5 linker is believed to regulate this gate. S5, the fifth transmembrane segment connecting the S4-S5 linker and S6, is a locus for disease-causing mutations. However, the specific role of S5 in channel gating has remained unclear. To address this question, we conducted functional analyses of channels carrying disease-causing mutations in S5 using a heterologous expression system in HEK293 cells. Remarkably, mutations in the anterior part of S5 exhibited a gain-of-function phenotype, while those in the posterior part resulted in severe loss-of-function. In the context of the near-atomic structure, the mutated residues in the anterior part were found to contribute to interactions with S6, whereas those in the posterior part were involved in interactions with adjacent transmembrane segments, crucial for pore formation. Given that the disease-causing mutations were predicted to disrupt interactions with these key residues and that disease-causing mutations were identified in the interacting partners, we subsequently characterized mutants of these interacting partners. The phenotype of these mutants resembled that of disease-causing mutations in S5. Consequently, our findings suggest that S5 may play a dual role in RyR1 channel gating: the anterior part stabilizes the channel in the closed state, while the posterior part contributes to the formation of a stable pore.

### [1O07-04-05]

### Scaffold Protein PDLIM5 Regulates TRPC Mediated SOCE in Myoblasts

*Mingyi Dong[1], Andrés Daniel Maturana[1] (*[1]Nagoya University*)

In vertebrate, muscle fibers are formed through myogenesis. We aim at identifying essential regulating factors that transduce cellular signals during myogenesis. In myoblasts, TRPC channels mediate a store operated calcium ($Ca^{2+}$) entry (SOCE) to regulate intracellular $Ca^{2+}$ homeostasis during the myogenic differentiation. PDLIM5 (ENH1) proteins belong the PDZ-LIM scaffold protein family that organize signaling events including regulation of ion channels activity and genes expression in muscles. ENH1 scaffolds PKD with LIM2 domain and its PDZ domain binds to the L-type voltage-gated calcium channels (L-VCC) subunit, leading to functional Protein Kinase D1-ENH1-L-VCC complex in hypertrophic cardiomyocytes. ENH1 has also been shown to regulate the activity of voltage-gated sodium channels. Some TRPC members possess a PDZ-binding domain at their C-terminal domain suggesting a physical potential for interaction with scaffold proteins. We hypothesized a possible relationship between TRPC channels and PDLIM5 scaffold protein during myogenesis.

Thapsigargin-induced SOCE was monitored by $Ca^{2+}$ imaging, using Fluo8 calcium probe, in C2C12 myoblasts with ENH overexpression, TRPC inhibition and PKC inhibition. Thapsigargin-induced $Ca^{2+}$ influx was repressed by TRPC specific inhibitors, $Gd^{3+}$ (200 μM) and 2-APB (50 μM) confirming that the Thapsigargin-induced SOCE was TRPC dependent. Overexpressing ENH1 almost doubled TRPC-mediated $Ca^{2+}$ entry and the increased SOCE amplitudes could also be suppressed by $Gd^{3+}$ and 2-APB. Interestingly, the overexpression of ENH4, a short splice variant of ENH1 lacking the 3 LIM domains and specifically expressed in skeletal muscle, significantly repressed the TRPC-mediated $Ca^{2+}$ entry in myoblasts. A physical interaction between ENH1 and TRPC1 in C2C12 myoblasts was then confirmed by co-immunoprecipitation. Furthermore, the inhibition of the protein kinase C (PKC, Gö6983, 100 nM) in ENH1-overexpressed myoblasts blocked TRPC1-dependent $Ca^{2+}$ entry suggesting a PKC-ENH1-TRPC1 signal complex in myoblast.
This signal complex could function to regulate morphological changes of myoblasts into myotubes and providing us new possible targets for clinic treatment for muscle dysfunction and exercise-induced muscle damage.

### Oral

### [1O07-05]
### Sensory function

March 28, 16:30 - 17:30, Room 7

### [1O07-05-01]

### A recurrent cortical circuit triggers somatosensory perception

*Yasuhiro Oisi[1], Yusuke Atsumi[1], Yoshiki Ito[1], Saito Yoshihito[1], Hiroyuki Uwamori[1], Maya Odagawa[1], Takayuki Suzuki[1], Chie Matsubara[1], Shigeki Kato[2], Kazuto Kobayashi[2], Kenta Kobayashi[3], Midori Kobayashi[1], Atsushi Kobayashi[4], Kanako Ueno[1], Masanori Murayama[1] ([1]*RIKEN*, [2]*Fukushima medical Univ.*, [3]*NIPS*, [4]*NI*)

We believe that our brain makes us perceive the truth of the external world as it is. But in fact, it does not. When a mosquito perches on your leg, you may sometimes perceive and chase it away, but sometimes you do not perceive and suffer from itch afterward. How does the brain perceive stimuli? has been the most fundamental question in the study of perception. Recent theoretical studies have emphasized the important role of long range projections, including feedforward(FF) and feedback(FB) inputs. These theories are being tested by many studies of the neural correlates of perception in monkeys and humans. However, it remains unclear how such hierarchical interactions contribute to perception due to methodological limitations in dissecting and manipulating circuits precisely in time and space in primate research. We have previously reported a recurrent hierarchical circuit consisting of cortical long-range projections between the secondary motor cortex (M2) and the primary somatosensory cortex (S1) in mice (Manita et al., Neuron 2015). Furthermore, somatosensory stimulation sequentially induced activity in S1, M2, and S1 on the recurrent circuit. M2 FB input can trigger dendritic spikes and burst firing in S1 neurons. Based on these results, we hypothesized that the M2 FB projection to S1 contributes to somatosensory perception. Here, we tested this hypothesis using optogenetic, chemogenetic, pharmacological, and lesions of the circuit during a somatosensory stimulus detection task. We defined a perceptual detection threshold in each mouse that performed the behavioral task and investigated how the threshold changes with circuit manipulations. First, we found that S1 and M2 lesions, pharmacological and optogenetic inhibition of each area significantly increased the threshold, indicating impaired perception. Pathway-specific optogenetic and chemogenetic inhibition of both the S1->M2 FF and M2->S1 FB projections also impaired perception. These results suggest that the S1-M2 recurrent circuit contributes to perception via FF and FB inputs. Next, we tested whether activation of either FF or FB projections is sufficient for somatosensory perception. Pathway-specific optogenetic activation of both S1 FF and M2 FB projections was able to induce illusory somatosensory perception. Finally, we investigated which pathway is closely correlated with perception. Pathway-specific activation of M2 FB inputs with pharmacological M2 inactivation was able to induce illusory perception. In contrast, activation of the S1 FB input with pharmacological S1 inactivation impaired perception. These results support our hypothesis that somatosensory perception requires S1 activity that is evoked by recurrent M2 FB inputs.

### [1O07-05-02]

### Roles of N-linked glycosylation in mGluR6 cell surface delivery and interaction with ELFN1

*Takumi Akagi[1], Atsushi Shimohata[1], Ryota Takeda[1], Tomomi Sakamoto[1], Ikuo Ogiwara[1], Makoto Kaneda[1] ([1]*Nippon Medical School*)

Metabotropic glutamate receptor 6 (mGluR6) is specifically expressed in retinal ON bipolar cells (ON-BCs) and binds glutamate released from photoreceptors, thereby contributing to visual information processing. mGluR6 also interacts with trans-synaptic adhesion molecules and engages in synapse formation between ON-BCs and photoreceptors. The N-terminal extracellular domain (ECD) of mGluR6 serves as a glutamate binding pocket, and is also implicated in mGluR6 subcellular localization and interactions with synaptic adhesion molecules, such as extracellular leucine-rich repeat and fibronectin type III domain-containing 1 (Elfn1). Four sequons for N-glycosylation, Asn-Xaa-Ser/Thr (where Xaa is any amino acid except for proline), has been predicted at the N-terminal ECD of mGluR6. We herein examined whether N-glycosylation at the ECD is involved in regulating mGluR6 cell-surface localization and interactions with Elfn1, using deglycosylation assay, immunocytochemistry, flow cytometry and pull-down assay on 293T cells expressing mGluR6 ECD mutants with asparagine-to-glutamine (N-to-Q) substitution at the asparagine residues at the four N-glycosylation sequons (positions 290, 445, 473 and 561). We first found that all of the four predicted sites were N-glycosylated, and that impairment of N-glycosylation led to a reduction in total expression levels of mGluR6 as determined by deglycosylation assay and analysis with an N-glycosylation inhibitor. We next performed immunocytochemistry and flow cytometry, and found that mGluR6 cell-surface localization was slightly reduced by N-to-Q substitution at 290 and remarkable impaired by N-to-Q substitution at 445. We also found that N-to-Q substitution at 445 interfered the interaction of mGluR6 with Elfin1, whereas disruption of N-glycosylation at N473 and N561 facilitated the protein-protein interaction. These results suggested that N-glycosylation in the N-terminal intracellular ECD is required for mGluR6 cell surface delivery and modulates the interaction with trans-synaptic adhesion molecules between ON-BCs and photoreceptors.

### [1O07-05-03]

### Submodalities of intestinal chemical senses studied with *in vivo* $Ca^{2+}$ imaging of the nodose petrosal ganglia neurons

*Hikari Takeshima[1], Keisuke Ito[2], Hideki Enomoto[2], Takeshi Imai[1] ([1]*Department of Developmental Neurophysiology, Graduate School of Medical Sciences, Kyushu Univ,* [2]*Division for Neural Differentiation and Regeneration, Department of Physiology and Cell Biology, Kobe Univ.*)

After the food intake, the intestinal lumen not only digests and absorbs nutrients but also senses various chemicals. In the intestinal lumen, enteroendocrine cells (EECs) sense a variety of chemical signals including nutrients. These cells not only secrete gastrointestinal hormones but also make synaptic connections known as neuropods to the nodose petrosal ganglia (NPG) neurons. NPG neurons send the chemical signals to the nucleus tractus solitarii (NTS) of the brain. However, it remains poorly understood how NPG neurons process and convey the information of different chemicals detected at the intestinal lumen. In one scenario, different nutrients may activate the same sets of "generic" nutrient sensor neurons. In another scenario, different types of nutrients may be distinguished, as is known for the taste system, where various chemicals are grouped into five taste submodalities.

In this study, we performed *in vivo* $Ca^{2+}$ imaging of NPG neurons while the intestinal lumen of mice was stimulated with different types of nutrients. First, we found that NPG neurons that respond to liquid nutrients are distinct from intestinal stretch-responding population. We also found that different types of nutrients (i.e., sugar, amino acids, fat, and salt) are detected by distinct sets of NPG neurons. These results indicate that there are functional submodalities for nutrient sensation in NPG neurons, similarly to the taste system. Thus, different types of nutrients detected in the gut may induce distinct behavior and/or reflex through the NPG neurons.

### [1O07-05-04]

### Hindbrain neurons that drive drinking in response to oral water

*Yu Yamada[1], Kengo Nomura[1], Shogo Soma[1], Naofumi Suematsu[2], Akiyuki Taruno[1] ([1]*Kyoto Prefectural University of Medicine*, [2]*University of Pittsburgh*)

Water consumption is crucial for maintaining fluid balance in terrestrial animals; thus, water tastes pleasant when one is thirsty. Over the past decade, detailed neural mechanisms have been uncovered that control the motivation to drink water based on internal signals indicating fluid imbalance, such as plasma osmolarity. However, the neural substrates that facilitate drinking behavior in response to external signals, like the sensation of water or liquid in the oral cavity or pharynx, have yet to be explored. Here we demonstrate that a subset of hindbrain neurons responds to water and might play a key role in water drinking behavior.

First, we conducted a behavior test to compare preferences between water and silicone oil, a water-free liquid. The number of licks for water was greater than that of silicone oil, even when the viscosity was adjusted to be equivalent, indicating that mice can discriminate between water and water-free liquid, and prefer water. Subsequently, we hypothesized the existence of brain neurons that selectively respond to oral water and facilitate drinking behavior, which can be referred to as “water taste neurons”. To find candidate brain regions, we searched for activated brain areas in response to water intake after water deprivation across the entire brain using c-Fos immunoreactivity. We focused on a subregion of the parabrachial nucleus (PBN), because the region was activated in response to water intake but not water deprivation per se, and it receives various sensory inputs from gustatory, vagal, and somatosensory afferents. To further clarify the response characteristics of the PBN neurons during water ingestion, we performed *in vivo* $Ca^{2+}$ imaging experiments on head-fixed, awake behaving mice. Notably, a subset of the PBN neurons were activated immediately after the onset of water-licking but not silicone oil, implying that the neurons receive water-related signals other than somatosensory signals from oral cavity or pharynx. Furthermore, acute optogenetic inhibition of the PBN decreased the lick number of water without increasing access latency to water, suggesting that the PBN neurons control water drinking behavior but not the motivated behavior to access water. These results suggested that putative water taste neurons in the PBN may play a crucial role in water drinking behavior driven by chemosensory information of water.

### [1O07-05-05]

### Regional cerebral blood flow responses to trigeminal olfactory stimulation

*Daichi Morihara[1,2], Jura Moriya[1,2], Fusako Kagitani[1], Sae Uchida[1] ([1]*Department of Autonomic Neuroscience, Tokyo Metropolitan Institute of Gerontology*, [2]*Tokyo University of Agriculture and Technology*)

Olfactory dysfunction is an early symptom of Alzheimer’s disease (AD). Basal forebrain cholinergic neurons, which degenerate in AD, project to the neocortex and olfactory bulb. Information about the sense of smell is conveyed by the olfactory nerve, or by the trigeminal nerve when irritating odors. We recently reported that olfactory nerve stimulation increases regional blood flow in the olfactory bulb, and the activation of nicotinic acetylcholine receptors (nAChRs) potentiates olfactory bulb blood flow responses. This study aimed to clarify 1) the effects of intranasal trigeminal stimulation on regional cerebral blood flow, 2) the effects of nAChR activation on these blood flow responses. Using anesthetized rats, regional blood flow in the olfactory bulb and frontal cortex was measured by laser Doppler flowmeter or laser speckle contrast imager. Arterial blood pressure was simultaneously recorded. Intranasal trigeminal nerve was stimulated electrically. A nAChR agonist, nicotine, at a dose of 30 µg/kg was intravenously injected.Intranasal trigeminal nerve stimulation increased blood flow in both olfactory bulb and frontal cortex with pressor response. In rats spinalized at the upper thoracic level, the intranasal stimulation did not elevate blood pressure and olfactory bulb blood flow, but increased blood flow in the frontal cortex. Intravenous injection of nicotine did not influence the olfactory bulb blood flow but augmented the increased blood flow in the frontal cortex following the intranasal trigeminal nerve stimulation.The present study revealed that 1) stimulation of the intranasal trigeminal nerve itself did not affect the olfactory bulb blood flow while it increased blood flow in the neocortex, 2) activation of nAChRs did not influence the olfactory blub blood flow while it potentiated the neocortical blood flow responses to intranasal trigeminal nerve stimulation. These findings suggest that the olfactory information transmitted via the different neuronal pathways, olfactory and trigeminal nerves, is processed in different regions in the brain. Moreover, activation of nicotinic cholinergic transmission potentiates both olfactory neural pathways.

### Oral

### [1O07-06]
### Autonomic nervous system

March 28, 17:30 - 18:30, Room 7

### [1O07-06-01]

### Activity patterns of the digastric, masseter, diaphragm, and abdominal muscles during hiccup-like movements induced by stimulation of Phox2B-positive neurons in the dorsal brainstem

*Makito Iizuka[1], Keiko Ikeda[2], Hiroyuki Igarashi[3], Kazuto Kobayashi[4], Hiroshi Onimaru[1], Masahiko Izumizaki[1] ([1]*Dept Physiol, Showa Univ Sch Med, Tokyo, Japan,* [2]*Dept Oral Physiol, Showa Univ Sch Dent, Tokyo, Japan,* [3]*Dept Physiol Pharmacol, Schulich Sch Med Dent, Robarts Res Inst, Western Ontario Univ, Canada,* [4]*Dept Molecular Genetics, Inst Biomed Sci, Fukushima Med Univ Sch Med, Fukushima, Japan*)

Previously, we found that blue light stimulation of the dorsal skull of transgenic neonatal rats in which Phox2B-positive neurons expressed one of the channelrhodopsin variants, ChRFR (C167A), caused sucking-like and hiccup-like movements under both conscious free-moving and lightly anesthetized sedated conditions. Hiccup-like movements are characterized by a depression in the thorax in phase with brief activity in the diaphragm. A feature defining hiccups is the inhibition of the abdominal wall muscle activity during the activity of the diaphragm. In this study, therefore, we additionally recorded the activity of the abdominal muscles and confirmed that hiccup-like movement induced by light are distinct from spasms observed in disinhibition. A detailed analysis was conducted on the differences between the hiccup-like movement and normal respiration. Hiccup-like movements were observed in 30 out of 86 trials (in 9 out of 11 rats). One trial from each rat was selected for analysis. The average period of hiccups was 0.88±0.38 sec (range: 0.36–1.28 sec), while the average period of resting respiration was 0.87±0.19 sec (range: 0.61–1.25 sec). There was no significant difference between the two (paired t-test, P=0.93). Using the averaged leaky-integrated waveform (time constant 0.01 sec), we measured parameters such as the duration of activity. The average duration of diaphragm activity during hiccup-like movements was 0.08±0.02 sec, which was significantly shorter than the duration of diaphragm activity during resting inspiration (0.23±0.05 sec, P<0.0001). The amplitude of the integrated diaphragm waveform during hiccup-like movements was larger, being 4.1±1.2 times that of the diaphragm activity during resting inspiration (n=9). Abdominal wall muscles often showed increased expiratory activity upon light stimulation, with no activity consistent with hiccup-like movements observed in all trials, and clear inhibition was observed in 4 out of 9 trials. Based on these results, we concluded that the hiccup-like movements induced by light were indeed hiccups.

### [1O07-06-02]

### Activation of a subclass of vagal afferent nerves through oxytocin treatment reduces anxiety and enhances social interaction by stimulating oxytocin neurons in the hypothalamic paraventricular nucleus.

*Kengo Iba[1], Yuta Masuda[1], Rika Kitano[1], Kento Ohbayashi[1], Yusaku Iwasaki[1] ([1]*Laboratory of Animal Science, Graduate School of Life and Environmental Sciences, Kyoto Prefectural Univ.*)

COVID-19 pandemic triggers a 25% increase in the worldwide prevalence rate of anxiety and depression. The central oxytocin (Oxt), which synthesized in the neurons of hypothalamic paraventricular nucleus (PVH) and supraoptic nucleus, is involved in the regulation of not only feeding behavior but also mental functions such as anxiety and sociality. Therefore, the Oxt neurons are a potential target for treating hyperphagic obesity and mental illness. However, there is currently no technology available to effectively activate Oxt neurons without causing side effects. In previous study (Y. Iwasaki et al., **BBRC** 2019), we have demonstrated that intraperitoneal (IP) administration of oxytocin activates PVH Oxt neurons via activation of vagal afferents expressing oxytocin receptors, thereby ameliorating hyperphagic obesity and diabetes in mice without inducing side effects. However, it remains unclear whether this peripheral-to-central coupling of Oxt, mediated by vagal afferent nerves, is also effective in regulation of mental functions. In present study, we examined whether peripheral oxytocin administration could regulate mental function such as anxiety and sociality via the Oxt's peripheral-to-central relay.

We measured anxiety behavior using the elevated plus maze and social behavior using the three-chamber social interaction test in C57BL/6J male mice. IP administration of Oxt one hour before the behavioral test significantly reduced anxiety-related behaviors and increased social behaviors. These effects are blunted by subdiaphragmatic vagotomy. Ip injection of Oxt activated PVH-Oxt neurons via vagal afferent neural pathway. When the neuronal activity of Oxt neurons in PVH was specifically and artificially suppressed using chemical genetic DREADD techniques, the anxiolytic and social promoting effects mediated by ip administration of Oxt were abolished. Furthermore, intraventricular administration of an oxytocin receptor antagonist completely inhibited the Oxt-induced psychotropic effects.

In conclusion, we demonstrate that the relay of peripheral Oxt to PVH Oxt neurons via vagal afferents reduces anxiety and enhances social interaction. This pathway holds promise for the development of psychotropic medications or functional foods with minimal side effects, which could contribute to better mental health.

### [1O07-06-03]

### Intestinal GLP-1 and pancreatic insulin enhance insulin action through cooperative action at the common hepatic branch of vagal afferents

*Kento Ohbayashi[1], Toshihiko Yada[2], Yusaku Iwasaki[1] ([1]*Laboratory of Animal Science, Graduate School of life and Environmental Sciences, Kyoto Prefectural University, Kyoto, Japan,* [2]*Division of Integrative Physiology, Kansai Electric Power Medical Research Institute, Kobe, Japan*)

[Background] Glucagon-like peptide-1 receptor agonists (GLP-1RAs) directly act on pancreatic beta-cells to enhance glucose-induced insulin secretion and effectively ameliorate hyperglycemia. On the other hand, endogenous GLP-1 secreted from the intestine is highly unstable, therefore its physiological functions have not been fully elucidated. We have previously found that stimulation of intestinal GLP-1 release with the rare sugar D-allulose (Allu) enhances insulin-induced blood glucose lowering effect (Y. Iwasaki, *Nat Commun*, 2018). However, the detailed mechanisms remain unproven. Here, we investigated the effects of intestinal GLP-1 on glucose metabolism and the underlying mechanisms.

[Results] A single peroral (po) administration of Allu promoted GLP-1 secretion, however, did not alter blood glucose and plasma insulin levels in healthy and type 1 diabetic Akita mice. In type 2 diabetic (diet-induced obesity or *db/db*) mice with hyperglycemia and hyperinsulinemia, po Allu significantly ameliorated hyperglycemia without increasing insulin secretion, suggesting enhanced insulin action. These beneficial effects were blunted by the genetic or pharmacological inhibition of the GLP-1R. Comparing these results suggest that intestinal GLP-1 enhances insulin action in a manner dependent on plasma insulin levels. Exogenous insulin injection after oral Allu administration potentiated insulin-induced blood glucose lowering effect in Akita mice. Furthermore, co-administration of Allu and sulfonylurea drug, to induce simultaneous secretion of GLP-1 and insulin, enhanced activation of vagal afferent neurons and potentiated the hypoglycemic effects of sulfonylurea drug. These effects were completely abolished by chemical denervation of the common hepatic branch of vagal afferents. Finally, endogenous GLP-1 release by Allu more rapidly and potently ameliorated insulin resistance and hyperglycemia in *db/db* mice compared to GLP-1RA.

[Conclusion] Intestinal GLP-1 and pancreatic insulin work in concert to act on the common hepatic branch of vagal afferents, thereby enhancing insulin action and ameliorating hyperglycemia.

### [1O07-06-04]

### Analysis of the central mechanism of the anorexigenic effect of rare sugar D-allulose

*Rika Kitano[1], Tenko Shimizu[1], Yuta Masuda[1], Kento Ohbayashi[1], Yusaku Iwasaki[1]
([1]*Laboratory of Animal Science, Graduate School of Life and Environmental Sciences, Kyoto Prefectural University*)

The obesity pandemic is a serious worldwide problem and cause a several diseases such as type-2 diabetes and cardiovascular. Overeating is a cause of obesity. However, there are very few safe and effective medications or food ingredients available to reduce appetite.
In the previous study, we found that peroral (po) administration of D-allulose (Allu), a rare sugar existing only a small amount in nature, suppresses food intake without causing adverse effects, thereby ameliorating obesity and diabetes (Y. Iwasaki et al. **Nat Commun** 2018). This mechanism is closely linked to the release of glucagon-like peptide-1 (GLP-1), an intestinal hormone, through the administration of Allu, and the stimulation of the vagal afferent nerves by GLP-1. However, it remains unclear which specific neurons within the central nervous system regulate food intake through this activation of vagal afferents. In the present study, we examined the hypothalamic neurons activated by Allu administration in mice and investigated whether these neurons are involved in the regulation of feeding.
We performed immunostaining for c-Fos in the hypothalamus following a single administration of Allu, which is a neural activating marker. Allu increased the number of neurons immunostained for c-Fos in the paraventricular nucleus (PVH) and the supraoptic nucleus (SON) of hypothalamus. The elevation of c-Fos expression was significantly reduced by the knockout of GLP-1 receptors or subdiaphragmatic vagotomy. On the other hand, Allu significantly increased the mRNA expression of vasopressin in the hypothalamus, which is a neuropeptide expressed in both the PVH and SON. Furthermore, Allu increased c-Fos expression in the vasopressin neurons in both the PVH and SON using the technic of double-immunostaining for c-Fos and vasopressin. Finally, intracerebroventricular preadministration of a vasopressin receptor antagonist completely blunted the decrease in food intake caused by Allu administration.
These results demonstrate that Allu-induced GLP-1 secretion activates the vasopressin neurons in the hypothalamus via vagal afferent nerves, thereby suppressing food intake.

### [1O07-06-05]

### Measurement of hypothalamic neuron activity triggered by the rare sugar D-allulose using fiber photometry technique.

*Yuta Masuda[1], Rika Kitano[1], Kento Ohbayashi[1], Yusaku Iwasaki[1] ([1]*Laboratory of Animal Science, Graduate School of Life and Environmental Sciences, Kyoto Prefectural University*)

Glucagon-like peptide-1 receptor (GLP1-R) agonists are known as anti-diabetic and anti-obesity drugs because they enhance glucose tolerance by promoting glucose-induced insulin secretion and reduce food intake. The GLP-1 receptor agonists are stable compounds *in vivo* and exhibit effective medicinal efficacy, however, they are frequently reported to have side effects including vomiting, nausea, and an increased heart rate. In contrast, endogenous intestinal GLP-1 is highly unstable due to the influence of degrading enzymes expressed in various organs. In the previous study, we demonstrated that GLP-1 secreted in response to D-allulose (Allu), a rare sugar and zero-calorie sweetener, effectively suppressed food intake in mice without causing adverse effect, thereby ameliorating hyperphagic obesity and diabetes (Y. Iwasaki et al., Nat Commun 2018). Furthermore, we have preliminarily found that Allu-induced GLP-1 secretion activates vasopressin (AVP) neurons in the paraventricular nucleus (PVH) and supraoptic nucleus (SON) in the hypothalamus. In present study, to investigate at which time the AVP neurons are activated after Allu administration, we sequentially measured the neural activity of AVP neurons in the PVH or SON in freely-moving mice using a fiber photometry system.AAV inducing the expression of GCaMP in a Cre-dependent manner was unilaterally micro-injected into the PVH or SON in AVP-Cre mice. The Allu solution (3 g/10 ml/kg) or saline (10 ml/kg) was administered into the stomach directly using a stainless-steel feeding needle, then, the fluorescence of GCaMP in the AVP neurons was sequentially recorded from 15 min before PO administration of Allu at 3 g/kg until 120 min afterward. We calculated $\Delta F/F$, which is ($F$ at the time) / ($F$ at baseline) × 100 (%).Cytosolic $Ca^{2+}$ signals in the PVH immediately increased and peaked at 5 min ($\Delta F/F$ was approximately 75%). The elevated signal slowly diminished over 90 min and returned to baseline. On the other hand, the $\Delta F/F$ in the SON also increased promptly and peaked at 10 min (approximately 80%). However, the increased $\Delta F/F$ remained at a high level of 50% even after 120 min, although it gradually decreased. There results suggest that PO Allu immediately activates both the AVP neurons in PVH and SON. The AVP neurons in the PVH transiently responded to Allu, while those in the SON continuously responded to Allu.

### Oral

### [2O07-01]
### Sensory function, Development

March 29, 8:50 - 9:50, Room 7

### [2O07-01-01]

### Collection of special extracellular fluid of the inner ear and elucidation of specific proteins in the fluid for overcoming hearing loss

*Masatoshi Fukuda[1,2], Hiroki Okanisihi[3], Daisuke Ino[1], Takashi Sato[2], Yumi Ohta[2], Hidenori Inohara[2], Yoshikatsu Kanai[3], Hiroshi Hibino[1] ([1]*Division of Glocal Pharmacology, Department of Pharmacology, Graduate School of Medicine, Osaka University, Suita, Osaka, Japan,* [2]*Department of Otorhinolaryngology, Graduate School of Medicine, Osaka University, Suita, Osaka, Japan,* [3]*Department of Bio-syastem Pharmacology, Graduate School of Medicine, Osaka University, Suita, Osaka, Japan*)

More than ten million people are suffering from hearing impairment in our country. Therefore, uncover its causes and develop treatment methods are our urgent task. Many cases of intractable hearing impairment are associated with disorders in the cochlea of the inner ear.The cochlea consists of three luminal spaces, two of which are filled with perilymph, exhibiting ion concentrations similar to plasma. On the other hand, the remaining one is filled with endolymph, which constantly shows high $K^+$ concentration of 150 mM and a high potential of +100 mV. This unique ion-electric environment forms the foundation of sensory cell sensitivity.However, due to its distribution within a confined space (just 1 µL in a mouse cochlea), detailed analysis of its properties has been nearly impossible.Hence, this study aimed to develop a method for collecting the ultimate bodily fluid, the inner lymphatic fluid, from live mice, and moreover to find out the key molecules for hearing loss.A micro pipette filled with conductive organic solvent was inserted into the cochlea, allowing real-time measurement of cochlear potential. After ensuring the high potential of the endolymph, samples were aspirated into the pipette. As a result, purified inner lymphatic fluid was successfully recovered from a single mouse cochlea. The endolymph samples were then compared to the perilymph fluid. As a result, we have identified some specific proteins that is not found in the perilymph fluid.In the future, we can anticipate significant progress in understanding the pathophysiology of hearing impairment.

### [2O07-01-02]

### Endocardially-derived tissue macrophages alleviate calcification of the heart after birth

Mei Wang[1], Norika Liu[1,2], Susumu Minamisawa[1], *Atsushi Nakano[1,2] ([1]*The Jikei University School of Medicine Cell Physiology,* [2]*California University Los Angeles*)

**Objectives:** Tissue macrophages proliferate locally, settle in adult tissues, and play various roles in maintaining homeostasis of tissues including the prevention of tissue calcification as a source of osteoclast. It is reported that macrophages derived from different origins contribute to different function. Macrophages mainly originate from the embryonic yolk sac or monocytes, but our group (and others) has found that macrophages also derive from Nkx2-5+ endocardial cells in fetus, known as endocardially-derived tissue macrophages (EcTM). However, the role of EcTM in the prevention of calcification in the cardiovascular system is not fully understood. This research seeks to uncover the role of EcTM in the development of calcification in postnatal hearts.
**Methods:** Histological analysis of adult wild-type and EcTM-deficient (Nkx2-5-cre; Csf1r-fl/fl) mice were performed to confirm the localization of microphage (marker: F4/80) and osteoclast (marker: ACP5) To test the development of calcification in vivo, adult wild-type and EcTM-deficient mice were subjected to calcification stimulation (Warfarin-containing diet for 4 months), and then calcified lesions were quantified with Alizarin Red staining. To understand the transcriptional profiling of EcTM in more detail, single-cell RNA sequencing (scRNA-seq) data of mouse hearts from different ages were analyzed.
**Result:** Immunohistochemistry of the hearts for the osteoclast marker Acp5 revealed a marked decrease in osteoclasts in the hearts of EcTM-deficient mice compared to wild-type mice. EcTM-deficient hearts exhibited more calcified lesions, predominantly at vascular lesions, than wild-type mice. The signal network analysis of scRNA-seq predicted that the EcTM subcluster produces Tumor necrosis factor α (Tnfα) that is required for osteoclast generation as an autocrine signal.
**Conclusion:** Our data suggested that EcTMs contribute to osteoclasts in adult mice that prevent calcification in the heart. scRNA-seq data suggested that TNFα may autonomously play a role in generating osteoclasts. Our study sheds light on the mechanisms how macrophages of different origins impact the maintenance of homeostasis and the pathophysiological processes of tissues.

### [2O07-01-03]

### Expression of matricellular protein in the mouse inner ear

*Kazuya Ono[1], Takeru Ota[1], Hiroshi Hibino[1] ([1]*Laboratory for Pharmacology Osaka University Graduate School of Medicine*)

The sensory epithelium of the cochlea, which transduce sounds into neural signals, consists of hair cells, supporting cells, and the extracellular components such as tectorial membrane that covers the epithelial zone. Sound stimulation causes the sensory epithelium to vibrate, which bends the hair bundle of hair cells. As a result, the mechanoelectro transduction (MET) channels located on the hair bundles open, leading to the influx of cations and excitation of the hair cells. The tectorial membrane is essential for proper vibration of hair cells. Despite elaborate structure, the molecular mechanisms underlying formation of cochlear components remains largely to be understood. In this study, we comprehensively compared RNA in the cochlea of newborn and adult mice. We identified Smoc2 as a gene whose expression peaks around the time of birth. Smoc2 encoding a matricellular protein is involved not only in extracellular matrix formation but also in cell differentiation by antagonizing BMP signal.  Histological analysis revealed that Smoc2 mRNA isexpressed in hair cells and supporting cells that produce the tectorial membrane during development. These results suggest that Smoc2  may be important for tectorial membrane and hair cell formation.

### [2O07-01-04]

### Role of expression of Transglutaminase 2 in the zebrafish retina just after optic nerve injury.

*Kayo Sugitani[1], Takumi Mokuya[1], Yuya Omori[1], Yu Kanai[1], Yurina Takaya[1] ([1]*Div Health Sci, Grad Sch Med Sci, Kanazawa Univ.*)

Unlike in mammals, fish central nervous system (CNS) neurons can regrow axons and restore their function after nerve injury. We have identified many nerve regeneration-associated molecules in the fish retina using zebrafish optic nerve injury models. Here, we specifically focused on molecules whose expression is upregulated within 24 hours after optic nerve injury. Among them, Transglutaminase 2 (TG2), well known as a protein cross-linking enzyme, was found to be upregulated in the retina at 30 minutes after the earliest optic nerve injury. In addition, TG2 showed increased enzyme activity in the injured optic nerve regions. A similar upregulation of Heat Shock Factor 1 (HSF1) was also observed in the injured retina.To investigate the role of TG2 expression after optic nerve injury, TG2-specific morpholino (MO) was used to suppress TG2 expression. Injection of TG2-MO into the eye not only suppressed TG2 expression after optic nerve injury but also suppressed HSF1 expression. Previous studies have shown that HSF1 expression plays an important role in the expression of the Yamanaka factors (Klf4, Oct4, Sox2) in the injured retina. The serial activations of HSF1-Yamanaka factors protected retinal cells and maintained their viability after optic nerve injury stress. These findings suggest that rapid TG2 expression is involved in HSF-1-mediated expression of the Yamanaka factors, which also affects cell survival and stem cell conversion in the injured retina.

### [2O07-01-05]

### Muscle-resident mesenchymal progenitors regulate the ectopic fat accumulation and muscle regeneration via a cilia-dependent manner

*Daishi Yamakawa[1], Kosuke Kasahara[1], Yasuko Bando[1] ([1]*Dept. of Mol Phys and Cardiovasc Biol, Graduate School of Medicine, Mie Univ*)

Skeletal muscle is composed of multinucleated contractile myofibers, which are formed during development by the proliferative growth and fusion of mononucleated muscle cells. During post-natal growth, the number of myofibers remains constant, and skeletal muscle can regenerate after injury. The potency of skeletal muscle regeneration depends primarily upon myogenic stem cells called satellite cells. The myogenic program also requires functional cross-talk between satellite cells/myoblasts and other resident cells in the skeletal muscle niche such as mesenchymal progenitors. After injury, skeletal muscle regenerates but fatty tissue accumulation is seen in aged muscle or muscular dystrophies. Mesenchymal progenitors are key players in these events; however, the effect of primary cilia on mesenchymal progenitors remains unclear. Here, it is reported that genetic ablation of trichoplein (TCHP), a ciliary regulator, induces ciliary elongation on mesenchymal progenitors after injury, which promotes muscle regeneration while inhibiting adipogenesis. The defective adipogenic differentiation of mesenchymal progenitors is attributed to cilia-dependent lipid raft dynamics dysfunction, which is critical for insulin/Akt signaling. It is also found that interleukin (IL) 13 is substantially produced by intramuscular mesenchymal progenitors, which are upregulated by ciliary elongation and contribute to regeneration. Mechanistically, long cilia excessively activate the IL33/ST2/JNK axis to enhance IL13 production, facilitating myoblast proliferation and M2 macrophage polarization. The results indicate that mesenchymal progenitors organize the regenerative responses to skeletal muscle injury via cilia-mediated insulin/Akt and ST2/JNK signaling pathways.

### Oral

### [2O07-02]
### Digestive system, Immunity, Blood

March 29, 9:50 - 10:50, Room 7

### [2O07-02-01]
### Effect of aging on claudin-8 expression and paracellular amino acid flux in mouse colonic MCE301 cells

*Akira Ikari[1], Ema Okamoto[1], Shunsuke Matsuda[1], Yuta Yoshino[1], Yoshifumi Morikawa[2], Koichi Suenami[2], Yoshiaki Tabuchi[3], Toshiyuki Matsunaga[1] ([1]*Gifu Pharmaceutical University,* [2]*Gifu Prefectural Police Headquarters,* [3]*University of Toyama*)

The dietary proteins are broken into di/tri-peptides and amino acids (AAs), which are absorbed by specific transporters in the small intestinal and colonic epithelial cells. Tight junctions (TJs) are constructed between neighboring cells and prevent free paracellular fluxes of mineral ions and aqueous molecules. However, it is unknown whether the TJs are involved in the regulation of paracellular fluxes to AAs. The paracellular fluxes are controlled by claudins (CLDNs), which comprise a family of over 20 members. In the present study, we found that CLDN8 expression is downregulated by AAs deprivation in mouse colon-derived MCE301 cells. The reporter activity of CLDN8 was not significantly changed by AAs deprivation, whereas the stability of CLDN8 protein was decreased. MicroRNA analysis showed that AAs deprivation increases miR-153-5p expression which targets CLDN8. The AAs deprivation-induced downregulation of CLDN8 expression was rescued by a miR-153-5p inhibitor. The CLDN8 silencing enhanced the paracellular fluxes to AAs, especially middle molecular size AAs. The expression levels of colonic CLDN8 and miR-153-5p in aged mice were lower and higher than those in young mice, respectively. We suggest that AAs deprivation downregulates CLDN8-dependent barrier function, mediated by the elevation of miR-153-5p expression in the colon, in order to compensate AA imbalance.

### [2O07-02-02]
### Muscular $Ca^{2+}$ activity and its regulation underlying region-specific movement of the colon

*Shinsuke Nakayama[1], Chiho Takai[1], Naoko Iwata[1] ([1]*Nagoya University Graduate School of Medicine*)

The colon displays region-specific characteristic movements complying with its functional state. In the mouse colon, the proximal region of the tract allows food residuals to move forward and backward, absorbing fluid and shaping faeces, while the middle/distal regions generate the propulsive movement of large amplitude, referred to as the colonic motor complex (CMC) propagating through the tract. The propulsive movement of the colon is thought 'neurogenic', nevertheless it is unclear how the enteric nervous system generates CWCs.
In this study, to monitor intracellular $Ca^{2+}$ ($[Ca^{2+}]_i$) in muscle bundles of the colon, we used transgenic mice, which selectively expressed a $Ca^{2+}$ indicator YC-Nano50 protein in smooth muscle cells by targeting the parvalbmin gene. The FRET-based measurement of the YFP/CFP fluorescence ratio revealed that basal $[Ca^{2+}]_i$ was maintained in a similar level throughout the tract in musculature samples containing enteric neurons and pacemaker interstitial cells. In the proximal region $[Ca^{2+}]_i$ continuously oscillated at an interval of approximately 4 s. On the other hand, in the middle/distal regions $Ca^{2+}$ wave complexes (CWCs) occurred intermittently. $[Ca^{2+}]_i$ increased slowly from the basal level to a threshold, followed by a train of $Ca^{2+}$ transients that persisted for tens of seconds. In the same samples used in $[Ca^{2+}]_i$ measurement, microelectrode array detected spontaneous electric activities essentially similar to colonic musculature samples in wild-type mice. Simultaneous motion tracking of the fluorescence images characterized the features of movement: slow rhythmic movement in the circular muscle direction greater than or equivalent to the longitudinal muscle direction in the proximal region and incomplete tetanic contractions with the magnitude greater in the longitudinal direction. The application of TTX decreased the basal $[Ca^{2+}]_i$ throughout the tract, and attenuated $Ca^{2+}$ oscillations in the proximal region and abolished CWCs in the middle/distal regions. The results indicated that the origin of rhythmicity was attributed to pacemaker cells in the proximal region and to smooth muscle itself in the middle/distal regions.

### [2O07-02-03]
### Loss of PYK2 in melanoma suppresses PD-L1 expression and improves anti-tumor immunity

*Yuto Mizuno[1], Masanari Umemura[1], Chihiro Hayashi[1], Humina Suzuki[1], Soichiro Ishikawa[1], Akane Nagasako[1], Yukie Yamaguchi[1], Yoshihiro Ishikawa[1] ([1]*Yokohama City University*)

[Introduction] Programmed cell death ligand 1 (PDL1) expression in cancer aids immune evasion. While inhibiting PDL1 is an effective anticancer strategy, the regulatory mechanisms of its expression remain unclear. PDL1 is typically expressed at low levels but can be induced by inflammatory cytokines like IFNγ. This study reveals the role of Proline-rich tyrosine kinase 2 (PYK2) in immune elimination of cancer and its importance in IFNγ-induced PDL1 expression. Furthermore, inhibition of PYK2 led to strong tumor rejection by $CD8^+$ T cells. [Materials and methods] The C8161 (human melanoma cell line) was utilized. VS6063 and TAE226 were used as PYK2 inhibitors. Melanoma RNAseq data (TCGA-SKCM) were soursed from The Cancer Genome Atlas (TCGA). Real-time qPCR, western blotting (WB) analysis and Killing Assay were conducted. The Killing assay was performed by co-culturing T cells and cancer cell lines to assess the disruption of cancer cells by T cells. [Results] Linear regression analysis between PDL1 and PYK2 mRNA levels in melanoma showed a significant correlation (R=0.52, p<0.001). To confirm the relationship between PYK2 and PDL1, we examined whether VS6063 or TAE226 could inhibit the IFNγ-induced PDL1 expression in C8161. Indeed, these inhibitors decreased both mRNA transcription and protein expression of PDL1 in response to IFNγ. It was suggested that PYK2 is involved in the transcription of PDL1 mRNA, we searched for upstream transcriptional factors. Interferon regulatory factor 1 (IRF1) and signal transducer and activator of transcription 1 (STAT1) have been reported as transcription factors promoted by IFNγ for PDL1 transcription. Analysis of TCGA data further revealed a positive correlation between IRF1 and PYK2 (R=0.69, *p*<0.001). Similarly, a positive correlation was observed between STAT1 and PYK 2 (R=0.47, *p*<0.001). Moreover, PYK2 inhibitor significantly negated the mRNA transcription and the protein expression of the IFNγ-induced STAT1 and IRF1 (n=4, *p*<0.001). These results indicate PYK2's role in regulating PDL1 expression through STAT1 and IRF1.Our results demonstrated that PYK2 inhibitors decreased the mRNA transcription and protein expression of PD-L1, thereby bolstering anti-tumor immunity. Subsequently, we assessed whether downregulation of PD-L1 expression in cancer cells amplified anti-tumor immunity using a Killing assay. T cells and cancer cell lines were co-cultured, and the efficacy of adding PYK2 inhibitors for enhancing anti-tumor effects was assessed. As expected, The Killing assay demonstrated enhanced anti-tumor effects when combining immune cells with PYK2 inhibitors. [Conclusion]PYK2 inhibitors can enhance tumor immunity and may work synergistically with immune checkpoint inhibitors.

### [2O07-02-04]

### Expression of Inflammatory Cytokines through the Activation of the Mechanoreceptor Piezo1 in Conjunctival Epithelial Cells – What Happens When We Rub Our Eyes

*Naoki Adachi[1], Seiya Fukuoka[1], Yasunori Takayama[1], Erika Ouchi[1], Hideshi Ikemoto[1], Takayuki Okumo[1], Masataka Sunagawa[1] ([1]*Department of Physiology, Showa University Graduate School of Medicine*)

Piezo1 is a mechanically activated ion channel with Ca2+ permeability, playing a vital role in sensing mechanical forces in various cells.In this presentation, we introduce the increased expression of inflammatory cytokines through the activation of Piezo1 in human conjunctival epithelial cells. In cultured human conjunctival epithelial cells, the expression of Piezo1 was confirmed, and an increase in the expression of inflammatory cytokines such as IL-6 and IL-8 was observed when treated with Yoda1, a selective Piezo1 agonist.In conjunctival epithelial cells, the activation of p38 MAPK and a transcription factor CREB were observed upon exposure to Yoda1. The activation of both p38 MAPK and CREB, as well as the production of IL-6, were inhibited by the p38 MAPK inhibitor SB203580, indicating that the activation of CREB and the production of IL-6 were promoted downstream of p38 MAPK activation after Yoda1 treatment.Furthermore, in the absence of extracellular Ca2+ and in the presence of the intracellular calcium chelator BAPTA-AM, no increase in the activation of p38 MAPK, CREB, or the expression of IL-6 was observed after Yoda1 exposure. This suggests that the activation of these intracellular signals and IL-6 expression requires Ca2+ influx through the Piezo1 channel.Experiments using a cell stretching device confirmed the activation of p38 MAPK and CREB and an increase in IL-6 expression with stretching stimuli in cultured conjunctival epithelial cells. Additionally, confirmation experiments in vivo are currently underway.These results indicate that mechanical stimulation leads to increased expression of inflammatory cytokines in conjunctival epithelial cells through the activation of the Piezo1 channel and intracellular signaling. These results suggest one of the things that can happen when we rub our eyes.

### [2O07-02-05]

### Different impacts of direct thrombin inhibitor and Factor Xa inhibitor on activated platelet-initiated plasma clot formation and lysis

*Yuko Suzuki[1], Hideto Sano[2,1], Nanami Morooka[1], Naoki Honkura[1], Tetsumei Urano[3,1] ([1]*Medical Physiology, Hamamatsu University School of Medicine,* [2]*Physiology, Tokai University School of Medicine,* [3]*Shizulka Graduate University of Public Health*)

Background: Fibrinolysis is spatiotemporally well-regulated and greatly influenced by activated platelets and coagulation activity. Our previous real-time imaging analyses revealed that clotting begins on activated platelet surfaces, leading to uneven-density fibrin structures, and fibrinolysis also begins from dense fibrin regions and propagates to the periphery. Although direct oral anticoagulants (DOACs) are widely used in clinical practice, their effects on thrombin-dependent activation of thrombin-activatable fibrinolysis inhibitor (TAFI) and fibrinolysis are still unknown.Objectives: This study investigated the effect of different DOACs on TAFI-mediated fibrinolysis inhibition.Methods: Using human platelet-containing plasma, we employed turbidimetric assay to measure clotting time and lysis time and thrombin generation assay in the presence of rivaroxaban (activated factor X inhibitor) or dabigatran (direct thrombin inhibitor). We also visualized clot formation and lysis by confocal laser scanning microscopy and evaluated the effects of rivaroxaban and dabigatran.Results: Rivaroxaban and dabigatran reduced thrombin generation, prolonged clotting time, and shortened lysis time comparably in a dose-dependent manner. The highest concentration of DOACs showed no further shortening of lysis time with activated TAFI inhibitor. Fibrin network structures initiated by activated platelets and fluorescent-labeled plasminogen localization were unique for these two drugs. Rivaroxaban maintained an uneven fibrin network but promoted faster plasminogen accumulation and fibrinolysis from outside dense fibrin regions. In contrast, dabigatran showed a more even fibrin network, and the fibrinolysis started from the activated platelets and propagated to the periphery.Conclusions: Visualizing and analyzing the patterns of fibrin network formation, plasminogen accumulation, and fibrinolysis offer new insights into the anticoagulants' specific impacts on coagulation and fibrinolysis.

### Oral

[2O07-03]
**Circulation**

March 29, 14:20 - 15:20, Room 7

### [2O07-03-01]

### Spatial organizations of heterochromatin underpin cardiomyocyte nuclear structural integrity against mechanical stress

*Maretoshi Hirai[1], Keita Fujiwara[1] ([1]*Kansai Medical Univ.*)

Cardiomyocyte nuclei are constantly exposed to mechanical stress, and how they maintain nuclear shape remains a mystery. In this study, we found that mouse cardiomyocyte nuclei have characteristic spatial organizations of heterochromatin (SOH), and that high-level expression of H2B-mCherry in cardiomyocytes causes the disruption of SOH and entire higher order genomic structures (HOGS), leading to extreme elongation and rupture of nuclei. Loose chromatin then leaks into the cytosol and activates the cGAS/STING pathway, causing severe inflammation and lethal cardiac dysfunction. Despite HOGS disruption, the change in gene expression was surprisingly mild, suggesting their primary role in nuclear structural integrity. Furthermore, by leveraging this heterochromatin disruption system, we provide mechanistic insights into the establishment and maintenance of SOH, which is driven by chromatin compaction by histone H1 and condensate formation by methyl-CpG-binding protein 2 (MeCP2). Thus, we have highlighted the essential role of SOH as a safeguard of nuclear shape and genomic integrity against mechanical stress.

### [2O07-03-02]

### S4C: Characterizing Chaotic Properties in Cardiomyocytes with Contraction Rhythm Homeostasis

*Seine A. Shintani[1,2,3] ([1]*Department of Biomedical Sciences, College of Life and Health Sciences, Chubu University,* [2]*Center for Mathematical Science and Artificial Intelligence, Chubu University,* [3]*Institute for Advanced Research, Nagoya University*)

In a groundbreaking study using rat-derived cultured cardiomyocytes, we unearthed an intriguing behavior not previously documented. When these cells were subjected to temperatures ranging between 37-43°C, the sarcomeres—fundamental contractile units within these cells—began to oscillate in a continuous pattern of contraction and relaxation. For the sake of clarity and communication within the scientific community, we christened this phenomenon "HSOs (Hyperthermal Sarcomeric Oscillations)."

One of the standout features of HSOs is their adaptability. As we manipulated intracellular calcium concentrations, the oscillations' waveform underwent modifications. However, despite these changes in form, the HSOs consistently exhibited a stable rhythmicity. Another fascinating observation was the seemingly chaotic behavior of the oscillations. Adjacent sarcomeres demonstrated a propensity to synchronize, either in tandem or in opposing phases, creating a mosaic of patterns.

To better understand the underpinnings of this behavior, we compared the HSOs to sarcomeric oscillations observed under constant calcium concentrations. This comparison revealed that the unpredictable, chaotic tendencies of the oscillations are pivotal in sustaining the consistent periodicity—a balance of order and chaos. Recognizing the importance and uniqueness of this discovery, we introduced a new term: "S4C (Sarcomere Chaos with Changes in Calcium Concentration)."

What's even more captivating is the broader physiological relevance of S4C. Our subsequent investigations have indicated that this S4C phenomenon plays a role in the intrinsic beating patterns of cardiomyocytes. In this presentation, our objective is not just to share these findings but to foster a rich discussion, exploring the ramifications of S4C in cardiac health and its potential implications in medical science.

### [2O07-03-03]

### Roles of pericytes in the regulation of wound angiogenesis revealed by fluorescence-based live-imaging in adult zebrafish

*Tomohiro Ishii[1], Shinya Yuge[1], Koji Ando[1], Shigetomo Fukuhara[1] ([1]*Dept. of Mol. Pathophysiol., Inst. of Adv. Med. Sci., Nippon Medical School*)

**[OBJECTIVES]** Pericytes are mural cells that wrap around the walls of small caliber vessels to regulate vascular integrity, blood flow, and vascular permeability. When tissues are injured, angiogenesis is induced to generate neovessels for tissue repair. Angiogenesis is a morphogenetic process in which new blood vessels form from pre-existing ones, involving endothelial cell (EC) sprouting, migration, and proliferation. It has long been thought that upon induction of angiogenesis, pericytes detach from the vessel wall to promote EC sprouting. However, we recently live-imaged cutaneous wound angiogenesis in adult zebrafish and showed that during this process, pericytes actively proliferate to cover the activated ECs without detaching from the vessel wall. Here, we investigated the role of pericytes in regulating wound angiogenesis.
**[METHODS]** Adult zebrafish that label endothelial cell and pericyte with fluorescent proteins were anesthetized with 2-phenoxy ethanol and skin vasculature was observed under confocal microscopy. For conditional pericyte ablation, we used the Nitroreductase/Metronidazole system. Using these methods, we analyzed cutaneous wound angiogenesis in the presence and absence of pericytes.
**[RESULTS]** Skin injury induced wound angiogenesis in both control and pericyte-ablated adult zebrafish. However, dense and disorganized blood vessels formed in the lesions of pericyte-ablated fish at 7 days post-injury compared to control fish. Consistently, pericyte-ablated fish exhibited increased vessel length compared with control fish. Therefore, we investigated the cause of this and showed that the number of EC sprouting events and the number of proliferating ECs in the capillaries increased in the pericyte-ablated fish compared to control fish. Furthermore, in the absence of pericytes, the injured vessels elongated while changing direction, thereby forming abnormal vasculature. In contrast, pericyte-covered injured vessels directionally elongated and formed repaired vessels with similar structures to the pre-injured ones.
**[CONCLUSIONS]** These results indicate that pericytes play an active role in generating functional blood vessels during wound angiogenesis by suppressing excessive sprouting and proliferation of ECs and facilitating directional vessel elongation.

### [2O07-03-04]

### Myocardial acute stretch-induced cellular ROS production is enhanced in high fat diet fed mice

*Yumiko Chiba[1], Gentaro Iribe[1] ([1]*Department of Physiology, Asahikawa Medical University*)

It is well known that diabetic patients have a higher incidence of heart failure than non-diabetic patients. Oxidative stress in the diabetic heart has been suggested as one of the main mechanisms for the development of heart failure. We recently reported that myocardial immediate reactive oxygen species (ROS) production by transient myocardial stretch is required to maintain contractility under stretched conditions. Here, we hypothesized that this stretch-induced physiological ROS production may be enhanced by hyperglycemia, so that it may finally be transformed to pathologically excessive oxidative stress as a possible reason for the higher incidence of heart failure in diabetic patients. To test this hypothesis, we investigated the effects of hyperglycemia on myocardial stretch-induced ROS production in the present study. Ventricular myocytes were enzymatically isolated from mice fed either high fat diet (HFD mice) or normal chow diet (NCD mice) as a control. Also, we isolated myocytes from streptozocin which pancreatic beta cell toxin induced hyperglycemia mice (STZ mice). Axial stretch of 8-10 % in sarcomere length were applied to the cells using a pair of carbon fibers attached to both cell ends. Stretch-induced change in cellular ROS production was estimated using 2’-7’ dichlorofluorescein. Although, both HFD and STZ mice showed hyperglycemia, the stretch-induced increase in ROS production was significantly higher in HFD mice than in NCD mice and was not different in STZ mice compared to NCD mice. The present results indicate that myocardial stretch-induced ROS can be enhanced in HFD-induced diabetics, however, hyperglycemia itself may not be the essential cause. (COI: NO)

### [2O07-03-05]

### Contribution of KATP channels activated in ventricular myocytes to repolarization: a simulation study

*Yukiko Himeno[1], Hiroto Nomura[1], Wenli Zhang[1], Yixin Zhang[1], Yuttamol Muangkram[1], Ayako Takeuchi[2], Akinori Noma[1], Akira Amano[1] ([1]*Ritsumeikan Univ.*, [2]*Fukui Univ.*)

ATP-sensitive $K^+$ (KATP) channel was initially found in cardiomyocytes (Noma *et al.* 1983). It is known that KATP channel current ($I_{KATP}$) is activated by a decrease in intracellular ATP concentration ($[ATP]_i$), an increase in ADP concentration ($[ADP]_i$), and/or a corresponding change in the ADP/ATP ratio. It has been suggested to have a cardioprotective effect. However, the activation ranges of $[ATP]_i$ reported in previous studies are very low, and the extent of its influence on the heart in physiological conditions is not well known. In this simulation study, we investigated the contribution of KATP channels activated in ventricular myocytes to repolarize those cells which were continuously depolarized. When the human ventricular cell (hVC) model was subjected to continuous early afterdepolarization (EAD) conditions by an additional sustained inward current component of the late sodium current ($I_{NaL}$) and the oxygen partial pressure was lowered, ATP consumption by contraction increased and $[ATP]_i$ decreased. As $[ATP]_i$ decreased, $I_{KATP}$ was activated, and EAD was stopped. When EADs were similarly generated continuously in a one-dimensional array hVC model in which repolarization propagates (Himeno *et al*., 2023), $I_{KATP}$ activation triggered the repolarization of the entire array. These results obtained using a comprehensive cardiomyocyte model revealed the interaction of these factors related to the protective effect of $I_{KATP}$, such as ATP consumption by the membrane and ATP consumption by contraction, the action of creatine kinase and adenylate kinase as the $[ATP]_i$ interfering systems, the ATP synthesis reaction by mitochondria, the matrix membrane potential, such as changes in membrane excitability and membrane ion transporters, changes in $[Na^+]$ and $[Ca^{2+}]$ concentrations, $Ca^{2+}$-induced $Ca^{2+}$ release by E-C coupling, myosin ATPase activity in the contractile system, etc.. Therefore, we concluded that $I_{KATP}$ indeed played a cardioprotective role in the ventricle and that it was possible to predict complex pathophysiological mechanisms of the arrhythmic state of the ventricle comprehensively in the present simulation study.

### Oral

[2O07-04]
**Circulation, Renal function**

March 29, 15:20 - 16:20, Room 7

### [2O07-04-01]

### Myofilament changes may drive right ventricular dysfunction ahead of pulmonary hypertension in a rat model of left ventricular diastolic dysfunction.

*Mark T. Waddingham[1], Hirotsugu Tsuchimochi[1], Takeshi Ogo[1], James Pearson[1]
([1]*National Cerebral and Cardiovascular Center*)

**Background:** Patients with left ventricle diastolic dysfunction (LVDD) often develop secondary pulmonary hypertension (PH) and subsequent right ventricle (RV) dysfunction. The pathological mechanisms underlying the RV dysfunction associated with LVDD-related PH is still unknown, however most likely involves posttranslational modifications of key myofilament proteins within the cardiac sarcomere that regulate myocardial contraction and relaxation.
**Objective:** To assess RV function and myofilament protein post-translational modifications in the Dahl salt-sensitive rat model of LVDD.
**Methods:** Male Dahl Iwai salt-sensitive (DIS, n=8) and Dahl Iwai salt-resistant (DIR, n=8) rats were provided a 4% NaCl diet for 12 weeks. LV and RV function was measured by echocardiography and pressure-volumetry under isoflurane anesthesia, respectively. Myofilaments were extracted from RV tissue and the phosphorylation status established by immunoblotting.
**Results:** Compared to the DIR rats, DIS rats exhibited significant LV hypertrophy and increased wall thickness, consistent with the development of LVDD. In the RV, DIS rats exhibited a mildly increased systolic pressure compared to DIR rats (28.3±1.4 vs. 25±1.2mmHg, P=0.07), but without overt PH. DIS rats exhibited RV dysfunction as evident by a significantly increased tau relaxation time constant and significantly reduced contractility index (both P<0.05 vs. DIR). Site-specific phosphorylation analysis of myofilament proteins cMyBP-C, cTnI and MLC-2v in RV myocardial tissue revealed significantly lower cMyBP-C (P<0.05 vs. DIR) and cTnI (P<0.001 vs. DIR) phosphorylation and elevated MLC-2v phosphorylation in DIS rats. Importantly, changes in RV myofilament protein phosphorylation status correlated with early changes in RV function.
**Conclusion:** Early changes in myofilament protein phosphorylation likely drive early RV dysfunction ahead of overt LVDD-related PH.
**COI:** NO

### [2O07-04-02]

### Sphingosine kinase 1 is integral for elastin deficiency-induced arterial muscularization

*Junichi Saito[1], Jui Dave[1], Inamul Kabir[1], Eunate Gallardo[1], Timothy Hla[2], Daniel Greif[1] ([1]*Yale University School of Medicine,* [2]*Harvard Medical School*)

**Introduction:** Defective elastin and smooth muscle cell (SMC) accumulation are characteristics of both arterial diseases and ductus arteriosus (DA) closure. Elastin deficiency induces SMC hyperproliferation; however, mechanisms underlying this effect are not well elucidated. As elastin (ELN) is expressed from embryonic day (E) 14 in the mouse aorta, our study started with an analysis of morphological and molecular differences between wildtype (WT) and *Eln(-/-)* aortas at E15.5. Through an unbiased screen of elastin-deficient arteries, we identified sphingosine kinase 1 (SPHK1) as a candidate molecule and assessed its function in this context.
**Methods:** Immunostaining for SMC and proliferation markers was performed on WT and *Eln(-/-)* mouse aortas at E13.5, E15.5, and postnatal day (P) 0.5. Bulk RNA-seq was conducted on mouse aortic SMCs isolated from WT or *Eln(-/-)* embryos at E15.5. SPHK1 expression was evaluated in human aortic SMCs in culture, in aortic samples derived from *ELN*-deficient patients, and in mouse WT and *Eln(-/-)* aortas and WT DA. SPHK1 inhibitor was administered daily to pregnant dams from E13.5 to E19.5, and pups were analyzed at P0.5.
**Results:** SMC hyperproliferation is first observed in *Eln(-/-)* aorta at E15.5, prior to morphological differences. Bulk RNA-seq reveals that Sphk1 is the most upregulated transcript in *Eln(-/-)* aortic SMCs at E15.5. Expression of reported upregulated genes (e.g., Notch pathway molecules) is not yet increased at this early stage. Reduced ELN increases SPHK1 levels in human aortic samples and mouse aorta. SPHK1 is also upregulated in WT DA compared to the adjacent descending aorta. SPHK1 inhibition attenuates SMC proliferation and muscularization in elastin-defective arteries, leading to extended viability of *Eln(-/-)* mice and patent DA in WT mice.
**Conclusions:** Elastin deficiency-induced SPHK1 stimulates SMC hyperproliferation. As sphingolipids modulate several pathways including Notch, we put forth SPHK1-mediated signaling as a key node in the initial stages of elastin deficiency-induced hypermuscularization. Combining these findings with our upcoming experiments of SMC-specific *Sphk1* deletion and sphingolipid receptor activity will provide steps toward novel therapeutics for pediatric arterial diseases.

### [2O07-04-03]

### Mechanisms of hypertension induction via autonomic-immune system

*Ryusuke Umene[1,2], Peter Joseph Kasyoki[2], Norito Washimine[1], Chia-Hsien Wu[2], Yasuna Nakamura[2], Tomoya Nishino[1], Tsuyoshi Inoue[2] ([1]*Department of Nephrology, Graduate School of Biomedical Sciences, Nagasaki University,* [2]*Department of Physiology of Visceral Function and Body Fluid, Graduate School of Biomedical Sciences, Nagasaki University*)

Background: Previous research has indicated the involvement of immune cells in the induction of hypertension, with various immune cells affecting the heart, kidneys, and blood vessels. In kidney, there is evidence suggesting a connection between sympathetic nerve activation and the development of renal fibrosis, as well as the infiltration of macrophages, which also contributes to renal fibrosis. Additionally, there is evidence supporting the idea that renal sympathetic nerve activation is linked to the onset of hypertension. However, a comprehensive explanation for these mechanisms has not yet been established. On the other hand, anti-inflammatory and organ-protective mechanisms mediated through the neuroimmune system have been revealed, especially the spotlight is on the renal protection mechanism through autonomic nerve stimulation via macrophages. Therefore, our aim is to understand the blood pressure control mechanism through the autonomic nervous system and the immune system.
Methods: We induced hypertension in mice by delivering angiotensin II (ATII) via an osmotic pump and high-salt intake. To evaluate the influence of autonomic agonists on the induction of hypertension through immunocytes under different conditions, we conducted adoptive transfers of splenocytes from wild-type donor mice to hypertensive recipient mice. Additionally, we examined whether the administration of ATII to wild-type mice with depleted white blood cell (WBC) lineage or mice with knockout of macrophage-specific autonomic receptors would induce hypertension.
Results: We successfully induced hypertension through the administration of ATII. The adoptive transfer of autonomic nerve-stimulated splenocytes led to hypertension in recipient mice who had received splenocytes treated with either nicotine or saline. However, the induction of hypertension was suppressed in mice that received noradrenaline-treated splenocytes. In wild-type mice with depleted macrophages, ATII-induced hypertension was effectively inhibited. Furthermore, mice with knockout of macrophage-specific autonomic receptors also exhibited reduced hypertension induction.
Conclusion: These results emphasize the role of splenocytes in the induction of hypertension, with sympathetically stimulated splenocytes demonstrating the ability to inhibit hypertension induction, likely through their effects on autonomic receptors present on macrophages.

### [2O07-04-04]

### Modeling cellular urate transport to understand renal transport of uric acid at the single-cell resolution in humans.

*Yoshihiko Sakaguchi[1,2,3,4,5], Pattama Wiriyasermkul[2,3], Masaki Miyasaka[2,3], Shushi Nagamori[2,3] ([1]*Department of Future Basic Medicine, Nara Medical University,* [2]*Center for SI Medical Research, The Jikei University School of Medicine,* [3]*Department of Laboratory Medicine, The Jikei University School of Medicine,* [4]*Tsukuba university The International Institute for Integrative Sleep Medicine,* [5]*PhD Program in Humanics, School of Integrative and Global Majors, University of Tsukuba*)

(Purpose) The kidney reabsorbs and secretes a variety of substances via transcellular transport across at least two membranes, the apical and basolateral membranes. A number of transporters have been reported as proteins that transport uric acid, an end product of purine metabolism in humans. However, it remains unclear how the transporters cooperate and regulate the reabsorption and secretion. Here, we present the expression patterns of the eight commonly known urate transporters at a single-cell resolution and propose a renal excretion model for cellular urate transport.
(Methods) We analyzed cellular transport directions of urate using publicly available single-cell RNA-sequencing data from three human renal biopsies. By classifying the tubular cells based on the expression pattern of urate transporters, we created an excretion model of urate transport in human renal tubular cells.
(Results and Discussion) We demonstrated two crucial characteristics of urate transport: Firstly, there is a specific cell population that is responsible only for reabsorption and secretion. Secondly, while the known transport direction of the urate transporters is appropriate for the entire tubule, some transporters can reverse the transport direction under physiological conditions, depending on the other transporters they co-express. This promising method extends the direction of transport from the molecular to the cellular level and is applicable to polarized epithelial cells other than renal cells, other substrates, and other species.

### [2O07-04-05]

### Physiological importance and association with podocyte function of the tRNA modification enzyme, *Cdkal1*

*Hiroko Nagata[1], Yuu Nagayoshi[1], Takeshi Chujyo[1], Hitoshi Nakazato[2], Kazuhito Tomizawa[1] ([1]*Department of Molecular Physiology, Kumamoto University,* [2]*Department of Pediatrics, Kumamoto University*)

Genome-wide association studies have reported that single nucleotide polymorphisms of the Cdk5 regulatory subunit associated protein1-like1(*CDKAL1*) gene are responsible for type 2 diabetes. We found that *CDKAL1* is an enzyme that thiomethylates $tRNA^{Lys (UUU)}$ at position 37 and that loss-of-function mutations of *CDKAL1* cause reduction of mature insulin secretion. In addition, *CDKAL1* mutation was also reported as a risk factor for chronic kidney disease (CKD) progression in patients with type 2 diabetes. Therefore, we make the hypothesis that tRNA thiomethylation related to physiological function in the kidney and CKD progression. First, we performed immunostaining of mouse kidney and *Cdkal1* was expressed at glomeruli. We also performed co-immunostaining with nephrin, and *Cdkal1* expression was co-localized with nephrin. From these results, *Cdkal1* might have physiological roles in podocytes. Next, we generated systemic *Cdkal1* knock out (KO) mice (*Cdkal1* CAG-Cre). *Cdkal1* CAG-Cre mice showed phenotype of albuminuria. We also performed electron microscopic images of the glomeruli and we found the podocyte foot process effacement in *Cdkal1* KO mouse. To examine the association between *Cdkal1* and podocyte function, we generated *Cdkal1* KO cells using CRISPR-Cas9 system in mice podocyte cell line (E11). We performed wound healing cell migration assay to examine the migratory ability of *Cdkal1* KO cells. As a result, we observed that cell migration ability was significantly reduced in *Cdkal1* KO cells. Next, proteome analysis revealed that lysine translation efficiency was decreased in *Cdkal1* KO cells. Gene Ontology analysis showed expression of proteins related with glomerular basement membrane or podocyte migration function significantly affected. Finally, we focused on the Lysine-rich proteins related with podocyte function. We found that Cd2 associated protein (Cd2ap) is one of Lysine-rich proteins and worked for a slit membrane in podocyte. The expression of Cd2ap is significantly decreased in *Cdkal1* KO podocytes. These results suggest that *CDKAL1* deficiency leads to decreased lysine translation efficiency in podocytes. Especially, the proteins related with podocyte function, like Cd2ap were decreased and this reduction causes the phenotypes of foot process effacement, migration abnormality and albuminuria.

### Oral

### [3O07-01]
### Neural network, Plasticity

March 30, 8:50 - 9:50, Room 7

### [3O07-01-01]

### Sustained fluorescent labeling of LTP spines in hippocampal neurons

*Yusuke Sugimoto[1], Shin-ya Kawaguchi[1] ([1]*Graduate School of Science, Kyoto University*)

Long-term potentiation (LTP) in the hippocampus is one type of synaptic plasticity underlying learning and memory in animals. Many studies have focused on the properties and mechanism of hippocampal LTP induction at synapses and its impact on learning. Among them, some previous reports suggested a relationship between the LTP induction and a positive feedback loop consisting of protein kinase C (PKC), mitogen activated protein kinase (MAPK), and so on. However, it remains largely unknown whether the signal cascade contributes to the LTP establishment and how the signaling pathway is activated and maintained in a hippocampal neuron. To examine these issues, we used a fluorescent probe composed of a mutated PKCα and Venus to detect and quantitively analyzed the activation of dynamics and functional role of the positive feedback loop in cultured hippocampal neurons. We transfected the mut-PKCα-Venus probe and induced chemical LTP (cLTP) in pyramidal neurons. Patch-clamp recording of mEPSCs indicated the LTP establishment by the cLTP stimulation. cLTP stimulation caused sustained accumulation of the probe into spines for 1 hour, while the probe was ubiquitously distributed before the stimulation. On the other hand, inhibition of MEK or CaMKII, which contributes to the PKC-MAPK signaling pathway, by U0126 or KN-62, respectively, abolished prolonged accumulation of the mut-PKCα-Venus. Accordingly, mEPSCs enhancement by cLTP stimulation was suppressed by the MEK or CaMKIIinhibition. Summarizing these results, the probe alters its own distribution toward synapses where LTP is established. Furthermore, we selectively and locally induced LTP at a specific spine by uncaging of MNI-Glu with 405nm spot laser illumination and studied the translocation of the fluorescent probe together with electrophysiological recordings. We demonstrate the relationship between the spatial patterns of the probe accumulation and the LTP establishment.

### [3O07-01-02]

### Development of hippocampal learning function and CA1 synaptic plasticity: pathway-specific excitatory postsynaptic currents correlated with individual learning performance

*Yuheng Yang[1], Yuya Sakimoto[1], Dai Mitsushima[1] ([1]*Department of Physiology, Yamaguchi University Graduate School of Medicine.*)

We previously showed that contextual learning requires synaptic delivery of AMPA receptors into the CA1 synapses in 4- to 5-week-old rats (Mitsushima et al., *PNAS* 2011, *Nat Commun* 2013). Our behavioral test battery showed that there is a critical period of contextual learning between postnatal days (PN) 16 and 22, independent of developmental changes in emotional, sensory, and motor functions (Sakimoto et al., *Sci Rep* 2022). To further identify the critical days of learning and associated plasticity, we subjected juvenile male rats to an inhibitory avoidance task and prepared hippocampal slices for patch clamp analysis. By stimulating ECIII-CA1 or CA3-CA1 input fibers, pathway-specific evoked excitatory postsynaptic currents were obtained in the same animals. We found a critical period of contextual learning between PN 16 and 17, but the training promoted postsynaptic plasticity before PN 16. Compared to untrained controls, IA training increased the AMPA/NMDA ratio of CA3-CA1 synapses from PN 16 to PN 23, and the AMPA/NMDA ratio of ECIII-CA1 synapses from PN 16 to PN 21. More importantly, in both synapses, contextual learning performance was positively correlated with the mean AMPA/NMDA ratio after PN 23, indicating a day of functional linkage between learning and plasticity. In the future, we hope to quantify excitatory synaptic AMPA receptors and elucidate in detail the developmental changes in training-dependent excitatory synaptic strengthening.

### [3O07-01-03]

### Local sustained calcium elevation at a dendritic branch triggered by high-frequency glutamate input

*Riku Okawa[1], Shin-ya Kawaguchi[1] ([1]*Graduate School of Science, Kyoto University*)

Cytoplasmic $Ca^{2+}$ rise is an important molecular signal for the synaptic plasticity induction. In cerebellar Purkinje cells, for example, elevation of intracellular $Ca^{2+}$ concentration ($[Ca^{2+}]_i$) induces long-term depression (LTD) or long-term potentiation (LTP) depending on the intensity of $[Ca^{2+}]_i$ rises.To monitor the $[Ca^{2+}]_i$ rise during synaptic plasticity induction in Purkinje cells, we expressed a $Ca^{2+}$ probe protein GCaMP7f and uncaged MNI-glutamate on a dendritic spine by high-frequency 405 nm laser illumination. 80Hz uncaging of MNI-glutamate caused strong $[Ca^{2+}]_i$ increase which is sufficient for the LTD induction there. Surprisingly, several tens of seconds after a large transient increase, $[Ca^{2+}]_i$ again started to rise locally, which persisted for longer than 30 minutes. This $[Ca^{2+}]_i$ elevation was confined to the stimulated point several μm in diameter. As a possible candidate mechanism for the local sustained $[Ca^{2+}]_i$ elevation, we tested the involvement of calcium-induced calcium release (CICR) or store-operated calcium entry (SOCE) which depends on the ER $Ca^{2+}$ store. However, even after the depletion of internal $Ca^{2+}$ store by thapsigargin, the local sustained $[Ca^{2+}]_i$ rise occurred. On the other hand, extracellular EGTA application rapidly cancelled the once-established sustained $[Ca^{2+}]_i$ rise, suggesting that it depends on continuous active mechanism to locally drive $Ca^{2+}$ at the stimulated site. Taken together, our study unveils a novel phenomenon of $Ca^{2+}$ dynamics which switches the $[Ca^{2+}]_i$ rise from transient to long signal. In this poster, we are going to demonstrate the spatiotemporal dynamics and molecule mechanism of this unique $Ca^{2+}$ signaling.

### [3O07-01-04]

### LINC complex-mediated regulation of axon initial segment

*Koichi Hasegawa[1], Noriyuki Hama[1], Mina Amemiya[1], Ken-ichiro Kuwako[1]
([1]*Department of Neural and Muscular Physiology, School of Medicine, Shimane University*)

The axon initial segment (AIS) is located at the proximal site of axon and plays a pivotal role in generation of action potentials through specific voltage-gated ion channels. The AIS has structural plasticity that alters its length and position in response to input stimuli, thereby fluctuating the composition of AIS molecules, such as voltage-gated $Na^+$/$K^+$ channels, and consequently regulating neuronal excitability. The nuclear membrane LINC complex, composed of Sun1/2 and Nesprin1/2, plays an important role in connecting the nuclear envelope to the cytoskeleton. Previous studies have shown that the LINC complex is involved in diverse cellular events, such as cell polarity and migration via cytoskeletal regulation. In this study, we focused on the regulation of AIS by the LINC complex. Expression of the Nesprin dominant-negative mutant (Nesprin-DN) that inhibits the LINC function significantly shortened AIS length in a variety of neurons *in vitro* and *in vivo*. Moreover, cortical neurons expressing Nesprin-DN completely abolished the structural plasticity of the AIS induced by chronic depolarization. To assess the importance of the LINC complex in neural function, we next performed patch clamp and behavioral analyses in mice in which Nesprin-DN was expressed in neurons throughout the brain by a blood-brain barrier-permeable adeno-associated virus vector. Expression of Nesprin-DN increased the current threshold of action potential and decreased the firing frequency. Furthermore, an open field test revealed the increased locomotor activity and the decreased anxiety in Nesprin-DN-expressing mice. Together, these findings suggest that the LINC complex plays an essential role in the regulation of neuronal excitability and higher brain function via AIS.

### [3O07-01-05]

### Ascertaining glutamate release sites at ribbon-type synapses in the goldfish retinal bipolar cell terminal

*Tomoko Oshima-Takago[1,2], Hirokazu Sakamoto[1], Yukihiro Nakamura[3,1], Shigeyuki Namiki[1], Kenzo Hirose[1], Masao Tachibana[4,2,1], Hideki Takago[2,1] ([1]*Department of Pharmacology, Graduate School of Medicine, The University of Tokyo,* [2]*Rehabilitation for Sensory Functions, Research Institute, National Rehabilitation Center for Persons with Disabilities,* [3]*Department of Pharmacology, Jikei University School of Medicine,* [4]*Center for Systems Vision Science, Research Organization of Science and Technology, Ritsumeikan University*)

Ribbon-type synapses in sensory organs process continuous signal flow of glutamatergic neurotransmission that is driven by slowly-inactivating $Ca^{2+}$ current. Previous electrophysiological studies demonstrated that the ribbon synapses in the goldfish retinal Mb1 ON-type bipolar cell terminal exhibits kinetically discrete types of glutamate (Glu) release upon long-lasting stimulation: the fast and slow components of evoked release. In order to identify the location of Glu release sites for both components of evoked release as well as spontaneous release, we here took advantage of Glu imaging with the enhanced hybrid-type Glu optical sensor (eEOS) targeting the retinal bipolar cell. Utilizing a combination of Glu or $Ca^{2+}$ imaging, whole-cell voltage-clamp recording, and live-labeling of synaptic ribbons, we obtained following results: first, evoked Glu and $Ca^{2+}$ hot spots upon brief stimuli occur predominantly at ribbon-associated active zones (AZs); second, the evoked Glu hot spots upon longer stimuli appears to occur at not only ribbon-associated but also ribbon-free AZs. Third, spontaneous Glu hot spots occur across the terminal. Thus, the bipolar cell ribbon synapses operate in spatiotemporally diverse manners presumably to separately convey the contrast and luminance information of light.

### Oral

### [3O07-02]
### Nutritional and metabolic physiology, Thermoregulation

March 30, 9:50 - 10:50, Room 7

### [3O07-02-01]

### Activity of medial prefrontal cortex correlate with salt preference and dietary knowledge.

*Yusuke Takatsuru[1], Sekine Yuka[1], Osera Tomoko[2] ([1]*Div. Multidimensional clinical medicine, Dept. Nutrition and health sciences, Toyo University,* [2]*Div. Applied nutrition, Dept. Nutrition and health sciences, Toyo University*)

Increasing the number of elderlies in Japan, we are in the face of a problem of appetite loss on them. Even if they have no dementia, some elderly difficult to image what they want to eat. In case of the non-recovered swallowing trouble, elderly "don't want to eat" because of they can only eat paste food. Functional near-infrared spectroscopy (fNIRS) is one of the non-invasive techniques for detecting the brain function like functional magnetic resonance imaging. If we find good methods for detecting the food preference using fNIRS, it could be useful for treat the elderly who lost the appetite. We aim to clarify the relationship between food-dependent medial prefrontal cortex (MPFC)/dorsolateral prefrontal cortex (DLPFC) activity and food preference/frequency of intake. The study was approved by the ethics committee of the Toyo University (TU2020-011-TU2021-H-099-TU2021-H-023) and all participants (11 males and 12 females) gave informed consent before the experiments. The All participants were asked food preferences/ frequency of intake using a questionnaire at the beginning of experiments. All participants ate the control dish (typical Japanese home-cooked meal: CD) or prefer dish (each participant purchase by themselves at the day of experiment: PD) on separate days. The activity of MPFC/DLPFC during eating was observed by fNIRS. Each fNIRS recording was; 20 sec for baseline, 20 sec for "just looking", and enough time for "eating" (Use first 10 min for analysis). When each participant just looking the dishes, some of them showed the positive response in MPFC/DLPFC. We found that the responder of CD disliked salty taste compared with the non-responder of CD. When each participant ate the dishes, some of them also showed the positive response in MPFC/DLPFC (however, the grouping of responders was different from when they were "just looking."). We found that CD respondents disliked salty taste compared with those in PD respondents. We also found that CD respondents know many seasonal foods compared with those in PD respondents. The activity of MPFC during PD eating was negatively correlate with the knowledge of seasonal foods. We concluded that activity of MPFC/DLPFC during eating something dishes is different depend on preference/ habits of food and technique of fNIRS could potential to estimate them. COI:No

### [3O07-02-02]

### Role of PVH dopaminergic neurons to orchestrate the feeding

*Winda Ariyani[1,2], Haruka Tsuneoka[2], Chiharu Yoshikawa[2], Izuki Amano[3], Noriyuki Koibuchi[3], Hiroshi Ichinose[4], Tadahiro Kitamura[2], Daisuke Kohno[2] ([1]*Department of Developmental Genetics and Behavioral Neuroscience, Gunma University Graduate School of Medicine,* [2]*Metabolic Signal Research Center, Gunma University Institute for Molecular and Cellular Regulation,* [3]*Department of Integrative Physiology, Gunma University Graduate School of Medicine.,* [4]*School of Life Science and Technology, Tokyo Institute of Technology.*)

Feeding is a fragmented process, which consists of food procurement, food consumption, and meal termination. Several neuronal populations are known to be responsible for orchestrating the feeding process. However, the neural pathways that control each of these processes are not fully understood. We focused on the dopaminergic neurons in the hypothalamic paraventricular nucleus (PVH) and determined their role in feeding behavior and their neural network. Lack of *Th* in PVH reduced food intake, food consumption duration, and food-seeking behavior after overnight fasting. The GCaMP fiber photometry confirmed the activation of PVH TH neurons during the initiation of the food consumption phase. Chemogenetis-induced activation or inhibition of these neurons increased or decreased food intake. Furthermore, the combination of chemogenetics and fiber photometry showed that PVH TH neurons receive projections from NPY/AgRP and POMC neurons. In addition, projections of these neurons to lateral habenula activate the dopamine receptor during food consumption, and local microinjection of D2R antagonists significantly reduces the food intake. These results indicate that PVH dopaminergic neurons are key feeding neurons that control food consumption downstream of the homeostatic feeding pathway, which may play a role in further craving food for meal initiation.

### [3O07-02-03]

### Increased expression of MAFbx (muscle atrophy F-box protein) under fasting conditions is required to prevent apoptosis in skeletal muscle undergoing atrophy

*Yoshihiro Egashira[1], Shuntaro Nakamura[1], Shinri Iwasaki[2], Fumihito Ono[1] ([1]*Osaka Medical and Pharmaceutical University,* [2]*Tama Aoba Hospital*)

Skeletal muscle undergoes atrophy in response to a variety of physiological and pathological stimuli, including starvation, disuse, denervation, and glucocorticoid treatment. Despite a gross loss of cytosolic mass, muscle fibers are resistant to apoptosis during atrophy. Since atrophy depends on the ubiquitin-proteasome system and is associated with a dramatic upregulation of MAFbx (muscle atrophy F-box protein), an E3 ubiquitin ligase, MAFbx expression has been implicated as a trigger of atrophy. However, the role of MAFbx remains controversial. Zebrafish larvae are a suitable vertebrate model to address this issue because of the ease of genetic manipulation and subsequent fluorescence imaging of their homogeneous muscle fibers. Here, we used CRISPR/Cas9 genome editing to generate MAFbx reporter knock-in (KI) zebrafish in which the TagRFP coding sequence was inserted in-frame downstream of the MAFbx start codon. A cassette expressing whole body EGFP was also included in the donor fragment, allowing us to simultaneously visualize muscle morphology and its MAFbx expression. We have shown that in heterozygous KI fish, reporter fluorescence appears only under fasting conditions, when muscle atrophy is in progress. The fact that the increase in MAFbx expression was associated with the progression of atrophy was consistent with previous studies in mouse models. We next examined homozygous KI fish lacking MAFbx. Although no defects were observed when the fish were fed, muscle fibers were severely degenerated in fasted homozygous KI fish. Consistently, locomotion as assessed by escape swimming was impaired in fasted homozygous zebrafish. These results suggest that MAFbx deficiency leads to muscle degeneration only when the fish are in a catabolic state. Finally, we have shown that this muscle degeneration is mediated by an apoptotic event using gene expression of the secretable form of annexin V, a marker of apoptosis. Our study revealed that MAFbx expression stimulated in catabolic state is required to prevent apoptotic cell death of atrophying muscles.

### [3O07-02-04]

### FTO-regulated epitranscription in AgRP neurons enhances weight gain

*Daisuke Kohno[1], Reika Kawabata-Iwakawa[4], Shigetomo Suyama[3], Kazuto Ohashi[1], Winda Ariyani[1], Tetsushi Sadakata[2], Hiromi Yokota-Hashimoto[1], Ayumu Konno[2,4], Chiharu Yoshikawa[1], Sho Matsui[5], Toshihiko Yada[6], Hirokazu Hirai[2,4], Tsutomu Sasaki[5], Tadahiro Kitamura[1] ([1]*Institute for Molecular and Cellular Regulation, Gunma University,* [2]*Gunma University,* [3]*Keio University,* [4]*Gunma University Initiative for Advanced Research,* [5]*Graduate School of Agriculture, Kyoto University,* [6]*Gifu University*)

FTO protein is an $N^6$-methyl-adenosine ($m^6A$) demethylase encoded by *Fto* gene, which contains obesity-associated SNPs. FTO protein is related to body weight control independent of, or partially under the influence of, *Fto* SNPs. However, the mechanisms by which FTO protein, particularly in the brain, affects body weight are unknown. We developed and characterized several mouse models in which *Fto* is deleted or overexpressed in hypothalamic feeding neurons. Mice lacking or overexpressing *Fto* in the agouti-related peptide (AgRP) neurons showed decreased or increased body weight, respectively. $m^6A$-seq revealed that FTO in AgRP neurons demethylated $m^6A$ on genes associated with membrane trafficking and alternative splicing. RNA-seq followed by isoform analysis revealed that FTO potently increased exon13 inclusion of *Kif1a*. This change in splicing was predicted by AlphaFold2 to alter the hinge domain conformation, and assays revealed functional differences in KIF1A isoforms. Furthermore, FTO affected dense-core vesicle trafficking and secretion in AgRP neurons. In conclusion, FTO regulates the epitranscriptome in AgRP neurons, thereby enhancing *Kif1a* exon13 inclusion, the axonal transport of dense-core vesicles, NPY/AgRP release, and weight gain. Our results reveal unprecedent regulatory mechanisms controlling AgRP neurons and energy homeostasis.

### [3O07-02-05]

### PKM2 Deficiency Represses β-catenin to Enhance the Differentiation of Primary Brown Preadipocytes

*Nicholas Gill[1], Jenna Demeter[1], Presley Dowker-Key[1], Alexis Farmer[1], Berfu Ozmen[3], Ahmed Bettaieb[1,2,3] ([1]*Department of Nutrition-University of Tennessee - Knoxville,* [2]*Department of Biochemistry, Cellular, and Molecular Biology-University of Tennessee - Knoxville,* [3]*Graduate School of Genome Science and Technology-University of Tennessee - Knoxville*)

Obesity is a major global health concern that elevates an individual's risk of developing comorbidities, such as cardiovascular diseases and cancer. Currently, standardized therapeutic approaches have not been successful in alleviating the multifaceted burden of obesity. Therefore, the development of safe and innovative strategies is necessary to address the detrimental expansion of adipose tissue. In recent years, a focus has been placed on enhancing the differentiation and thermogenic activity of brown adipose tissue (BAT) has emerged as an appealing approach to promote energy expenditure and metabolic homeostasis. Further elucidating the complex regulatory pathways that govern BAT development and function may reveal novel targets that can be exploited to combat obesity. The glycolytic enzyme and transcriptional regulator pyruvate kinase muscle isozyme 2 (PKM2) has recently surfaced as a modulator of both adipogenesis and thermogenesis. PKM2 is upregulated under conditions such as cold exposure and hypoxia that promote BAT differentiation and thermogenic activity while also being allosterically regulated by various metabolic substrates. These observations lend credibility to the notion that PKM2 may play an important role in BAT physiology, however, PKM2's role in brown adipogenesis and the underlying mechanisms have yet to be investigated. Therefore, with this research, we strived to elucidate the extent of regulatory control PKM2 exerts during the differentiation of brown adipocytes and the key mediators involved. We employed the use of genetic and pharmacological strategies to manipulate PKM2 and downstream signaling molecules pre- and post-expression, and observed the effects on primary brown preadipocyte differentiation. We monitored the differentiation process by examining lipid accumulation and the expression of brown adipocyte marker proteins such as uncoupling protein 1. Our findings validated the role of PKM2 in brown preadipocyte differentiation and identified novel downstream target genes that function as key mediators of PKM2's role in brown adipogenesis. Most notably, our research provides compelling evidence to bolster the inhibition of PKM2 as a target to treat obesity and its associated metabolic dysregulation through the upregulation and activation of BAT.
COI: No

### Oral

[3O07-03]
**Stress, Drug Action, Pharmacology, Others**

March 30, 14:20 - 15:20, Room 7

### [3O07-03-01]

### Characteristics of plasma iron metabolism in peripheral and central fatigue models in rats

*TAKURO KARAUSHI[1], TATSUYA SATO[1,2], NOBUTOSHI ICHISE[1], HIROYORI FUSAGAWA[1,3], TOSHIFUMI OGAWA[1,2], TAIKI KUDO[1], NORITSUGU TOHSE[1] ([1]*Department of Cellular Physiology and Signal Transduction, Sapporo Medical University School of Medicine*, [2]*Department of Cardiovascular, Renal, and Metabolic Medicine, Sapporo Medical University School of Medicine*, [3]*Department of Orthopaedic Surgery, Sapporo Medical University School of Medicine*)

**Backgrounds:** Fatigue is defined as a reduction in physical activity with discomfort caused by physical or mental overexertion, although the pathophysiology of fatigue has not been fully elucidated as numerous factors are postulated to play roles in the fatigue state. In the present study, we aimed to characterize plasma iron metabolism, which plays an essential role in cellular function, inflammation, and redox reactions, using rat models of physical (i.e. peripheral fatigue) and mental fatigue (i.e. central fatigue). **Methods:** Eight-week-old male and female Wistar rats were divided into three groups; peripheral fatigue, central fatigue, and sedentary control. Peripheral fatigue was induced by forced treadmill running at a speed of 15 m/min for 30 min/day, and central fatigue was induced by keeping the rats in a water-filled cage with 2.5 cm of water immersion for 5 days. A sedentary group was set up in which rats were simply allowed to enter and leave the cage for 5 days. Plasma was collected from the tail vein of the rats before and 5 days after fatigue induction to measure plasma iron, transferrin saturation (TSAT), and plasma hepcidin, which is known as a master regulator of systemic iron homeostasis. **Results:** In the female peripheral fatigue group, plasma iron was 45% lower and TSAT was 26% lower after fatigue induction than those before fatigue induction. These changes were not observed in male peripheral fatigue group and sedentary control groups. Consistently, plasma hepcidin levels were also significantly increased 2.2-fold by peripheral fatigue induction in female rats. In the central fatigue group, plasma hepcidin levels increased 1.7-fold in male rats and 2.6-fold in female rats by fatigue induction, but plasma iron levels or TSAT were not significantly altered by fatigue induction. **Conclusions:** The findings suggest that fatigue can increase plasma hepcidin levels, but characteristics of changes in plasma iron levels and TSAT are different between peripheral fatigue and central fatigue. Our results also suggest that fatigue-induced changes in plasma iron metabolism may be more evident in female rats than those in male rats.

### [3O07-03-02]

### Research of new treatment for rare genetic disorders with self-amplifying RNA.

*Takashi Iezaki[1], Motohiko Sato[1] ([1]*Aichi Medical Univ.*)

Background
Fibrodysplasia ossificans progressiva (FOP) is one of the rare genetic disorders with soft tissue progressive ossification. It is reported to be caused by ACVR1 R206H genetic mutation, but radical treatment for this disease has not been developed. In recent years, genome editing technology using CRISPR/Cas9 has been gathering attention as a technology of radically treatment for genetic disorders. However, it is still difficult to apply gene repair treatment by genome editing to humans due to off-target risk and ethical problems. In addition, the viral vector is currently the mainstream for introducing genome editing technology into tissues, but introducing a virus vector into the human body is dangerous and costly. Here, we developed gene transfer method into the human cells using self-amplifying RNA (saRNA) and lipid nanoparticle. saRNA is an mRNA which contains a target gene sequence and virus-derived RNA replicon. Since the mRNA continues to self-replicate using the virus-derived RNA replication mechanism, it can express a gene of interest for a long period of time without genome integration. This method has the potential to solve problems in conventional gene therapy and is applicable to various genetic disorders. Therefore, we examined whether the cell phenotype of *in vitro* FOP model is rescued by saRNA transfection in this research.

Methods
R206H *ACVR1* mutant construct (*ACVR1R*$^{206H}$) was retrovirally infected in myoblast cell line C2C12. Infected cells were transfected saRNA for inhibiting *ACVR1R*$^{206H}$ expression, followed by stimulation of chondrocyte differentiation medium. After 6 days incubation, chondrocyte markers and ACVR1 signaling were analyzed by ALP staining, Immunoblotting, and qPCR.

Results
The introduction of *ACVR1R*$^{206H}$ significantly increased ALP activity, Smad phosphorylation, and chondrocyte marker expression. In addition, *ACVR1R*$^{206H}$ silencing with saRNA significantly attenuated increased ALP activity, Smad phosphorylation, and chondrocyte marker expression caused by *ACVR1R*$^{206H}$ introduction.

Discussion
These results showed that transfection of saRNA inhibited *ACVR1R*$^{206H}$ function and rescued abnormal chondrocyte differentiation.

### [3O07-03-03]

### Real-Time Intraocular Antiglaucoma Drugs Measurement Using Boron-doped Diamond Microelectrodes.

*Genki Ogata[1], Mao Yoneda[1], Risa Ogata[1], Ai Hanawa[1], Kai Asai[1], Reiko Yamagishi[2], Megumi Honjo[2], Makoto Aihara[2], Yasuaki Einaga[1] ([1]*Dept of Chem, Fac of Sci and Tech, Keio Univ*, [2]*Dept of Ophthalmol, Univ of Tokyo Sch Med*)

The primary treatment for glaucoma, the leading cause of intermediate vision impairment, involves administering ocular hypotensive drugs in topical eye drops. Observation of the real-time changes in the drugs through the cornea and reaching the anterior chamber is essential to improve or develop a safe, reliable, and effective medical treatment. Conventional methods such as LC-MS/MS are used to measure the temporal changes in the drug in the aqueous humor; however, this technique involves multiple measurements of the eyes of multiple test subjects to measure changes over time with high temporal resolution. To resolve this problem, we developed a measurement method that utilizes boron-doped diamond (BDD) microelectrodes to track the real-time drug concentrations in the anterior chamber of the eye. First, we optimize the method for continuously measuring timolol maleate (TIM), a sympathetic beta-receptor antagonist, and obtain the calibration curves of each BDD microelectrode in the aqueous humor collected from porcine eyes. Next, we demonstrate the continuous *ex vivo* monitoring of the TIM in the enucleated porcine eyes. The results suggest that changes in the intracameral TIM concentration can be monitored using BDD microelectrodes.

### [3O07-03-04]

### Brain-grounding of semantic vectors improves neural decoding of visual stimuli

*Shirin Vafaei[1], Ryohei Fukuma[1], Takufumi Yanagisawa[1] ([1]*Osaka University*)

**Introduction**

Developing algorithms for accurate and comprehensive decoding of neural representation of objects is one of the fundamental goals in neuroscience. Recent studies have demonstrated the feasibility of using neuroimaging and machine learning techniques to decode the neural activity of visual stimuli (Horikawa and Kamitani 2017, Gauthier and Levy 2019). However, their prediction accuracy highly depends on the way that labels of the visual stimuli are denoted in their algorithms (Gauthier and Levy 2019). In current studies, labels are defined by word embedding vectors derived from neural network latent spaces that encode the "distributional semantics" and are based on patterns of co-occurrence of words in large text corpora (Pennington, Socher, and Manning 2014, Mikolov et al. 2013). On the other side, a semantic meaning in the brain is conveyed through various modalities such as perception, imagination, action, hearing or reading and therefore the semantic space of human brain, or brain space (Huth et al. 2012), is formed based on incorporating information from diverse sources[6]. In this study, we propose that by integrating features from the brain space into the current commonly used word embedding spaces, we can obtain a new brain-grounded, more brain-like vector representation of labels, that by using them, decoders can better learn to map the neural activity patterns to their corresponding embedding vectors compared to the cases where original word embeddings were adopted.

**References**

Gauthier, Jon, and Roger Levy. 2019. "Linking Artificial and Human Neural Representations of Language." In Proceedings of the 2019 Conference on Empirical Methods in Natural Language Processing and the 9th International Joint Conference on Natural Language Processing (EMNLP-IJCNLP), 529–39. Hong Kong, China: Association for Computational Linguistics. https://doi.org/10.18653/v1/D19-1050.

Horikawa, Tomoyasu, and Yukiyasu Kamitani. 2017. "Generic Decoding of Seen and Imagined Objects Using Hierarchical Visual Features." Nature Communications 8 (1): 15037.
https://doi.org/10.1038/ncomms15037.

Huth, Alexander G., Shinji Nishimoto, An T. Vu, and Jack L. Gallant. 2012. "A Continuous Semantic Space Describes the Representation of Thousands of Object and Action Categories across the Human Brain." Neuron 76: 1210–24.

Mikolov, Tomas, Kai Chen, Gregory S. Corrado, and Jeffrey Dean. 2013. "Efficient Estimation of Word Representations in Vector Space." In International Conference on Learning Representations.

### Oral

[3O07-04]
**Medical education, Others**

March 30, 15:20 - 16:20, Room 7

### [3O07-04-01]

**An attempt at realistic Structure and Function of the Human Body education under coronavirus: Possibilities of using VR in face-to-face lectures**

*Hidetsugu Kohzaki[1] ([1]*Faculty of Nursing, Shumei University*)

[Purpose] COVID-19 made it necessary to lecture first-year nursing students remotely on the Structure and Function of the Human Body (SFHB). As reported at last year's 100th Anniversary Meeting of the Physiological Society of Japan (1), VR and AR teaching materials were'were evaluated in depth for remote lectures, even though the teaching materials used were not necessarily aimed at nurses. This time, I report on the effectiveness of this method in face-to-face classes. [Methods] Videos, online teaching materials, and VR/AR materials contributing to the teaching of SFHB were projected onto screens during face-to-face classes, and 45 students were anonymously asked for their impressions. Responses were received from 22 students (valid response rate, 48.9%). To evaluate the usefulness of face-to-face classes, we compared rubrics, class improvement questionnaires, students' reflections after each lecture, and achievement levels based on exams with those based on distance lectures. Our university is a participant in the Society for the Administration of Remuneration for Public Transmission of School Lessons.
The study protocol by the Yamato University Faculty of Health Sciences Research Ethics Committee (YHA2015 -11). [Results] 1. Our evaluation of the responses to the use of VR and AR teaching materials revealed that 95.5% of students found them "very interesting" or "interesting," and 0% found them "not interesting." In the questionnaire, I received comments such as "Unlike with textbooks, the content was easier to understand in VR because it was viewed three-dimensionally." All items in the class improvement questionnaire received a score of 2.80 or higher out of 3.00. The test results were also on target.[Discussion] The surveyed students seemed to be interested in VR and AR, and although the teaching materials used this time were not necessarily aimed at nurses, they were well received not only remotely but also in face-to-face lectures. We inferred that the rubric created from the above achieved its usefulness in face-to-face classes at an early stage. Currently, with a co-researcher, I am creating a VR world for nurses (Nurse World). I would also like to investigate AR/VR teaching materials for nursing students (2). I want to create better teaching materials while evaluating them by using the rubric I created.
[Acknowledgment] I thank The OCD Co., Ltd. and Dr. Tetsu Terada for their helpful discussions. Also, I thank our collaborators at Nurse World. This research was partially supported by JSPS KAKENHI, Grant Number: JP21H03113.
(1). **Kohzaki, H*.** Proceedings of the 100th Anniversary Meeting of the Physiological Society of Japan. Journal of Physiological Society of Japan, 73 (Suppl 1), 11, 239, (2) **Kohzaki, H*.** Proceedings of the 1st International Conference on ICT Application Research (IAR 2023), Fukui, Japan, 2023

### [3O07-04-02]

**Beginner students' preference in assignment of quizzes with straight forward illustrations showing the answer**

*Masato Shibuya[1,2] ([1]*Dept of Physiol Kagawa Nutrition Jr Col,* [2]*Life Sci Edu Sharing Gr*)

"Step-by-step studies of Life Sciences" has been an e-learning system featuring straight forward illustrations, small step presentation, and quizzes for beginners self-learning basic life-sciences. In a health-related junior high school, during the spring vacation, students were assigned text-only quizzes without the illustrations presenting the content and the answer. The passing score was 75%. During the summer vacation, students were assigned quizzes with illustrated answers. The passing score was 100%, with the slogan "100%, 100 times". In an anonymous survey, out of 64 students, the quizzes with illustrated answers compared to the conventional quizzes was preferred in 36 and was rather preferred in 19 being helpful for memorization, and 46 and 13 respectively for motivation in understanding the illustration and content. The text only quiz was preferred in 9 students for memorization and 5 students for motivation. The study indicates that the assigning quizzes with straight forward illustrations showing the content, promotes self-studying in beginners.

### [3O07-04-03]

**A problem of individual differences of behavioral data for itch-induced scratching bouts in mice**

*Kotaro Honda[1], Mitsutoshi Tominaga[1], Kenji Takamori[1,2] ([1]*Juntendo Itch Research Center (JIRC), Institute for Environmental and Gender-Specific Medicine, Juntendo University Graduate School of Medicine,* [2]*Department of Dermatology, Juntendo University Urayasu Hospital, Chiba, Japan*)

Perception of itching in both humans and animals is commonly assessed through scratching behavior, given the knowledge that itch sensation triggers repetitive scratching bouts. The average number of scratching bouts in experimental animals serves as a well-established itch index for itch studies. However, under identical experimental conditions, individual animals often exhibit a diverse range of scratching bout numbers, and these individual differences are still unclear.This study introduces a novel methodology for itch research that deconstructs individual scratching bouts numbers into various sizes of repeat lengths within the bout sequence. Subsequently, the repeat lengths are reintegrated into a probability distribution for the experimental condition. The resulting probability distribution of repeat length was modeled as Bernoulli trials until the first success known as a geometric distribution. Individual differences in the scratching bouts numbers were explained through consistent success probability across individuals and the variability in the number of starting points in the scratching bout sequence. However, the characteristics of the success probability remain largely undisclosed. This research aimed to analyze changes in success probability, particularly the lower limit, in response to varying pruritogen dosage concentrations. Three pruritogens, representing stimulators for NP1, NP2, and NP3 peripheral neurons, were selected for the study. Beta-alanine concentrations of 50–400 mM consistently yielded a success probability of approximately 0.23. Compound 48/80, an inducer of histaminergic itch, at concentrations of 1.25–20 μg exhibited a transient stall near 0.25 between 1.25 and 2.5 μg, decreasing to approximately 0.14. Chloroquine, analyzed at concentrations of 6.25–200 μg, showed a transient stall near 0.18 between 6.25 and 25 μg, eventually decreasing to around 0.07.In conclusion, the decrease in success probability is dose-dependent but exhibits a gradual phased pattern rather than a direct proportion to dosage. The lower limit of success probability may be influenced by primary afferent pathways. Further analysis under conditions involving stimulating of multiple types of primary afferents is warranted.

### Oral

[3O09-01]
### Behavior, Biological rhythm, Sleep

March 30, 8:50 - 9:50, Room 9

### [3O09-01-01]
### Effects of conditioned taste aversion to saccharin on sweet food intake.

*Zimo Wei[1], Helai Huang[1], Tomohiko Yoshizawa[1], Inui Tadashi[1], Funahashi Makoto[1]
(*[1]Hokkaido Univ Grad Sch Dent Med Dept Oral Physiol*)

We investigated if rats avoided eating sweetened foods that contained saccharin when they had conditioned taste aversion (CTA). Usually, rats that have obtained CTA avoid ingestion of saccharin solution despite being thirsty due to water deprivation for 21 hours and 40 minutes. In other words, it is thought that the drinking behavior induced by thirst was suppressed by the memory of aversion to taste. Therefore, in this study, we hypothesized that rats resist hunger and avoid consuming sweet feed when they acquire taste aversion memory. Changes in feeding, water consumption, and body weight after CTA acquisition were measured using male SD rats (7 weeks of age) as experimental animals. The conditioned stimulus was 0.1% saccharin solution, and the unconditioned stimulus was nausea induction by injection of emetine dihydrochloride (5.54 mg/kg, 1% BW, i.p.) or cisplatin dihydrochloride (3mg/kg, 1% BW, i.p.). Rats injected with saline (1% BW, i.p.) as an unconditioned stimulus were included as a control group. Some rats were injected with ondansetron (5-HT3 receptor antagonist, 0.1 mg/kg, 0.1% BW i.p.) at 30 minutes before conditioning. The sweetened diet was prepared by mixing normal solid feed with an equal amount of 0.2% saccharin solution. Normal diet was also processed in the same way to match the texture. After a one-day recovery day after CTA acquisition, the amount of sweet diet intake was measured for five days. The emetine-treated group showed a significant decrease in sweet diet intake in the test day of 1-4 as compared to the conditioning day ($F(5,30) = 4.644$, $P < 0.05$, Dunnett's test). The cisplatin-treated group showed a significant decrease in sweet diet intake in the test day of 1 and 2 ($F(5,48) = 2.976$, $P < 0.05$, Dunnett's test). Pre-administration of ondansetron abolished the decrease in sweet feed intake in the cisplatin group. In the control group, there was no significant decrease in sweet food intake on any test day, but rather there was a tendency to increase intake on test days 3 to 5 compared to conditioning days. This result revealed that eating behavior was suppressed even in rats that had acquired CTA, even against hunger. It was also suggested that this feeding suppression occurs with the involvement of the nervous system mediated by the 5-HT3 receptor. This suggests that the central mechanism of CTA may be related to the pathogenesis of eating disorders.

### [3O09-01-02]
### Circasemidian, circadian, and longer-period activity rhythms in caffeine-treated molecular clock deficient mice

*Satoru Masubuchi[1], Takako Yano[1], Komatsu Kouji[1], Keisuke Ikegami[1], Takeshi Todo[2], Wataru Nakamura[3] (*[1]Department of Physiology, Aichi Medical University School of Medicine, [2]Department of Radiation Biology and Medical Genetics, Graduate School of Medicine, Osaka University, [3]Department of Oral-Chrono Physiology, Graduate School of Biomedical Sciences, Nagasaki University*)

Mammalian circadian rhythms are driven by the transcriptional-translational feedback loop of clock genes in the hypothalamic suprachiasmatic nucleus. However, chronic methamphetamine treatment induces circadian activity rhythms in arrhythmic animals with suprachiasmatic nucleus lesions or clock gene deletions. Activation of dopaminergic neurotransmission by methamphetamine is considered to induce activity rhythms. Adenosine antagonizes the actions of dopamine at heteromers of dopamine and adenosine receptors (dopamine D1 and adenosine A1 receptors, dopamine D2 and adenosine A2A receptors). In this study, we considered that adenosine inhibition acts similarly to methamphetamine, and administered an antagonist of adenosine A1 and A2A receptors, caffeine, in drinking water. Chronic caffeine treatment extended the circadian activity period of wild-type mice under constant darkness. The circadian period extension continued for three weeks after the replacement of caffeine with water. Chronic caffeine treatment induced circasemidian (~12 h), circadian, and longer-period activity rhythms in clock gene deficient, cryptochrome (Cry) 1 and Cry 2 double knockout mice under constant darkness. These activity rhythms changed periods spontaneously over time and became arrhythmic upon caffeine withdrawal. In humans, rhythms with shorter or longer than 24 h periods are hypothesized to cause internal desynchronization of the sleep-wake rhythm from the ~ 24 h body temperature rhythm under temporal isolation. Circasemidian rhythms are hypothesized to cause afternoon sleepiness and nap. Caffeine-induced rhythms may help in understanding rhythms with not around 24 h periods in humans.

### [3O09-01-03]
### Elucidating the function of dorsomedial hypothalamus in regulation of circadian body temperature rhythm

*Ryusuke Yoshimoto[1,2], Ruth Li[2,3], Arisa Hirano[2,4], Takeshi Sakurai[2,4] (*[1]Ph.D. Program in Humanics, School of Integrative and Global Majors, University of Tsukuba, [2]International Institute for Integrative Sleep Medicine, [3]Doctoral Program in Medical Sciences, [4]Faculty of Medicine, University of Tsukuba*)

Most organisms display 24-hour periodic rhythms, known as circadian rhythms. In mammals, the suprachiasmatic nucleus (SCN) located in the hypothalamus regulates these circadian rhythms in . One of these rhythms, the circadian rhythm of core body temperature (CRT) is considered to play a crucial role in entraining peripheral clocks and in regulating the sleep-wake cycle and metabolism. To clarify the neural regulation of CRT, we focused on the dorsomedial hypothalamus (DMH), which is known to contain body-temperature-regulating neurons. Moreover, the SCN projects to the DMH area. Therefore, we propose that the DMH neurons regulate CRT by receiving the time-dependent signal from the SCN. To explore the relationship between the DMH neurons and CRT, we investigated whether the DMH activity fluctuates in a time-dependent manner. These mice are into Light-Dark cycle of 24 hours (Zeitgeber time: ZT) and we sampled these mice every 4 hour by perfusion fixation. (ZT0, ZT4, ZT8, ZT12, ZT16, ZT20). And we performed immunostaining using anti-c-Fos antibody, a maker for neural activation. As a result, the DMH activity is troughed at ZT4 and increased in active phase, which suggest that the DMH has time dependent neural activity.In addition, we examined the effect of inhibiting the DMH on CRT using a neurotoxin, tetanus toxin light chain (TeNTLC). This tetanus toxin inhibits release of neurotransmitter in the synapse. We expressed TeNTLC in the DMH regions of wild type mice and recorded body temperature and locomotor activity before and after expression of TeNTLC. We discovered that CRT was attenuated after inhibiting the DMH with TeNTLC, although these mice sustained body temperature around 36-38 degree Celsius. Interestingly, the circadian rhythm of locomotor activity is also disrupted. These results suggest that the DMH is involved in the regulation of CRT.
In the future, we will perform real-time monitoring of the neuronal activity in the DMH and identify the cell types of neurons involved in the regulation of body temperature rhythm. Understanding the function of DMH in the generation of CRT contributes to clarifying the entrainment of the circadian clock and the regulation of sleep and metabolism.

### [3O09-01-04]

### The effect of the previous night's sleep quality on Salience and Default Mode Networks during the daytime

*Yasuka Sahara[1,2], Makiko Yamada[1], Yoshiyuki Hirano[1,3,4], Daisuke Matsuyoshi[1], Haruki Nishimura[1,5], Yasunori Aizawa[1,6], Noriaki Yahata[1], Eiji Shimizu[2,3,4], Takayuki Obata[1,3,4] ([1]*Quantum Life and Medical Science Directorate, National Institutes for Quantum Science and Technology,* [2]*Graduate School of Medicine, Chiba University,* [3]*Research Center for Child Mental Development, Chiba University,* [4]*United Graduate School of Child Development, Osaka University,* [5]*The Ohara Memorial Institute for Science and Labour,* [6]*Graduate School of Medicine, Tohoku University*)

【Introduction】 Although fMRI is a widely used tool for non-invasive examination of human brain function, sampling bias may decrease the reliability of fMRI data. Amongst the various causes of sampling bias, the awareness level of subjects inside a scanner has been reported to impact resting state fMRI (rsfMRI) data. However, the effect of prior sleep quality on rsfMRI data has not been discussed. To establish a method that reduces the variability in fMRI data due to fluctuations in awareness level, we investigated the relationship between rsfMRI data during the daytime and sleep quality the night before. Technical aspects of this study were presented at JSMRM 2023. 【Materials & Methods】 Seventeen healthy participants (27.3±1.5 years old, 3 male) had two sets of tests where sleep quality and rsfMRI data were measured on two consecutive days. We measured sleep quality with a portable EEG sleep monitor (Sleep Profiler, IMI CO., LTD). The rsfMRI data was acquired over 12 minutes with the subject's eyes open. To assess the relationship between sleep quality and brain function, the salience network (SN) and default mode network (DMN) were chosen as regions of interests (ROIs) for ROI-to-ROI analysis. Correlation coefficients between the responses in each of the 7 nodes of SN and the 4 nodes of the DMN were calculated, giving an overall total of 55 correlation coefficients for the two ROIs. We investigated the effect of prior sleep quality on the connectivity between each node in the two networks with multiple regression analysis, with ROI-to-ROI connectivity as the dependent variable and sleep quality (total sleep time, Wake After Sleep Onset (WASO), REM ratio, Spindle duration (SpD), NREM (N1, N2, N3) ratio) as the independent variables.
【Results】 We removed the ratio of N1 and N3 from the independent variables for avoiding multicollinearity. Only the SN-DMN and intra-SN connectivities were found to be significantly related to the independent variables representing sleep quality, with standardized coefficients of each model were 0.567 ($p = .0005$, pFDR = .028) and 0.539($p = .0011$, pFDR = .030)), respectively. WASO had a negative association with SN-DMN ($p < .0001$). WASO and SpD were positively related to SN ($p = .046$ and $p < .0001$, respectively).
【Conclusion】 Sleep quality during the preceding night may affect the SN-DMN and intra-SN connectivity on the following day.
【COI disclosure】 The authors disclose COI in relation to this presentation (Employment position).

### [3O09-01-05]

### Phosphorylation of DNA-binding domains of CLOCK-1 BMAL1 complex is essential for PER-dependent inhibition in mammalian circadian clock.

*Yuta Otobe[1], Eui Min Jeong[2], Shunsuke Ito[1], Yoshitaka Fukada[1], Jae Kyoung Kim[2], Hikari Yoshitane[1,3] ([1]*Department of Biological Sciences, School of Science, The University of Tokyo,* [2]*Department of Mathematical Sciences, Korea Advanced Institute of Science and Technology,* [3]*Circadian Clock Project, Tokyo Metropolitan Institute of Medical Science*)

In mammals, CLOCK and BMAL1 proteins form a heterodimer that binds to E-box sequences and activates transcription of target genes, including *Per*. Translated PER proteins then bind to the CLOCK-BMAL1 complex to inhibit its transcriptional activity. However, the molecular mechanism and the impact of this PER-dependent inhibition on the circadian clock oscillation remain elusive. We previously identified Ser38 and Ser42 in a DNA-binding domain of CLOCK as phosphorylation sites at the PER-dependent inhibition phase. In this study, knockout rescue experiments showed that non-phosphorylatable (Ala) mutations at these sites shortened circadian period, whereas their constitutive-phospho-mimic (Asp) mutations completely abolished the circadian rhythms. Similarly, we found that non-phosphorylatable (Ala) or constitutive-phospho-mimic (Glu) mutations at Ser78 in a DNA-binding domain of BMAL1 also shortened the circadian period or abolished the rhythms, respectively. The mathematical modeling predicted that these constitutive-phospho-mimic mutations weaken the binding of the CLOCK-BMAL1 complex and that the non-phosphorylatable mutations inhibit the displacement (reduction of DNA-binding ability) of the CLOCK-BMAL1 complex from DNA by PER. Biochemical experiments supported the importance of these phosphorylation sites for displacement of the complex in the PER2-dependent inhibition. Our results provide direct evidence that phosphorylation of CLOCK-Ser38/Ser42 and BMAL1-Ser78 plays a crucial role in the PER-dependent inhibition and the determination of circadian period.

### Oral

### [3O09-02]
### Pathophysiology, Study Methodology

March 30, 9:50 - 10:50, Room 9

### [3O09-02-01]

### Reinstating olfactory bulb-derived limbic gamma oscillations alleviates depression-like behavioral deficits in rodents

*Yuichi Takeuchi[1] ([1]*Facult. Pharmaceut. Sci., Hokkaido Univ.*)

Although the etiology of major depressive disorder remains poorly understood, reduced gamma oscillations is an emerging biomarker. Olfactory bulbectomy, an established model of depression that reduces limbic gamma oscillations, suffers from non-specific effects of structural damage. Here, we show that transient functional suppression of olfactory bulb neurons or their piriform cortex efferents decreased gamma oscillation power in limbic areas and induced depression-like behaviors in rodents. Enhancing transmission of gamma oscillations from olfactory bulb to limbic structures by closed-loop electrical neuromodulation alleviated these behaviors. By contrast, silencing gamma transmission by anti-phase closed-loop stimulation strengthened depression-like behaviors in naive animals. These induced behaviors were neutralized by ketamine treatment that restored limbic gamma power. Taken together, our results reveal a causal link between limbic gamma oscillations and depression-like behaviors in rodents. Interfering with these endogenous rhythms can affect behaviors in rodent models of depression, suggesting that restoring gamma oscillations may alleviate depressive symptoms.

### [3O09-02-02]

### TRP channel on the blood vessels may be the sensor for the atmospheric pressure change inducing migraine.

*Hitoshi Maeda[1], Shunichi Kuwana[1] ([1]*Uekusa Gakuen University*)

Spreading depression (SD), a wave of cellular depolarization that propagates slowly across the brain surface followed by suppression of brain activity, has been presumed to be the physiological substrate of the migraine aura. However, its generation source has not been obvious yet. We examined whether SD can occur when the atmospheric pressure changes. We made a specific chamber attached a vacuum valve, which can artificially reduce internal pressure, and directly imaged cortical activity using the autofluorescence of mitochondrial flavoproteins, which was presented previously at FAOPS2018. When the pressure in the chamber began to decrease, there was an enhancement change in fluorescence from the tissue around the cerebrovascular vessels, and it was found that the fluorescence intensity increased by a barometric pressure drop of about 5 hPa, and it spread diffusely to neighboring tissues. The fluorescence of the blood vessels decreased in reverse. When the barometric pressure drop was stopped, the fluorescence changes also returned to normal. It was reported previously that chronic migraine model mice created by frequent administration of NTG are prone to SD, and the spreading of the fluorescence change in the brain surface was more clearly appeared in migraine model than wild type mice. The propagation of the fluorescence changes observed was similar in response to the SD reported last time, and it was possible to induce SD by ordinary electrical stimulation after the barometric pressure stimulation experiment, so it was considered to be a reaction equivalent to SD. We checked whether SD is caused by activation of TRP channels present in blood vessels due to changes in atmospheric pressure causing dilation of cerebral vessels. When an agonist of TRPV4 was administered in the vicinity of the blood vessels, SD occurred. Therefore, environmental changes such as atmospheric pressure changes can cause SD, and it was suggested that the reason is the activation of TRP channels on the blood vessels.

### [3O09-02-03]

### Development of therapeutics targeting tumor blood vessels derived from brain tumor stem cells

*Hiroyuki Michiue[1] ([1]*Okayama University Neutron Therapy Research Center*)

Many malignant tumors, including glioblastomas, have tumor blood vessels that feed the tumor tissue. VEGF (vascular endothelial growth factor) antibody (bevacizumab) has been reported to be effective in treating colorectal cancer, but its therapeutic efficacy in many malignancies, including glioblastoma, has not been demonstrated. However, its therapeutic efficacy in many malignant tumors such as glioblastoma has not been demonstrated. We propose a new therapeutic strategy targeting tumor-derived endothelial cells (TDECs), which differentiate into vascular endothelial cells from brain tumor stem cells. We have confirmed that TDECs differentiate into endothelial age cells by culturing 005 mouse-derived brain tumor stem cells in a hypoxic, vascular endothelial cell culture medium for 5 days. We confirmed the absence of VEGFR-2 expression, the primary target of VEGF, in endothelial cells. They also found that treatment with Axitinib, a VEGF receptor inhibitor, reduced the number of VEGFR-expressing tumor vessels and increased the number of non-VEGFR- expressing tumor vessels in a mouse brain tumor model. In previous reports, TDEC-derived tumor vessels accounted for about 10% of the tumor vessels, which is similar to our results. On the other hand, we observed that VEGFR inhibitor administration did not decrease tumor vessels, but increased tumor vessels composed of TDEC. Next, to develop a drug for TDEC tumor prefectures, we cultured the cells on Matrigel and simultaneously administered the drug to confirm the inhibitory effect of the drug on angiogenesis. To target tumors that spread into the normal brain, drug repositioning was chosen over existing drugs that cross the blood-brain barrier. The anti-angiogenic effects of 19 drugs, mainly antidepressants, were confirmed, and sertraline (SSRI) was finally selected. Sertraline showed no effect on VEGFR-2 expressing normal endothelial cells, but only on tumor vessels by TDEC, and RNA-Seq analysis confirmed that it inhibited vascular growth by Lama4 and Ang2, The results confirmed that concurrent administration of Axitinib and Sertraline had a superior prognostic effect in a mouse model. We will determine the dosage and other details for further clinical studies.

### [3O09-02-04]

### New Pathophysiology of Human Aortic Dissection through Single-Cell RNA-seq Analysis

*Kazuaki Yoshioka[1], Kenji Iino[2], Tomohiro Iba[1,3], Aya Matsui[1], Hirofumi Takemura[2], Hisamichi Naito[1] ([1]*Department of Physiology, Kanazawa University Graduate School of Medical Sciences,* [2]*Department of Cardiovascular Surgery, Kanazawa University Graduate School of Medical Sciences,* [3]*Department of Cellular and Molecular Function Analysis , Kanazawa University Graduate School of Medical Sciences*)

Human aortic dissection (AD) is an acute condition characterized by hemorrhage into the tunica media of the aortic wall, which can lead to vessel rupture. AD has various causes, but all cases share common features, such as tissue remodeling. Vasa vasorum (VV), defined as the vessels of vessels, are small blood vessels that supply nutrients and oxygen to the walls of larger blood vessels. Although adventitial angiogenesis is a well-established feature contributing to lesion progression in AD, the cellular heterogeneity within endothelial cells (EC) has not been thoroughly characterized. To gain insight into the pathogenesis of AD, our goal was to comprehensively profile the cellular composition of the adventitial VV in AD and identify molecular changes within each EC population. In this study, we conducted single-cell RNA sequencing (scRNA-seq) on outer lesions of ascending aorta from AD patients. Clustering analysis of the transcriptional profiles from 7,633 cells revealed 15 clusters representing 8 EC types: venous, arterial, capillary, post-capillary venule, tip cell, proliferating, immature, and angiogenic ECs. Comparing our findings with publicly available datasets of the human normal aorta and non-dissecting aortic diseases, we observed a transition from venous to proliferative immature angiogenic ECs, accompanied by a hypoxic response characterized by HIF1A upregulation and the accumulation of carbonic anhydrase IX (CAIX), a mediator of hypoxia-induced stress response, in the adventitial and medial VV. Integrating our scRNA sequencing data with histological analysis confirmed VV dysfunction in AD. The presence of immature angiogenic structures was associated with the accumulation of inflammatory macrophages. These findings suggest that acute AD progresses from the adventitial region with VV dysfunction, making it a valuable target for future therapeutic strategies.

### [3O09-02-05]

### Effect of catecholamine synthesizing pathway within malignant peripheral nerve sheath tumors on cancer stemness and its potential as a therapeutic target.

*Haruyoshi Katayama[1,2], Takuto Itano[1,2], Eiji Nakata[1], Toshifumi Ozaki[1], Atsushi Fujimura[2] ([1]*Department of Orthopaedic Surgery, Okayama University Graduate School of Medicine, Dentistry and Pharmaceutical Sciences,* [2]*Department of Cellular Physiology, Okayama University Graduate School of Medicine, Dentistry and Pharmaceutical Sciences*)

**[**Introduction**]** Malignant peripheral nerve sheath tumors (MPNSTs) are soft-tissue sarcomas that are derived from the Schwann cell lineage which show a poor prognosis. We previously reported that exogenous adrenaline (Ad) contributes to the enhanced cancer stemness of MPNSTs (Huang R. Biochem Biophys Res Commun 2021). The aim of this study was to clarify the existence of catecholamine synthesizing pathway in MPNST tumor cells and its effect on maintaining cancer stemness, as well as its potential as a therapeutic target.**[**Methods**]** Three human MPNST cell lines (FMS-1, HS-PSS, HS-Sch-2) and a mouse Schwann cell line, IMS32, were used in this study. Western blotting (WB) and immunofluorescence staining (IF) were performed to confirm the presence of catecholamine synthesizing pathway and catecholamines (Ad, noradrenaline) in each cell line. We also used shRNA and inhibitors of catecholamine synthesizing pathway to confirm changes in self-renewal capacity, maintenance of undifferentiation states, and tumorigenic capacity. We also examined the safety and synergistic effect of the inhibitors in combination with doxorubicin, the first-line drug for soft-tissue sarcoma treatment by MTS assay.**[**Results**]** WB and IF confirmed the presence of synthesizing pathway and catecholamines in MPNST. Knockdown of each synthase resulted in a decrease in stem cell surface marker, a transcriptional regulator and its target genes, and both sphere and colony forming capacity. Knockdown of Dopa decarboxylase (DDC) of tumor cells decreased the tumorigenic capacity in Xenograft mice compared to control tumor cells. MTS assay showed that the IC50 of the DDC inhibitor, benserazide, for tumor cell lines was 10-15 µM, whereas that of IMS32 was 91.7 µM. We also showed that the effective concentration of doxorubicin was decreased by pre-treatment with benserazide.**[**Discussion**]** The presence of the catecholamine synthesizing pathway in MPNST cells and the possibility of reducing cancer stemness by inhibiting the pathway were confirmed. In addition, the synergistic effects with existing chemotherapy drug and safety of the inhibitor were also confirmed.

### Poster Presentation

Day 1
(March 28, 13:00 - 14:20)

[1P] Neurophysiology, Neuronal cell biology - Neural network
[1P] Neurophysiology, Neuronal cell biology - Neurochemistry
[1P] Neurophysiology, Neuronal cell biology - Neurons, Synapses
[1P] Neurophysiology, Neuronal cell biology - Glia
[1P] Neurophysiology, Neuronal cell biology - Higher brain function
[1P] Neurophysiology, Neuronal cell biology - Motor function
[1P] Neurophysiology, Neuronal cell biology - Sensory function, Sensory organ
[1P] Neurophysiology, Neuronal cell biology - Others
[1P] Molecular physiology, Cell physiology - Membrane transport
[1P] Molecular physiology, Cell physiology - Ion channels, Receptors
[1P] Molecular physiology, Cell physiology - Others
[1P] Muscle
[1P] Digestion, Digestive system
[1P] Oral physiology
[1P] Circulation
[1P] Reproduction
[1P] Endocrine
[1P] Autonomic nervous system
[1P] Environmental physiology
[1P] Nutritional and metabolic physiology, Thermoregulation
[1P] Behavior, Biological rhythm, Sleep
[1P] Pathophysiology
[1P] Sensory function

### Poster

### [1P]
### Neurophysiology, Neuronal cell biology
### Neural network

March 28, 13:00 - 14:20, Poster Room

### [1P-001]

**Projection pattern-specific difference in noradrenergic modulation of excitatory synaptic transmission in the superior collicular neurons underlying stress-induced modulation of defensive behavior.**

*Madoka Narushima[1], Junichi Nabekura[1] ([1]*National Institute for Physiological Sciences, Division of Homeostatic Development*)

The superior colliculus (SC) is a brain area that conducts sensory-motor behaviors. SC neurons receive various inputs including excitatory inputs from the retina and the sensory cortex, inhibitory inputs from the thalamus and interneurons in the SC, and modulatory inputs such as dopamine or noradrenaline (NA). NA input from the locus coeruleus to the SC is enhanced in response to stress and accelerates SC-related sensory-motor behaviors such as defensive behaviors to the visual looming stimuli (Li et al., 2018). However, because of the diversity of the neurons and inputs in the SC, the cellular mechanisms for the NAergic modulation in the SC is not well understood. We focused on NAergic modulation of the excitatory retinal and V1 cortical synapses, to the SC neurons projecting to the lateral posterior (LP) nucleus in the thalamus or to the para bigeminal nucleus (PBGN) in the midbrain, which are both related to visual-guided defensive behaviors such as escape or freezing (Shang et al., 2019). We performed slice patch clamp recordings from LP or PBGN-projecting SC neurons labeled by the retrograde fluorescent tracer. Because it was difficult to separately stimulate retinal and cortical inputs, we took advantage of optogenetic techniques that expressed channelrhodopsin 2 (ChR2) in retinal or cortical fibers by using transgenic mice lines or virus injection. Interestingly, NA differently regulated retinal and cortical synapses onto PBGN or LP projecting neurons through different subtypes of receptors. Whereas retinal or cortical inputs onto PBGN projecting neurons in the superficial layer of the SC were regulated by Gi/o-coupled alpha2 or Gs-coupled beta receptor respectively, neither of them onto LP projecting neurons was affected by NA application. We also investigated the contribution of the cortical input to stress-induced modulation of defensive behaviors. Our findings provide new insights into how NA regulates the stress-induced change of sensory-motor behaviors through the modulation of excitatory inputs onto the SC neurons.

### [1P-002]

**Anatomical analysis of neuronal projection from the amygdala to the motor cortices in rats.**

*Masaki Okubo[1,2], Taichi Goto[1,2], Ichiro Takashima[3], Shinya Yamamoto[1,2], Nobuo Kunori[1] ([1]*AIST,* [2]*University of Tsukuba,* [3]*Daiichi Institute of Technology*)

Motor function, the ability to regulate motor behavior in response to a changing environment, is one of the fundamental abilities of living organisms. The primary and secondary motor cortices (M1 and M2) are known to be important in motor function: the M1 is involved in motor execution and motor learning, whereas the M2 is thought to contribute to fine motor control and cognitive functions, such as decision making, in addition to the role of the M1 described above. The neural activity in the motor cortices underlying the motor functions could be affected by neuronal input from other brain areas. Indeed, the M1 and M2 receive neuronal inputs from many brain areas through the thalamocortical, cortico-cortical, and subcortical-cortical pathways. Although many studies have elucidated various neuronal inputs to the M1 and M2, the significance of subcortical inputs in motor functions is relatively understudied.
The basolateral amygdala (BLA) is a subcortical brain region projecting to the frontal cortex. The BLA is involved in various emotional behaviors (e.g. fear and addiction), which have been expected to influence motor functions. Previous studies indicated that the BLA neurons project directly to the motor cortices. However, the anatomical and functional details of the projection have not been fully examined.
In the present study, the BLA neurons projecting to the M1 and M2 were clarified by using a retrograde tracer. Cholera toxin b subunits with different fluorescent properties were injected into the M1 and M2 in the same rat, and retrogradely labeled neurons in the BLA were confirmed histologically. Our results showed that the BLA neurons innervating the M1 and M2 were predominantly located in the rostral part of BLA. Furthermore, some neurons projected to the contralateral hemisphere, and others innervated the M1 and M2 simultaneously. These results suggest that emotional signals from the BLA may influence both motor and cognitive functions.

### [1P-003]

**Modulation of Brain Neural Functional Connectivity Through Physical Exercise**

*Seiichiro Amemiya[1,3], Natsuko Kubota[2], Ichiro Kita[3] ([1]*RIKEN Center for Brain Science,* [2]*Tokyo International Univ.,* [3]*Tokyo Metropolitun Univ.*)

Physical exercise has positive effects on brain function, including learning and memory, emotion and executive functions. The brain works as a dynamic neural network where multiple regions coordinate their activities (functional connectivity). Thus, it is conceivable that physical exercise can modulate the functional brain network. Furthermore, previous researches have indicated that the beneficial effects of exercise on brain functions diverge depending on intensity of the exercise. Therefore, it is possible that the functional brain network recruited by exercise depend on the intensity of the exercise. This study aims to investigate whether physical exercise can alter the network structure of functional connectivity in response to varying exercise intensities. We quantified the neural activity in multiple brain regions, including cortex, basal ganglia, limbic are, hypothalamus and brain stem, during treadmill running at different intensity (0, 15, 25 m/min) in male Wistar rats, using c-Fos immunohistochemistry. Subsequently, we assessed the strength of functional connectivity between all pairs of brain regions based on the correlation of c-Fos positive cell counts. The network structure of functional connectivity was analyzed using graph theory. Physical exercise increased neural activity in each brain region, while the network density (the number of connections between different brain regions) decreased in an exercise intensity-dependent manner. Additionally, differences in several network properties associated with small-world networks and scale-free networks were observed depending on the intensity of exercise. These findings suggest that physical exercise can reconfigure the network structure of functional connectivity based on exercise intensity.

### [1P-004]

### Hippocampal β rhythm suppresses GABAA-antagonist-induced epileptiform activities in rat brain slices

Toyohiro Sawada[1], Masaya Shigemoto[1], Hirofumi Arai[1], Ryusei Maeda[1], *Kiyohisa Natsume[1] ([1]*Kyushu Institute of Technology*)

Low dose application (30 μM) of the cholinergic agent carbachol (CCh) induces intermittent β frequency burst activity, termed CIBA, in rat hippocampal slices. $GABA_A$ receptor antagonists induce epileptiform activities in the slices. Co-application of CCh at concentrations sub-threshold of CIBA induction suppressed the generation of the part of the activities. Pre-established CIBA prevents the induction of epileptiform activities by $GABA_A$ antagonism, and the activities were induced only after CCh removal. This suggests that CIBA mainly prevents the generation of epileptiform discharges. This prevention was suppressed by application of the adenosine $A_1$ receptor antagonist, 8-cyclopentyltheophylline. We propose that CIBA could be a suitable *in vivo* model of β rhythms. Our results suggest that during generation of β rhythms, release of endogenous adenosine from hippocampal neurons results in an extracellular increase in concentration that suppresses the generation of epileptiform discharges through activation of $A_1$ receptors.

### [1P-005]

### Distribution and function of glycinergic neurons in the ventromedial medulla (VMM) sending projections to monoaminergic nuclei

*Takahiro Suzuki[1], Shingo Soya[1], Shuntaro Uchida[2], Yuki Saito[1], Yoan Cherasse[1], Takeshi Sakurai[1] ([1]*WPI-IIIS, University of Tsukuba,* [2]*RIKEN BDR*)

[Introduction]
Inhibitory glycinergic neurons in the ventromedial medulla (VMM) ($Gly^{VMM}$) comprise a group of neurons that send projections to somatic motor neurons, including those in the anterior horn of the spinal cord and motor nuclei in the brain stem. Inhibition of this population has been shown to eliminate REM-atonia. However, it is worth noting that $Gly^{VMM}$ neurons also projects to various regions other than somatic motor neurons, such as the locus coeruleus (LC) and dorsal raphe nucleus (DR), and the precise distribution and function of these projections remain unclear.[Aim]Our primary focus is on $Gly^{VMM}$ neurons that send projections to the LC ($Gly^{VMM\rightarrow LC}$ neurons). We aimed to investigate their inputs and outputs organizations, and to gain better understanding of their functions in sleep/wakefulness regulation and other physiological processes.

[Methods]
To explore the input-output organization of $Gly^{VMM\rightarrow LC}$ neurons , we utilized the cTRIO method with GlyT2-icre mice. This method involves the use of a modified rabies virus vector (SADΔG(EnvA)) that infects retrogradely. These manipulations resulted in the expression of mCherry in $Gly^{VMM\rightarrow LC}$ axons and GFP in the input cells. We then conducted immunohistochemistry to label mCherry and GFP, followed by capturing microscopic images for analysis of their expression patterns.

For fiber photometry imaging, a Cre-dependent AAV carrying GCaMP6 was injected into the VMM. Concurrently, electrodes for EEG recording were implanted, along with optical fibers, enabling simultaneous recording of EEG and $Gly^{VMM}$ neurons activity during sleep. Two weeks later, we performed simultaneous EEG and fiber photometry recordings to analyze the activity of $Gly^{VMM}$ neurons during each sleep phase.

[Results]
The input cells of $Gly^{VMM\rightarrow LC}$ neurons were found in regions including the SC (superior colliculus) and PB (parabrachial nucleus). Also, $Gly^{VMM\rightarrow LC}$ axons were found in regions such as DR and P5 (peritrigeminal zone), and in the spinal cord such as the IML (intermediate matter lateral nucleus). In particular, axons to somatic motor neurons were not found in $Gly^{VMM\rightarrow LC}$.

Photometry results suggest that the activity of $Gly^{VMM}$ is increased during REM sleep. This is consistent with the fact that $Gly^{VMM}$ is involved in REM-atonia.

[Discussion]
Our present results suggest that the distribution and function of $Gly^{VMM\rightarrow LC}$ are different from those of $Gly^{VMM}$neurons projecting motor neurons. The marked axonal projections by $Gly^{VMM\rightarrow LC}$ neurons in the IML suggests that these neurons may be implicated in the regulation of the sympathetic preganglionic fibers.

Future prospects include recording pathway-specific neural activity by photometry and examining the effects of pathway-specific manipulation on sleep-wake state using DREADD.

### [1P-006]

### Role of TRPA1 on respiratory network in the isolated brainstem-spinal cord preparation from neonatal rat

*Naoko Masutani[1], Ryo Kawabata[2], Yuki Kosaka[1], Kohei Koga[2], Akiko Arata[1] ([1]*Dept. of Physiome , Hyogo Medical University,* [2]*Department of Neurophysiology, Hyogo Medical University*)

TRPA1 is a thermosensory TRP channel known to be expressed primarily in myelinated A-delta and unmyelinated C fibers of peripheral nerves found in the axons of the spinal cord, vagus, and trigeminal nerves. Sensation of nociceptive spinal signals is projected via the dorsal horn to the lateral parabrachial nucleus of the pons (LPB), also known as the inspiratory-expiratory (I-E) phase-switching system, which controls respiratory rate. Contribute to TRPA1 is also known to be sensitive to hypoxia, which is important for respiratory control. However, the relationship between TRPA1 and respiratory rhythm has not been fully investigated. In this study, we investigated the effects of TRPA1 on respiratory rhythm in pons-medulla-spinal cord and medullary-spinal cord preparations isolated from 0- to 2-day-old rats. Respiratory activity was recorded from the cervical fourth (C4) ventral nerve root. Additionally, neurons in the pons were recorded using the patch-clamp technique. In this study, TRPA1 reduced respiratory rate. This inhibition was blocked by application of the GABAA antagonist bicuculline, suggesting that the GABAergic inhibitory system mediated the respiratory depression by TRPA1. Neurons (probably non-respiratory neurons) were recorded from the LPB in the pons, which receives ascending projections from peripheral sensation. TRPA1 excited these LPB neurons and increased excitability of the parabrachial circuit. On the other hand, neurons recorded from KF (possibly I-E neurons) were excited by TRPA1. This neuron responded to temporary excitation and was then inhibited. This response may be a switch mechanism to descending inhibitory system of respiration. In with pons preparations, a slight respiratory enhancement was observed due to the effects of hypoxia, and TRPA1 antagonists blocked this enhancement. These results suggested that hypoxia excites both central chemoreceptor neurons in the medulla and possibly pontine-mediated GABAergic neurons. TRPA1 may contribute to both the pontine affective pathway ascending from peripheral sensation and the medullary survival pathway regarding oxygen tension.

### [1P-007]

### Therapeutic mechanism of an anticholinergic agent on parkinsonian symptoms: a monkey study

*Yuki Hayashida[1], Satomi Chiken[2,3], Nobuhiko Hatanaka[2,3], Hideki Hida[4], Atushi Nambu[5] ([1]*Nagoya City University Medical School,* [2]*Section of Multilayer Physiology, National Institute for Physiological Sciences,* [3]*Physiological Sciences, SOKENDAI,* [4]*Department of Neurophysiology and Brain Science, Nagoya City University Graduate School of Medical Sciences,* [5]*Section of NBR Project, National Institute for Physiological Sciences*)

Parkinson's disease (PD) is one of common neurological disorders and characterized by akinesia, rigidity, tremor, and non-motor symptoms. PD is caused by degeneration of dopaminergic neurons in the substantia nigari pars compacta. PD can be successfully treated by L-DOPA, which supplements reduced dopamine. Anticholinergic agents, such as biperiden, are also effective and have long been used. The balance between dopamine and acetylcholine in the striatum is considered to be important, and anticholinergic agents may decrease acetylcholine in the striatum and keep balance. However, neurophysiological bases of the effectiveness of anticholinergic agents in the system level remain to be clarified. In the present study, we examined neuronal activity of the external segment of the globus pallidus (GPe) in a normal and an MPTP-treated PD monkeys, one of the targets of the striatum, before and after systemic administration of biperiden (2-5 mg, i.m.). We also examined the responses evoked by electrical stimulation of the primary motor cortex and supplementary motor area. GPe neurons of the normal monkey spontaneously fired at high firing rates (40-80 Hz), and cortical stimulation induced a triphasic response composed of early excitation, inhibition, and late excitation. In the PD monkey, firing rates of GPe neurons were lower, and cortically evoked late excitation was longer in comparison with those in the normal monkey. Biperiden administration ameliorated PD motor signs. After biperiden administration, spontaneous firing rates of GPe neurons were reduced, inhibition evoked by cortical stimulation was extended, and late excitation was reduced in both the normal and PD monkeys. These activity changes in the GPe will give us neurophysiological rationale for PD treatment by anticholinergic agents.

### Poster

### [1P]
### Neurophysiology, Neuronal cell biology
### Neurochemistry

March 28, 13:00 - 14:20, Poster Room

### [1P-008]

### Task control based on multi-area brain activities with an optical brain-machine interface

*Konosuke Kitajima[1], Akinori Sato[1], Kentaro Ibuka[1], Kei Ito[1], Ryosuke Takeuchi[1], Fumitaka Osakada[1,2,3,4] ([1]*Laboratory of Cellular Pharmacology, Graduate School of Pharmaceutical Sciences, Nagoya Univ.*, [2]*Laboratory of Neural Information Processing, Institute for Advanced Research, Nagoya Univ.*, [3]*Inst of Nano-Life-Syst, Nagoya Univ.*, [4]*Inst for Glyco-core Res, Nagoya Univ.*)

A brain-machine interface (BMI), a system for directly connecting external devices and the brain, enables us to control machines by brain signals. A BMI requires recording and identifying brain activity that guides specific behaviors. However, the existing systems hinder recording a wide range of brain activity simultaneously because electrodes are implanted locally in the brain to measure neural signals. To address this issue, we developed an optical BMI system combined with wide-field $Ca^{2+}$ imaging that acquires neural activity across dorsal cortical areas. In this study, we expressed the $Ca^{2+}$ sensor jGCaMP7f in the cerebral cortex of mice by retro-orbital injection of BBB-crossing AAVs. We trained the mice to perform a lever-press task in which a reward is delivered for pressing a lever in response to auditory cues. Wide-field $Ca^{2+}$ imaging identified brain regions that were activated before, during, and after their lever-press action. We subsequently examined whether the mice could control the task by switching the reward trigger from the lever to the neural activity that we defined. The mice adapted to this optical BMI system and controlled the task based on their volitional modulation of neural activities. We also examined how brain activity changed during the operation of the BMI-controlled task. Various dimensionality reduction analyses demonstrated that mice spontaneously altered their macroscopic brain activity and propagation across the cortex to control this optical BMI. These results indicate that mice can volitionally generate defined patterns of neural activity across areas to operate the task and that utilizing multi-area activities for BMI operation improves BMI performance. This novel BMI system could contribute to the macro-scale analysis of brain plasticity. Understanding the mechanism underlying the brain adaption to a BMI in a brain-wide manner will be crucial for developing neuroprosthetics to restore nervous system function.

### [1P-009]

### Analysis of sodium channel turnover using mice that can distinguish between old and new Nav1.6 protein ~Toward understanding mechanism of homeostasis of neuronal function~

*Kohei Yamamoto[1], Yoshifumi Ookouchi[1], Daisuke Yoshioka[1], Manabu Abe[3], Kenji Sakimura[3], Yasushi Okamura[1,2] ([1]*Integrative physiology, Grauate Schoo; of Medicine/ Faculty of Medicine, Osaka University*, [2]*Grauate School of Frontier Biosciences , Osaka University*, [3]*Brain research Institute, Niigata university*)

Our neurons survive and maintain biological function throughout our life. The axon initial segment (AIS) and node of Ranvier in neurons are important structures for the generation of action potentials and saltatory conduction, respectively. However, the mechanism by which the functions of the AIS and node are maintained throughout life is still unknown. To understand this mechanism *in vivo*, we established a mouse line (Nav1.6-Flex) that enables us to analyze the turnover of the voltage-gated sodium channel, Nav1.6, which are localized in the AIS and node, regulating the membrane excitability. Replacement of old to new Nav1.6 can be visualized by the conversion of Nav1.6-GFP (old) to Nav1.6-tdTomato (new) under the activity of Cre recombinase, which the expression is driven by AAV. We focused on the retinal ganglion cells (RGCs) in this study, because it has two structural advantages: AIS and node of RGCs can be observed separately in retina and optic nerve, because mouse RGCs has unmyelinated axon in retina. Additionally, it is easy to apply functional manipulation (e.g. monocular deprivation and dark condition) to RGCs. We examined the replacement of GFP signals to tdTomato signals in AIS of retina and node of optic nerve on day 3, 7 and 14 after the injection. In AIS of retina 14 day after the injection, we observed tdTomato signal, but GFP signal was not seen in the same region. On the other hand, in node of optic nerve, both signals were comparable. These results suggest that turnover rate of Nav1.6 in AIS is faster than the turnover that occurs in the node. Analysis is currently underway in the hippocampus as well. In addition, we will perform experiments that change turnover rate such as aging and monocular deprivation. These results will lead to a better understanding of mechanisms of neuronal homeostasis. COI : No

### [1P-010]

### The modulation of mitochondrial function with increase of NAD/NADH ratio protects blood-brain barrier disruption in acute brain injury

*Heonjong Yoo[1], Woosuk Chung[2], Eungseok Oh[3], Min Joung Lee[4], Jong Huh An[4,5,6], Hyun Joo Choi[4,5,6], Chang Hee Pyo[4,5,6], Jun Young Heo[4,5,6] ([1]*Department of Obstetrics and Gynecology, Chungnam National University School of Medicine*, [2]*Department of Anesthesia and Pain Medicine, Chungnam National University Hospital, Daejon, South Korea*, [3]*Department of neurology, Chungnam National University School of Medicine*, [4]*Department of Biochemistry, Chungnam National University School of Medicine, Daejeon, South Korea*, [5]*Department of Medical Science, Chungnam National University School of Medicine, Daejeon, South Korea*, [6]*3.Infection Control Convergence Research Center, Chungnam National University School of Medicine, Daejeon, South Korea*)

The blood-brain barrier (BBB) is composed of endothelial cells, astrocytes, and pericytes known as neurovascular unit. Endothelial cells have junctional proteins to maintain BBB integrity as a first line of barrier. Maintenance of BBB is essential to protect the brain from the infiltration of pathogens and prevent acute brain injury by oxidative stress. To date, the treatment of anticoagulants and thrombolysis has been used for stroke patients after the occurrence of the diseases. According to our previous reports, the maintenance of mitochondrial Oxphos in cerebral endothelial cells is critical for BBB integrity. But therapeutic agents for the maintenance of BBB and the prevention of BBB disruption still need to be developed. In this study, we found a supplement that can increase NAD/NADH ratio with enhancement of mitochondrial respiration before the induction of oxidative stress by oxygen-glucose deprivation (OGD) in the cerebral endothelial cells. Evans blue assay are performed after OGD, ischemic condition with the supplement pretreatment *in vitro* BBB model and Junctional proteins expression are investigated. Furthermore, we examined mitochondrial complex expression and mitohormetic response to investigate the effect of the supplement on mitochondrial functions. Pretreatment of the supplement in mouse intracerebral hemorrhage model alleviated the injury by increasing the junctional protein expression and mitochondrial modulation in cerebral endothelial cells. These results demonstrate that modulating mitochondria in cerebral endothelial cells can prevent BBB disruption.

### Poster

### [1P]
### Neurophysiology, Neuronal cell biology
**Neurons, Synapses**

March 28, 13:00 - 14:20, Poster Room

### [1P-011]

**Diminished spontaneous firing of striatal cholinergic interneurons in aged mice.**

*Etsuko Suzuki[1], Toshihiko Momiyama[1] ([1]*Jikei University, Sch. of Med. Dept. Pharmacology*)

It has been reported that the spontaneous firing of striatal cholinergic interneurons (ChINs) increases with postnatal development. However, changes in spontaneous firing frequency and firing properties during aging has not been investigated yet. In this study, cell-attached and whole-cell patch-clamp studies were carried out to investigate changes in firing properties of ChINs during aging. Brain slices were prepared from 2–3-month-old, 11–12-month-old and 24-month-old mice of either sex. Frequencies of spontaneous firing at 2–3-month-old, 11–12-month-old and 24-month-old were 4.55 ± 1.01 Hz (n = 18), 8.73 ± 2.28 Hz (n = 8) and 3.38 ± 0.84 Hz (n = 15), respectively. Firing frequency at 24 month of age was significantly lower than that of 11–12-month-old (p = 0.028). Since spontaneous firing appeared to be irregular in 24-month-old mice, the coefficient of variation (CV) of the inter-event interval of spontaneous firing was analyzed. CV at 24 months of age (0.62 ± 0.08, n = 15) was significantly larger than that of 2-3-month of age (0.34 ± 0.05, n = 18, P = 0.024), indicating that the regular pattern of spontaneous firing is disrupted in 24-month-old mice. Moreover, the voltage sag induced by hyperpolarizing current injection was analyzed. Notably, the sag ratio at 24-month-old was smaller (1.1 ± 0.02, n = 6) than that observed at 2–3-month-old (1.2 ± 0.01, n = 7, P = 0.017) and 11–12-month-old (1.2 ± 0.02, n = 5, P < 0.01), which implies a decline in the h-current responsible for the voltage sag during the aging. Considering the well-established significance attributed to the h-current in the modulation of spontaneous firing within ChINs, these observations collectively suggest that the attenuation of the h-current may serve as an underlying mechanism for the diminution in firing activity.

### [1P-012]

**A *de novo* mutation of the voltage-gated $K^+$ channel Kv2.1 in a patient with cerebral cortical dysplasia.**

*Tenpei Akita[1], Takuya Hiraide[2], Kenji Uematsu[3], Sachiko Miyamoto[2], Mitsuko Nakashima[2], Masayuki Sasaki[3], Atsuo Fukuda[4], Mitsuhiro Kato[5], Hirotomo Saitsu[2]
([1]*Division of Health Science, Department of Basic Nursing, Hamamatsu University School of Medicine,* [2]*Department of Biochemistry, Hamamatsu University School of Medicine,* [3]*Department of Child Neurology, National Center Hospital, National Center of Neurology and Psychiatry,* [4]*Department of Neurophysiology, Hamamatsu University School of Medicine,* [5]*Epilepsy Medical Center, Showa University Hospital*)

The voltage-gated $K^+$ channel Kv2.1 (KCNB1) is a major channel for delayed rectifier $K^+$ current and is widely expressed in neurons of the cerebral cortex and hippocampus. Starting from previous reports including ours (Sci Rep, 5:15199, 2015), many cases have been reported showing that mutations in this channel gene cause developmental and epileptic encephalopathy (DEE), resulting in intractable and severe psychomotor developmental disorders. Recently, we found a case of *de novo* mutation of this gene associated with cerebral cortical dysplasia including periventricular ectopic gray matter (heterotopia), polymicrogyria, and abnormal corpus callosum. The mutation occurred in the S1 transmembrane segment of the voltage-sensing domain of the channel (p.(Ala192Thr)). Clinical findings included delayed language development, intellectual disability, attention deficit hyperactivity disorder, late-onset epileptic seizures, and peripheral axonal neuropathy, although these findings were milder than those seen with other mutations in this gene. Regarding the electrophysiological properties of the channels, both channel activation and inactivation at membrane potentials below −20 mV were significantly reduced by the mutation. It remains to be elucidated how this change in channel properties caused the cortical dysplasia. Kv2.1 expression is known to increase mainly after birth. Since no other pathological gene mutations were found in the patients' whole exome analysis, it is possible that the effects of low levels of mutant Kv2.1 expression during fetal life may be involved in the formation mechanism of the cortical dysplasia, and further investigation is needed.

### [1P-013]

**Developmental organization of mouse cochlear nucleus during embryogenesis revealed by optical recording with voltage-sensitive dye**

*Yoko Momose-Sato[1], Katsushige Sato[2] ([1]*Department of Nutrition and Dietetics, College of Nutrition, Kanto Gakuin University,* [2]*Department of Health and Nutrition Sciences, Faculty of Human Health, Komazawa Women's University*)

The central issue in developmental neuroscience is when and how neural synaptic networks are established and become functional within the central nervous system (CNS). Investigations of neural network organization have been hampered because conventional electrophysiological methods have some technical limitations. In the present study, we applied the multiple-site optical recording technique with a voltage-sensitive dye and surveyed the developmental organization of the auditory system in the mouse embryo. Stimulation of the cochlear nerve in E12 to E15 mouse embryos elicited EPSP-related optical responses in the lateral brainstem near the entry of the cochlear nerve, which corresponded to the auditory sensory nucleus, the cochlear nucleus. The EPSP was mediated by glutamate and mainly dependent on NMDA receptors. The EPSP was detected from E12, indicating that functional connections between the periphery and neurons in the cochlear nucleus are established at this stage. The results show that postsynaptic responses in the cochlear nucleus are expressed much earlier than reported previously (E15: Marrs and Spirou, 2012) and suggest that functional synapses are generated soon after the arrival of afferent fibers (E12: Lu et al., 2011) and even before morphological differentiation of both the pre- (E11-E15: Ruben, 1967) and post-synaptic neurons (E10-E14: Taber Pierce, 1967) has been completed.

### [1P-014]

### Potentiation of metabotropic glutamate receptor 1 in the neonatal hippocampus.

*Megumi Taketo[1] ([1]*Dept. Physiology, Facult. Med, Kansai Medical Univ.*)

Metabotropic glutamate receptors (mGluRs) are G-protein coupled receptors which participate in the regulation of cell excitability and synaptic plasticity in the central nervous system. Eight subtypes of mGluRs are classified into three groups. Among them, group I mGluRs consist of mGluR1 and mGluR5, couple to $G_{q/11}$ proteins, while Group II receptors consist of mGluR2 and mGluR3, couple to $G_{i/o}$ proteins. Group I receptors activate phospholipase C and mobilize intracellular $Ca^{2+}$, in addition, regulate several channels and other signaling proteins. Cajal-Retzius (CR) cells locating hippocampal marginal zone, are early-developed neurons and control the radial migration of neurons by production and secretion of the glycoprotein, reelin. CR cells also modulate network activity through projecting their dendrites to other neurons. Previous studies demonstrated that mGluR1 is expressed by hippocampal CR cells, however, the physiological roles of mGluR1 in CR cells remain unclear. A growing body of evidence suggests that there is interaction between G-protein-coupled receptors. In this study, calcium imaging revealed that activation of mGluR1 elevated intracellular $Ca^{2+}$ concentration ($[Ca^{2+}]_i$) in CR cells. The $[Ca^{2+}]_i$ elevation was still observed in the presence of several $Ca^{2+}$ channel blockers. The effect of activation of other G-protein coupled receptors on the mGluR1-mediated $Ca^{2+}$ responses was then examined. Adenosine receptors are also G-protein coupled and subdivided into $A_1$, $A_{2A}$, $A_{2B}$, and $A_3$. Among these, $A_1$ receptor ($A_1R$) basically interacts with $G_{i/o}$ and inhibits adenylate cyclase. $A_1R$ expressed in the central nervous system, has several functions including transmitter release and the reduction of neuronal excitability. In CR, application of $A_1R$ agonists potentiated mGluR1-induced elevation of i $[Ca^{2+}]_i$. Activation of $A_1R$ itself did not change $[Ca^{2+}]_i$. The potentiated responses were independent of extracellular $Ca^{2+}$ and dependent on G protein. The potentiation of mGluR1-induced $[Ca^{2+}]_i$ elevation was also enhanced by activation of group II mGluRs. These results suggest that mGluR1 and other G protein-coupled receptors cooperatively influence postnatal hippocampal development by facilitating $Ca^{2+}$ mobilization in CR cells.

### [1P-015]

### Microglia Mediate Synaptic Loss in the Early Stages of Alzheimer's Disease

*Zhongtian Guo[1,2], Daisuke Kato[1,2], Reon Kondo[1], Ikuko Takeda[1,2], Hiroaki Wake[1,2]
([1]*Department of Anatomy and Molecular Cell Biology Nagoya University Graduate School of Medicine,* [2]*Division of Multicellular Circuit Dynamics, National Institute for Physiological Sciences National Institute of Natural Sciences*)

Alzheimer's disease (AD) express cognitive decline with pathological features of amyloid β accumulation. Synapse numbers reduces in the early stages of AD that ultimately cause the cognitive decline. Microglia, a sole immune cell in CNS, is traditionally known to contribute on the pathology via the removal of amyloid plaques. In addition, microglia activate to promote chronic inflammation to promote cognitive decline. However, it is less known when and how the reduction in synapse number occurs in AD pathology, and whether microglia contribute to this synaptic loss. In this study, to identify the precise timing of synaptic reduction in AD pathology and the contribution of microglia to this process, we observed the time course of amyloid plaque, synapse and microglia using 2-photon microscopy over time and quantified their changes in APP knock-in mice. Our findings revealed that amyloid plaques were first detected very sparsely at 7 weeks of age, were barely detectable around the dendrites at 12 weeks of age. In contrast, the synaptic density decreased significantly from 12 weeks of age and continued to decline over time. Furthermore, we found that the inter-cluster distance was significantly increased, and the remaining synapses formed the clusters. Furthermore, to investigate whether microglia are associated with synaptic reduction, we pharmacologically ablated microglia. the results suggests that the progression of synaptic reduction was delayed in microglia ablated AD mice. We further analyzed the contact between microglial processes and synapses, found that synapses with low microglial contact were more prone to loss. These results provide insight into the temporal and spatial features of synaptic loss in the early stages of AD and suggest an association between microglia and this process. In the future, we will determine which brain regions the removed synapses are projected from and whether the microglial approach to synapses changes depending on the source of projection.

### [1P-016]

### Effects of a GluA1 missense mutation associated with neurodevelopmental disorders and intellectual disabilities on the heteromeric AMPA receptor function with GluA2

*Taku Uchida[1], Kogo Takamiya[1] ([1]*Department of Neuroscience, Section of Integrative Physiology, Faculty of Medicine, University of Miyazaki.*)

The cause of neurodevelopmental disorders (NDDs) including ASD and ADHD remains elusive. However, research points to the influence of genetic factors and abnormal synaptic morphology in neurons, suggesting that changes in synaptic function play a significant role in these disorders. NDDs itself has no intellectual disability (ID) requirement, but is subdivided according to the degree of language ability especially in ASD. Over the past decade, a series of missense mutations in the *GRIA1*, which gene encodes GluA1, an AMPA receptor subunit, has been discovered in patients with NDDs and ID. These mutations alter AMPA receptor function, potentially contributing to the pathogenesis of those disorders through synaptic alterations. AMPA receptors often creating heteromeric forms of GluA1 and GluA2. Notably, the functional properties of monomeric mutant GluA1 and heteromeric forms would differ, with the latter being more relevant to pathology. To investigate this, we focused on the A636T missense mutation in GRIA1 found in patients diagnosed with ADHD and/or ASD with ID. A636T mutant GluA1 exhibits unique physiological characteristics, including a huge agonist-induced current and a persistent inward current known as the "Initial Inward Current." Co-expression of A636T mutant GluA1 with wild-type GluA1 in HEK293T cells eliminated the Initial Inward Current. Heteromeric AMPA receptors with GluA2 displayed a similar current amplitude to wild-type receptors but exhibited delayed currents. These effects were also observed in spontaneous EPSCs when A636T mutant GluA1 was expressed in cultured mouse neurons. These results suggest that the pathogenesis of NDDs with ID associated with the GluA1 A636T mutation could be linked to prolonged episodic currents rather than instantaneous large current or tonic inward currents.

### [1P-017]

### Proton dynamics associated with glutamate transport into synaptic vesicles in living neurons

*Hiroyuki Kawano[1], Yoshihiro Egashira[2], Takahiro Nakayama[1], Yasunori Mori[3], Shigeo Takamori[1] ([1]*Doshisha University,* [2]*Osaka Medical and Pharmaceutical University,* [3]*University of Yamanashi*)

Emerging evidence suggests that neurons in various brain regions release multiple neurotransmitters even from single synaptic vesicles. Since the dependence of neurotransmitter uptake on a proton motive force (either membrane potential, $\Delta\Psi$, or chemical gradient, $\Delta$pH) varies depending on the charge of neurotransmitters, the charge imbalance associated with neurotransmitter transport might influence the proton motive force, potentially affecting the uptake of one neurotransmitter by another. This concept, known as 'vesicle synergy' for neurotransmitter uptake, has limited supporting evidence in physiological conditions, especially for two principal excitatory and inhibitory neurotransmitters: glutamate and GABA. To gain deeper insights into vesicle synergy involving glutamate transport, we aim to monitor the proton dynamics associated with glutamate transport in living hippocampal neurons. To achieve this, we prepared cultured neurons with undetectable glutamate release by knocking down vesicular glutamate transporter 2 (VGLUT2) in neurons derived from VGLUT1-knockout mice. We monitored vesicular pH by expressing an optimal pH indicator, mOrange2, with pKa of 6.5 positioned in the luminal loop of synaptophysin (Syp-mOr) either in wild-type neurons or in VGLUT-free neurons. We observed a significant delay in the post-exocytic decay of Syp-mOr2 fluorescence in the absence of VGLUTs. This delay primarily stemmed from the reduced rate of re-acidification of VGLUT-free vesicles by ~ 5-fold, rather than that of endocytosis. Furthermore, while the resting vesicular pH remained unchanged (approximately 5.9), the luminal buffering capacity of VGLUT-free vesicles was significantly reduced compared to wild-type vesicles (approximately 60 mM/pH for wild-type versus approximately 40 mM/pH for VGLUT-free neurons). These results indicate that glutamate loading is associated with an increase in net $H^+$ influx, presumably due to the activation of V-ATPase through the dissipation of membrane potential by the transport of negatively charged glutamate. Our results align with classical acridine orange assays using isolated synaptic vesicles but conflict with a recent report demonstrating the facilitation of re-acidification in the absence of glutamate, proposing a glutamate:$H^+$ mechanism. Since proton dynamics associated with glutamate transport differ substantially from those associated with GABA transport that we reported earlier, our new results suggest the potential for vesicle synergy resulting from the co-packaging of glutamate and GABA into the same vesicle.

### Poster

### [1P]
### Neurophysiology, Neuronal cell biology
### Glia

March 28, 13:00 - 14:20, Poster Room

### [1P-018]
### Hypotonic stimuli potentiate TRPV4 activation in astrocytes

*Koji Shibasaki[1], Kabashima Rimika[1], Amane Tateishi[1] ([1]*Laboratory of Neurochemistry, Department of Nutrition Science, University of Nagasaki*)

We previously reported that TRPV4 is expressed in ~30% subpopulation of astrocytes in brain, and the TRPV4 activation leads to gliotransmitter (ATP and glutamate) release and increases synaptic transmission (Shibasaki et al. JBC 2014, J. Anesth 2016). It has been reported that TRPV4 is activated by various stimuli such as warmth temperature (>34°C), hypotonic stimulus, extracellular arachidonic acid and mechanical stimulus. All TRP channels have unique properties called as synergistic effects. Therefore, when we apply two different agonists, thresholds of each agonist can be effectively reduced. As the result of the changes of thresholds, we can observe significant TRP channel activation by combination of two different agonists. These backgrounds indicate that TRPV4 can be potentiated by combinational application of two different agonists. In this study, we examined the possibility by an electrophysiological and an Ca2+-imaging experiments. Combinational application of lipid and hypotonic stimuli significantly potentiated the TRPV4 activation. These results indicate that endogenous TRPV4 is strongly activated by multiple ligands, and lead to enhanced gliotransmitter release in naïve astrocytes.

### [1P-019]
### Alteration in the Blood-Brain Barrier and microglia in a mouse model of Alzheimer’s disease.

*Lingnan Hou[1], Jinglei Cheng[1], Zhongtian Guo[1], Wanying Li[1], Daisuke Kato[1,2], Hiroaki Wake[1,2] ([1]*Department of Anatomy and Molecular Cell Biology, Nagoya University Graduate School of Medicine,* [2]*Division of Multicellular Circuit Dynamics, National Institute for Physiological Sciences, National Institutes of Natural Sciences*)

Alzheimer's disease (AD) is a neurodegenerative disorder characterized by pathological features such as the accumulation of amyloid plaques and tau tangles. Previous study using Gd-enhanced human magnetic resonance imaging has shown that alteration of the blood-brain barrier (BBB) is detected in the early stages of AD. BBB disruption may adversely affect neurological function by activating microglia with invading inflammatory mediators in the central nervous system (CNS). In contrast, the infiltration of immune cells into the CNS following BBB disruption may facilitate the removal of Aβ via phagocytosis of microglia and macrophages. However, the time course of BBB disruption in AD pathology as well as the contribution of microglia to this process are not known. In this research, to identify the time course of BBB disruption and their effect on neuron, we used the APP knock-in mouse as AD model (provided by Dr. Takaomi Saido at Riken) to observe the structure of the BBB on different time course using electron microscopy. Our findings revealed a reduction in tight junction length in AD model mice, starting around 16~24 weeks. We further investigated the dynamics of microglia and BBB permeability over time using *in vivo* two-photon microscopy to quantify these changes. We found that microglia start to accumulate with blood vessels from 12~13 weeks before the loss of tight junction. These findings suggest that in the AD model, the activation of microglia by amyloid-beta could precede and potentially lead to changes in BBB permeability. We are currently using a combination of two-photon microscopy and immunoelectron microscopy to identify more precise timing of BBB disruption and to investigate whether immune cells as well as microglia contribute to BBB disruption.

### [1P-020]
### Astrocyte driven dismantling of S1 chronic pain circuits requires recruitment of microglia

*Dennis Lawrence Cheung[1], Ikuko Takeda[2], Junichi Nabekura[1] ([1]*The National Institute for Physiological Sciences,* [2]*Nagoya University*)

Chronic pain is a major public health problem, affecting one in five individuals yet lacking effective treatment options. We have previously reported that dismantling putative maladaptive S1 neuronal circuits effectively cures allodynia in the partial sciatic nerve ligation mouse model (Takeda et al. 2022, Nature Communications). This treatment involved transiently blocking afferent input to S1 whilst simultaneously activating S1 astrocytes through either the CNO-DREADD system or transcranial direct-current stimulation. During the treatment period, S1 dendritic spine turnover significantly increased, resulting in enduring relief from allodynia.

In this study, we now examine the downstream mechanisms by which astrocyte activation cures allodynia. Significantly, we find that the recruitment of microglia is a central underlying mechanism. S1 astrocyte activation triggers proliferation of microglia within S1 and their adoption of a more activated morphology. Furthermore, S1 astrocyte activation fails to cure allodynia if microglia are prior ablated by PLX or if microglial activation is suppressed by minocycline. However, the interaction between astrocytes and microglia does not appear to be linearly unidirectional. Solely activating microglia by administering lipopolysaccharide immunogen fails to cure allodynia. Thus, we are now investigating bi-directional signalling pathways between astrocytes and microglia and how this facilitates increased dendritic spine turnover.

### [1P-021]

### *PlxnA3*-deficient mice exhibit agenesis of corpus callosum and decreased cell number of astrocytes in the midline glial structure: indusium griseum

*Kazunori Yukawa[1], Md. Mosharaf Hossain[1], Takamasa Tsuzuki[1], Ikuko Takahashi[2], Takahiko Kawasaki[3], Takayuki Negishi[1] (*[1]Department of Physiology, Faculty of Pharmacy, Meijo University, [2]Radioisotope Center, Faculty of Pharamacy, Meijo University, [3]Brain Function Laboratory, National Institute of Genetics*)

The corpus callosum (CC) is the largest commissure that forms the major interhemispheric connection. Plexin-A3 (PlxnA3) is a transmembrane receptor for semaphorins, a family of axon guidance factors playing crucial roles during nervous system development. *PlxnA3* is expressed in the cingulate cortex from which callosal pioneer axons extend toward the midline for the initiation of CC development. However, the role of PlxnA3 in CC development remains unknown. In the analyses to examine the role of PlxnA3, the midline crossing of neuropilin1-expressing callosal pioneer axons at embryonic day 17.5 (E17.5) was significantly impaired in *PlxnA3* KO mice under a genetic background of BALB/cAJcl mice as compared with wild type (WT) mice ($c^2$ test, $P < 0.05$). *PlxnA3* KO mice exhibited agenesis of corpus callosum in the rostral and medial part of the CC at postnatal day 0.5 ($c^2$ test, $P < 0.05$). Immunohistochemistry (IHC) to examine PlxnA3 expression in the cortical midline at E17.5 disclosed the expression of PlxnA3 in GFAP-positive (GFAP+) mature astrocytes residing in indusium griseum (IG) and midline zipper (MZ), and in GLAST+ cells. To examine the formation of guidepost structures in *PlxnA3* KO mice, IHC was performed in both WT and *PlxnA3* KO brains at E17.5 with antibodies against GFAP and Sox9, a glial nuclear marker expressed in radial glia, glial progenitors and mature glia. As a result, both GFAP+ cells and Sox9+ cells in the IG region of *PlxnA3* KO brains were significantly less than those of WT ($P < 0.05$, Student's *t* test). Thus, both GFAP+ cells and Sox9+ cells in the IG region of *PlxnA3* KO brains were significantly less than WT, but not in the MZ region. Axon guidance molecules are produced and secreted by mature GFAP+ astrocytes in the IG. To reveal the expression pattern of *Slit2* mRNAs in the IG of *PlxnA3* KO brains at E17.5, we performed *in situ* hybridization of *Slit2* mRNAs followed by IHC with anti-GFAP antibody. *Slit2* mRNAs were expressed in GFAP+ cells in IG and MZ in WT brains at E17.5. In contrast, *Slit2* mRNAs were hardly localized in IG, and rather diffusely distributed in the cortical midline in *PlxnA3* KO brains at E17.5. Taken together, our results indicate that PlxnA3 is crucial for the formation of IG in which mature astrocytes gather and secrete axon guidance cues like Slit2 to navigate the pioneer axons for CC development.

### [1P-022]

### Functional roles of a synaptic adhesion-like molecule, LRFN2, in gliomagenesis

*TAKUMA YOSHIZAWA[1], Kenny Daun[1], Tadateru Fukami[2], Naoko Morimura[1], Seiji Hitoshi[1] (*[1]Department of Integrative Physiology, Shiga University of Medical Science, Otsu, Shiga, [2]Department of Neurosurgery, Shiga University of Medical Science, Otsu, Shiga*)

Glioblastoma multiforme (GBM) is the most common malignant and lethal primary brain tumor. Recent findings indicate that GBM cells are integrated into neuronal circuits via synapses between neurons of surrounding healthy tissue and GBM cells. Facilitation of neuron-GBM glutamatergic synaptic pathway contributes to not only generating tumor-related epilepsy but also accelerating tumor growth, invasion, and recurrent disease. In this study, we postulated that a synaptic adhesion-like molecule, *LRFN2*, which physically and functionally interacts with excitatory postsynaptic components such as the NMDA receptor, AMPA receptor, and PSD-95, is involved in the vicious synaptic signaling between neurons and GBM cells. We first analyzed expression levels of *LRFN2* using the clinical glioma datasets obtained from the Cancer Genome Atlas (TCGA) and analyzed an association between the *LRFN2* expression levels and survival rates in patients with various glioma classes. We found that a low *LRFN2* expression was associated with a shorter survival rate than patients with glioma expressing high levels of *LRFN2*. Furthermore, the *LRFN2* expression was lower in GBM than in low-grade glioma (LGG), suggesting that the highly malignant GBM possesses lower *LRFN2* expression patterns. Using patient-derived GBM lines, we introduced a *LRFN2* expression vector and confirmed its overexpression by western blotting and immunocytochemical staining. We then asked whether *LRFN2* overexpression alters cell proliferation and suppresses tumor growth. To further elucidate this notion, we will examine the malignant transformation of *LRFN2*-overexpressing GBM cells by direct contact with healthy neurons. We will also determine their functional integration to gain deeper insight into mechanisms that could lead to new therapeutic strategies targeting this malignant tumor.

### [1P-023]

### Ameliorative effects of a dopamine D1-like receptor agonist SKF-81297 on traumatic brain injury model rats by preventing neuroinflammatory reactions

*Ayane Takenaga[1,2], Mohammed E Choudhury[2], Naoki Abe[3], Noriyuki Miyaue[1], Masahiro Nagai[1], Tasuku Nishihara[3], Junya Tanaka[2] (*[1]Department of Clinical Pharmacology and Therapeutics, Graduate School of Medicine, Ehime University, [2]Department of Molecular and Cellular Physiology, Graduate School of Medicine, Ehime University, [3]Department of Anesthesia and Perioperative Medicine, Graduate School of Medicine, Ehime University*)

### Poster

### [1P]
### Neurophysiology, Neuronal cell biology
### Higher brain function

March 28, 13:00 - 14:20, Poster Room

### [1P-024]

### Comparison of neural activity in the dorsal premotor cortex and lateral prefrontal cortex during a path-planning task

*Kazuhiro Sakamoto[1,2], Naohiro Saito[2], Shun Yoshida[2], Hajime Mushiake[2] ([1]*Tohoku Medical and Pharmaceutical University,* [2]*Tohoku University*)

To achieve goals in an environment where unexpected events may occur, possible actions must be planned and prepared. The dorsal premotor cortex (PMd) and the lateral prefrontal cortex (lPFC) are known to play important roles in the selection of motor actions based on the environmental context and in the executive functions required to plan goal-directed behavior, respectively. We have previously recorded single-unit activity in the monkey lPFC during a path-planning task, in which the subject had to plan multiple steps of cursor movements to reach a final goal in a maze-like display, and reported dynamic neuronal activity reflecting behavioral planning, i.e. neurons encoding a final goal early in the preparation period and an immediate goal later in the period (final-to-immediate neurons) (Sakamoto et al., 2008, 2013, 2020). On the other hand, neurons encoding potential actions have been reported in the PMd, i.e. cells that are active immediately after the presentation of a cue suggesting a possible behavior until the behavior is executed or until the execution of the behavior proves impossible (potential action neurons) (see Cisek, Kalaska, 2010). We have also obtained preliminary results that are consistent with this (Toyoshima et al., 2011). The present study analyzes PMd cells that are task-selectively active during the preparatory period and examines how they encode potential behavior and how their encoding properties differ from those of final-to-immediate neurons in the lPFC. This work was supported by JSPS KAKENHI Grant Number 17K07060, 20K07726 (Kiban C), MEXT KAKENHI Grant Number 20H05478, 22H04780 (Hyper–Adaptability) and SIP Project Phase 3 (Development of Quantum Spin Sensor and Development and Demonstration of Use Cases). The authors declare no competing financial interests.

### [1P-025]

### Relationship between individual differences in taste temporal order judgments, subjective feelings of taste perception and eating behaviors

*Makoto Wada[1], Kouji Takano[1], Tatsu Kobayakawa[2] ([1]*National Rehabilitation Center for Persons with Disabilities,* [2]*National Institute of Advanced Industrial Science and Technology*)

Due to differences in receptor type (i.e., ionotropic or metabotropic), salty taste is thought to be detected faster than sweet taste. In fact, latency of gustatory evoked magnetic fields of salty tastant occurs faster than sweet’s one by approximately 100 ms. However, from our previous study, when presented with a mixture of sweet and salty, there were large individual differences in whether the salty or sweet taste was perceived first, suggesting that participants with higher empathy quotient score tended to judge the sweet tastant as coming first, in contrast to the receptor characteristics. By comparing the results of the psychophysical experiments with those of the participants interviewed, the present study examined how these individual differences affect eating behavior and subjective taste perception.Participants (n=30) were required to reproduce the orders of tastants (Salty: 0.5M NaCl, Sweet: 1M Sucrose, Their mixture) by pressing buttons with forced-choice manners. The participants were also required to answer questionaries about subjective feelings of taste perception (Chen et al., 2023) and eating behaviors (Chen et al., 2022). In most of the participants, orders were correctly reproduced with stimulus onset asynchrony of approximately 500 ms. When the mixture was delivered, “sweet first” judgment ratio was correlated with empathy quotient score. We found the “sweet first” judgment ratio was also correlated with scores of questions “I feel the sweet taste lasting a long time in the mouth” (p = .002) and “I can't eat hot food” (p = .043). In contrast, a score of a question “I don't like mixed tastes, like mixture from sweet and sour” was correlated with "salty first" judgment (p = .031) . The results suggest that the magnitude of variance in neural representations related to sweet taste might influence the perception of the mixture. We will investigate why this tendency is related to empathy traits.

### [1P-026]

### Pathophysiological roles of DAMPs for depression using a zebrafish model

*Akinobu Sohda[1], Ryohei Umeda[2], Kenshiro Shikano[1], Hitoshi Teranishi[1], Shinichiro Kume[3], Takatoshi Hikida[4], Reiko Hanada[1] ([1]*Oita-Univ. Faculty of Medicine Department of Neurophysiology,* [2]*Oita-Univ. Faculty of Medicine Department of Advanced Medical Science,* [3]*Oita-Univ. Faculty of Medicine Department of Pathophysiology,* [4]*Osaka-Univ. Institute for Protein Research Laboratory for Advanced Brain Functions*)

In contemporary society, the prevalence of depression is on the rise, largely driven by various stressors, posing a significant societal concern. However, many aspects of the pathophysiological mechanisms of depression remain unclear. Recent reports have highlighted the involvement of brain inflammation in the pathophysiology of depression, with increasing attention not only to inflammatory cytokines but also to the role of damage-associated molecular patterns (DAMPs), endogenous molecules associated with damage. DAMPs are released from impaired nerve cells under cellular stress, amplifying brain inflammation and contributing to the worsening of depression. Nevertheless, the dynamics of DAMPs and their roles in disease progression are largely uncharted. Clodronate is known to have a pharmacological effect of inhibiting the release of ATP, a type of DAMPs, from immune cells, although its impact on depression has not been reported. Therefore, this study focused on neuro-modulatory factors among DAMPs, particularly ATP and adenosine (ADO), and investigated the effects of clodronate in a zebrafish model of depression, while conducting an analysis of its role.

“The learned helplessness model” is widely used for evaluating depression, but there have been few reports on its use with zebrafish. In this model, the learned helplessness model was established by zebrafish to two days of inescapable conditions, involving the presentation of a cue followed by unavoidable electric shocks. On the third day, a setup was used to allow the zebrafish to escape the electric shocks by moving to a neighboring chamber within 13 seconds of the cue presentation. As expected, it exhibited a significant reduction in the number of successful escapes compared to the control group, confirming the establishment of the learned helplessness model.

Subsequently, the study examined the effects of clodronate, which has an inhibitory effect on ATP release when administered, on the behavior of zebrafish. Clodronate was orally administered for 12 days. Novel Tank Diving tests were conducted on the 1st, 3rd, and 8th days, with learned helplessness training on the 10th and 11th days, followed by the evaluation of the number of successful escapes on the 12th day. As a result, the impact of clodronate on psychoneurological activity became evident, and these findings are reported herein.

### [1P-027]

### Functional analysis of frontal cortex integration of reward expectation and locomotor behavior in mice

*Joao Pedro Junqueira[1], Tomofumi Otsuki[1], Jumpei Matsumoto[1,2], Tsuyoshi Setogawa[1,2], Hiroshi Nishimaru[1,2] ([1]*System Emotional Science, Faculty of Med, Univ of Toyama, Toyama, Japan*, [2]*RCIBS, Univ of Toyama, Toyama, Japan*)

The rodent dorsomedial prefrontal cortex (dmPFC), including the anterior cingulate cortex (ACC) and secondary motor cortex (M2), has been shown to integrate sensory information and motor control during the decision-making process to allow the animal to accurately time its actions to interact with its environment. Few studies, however, have investigated the role of the dmPFC in producing accurately timed sound-guided locomotor and licking responses. By training mice in a self-paced goal-directed locomotor task while manipulating neuronal activity in the dmPFC, this study examined the contributions of this region in the conversion of goal-signaling stimuli into control of locomotion and licking responses. Water-restricted mice (n = 6) could initiate trials by spontaneously starting to run while head-fixed over a horizontal disc. After the mouse ran for 1-2 seconds, a sound cue was played and, after sustaining locomotion for 1 second, the subjects were rewarded with a water drop. After 7-10 sessions, they learned to decelerate running by the time of the reward delivery. They also learned to start licking after the cue. Pharmacological suppression of dmPFC activity by $GABA_A$ receptor agonist muscimol significantly impaired the task performance by increasing the rate of trials in which mice prematurely halted their running before reaching their goal. The timing of licking onset was also significantly shifted forward, increasing the frequency of trials where mice licked before the sound cue was presented. Our results suggest that dmPFC activity is involved not only in inhibiting premature initiation of appetitive licking bursts as previously reported, but also in sustaining locomotor behavior until goals are reached. This work was supported by KAKENHI No, 23H04673 (HN); JST SPRING, Grant Number, JPMJSP2145 (TO). COI: No.

### [1P-028]

### Functional connectivity of language networks in patients with behaviorally diagnosed unresponsive wakefulness syndrome.

*Kouji Takano[1], Yoji Okahara[2], Kenji Kansaku[3] ([1]*Dept. of Rehabilitation for Brain Functions, Research Institute of National Rehabilitation Center for Persons with Disabilities*, [2]*Department of Neurological Surgery, Chiba Cerebral and Cardiovascular Center*, [3]*Department of Physiology, Dokkyo Medical University School of Medicine*)

Severe head trauma can cause disordered consciousness, exemplified by an unresponsive wakefulness syndrome (UWS) or minimally conscious state (MCS). In our previous study (Okahara et al., 2023), we used an approach combining fMRI and passive listening tasks to evaluate the level of speech comprehension (Kansaku et al., 2000), with portable brain–computer interface (BCI) modalities that were applied to elicit an active response to attentional modulation tasks (Sakurada et al., 2015). We included 10 patients who were clinically diagnosed with UWS. Significant activation distributed over the primary auditory cortex and superior temporal gyrus (STG) was found in 4 of the 10 patients, and 2 out of the 4 patients showed additional significant activation in the left middle temporal gyrus (MTG), and were able to control the BCI with reliable accuracy. Thus the 2 patients who showed both passive and active neural responses can be physiologically diagnosed as MCS. In this study, we analyzed the resting-state fMRI functional connectivity of the above 4 patients. We compared functional connectivity between patients who showed activation in the primary auditory cortex and STG (n=2) and those who showed additional activation in the left MTG (n=2), by using the CONN Toolbox (https://web.conn-toolbox.org/).
Seed-based resting-state fMRI functional connectivity analysis was recruited, and the left MTG was used as the seed region. We found significantly higher functional connectivity between the left MTG and the left IFG in the patients who showed additional activation in the left MTG ($p<0.05$, FWE corrected).
The results suggest that the functional connectivity of language networks was preserved in the patients who showed additional activation in the left MTG.

### [1P-029]

### Spontaneous High-Frequency Firings (Super bursts) of Hippocampal CA1 Neurons Cause Diversification of Ripple Firings and Memory Formation

Junko Ishikawa[1], *Dai Mitsushima[1] ([1]*Yamaguchi University Graduate School of Medicine*)

Contextual learning requires hippocampal CA1 neurons, processing spatiotemporal information of experiences. By recording multiple-unit firings of hippocampal CA1 neurons in freely moving male rats, we previously found that episodic experiences induced high-frequency spontaneous firings (super bursts), followed by an increase in ripple-firings and synaptic plasticity in CA1 neurons. Moreover, we found episode-specific diversity in the features of super bursts, synaptic plasticity, and ripple-firings. These changes were particularly prominent by strong emotional episode of “restraint stress”. Since high-frequency firing of CA1 neurons is well known to promote synaptic plasticity, we hypothesized that the super bursts trigger memory process in CA1 neurons. To test this hypothesis, we eliminated the super bursts that occur during experiencing restraint stress by using a system that immediately detects the onset of super bursts and simultaneously stimulates the hippocampal commissure to suppress CA1 neural activity. Elimination of the super bursts successfully prevented freezing behavior exhibited when the subject was placed again in a restraint stress location. Moreover, the super bursts elimination induced by restraint stress significantly reduced the increase and the diversification of ripple-firings. These results suggest a causal relationship between super bursts, the diversification of ripple firings, and memory formation in emotional experiences. The episode-specific induction of super bursts and subsequent diversification of ripple firings may play an important role in contextual memory processing in hippocampal CA1 neurons.

### [1P-030]

### The area of the mouse brain activated by electric shock for the treatment of depression was studied using the Mn MRI method

*Akio Inoue[1], Yuriko Inoue[2], Hiromitsu Ezure[2], Naruto Otsuka[2], Koichi Shiraishi[3] ([1]*Human Brain Research Center, Graduate School of Medicine, Kyoto University*, [2]*Dep. Anat., Showa Univ. Sch. Med.,*, [3]*Dev. Med. Eng. Jikei Uni. Med.*)

The recovery of depression by antidepressants requires three weeks, so the decrease in serotonin or noradrenaline is not the cause of depression. We found that the recovery of the mouse depression model, which is induced by the repeated restraint stress, by electric shock also requires three weeks as in humans. Therefore, antidepressants and electric shock may recover depression by a similar mechanism, Then, we studied which part of the brain is activated by electric shock using the Mn-MRI method. Mn ions enter into nerve cells through the membrane Ca-channel, depending on nerve activity, and Mn ions induce the increase of the T1 signal of MRI. So, the Mn-MRI method is used to measure the nerve activity in vivo. However, Mn ions inside the cells are released and disappear by nerve activation. Therefore, a mouse should be activated only when Mn ions are charged. When Mn ions are injected into the abdominal cavity, Mn ions enter into blood vessels and finally. Mn ions enter into the brain through the choroid plexus. Mn concentration in the brain is maintained for a few hours. As antidepressants work almost one day, it is difficult to apply the Mn-MRI method, but electric shock works transiently. Then, we applied electric shock for three minutes. The next day, we measured the T1 signal of the mouse brain using the Bruker 9.4T MRI machine. We found that the hippocampus is specifically activated by electric shock for either normal or depressed mice. As electric shock works for a variety of brain diseases, we concluded that the hippocampus works as a controller of the brain.

### Poster

[1P]
### Neurophysiology, Neuronal cell biology
**Motor function**

March 28, 13:00 - 14:20, Poster Room

### [1P-031]

### Three-dimensional kinematical analysis revealed spatiotemporal improvement of ankle mobility in hemidecorticated mice after undergoing therapeutic aerobic running

*Akira YOSHIKAWA[1,2], Hirokazu Ohtaki[3,4], Kazuyuki Miyamoto[4,5], SungHyek Kim[6], Kazunori Hase[7], Makoto Yoshida[7], Shotaro Kamijo[1,8], Sawa Kamimura[1,9], Nobuyoshi Koiwa[10], Masahiko Izumizaki[1] (*[1]Department of Physiology, Showa University School of Medicine, [2]Division of Health Science Education, Showa University School of Nursing and Rehabilitation Sciences, [3]Department of Functional Neurobiology, Tokyo University of Pharmacy and Life Sciences School of Pharmacy, [4]Department of Anatomy, Showa University School of Medicine, [5]Department of Emergency, Critical Care and Disaster Medicine, Showa University School of Medicine, [6]Department of Shizuoka Physical Therapy, Tokoha University Faculty of Health Science, [7]Department of Mechanical Systems Engineering, Tokyo Metropolitan University Faculty of Systems Design, [8]Department of Physiology, Showa University School of Pharmacy, [9]Department of Otorhinolaryngology Head and Neck Surgery, Showa University School of Medicine, [10]Department of Health and Science, University of Human Arts and Sciences*)

Motor dysfunction is a sequela of traumatic brain injury or stroke that frequently causes gait impairment. Treadmill aerobic running is often used as a physical exercise task in human and animal studies to elucidate the recovery of gait impairment. In rehabilitative animal studies, behavioral evaluations are often to quantify qualitative behavioral scores. However, local or small behavioral impairments are hard to assess using these methods. The purpose of this study was to demonstrate motor function after brain damage (BD) with/without aerobic running exercise ([Ex]: 10 m/min for 30 min 5 times/week) using quantitative analysis. The baseline running fitness of the experimental mice was evaluated prior to left hemidecortication (BD). The concrete method of running fitness is that 8 weeks male mice were examined an incremental running test while the pulmonary gas exchange of $O_2$ and $CO_2$ were measured. After mice underwent four weeks of the Ex regimen, their gait was recorded and evaluated using three-dimensional kinematical analysis as the quantitative analysis and compared with that of BD mice who did not undergo the Ex regimen. As a result, BD without Ex mice demonstrated significant impairment in stride, step, and stride width compared with the BD with Ex mice. Trajectory analysis revealed significant restriction in both ankles in the BD without Ex mice, and impairment in the dorsal/planter flexing was also observed. These results suggested that aerobic running exercises improved the posture, steps, and ankle joint movement in gait. Kinematical analysis allowed quantitative analysis of the limb's spatiotemporal movement in both the unaffected and affected sides in this study.

### [1P-032]

### Effects of social isolation stress on behavioral specificity and learning

*Mao Morita[1], Hideo Kawaguchi[1] (*[1]TOYO University*)

Experiencing social isolation in childhood, when the brain, body, and emotions are still developing, is said to interfere with the development of appropriate relationships with others later in life.
Studies in mice have reported that mice exposed to social isolation (SI) stress exhibit symptoms and behavioral specificities similar to depression and anxiety.
However, it is still unclear how early (just after weaning) social isolation affects brain functions such as learning ability and emotion, and what differences are observed at the genetic and protein levels.
In this study, we therefore evaluated memory, learning, and emotional behaviors in SI groups of C57BL/6 mice raised alone immediately after weaning and compared them to grouped controls.
Open field experiments, social interaction tests, elevated cross maze, novel object recognition experiments, spatial recognition experiments, and multi-directional maze tests were used for behavioral assessment.
Interestingly, in the social interaction test, the SI group showed excessive contact and strong interest in mice they had never met before compared to the control group.
In the multi-directional maze test, the SI group tended to learn maze paths more slowly than the control group.
These data suggest that social isolation during childhood affects memory and learning ability, two brain functions that are particularly important for survival.
Furthermore, it is possible that the animals have a strong desire to interact with others, but do not know how to do so appropriately and are unable to successfully navigate distance.
In addition, the introduction of environmental enrichment to improve living conditions and reduce stress is recommended in the husbandry and management of laboratory animals.
It has been shown that the introduction of environmental enrichment promotes synaptogenesis and enhances neurodevelopment and brain activity.
In this study, we attempt to reduce social isolation stress and the associated decrease in brain activity through environmental enrichment.In addition to these data, we will perform immunostaining of c-Fos, a marker of neural activity, in the brains of mice after behavioral assessment to identify brain regions affected by social isolation stress.

### [1P-033]

### Effect of infection-inducing substance Poly(I:C) on body movement in the spinal cord preparations

*Yuki Kosaka[1], Chiaki Uchida[1], Naoko Masutani[1], Hirotaka Ooka[1], Seiichi Morokuma[2], Akiko Arata[1] (*[1]Dept. of Physiome ,Hyogo Medical University, [2]Dept. of Health Science, Graduate School of Medical Sciences, Kyushu University*)

The fetal movement has a great influence on fetal development and survival rate. Poly(I:C): PIC is an immunostimulant in viral infections, and it triggers an immune response for viral infections. It is reported that the application of PIC to pregnant mice increases the risk of psychiatric disorders such as schizophrenia, mental retardation, autism, and behavioral disorders in fetal mice. However, the effects of fetal infection on motor function have not been well understood. In our previous studies, the ultrasonic observation of fetal movements of the fetal rats within the pregnant rats administered with PIC showed that PIC suppressed normal fetal movements and increased peristaltic movement, which is not normally observed (Sakuma, Yoshida, et al. 2020). These results suggest that infections may reduce fetal movement. However, the effects of PIC on the spinal cord, which causes fetal movement during the perinatal period are still unknown. In this study, we examined the effects of PIC during the perinatal stages using brainstem-whole spinal cord preparation. We recorded the cervical fourth ventral nerve activity (C4) as respiration for an index of alive and the lumber fourth ventral nerve activity (L4) as a lower limb body movement activity. The body movement activity has been reported to be suppressed by glycine in the neonatal period.So we applied Strychnine, as a glycine antagonist, to the spinal cord preparations isolated from postnatal 0-2-day-old rat to reappear the body movement that was depressed by glycine. Applying PIC under strychnine induced a significant increase in body movement followed by a small activity, which was more effective in the P0 than in the P2. The application of PIC also suppressed normal body movement activity. Both of them continued to be seen even when the application was returned to strychnine alone. These results showed that PIC changed normal body movement to body movement with shaking activity. This shaking activity may correspond to the peristaltic movements observed in behavioral experiments on fetal and pregnant rats. These indicate that infection during the fetal period may alter fetal body movement activity, triggering diseases and behavioral disorders such as schizophrenia, autism, Down's syndrome, and developmental disorders.

### [1P-034]

### Visualize the whole-brain neural activity during voluntary movement by quantitative activation-induced manganese-enhanced MRI

*Yuika Furusawa[1], Yuki Uemura[1], Kayo Nishizawa[2], Chika Matsushita[1], Jun-ichiro Enmi[3,4], Atsushi Tamura[1,5], Yoshichika Yoshioka[3,4], Kazuto Kobayashi[2], Makoto Osanai[1,3] ([1]*Osaka Univ Grad Sch Med,* [2]*Fukushima Medical Univ Sch Med,* [3]*CiNet,* [4]*Grad Sch Front Biosci, Osaka Univ,* [5]*Grad Sch Pharm Sci, Tohoku Univ Grad Sch Med,*)

Although it is known that the cerebral cortex – basal ganglia – thalamus loop circuit is involved in voluntary movement, the respective area to the motor control is not clear because of the difficulties of recording the neural activity during the motion. Parkinson's disease (PD) is known to be caused by the depletion of dopamine in the striatum, which alters the activity pattern of the basal ganglia and becomes symptomatic. However, it is not clear which brain regions of neural activity are altered in PD subjects compared with healthy subjects, particularly the differences in neural activity during motion. Therefore, we measured whole-brain neural activity during coordinated movement in the PD mice model and healthy control.PD model mice were produced by the MPTP administration. We performed the rotarod test as a coordinated motor task in healthy mice and in PD model mice, and measured whole-brain neural activity during the rotarod test by quantitative activation-induced manganese-enhanced MRI (qAIM-MRI).Manganese ion (Mn2+) can pass through voltage-dependent calcium channels (VDCCs), and is extruded very slowly from the cell. VDCCs open more frequently in highly active neurons; hence, in the presence of Mn2+ in the extracellular solution, highly active neurons accumulate larger amounts of Mn2+ than weakly active neurons. Therefore, Mn2+ is a surrogate marker of Ca2+ influx in excitable tissues. Mn2+ shortens the longitudinal relaxation time (T1) of H+, which can be quantified by MRI, and the longitudinal relaxation rate R1 (=1/T1) is proportional to Mn2+ concentration. Based on these ideas, qAIM-MRI can use R1 to measure neural activity changes in a freely moving subject.In healthy mice, several regions showed significantly higher neural activity in the mice with the rotarod test than in the mice in their home cage. In addition, many regions showed positive correlations between rotarod test scores and the neural activities. However, in PD model mice, several regions showed lower neural activities in the rotarod test group compared with the non-test group. The regions exhibited positive and negative correlations between the rotarod test scores and the neural activities. In PD model mice, positive correlations between rotarod test scores and the expression level of tyrosine hydroxylase (TH), which is an enzyme producing dopamine, were observed.

### [1P-036]

### Changes in primary motor cortex excitability and motor performance through a combined intervention of high-frequency rTMS and motor imagery

*Miyabi Toriyama[1], Amiri Matsumoto[1], Rieko Aruga[1], Miki Kaneshige[1], Nan Liang[1] ([1]*Human Health Sciences, Faculty of Medicine, Kyoto University*)

### [1P-035]

### Morphological change of climbing fibers in the cerebellar cortex are not shown at P15 but at P28 in rats of neonatal white matter injury

*Shiori Tominaga[1,2], Cha-Gyun Jung[1], Naoki Tajiri[1], Shinya Ueno[1], Yuji Watanabe[3], Hideki Hida[1] ([1]*Nagoya City University Graduate School of Medical Sciences,* [2]*Nagoya City University School of Biology & Integrated Sciences,* [3]*Nagoya City University Graduate School of Science*)

In a rat model of neonatal white matter injury (NWMI) that is made by hypoxic-ischemia at postnatal day 3 (P3), specific damage to oligodendrocyte progenitors and subsequent myelination failure was resulted in the dysfunction of the motor execution system. However, developmental changes in the motor control system are not clarified in the NWMI model yet. To answer this question, we performed immunohistochemistry of vGluT2 (a marker for climbing fibers) and Calbindin D28 (a marker for Purkinje cells) in the NWMI model at P15 when both cells are developing and at P28 when the growth advanced in both cells. Developing climbing fibers were successfully detected on Purkinje cells at P15, although the staining pattern of GluT2 was similar in both sides of the cerebellum. However, significant difference in the staining pattern of climbing fibers on Purkinje cells were unexpectedly observed at P28: the ratio of the vGluT2-positive area to cerebellar cortex was small in the ipsilateral cerebellum as compared to the contralateral side. Data suggest that the pruning of surplus climbing fibers on Purkinje cells which is detected in normal development after P15 is insufficient in our NWMI model rat cerebellum, indicating the possibility of the relation to motor dysfunction at P28.

### Poster

[1P]
### Neurophysiology, Neuronal cell biology
**Sensory function, Sensory organ**

March 28, 13:00 - 14:20, Poster Room

### [1P-037]
### Searching for novel mechanotransducer channels using cell lines derived from DRG neurons

*Mai Oda[1], Viktor V. Feketa[1], Elena O. Gracheva[1], Sviatoslav N. Bagriantsev[1] ([1]*Yale University School of Medicine*)

In vertebrates, mechanical stimulation such as touch, vibration, and stretch applied to the skin is detected by mechanoreceptor nerve endings. Mechanical stimulation evokes excitatory ionic currents via the activation of mechanotransducers (mechanically gated ion channels) in the mechanoreceptor membrane. Mouse somatosensory neurons from dorsal root ganglion (DRG) neurons generate three types of mechanically activated current (MA current) in response to mechanical indentation. Neurons with fast inactivating MA current detect light touch via the mechanotransducers channel Piezo2. On the other hand, neurons with intermediate and slow inactivating MA currents detect painful touch. However, the mechanotransducers mediating these types of MA currents remain unknown. To identify a novel mechanically gated ion channel that senses painful touch in DRG neurons, we used whole-cell patch clamp to examine the response of DRG neuron-derived immortalized cell lines (Cell #1 – Cell #9) to mechanical indentation. First, we confirmed that all cell lines were mechanosensitive, and transcriptome results for these cell lines showed that Piezo1 is highly expressed in these cell lines, but Piezo2 is not. siRNA-mediated knockdown of Piezo1 completely suppressed MA current in Cell #1, but not in Cell #6. These results suggest that Cell #6 expresses unknown mechanotransducers, different from Piezo1. Next, we examined the effect of candidate genes (ion channels and membrane proteins with unknown functions that have upregulated expression levels in Cell #6 compared to Cell #1) on MA current generation following knockdown with siRNA. As a result, we found that knockdown of Candidate17 mostly abolished the amplitude of MA current in Cell #6, but not in Cell #1. Next, we assessed the mechanosensitivity in dissociated DRG neurons using Candidate 17 KO mice. We found that MA currents of fast and slow types were significantly decreased in Candidate 17 KO mice compared to WT. We are currently investigating the function of Candidate 17 in DRG neurons in detail. These results suggest that Candidate17 may function as a modulator of unknown mechanotransducer(s), or Candidate17 and unknown components form a novel mechanically gated ion channel in mouse somatosensory neurons.

### [1P-038]
### A comparative analysis of the electrophysiological characteristics in bipolar cells across vertebrate species using a mathematical model

*Yuttamol Muangkram[1], Yukiko Himeno[1], Akira Amano[1] ([1]*Dept. of Bioinformatics, Coll. of Life Sciences, Ritsumeikan Univ.*)

Bipolar cells are specialized neurons located in the retinas of animals, including both lower and higher vertebrates. They play a crucial role in transmitting visual information from photoreceptor cells to ganglion cells. In lower vertebrates, various ionic currents, such as $I_h$, $I_{Kv}$, $I_A$, $I_{Ca}$, and $I_{KCa}$, have been identified, while their presence in higher vertebrates remains unclear. Although mathematical models have been developed to understand the electrophysiological properties of these currents, specific isoform information essential for characterizing their unique electrophysiological characteristics remains incomplete and requires further investigation. In this study, we conducted an extensive review of a gene expression database related to ionic currents in bipolar cells. Additionally, we analyzed available electrophysiological recordings from a range of vertebrate species, with a particular focus on the major genes responsible for encoding ion channels, including *Hcn1*, *Kcnb1*, *Kcnma1*, *Cacna1f*, *Cacna1g*, *Atp1a3*, *Atp2b1*, *Slc12a2*, and *Slc12a5*. Our simulation results revealed minor distinctions between lower and higher vertebrates in terms of these ionic currents. This research contributes to our overall understanding of how bipolar cells function in transmitting signals and maintaining normal physiological processes, which is vital in the context of preventing retinal diseases. This work was supported by JSPS KAKENHI Grant Number 22K20514.

### [1P-039]
### Degraded form vision and maintained brightness vision quantified in retinitis pigmentosa model rat

*Naofumi Suematsu[1,2], Akinori Sato[1,3], Akihiro Kimura[1,4], Satoshi Shimegi[1,5], Shogo Soma[1,6] ([1]*Graduate School of Medicine, Osaka University,* [2]*Department of Bioengineering, University of Pittsburgh,* [3]*Graduate School of Pharmaceutical Sciences, Nagoya University,* [4]*Department of Healthcare, Osaka Health Science University,* [5]*Center for Education in Liberal Arts and Sciences, Osaka University,* [6]*Department of Molecular Cell Physiology, Kyoto Prefectural University of Medicine*)

The retinitis pigmentosa is one of the biggest causes of blindness, which genetically induces impairments in retinal epithelium and photoreceptors. Understanding how visual responses and visual ability decreased during the disease progress is important to estimate disease staging in a patient, establish a therapeutic plan in advance, and evaluate the effects of interventional treatments. In the current study, we used the Royal College of Surgeons rat, an animal model with inherited retinitis pigmentosa, and evaluated form visual acuity and brightness visual detectability with behavioral tests as well as electrophysiological recordings in the dorsal lateral geniculate nucleus, superior colliculus, and primary visual cortex. The perceptual form vision tested by the two-alternative forced-choice task with drifting grating presentation, in which rats had to detect the stimulus to obtain a reward water drop, was maintained until 6 weeks old but attenuated during 7–8 weeks old, then completely lost after 9 weeks old. The neuronal responses in the three early visual pathways to flashing grating stimuli with various contrasts and spatial frequencies also showed a similar degeneration progress as the behavioral evaluations. On the other hand, the perceptual brightness vision, tested by the three-alternative forced-choice task where rats had to locate a uniform light source randomly turned on, was maintained until at least 11 weeks old. Along with this result, the neural responses in the three early visual pathways to the uniform flashlight stimulus were also maintained in the same period. Our findings suggest that the form vision was primarily affected by the disease progress of the retinitis pigmentosa, while the brightness vision has a potential robustness to the retinal degeneration. This situation may be due to the non-selective retinal degeneration rather than the selective dysfunction of a functionally distinct visual pathway/system. These results will provide useful and fundamental knowledge for evaluating the protective or restoration effects of the experimental treatments for retinitis pigmentosa.

### [1P-040]

### Reconstruction of Transretinal Extracellular Current by using Mathematical Model of Rod 1D Cable

*Shaocong Ou[1], Kouta Hori[1], Yuttamol Muangkram[1], Yukiko Himeno[1], Akira Amano[1]
([1]*Graduate School of Life Sciences, Ritsumeikan University*)

Electroretinogram (ERG) records transretinal voltage as a measure of the electrophysiological activity of the retina. It is used in the diagnosis of retinal diseases. However, the detailed mechanism of generation of ERG waveforms is not fully understood. For example, the origin of the "nose"-like waveform produced on ERG by stimulation of rod photoreceptors remains unclear. In this paper, we propose a 1D bi-domain cable model of rod photoreceptor cell that enables examination of the relationship between the photoreceptor components of ERG waveforms and the ionic currents of the rod membrane by calculating extracellular current and potential distribution under various conditions. However, since the information on the distribution of each individual membrane ionic current is quite limited, proposed model is based on the hypothetical current distribution partially supported by some reports of the immunostaining data. Our analysis of the relationship between the resulting individual membrane ionic current and the extracellular potential showed that the ERG "nose" is predominantly related to the $I_h$ current. We then created six rod models in which the cell body was located at different positions along the axon. Results showed that when a cell body is located close to the synaptic terminal, the cell has a larger ERG amplitude than cells with their body located close to the outer segment. However, there was little difference between the ERG waveforms of these cells.

### [1P-041]

### The role of α₂δ-1 subunit expressed in excitatory neurons in the spinal dorsal horn in mechanical hypersensitivity.

*Keisuke Koga[1], Kenta Kobayashi[2], Makoto Tsuda[3], Kazufumi Kubota[4], Yutaka Kitano[4], Hidemasa Furue[1] ([1]*Hyogo Medical University,* [2]*National Institute for Physiological Sciences,* [3]*Kyushu University,* [4]*Daiichi-Sankyo Co., Ltd.*)

Neuropathic pain is an intractable pain symptom that occurs after nerve damage and is caused by aberrant excitability of spinal dorsal horn (SDH) neurons. The current therapeutic drugs, gabapentinoids, reduce spinal neurotransmitter releases, and alleviate neuropathic pain by binding to $\alpha_2\delta$-1 subunits. Although $\alpha_2\delta$-1 is expressed both in the primary afferent and SDH neurons, the contribution of $\alpha_2\delta$-1 in SDH neurons to neuropathic pain conditions after nerve injury is not fully understood. In this study, we investigated whether $\alpha_2\delta$-1 in SDH neurons is involved in mechanical hypersensitivity and aberrant synaptic transmission after peripheral nerve injury. Using in situ hybridization technique, we found that *Cacna2d1*, mRNA coding $\alpha_2\delta$-1, was mainly expressed with *Slc17a6*, an excitatory neuronal marker, but not with *Slc32a1*, an inhibitory neuronal marker in the SDH. Using clustered regularly interspaced short palindromic repeats (CRISPR)-Cas9 system, we showed that SDH neuron-specific ablation of *Cacna2d1* alleviated mechanical hypersensitivity following nerve injury. We further found that excitatory post-synaptic responses evoked by electrical stimulation applied to SDH were significantly enhanced both in the nerve injured mice and in the presence of inhibitory neurotransmitter antagonists. These enhanced responses were significantly suppressed by the SDH neuron-specific ablation of *Cacna2d1*. Furthermore, the facilitation of A-fiber synaptic responses induced by disinhibition was also suppressed by the manipulation. These results suggest that $\alpha_2\delta$-1 expressed in SDH excitatory neurons enhances spinal nociceptive synaptic transmission and contributes to the development of peripheral nerve injury-induced mechanical hypersensitivity.

### [1P-042]

### Eye movement as a biomarker for early diagnosis of dementia

*Yuko Sugita[1], Yoshihiro Kokubo[2], Gao Qi[2], Takahisa Furukawa[1] ([1]*Osaka University,* [2]*National Cerebral and Cardiovascular Center Hospital*)

Dementia is a psychotic disorder that impairs self- and social-functioning and worsens over time. It affects an individual's memory, thought process, and ability to perform daily activities. Though dementia mainly occurs in older people, not everyone gets affected as they age. This condition is caused by diseases that destroy nerve cells and cause brain damage over time, typically reducing cognitive function. The lack of awareness and understanding of dementia hinders the diagnosis and management of the disease and also propagates stigma. As dementia pathology seems to start long before the symptoms appear, early diagnosis is of great clinical importance. Although various genetic, physiological, and neuroimaging studies focusing on noting the changes in patients with dementia have been conducted, no objective diagnostic biomarker in the clinical setting has been established to date. Previous studies on eye movement abnormalities in dementia have demonstrated a significant decline in saccade control and smooth pursuit eye movement. Thus, multiple eye movement measures may potentially increase diagnostic accuracy between a patient with dementia and a healthy control.This study aimed to examine eye movement measures as diagonistic biomarker that best characterize dementia and also distinguish patients with dementia from healthy controls. Local residents have poor knowledge about the relationship between cognitive decline and eye movement abnormalities. Participants aged > 65 years were enrolled from Suita City, Osaka Prefecture, through local advertisements, and their eye movements were evaluated through free viewing as well as saccade and smooth pursuit tests. We compared findings with those of younger participants and found that older adults had a longer onset time for saccadic eye movements than younger ones (from 20s to 50s). Our upcoming studies on dementia will evaluate the utility of other sensory functions (e.g., auditory and olfactory) and the results of the Mini-Mental State test, which has already been established as a reliable dementia test. These studies would help obtain sufficient understanding of visual and auditory functions in older adults, which would help identify visual and auditory abnormalities that can be used as diagnostic biomarkers for dementia.

### [1P-043]

### Hindbrain neurons that drive drinking in response to oral water

*Yu Yamada[1], Kengo Nomura[1], Shogo Soma[1], Naofumi Suematsu[2], Akiyuki Taruno[1]
([1]*Kyoto Prefectural University of Medicine,* [2]*University of Pittsburgh*)

Water consumption is crucial for maintaining fluid balance in terrestrial animals; thus, water tastes pleasant when one is thirsty. Over the past decade, detailed neural mechanisms have been uncovered that control the motivation to drink water based on internal signals indicating fluid imbalance, such as plasma osmolarity. However, the neural substrates that facilitate drinking behavior in response to external signals, like the sensation of water or liquid in the oral cavity or pharynx, have yet to be explored. Here we demonstrate that a subset of hindbrain neurons responds to water and might play a key role in water drinking behavior.
First, we conducted a behavior test to compare preferences between water and silicone oil, a water-free liquid. The number of licks for water was greater than that of silicone oil, even when the viscosity was adjusted to be equivalent, indicating that mice can discriminate between water and water-free liquid, and prefer water. Subsequently, we hypothesized the existence of brain neurons that selectively respond to oral water and facilitate drinking behavior, which can be referred to as "water taste neurons". To find candidate brain regions, we searched for activated brain areas in response to water intake after water deprivation across the entire brain using c-Fos immunoreactivity. We focused on a subregion of the parabrachial nucleus (PBN), because the region was activated in response to water intake but not water deprivation per se, and it receives various sensory inputs from gustatory, vagal, and somatosensory afferents. To further clarify the response characteristics of the PBN neurons during water ingestion, we performed *in vivo* $Ca^{2+}$ imaging experiments on head-fixed, awake behaving mice. Notably, a subset of the PBN neurons were activated immediately after the onset of water-licking but not silicone oil, implying that the neurons receive water-related signals other than somatosensory signals from oral cavity or pharynx. Furthermore, acute optogenetic inhibition of the PBN decreased the lick number of water without increasing access latency to water, suggesting that the PBN neurons control water drinking behavior but not the motivated behavior to access water. These results suggested that putative water taste neurons in the PBN may play a crucial role in water drinking behavior driven by chemosensory information of water.

### Poster

[1P]
### Neurophysiology, Neuronal cell biology
**Others**

March 28, 13:00 - 14:20, Poster Room

### [1P-044]
### Effects of menthol on nociceptive behaviors in the oral region of rats.

*Fukuzaki Mari[1,2], Nakatomi Chihiro[2], Hsu Chia-Chien[2], Kubo Sayaka[3], Kawamoto Tatsuo[1], Ono Kentaro[2] ([1]*Division of Orofacial Functions and Orthodontics, Department of Health Promotion, Kyushu Dental University,* [2]*Division of Physiology, Department of Health promotion, Kyushu Dental University,* [3]*Daiichi Sankyo Healthcare Co., Ltd.*)

Menthol is a typical agonist of TRPM 8 and has been suggested to mediate both analgesia and nociception, but its mechanism of action is unknown. To investigate the role of TRPM8 in pain in the oral region, we examined effects of topical application of menthol on nociceptive behaviors in rats. Wild-type male Wistar rats (300-500 g) were used for experiments. Menthol (1 M,100 mM,10 mM), AITC, an agonist of TRPA1 (100 mM), and capsaicin, an agonist of TRPV1 (100 µM) were used as stimulants, and 1% DMSO was used as a control. A drop of the stimulants was applied on the labial fornix region of the lower incisors of the rats. Immediately after the dropping, mouth rubbing by both fore-limbs was observed for 5 min as a nociceptive behavior. To investigate the analgesic effects of menthol on TRPA1- or TRPV1- mediated nociception, mixtures of menthol and capsaicin or AITC were applied. The TRPM8 antagonist AMG-333 was administered orally to clarify the involvement of TRPM8 in the effects of menthol. Since TRPA1 is activated by menthol, we performed the same experiments in TRPA1 knockout male rats. The application of high concentrations of menthol (1M) prolonged the rubbing time compared to the control groups, suggesting that 1 M menthol induce nociceptive behavior. Since the prolonged rubbing time was not observed in the TRPA1 knockout rats and was inhibited by AMG-333, the nociceptive behavior was caused by the activation of TRPM8. In contrast to the high concentration of menthol, low concentrations of menthol (10 and 100 mM) did not increase the rubbing time and inhibited capsaicin-induced prolongation of rubbing time. The inhibition was not observed following administration of AMG-333, suggesting that the analgesic effect for capsaicin-induced nociception was caused by the activation of TRPM8. The analgesic effect of menthol was not observed in AITC-induced nociception. Our results suggest that activation of TRPM8 by high and low concentrations of menthol induces nociception and analgesic effects, respectively.
Conflicts of interest: This work was funded by Daiichi Sankyo Healthcare Co., Ltd. C. Nakatomi received research grant support from Daiichi Sankyo Healthcare Co., Ltd.

### [1P-045]
### Investigation of heartbeat-evoked potentials pre- and post-perception of milk ejection in breastfeeding mother

*Nami Ohmura[1,2], Lana Okuma[2], Naomi Takamiya[1], Takafumi Sasaoka[1], Kumi O. Kuroda[3,2], Shigeto Yamawaki[1] ([1]*Hiroshima University,* [2]*RIKEN,* [3]*Tokyo Institute of Technology*)

Breastfeeding is the most fundamental maternal behavior in mammals. It is important not only for infant nutritional and immunological aspects but also for the attachment formation between mother and infant. In human mothers, breastfeeding relieves psychological stress and anxiety, and facilitates positive affect. We reported that mothers exhibit parasympathetic dominant states during breastfeeding by investigation of maternal electrocardiogram (ECG) (Ohmura et al., *J Physiol Sci.*, 2023). Mothers often perceive milk ejection during breastfeeding, and 76.9 % of mothers perceived milk ejection in the study. The sensation of milk ejection is considered one of the internal body sensations, termed interoception. The heartbeat-evoked potential (HEP), the event-related potential of the electroencephalography (EEG) following the R peak of the ECG, is used as an electrophysiological marker of interoception. We recorded ECG and EEG of breastfeeding mothers and compared HEPs during the periods before and after the perception of milk ejection, i.e., pre-conscious and post-conscious states of interoception. The amplitude of HEPs tended to differ before and after the perceived milk ejection. These results suggest that HEPs may represent differences in brain processing of interoception. Since alteration in interoception is thought to be associated with affect this study may contribute to understanding the mechanisms of maternal mood/emotional changes associated with milk ejection in the future.

### [1P-046]
### Eyeblink conditioning establishment by limited pairing of sensory stimuli

*Ryota Iwase[1], Shin-ya Kawaguchi[1] ([1]*Graduate School of Science, Kyoto University*)

Eyeblink conditioning has been one of the major models of cerebellar-dependent motor learning in mice. Typically, high ratio pairing of conditioned stimulus (CS, tone) and unconditioned stimulus (US, air puff) has been used for the training. Interestingly, the effects of decreasing the ratio of pairing to 50 % have been well studied and animals exhibit conditioned response (CR) as much as with the high ratio pairing training. However, it remains elusive what is the minimal requirement of paired stimuli for the successful acquisition of CRs. To address this issue, here we reduced the ratio of CS-US paired trials to 10 % and quantitatively analysed the percentage of CR occurrence and the dynamics of responses, such as, onset latency, peak latency, amplitude, velocity of eyelid closure. Mice were head-fixed on a treadmill, allowing them to walk freely, and given 100 trials of daily training for 10 consecutive days consisting of 90 % CS-US pairing (high-ratio) or 10 % pairing (less-paired). The interstimulus interval was set to 250 ms and the intertrial interval was set to 10-25 sec randomly for each trial. Eyelid movements were monitored by a high-speed camera. Surprisingly, the less-paired group efficiently acquired CRs even with ~11 % reinforcement experience. Notably, CRs established in the less-paired group seemed not so sophisticated as those in the high-ratio pairing group in terms of the movement control in timing. In addition, the velocity of eyelid closure was different from each other. We also examined which part of the brain mediated the CR expression using pharmacological inactivation technique. We specifically applied muscimol, GABAA receptor agonist, through an implanted cannula to the cerebellar nuclei region of CR expressing mice and observed the effects of inactivation of neurons there.Here we like to demonstrate how the classical reinforcement learning is robust to the change of experience intensity, that is, stimulus pairing ratio and to highlight the distinct aspects of learned behaviors established in different paradigms. We also aim to show important brain regions for the CR expression.

### [1P-047]

### Withdrawn

### [1P-048]

### Effects of uncertainty and surprise in chord sequence on interoceptive processing: a heartbeat evoked potentials (HEPs) study

*Naomi Takamiya[1], Tatsuya Daikoku[1,2], Takafumi Sasaoka[1], Maro G Machizawa[1], Ono Kentaro[1], Toru Maekawa[1], Nami Ohmura[1], Alan S R Fermin[1], Shigeto Yamawaki[1]
([1]*Hiroshima University,* [2]*The University of Tokyo*)

*[INTRODUCTION]* Emotional states induced by music listening are influenced by uncertainty and surprise in chord sequence prediction (Cheung et al., 2019). A recent emotion theory has suggested that emotions emerge via predictive processes and interoceptive inference over ascending visceral and physiological signals to the brain (Seth, 2013). This led us to hypothesize that uncertainty and surprise in chord sequence can affect our interoceptive systems and subsequent emotional states. To test this hypothesis, we quantified uncertainty and surprise using a statistical model of music and examined how music parameters influence music-evoked emotions by heartbeat evoked potentials (HEPs), a neural marker of preconscious cardiac interoception.
*[METHODS]* Participants: Twenty-five healthy adults. Stimuli: We computed the surprise and uncertainty using a statistical model that learned every chord in the McGill Billboard corpus. Using this model, chord sequences of 4 chords were generated and categorized into 4 types: low uncertainty + small surprise, low uncertainty + large surprise, high uncertainty + small surprise, and high uncertainty + large surprise. Measurement procedure: The 64-ch electroencephalogram (EEG) and electrocardiogram (ECG) were measured while participants listened to stimuli. Each chord was presented every 2 seconds. Participants were instructed to predict the 4th chord. Following the stimuli presentation, participants were asked to indicate whether their prediction was accurate/inaccurate and to rate the degree of pleasantness/unpleasantness. Each category was recorded in 100 trials. EEG data were compared across categories for HEPs time-locked to the R-wave peak in the ECG detected after each stimulus chord.
*[RESULTS]* Ratings of pleasantness/unpleasantness were modulated by objective uncertainty and surprise predicted by the statistical model. However, when stimuli were categorized based on participants' self-reported prediction accuracy, specifically their subjective surprise, rather than objective surprise, we observed that stimuli with high subjective surprise tended to evoke more unpleasant feelings. Furthermore, HEPs also showed distinct patterns depending on emotional state and stimulus category.
*[CONCLUSION]* As hypothesized, these results suggest the possibility that uncertainty and surprise of chord sequence may induce emotions and modulate interoceptive processing. Our future research will investigate how individual characteristics, such as individual differences in depressive tendencies and interoceptive awareness, are linked with HEP and its modulation by uncertainty and surprise.

### [1P-049]

### Functional cortical network changes in schizophrenia model mouse

*Takamasa Yoshida[1,2], Satoshi Kuroki[3,2], Chie Sano[2,4], Reiko Ando[2,5], Toshiaki Nakashiba[2,6], Shigeyoshi Itohara[2], Naoki Tanaka[7,8], Toshihiro Hayashi[1] ([1]*Dept. of Physiology, Teikyo Univ. School of Medicine,* [2]*Lab. for Behavioral Genetics, RIKEN CBS,* [3]*Dept. of Physiology, Osaka Metropolitan Univ. Graduate School of Medicine,* [4]*Support Unit for Animal Resources, RRD, RIKEN CBS,* [5]*Support Unit for Bio-Material Analysis, RRD, RIKEN CBS,* [6]*Experimental Animal Division, RIKEN BRC,* [7]*Dept. of Biomedical Engineering, Faculty of Science and Engineering, Toyo Univ.,* [8]*Research Institute of Industrial Technology, Toyo Univ.*)

Imaging studies, particularly fMRI studies, of schizophrenia patients and its mouse models have revealed abnormalities in functional connectivity across all brain regions. In schizophrenia, multisensory integration is thought to be impaired by disruption of functional networks among brain regions, but the mechanism remains poorly understood. In our previous study, using wide-field calcium imaging, we found that multisensory input induced phase locking of slow-wave oscillations in the cortical association cortex of normal mice. If the functional cortical network is affected in a mouse model of schizophrenia, the phase locking during multisensory input may also be altered. In this study, we first examined the functional connectivity in the slow-wave frequency band of spontaneous activity across the dorsal cerebral cortex of poly (I:C)-induced schizophrenia model (SZ model) mice using wide-field calcium imaging to determine whether the functional cortical network is impaired in SZ model mice. The results showed that in the SZ model mice, the homotopic correlation between the left and right hemispheres was attenuated only in the visual cortex, whereas functional connectivity between other regions remained unchanged between control and SZ model mice. We next analyzed the functional cortical network of the SZ model mouse using Granger causality, which allows us to infer information flow and causal relationships between brain regions. As a result, the number of significant causal links increased, and the network structure became more complex in the SZ model mice compared to the control mice. These results indicate that the network structure based on correlation analysis is different from that based on causality analysis, suggesting that causal analysis is valuable for analyzing complex brain networks. In the next step, we will examine whether phase locking to multisensory inputs is transformative in the SZ model mice.

### [1P-050]

### Multimodal Brain Activity Measurements during Focal Brain Cooling: A Study of Regional Cerebral Hemodynamics in Epileptic Focus

*Sayuki Takara[1], Takao Inoue[1], Sadahiro Nomura[2], Toshitaka Tamakawa[3], Hirochika Imoto[2], Yuichi Maruta[2], Masatsugu Niwayama[4], Michiyasu Suzuki[2] ([1]*Organization of Research Initiatives, Yamaguchi University,* [2]*Department of Neurosurgery, Yamaguchi University School of Medicine,* [3]*Faculty of Advanced Science and Technology, Kumamoto University,* [4]*Graduate School of Medical Photonics, Shizuoka University*)

Introduction:
Epilepsy is a chronic neurological disorder characterized by abnormal brain activity, which is detectable through abnormal electrocorticography (ECoG). It is known that cooling the brain can suppress this abnormal brain activity at the epileptic focus. Therefore, we aimed to evaluate the impact of brain cooling on the epileptic focus by accurately measuring brain temperature and assessing the degree of ECoG suppression. We established a multi-modality measurement probe to simultaneously collect data from ECoG, brain temperature, and cerebral hemodynamics as a multifaceted evaluation, investigating their combined impact on brain activity.
Methods:
We conducted this study with 13 patients suffering from intractable epilepsy (12 with temporal lobe epilepsy and 1 with occipital lobe epilepsy). Some patients reported discomfort due to stimulation that intensified abnormal brain waves, prompting the use of anesthesia with 2.5% sevoflurane. Multi-modality probes were strategically placed in the epileptogenic cortical region determined by pre-surgical evaluations for the planned resection area. A portion of the probe was covered with a 30 mm square-shaped cooling device (a water-circulated titanium device or a Peltier device) to cool the brain surface. The measurement duration was 30 minutes, with the central 10 minutes designated as the cooling period.
Results:
As brain temperature decreased, ECoG amplitudes decreased, while the concentrations of oxygenated and deoxygenated hemoglobin concentration displayed a non-linear response. Oxyhemoglobin concentrations decreased within the temperature range of 18 to 27°C, while deoxyhemoglobin concentrations increased. However, no consistent trend was observed in total hemoglobin concentration, which correlates cerebral blood flow.
Conclusion:
The combined measurement of ECoG and cerebral hemodynamics suggests an effective approach for evaluating the cooling effect on epileptic focus.

### Poster

### [1P]
### Molecular physiology, Cell physiology
### Membrane transport

March 28, 13:00 - 14:20, Poster Room

### [1P-051]

### Facilitative action of glucose on insulin granule behavior in mouse pancreatic islet cells

Tomomi Oshima[1], *Hiroyasu Hatakeyama[1], Noriko Takahashi[1] ([1]*Department of Physiology, Kitasato University School of Medicine*)

Glucose-stimulated insulin secretion in pancreatic β-cells is the primary mechanism for glucose homeostasis and its dysregulation can result in diabetes. One of the critical processes in insulin secretion is the recruitment of intracellular insulin granules to the juxtamembrane regions prior to exocytosis, but its regulatory mechanisms remain uncertain. Here, we performed live-imaging analysis of insulin granule behavior in mouse pancreatic islet cell clusters expressing insulin-HaloTag via adenoviral infection. In the cells stained with the HaloTag TMR ligand, we visualized granular structures thought to be insulin granules and tracked the movement of these granular structures using spinning disk confocal microscopy to analyze their behavior. Acute stimulation of the cells with high glucose markedly facilitated insulin granule behavior within several minutes of glucose stimulation. In contrast, cells treated with 2-deoxyglucose, a non-metabolizable glucose analog, or exposed to artificially elevated cytosolic $Ca^{2+}$ concentrations via uncaging of caged-$Ca^{2+}$ compounds did not exhibit such facilitation of the behavior. These observations suggest that metabolites generated during glucose metabolism play an important role in the facilitation of insulin granule behavior. Previously, we had established a method for high-precision measurements of insulin granule behavior in rat INS-1 insulinoma cells using Quantum dot fluorescent nanocrystals, and we have successfully introduced the nanocrystals into pancreatic islet cell clusters, which is expected to be extended to detailed quantification of insulin granule behavior in pancreatic islet cells. Overall, our live-imaging analysis of intracellular insulin granule behavior will provide new insights into its regulatory mechanisms and etiology of diabetes.

### [1P-052]

### Effects of sertraline on $Mg^{2+}$ extrusion in rat ventricular myocytes

*Michiko Tashiro[1], Masato Konishi[2], Utako Yokoyama[1] ([1]*Dept. Physiology, Tokyo Medical Univ.*, [2]*Tokyo Medical Univ.*)

Intracellular free $Mg^{2+}$ concentration ($[Mg^{2+}]_i$) is tightly regulated because $Mg^{2+}$ affects $Ca^{2+}$ signaling involved in cardiac contraction. $Na^+$-dependent $Mg^{2+}$ extrusion ($Na^+/Mg^{2+}$ exchange) is recognized to play an important role in intracellular $Mg^{2+}$ homeostasis. However, the molecular mechanism of the $Mg^{2+}$ extrusion is still unclear. We previously reported that the activity of $Na^+/Mg^{2+}$ exchange was inhibited by KB-R7943, an inhibitor of $Na^+/Ca^{2+}$ exchanger, with half inhibitory concentrations ($IC_{50}$) of 21 μM in the $Ca^{2+}$-free Tyrode's solution at 25°C. In this study, we found that sertraline, a selective serotonin reuptake inhibitor, inhibited $Na^+$-dependent $Mg^{2+}$ extrusion more potently than KB-R7943.We measured $[Mg^{2+}]_i$ from a single ventricular myocyte acutely isolated from rats using a fluorescent indicator, mag-fura-2. After background measurements, the cells were soaked in a low-$Na^+$ and high-$Mg^{2+}$ solution to load $Mg^{2+}$. $[Mg^{2+}]_i$ was increased from ~ 0.9 to ~1.5 mM for 2 h. The addition of extracellular $Na^+$ caused a decrease in $[Mg^{2+}]_i$. We analyzed the rate of decrease in $[Mg^{2+}]_i$ as $Na^+/Mg^{2+}$ exchange transport activity. Since the rate depends on $[Mg^{2+}]_i$ just before $Na^+$ addition, the rates were corrected for relative values based on previously determined correlations. The inhibition of $Na^+/Mg^{2+}$ exchange transport by sertraline was concentration-dependent ($IC_{50}$ 8.6 μM) and reversible. We also observed whether sertraline inhibited $Mg^{2+}$ influx. To deplete $Mg^{2+}$, the cells were soaked in a high-$K^+$ and $Mg^{2+}$-free solution. $[Mg^{2+}]_i$ was decreased from ~ 0.9 to ~ 0.5 mM for 30 min by $Mg^{2+}$ depletion. Perfusing the $Ca^{2+}$-free Tyrode's solution (containing 1 mM $Mg^{2+}$) recovered $[Mg^{2+}]_i$. The time course of the $[Mg^{2+}]_i$ recovery was fitted by a single exponential function, and the first derivative at time 0 was analyzed as the $Mg^{2+}$ influx rate. The $Mg^{2+}$ influx rate in the presence of sertraline was 0.26 ± 0.1 μM/sec (mean ± SD), which was not significantly different from that obtained without sertraline (0.27 ± 0.2 μM/sec). In conclusion, sertraline showed the highest potency of inhibition of $Na^+/Mg^{2+}$ exchange transport among the inhibitors reported to date, with little influence on the $Mg^{2+}$ influx. It suggests sertraline could be used as a tool to identify molecules involved in the physiological $Mg^{2+}$ extrusion system.

### [1P-053]

### Measurement of water flux across lipid bilayer under controlled bilayer tension using water-in-oil microdroplets and its application to AQP study.

Misuzu Ueki[1], Takahisa Maki[1], *Masayuki Iwamoto[1] ([1]*University of Fukui*)

The primary pathways for water permeation across cell membranes involve the lipid bilayer and aquaporin (AQP) water channels. Understanding the mechanisms that regulate water flow through these pathways is essential for comprehending various cellular processes and physiological phenomena driven by water permeation. While extensive research has shed light on the properties of the lipid bilayer and AQP function individually, the interaction between these pathways, particularly how lipid bilayer characteristics like composition and tension influence AQP activity, remains only partially understood. This knowledge gap is due to the inherent difficulties in quantifying water flux through AQPs in a controlled lipid bilayer environment. This study presents an experimental approach for measuring water flux while manipulating lipid bilayer properties using a contact bilayer formed between two water-in-oil microdroplets. Notably, our method enables precise control of bilayer tension through pressure adjustments following the Young-Laplace principle. To validate the reliability of our method, we initially assessed the water permeability of the contact bilayer in the absence of AQPs. We induced changes in the volume of each microdroplet by creating an osmotic gradient between the two droplets, causing water to move across the contact bilayer. We measured the bilayer area and the rate of volume change for the droplets from microscope images, which allowed us to analyze the water flux. We successfully manipulated bilayer tension within a range of approximately 1-8 mN/m, a tension range that is relevant to many mechanosensitive channels. Subsequently, we reconstituted AQPZ into the contact bilayer and attempted to measure water flux. We discuss the applicability of our experimental approach and the outstanding challenges in fully understanding the interplay between the lipid bilayer and AQPs.

### [1P-054]

### Mathematical model of intracellular pH changes induced by $NH_4^+$ pulse in pancreatic duct cell

*Makoto Yamaguchi[1], Yoshiro Sohma[2], Akiko Yamamoto[1], Yuka Kozawa[1], Itsuka Taniguchi[1], Nao Nomura[1], Mayuko Higuchi[1], Hiroshi Ishiguro[1] ([1]*Department of Human Nutrition, Nagoya University Graduate School of Medicine,* [2]*Department of Pharmaceutical Sciences and Center for Medical Sciences, International University of Health and Welfare*)

The $NH_4^+$ pulse technique has been used to evaluate the activity of membrane $H^+$/$HCO_3^-$ transport in various cell types. Addition of $NH_4^+$ to the bath causes rapid cellular alkalinization followed by partial recovery and subsequent removal of $NH_4^+$ causes rapid acidification followed by recovery to the baseline. Those changes are thought to be largely due to membrane diffusion of $NH_3$, consumption of $H^+$ by intracellular buffering, and membrane $H^+$/$HCO_3^-$ transport. In the present study, we have tried to simulate changes in intracellular pH ($pH_i$) induced by $NH_4^+$ pulse using a mathematical model of pancreatic duct cell.

Our previous model of pancreatic duct cell was constructed using MATLAB/Simulink (MathWorks) (Yamaguchi, J Physiol 2017). Various ion channels/transporters, water and gas ($CO_2$, $NH_3$) permeabilities are localized in basolateral and apical membranes and intracellular $H^+$ buffering systems are included: $HCO_3^-$-$CO_2$, intrinsic (non-$CO_2$), and $NH_3$-$NH_4^+$ buffering. Intrinsic buffering is modelled as the equilibrium between a single weak base $B^-$ and its conjugate weak acid HB ($HB \Leftrightarrow H^+ + B^-$). The model is capable of producing $HCO_3^-$-rich (~140 mM) fluid secretion at a rate of ~3 nl $min^{-1}$ $mm^{-2}$ (luminal area of epithelium).

When the bath was superfused with the standard $HCO_3^-$-$CO_2$-buffered solution and 20 mM $NH_4Cl$ was added to the bath, $pH_i$ of the model cell was rapidly elevated from baseline (~7.4) to ~8.0 and then slowly declined to ~7.7 in the presence of $NH_4Cl$. When $NH_4Cl$ was removed from the bath, $pH_i$ rapidly fell to ~6.9 and then slowly recovered to the baseline. Then, we have analyzed the effects of varying the values of intracellular activity of carbonic anhydrase (CA: catalyze the reaction of $H_2O + CO_2 \Leftrightarrow H_2CO_3$), membrane permeability of $CO_2$ and $NH_3$, $NH_4^+$ selectivity of basolateral $K^+$ channel, and intrinsic $H^+$ buffer ([HB] + [$B^-$]) on the magnitude and shape of $pH_i$ changes. While all parameters had significant contribution to the shape of $pH_i$ changes by $NH_4^+$ pulse, the intracellular CA activity had largest contribution to the magnitude of $pH_i$ changes by $NH_4^+$ pulse. When CA activity was set to 3-fold of non-catalyzed reaction rate, the magnitude of $pH_i$ changes best fitted to the experimental data (Hegyi, Am J Physiol Cell Physiol 2003).

In summary, we have successfully constructed a mathematical model of pancreatic duct cell which simulates $pH_i$ changes by $NH_4^+$ pulse.

### [1P-055]

### Intracellular C-terminal domain of mGluR6 is involved in regulating receptor subcellar localization

*Ikuo Ogiwara[1], Atsushi Shimohata[1], Takumi Akagi[1], Makoto Kaneda[1] ([1]*Department of Physiology, Nippon Medical School*)

Metabotropic glutamate receptor 6, mGluR6, localizes at the dendritic tips of retinal ON-bipolar cells, and plays critical roles in the retinal processing of visual signals. mGluR6 interacts with scaffold proteins via its intracellular C-terminal domain (CTD). We here examined the roles of mGluR6 CTD in mGluR6 subcellar distribution, using mGluR6 CTD mutants expressed in human embryonic kidney 293T cells with immunocytochemistry and flow cytometry. We showed that full-length mGluR6 was distributed in the endoplasmic reticulum (ER) and transported to the cell surface, whereas mGluR6 CTD mutants with 15- and 20-amino acid deletions from the C terminus localized to the ER, but were deficient at the cell surface. We also showed that the surface-deficient mGluR6 CTD mutant still did not localize to the cell surface and was retained in the ER when co-expressed with full-length mGluR6. Forming of heteromeric complexes between full-length and mutated mGluR6 rather led to a reduction in surface levels of full-length mGluR6. We finally demonstrated that the cell surface deficiency of mGluR6 mutants was rescued by introducing an alanine substitution in a cluster of di-basic residues, such as di-arginine (RXR) and di-lysine (KKXX) sequences, within the CTD, which resembled ER retention motifs. These observations suggest that mGluR6 intracellular trafficking is regulated via basic ER retention motifs in the CTD.

### [1P-056]

### Numerical analysis of RNA extraction by nano electroporation

*Emika Asechi[1], Kenji Nakashima[1], Fuminori Matsuyama[1], Mahmoud N. Abdelmoez[2], Taikopaul Kaneko[3], Misa Minegishi[3], Hirofumi Shintaku[3,4] ([1]*National Institute of Technology, Sasebo College,* [2]*Assiut University,* [3]*Institute of Physical and Chemical Research,* [4]*Kyoto University*)

### Poster

### [1P]
### Molecular physiology, Cell physiology
### Ion channels, Receptors

March 28, 13:00 - 14:20, Poster Room

### [1P-058]

### CALML6 is involved in the migration of oral cancer cells

*Soichiro Ishikawa[1,2], Masanari Umemura[1], Rina Nakakaji[1,2], Akane Nagasako[1], Kagemichi Nagao[1], Yuto Mizuno[1], Eriko Yamashita[1,2], Fumina Suzuki[1], Wakana Fukae[1], Kenji Mitsudo[2], Yoshihiro Ishikawa[1] (*[1]Cardiovascular Research Institute (CVRI), Yokohama City University Graduate School of Medicine, [2]Department of Oral and Maxillofacial Surgery, Yokohama City University Graduate School of Medicine*)

**Introduction**
Lymph node metastasis resulting from the migration of oral cancer cells is an important prognostic factor. We previously reported that EP4, a prostaglandin $E_2$ ($PGE_2$) receptor, regulates the cell migration in oral cancer cells through $Ca^{2+}$ signaling. However, how $Ca^{2+}$ signaling regulates cell migration remains unclear. Intracellular $Ca^{2+}$ regulates various signaling pathways, either directly or by interacting with proteins such as calmodulin (CaM). Furthermore, $Ca^{2+}$ signaling is directly or indirectly involved in cancer cell proliferation and migration. Thus, we hypothesize that EP4 regulates cell migration through calcium-binding proteins and their subsequent signaling in oral cancer.
**Materials and Methods**
Human gingival fibroblasts, HGnF and Human tongue squamous cell carcinoma cell lines, HSC-3 were used. EP4 agonist (ONO-AE1-437) was used. HSC-3 cells were transduced with calmodulin like 6 (CALML6) shRNA, and scramble control shRNA using lentivirus. We conducted RNA sequencing (RNA seq) to identify molecules associated with EP4 in HSC-3 cells. mRNA transcription levels were evaluated by quantitative real-time reverse transcription-polymerase chain reaction (qPCR). Protein expressions levels were evaluated by western blotting. Immunohistochemistry was performed to evaluate protein expression in tongue tumor tissues. The cell migration ability was evaluated using the scratch assay. Production of reactive oxygen species (ROS) was also evaluated by both DCFH-DA fluorescence and electron spin resonance (ESR).
**Results**
Gene ontology (GO) analysis showed that EP4 stimulation significantly enriched gene expression related to calcium binding proteins. Among the 62 genes identified, CALML6 expression was markedly increased. Interestingly, the mRNA and protein expression of CALML6 in HSC-3 cells were higher than those of HGnF cells (n=4, $p<0.05$, $p<0.01$). The protein expression level of CALML6 in tongue tumor tissue was higher than that in adjacent normal tongue tissue, as determined by immunohistochemistry. Furthermore, EP4 agonist promoted the cell migration and ROS production in HSC-3 cells, but did not promoted cell proliferation (n=4 or 6, ns; not significant, $p<0.001$). In contrast, knockdown of CALML6 suppressed the EP4-stimulated cell migration and ROS production (n=4 or 6, ns; not significant, $p<0.001$).Taken together, EP4 regulated cell migration through CALML6 in oral cancer.
**Conclusion**
The EP4/$Ca^{2+}$/CALML6 pathway represents a novel signal transduction mechanism in oral cancer and may be a new target for oral cancer therapy.

### [1P-057]

### A novel inhibitor of OTOP1, a proton ($H^+$) channel, was identified by screening of a chemical library

*Soichiro Yamaguchi[1], MD Mominul Islam[2], Omi Sasaki[3], Saori Yano-Nashimoto[1], Yuko Okamatsu-Ogura[4] (*[1]Laboratory of Physiology, Department of Basic Veterinary Sciences, Faculty of Veterinary Medicine, Hokkaido University, [2]Laboratory of Physiology, Department of Basic Veterinary Sciences, Graduate School of Veterinary Medicine, Hokkaido University, [3]Laboratory of Physiology, Department of Basic Veterinary Sciences, School of Veterinary Medicine, Hokkaido University, [4]Laboratory of Biochemistry, Department of Basic Veterinary Sciences, Faculty of Veterinary Medicine, Hokkaido University*)

Otopetrin1 (OTOP1) had been known as a gene product which was required for the formation of otoconia in the vestibular system. More recently, OTOP1 was revealed to be a proton ($H^+$) selective channel, which detects acidic stimuli in sour taste receptor cells. Although zinc ion ($Zn^{2+}$) can inhibit OTOP1 currents at mM order, no other potent inhibitors nor activators of OTOP1 were discovered. If such a channel modulator is identified, physiological roles of OTOP1 in the vestibule and other cells will be revealed more easily. Therefore, we screened the Library of Pharmacologically Active Compound 1280 (LOPAC1280, Sigma Aldrich) by whole-cell patch clamp measurements to identify a novel modulator of OTOP1 activity. From the screening, no activators were found, but reactive blue 2 was raised as a possible inhibitor of OTOP1. However, 100 µM reactive blue 2 inhibited only about 50% of OTOP1 currents. Therefore, to identify a more potent inhibitor, we evaluated three analogues of reactive blue 2. Among them, cibacron blue 3G-A inhibited OTOP1 currents the most potently. Cibacron blue 3G-A inhibited OTOP1 currents in a concentration-dependent manner. The data fit the Hill equation, where the inhibitory concentration ($IC_{50}$) and the Hill coefficient were 5.0 µM and 1.1, respectively. Furthermore, we evaluated and compared the inhibitory features of cibacron blue 3G-A and $Zn^{2+}$. We found that the inhibition of OTOP1 currents by $Zn^{2+}$ was attenuated in a chloride-deficient glutamate-rich solution and at depolarized membrane potentials, but the inhibition by cibacron blue 3G-A was not affected. The inhibition by $Zn^{2+}$ was also known to be weakened in the acidic solution, but the inhibition by cibacron blue 3G-A was not decreased. These inhibitory features suggest that the inhibition of OTOP1 by cibacron blue 3G-A is neither likely to be a pore-blocking inhibition nor a competitive inhibition. Furthermore, these inhibitory features may improve usability of cibacron blue 3G-A as an inhibitor of OTOP1 because it is less affected by the experimental conditions which are adopted when OTOP1 activity is evaluated (chloride-free condition, low pH, and membrane depolarization).

### [1P-059]

### Functional and structural basis of hERG facilitation by its blockers

*Kazuharu Furutani[1], Aiyana Cortez[2], Igor Vorobyov[2], Vladimir Yarov-Yarovoy[2], Satomi Kita[1] (*[1]Tokushima Bunri Univ., [2]Univ. California, Davis*)

Pharmacological modulation of the human Ether-à-go-go-Related Gene (hERG) channel, a voltage-gated potassium channel that plays a pivotal role in the repolarization of action potentials in ventricular myocytes in the heart, significantly affects cardiac electrophysiology and can be antiarrhythmic or proarrhythmic. Certain hERG channel blockers can facilitate hERG activation to increase hERG currents, which may reduce the proarrhythmic potential. However, the molecular mechanisms underlying the facilitation effect of hERG blockers remain unclear. Here, we demonstrate that 1) nifekalant accesses the receptor site within the pores of the open or inactivated channels at depolarized potentials, 2) upon return to the resting potentials, channels close and trap nifekalant inside, 3) trapped nifekalant biases the open-closed equilibrium towards the open state, and 4) the kinetics of drug escape from the channel are faster than channel closing rates at potentials where facilitation of hERG current is observed, thereby drug unbinding reveals channels that have been biased towards the open state. Simulations with a Markov model of such a nifekalant-hERG interaction successfully reproduced the key characteristics of hERG facilitation. We also presented a potential structural model for hERG channel facilitation through drug interactions with the hydrophobic pocket of the hERG pore domain. This pattern of interaction is consistent with experimental data, suggesting that facilitating drugs may act as a wedge to bias hERG channel equilibrium towards the open state and increase hERG current amplitude in response to low-voltage depolarization.

### [1P-060]

### The role of PKD2L1 cation channel in the bitter aftertaste perception of quinine

*Takahiro Shimizu[1], Takuto Fujii[1], Keisuke Hanita[1], Ryo Shinozaki[1], Yusaku Takamura[2], Yoshiro Suzuki[3], Teppei Kageyama[1], Mizuki Kato[1], Hisao Nishijo[2], Makoto Tominaga[3], Hideki Sakai[1] ([1]*University of Toyama, Faculty of Pharmaceutical Sciences,* [2]*University of Toyama, Faculty of Medicine,* [3]*National Institute for Physiological Sciences*)

Bitterness contributes to defense responses to avoid toxic foods. The bitterness is composed of two kinds of taste: a taste in the presence of bitterants and an aftertaste that persists after the removal of bitterants. However, the mechanism that produces the aftertaste is poorly known. Interestingly, the polycystic kidney disease 2-like 1 (PKD2L1) cation currents were increased after the removal of quinine, but not denatonium, in whole-cell recordings of PKD2L1-expressing HEK293T cells. Besides, $Ca^{2+}$ response was observed after the removal of quinine, but not denatonium, in $Ca^{2+}$ imaging of mouse Type III taste cells endogenously expressing PKD2L1. The quinine-induced $Ca^{2+}$ response disappeared in the PKD2L1-deficient Type III cells, suggesting that the PKD2L1 channel shows the off-response to quinine. In short-term two-bottle preference and lick tests, on the other hand, wild-type mice avoided normal water while the PKD2L1-deficient mice preferred normal water after being given quinine-containing water. Therefore, we propose that the PKD2L1 cation channel expressed in Type III taste cells is a novel sensor for the aftertaste of quinine, but not denatonium.

### [1P-061]

### The effects of hydrogen sulfide on the voltage-gated potassium channels

*Akira Kawanabe[1], Yuichiro Fujiwara[1,2] ([1]*Faculty of Medicine, Kagawa University,* [2]*School of Medicine, Hiroshima University*)

Kv7/KCNQ channels are a representative subfamily of voltage-gated potassium channels, including five members (Kv7.1-7.5 or KCNQ1-5), and play important physiological functions in neuronal and cardiac excitability. Particularly, Kv7.2/7.3 heteromultimer exhibits a slow activating voltage-dependent potassium current known as “M-current” that modulates the firing rate of action potentials in neuron. High concentration of Hydrogen sulfide ($H_2S$) is known as a natural hazardous gas. Since endogenous $H_2S$ has been identified in the brain and body in recent years, it has been proposed that low concentration of $H_2S$ acts as a signaling gas for physiological and pathological processes like other well-known gases, CO and NO (Wang 2002, Kimura 2021). In the nervous system, $H_2S$ may influence synaptic transmission via the regulation of ion channels, enzymes, and second messengers. It has been reported that $H_2S$ influences the activity of the voltage-gated potassium channels (Kv7.2/7.3) that regulate electrical activity in neurons to modulate neuropathic pain (Mannelli et al., 2017). However, the molecular mechanism of action of $H_2S$ in Kv7.2/7.3 channels is poorly understood. In this study, we examined the macroscopic currents of the Kv7.2/Kv7.3 channels using the whole-cell recording technique with $H_2S$. The application of NaHS (an $H_2S$ donor) to Kv7.2/7.3 expressed in CHO cells increased the amplitude of the outward potassium currents under the depolarizing step pulse, accompanied by the leftward shift of the voltage-dependence. The enhanced current decreased after treatment with the reducing agent, indicating that $H_2S$ induces the effect. In addition, we attempted to perform an analysis of $H_2S$ effects on other Kv7 subfamily members. Comparing the different effects among the Kv7 subfamily, we will discuss the molecular mechanisms and physiological significance of these regulations.
COI: No

### [1P-062]

### Arachidonic acid regulates the voltage sensor activation in the voltage-gated $H^+$ channels

*Maki Takata[1], Kohei Takeshita[2], Akira Kawanabe[1], Yuichiro Fujiwara[1,3] ([1]*Molecular Physiology & Biophysics, Faculty of Medicine, Kagawa University,* [2]*RIKEN SPring-8 Center,* [3]*Physiology and Biophysics, Graduate School of Biomedical and Health Sciences, Hiroshima University*)

Voltage-gated $H^+$ channels (Hv) are expressed on phagocytic cells, such as neutrophils and macrophages, and play an important role in the innate immune system. Their activity is regulated by many factors, including membrane potential, pH, temperature (body temperature), membrane stretch, heavy metal ions, and lipids. Among them, arachidonic acid, an unsaturated fatty acid, is a known inflammatory lipid mediator that is produced during inflammatory reactions and activates Hv channels. We reported that arachidonic acid directly binds to Hv channels and increases their activation kinetics (The 100th Anniversary Annual Meeting of The Physiological Society of Japan). This suggests that arachidonic acid influences the operation of the voltage sensor. In this study, we focused on this point and performed the following experiments using electrophysiological techniques. We analyzed the correlation between the effect of arachidonic acid on mouse-Hv1 (mHv1) and the extracellular $Zn^{2+}$, which slows down the activation kinetics of the voltage sensor (S4) and keeps it in a resting state. We observed that the activation effect of arachidonic acid on mHv1 was down-regulated by the addition of $Zn^{2+}$ in a dose-dependent manner. To gain more insight into the effect of arachidonic acid, we also analyzed the effects on various S4 mutants that alter the activation kinetics and voltage dependence of mHv1 through electrophysiological and direct interaction analysis. In this presentation, we would like to discuss the effect of arachidonic acid on the operation of the voltage sensor of Hv channels.

### [1P-063]

### IFN-γ Regulates PD-L1 Expression via Orai1 in Glioblastoma

*Mio Mochizuki[1], Masanari Umemura[1], Yuto Mizuno[1], Chihiro Hayashi[1], Fumina Suzuki[1], Akane Nagasako[1], Rina Nakakaji[1], Kagemichi Nagao[1], Yoshihiro Ishikawa[1] ([1]*Cardiovascular Research Institute (CVRI) Yokohama City University Graduate School of medicine*)

**Background:** Anti-tumor immunity plays a pivotal role in attacking and eliminating cancer cells. However, many cancer cell types express PD-L1 (Programmed cell death ligand 1), which suppresses this antitumor immunity by binding to PD-1 (Programmed cell death 1) found on cytotoxic T cells. While it is widely recognized that interferon-gamma (IFN-γ) upregulates PD-L1, the precise regulatory mechanism remains elusive. In our 2014 study, we posited an interesting link, suggesting that store operated $Ca^{2+}$ entry (SOCE), a mechanism underlying persistent $Ca^{2+}$ signaling, regulates proliferation and cell migration in melanoma. Building on this, we hypothesized that SOCE also plays a part in regulating PD-L1 expression in glioblastoma (GB) cells. Our prior research hinted at a potential connection between intracellular $Ca^{2+}$ and this regulation. Consequently, we focused on the Orai1 $Ca^{2+}$ channel, a crucial component of SOCE, to reveal the mechanism by which IFN-γ regulates PD-L1 expression.
**Methods:** We utilized the human GB cell line, LN-229, for this study. Cells underwent treatment with IFN-γ, Orai1 inhibitors (SKF-96365 and YM-58483), and a sarcoplasmic/endoplasmic reticulum $Ca^{2+}$ ATPase (SERCA) inhibitor, Thapsigargin (TG). PD-L1 protein levels were evaluated using western blotting, and mRNA expression levels were quantified via real-time quantitative polymerase chain reaction (qPCR).
**Results and Discussion:** To investigate the role of Orai1, we established Orai1 knockdown cells using lentiviral shRNA system. Inhibition of Orai1, either by specific inhibitors or through lentiviral shRNA knockdown, prevented the upregulation of PD-L1 protein expression induced by IFN-γ. This finding demonstrated that Orai1 regulated the PD-L1 protein expression. Moreover, to assess the impact of intracellular $Ca^{2+}$ influx on PD-L1 protein expression, we treated LN-229 cells with TG to activate the stromal interaction molecule (STIM) and open Orai1. As expected, activation of Orai1 by TG caused an elevation in PD-L1 protein levels. Importantly, this TG-driven protein upregulation was reversed when Orai1 was knocked down by shRNA. These data suggest that Orai1 stimulation leads to an increase in PD-L1 protein expression. In contrast to previous findings, our investigations showed that PD-L1 mRNA levels remained unchanged as measured by qPCR. Taken together, our data suggest that the upregulation of PD-L1 protein expression in response to IFN-γ, mediated by Orai1, might not be due to changes in mRNA levels but could be attributed to other mechanisms, such as ubiquitination.

### [1P-064]

### The allosteric gate mechanisms for retinal CNG and cardiac HCN channels

*Mai Kiritoshi[1], Kenji Akiyoshi[1], Yuya Matsubara[1], Morihiro Shimizu[2], Futoshi Toyoda[1], Mariko Omatsu-Kanbe[1], Masaaki Ogawa[1] ([1]*Department of Physiology, Shiga University of Medical Science,* [2]*Department of Anesthesiology, Shiga University of Medical Science*)

Cyclic nucleotide-regulated ion channels are the nonselective cation channels whose opening is regulated by cAMP or cGMP bindings. Two related families have been identified, the cyclic nucleotide-gated (CNG) channels and the hyperpolarization-activated cyclic nucleotide-gated (HCN) channels. CNG channels play a fundamental role in sensory transduction in retinal photoreceptors and olfactory neurons. HCN channels are present in cardiac pacemaker cells to contribute to the heart rhythm. Both channels share common structural features of six transmembrane tetrameric ion channels where each subunit consists of a voltage-sensing domain (VSD, S1-S4), a pore domain (PD, S5-S6) and a cyclic nucleotide-binding domain (CNBD, cytosolic C-terminus). However, there are functional differences between CNG and HCN channels in their gating. CNG channels are opened by a direct binding of cyclic nucleotides (mainly ligand-gated) but are activated weakly by membrane depolarization. In contrast, HCN channels are principally operated by membrane hyperpolarization (voltage-gated) and their activity is just modulated by cAMP bindings. In the present study, we applied the allosteric modular gating model (Shimizu *et al*., 2022) to explain distinct gating properties of CNG and HCN channels. Human retinal CNGA3 channels and cardiac HCN4 channels were heterologously expressed in HEK or CHO cells and patch-clamp recordings were performed. Based on the experimental data, the model parametrization for CNGA3 and HCN4 channels was successfully accomplished to fit the voltage- and ligand-dependent change in $G/G_{max}$. The newly developed allosteric modular gating models may prove useful to understand the gating behaviors of CNG and HCN channels and predict the mechanism of the drug action.

### [1P-065]

### Molecular mechanisms of histamine-induced cell shrinkage of U937 cells and effects of Sho-seiryu-To and Saiko-keishi-To

*Aoto Aoyama[1], Kentaro Koyabu[1], Ryuma Okada[1], Hirono Sugawara[1], Ayako Sakai[1], Kaori Sato[1], Tomohiro Numata[1] ([1]*Akita University*)

### [1P-066]

### Involvements of $K^+$ channels and $Cl^-$ channels in the cell water excretion effect of Boui-ougi-to

*Taro Suzuki[1], Haruna Saito[1], Ayako Sakai[2], Kaori Sato-Numata[2], Yasunobu Okada[2,3,4,5,6], Tomohiro Numata[2] ([1]*School of Medicine, Akita University, Akita, Japan,* [2]*Department of Integrative Physiology, Graduate School of Medicine, Akita University, Akita, Japan,* [3]*National Institute for Physiological Sciences, Okazaki, Japan,* [4]*Department of Physiology, School of Medicine, Aichi Medical University, Nagakute, Japan,* [5]*Department of Physiology, Kyoto Prefectural University of Medicine, Kyoto, Japan,* [6]*Cardiovascular Research Institute, Yokohama City University, Yokohama, Japan*)

### Poster

### [1P]
### Molecular physiology, Cell physiology
**Others**

March 28, 13:00 - 14:20, Poster Room

### [1P-067]

### Inhibition of PYK2 suppresses PD-L1 expression in breast cancer

*Fumina Suzuki[1], Masanari Umemura[1], Yuto Mizuno[1,2], Chihiro Hayashi[1], Mio Mochizuki[1], Wakana Fukae[1], Soichiro Ishikawa[1,3], Akane Nagasako[1], Yoshihiro Ishiakwa[1] (*[1]Cardiovascular Research Institute (CVRI), Yokohama City University Graduate School of Medicine, [2]Department of Environmental Immuno-Dermatology, Yokohama City University Graduate School of Medicine, [3]Department of Oral and Maxillofacial Surgery, Yokohama City University Graduate School of Medicine*)

[Introduction] Interferon-γ (IFN-γ) augments the expression of the immune checkpoint molecule, programmed death-ligand 1 (PD-L1). Increased PD-L1 expression can suppress tumor immunity and promote tumor growth in various cancer types, including breast cancer. However, the mechanism underlying IFN-γ mediated PD-L1 signaling remains not fully elucidated. This study aims to shed light on this signaling pathway and to identify potential novel inhibitors for breast cancer treatment. [Materials and methods] We utilized the MDA-MB-468 human breast cancer cell line for our experiments. Quantitative reverse transcription PCR (qRT-PCR) was employed to assess gene expression levels. VS-6063, an inhibitor of proline-rich tyrosine kinase 2 (PYK2) and focal adhesion kinase (FAK), was used. Additionally, we evaluated the effects at the protein level using western blot (W.B.) analysis. [Results] To identify candidate genes involved in the regulation of PD-L1 expression, we conducted a linear regression analysis using mRNA samples derived from human breast cancer tissues. This analysis revealed a significant correlation between PYK2 and PD-L1 mRNA levels. Subsequent qRT-PCR experiments in MDA-MB-468 cells confirmed that IFN-γ indeed enhances PD-L1 mRNA transcription. W.B. analyses further demonstrated that VS-6063 could negate IFN-γ-induced PD-L1 protein expression, suggesting that VS-6063 can attenuate IFN-γ-stimulated upregulation of PD-L1 at both the mRNA and protein levels. [Conclusion] Our findings indicate that PYK2 serves as a critical intermediary in IFN-γ-induced PD-L1 signaling. Thus, PYK2 inhibitors might represent promising candidates to enhance the efficacy of immune checkpoint inhibitors in breast cancer therapy.

### [1P-068]

### Enhancement of ciliary bend amplitude stimulated by Ryu-kaku-san in ciliated normal human bronchial epithelial cells.

*Taisei Tsujii[1,2], Kikuko Amagase[1], Yoshinori Marunaka[2,3], Takashi Nakahari[2] (*[1]Laboratory of Pharmacology & Pharmacotherapeutics, Graduate School of Pharmaceutical Sciences, Ritsumeikan University, Shiga, Japan, [2]Research Unit for Epithelial Physiology, Research Organization of Science and Technology, BKC, Ritsumeikan University, Kusatsu, Japan, [3]Medical Research Institute, Kyoto Industrial Health Association, Kyoto, Japan*)

Ryu-kaku-san (a frequently prescribed traditional herbal medicine) is used to facilitate expectoration and to prevent coughing in Japan. However, the effects of Ryu-kaku-san on the airway ciliary beating remain uncertain. [Method] Ryu-kaku-san was dissolved by water (500 mg/mL). After centrifugation of Ryu-kaku-san dissolved solution, the supernatant was diluted to its final concentration just before the experiments. We used ciliated normal human bronchial epithelial cells (c-NHBEs), which were differentiated from the NHBE by the air liquid interface (ALI) culture. Ciliated-NHBEs were set and perfused on the stage of an inverted light microscope and their beating cilia were observed by a high-speed video microscopy (HSVM). After the experiments, the images recorded by the HSVM were analyzed by an image analysis program and the frequency (CBF) and amplitude (CBD, ciliary bend distance, an index of the amplitude) were measured to assess the activity of the ciliary beating. Experiments were carried out at 37 °C. [Results] Stimulation with Ryu-kaku-san ranging from 1 mg/mL to 50 mg/mL enhanced CBD, but not CBF, in a concentration dependent manner in c-NHBEs. Ryu-kaku-san at 20 mg/mL, which maximally enhanced CBD by 45% in c-NHBEs, was used for the experiments. A nominally $Ca^{2+}$-free solution or the prior treatment of PKI-A (1 µM, an inhibitor of PKA) did not affect the CBD enhanced by Ryu-kaku-san, suggesting that it stimulates neither $Ca^{2+}$ mobilization nor cAMP accumulation in c-NHBEs. A decrease in $[Cl^-]_i$ has already been shown to enhance CBD, not CBF, in airway ciliary cells. We examined the effects of an inhibitor of $Cl^-$ channels (10 µM NPPB) on CBD enhanced by Ryu-kaku-san. The addition of NPPB alone decreased CBF and CBD by 5%, and the further stimulation with Ryu-kaku-san did not enhance CBD or CBF. Thus, an increase $[Cl^-]_i$ induced by NPPB appears to abolish the increase in CBD stimulated by Ryu-kaku-san in c-NHBEs. Moreover, the addition of bumetanide (10 µM, an inhibitor of NKCC) alone increased CBD, not CBF, and then the stimulation with Ryu-kaku-san increased CBD, indicating that it does not inhibit NKCC. [Conclusion] Ryu-kaku-san appears to enhance CBD, not CBF, mediated by a decrease in $[Cl^-]_i$ which enhances CBD in c-NHBEs. It may stimulate $Ca^{2+}$-insensitive $Cl^-$ channels, leading to activate $Cl^-$ secretion.

### [1P-069]

### Reduced intracellular $Cl^-$ concentration promotes cell migration and invasion via activation of the Ras-ERK signaling pathway and facilitation of matrix metalloproteinase-1 (MMP-1) expression in DU145 cells

*Hiroaki Miyazaki[1], Koya Nakano[1], Junichi Sato[1] (*[1]Department of Life Science, Faculty of Science and Engineering, Setsunan University*)

Our previous study revealed that changes of the intracellular $Cl^-$ concentration ($[Cl^-]_i$) affected cell proliferation in cancer cells. However, the role of $Cl^-$ on cell migration and invasion in cancer cells remains unanalyzed. Therefore, the aim of the present study is to investigate whether changes of $[Cl^-]_i$ affects cell migration and invasion of cancer cells. In human prostate cancer DU145 cells, cell migration and invasion were enhanced by culturing in the low $Cl^-$ medium (replacement of $Cl^-$ by $NO_3^-$). Since the previous studies suggested that ERK/MAPK signaling has been involved in cell migration and invasion in several types of cancer, we first examined the effect of $Cl^-$ on the activation of ERK. In the low $Cl^-$ condition, phosphorylation levels of ERK were transiently upregulated. The inhibition of ERK activation by the application of MEK inhibitor, U0126, completely abolished the enhancement of cell migration and invasion in the low $Cl^-$ condition. From these results, we concluded that the enhancement of cell migration and invasion of DU145 cells in the low $Cl^-$ condition is due to the activation of ERK. We subsequently considered how the increased levels of ERK phosphorylation in the low $Cl^-$ environment was accompanied by facilitation of its invasive potential. We then investigated whether matrix metalloproteases (MMPs) are involved in facilitating the invasive ability of DU145 cells under low $Cl^-$ conditions. As a result, we found that DU145 cells in the low $Cl^-$ condition caused significant transient ERK1/2 activation followed by an increase of MMP-1 mRNA levels. Inhibition of ERK1/2 activation in the low $Cl^-$ condition reduced enhancement of MMP-1 mRNA levels and decreased cell migration and invasion. These observations indicate that $[Cl^-]_i$ plays important roles in metastatic function by regulating the ERK1/2 signaling pathway in human prostate cancer cells, and intracellular $Cl^-$ would be one of the key targets for anti-cancer therapy.

### [1P-070]

### Effect of cell membrane lipids to sphingosylphosphorylcholine-induced vascular smooth muscle cell contraction

*Natsuko Tsurudome[1], Yuji Minami[2], Katsuko Kajiya[2] ([1]*Department of Cardiovascular Physiology, Faculty of Medicine, Kagawa University, Japan,* [2]*Department of Food Science and Biotechnology, Faculty of Agriculture, Kagoshima University, Japan*)

Sphingosylphosphorylcholine (SPC), one of the sphingolipids, is a causative factor of vasospasm. We reported that it induces the contraction of vascular smooth muscle cells (VSMC) and that fisetin, a flavonoid can prevent it. However, the pathogenesis and preventive mechanisms have not been fully understood. Lipid rafts, part of the plasma membrane, are considered to be associated with this mechanism, but the composition of VSMC lipid rafts remains largely unknown, making it difficult to understand the mechanism. In this study, we aimed to elucidate the lipid composition of VSMC lipid rafts and evaluate the permeability of SPC and fisetin to liposomes based on lipid composition to reveal their interaction.
We isolated lipid raft fractions enriched in flotillin1, a marker protein of lipid raft, from VSMCs using sucrose density gradient centrifugation, and analyzed their lipid composition by LC-ESI-MS/MS. From the results, we prepared liposomes as a model for lipid rafts or the plasma membrane by changing their cholesterol composition. The permeability analysis of SPC and fisetin to these liposomes also examined other related compounds based on the structures of SPC and fisetin to clarify their structural properties.
As a result, flotillin1 was enriched in fraction 6 considered a lipid raft fraction. Lipid rafts in SPC-stimulated VSMCs were enriched in phosphatidylcholine, dimethyl-phosphatidyl ethanolamine, and phosphatidylethanolamine compared with unstimulated cells. SPC tended to be less permeable to liposomes of any lipid composition, while fisetin exhibited high permeability. Notably, the permeability of SPC was significantly influenced by the cholesterol content in liposomes that mimicked the composition of VSMC lipid rafts.
From this study, we considered that SPC induces VSMC contraction without permeating the plasma membrane, while fisetin permeates the plasma membrane and prevents SPC-induced contraction. These results help us understand the mechanism of SPC and fisetin on SPC-induced VSMC contraction.

### [1P-071]

### Identification of the amino acid sequences that contribute to HAP1 cytoprotection under the conditions of proteasome inhibition

*Kanako Nozaki[1], Akie Yanai[1], Islam Md Nabiul[1], Koh-hei Masumoto[1], Shinoda Koh[1]
([1]*Yamaguchi University Graduate School of Medicine*)

HAP1 (huntingtin-associated protein 1), a major component molecule of stigmoid body (STB), has been suggested to be an intrinsic cytoprotective factor.The decline of proteasome activity with aging is one of the triggering factors for most neurodegenerative diseases. Recently, we found that HAP1 has a protective effect against apoptosis under proteasome inhibition. Therefore, we hypothesis that STB/HAP1 is the key factor to escape cellular degradation in many neurodegenerative diseases associated with aging. For the clinical application of STB/HAP1 protective function, it is important to identify the amino acids sequence that are essential for the cytoprotective action. Therefore, in this study, we attempted to identify the site among HAP1 amino acids that is directly related to the cytoprotective function.The cDNA encoding HAP1 was fragmented, and these fragments were inserted into the expression plasmids, and the gene were transfected into the immortalized cells derived from mouse neurons that does not express endogenous HAP1. Then, the cells were treated with proteasome inhibitor and were collected for the western blot analysis. Western blot for cleaved PARP, an apoptosis indicator, showed that the expression of cleaved PARP in the cells expressed HAP1 fragment (xxx-yyy) was drastically decreased compared to that expressed endogenous full-length HAP1. The result suggests that fragmented HAP1 (xxx-yyy) had even stronger cytoprotective effects than the original full-length HAP1. Since the cytoprotective effect of HAP1 was enhanced by fragmentation of the amino acid sequence, the removal of amino acid sequences that are not necessary for protective functions might lead to get more effective HAP1 molecule for therapeutic purposes.

(COI: NO)

### [1P-072]

### Effect of reduced intracellular Cl– concentration on cell proliferation in a human breast cancer cell line, MDA-MB231 cells

*Koya Nakano[1], Hiroaki Miyazaki[1] ([1]*Department of Life Science, Faculty of Science and Engineering, Setsunan University*)

The intracellular Cl– concentration ([Cl–]i) of cancer cells is assumed to change in tumor microenvironments in vivo because high CO2 partial pressure due to high metabolic activity of cancer cells promotes Cl–/HCO3– exchange transport via anion exchangers. Although our previous study showed that proliferation of MDA-MB231 cells is significantly diminished in the low Cl– (replacement of NaCl with NaNO3) condition, its mechanism has been largely unknown. In this study, we investigated the mechanism of cell growth inhibition by reduction of [Cl–]i. Accordingly, we first examined the effects of [Cl–]i on cell-cycle progression by using Cell-Clock Cell Cycle Assay Kit. Interestingly, results of cell cycle analysis showed that there were no significant differences in cell cycle profile patterns between cells cultured in the normal or the low Cl– condition. This result suggests that MDA-MB231 cells cultured in the low Cl– condition prolonged the duration of all phases of the cell cycle (G1, S and G2/M). To elucidate the mechanism of cell cycle delay, we confirmed the protein expression level of p21 (a CDK inhibitor) and the phosphorylation level of histone H3, which are respectively key factors of transition from G1 to S phase and G2 to M phase in cell cycle progression. As a result, there were no significant differences between p21 protein expression in the normal and the low Cl– condition. On the other hand, phosphorylation of histone H3, which is strongly correlated with the transition from G2 to M phase, was diminished in the low Cl– condition. These results suggest that cell cycle transition from G2 to M phase of MDA-MB231 cells was prolonged under the low Cl– condition. We also evaluated 5-ethynyl-2'-deoxyuridine (EdU; a thymidine analogue) incorporation in MDA-MB231 cells to evaluate the effect of the low Cl– condition on S phase progression. The results showed that the incorporation of EdU into MDA-MB231 cells was significantly reduced in the low Cl– condition. Thus, it is clear that S-phase progression is also delayed in the low Cl– condition. Based on these results, we are now investigating the effect of Cl– on cell cycle progression using cell cycle synchronized cells to further clarify the role of Cl– on cell cycle progression in MDA-MB231 cells.

### [1P-073]

### The analysis of change in subcellular localization of a putative sleep regulator, ARHGEF2 via SIK3

*Ami Kato[1], Kei Nishida[2], Qianyun Gao[2], Tomohiro Kiatazono[2], Masashi Yanagisawa[2]
([1]*Oita Univ.,* [2]*International Institute for Integrative Sleep Medicine University of Tsukuba*)

### [1P-074]

### Inflammatory amplification mechanism between $PGE_2$-EP4 signals and TLR2 signals in abdominal aortic aneurysm

*Mirai Osanai[1], Takashi Nakamura[1], Taro Hiromi[1], Utako Yokoyama[1] ([1]*Department of Physiology, Tokyo Medical University*)

### [1P-075]

### Elucidation of the mechanism of IL-6 synergistic amplification induced by prostaglandin $E_2$ receptor EP4 and Toll-like receptor 2 signals

*Ryu Mitsuhashi[1], Takashi Nakamura[1], Utako Yokoyama[1] ([1]*Tokyo Medical Univ.*)

### Poster

### [1P]
### Muscle

March 28, 13:00 - 14:20, Poster Room

### [1P-076]

**Effects of temperature on water-induced tension response of the guinea pig taenia caecum**

*Yukisato Ishida[1], Masaru Watanabe[1], Naoya Nakahara[2], Shigeru Takemori[2] ([1]*Graduate School of Human Health Sciences, Tokyo Metropolitan University,* [2]*Department of Molecular Physiology, Jikei University School of Medicine*)

Muscle contractile responses to water were reported (Herman, 1879; Asano, 1916; Noguchi et al., 1955). Natori (1954) reported the distilled water-induced contractile response of Natori's skinned fiber, which was recently confirmed by us. We also reported the properties of contractile responses to pure water ($H_2O$) in smooth muscle of the guinea pig taenia caecum (Ishida *et al*. Proc 100th Ann Meeting PSJ, p99, 2023). Here we present the distinct effects of low (7, 17 and 25°C) and high (41°C) temperatures on the $H_2O$-induced tension of the taenia. When the taenia was properly stretched, the exposure to $H_2O$ reproducibly elicited a sustained tension at 33°C. When the temperature was lowered, the magnitude of maximum response to $H_2O$ was not affected, but the sustained tension at 7°C was attenuated by 15%. Half times to peak tension, approximately 45 sec at 33°C, were apparently prolonged 2.2-2.5 times at 17 and 7°C. Half times in decay response to re-exposure to PSS, about 3 sec at 33°C, were also prolonged 1.5 times at 7 and 17°C. On the other hand, raising temperature to 41°C attenuated the peak and sustained responses to $H_2O$ by 25 and 70% of those at 33°C respectively, and seemed to accelerate the raising phase, but not decaying phase, of tension response to $H_2O$. Contractile responses to high $K^+$ (45.6 mM) and carbachol (10 μM) were attenuated at both low and high temperatures. These alterations at low and high temperatures were nearly reversible, when temperature was reversed to 33°C. These results indicate that the $H_2O$-induced tension of the taenia is resistant to low, but not high, temperature. Presumably, the association of proteins contributes to the tension response to $H_2O$ in the taenia, although the contribution of actomyosin has yet been obscure. (COI: NO)

### [1P-077]

**Analysis of skeletal muscle fiber-specific KO mice for Acin1, a peripheral factor of exon junction complex**

*Mamoru Aoto[1], Hiroshi Sakai[2,3], Naohito Tokunaga[4], Mei Miyazaki[4], Yuki Imai[2,3], Noriaki Mitsuda[1] ([1]*Department of Circulatory Physiology, Ehime University Graduate School of Medicine, Japan,* [2]*Division of Integrative Pathophysiology, Proteo-Science Center, Ehime University, Japan,* [3]*Department of Pathophysiology, Ehime University Graduate School of Medicine, Japan,* [4]*Division of Medical Research Support the Advanced Research Support Center (ADRES), Ehime University, Japan*)

In general, gene expression is not only subject to transcriptional regulation, but also to post-transcriptional regulation, including splicing, mRNA modification, transport, stabilization, and translation, in which many RNA-binding proteins are involved. Alternative splicing (AS), a form of post-transcriptional regulation, is an important mechanism for generating multiple protein isoforms from a single gene that differ not only in structure but also in localization, expression level, and biological function. For example, more than 90% of human genes are subject to AS, and it has become clear that dysregulation of AS leads to severe tissue dysfunction and causes disease. In recent years, many AS events have been identified during myogenesis. A large number of abnormal AS events have been identified in several types of muscular dystrophies, all of which are associated with abnormalities in muscle regeneration.

Acin1 (Acinus), a peripheral factor of the exon junction complex, has an RNA recognition sequence and has been implicated in the regulation of AS. We found that Acin1 is highly expressed in the nuclei within myofibers (myonuclei) in skeletal muscle by immunostaining against Acin1, and therefore we generated myofiber-specific Acin1 KO mice (KO mice) to analyze the physiological function of Acin1 in skeletal muscle. As a result, the weight of lower limb skeletal muscles (tibialis anterior, gastrocnemius, and soleus muscles) was increased in KO mice compared to control (Ctrl) mice, and the cross-sectional area of myofibers, number of central nuclei, and expression of muscle regeneration marker were increased in tibialis anterior muscle, suggesting that loss of Acin1 causes some damage to myofibers and induces muscle regeneration. This was strongly suggested by RNA-Seq analysis of tibialis anterior muscle isolated from Ctrl and KO mice, which showed that KO mice had increased expression of mRNAs involved in muscle differentiation and muscle regeneration. In addition, analysis of genes with altered AS events in KO mice using two different software packages revealed significant differences in 44 and 16 AS events, respectively, of which 5 events were shared. These results indicated that Acin1-mediated AS has a role in maintaining skeletal muscle physiology. (COI: No)

### [1P-078]

**Motor function of zebrafish lacking voltage-gated sodium channels in muscles**

*Souhei Sakata[1], Fumihito Ono[1] ([1]*Osaka Medical and Pharmaceutical University*)

It is an established view that the action potential is indispensable for the muscle contraction. However, as reported in the past annual meetings of the Physiological Society of Japan, we found that zebrafish larva lacking voltage-gated sodium channels in muscle (NavDKO) was able to swim like WT fish and $[Ca^{2+}]i$ in WT muscle fiber was elevated by the application of acetylcholine even in the presence of 1 μM tetrodotoxin. These demonstrated that muscle fiber of zebrafish larva contracts in the absence of the action potential. We simulated the membrane potential in the condition that muscle fiber lacks the voltage-dependent sodium conductance and found that the membrane was depolarized over 0 mV if the synaptic currents were simultaneously injected at all neuromuscular junctions (NMJs). The simulation also suggested that the depolarization over 0 mV was achieved by the small size of the fiber relative to the amplitude of the synaptic current. This raises an idea that a large fish lacking Nav is unable to swim like WT fish. Therefore, we accessed the swimming capability of the adult NavDKO using the escape response and the swimming treadmill. The escape response was evoked by pinching the tail of the fish in the methylcellulose solution. We estimated the maximum turn speed and did not find a significant difference between WT and the NavDKO. In the treadmill, tested fish swam in a chamber in which the flow rate is variable. The flow rate was increased 1 cm/s every minute starting from 20 cm/s, and the flow rate at which the fish failed to resist the flow was designated the critical swimming speed (Ucrit). We will show the results of the swimming capability of the NavDKO examined by the swimming treadmill and discuss the physiological implication of the Nav channels in zebrafish muscle in the meeting.

### [1P-079]

### The role of muscle satellite cells in muscle hypertrophy

*Yanzhu Chen[1], YUBING DONG[1], Kimiaki Katanosaka[2], Keiji Naruse[1], Yuki Katanosaka[1,3] ([1]*Department of Cardiovascular Physiology, Graduate School of Medicine, Dentistry and Pharmaceutical Sciences, Okayama University,* [2]*College of Life and Health Sciences, Chubu University,* [3]*College of Pharmacy, Kinjo Gakuin University*)

**Aims:** Mechanical overloading of muscle is known to cause muscle hypertrophy, but the detailed mechanism remains unclear. In this study, we examined the role of the transient receptor potential vanilloid family type 2 (TRPV2) in muscle satellite cells (MuSCs) in the onset of muscle hypertrophy induced by mechanical loading.
**Methods and Results:** Detailed analysis of TRPV2 expression in muscle tissue and isolated muscle fibers revealed that TRPV2 is strongly expressed in specific fast-twitch muscle fibers and in MuSCs during muscle regeneration. Therefore, we generated MuSCs-specific *TRPV2*-deficient mice and analyzed mechanical overload-induced muscle hypertrophy. The plantar muscles of foxed-TRPV2 mice showed markedly hypertrophy to mechanical loading for 2 weeks. Isolated cells from the hypertrophied plantar muscles had approximately 1.5-fold increase in the number of nuclei compared to the pre-loaded or vehicle group. On the other hand, plantar muscles of MuSCs-specific *TRPV2*-deficient mice show no hypertrophy and change in the number of nuclei to mechanical overloading. This indicates that the fusion of MuSCs is also involved in the muscle hypertrophy induced by mechanical overloading. MuSCs isolated from floxed-TRPV2 mice showed increased expression of TRPV2 and marked $Ca^{2+}$ oscillation early in the fusion process. Addition of tranilast, an inhibitor of TRPV2, inhibited $Ca^{2+}$ oscillation and cell fusion between MuSCs. This suggests that TRPV2 is involved in the fusion process of muscle satellite cells.
**Conclusions:** These results indicate that TRPV2 in MuSCs plays an important role in muscle hypertrophy induced by mechanical overloading. The results of this study may provide knowledge for the future development of prevention and treatment strategies for sarcopenia and other disorders.

### [1P-080]

### Elucidation of the muscle pathophysiology in overuse using a repetitive isometric contraction model with electrical stimulation

*Hiroyori Fusagawa[1,2], Takashi Yamada[3], Tatsuya Sato[1,4], Azuma Naito[3], Nao Tokuda[3], Nao Yamauchi[3], Nobutoshi Ichise[1], Takuro Karaushi[1], Toshifumi Ogawa[1,4], Atsushi Teramoto[2], Noritsugu Tohse[1] ([1]*Department of Cellular Physiology and Signal Transduction, School of Medicine, Sapporo Medical University,* [2]*Department of Orthopaedic Surgery, School of Medicine, Sapporo Medical University,* [3]*Graduate School of Health Science, Sapporo Medical University,* [4]*Department of Cardiovascular, Renal and Metabolic Medicine, School of Medicine, Sapporo Medical University*)

**Background**: Overuse occurs in many manual laborers and athletes who do not get adequate rest, and a significant number of patients suffer from muscle pain, weakness, and stiffness. However, intramuscular disorders in overuse are not well understood because of the difficulty in obtaining samples from healthy workers and athletes. **Methods and Results**: In the first experiment, six 6-week-old male Wistar rats were used, with the left leg as the overuse side and the right leg as the unloaded control side, and isometric contraction was induced by electrical stimulation using surface electrodes to the plantar flexor muscles, which were fixed at the ankle joint to a plantar plate connected to a tension transducer. Isometric exercise using electrical stimulation (4 sets of 5 contractions at 5-min intervals consisting of 2-s contraction with 100 Hz, 0.5 ms, 45 V every 6 s) was performed daily for 2 weeks as an overuse load. Isometric torque of the plantar flexor muscles was measured daily, and Hematoxylin-Eosin (HE) and Masson-Trichrome (MT) staining were performed on gastrocnemius muscle tissue sections 24 hours after the final load. Maximal isometric was significantly decreased on the overuse side from the fourth day of loading to the last day of loading compared to the control side. In histological analysis of the gastrocnemius muscle after 2 weeks of loading, the HE-stained tissue sections on the overuse side showed areas of disruption of normal muscle tissue, occupied 8.2% of the total area. The area was confirmed to be replaced partly by collagen fibers by MT staining. In the second experiment, five rats were trained for 1 to 5 days each, and HE and Evans blue dye (EBD) staining were performed on gastrocnemius muscle tissue sections. EBD staining showed no detectable muscle damage from the first day to the fifth day of overuse, and a marked inflammatory cell infiltrate was observed between muscle fibers on fifth day.
**Conclusions**: We confirmed progressive onset of muscle dysfunction and fibrosis in the rat plantar flexor muscles after repeated isometric contraction loading every day, which occurs without apparent muscle damage. The identification of a novel muscle physiological response to overuse is an important finding for the development of therapeutic interventions for physically inactive workers who do not get enough rest.

### [1P-081]

### Behavior analysis of locomotor performance transition using acetylcholine receptor ε subunit-deficient zebrafish

*Toshiki Tokuno[1], Mikoto Nakajo[1], Shintaro Matsunada[1], Fumihito Ono[1] ([1]*Osaka Medical and Pharmaceutical University*)

### [1P-082]

### The role of vimentin cleavage in the abnormal vascular smooth muscle contraction

*Keisuke Shigenobu[1,2], Tatsuo Miyamoto[1], Kentaro Matuzaki[3], Yoko Tanabe[3], Hiroko Kishi[1,3] ([1]*Department of Molecular Physiology, Yamaguchi University Graduate School of Medicine,* [2]*Fourth year at Yamaguchi University School of Medicine,* [3]*Department of Environmental Physiology, Faculty of Medicine, Shimane University*)

### [1P-083]

### Comparison of nicotinic acetylcholine receptors in murine muscle

*Chihiro Nishiwaki[1], Eriko Daikoku[1], Fumihito Ono[1] ([1]*Osaka Medical and Pharmaceutical University*)

### Poster

### [1P]
### Digestion, Digestive system

March 28, 13:00 - 14:20, Poster Room

### [1P-084]

### Analysis of the regulation mechanism of intestinal motility by bitter substances

*Masato Ota[1], Tomomi Hara[1], Sachina Yoshida[1], Mao Fujiwara[1], Reina Torii[1], Tomomi Imaeda[1], MIki Takahashi[1], Atsuko Yamashita[1], Takashi Kondo[2] ([1]*Japan Women's University,* [2]*RIKEN-IMS*)

It is known that taste stimuli and components such as umami and sweet taste reaching the intestinal tract activate vagal afferents and reflexively alter autonomic nervous system activity in the vagal centrifugal tract. Although it has been reported from heart rate analysis that bitter taste components increase sympathetic activity, their effects on intestinal motility are not clear. The aim of this study was to observe the effects of bitter substances on intestinal motility and to investigate the regulatory mechanisms of bitter substances. Quinine, denatonium and thiamine were used as bitter substances and their effects on rhythmic contraction movements were investigated using excised intestinal tracts. As these bitter substances have different types of bitter taste receptors, gene expression of a number of bitter taste receptor families in the intestinal tract was investigated by PCR. In vivo experiments in wild-type mice showed that thiamine altered contractile movements in the stomach and small intestine, but these effects of thiamine were attenuated in knockout mice of the bitter taste receptors Tas2R119 and TA2R139, which are ligand for thiamine. It was strongly suggested that thiamine modulates gastric and intestinal contractile movements via Tas2R119 and TA2R139.

### [1P-085]

### Inhibitory effects of somatostatin on glucagon-like peptide-1-mediated peristalsis in the rat proximal colon

*Hiroyuki Nakamori[1], Fuko Hosoi[1], Hikaru Hashitani[1] ([1]*Department of Cell Physiology, Nagoya City University Graduate School of Medical Sciences, Nagoya, Japan.*)

In the colonic epithelium, three major types of enteroendocrine cells are distributed, namely serotonin-secreting enterochromaffin cells, glucagon-like peptide-1 (GLP-1)-secreting L cells and somatostatin-secreting D cells. Since L cell-derived GLP-1 accelerates peristalsis in the proximal colon, somatostatin released from D cells that inhibits GLP-1 secretion by activating somatostatin receptor subtype 5 (SSTR5) on L cells, may inhibit the GLP-1-induced peristalsis acceleration. Here, effects of exogenous somatostatin on GLP-1-induced acceleration of colonic peristalsis were investigated. Cannulated segments of rat proximal colon were placed in an organ bath, serosally perfused with oxygenated physiological salt solution, and luminally perfused with disgassed 0.9% saline. All drugs were applied intraluminally. Colonic wall motion was recorded using a webcam and converted into spatio-temporal maps. Somatostatin (1 µM) alone changed neither the frequency of oro-aboral propagating peristaltic contractions nor the minimum colonic diameter, representing the colonic contractility. GLP-1 (30 nM but not 10 nM) increased the frequency of peristaltic waves and reduced the minimum diameter. In the presence of somatostatin (1 µM), GLP-1 (30 nM) failed to accelerate colonic peristalsis. In the colonic segments that had been pretreated with SSTR5 antagonist 1 (300 nM), a selective SSTR5 antagonist, GLP-1 (10 nM) became capable of increasing the frequency of peristaltic waves without changing the minimum diameter, while SSTR5 antagonist 1 alone had no effect on colonic peristalsis. Thus, somatostatin is capable of counteracting the acceleratory effect of GLP-1 on colonic peristalsis through the activation of SSTR5. In the proximal colon, peristalsis may well be regulated by the functional interaction between excitatory L cells and inhibitory D cells in response to changes in colonic luminal environments.

### [1P-086]

### Gastrointestinal Implications of High Salt Diet: Insights from a Mouse Model

*Masumi Eto[1], Yohei Mochizuki[1], Toshiyasu Matsui[1], Minami Ohashi[1], Yuki Shimojima[1], Masakatsu Nohara[1], Ikki Mitsui[1], Kosuke Takeya[1], Yoshinori Tanaka[1], Risuke Mizuno[1] ([1]*Okayama University of Science Department of Veterinary Medicine*)

Excessive salt intake is a pervasive global health concern, affecting individuals across age, gender, and socio-economic backgrounds. This issue has been extensively linked to an elevated risk of hypertension, cardiovascular diseases, and renal complications. However, the influence of a high salt diet (HSD) on gastric functions remains a relatively unexplored area of research. In the course of investigating the impacts of HSD on circulatory systems, we made an intriguing discovery: a deformation of the stomach in mice and examined dysfunctions in stomach emptying. For a duration of either 2 or 4 weeks, mice were subjected to HSD, and then subjected to a series of assays to determine effects of HSD on gastric function and structure through ultrasonography and histology, as well as characterizing high potassium- and carbachol-induced contractions of smooth muscle strips, and protein expression. HSD induced a marked extension of the gastric fundus area (forestomach) in mice when compared to those on a normal salt diet (NSD). The expansion of the stomach walls was associated with a significant reduction in thickness, and notably devoid of any signs of pathological apoptosis. Ultrasonography showed impaired gastric motility in HSD mice, which was further corroborated by suppressed gastric emptying in these animals. To delve deeper into the mechanisms underlying these changes, we examined the response of smooth muscle strips to high potassium and carbachol-induced contractions, as well as the expression of various proteins. Our results demonstrated that smooth muscle strips from HSD mice exhibited heightened sensitivity to inhibitors of Rho-associated protein kinase (ROCK), including H1152 and SR3677, as well as protein kinase C and phosphodiesterase antagonists when compared to NSD mice. This increased sensitivity was associated with an upregulation of ROCK expression in HSD mice. Furthermore, collagen deposition was evident in the smooth muscle layers of the gastric walls in HSD mice, suggesting the development of fibrosis. Thus, a high-salt diet may lead to gastric dysfunctions through disturbed the $Ca2+$ sensitization signaling, contributing to both structural and functional changes in the stomach. Supported by research funds from Society for Research on Umami Taste and JSPS KAKENHI JP19K06573 and 23K06339 (to ME). No COI.

### [1P-087]

### Investigation of effects of cholecystokinin-8 in the spinal defecation center

*Takuto Matano[1], Takagi Ryuuta[1], Ueda. H. Hiromi[1,2], Fujimoto Yoshikazu[3], Shiraishi Mitsuya[1], Naitou Kiyotada[1] ([1]*Department of Basic Veterinary Science, Joint Faculty of Veterinary Medicine, Kagoshima University,* [2]*Supportive Center for Brain Research, National Institute for Physiological Sciences,* [3]*Transboundary Animal Diseases Research Center, Joint Faculty of Veterinary Medicine, Kagoshima University*)

### [1P-088]

### Effect of aging on claudin-4 expression and paracellular leakage of amino acids in colonic epithelial cells

*Shunsuke Matsuda[1], Ema Okamoto[1], Yuta Yoshino[1], Yoshifumi Morikawa[2], Koichi Suenami[2], Yoshiaki Tabuchi[3], Toshiyuki Matsunaga[1], Hisayoshi Hayashi[4], Akira Ikari[1] ([1]*Gifu Pharmaceutical University,* [2]*Gifu Prefectural Police Headquarters,* [3]*University of Toyama,* [4]*University of Shizuoka*)

### [1P-089]

### Enhancement of colorectal motility by stimuli to bladder in rats

*Nanami Ashiya[1], Natsufu Yuki[2], Tomoya Sawamura[2], Takahiko Shiina[1,2], Yasutake Shimizu[1,2] ([1]*Laboratory of Physiology, Joint Department of Veterinary Medicine, Faculty of Applied Biological Sciences, Gifu University,* [2]*Laboratory of Physiology, Joint Graduate School of Veterinary Sciences, Gifu University*)

### Poster

### [1P]
### Oral physiology

March 28, 13:00 - 14:20, Poster Room

### [1P-090]

### Effects of Rice-*koji* extracts and ergothioneine on anxiety- and pain-like responses under psychophysical stress conditions in mice.

*Kajita Piriyaprasath[1], Yuya Iwamoto[1,2], Mana Hasegawa[1,2], Yoshito Kakihara[3], Noritaka Fujii[2], Kensuke Yamamura[1], Keiichiro Okamoto[1] ([1]*Division of oral physiology, Graduate School of Medical and Dental Sciences, Niigata University, Niigata City, Japan,* [2]*Division of General Dentistry and Dental Clinical Education Unit, Graduate School of Medical and Dental Sciences, Niigata University, Niigata, Japan,* [3]*Division of Dental Pharmacology, Graduate School of Medical and Dental Sciences, Niigata University, Niigata, Japan*)

Rice-Koji, a starter in the brewing of Sake, is a traditional rice-fermented food in Japan. This study aimed to determine the effect of Rice-Koji on stress-induced nociception in male mice. In the parallel experiment, the effects of ergothioneine (EGT), contained in Rice-Koji, were also assessed to determine if EGT could exert similar effects to Rice-Koji. Repeated forced swim stress (FST) conditionings for 3 days were employed to induce psychophysical stress conditions. Rice-Koji or EGT was administered orally twice/day. Anxiety- and pain-like behaviors in the hindpaw were determined by multiple behavioral tests. Anxiety- and pain-like behaviors were increased after FST; however, both Rice-koji and EGT prevented an increase in anxiety- and pain-like behaviors. The c-Fos immunoreactivities were conducted to assess the effect of Rice-Koji and ERGO on neural responses in the brain. In the paraventricular nucleus of the hypothalamus, saline-treated FST mice showed a decrease in c-Fos expression evoked by formalin when compared with saline-treated Sham mice; however, EGT increased c-Fos expressions compared with saline treatment. In the nucleus raphe magnus and lumbar spinal dorsal horn, the c-Fos expression was increased after FST in the saline treatment group; however, both Rice-Koji and EGT decreased formalin-evoked c-Fos expressions. In vitro experiments were conducted on SH-SY5Y cells. MTT assay revealed that Rice-Koji and EGT displayed no significant effect on cell viability. Rice-Koji decreased the level of brain-derived neurotrophic factor (BDNF), while EGT exerted bidirectional changes. These findings indicate that Rice-Koji and EGT could have the ability to regulate neural responses without causing harmful effects on the neural cells. In conclusion, daily consumption of Rice-Koji is beneficial in reducing stress-induced pain-like responses, possibly mediated through the actions of ERGO contained in Rice-Koji.

### [1P-091]

### Changes in lick rates to NaCl and KCl during riboflavin deficiency

*Toshiaki Yasuo[1], Shusuke Iwata[1], Takeshi Suwabe[1], Shinpei Takahashi[1], Noritaka Sako[1] ([1]*Asahi university*)

To investigate the effects of taste system during riboflavin deficiency, we conducted behavioral, anatomical, and molecular biological experiments with rats. Rats in the riboflavin-deficient group were given riboflavin-free diet for 25 days. Rats in the normal group were given normal diet. In behavioral study, we assessed ingestive behavior using 10-s lick tests in order to check taste sensitivity. In an anatomical study, we analyzed the number of fungiform papillae taste buds per unit area in the anterior two-thirds of the tongue. In a molecular biological study, we measured gene expression of taste-related molecules such as *Slc1a3*, *Gnat3*, *Trpm5*, *Car4*, *Scnn1a*, and *Calhm1* in taste buds. As a results, licking rates for NaCl and KCl in riboflavin-deficient rats were significantly higher than those in normal rats. There was no significant difference in the number of fungiform papillae taste buds per unit area in the riboflavin-deficient rats relative to the normal rats. There were no significant changes in the mRNA expression of several taste-related molecules in the fungiform papillae taste bud cells of riboflavin-deficient and normal rats. These results suggest that sodium taste sensitivity and/or preference may change during riboflavin deprivation. Further investigation is required.

### [1P-092]

### Capsaicin enhances gustatory responses to sugars of mouse chorda tympani nerve

*Shusuke Iwata[1], Shinpei Takahashi[1], Toshiaki Yasuo[1], Takeshi Suwabe[1], Noritaka Sako[1], Yuzo Ninomiya[2,3] ([1]*Dept. Oral Physiol. Asahi Univ. Sch.,* [2]*Dept. of Oral Physiol., Grad. Sch. of Med., Dent., and Pharm. Sci., Okayama Univ.,* [3]*Monell Chemical Senses Center*)

There are several reports investigating potential effects of capsaicin (CAP) on taste responsiveness, but results have reported so far are controversial. For example, CAP had no effect on, modified, or elicited particular taste(s) in humans and rodents. Concentrations of CAP (30-500μM) used in most of those studies, however, are known to cause desensitization (reduction of responsiveness to repeated stimulations), thereby potentially irreproducible results. In this study, to avoid the CAP desensitization, we used lower concentrations of CAP (0.1-10 μM) and examined potential effects of CAP on taste responses of the mouse chorda tympani nerve to various tastants. The results showed that CAP alone elicited no taste nerve responses. Intriguingly, addition of CAP enhanced responses to mono- and di-saccharide sugars and NaCl but not to artificial sweeteners and other bitter, sour, salty and umami compounds. Moreover, sodium glucose co-transporter (SGLT) 1 inhibitor abolished enhancing effects of CAP to responses to sugars. Taken together, these results suggest that CAP enhances gustatory responses to sugars via SGLT1 independently of the T1Rs-dependent sweet taste pathway which is broadly sensitive to both sugars and artificial sweeteners.

### [1P-093]

### Sex differences related to changes in the threshold for thermal stimulation of medullary dorsal horn neurons in lingual neuropathy model animals

*Yoshiyuki Tsuboi[1], Kaizu Akihiro[1] ([1]*Department of Physiology, Nihon University School of Dentistry*)

We investigated the effects of minocycline and pioglitazone on mechanical and thermal stimulation of the tongue after lingual nerve compression (LNI) and a reduction in the head escape reflex threshold (HWRT). HWRT reduction was observed only in male mice after in-tank minocycline administration and mechanical or thermal stimulation after LNI. In contrast, after intra-tank pioglitazone administration, the HWRT decline was suppressed only by mechanical or thermal stimulation after LNI. However, sex differences in the response to thermal stimulation at the medullary neuronal level are unknown. Therefore, we aimed to record the effect of changes in threshold on thermal stimulation of medullary neurons by minocycline and pioglitazone administration and to investigate sex differences at the neuronal level.
After exposing the lingual nerve in male and female mice, LNI was performed by compressing the lingual nerve for 30 s at 30 g. On day 3 after LNI, we recorded single neuronal activities following heat stimulation to the tip of the tongue and measured the neuronal threshold for stimulation 0.5 and 1 h after minocycline, a microglial activation inhibitor, or pioglitazone, an agonist of peroxisome proliferator-activated receptor γ expressed in T cells to the medullary dorsal horn.
In both males and females, the firing threshold of neurons for thermal stimulation of the tongue was significantly reduced after LNI. The application of minocycline in males and pioglitazone in females significantly increased neuronal thresholds for heat stimulation. These results suggest that sex differences in tongue neuropathic pain after lingual nerve injury involve differences in immune cells expressed in the central nervous system and regulation of the ascending nociceptive pathway.

### [1P-094]

### Changes in stimulus responsiveness of newly formed and old secretory granulesin the parotid gland over time

*Miyuki Toda[1], Megumi Yokoyama[1], Osamu Kato[1], Junko Yoshigaki[1] ([1]*Department of Physiology, Nihon University School of Dentistry at Matsudo,*)

[Objective] Salivary gland acinar cells synthesize salivary proteins and store them in secretory granules. Proteins in the secretory granules are secreted upon stimulation, but when secretion is continuously inhibited, the secretory granules accumulate intracellularly. Prolonged intracellular storage of secretory granules can cause tissue damages. To maintain tissue homeostasis, aged secretory granules must be properly disposed of. In this study, we analyzed the stimulus-responsiveness of secretory granules over time in order to clarify the processing mechanism of secretory granules. [Method] We prepared an expression vector for cystatin D-fused HaloTag protein (Cst5-Halo), which binds HaloTag fluorescent ligands. Primary culture of parotid acinar cells expressed Cst5-Halo were labeled with the HaloTag TMR ligands. After washout of excess ligands, the cells were cultured for 3 and 6 h. Then, the cells were incubated in the absence or presence of isoproterenol (Iso) and the cells and medium were harvested. The cell lysates and medium were labeled with HaloTag AlexaFluor 660 (AF660) ligand, electrophoresed, and transferred to PVDF membranes. By measurement of TMR and AF660 signals of Cst5-Halo retained in the cells and secreted to the medium, the percentage of secreted Cst5-Halo was calculated. [Results and Discussion] When stimulated with Iso at 3 h after TMR labeling, the secretion of AF660-labeled proteins was significantly higher than those labeled with TMR. In contrast, at 6 hours, the secretion of AF660-labeled proteins declined and there was no significant difference between AF660- and TMR-labeled proteins. These results suggest that newly formed granules have a higher sensitivity to stimulants than older ones and their response declined over time. [Conflict of Interest] I do not have a conflict of interest.

### [1P-095]

### Taste sensory evaluation of sodium with thickeners by Time-intensity method

*Chikayo Maeda[2], Shinpei Takahashi[1], Toshiaki Yasuo[1], Takeshi Suwabe[1], Yoko Iwase[2], Keika Gen[2], Nobuyuki Sakai[3], Noritaka Sako[1] ([1]*Dept. Oral Physiol., Asahi Univ. Sch. Dent., Gifu, Japan,* [2]*Dept. Dent. for Disability & Oral Health, Asahi Univ. Sch. Dent., Gifu, Japan,* [3]*Dept. Psych., Tohoku Univ. Sch. Arts & Letters, Sendai, Japan*)

In the clinical situation, viscous foods mixed with thickeners are served to patients with dysphagia. The patients who eat the foods with the thickeners often complain of distortion of the taste. Our previous electrophysiological and behavioral studies in rats have shown that the taste nerve responses and preferences for the gustatory stimuli when they are mixed with some thickeners. We found that taste nerve responses to NaCl solution were suppressed when it was mixed with some thickeners. And preference to the NaCl solution with thickeners was also suppressed. In this study, therefore, we investigated whether similar effects was observed in humans by using Time-intensity (TI) method and visual analogue scale (VAS) method. We used 0.8% NaCl solution mixed with three types (A, B and C) of commercial thickeners as test stimuli. The concentration of the thickeners was equivalent to "Extremely thick" by referring to the "Society of Eating and Swallowing Rehabilitation Classification 2021 (Thickening) Quick Reference Table." In TI method, subjects took 0.8% NaCl solution with and without thickeners into their mouth and tasted it for 10 sec each and were asked to continuously evaluate the saltiness intensity for 80 sec with the software. In VAS evaluation, subjects were asked to taste and evaluate various NaCl solutions with different saltiness concentrations and with various thickeners. This study was approved by the Ethics Committee of Asahi University, Japan (Approval No. 3204). The results showed that subjects evaluated the salt intensity less salty to the NaCl solution with the thickeners than without them. This result could be found in the evaluation with thickener A and B, but not for thickener C. This result shows that the patients eat the foods with thickeners feel salty taste weaker than the foods without thickeners and suggests that this effect is supported by the similar mechanism as shown in our previous animal experiments.

### [1P-096]

### Modification of metallic taste in postmenopausal osteoporosis model mice

*Yuko Kawabata[1], Shingo Takai[1,2], Shusuke Iwata[3], Keisuke Sanematsu[1,4,5], Fuminori Kawabata[6], Takashi Kanematsu[7], Eijiro Jimi[5,8], Noriatsu Shigemura[1,4] ([1]*Dept Oral Neurosci, Kyushu Univ Grad Sch Dent,* [2]*DDR Cent, Kyushu Univ Grad Sch Dent,* [3]*Dept Oral Physiol, Asahi Univ Sch Dent,* [4]*R&D Cent for Five-Sense Devices, Kyushu Univ Grad Sch Dent,* [5]*OBT Res Cent, Kyushu Univ Grad Sch Dent,* [6]*Fac Agric Life Sci, Hirosaki Univ,* [7]*Dept Cell Biol Aging Sci & Pharmacol, Kyushu Univ Grad Sch Dent,* [8]*Lab Mol Cell Biochem, Kyushu Univ Grad Sch Dent*)

Postmenopausal women have significant bone loss and the changing taste preferences caused by altered systemic homeostasis associated with estrogen deficiency. However, the molecular mechanisms behind the taste changes remain unclear. Taste plays an important role in regulating feeding behavior that reflects physiological nutritional and mineral demands. It is presumed that abnormal bone calcium metabolism connected with menopause leads to modulation of mineral or ionic sensing in the peripheral taste organs. In this study, we analyzed the gene expression in taste buds and taste preference using the mice with osteoporosis induced by ovariectomy. Female C57BL/6J mice were either sham-operated or ovariectomized (OVX) at 8 weeks of age and analyzed at 8 weeks after operation. First, we conducted comprehensive analysis of RNA sequencing (RNA-seq) gene expression profiling of total RNA isolated from taste buds of the circumvallate papillae in the sham-operated and OVX group. In the OVX group, multiple genes related to metal ion binding and calcium channel activity were upregulated, compared to the sham-operated mice. Next, to look at the changes in taste behavior responses, the number of 5-second licks to various taste solutions was measured. As a result, the aversive responses to calcium chloride ($CaCl_2$) and magnesium chloride ($MgCl_2$) (metallic taste) in the OVX group were significantly enhanced compared to those of the sham group. These results suggest that the postmenopausal osteoporosis may affect the expression of genes related to mineral receptivity in the peripheral taste organs and enhance the aversive response to divalent metal salts. The abnormal bone calcium metabolism associated with menopause may alter the mineral receptive mechanisms in the taste organs, resulting in modified perception of metallic taste. Postmenopausal women may become increasingly averse to metallic taste, leading to deficient calcium intake, which can form the vicious cycle that leads to further bone loss.

### Poster

### [1P]
### Circulation

March 28, 13:00 - 14:20, Poster Room

### [1P-097]

### ATF3 facilitates PVAT anticontractile activity and vasorelaxation by governing adipocyte-derived factor HDL-bound S1P release

*Yu Han Su[1,2], Hsiao Fen Li[4], Po Yi Chen[1,2], Heng Lin[4], Tzu Ling Tseng[1,2,3] ([1]*Hualien Tzu Chi Hospital CardioVascular Research Center*, [2]*Department of Medical Research, Hualien Tzu Chi Hospital*, [3]*Tzu Chi University of Science and Technology*, [4]*Department of Physiology, School of Medicine, College of Medicine, Taipei Medical University*)

**BACKGROUND AND PURPOSE** A critical role of the perivascular adipose tissues (PVATs) is to modulate vascular homeostasis and protect against the blood pressure dysregulation caused by cardiovascular dysfunction. We demonstrate that the activating transcription factor (ATF)-3 gene in PVATs is crucial for decreasing vascular wall tension abnormalities; however, the mechanism through which this protection is achieved remains unclear. We hypothesise that ATF3 regulates the biosynthesis of PVAT-derived relaxing factors (PVDRFs), and its secretion contributes to vasorelaxation. **EXPERIMENTAL APPROACH** In this study, we employed multifaceted approaches to resolving these concerns, including an in vivo animal model using global ATF3-deficient mice, in vitro blood vessel myography, and biochemical analyses to quantify ATF3-mediated PVDRFs release and reactivity in the vasculature. **KEY RESULTS** We found that wild-type (WT) thoracic aortic PVAT extracts significantly induced the resting-tone dilation and attenuated the contractile responses induced by vasoconstrictors compared with those of $ATF3^{-/-}$ mice. The heat-stable PVAT extracts from the WT mice caused sustained, reproducible vasodilation without tachyphylaxis in the control aortic rings of the mice. The results of the biochemical evaluation of PVDRF release revealed that the $ATF3^{-/-}$ mice had lower sphingosine 1-phosphate (S1P) and HDL-C levels than the WT mice. Furthermore, the PVAT extracts from the WT mice induced long-lasting vasorelaxation, which was substantially blocked by sphingosine-1-phosphate receptor 3 antagonist TY52156 and scavenger receptor group B type 1 receptor antagonist glyburide (10 μM). **CONCLUSIONS AND IMPLICATIONS** We suggest that ATF3 within PVAT can modulate vascular functioning by strengthening the sphk1–S1P–s1pr3 lipid axis and stimulating the S1P bound to HDL form vasodilator HDL–S1P. ATF3 is an essential modulator in maintaining PVAT physiological function, providing a novel target for treating obesity-related cardiovascular diseases and an avenue for clinical intervention.

### [1P-098]

### Roles of endothelial prostaglandin $I_2$ in maintaining synchronous spontaneous $Ca^{2+}$ transients in rectal capillary pericytes

*Retsu Mitsui[1], Kyoko Miwa-Nishimura[1], Hikaru Hashitani[1] ([1]*Nagoya City University*)

Capillary pericytes in hollow visceral organs act as pacemaker cells for spontaneous vasomotion. Thus, they periodically generate synchronous spontaneous $Ca^{2+}$ transients to drive the upstream vascular segments. Here, we further explored mechanisms underlying the synchrony of spontaneous $Ca^{2+}$ transients. Intracellular $Ca^{2+}$ dynamics of capillary pericytes were visualised using mucosa-submucosa preparations of the rectum of NG2-GCaMP mice. Spontaneous $Ca^{2+}$ transients in rectal capillary pericytes that propagated to the upstream arterioles were disrupted or prevented by the gap junction blocker carbenoxolone (3 μM). The spontaneous $Ca^{2+}$ transients were diminished by the inhibitor of endoplasmic reticulum (ER) $Ca^{2+}$-ATPase, $IP_3$ receptor, $G\alpha_{q/11}$ (1 μM YM-254890) or Ani9 (3 μM), a TMEM16A (ANO1) $Ca^{2+}$-activated $Cl^-$ channel inhibitor, while nifedipine was ineffective. Consistently, capillary pericytes expressed TMEM16A-immunoreactivity. The synchrony of spontaneous $Ca^{2+}$ transients were disrupted by cyclooxygenase (COX) inhibitors known as non-steroidal anti-inflammatory drugs (NSAIDs, 1 μM indomethacin or 10 μM diclofenac), COX-2 inhibitor NS 398 (10 μM) or prostaglandin $I_2$ ($PGI_2$) receptor antagonist RO1138452 (1 μM) with a rise in the basal $Ca^{2+}$ level. Subsequent levcromakalim (100 nM), a $K_{ATP}$ channel opener, restored the synchrony and reversed the raised $Ca^{2+}$ level. COX-2 immunoreactivity was detected in the endothelium but not pericytes. In indomethacin-treated pericytes, $PGI_2$ (100 nM) restored the synchrony and reversed the $Ca^{2+}$ level in a manner sensitive to glibenclamide (10 μM), a $K_{ATP}$ channel blocker. In conclusion, spontaneous ER $Ca^{2+}$ release in the rectal capillary pericytes opens TMEM16A to depolarise their membrane. The resultant depolarisations sequentially spread to their neighbours via gap junctions, in which ER $Ca^{2+}$ release was triggered by voltage-dependent $IP_3$ production. The synchrony of pericyte $Ca^{2+}$ transients appears to depend on their relatively hyperpolarised membrane resulting from the opening of $K_{ATP}$ channel by COX-2-mediated, constitutive endothelial $PGI_2$ release. These mechanisms may explain the well-known adverse effects of NSAIDs that are attributable to the reduction in mucosal blood flow in the gastrointestinal tract.

### [1P-099]

### Disruption of the nonneuronal-cardiac cholinergic system in cardiomyocytes causes cardiac dysfunction associated with systemic inflammation

*Takashi Sonobe[1], Masayuki Tsuda[2], Yuko Kai[1], Shino Oikawa[1], Asuka Mano[1], Yoshihiko Kakinuma[1] ([1]*Nippon Medical School*, [2]*Kochi University*)

Previously we reported that the non-neuronal cardiac cholinergic system (NNCCS) plays a pivotal role in sustaining a variety of cardiac functions, including glucose metabolism, gap junction, cell survival signaling, resilience to hypoxia or ischemia, using a murine heart-specific gain of NNCCS function model. However, a detailed report regarding the cardiovascular system in the loss of NNCCS function model have never been performed. The loss of function model of NNCCS, i.e., tamoxifen-induced Cre-mediated deletion of choline acetyltransferase gene in the heart, revealed a decline of cardiac function within 2 weeks after induction. Some of deleted mice demonstrated typical phenotypes of heart failure, and the mortality rate reached to more than 30%. The molecular makers related to metabolism was downregulated and ATP production in the heart was also blunted. Along with the heart failure-associated phenotypes, the deleted mice showed systemic inflammatory responses and an extra-cardiac organ, spleen, also showed elevation of cytokine expression. These data indicate that downregulation of NNCCS not only influences cardiac functions but also induces systemic inflammation, suggesting that the function of NNCCS regulates the heart and furthermore negatively regulates inflammatory responses.

### [1P-100]

### TRPV2 is crucial for the maturation of Ca2+ handling in neonatal cardiomyocyte

*YUBING DONG[1], GUOHAO WANG[1], Yanzhu Chen[1], Kimiaki Katanosaka[3], Keiji Naruse[1], Yuki Katanosaka[2] (*[1]Department of Cardiovascular Physiology, Graduate School of Medicine, Dentistry and Pharmaceutical Sciences, Okayama University, [2]College of Pharmacy, Kinjo Gakuin University, [3]College of Life and Health Sciences, Chubu University*)

Aims: We examined the role of the transient receptor potential, vanilloid family type 2 (TRPV2) in the maturation of $Ca^{2+}$ handling in cardiomyocytes using a culture system of neonatal mouse cardiomyocytes. Methods and Results: In cultured newborn cardiomyocytes isolated from floxed-TRPV2 mice, we followed the formation of intercalated discs, the reorganization of myofibrils and change in $Ca^{2+}$ handling with time in myocytes. These cardiomyocytes increase $Ca^{2+}$ oscillation and caffeine-induced $Ca^{2+}$ release, which reflects $Ca^{2+}$ accumulation in the sarcoplasmic reticulum (SR), over the course of culture time. In this experimental system, overexpression of Cre-recombinase by adenovirus not only markedly decreased TRPV2 expression in cardiomyocytes, but also inhibited NCX1 expression, which is specifically expressed in cardiomyocytes. These TRPV2-deficient cells showed no increase in $Ca^{2+}$ oscillation or $Ca^{2+}$ accumulation of SR after 24 hours in culture. In addition, the development of F-actin is severely inhibited, and there is no localization of Cx43 to the intercalated disc. On the other hand, overexpression of TRPV2 markedly promoted $Ca^{2+}$ oscillation and increased SR $Ca^{2+}$ accumulation, and F-actin development was also prominent. Synchronized beating was also observed in these cells. In cultured cell systems, nuclear translocation of MEF2 was also dependent on TRPV2 expression. Conclusions: These results suggest that TRPV2 is crucial for the maturation of $Ca^{2+}$ handling and characterization in cardiomyocytes. Our results will provide knowledge for understanding cardiomyocyte maturation and for future applications in regenerative medicine.

### [1P-101]

### Moku-boi-to's Defensive Mechanism Against ET-1-Induced Cardiac Damage

*Hideaki Tagashira[1], Fumiha Abe[1], Tomohiro Numata[1] (*[1]Akita Univ.*)

In recent years, Japanese herbal medicine has gained significant attention for its perceived safety and increasing user popularity. However, the medical community tends to avoid its use due to the need for more scientific evidence supporting its efficacy. Recently, we highlighted the protective effects of Moku-boi-to (MBT) against angiotensin II-induced hypertrophy. However, its potential to mitigate damage from other cardiac injury-inducing factors remains unclear. This study aims to explore the impact and mechanism of MBT on potential cardioprotective effects against endothelin-1 (ET-1)-induced neonatal rat ventricular myocyte (NRVM) hypertrophy and cell death. Additionally, it delves into exploring novel therapeutic strategies, aiming to contribute to development. After administering 100 nM ET-1 to NRVM, the cell cross-sectional area gradually increased, peaking 48 hours later, resulting in hypertrophic cardiomyocytes approximately twice the size of the control. We observed that MBT administration suppressed this ET-1-induced hypertrophy in a dose-dependent manner, with an $IC_{50}$ of 417.1 μg/ml. Notably, the increase in mRNA expression of cardiac hypertrophy markers *ANP, BNP, b-MHC*, and *RCAN1* at 48 hours was significantly suppressed by MBT. Additionally, MTT and LDH release assays indicated reduced cytotoxicity during cardiac hypertrophy. Further analysis revealed that MBT effectively mitigated the increase in intracellular $Ca^{2+}$ induced by ET-1. Moreover, MBT treatment improved mitochondrial morphology abnormalities, reduced ATP levels, and mitigated increased ROS production caused by ET-1. MBT treatment also significantly affected cardiac hypertrophy and dysfunction in an isoproterenol-induced heart failure mouse model. These findings suggest that MBT can protect against ET-1-induced cardiac damage by improving mitochondrial dysfunction. The study offers fresh evidence that MBT holds promise as a safe and effective preventive agent against cardiac hypertrophy and cell death.

### [1P-102]

### Loss of endothelial cell-derived acetylcholine production causes elevated systemic blood pressure and impaired exercise capacity in mice

*Takashi Sonobe[1], Yoshihiko Kakinuma[1] (*[1]Department of Bioregulatory Science, Nippon Medical School*)

Vascular endothelial cells produce acetylcholine (ACh). The endothelium-derived ACh is suggested to be a key modulator of endothelial cell functions by an autocrine-like mechanism that regulates an arterial tonus. We hypothesized that loss of the endogenous production of ACh in endothelial cells impairs basal regulation of arterial tonus and thus, impairs whole-body endurance exercise capacity, which relies on adequate blood supply to the active skeletal muscle. To test this hypothesis, we used endothelial cell-specific knockout mice of choline acetyltransferase (ChAT), an enzyme essential for the production of ACh. *VEcad-CreERT2+ChATflox/flox*; eChAT-KO mice were developed. At 7 days after tamoxifen/vehicle treatment, systemic blood pressure was measured by a noninvasive tail-cuff blood pressure monitoring system. The mice then underwent an exercise tolerance test on a rodent treadmill with 5-degree incline. Tamoxifen-treated eChAT-KO mice indicated higher systolic, mean, and diastolic blood pressure compared to vehicle-treated/control mice. The eChAT-KO mice also indicated lower total work rate during the exercise tolerance test than the control mice. These results suggest that the endothelium derived ACh regulates basal peripheral arterial tonus via endothelial dependent mechanisms.
The endothelium derived ACh, also known as the non-neuronal cholinergic system in the endothelial cells may be a potential therapeutic target for endothelial dysfunction leading to cardiovascular disease. COI: No

### [1P-103]

### Identification of atypically-shaped cardiomyocytes (ACMs) in ANP promoter-driven AcGFP-expressing mice

*Mariko Omatsu-Kanbe[1,6], Ryo Fukunaga[1], Kakeru Shimoda[2], Akiyuki Nishimura[2], Masakazu Agetsuma[3,2], Hiroki Satooka[4], Makio Higuchi[4], Junichi Nabekura[2], Motohiro Nishida[2,5], Takako Hirata[4] (*[1]Dept. Physiology, Shiga University of Medical Science, [2]National Institute of Physiology, [3]Institute for Quantum Life Science, [4]Dept. Fundamental Biosciences, Shiga University of Medical Science, [5]Kyushu University, [6]Research Strategy Promotion Office, Shiga University of Medical Science*)

The functional mammalian heart comprises heterogeneous cell lineages, including cardiomyocytes, vascular smooth muscle cells, fibroblasts and cardiac stem or progenitor cells. We investigated the functions and characteristics of atypically-shaped cardiomyocytes (ACMs), identified as spontaneously beating cells in the cultures of interstitial cell fractions obtained from adult mouse heart. Although ACMs originate from cardiac ventricles, those express proteins specific for both atrial and ventricular myocytes, SA nodal cells and fetal heart cells, thus suggesting that these cells are likely to be cardiac progenitors or immature cardiomyocytes. We have sought the possible markers for ACMs to clarify the localization and role in the heart. ACMs in the culture are characterized by the abundant expression of atrial natriuretic peptide (ANP) usually absent in the ventricular myocytes. In this study, we prepared ANP promoter-driven AcGFP-expressing mice generated by infection of adeno-associated virus (AAV2-9) encoding pANP-AcGFP and successfully identified ACMs in the interstitial spaces among ventricular myocytes.

### [1P-104]

### Augmented store-operated $Ca^{2+}$ entry in senescent vascular endothelial cells

*Mayumi Hirano[1], Katsuya Hirano[1] ([1]*Department of Cardiovascular Physiology, Faculty of Medicine,Kagawa University*)

**Background**: Store-operated $Ca^{2+}$ entry (SOCE) plays a critical role in generation of intracellular $Ca^{2+}$ signal in vascular endothelial cells, thereby regulating endothelial barrier. Endothelial barrier disruption is associated with age-related vascular diseases, such as atherosclerosis and hypertension. The present study investigated the effect of aging on the SOCE by inducing replicative cell senescence in cultured porcine aortic endothelial cells (PAEC).
**Methods and Main findings**: In younger PAEC of lower numbers of replication (passages 9-15) and at confluence (day 7), 1 U/mL thrombin decreased trans-endothelial electrical resistance and induced di-phosphorylation of 20 kDa myosin light chain and actin bundle formation at cell periphery as early events seen 3 min after stimulation. Later, the peripheral actin bundles were reorganized to stress fiber after 15 min of thrombin stimulation. In younger PAEC, but at early culture days, when the cell-cell contact remains immature, thrombin directly induced actin stress fiber formation as early events. In aged PAEC of higher number of replication (passage 23-30), which exhibited high level of the senescence-associated β-galactosidase activity, actin stress fibers were observed at the resting state without any stimulation, and thrombin stimulation further augmented actin stress fiber formation. SOCE was investigated by using thapsigargin, an inhibitor of endoplasmic $Ca^{2+}$ ATPase and fura-2 fluorometry. Thapsigargin (1 μM) induced a transient elevation of cytosolic $Ca^{2+}$ concentrations ($[Ca^{2+}]_i$) in the absence of the extracellular $Ca^{2+}$, presumably due to release from the intracellular $Ca^{2+}$ stores in younger PAEC, while subsequent replenishment of 2 mM extracellular $Ca^{2+}$ induced a sustained $[Ca^{2+}]_i$ elevation, presumably due to SOCE. The similar effect of thapsigargin was seen in the aged PAEC; however, the level of SOCE seen in the aged PAEC was significantly higher than that seen in younger PAEC.
**Conclusions**: The integrity of the cell-cell contact were impaired by replicative senescence. Augmented SOCE in aged endothelical cells might contribute to impairment of cell-cell contact.

### [1P-105]

### Bioinformatic Identification of Potential RNA Alterations on the Atrial Fibrillation Remodeling from Human Pulmonary Veins

*Wataru Igarashi[1], Daichi Takagi[1], Okada Daigo[2], Daiki Kobayashi[3], Miho Oka[4], Toshiro Io[4], Kyoichi Ono[3], Hiroshi Yamamoto[1], Yosuke Okamoto[3] ([1]*Department of Cardiovascular Surgery, Akita Graduate School of Medicine,* [2]*Center for Genomic Medicine, Graduate School of Medicine, Kyoto University,* [3]*Department of Cell Physiology, Akita Graduate School of Medicine,* [4]*Research department, Ono pharmaceutical CO., LTD.*)

[Backgrounds] Atrial fibrillation (AF) is the most frequent persistent arrhythmia and requires clarification of etiology. Each of the genetic backgrounds of AF patients identified from GWAS analysis are mostly not causative genes with low relative risk. Most transcriptome analyses of AF are limited to the atrial samples on the assumption that pathological RNA alterations occur in the atrium.[Material and methods] We analyzed the expression levels of protein-coding and long noncoding RNAs (lncRNAs) in six cardiac regions by RNA-seq. Totally, 23 AF and 23 non-AF samples were analyzed, which were collected from the left atrium, right atrium, sinus node, left ventricle, right ventricle, and pulmonary vein (PV). [Results] In the Differential Expression Genes (DEGs) analysis, of the six cardiac regions, gene expression was most affected by the presence of non-paroxysmal AF in the PV, not the atrium. Gene ontology analysis identified significant DEGs only in PVs. Among them, ion channel-related genes were enriched. Co-expression network analysis for DEGs identified cancer-associated lncRNAs such as SAMSSON and FOXCUT and a cancer-associated transcription factor called FOXC1 in the up-regulated gene cluster. Reportedly, FOXC1 cooperates with FOXCUT to promote the epithelial–mesenchymal transition (EMT).[Conclusions] (1) PV was the region with the most intensive gene expression alterations by AF incidence. (2) The AF remodeling can be attributable to a post-transcriptional genetic regulation similar to carcinogenesis such as EMT.

### [1P-106]

### Comparison of Smooth Muscle Regulatory Protein CPI-17 Expression in Rat Renal Arterioles.

*Kosuke TAKEYA[1], Hyebeen Kim[1], Yoshinori Tanaka[1], Masumi Eto[1] ([1]*Okayama Univ. Sci.*)

BACKGROUND: The resistance vessels responsible for regulating kidney function, including the interlobular artery and the afferent and efferent arterioles, exhibit significant variations in how they control blood flow. Understanding these differences is crucial for comprehending how the renal blood flow is regulated and how diseases can impact these processes. For example, in response to stimulation with angiotensin II, quicker contraction occurred in the afferent arterioles compared to the efferent arterioles. In the prior study, we reported distinct phosphorylation signaling patterns between the afferent and efferent arterioles, but the underlying molecular mechanisms remain unclear. The extent of myosin light chain phosphorylation in arteries is defined through the balance between kinase and phosphatase activities. The myosin light chain phosphatase activity is regulated by an endogenous inhibitor protein, such as CPI-17. In this study, we tested whether CPI-17 is involved in the differential functions in renal arterioles.
METHODS: A rat left kidney was perfused with DMEM containing 1.5% agarose via the renal artery and then excised. After cooling and solidifying the agarose, kidney slices were digested with collagenase and dispersed vessels in DMEM. Renal arterioles were meticulously isolated under a microscope and utilized for measurements of myosin light chain phosphorylation, RT-qPCR, and immunostaining.
RESULTS AND CONCLUSION: Angiotensin II stimulation induced both mono-phosphorylation (at Ser19) and di-phosphorylation (at Thr18 and Ser19) of the myosin regulatory light chain in the efferent arterioles, while only mono-phosphorylation was observed in the afferent arterioles, suggesting distinct phosphorylation signaling mechanisms in these vessels. RT-qPCR demonstrated higher levels of MYPT1 mRNA in the afferent arterioles, and immunostaining showed increased expression of CPI-17 proteins in the afferent arterioles. These findings indicate that CPI-17 likely plays a more significant role in phosphorylation regulation in the renal afferent arterioles compared to the efferent arterioles.
COI: properly declared

### Poster

### [1P]
### Reproduction

March 28, 13:00 - 14:20, Poster Room

### [1P-107]

### Functional coupling between mechanosensitive channels and IK channels in the regulation of oviduct contraction

*Ayako Sakai[1], Kaori Sato-Numata[1], Masami Yoshino[2], Tomohiro Numata[1] ([1]*Akita Univ.*, [2]*Tokyo Gakugei Univ.*)

The synchronized regulation of ion channels in response to local mechanical stimuli is critical for controlling various physiological processes. This process includes linking muscle cell excitation to contraction and regulating rhythmic oviduct activity. This study focused on the intricate interplay between stretch-activated (SA) cation channels and intermediate conductance $Ca^{2+}$-activated potassium (IK) channels in cricket oviduct myocytes. The cell membrane deformation caused by muscle contraction activates SA channels, leading to a subsequent influx of $Ca^{2+}$. This influx, then, triggers the activation of spatially localized IK channels, thereby fine-tuning spontaneous muscle contraction. To characterize endogenously expressed IK channels and evaluate the functional relevance of the extracellular $Ca^{2+}$ sources that activate these channels, we conducted patch clamp experiments on cricket oviduct myocytes. Our initial investigation aimed to differentiate IK channels from big conductance $Ca^{2+}$-activated potassium (BK) channels by adding extracellular $Ba^{2+}$. The single-channel conductance of the identified IK channels was measured at 62 pS. These channels exhibited activity correlated with intracellular $Ca^{2+}$ concentration but did not display voltage dependence, confirming their endogenous expression in cricket oviduct myocytes. Subsequently, we explored the $Ca^{2+}$ influx pathway responsible for activating IK channels. The absence of extracellular $Ca^{2+}$ or the presence of $Gd^{3+}$ resulted in the suppression of IK channel activity. Furthermore, $Gd^{3+}$ loading inhibited the increase in intracellular $Ca^{2+}$ induced by shear stress. Finally, we investigated the spatial proximity between SA and IK channels. Manipulations such as extracellular $Ca^{2+}$ removal, localized $Ca^{2+}$ administration via a pipette, and membrane stretching stimuli increased SA channel activity, subsequently triggering IK channel activity. Membrane stretch-induced activation of SA and IK channels exhibited a positive correlation. Notably, the appearance of IK channel activity and its response to mechanical membrane stretching was contingent on the presence of $Ca^{2+}$ in the pipette. In summary, our findings provide strong evidence for the endogenous expression of IK channels in cricket oviduct myocytes and highlight their regulation by adjacent SA channel activity. This functional coupling between SA and IK channels may serve as the molecular foundation for spontaneous rhythmic contractions in these cells.

### [1P-108]

### Roles of PDGFRα positive cells and TMEM16A in developing spontaneous contractions of the rat caudal epididymis

*Wataru Kudo[1], Retsu Mitsui[1], Hikaru Hashitani[1] ([1]*Department of cell physiology, Nagoya city University Graduate school of medicine*)

The epididymis is finely coiled tubular structure where sperm undergo maturation and storage prior to ejaculation. We have demonstrated that that the epididymal duct of proximal cauda exhibit spontaneous phasic contractions (SPCs) relying on spontaneous $Ca^{2+}$ release from internal stores that triggers the opening of $Ca^{2+}$-activated $Cl^-$ channel (CaCCs). Here, we further investigated mechanisms underlying SPCs focusing on TMEM16A, a CaCC, and also aimed to identify pacemaker cells for SPCs.Perforated whole-cell patch clamp technique was applied to enzymatically-isolated epididymal cells from male rats (6-8 weeks). SPCs of caudal epididymal duct were recorded by measuring the distance between the two threads tied around the both edges of duct segment with edge-tracking software (Diamtrak). The localization of TMEM16A and platelet-derived growth factor receptor α (PDGFRα) positive cells in the caudal epididymal duct was examined by immunohistochemistry.Approximately one-third of isolated epididymis cells exhibited spontaneous transient inward current (STICs) at the holding potential -60 mV. The reversal potential for STICs was close to the calculated chloride equivalent potential depending on extracellular $Cl^-$ concentrations. Ani9, the TMEM16A specific inhibitor (3 µM), decreased both amplitude and frequency of STICs. The sarco/endoplasmic reticulum $Ca^{2+}$ - ATPase (SERCA) inhibitor, cyclopiazonic acid (CPA) (30 µM) abolished STICs. Ani9 (3 or 10 µM) reduced the frequency of SPCs without changing their amplitude. Immunohistochemistry revealed that cells located in the innermost smooth muscle layer coexpressing α-smooth muscle actin (α-SMA) and PDGFRα also express TMEM16A.   These results suggested that PDGFRα(+)α-SMA(+) cells located in the innermost smooth muscle layer may act as pacemaker cells to drive SPCs by the mean of TMEM16A. STICs appear to arise from spontaneous $Ca^{2+}$ release and subsequent opening of TMEM16A, and resultant depolarizations spread to the outer smooth muscle layer to develop SPCs.

### [1P-109]

### Excluding poor quality embryos using membrane potential measurement: an application to mouse early embryo selection

*Masao MIYAKE[1], Susumu YOSHIE[1], Satoru KANEKO[2], Akihiro HAZAMA[1] ([1]*Department of Cellular and Integrative Physiology, Fukushima Medical University School of Medicine*, [2]*Ichikawa General Hospital, Tokyo Dental College*)

Morphological inspection is the most commonly used technique to pick quality oocytes and embryos for artificial fertilization. To raise reproductive ratio, a new selection method from a new point of view is needed. The membrane potential reflects expression of ion channels and completeness of cell membrane, it may evaluate ovum quality. We previously showed that there was a wide dispersion of membrane potential among eggs without morphological difference. It implied this technique could be applied for quality selection. In this study, we analyzed the relationships between embryogenic outcome and membrane potential of mouse embryos after freeze-thaw cycle. Single-cell, two-cell and four-cell embryos were applied to the freeze-thaw cycle, and measured membrane potential. Some embryos performed good morphological characteristics, and could reach blastocysts. But most embryos which performed near zero voltage stopped development. The near zero voltage embryos are possible to be scratched during conventional protocol. This method may be applicable to exclude damaged embryos. All experiments were planned toward institutional guidelines and reviewed by institutional animal care and use committee.(COI:NO)

### Poster

### [1P]
### Endocrine

March 28, 13:00 - 14:20, Poster Room

### [1P-110]

#### Effects of oxytocin in breast milk on nurturing behavior in offspring

*Yusuke Watanabe[1], Itsuki Takahashi[1], Kaoru Uchida[1], Wataru Miyazaki[2], Noriaki Shimokawa[1,3] ([1]*Department of Nutrition, Takasaki University Graduate School of Health and Welfare,* [2]*Department of Bioscience and Laboratory Medicine, Hirosaki University Graduate School of Health Sciences,* [3]*Department of Integrative Physiology, Gunma University Graduate School of Medicine*)

Breastfeeding is a core component of nurturing behaviors, and breast milk is essential for adequate nutrition and growth of newborns. We are looking for a factor in the mother's milk that regulates the expression of nurturing behavior in the next generation. Oxytocin (OXT), a posterior pituitary hormone, induces uterine contractions during parturition, and milk ejection during lactation. It has also been shown to be involved in reproductive and nurturing behaviors. We previously confirmed that OXT is present in mouse milk and revealed changes in OXT levels during lactation. To understand the effect of OXT in breast milk on nurturing behavior in the next generation, low-OXT content breast milk was prepared by removing OXT using an OXT antibody. Female mice artificially raised with low-OXT breast milk matured normally and produced their own pups; however, nurturing behavior was strongly suppressed. Interestingly, when OXT was added to low-OXT breast milk, the resulting pups exhibited normal nurturing behavior as adults. These observations indicate that OXT in breast milk may regulate nurturing behaviors in offspring. Further studies are needed to analyze the molecular mechanisms underlying the effects of OXT on nurturing behavior in offspring.

### [1P-111]

#### Behavioral Analysis of Mice with Congenital Hypothyroidism Using Automated Group-based Behavior Recording System

*Fumihiko Maekawa[1], Kazuaki Nakamura[2], Kenichi Kobayashi[3] ([1]*National Institute for Environmental Studies,* [2]*National Research Institute for Child Health and Development,* [3]*National Institute of Occupational Safety and Health*)

Among the chemicals found in the environment, persistent organic pollutants such as dioxin, PCBs, PFOS, PFOA, and brominated flame retardants are known to disrupt the effects of thyroid hormones. Previous studies on thyroid function disrupting effects of these chemicals in animal models have reported the effects on spontaneous activity, cognitive functions, as well as other functions. Our research group has been conducting exposure effect assessment of metals such as arsenic and methylmercury and dioxins using IntelliCage, an automated group-based behavior recording system that can automatically examine effects of chemical exposures and has developed endpoints that can assess the effects. In this research report, we explored how hypothyroidism induces behavioral abnormalities in growth-retarded (*grt*) mice, which develop growth retardation due to congenital thyroid hypoplasia, as a first step in exploring effect assessment endpoints for chemicals that induce such hypothyroidism. *Grt* mice have an amino acid substitution mutation in tyrosylprotein sulfotransferase 2 (*Tpst2*), which transfers a sulfate group to a tyrosine residue of a specific protein and exhibit typical primary hypothyroidism with low thyroid hormone level and elevated thyroid-stimulating hormone (TSH) level. Adult male and female *grt* mice were introduced into the IntelliCage and their basal activity was measured by counting the number of times they entered the four corners of the IntelliCage, which are small rooms with access to water supply bottles. Compared to normal mice in terms of the number of times they entered these rooms per week, an overall trend toward decreased activity was observed in male and female *grt* mice, and the effect was particularly pronounced in female mice. A similar decrease was also observed with respect to nose-poking (pressing the switch to the water bottle) inside the corner, which is necessary to access the water supply. On the other hand, no significant differences were observed in the amount of licking the water bottle. These results are consistent with those reported in a previous study of reduced activity in a mouse model of congenital thyroid hypoplasia, and it is evident that a similar finding can be obtained by using the automated group-based behavior recording system. In the forthcoming study, we plan to apply the same device to search for endpoints to evaluate the effects on cognitive functions.

### [1P-112]

#### The nuclear receptor corepressor, silencing mediator of retinoid and thyroid hormone receptors (SMRT), play unique roles in cerebellar Purkinje cells

*Izuki Amano[1,2], Ayane Ninomiya[1], Kisho Obi[1], Ritter Megan[2], Hollenberg Anthony[2], Noriyuki Koibuchi[1] ([1]*Department of Integrative Physiology Gunma University Graduate School of Medicine,* [2]*Boston University Chobanian & Avedisian School of Medicine Dept of Medicine*)

Thyroid hormone (TH) is essential for brain development. The cerebellum is known to be one of a sensitive brain region for TH. In previous studies, we have focused on the mechanism of TH action in cerebellar Purkinje cells, the only output neurons in the cerebellar cortex. In this study, we focused on silencing mediator of retinoid and thyroid hormone receptors  (known as SMRT or NCoR2), a repressive transcriptional coactivator that regulates gene expression through the TH receptor, to investigate how SMRT affects neurodevelopment in the cerebellum. First, we generated a model in which SMRT is knocked out specifically in cerebellar Purkinje cells by crossing L7/pcp2-Cre mice, which express Cre specifically in cerebellar Purkinje cells, with SMRT-flox mice. No growth or developmental abnormalities were observed in these mice. Subsequently, we performed the rotarod and ladder beam tests to evaluate cooperative movement and motor learning, one of the major cerebellar functions, and found that the mice exhibited mild cooperative movement and motor learning deficits. In addition, we conducted social behavioral tests, which has recently been attracting attention as one of the novel cerebellar functions and found that these mice exhibit mild social behavioral abnormalities. Furthermore, we performed electrophysiological analysis using the slice patch clamp method to characterize the neurophysiology of Purkinje cells caused by SMRT deletion. The results showed an increase in membrane excitability. In addition, while no morphological abnormalities were observed at the macroscopic level, an increase in the number of spines was observed. These results indicate that deletion of SMRT in Purkinje cells causes various cerebellar ataxia symptoms by inducing neurophysiological abnormalities.

### [1P-113]

### Inhibitory effect of vasopressin on oxytocin-induced milk ejection and its mechanism in mice

*Akihiro Kamikawa[1] ([1]*Obihiro University of Agriculture and Veterinary Medicine*)

Oxytocin (OT)-induced milk ejection is the final and essential step in milk secretion. Arginine vasopressin (AVP), responsible for maintaining water and circulatory homeostasis, can bind to and activate the OT receptor due to the molecular similarity of these two neurohypophysial hormones; 7 out of 9 amino acids are identical. Although it was reported approximately six decades ago that AVP inhibits OT-induced milk ejection in rabbits, the underlying mechanisms have yet to be fully elucidated. Therefore, I investigated the effect of AVP on OT-induced milk ejection and its mechanism in a mouse model. Cannulas were inserted into the mammary duct and the jugular vein of anesthetized C57BL/6 mice to record milk ejection and to administer hormones, respectively. First, the effects of AVP on milk ejection were investigated. Intravenous and intraperitoneal administration of AVP induced milk ejection with less potent compared to OT. The AVP-induced milk ejection was inhibited by the OT receptor antagonist, $(d(CH_2)_5^1$, $Tyr(Me)^2$, $Thr^4$, $Orn^8$, des-Gly-$NH_2^9)$-Vasotocin. Second, the interaction between AVP and OT in the milk ejection was examined. Continuous milk ejection by the intraperitoneal administration of OT was paused by the intravenous administration of AVP. Furthermore, intravenous AVP pretreatment inhibited milk ejection induced by subsequent OT administration. These inhibitory effects of AVP were ameliorated by the antagonist of V1a AVP receptor, $[d(CH_2)_5^1$, $Tyr(Me)^2$, $Arg^8]$-Vasopressin, at a concentration where the elevation of blood pressure caused by AVP was fully inhibited. These results suggest that AVP induces milk ejection through OT receptor, but inhibits OT-induced milk ejection possibly through its influence on vascular system and tissue blood flow via V1a receptor. It would be of interest to elucidate the physiological significance of this interaction in the future.

### [1P-114]

### Prolactin axis shift and alterations of placental proteomic profile in late prepartum mice

*Taku James Sairenji[1], Shinnosuke Masuda[1,2], Yuki Fujiwara[1], Noriaki Shimokawa[1,3], Noriyuki Koibuchi[1] ([1]*Dept Intgr Physiol, Med Grad Sch, Gunma Univ, Japan,* [2]*Epigenetics and Metabolism, IMCR, Gunma Univ, Japan,* [3]*Dept Nutr, Takasaki Univ Health Welf, Japan*)

The placenta secretes a prolactin (PRL)-like hormone PRL3B1 (placental lactogen II) in rodents. PRL3B1 is a luteotropic hormone essential for maintaining pregnancy until labor in mice. Previously, only one report examined the secretion pattern of PRL3B1 in prepartum mice. By measuring maternal plasma PRL3B1 and PRL every 4 hrs from gestational day 17 (G17), we newly discovered that maternal plasma PRL3B1 levels decreased rapidly in prepartum C57BL/6 mice. Interestingly, the onset of this decline coincided with the PRL surge at G18. Moreover, the expression level of *Prl3b1* mRNA was determined by RT-qPCR in the prepartum placenta and remained stable until parturition, suggesting PRL3B1 production or secretion was suppressed. We hypothesized that production of the PRL family, the 25 paralogous PRL genes exclusively expressed in mice placenta, would decrease alongside PRL3B1 during this period. To investigate this hypothesis and seek proteomic changes, we performed a shotgun proteome analysis of the placental tissue using data-independent acquisition mass spectrometry (DIA-MS). Up to 5891 proteins were identified, including 17 PRL family members. Relative quantitative analysis between G17 and G18 placentas showed no significant differences in PRL3B1 expression and most PRL family members except PRL7C, concluding that PRL3B1 secretion is suppressed at G18. DIA-MS identified 39 differentially expressed proteins (DEPs) with fold change ≥ 2. Thirty-two proteins were upregulated, and 7 were downregulated in the G18 group. Among the DEPs, AOC1L2, ENDOU, NPR3, PGR, SLCA1A, and UNC5B could represent new targets to elucidate the function of the terminal placenta.

### [1P-115]

### Deschloroclozapine exhibits an exquisite agonistic effect at lower concentration compared to clozapine-N-oxide in hM3Dq expressing chemogenetically modified rats

*Makiko Shimizu[1], Mitsuhiro Yoshimura[1], Kazuhiko Baba[2], Naofumi Ikeda[2], Yuki Nonaka[1], Takashi Maruyama[1], Tatsushi Onaka[3], Yoichi Ueta[1] ([1]*Department of Physiology, School of Medicine, University of Occupational and Environmental Health, Kitakyushu, Japan,* [2]*Department of Orthopaedic Surgery, School of Medicine, University of Occupational and Environmental Health, Kitakyushu, Japan,* [3]*Division of Brain and Neurophysiology, Department of Physiology, Jichi Medical University, Shimotsuke, Japan*)

Background: Chemogenetics has revolutionised our understanding of neuron-specific functions, particularly in behavioural research, allowing precise manipulation of targeted neurons. However, the common agonist clozapine-N-oxide (CNO) used for designer receptors exclusively activated by designer drugs (DREADDs) raises concerns due to potential off-target effects and side effects. Deschloroclozapine (DCZ), a metabolite of clozapine, has emerged as a promising alternative for DREADDs activation with minimal off-target interactions.

Methods: We compared the effects of CNO and DCZ in OXT-hM3Dq-mCherry transgenic rats expressing the hM3Dq receptor and mCherry specifically in oxytocin neurons. The administration of CNO (1 mg/kg) and DCZ (0.1 mg/kg) was followed by assessment of oxytocin levels, brain neuronal activity by Fos protein analysis and analgesic behaviour.

Results and discussion: DCZ at 0.1 mg/kg effectively activated DREADDs and showed comparable or superior efficacy to CNO at 1 mg/kg. This highlights the potential of DCZ in rat models for neurobehavioural studies, given its precision, minimal off-target effects and cost-effectiveness. Our findings also elucidate the specific brain nuclei activated by chemogenetic stimulation of oxytocin, extending the scope of DCZ application beyond mice and monkeys to transgenic rat models.

### [1P-116]

### Role of Nrf2 in insulin secretion from pancreatic β cells

*Yuta Kato[1], Taiyo Yoshibe[1], Eri Mukai[1] ([1]*Graduate school of Life Sciences, Ritsumeikan University*)

Defective insulin secretion from pancreatic b cells contributes to onset and development of type 2 diabetes. Insulin secretion from b cells is regulated by intracellular glucose metabolism, in which glucose-induced ATP production plays an essential role. In diabetic b cells, ATP production is impaired by excessively produced reactive oxygen species (ROS). When intracellular ROS increases, nuclear factor erythroid 2p45-related factor 2 (Nrf2), a transcription factor that regulates genes related to the antioxidants, dissociates from its regulatory factor Kelch-like ECH-associated protein 1 (Keap1) and then translocates into the nucleus. It has been reported that Nrf2 also transcripts genes related to glucose metabolism in lung cancer. In b cells, Nrf2 has been shown to activate the expression of antioxidant enzymes to raise cell viability, but the effect of Nrf2 on insulin secretion is unknown. We have previously shown that Nrf2 deficiency in pancreatic b cells reduces glucose-stimulated insulin secretion (GSIS) and that the reduction is partially restored by ROS scavengers. In the present study, we investigated the regulatory mechanisms of Nrf2 on GSIS.

Contrary to the effect of Nrf2 deficiency on GSIS in our previous study, Keap1 deficiency in INS-1 cells enhanced GSIS. Elevation of intracellular ATP levels by high glucose was attenuated by Nrf2 deficiency, while it was enhanced by Keap1 deficiency. We next examined the effects of Nrf2 and Keap1 deficiency on the expression of glutathione, an antioxidant tripeptide, and NADPH, the cofactor of antioxidant enzymes, both of which promote exocytosis of insulin granules. Nrf2 deficiency decreased mRNA level of glutamate-cysteine ligase catalytic subunit (Gclc), a part of synthetic enzyme of glutathione, and those of NADPH synthesis enzymes, such as glucose-6-phosphate dehydrogenase (G6pd) and malic enzyme 1 (Me1). In contrast, Keap1 deficiency increased mRNA levels of Gclc, G6pd, Me1, and isocitrate dehydrogenase 1 (Idh1), another NADPH synthesis enzyme. Furthermore, Nrf2 deficiency reduced the elevation in the NADPH/NADP+ ratio by high glucose.

The present study showed that Nrf2 positively regulates GSIS via ATP production whereas Keap1 negatively regulates it. Glutathione and NADPH were also suggested to contribute to the regulation of GSIS. Since the NADPH synthesis enzymes examined in the present study are associated with glycolysis and TCA cycle, Nrf2 may regulate GSIS via glucose metabolisms.

### Poster

### [1P]
### Autonomic nervous system

March 28, 13:00 - 14:20, Poster Room

### [1P-117]

### Activity of the brown adipose tissue, as evaluated by the interscapular skin temperature, increases at late phase of anaphylactic hypotension in awake rats

*Toshishige Shibamoto[1], Munenori Ono[2], Mamoru Tanida[1], Yuhichi Kuda[1], Yasutaka Kurata[1] ([1]*Department of Physiology II, Kanazawa Medical University,* [2]*Department of Physiology I, Kanazawa Medical University*)

The brown adipose tissue (BAT), that generates heat once activated, is largely located just beneath the interscapular region, the skin temperature of which (TiScap) could indirectly reflect BAT activity, that is regulated humorally and neurally via β3-adrenoceptor. We have recently reported that BAT is not primarily involved in anaphylactic hypothermia, as based on the observation that the core body temperature (Tcore) and TiScap, as measured by the nano-tag® and thermography, respectively, decreased, but that the decrease of Tcore preceded that of TiSap (Am J Physiol. 325: R446-R455, 2023). However, the exact activity of BAT should be evaluated by the difference between TiScap and Tcore (ΔTiScap). Thus, we here determined the changes in ΔTiScap by measuring Tcore, TiScap and mean blood pressure (MBP) during anaphylactic hypotension in awake ovalbumin-sensitized (anaphylaxis group; n=7) and -nonsensitized (control group; n=7) Sprague-Dauley rats. In the anaphylaxis group, MBP decreased to 53±2 mmHg at 8 min after antigen injection, and Tcore and TiScap significantly decreased by 1.9°C and 1.7°C at 30 and 30.5 min after antigen, respectively, followed by a tendency of recovery toward the baseline. ΔTiScap in the anaphylaxis group did not increase from the baseline of -1.84±0.13°C until 42.5 min after antigen injection, as compared with the control group, but thereafter significantly increased to the peak of -0.92±0.21°C at 47.5 min after antigen. Indeed, we previously reported that plasma catecholamines markedly increased at the late phase of anaphylaxis of the anesthetized rats (Am J Physiol. 305: R900-R907, 2013), which could account for the increased activity of BAT. In conclusion, activity of the brown adipose tissue may increase at late phase of anaphylactic hypotension in awake rats.

### [1P-118]

### Activation of a subclass of vagal afferent nerves through oxytocin treatment reduces anxiety and enhances social interaction by stimulating oxytocin neurons in the hypothalamic paraventricular nucleus.

*Kengo Iba[1], Yuta Masuda[1], Rika Kitano[1], Kento Ohbayashi[1], Yusaku Iwasaki[1] ([1]*Laboratory of Animal Science, Graduate School of Life and Environmental Sciences, Kyoto Prefectural Univ.*)

COVID-19 pandemic triggers a 25% increase in the worldwide prevalence rate of anxiety and depression. The central oxytocin (Oxt), which synthesized in the neurons of hypothalamic paraventricular nucleus (PVH) and supraoptic nucleus, is involved in the regulation of not only feeding behavior but also mental functions such as anxiety and sociality. Therefore, the Oxt neurons are a potential target for treating hyperphagic obesity and mental illness. However, there is currently no technology available to effectively activate Oxt neurons without causing side effects. In previous study (Y. Iwasaki et al., *BBRC* 2019), we have demonstrated that intraperitoneal (IP) administration of oxytocin activates PVH Oxt neurons via activation of vagal afferents expressing oxytocin receptors, thereby ameliorating hyperphagic obesity and diabetes in mice without inducing side effects. However, it remains unclear whether this peripheral-to-central coupling of Oxt, mediated by vagal afferent nerves, is also effective in regulation of mental functions. In present study, we examined whether peripheral oxytocin administration could regulate mental function such as anxiety and sociality via the Oxt's peripheral-to-central relay.

We measured anxiety behavior using the elevated plus maze and social behavior using the three-chamber social interaction test in C57BL/6J male mice. IP administration of Oxt one hour before the behavioral test significantly reduced anxiety-related behaviors and increased social behaviors. These effects are blunted by subdiaphragmatic vagotomy. Ip injection of Oxt activated PVH-Oxt neurons via vagal afferent neural pathway. When the neuronal activity of Oxt neurons in PVH was specifically and artificially suppressed using chemical genetic DREADD techniques, the anxiolytic and social promoting effects mediated by ip administration of Oxt were abolished. Furthermore, intraventricular administration of an oxytocin receptor antagonist completely inhibited the Oxt-induced psychotropic effects.

In conclusion, we demonstrate that the relay of peripheral Oxt to PVH Oxt neurons via vagal afferents reduces anxiety and enhances social interaction. This pathway holds promise for the development of psychotropic medications or functional foods with minimal side effects, which could contribute to better mental health.

### [1P-119]

### Intestinal GLP-1 and pancreatic insulin enhance insulin action through cooperative action at the common hepatic branch of vagal afferents

*Kento Ohbayashi[1], Toshihiko Yada[2], Yusaku Iwasaki[1] ([1]*Laboratory of Animal Science, Graduate School of life and Environmental Sciences, Kyoto Prefectural University, Kyoto, Japan,* [2]*Division of Integrative Physiology, Kansai Electric Power Medical Research Institute, Kobe, Japan*)

[Background] Glucagon-like peptide-1 receptor agonists (GLP-1RAs) directly act on pancreatic beta-cells to enhance glucose-induced insulin secretion and effectively ameliorate hyperglycemia. On the other hand, endogenous GLP-1 secreted from the intestine is highly unstable, therefore its physiological functions have not been fully elucidated. We have previously found that stimulation of intestinal GLP-1 release with the rare sugar D-allulose (Allu) enhances insulin-induced blood glucose lowering effect (Y. Iwasaki, *Nat Commun*, 2018). However, the detailed mechanisms remain unproven. Here, we investigated the effects of intestinal GLP-1 on glucose metabolism and the underlying mechanisms.
[Results] A single peroral (po) administration of Allu promoted GLP-1 secretion, however, did not alter blood glucose and plasma insulin levels in healthy and type 1 diabetic Akita mice. In type 2 diabetic (diet-induced obesity or *db/db*) mice with hyperglycemia and hyperinsulinemia, po Allu significantly ameliorated hyperglycemia without increasing insulin secretion, suggesting enhanced insulin action. These beneficial effects were blunted by the genetic or pharmacological inhibition of the GLP-1R. Comparing these results suggest that intestinal GLP-1 enhances insulin action in a manner dependent on plasma insulin levels. Exogenous insulin injection after oral Allu administration potentiated insulin-induced blood glucose lowering effect in Akita mice. Furthermore, co-administration of Allu and sulfonylurea drug, to induce simultaneous secretion of GLP-1 and insulin, enhanced activation of vagal afferent neurons and potentiated the hypoglycemic effects of sulfonylurea drug. These effects were completely abolished by chemical denervation of the common hepatic branch of vagal afferents. Finally, endogenous GLP-1 release by Allu more rapidly and potently ameliorated insulin resistance and hyperglycemia in *db/db* mice compared to GLP-1RA.
[Conclusion] Intestinal GLP-1 and pancreatic insulin work in concert to act on the common hepatic branch of vagal afferents, thereby enhancing insulin action and ameliorating hyperglycemia.

### [1P-120]

### Association between peripheral physiological functions and subjective happiness, loneliness, and depressive symptoms.

*Masahiro Matsunaga[1], Keiko Ishii[2], Ohtsubo Yohsuke[3], Noguchi Yasuki[4], Yamasue Hidenori[5] ([1]*Aichi Medical University,* [2]*Nagoya University,* [3]*The University of Tokyo,* [4]*Kobe University,* [5]*Hamamatsu University*)

In this study, we used a device (SKY10-self) that can measure the overall state of the body, such as the autonomic nervous functions, from physiological data including the electrocardiograms in about 5 minutes without putting any strain on the body. The purpose of this study was to clarify the relationship between psychological indices (subjective happiness level, loneliness, and depressive symptoms) and peripheral physiological functions. Healthy male and female volunteers aged 18 to 88 participated, and psychological indices were evaluated using a questionnaire and physical functions were evaluated using SKY10-self. The results showed a negative correlation between subjective happiness level and the proportion of low-frequency (LF) component of heart rate variability, and a positive correlation between loneliness and the proportion of LF component of heart rate variability. In addition, a positive correlation was found between subjective depressive symptoms and risk assessment scores for neuromuscular disorders in the cervical spine. Porges (2009) has recently established the polyvagal theory, which behaviorally links the mammalian myelinated vagal neurons (ventral vagal nerve complex), which originate in the nucleus ambiguus, to social communication (such as facial expression, vocalization, or listening). The ventral vagal nerve complex regulates cardiac functions and controls the tone of the trapezius and sternocleidomastoid muscles. Therefore, the finding of a relationship between psychological indices related to social emotions (subjective happiness, loneliness, depressive symptoms) and physiological funvtions related to the ventral vagal nerve complex suggests that there may be a significant correlation between human social functions and autonomic nervous functions.

### [1P-121]

### Effect of basal forebrain stimulation on regional blood flow in the piriform cortex

*Jura Moriya[1,2], Daichi Morihara[1,2], Fusako Kagitani[1], Sae Uchida[1] ([1]*Department of Autonomic Neuroscience, Tokyo Metropolitan Institute for Geriatrics and Gerontology,* [2]*Tokyo University of Agriculture and Technology*)

The piriform cortex, a major olfactory cortical area, receives odor information directly from the olfactory bulb, and sends the signals to the higher-odor association areas. The piriform cortex as well as the olfactory bulb, neocortex and hippocampus receives cholinergic projections from basal forebrain. Activation of the basal forebrain cholinergic system projecting into the neocortex and hippocampus releases acetylcholine (ACh), resulting in vasodilation and an increase in the regional blood flow in these regions. In the olfactory bulb, activation of the cholinergic neurons increases ACh release but has no vasodilatory action. Physiological role of the cholinergic projection to the piriform cortex is not well studied yet. Recently we showed that focal chemical stimulation of the diagonal band of Broca (HDB) in the basal forebrain, which is the main source of cholinergic input to the piriform cortex, increased extracellular ACh release in the piriform cortex. This study aimed to clarify whether the basal forebrain cholinergic projection to the piriform cortex contribute to the regulation of regional blood flow.

Using anesthetized rats, regional blood flow in the piriform cortex was measured by laser Doppler flowmetry. The HDB was focally stimulated chemically by microinjection of L-glutamate (50 nmol in 50 nl or 100 nmol in 100 nl) for 1 min.

The HDB stimulation increased regional blood flow in the ipsilateral piriform cortex in 4 of 6 rats with 50 nmol of L-glutamate, and in all 6 rats with 100 nmol of L-glutamate. The blood flow reached a maximum at 2-4 min after the stimulation, reaching approximately 112% and 127% of the pre-stimulus basal levels, respectively. The mean arterial pressure was not influenced significantly by the HDB stimulation.

The present study showed the vasodilatory role of basal forebrain input to the piriform cortex, like the neocortex and hippocampus. The increased regional blood flow in the piriform cortex may contribute the protection of neurons and maintenance of functions by providing sufficient oxygen and nourishments.

### [1P-122]

### Does the range of tones in a music piece affect autonomic nervous activity while listening?

*Junko Hoshi[1], Konosuke Sasaki[2], Ryoko Maruyama[1,2] ([1]*Dokkyo Medical Univ.,* [2]*Tohoku Univ.*)

We evaluated minute-by-minute changes in autonomic nervous system activity in healthy young adults listening to music. Forty volunteers participated in 3 tasks consisting of 3 experimental conditions: a 10-min rest and sympathetic nervous activity load test, followed immediately by 8 min of listening to Mozart's "Piano Sonata for Two Hands" in D major (K448), J. S. Bach's "Brandenburg Concerto No. 4" in G major (BWV1049), or silence as a control, in a randomized order. Electrocardiography was continuously recorded from the start to the end of each data collection point. We compared the average changes in the low frequency (LF)/high frequency (HF) ratio (LF/HF), a measure of sympathetic nervous activity, between the loading and listening periods with K448, BWV1049, or silence. LF/HF decreased significantly from loading to listening for BWV1049 and silence, but not for K448. We also investigated whether there was a difference between the three conditions for each minute of the 8 minutes of music listening, but found no significant difference in LF/HF. However, the pattern of change in LF/HF over time during music listening from 2 to 3 minutes after the start of listening showed different responses: higher during K448 listening and lower during BWV1049 listening. A comparison of the tones used during the first 2 to 3 min of the two pieces revealed that K448 was played in a wider range of tones than was BWV1049. Our findings suggest that the range of tones of a music piece may affect autonomic nervous activity. COI: This study was supported by JPSS KAKENHI Grant Number JP20K23214.

### [1P-123]

### Distribution of subcluster neurons of the nucleus of the solitary tract; on neurons responding to hypoxia

*Shigefumi Yokota[1], Matsuo Hiroyuki[2] ([1]*Dept. of Anat. & Neurosci., Shimane Univ. Sch. of Med.,* [2]*Dept. of Exp. Animals., Interdiscip. Ctr. for Sci. Res., Shimane Univ.*)

General visceral information, including arterial partial oxygen pressures, is conveyed to the nucleus of the solitary tract (NTS) via the vagus and glossopharyngeal nerves, and this information is used to maintain homeostasis by the central nervous system. Recent transcriptome analyses have shown that NTS neurons can be classified into 12 glutamatergic and 3 GABAergic subclusters. Although transcriptome analysis has revealed the types and amounts of mRNAs expressed in NTS neurons, the distribution of neurons classified by these types of mRNAs is still unknown. Furthermore, it is also unknown which subcluster neurons respond to specific visceral stimuli. In this study, we first showed the distribution of each subcluster neuron using combinations of *in situ* hybridization to detect marker mRNAs and immunohistochemistry. For this purpose, we prepared probes to detect 12 characteristic marker mRNAs. We then classified the glutamatergic neurons that express *Fos* mRNA after exposure to hypoxia into those 12 subclusters. The results showed that each subcluster neuron was distributed in a characteristic pattern. For example, some were distributed specifically in the central subnucleus of the NTS, while others were densely distributed in abundance adjacent to the ventral margin of the area postrema. After exposure to hypoxia (8% O2, 92% N2) for 30 min, we found many *Fos* mRNA-positive neurons in the NTS; approximately 75% of them were *Vglut2*-expressing glutamatergic neurons, 22% of them were *Vgat*-expressing GABAergic neurons, and 3% of them were unidentified neurons. In addition to our subcluster classification method, the use of in situ hybridization to detect *Fos* mRNA enabled us to classify *Fos* mRNA-expressing glutamatergic neurons into 12 subclusters. The subcluster neurons shown in this study indicate the origin of the central neuronal network of respiratory regulation by arterial partial oxygen pressures. We will further analyze the neural circuits involved in the homeostatic response, originating from NTS neurons in response to hypoxia.

### Poster

### [1P]
### Environmental physiology

March 28, 13:00 - 14:20, Poster Room

### [1P-124]

**Period-dependent maternal separation may alter the emotional behavior of mouse pups via monoamine signaling**

*Noriyuki Shimizu[1,2], Yurika Imada[4], Sachiko Chikahisa[2,3], Tetsuya Shiuchi[2], Hiroyoshi Sei[2] ([1]*Department of Anatomy and Cell Biology, Institute of Biomedical Sciences, Tokushima University Graduate School.,* [2]*Department of Physiology, Institute of Biomedical Sciences, Tokushima University Graduate School.,* [3]*Department of Health and Nutrition, Faculty of Human Life Science, Tokushima, Shikoku University.,* [4]*University Faculty of Medicine, Tokushima University Graduate School.*)

Early postnatal stress due to abuse or neglect can increase the risk of developing emotional disorders in adulthood, but the mechanism is still unclear. In this study, we focused on the period of infancy and investigated how period-dependent maternal separation (MS) affects the emotional behavior of mouse pups.

Female C57BL/6J mice (10 weeks old) were mated with male mice of the same age. Pups were separated from their mothers for 3 hours per day. Two MS paradigms were performed: postnatal day (PND) 2–12 (early) and PND 10–20 (late). The control was the non-MS group. Pups were weaned at 3 weeks of age. When the pups reached 10 weeks of age, their emotional behaviors were evaluated using behavior analysis.

The early-MS group showed a decrease in sniffing time in the social interaction analysis compared to the control group. Compared to the control, the late-MS group spent more time in the center area in the open field, and more time and entries into the open arms in elevated plus maze. The early-MS showed decreased dopamine content in lateral septum and decreased mRNA expression of genes related to dopamine signaling, such as dopamine receptor D3 (Drd3), monoamine oxidase A (Maoa) and solute carrier family 6 member 3 (Slc6a3). On the other hand, late-MS reduced serotonin content and decreased mRNA expression of hydroxytryptamine receptor 2A (Htr2a) in frontal cortex.

These results indicate that the duration and timing of separation from the mother may alter the emotional behavior for mouse pups via monoamine signaling (dopamine and serotonin).

### [1P-125]

**Physiological and cellular impacts of chronic oral exposure to plastic nanoparticles in area postrema and nucleus tractus solitarii rats**

*Linh Thuy Pham[1,4], Ko Yamanaka[2], Yasunori Miyamoto[3,4], Hidefumi Waki[1,2], Sabine S. S. Gouraud[5] ([1]*Inst. of Health & Sport Sci. & Med., Juntendo Univ., Chiba, Japan,* [2]*Dept. of Physiol., Grad. Sch. of Health & Sports Sci., Juntendo Univ., Chiba, Japan,* [3]*Inst. for Human Life Innovation, Ochanomizu Univ., Tokyo, Japan,* [4]*Grad. Sch. of Humanities & Sci., Ochanomizu Univ., Tokyo, Japan,* [5]*Dept. of Natural Sci., College of Liberal Arts, International Christian Univ., Tokyo, Japan*)

**[Aims]** Recently, human oral exposure to environmental plastic particles has raised significant concern. Microplastics have been detected in human blood, while nanoplastics were found to accumulate in multiple organs and cross the blood-brain barrier (BBB). However, the impact of nanoplastics on BBB-free areas such as Area Postrema (AP) remains unstudied. AP is involved in blood pressure (BP) control via its connection to Nucleus Tractus Solitarii (NTS), a BBB-protected region crucial for regulating the set point of BP. AP exhibits a denser population of microglial cells, the brain's immune cells, in comparison to the NTS. We investigated the effect of oral exposure to polystyrene nanoparticles (PSNPs) on rats' physiological parameters **and microglial** reactivity in AP and NTS. **[Methods]** Four-week-old Wistar rats (n=18) were conditioned to consume either a 3% sucrose solution containing Aminated PSNPs (NP+ group), Carboxylated PSNPs (NP- group) or no PSNPs (CTR group). The physiological parameters were assessed weekly, and after two months of exposure, brain tissues were collected for microglia morphology study. **[Results]** A two-month PSNP exposure significantly decreased the heart rate and increased the urine osmolality. Furthermore, microglia in the NTS exhibited more pronounced reactivity to PSNPs compared to those in the AP. **[Conclusion]** These observations suggest that the degree of microglia sensitivity to circulating PSNPs depends on the presence of a BBB. The reaction of microglia to PSNPs in AP and NTS might mediate changes in the functioning of neighboring neurons, potentially influencing the regulation of heart rate and urine osmolality through mechanisms that require further investigation.

*COI: NO*

### [1P-126]

**Effects of tail suspension on hypothalamus oxytocin neurons in oxytocin-mRFP1 transgenic rats**

*Hirofumi Hashimoto[1,2], Hajime Hoshiai[2], Yumi Chiba[2], Takashi Mizushima[2], Naohiko Anzai[1] ([1]*Dept. Pharmacology, Chiba Univ. Graduate Sch. Med.,* [2]*Department of Rehabilitation, Dokkyo Medical University*)

Oxytocin (OXT) is a well-known neuropeptide related with uterine contractions and milk ejection reflex. Previous study showed that plasma OXT level decreased and administration of OXT induced muscle regeneration in aging mice. We showed that the fluorescence intensity of mRFP1 increased in the supraoptic (SON) and paraventricular nuclei (PVN) in aging OXT-monomeric red fluorescent protein 1 (mRFP1) transgenic rats. We also showed that Fos-like immunoreactive (LI) cells significantly increased in the SON and PVN in immunohistochemistry study for Fos at 90 mins after tail-suspended (TS) stimulation.

In this study, two weeks after tail-suspended (TS) stimulation, we investigated changes of the fluorescence intensity of mRFP1 in the young OXT-mRFP1 transgenic rats. The fluorescence intensity of mRFP1 did not any changes in the SON PVN in TS group, compared with control group. These results suggested that the stimulation of TS for two weeks did not change the central oxytocin neurons in young rats.

### [1P-127]

### The influence of indoor environment factors on thermal comfort and fatigue

*KEI NAGASHIMA[1], Hironori Watanabe[1], TAISUKE SUGI[1] ([1]*Waseda University*)

**[Background]** Thermal comfort is a parameter evaluating ideal living and working environments. Many factors are proposed for the assessment of the environments; however, the psychophysiological meanings remain unclear. **[Aims of the study]** First, we assessed the influence of ambient temperature and relative humidity on thermal comfort. Second, we hypothesized that thermal comfort would be related autonomic activity assessed by heart rate variability (HRV), stress hormones secreted in saliva, and work performance evaluated by calculation task. **[Methods]** Twelve healthy volunteers (age ranged 20 – 37 y; seven males, five females). They sat on a chair for 120 min in an environmental chamber maintained at an ambient temperature of 26˚C or 33˚C and relative humidity of 30% or 60%. They completed the 4 trials with a two-day interval at least. During each trial, the participants repeated 20-min calculation task three times (Kraepelin test) with a 10-min interval. Thermal and humid sensations, thermal comfort and psychological and physical fatigue were separately reported by a visual analogue scale before starting each trial and each end of the calculation task. Saliva was sampled after the report, of which amylase and IgA levels were analyzed by ELISA. ECG was continuously recorded for later analyses of HRV. **[Results]** Participants reported hotter and uncomfortable at 33˚C and 60% and cooler and comfortable at 26˚C and 30%; however, the ratings between 33˚C/30% and 26˚C/60% did not differ. The ratings for psychological fatigue increased with time; however, no differences were observed among the four trials. The amount of the completed calculation tasks and saliva levels of amylase and IgA were not different among the trials. Based on the HRV analysis, RMSSD decreased from 55 ± 10 to 40 ± 11 msec in the 33˚C/60% trial; however, the value remained unchanged in the 26˚C/30% trial. **[Conclusion]** The present study indicated that both thermal sensation and comfort were determined by the influence of ambient temperature and relative humidity. Moreover, to estimate the thermal perception, humidity is an important indicator. We could not verify that thermal comfort is a determinant for psychological working performance and fatigues. However, the vagal activity estimated by HRV analysis may suggest that greater sympathetic activation is needed to complete the same task in an uncomfortable environment.

### [1P-129]

### Characteristics and usefulness of the house musk shrew (*Suncus murinus*) as a model for daily torpor

*Saho Okuda[1], Yuuki Horii[1,2], Kanako Okadera[1], Takahiko Shiina[1], Yasutake Shimizu[1]
([1]*Laboratory of Veterinary Physiology, Faculty of Applied Biological Sciences, Gifu Univ.*, [2]*Institute for Glyco-core Research (iGCORE), Gifu Univ.*)

### [1P-128]

### Simulated microgravity inhibits myoblast proliferation via reduction in intracellular $Ca^{2+}$ levels

*Ayaka Ichihara[1,2], Jun Tanihata[2], Yuki Enoki[1], Kazuaki Matsumoto[1], Susumu Minamisawa[2] ([1]*Division of Pharmacodynamics, Keio University Faculty of Pharmacy*, [2]*Division of Aerospace Medicine, Department of Cell Physiology, The Jikei University School of Medicine*)

[Background] Microgravity induces muscle atrophy due to decreased protein synthesis and increased protein degradation in muscle, which is one of the most serious physical problems when going to space and needs to be solved. Although many previous studies have studied about protein degradation under microgravity, the only report focused on the effect of microgravity on proliferation capacity (Tatiana et al. FASEB J. 2013) and indicated that simulated microgravity inhibited myoblast proliferation through decreased TRPC1 expression. However, the relationship between the suppression of myoblast proliferation and intracellular $Ca^{2+}$ levels ($[Ca^{2+}]_i$) under microgravity remains unclear. Therefore, we hypothesized that microgravity would decrease in $[Ca^{2+}]_i$ and then inhibit cell proliferation. The purpose of this study was to test our hypotheses, and to identify the intracellular $Ca^{2+}$ dynamics-related factors that are altered by microgravity.

[Methods] The mouse skeletal muscle-derived cell line C2C12 cells were divided into two groups: a control group cultured under 1G condition (CON group: n=6/time point) and a simulated microgravity group cultured under 1/1000G condition using the gravity control device Gravite® (SMG group: n=6/time point). Cell number was measured at 24 and 48 hours. $[Ca^{2+}]_i$ were examined using Fluo4-AM at 24 and 48 hours. mRNA expressions of the cell cycle markers (Ki67), mechanosensitive ion channels (TRPC1, TRPM7 and Piezo1,2), and intracellular $Ca^{2+}$ dynamics-related factors on the sarcolemma and on the endoplasmic reticulum membrane were analyzed by quantitative RT-PCR at 24 and 48 hours.

[Results] Cell number and $[Ca^{2+}]_i$ at 24 and 48 hours were significantly lower in the SMG group than in the CON group. On the other hand, mRNA expressions of the cell cycle marker Ki67 and mechanosensitive ion channels TRPC1, TRPM7 and Piezo2 were decreased in the SMG group only at 48 hours.

[Conclusion] Although microgravity reduced cell proliferation and $[Ca^{2+}]_i$ at 24 and 48 hours, mRNA expressions of mechanosensitive ion channels were reduced only at 48 hours. These results suggest that the inhibition of cell proliferation occurred by different mechanisms in the early phase (0-24 hours) and the late phase (24-48 hours). In the early phase, the mechanism by which $[Ca^{2+}]_i$ were reduced is not clear from this study. In the late phase, microgravity reduced the mRNA expressions of mechanosensitive ion channels and decreased $[Ca^{2+}]_i$, which delayed the cell cycle and inhibited cell proliferation. Therefore, it is necessary to identify the mechanisms by which microgravity alters $[Ca^{2+}]_i$ in the early phase and late phase, respectively.

### Poster

### [1P]
### Nutritional and metabolic physiology, Thermoregulation

March 28, 13:00 - 14:20, Poster Room

### [1P-130]

### Thermoregulation by female hormones in ovariectomized rats administered TREK agonist

*Yuki Uchida[1], Shotaro Kamijo[2], Yuki Samejima[1,3], Motoyasu Honma[1], Yuri Masaoka[1], Masahiko Izumizaki[1] ([1]*Department of Physiology, Showa University School of Medicine,* [2]*Division of Physiology, Toxicology and Therapeutics, Department of Pharmacology, Showa University,* [3]*Department of Orthopaedic Surgery, Showa University Fujigaoka Hospital, Kanagawa, Japan*)

**INTRODUCTION** The TWIK-related potassium (TREK) channels have been identified as novel cold receptors. The TREK channels are co-localized with another cold receptor, transient receptor potential cation channel subfamily M member 8 (TRPM8), in sensory neurons. The effect of estradiol ($E_2$) and progesterone (P) on thermoregulatory responses through TREK in ovariectomized rats was elucidated. **METHODS** [*Experiment 1*] Ovariectomized rats were implanted a silastic tube with or without $E_2$ underneath the dorsal skin ($E_2$ (+) and $E_2$ (−) groups) and data logger for body temperature ($T_b$) and activity measurement into the peritoneal cavity. On the day of the experiment, rats were intraperitoneally administered a TREK agonist (Ostruthin, 4.2μg) or vehicle at 10:00. Measurements included $T_b$, activity, tail skin temperature ($T_{tail}$) assessed by thermography, oxygen consumption assessed by the expiratory gas monitor, and thermoregulatory behavior that assessed by tail-hiding behavior for 2 hours at 27ºC. After the experiment, the blood and dorsal root ganglia were collected. Plasma catecholamine, triiodothyronine, thyroxine, and mRNA levels of TREK1, TREK2, TRAAK, and TRPM8 were assessed. [*Experiment 2*] Ovariectomized rats were implanted silastic tubes with or without P underneath the dorsal skin (P(+) and P(−) groups). The experiment was conducted similar to *Experiment 1*. **RESULTS** TREK agonist increased $T_b$ in the $E_2$ (+) group. In TREK agonist group, $T_b$ in the $E_2$ (+) group was greater than that in the $E_2$ (−) group. TREK agonist decreased $T_{tail}$. There was a tendency for $E_2$ to decrease $T_{tail}$. $E_2$ increased plasma triiodothyronine. In TREK agonist group, $E_2$ increased TREK1 mRNA level in the dorsal root ganglia. In $E_2$ (−) group, TREK agonist increased TRPM8 mRNA level. No significant differences in other measurements were observed among the groups. In TREK agonist group, $T_b$ was not different between P(+) and P(−) groups. **CONCLUSION** $E_2$, not P, might increase $T_b$ in ovariectomized rats through TREK channels. COI:NO, KAKENHI:20K07275

### [1P-131]

### Imaging analysis of metabolic kinetics in hepatocytes during glucose depletion and reperfusion

*Saki Tsuno[1,2], Kazuki Harada[1], Mina Horikoshi[3], Marie Mita[1], Tetsuya Kitaguchi[4], Masami Yokota Hirai[5], Mitsuharu Matsumoto[2], Takashi Tsuboi[1,3] ([1]*Department of Life Sciences, Graduate School of Arts and Sciences, The University of Tokyo,* [2]*Dairy Science and Technology Institute, Kyodo Milk Industry Co. Ltd.,* [3]*Department of Biological Sciences, Graduate School of Science, The University of Tokyo,* [4]*Laboratory for Chemistry and Life Science, Institute of Innovative Research, Tokyo Institute of Technology,* [5]*RIKEN Center for Sustainable Resource Science*)

The liver plays a crucial role in various physiological processes. Maintaining energetic homeostasis requires adequately absorbing and delivering energy sources, including glucose. When fasting, blood glucose levels decrease, and the liver provides glucose to the body through glycogenolysis and gluconeogenesis. When feeding occurs, there is a sufficient amount of glucose from energy sources available and fasting-induced responses quickly disappear. During these environmental changes, hepatocytes can switch metabolic processes. However, the dynamics of metabolites (i.e., lactate, pyruvate and ATP) in hepatocytes during the metabolic switching remain unknown. The purpose of this study is to explore the molecular mechanisms responsible for the rapid response to glucose absorption. We analyzed the dynamics of intracellular metabolites during the recovery from glucose deprivation, which mimics the transition from fasting to glucose feeding in primary mouse hepatocyte cultures *in vivo*. Glucose administration to hepatocytes under glucose-deprived conditions resulted in little change in the cytoplasmic lactate, pyruvate, and ATP concentrations. In contrast, we observed a decrease in the mitochondrial ATP concentration. The reduction in mitochondrial ATP concentration was associated with increased protein synthesis rather than that of glycogen synthesis and activation of urea cycle. Furthermore, glucose administration had little effect on the production of intracellular reactive oxygen species and cell viability. These findings suggest the significance of mitochondrial ATP during glucose deprivation or administration in hepatocytes.

### [1P-132]

### Analysis of astrocytic Glut1 function for the development of a novel therapy for Glut1 deficiency syndrome

*Shoko Tamura[1], Hiroko Shimbo[1,2], Jonghyuk Park[1], Haruo Okado[1], Shinobu Hirai[1] ([1]*Tokyo Metropolitan Institute of Medical Science, Department of Psychiatry and Behavioral Sciences, Brain Metabolic Regulation Group,* [2]*Kanagawa Children's Medical Center, Clinical Research Institute*)

**[Background]** Glucose is the main energy source for cells that make up the brain, and glucose transporters primarily mediate glucose uptake into the brain. Glucose transporter-1 (Glut1) has been reported to be mainly expressed in vascular endothelial cells and astrocyte endfeet. Glut1 deficiency syndrome (Glut1-DS) is a metabolic encephalopathy caused by impaired glucose transport into the brain. Glut1-DS is a congenital disease, and heterozygous de novo mutations in the Glut1 gene (*SLC2A1*) have been found in the majority of Glut1-DS patients. In Glut1-DS, the blood glucose level is normal, but CSF glucose is low, resulting in hypoglycemia within the central nervous system, which leads to a variety of CNS dysfunctions including cognitive and locomotor disability. Several papers have reported that Glut1 expressed in vascular endothelial cells (ECs) is important in neurological symptoms, and the development of Adeno Associated Virus (AAV) treatment in Glut1 DS patients also targets the vascular ECs.
**[Methods]** In this study, we focused on the astrocytic Glut1 and investigated its role in Glut1 DS by using astrocytes-specific Glut1 haploinsufficient (Aldh1l1-cre;Glut1 fl/+) mice. P48-62 male and female Aldh1l1-cre;Glut1 fl/+ mice were also used in the measurement of the cerebral spinal fluid (CSF) and interstitial fluid (ISF) glucose concentration. These mice were also used for behavioral tests to evaluate cognitive and locomotor function. The degree of inflammation in the brain of Aldh1l1-cre;Glut1 fl/+ mice was analyzed after behavioral tests.
**[Results]** First, we found that Glut1 was expressed throughout astrocytic brunches, not just at endfeet. Next, we analyzed Aldh1l1-cre;Glut1 fl/+ mice. The glucose level in the CSF and ISF was decreased in Aldh1l1-cre;Glut1 fl/+ mice despite normal blood glucose levels. Behavioral tests showed decreased cognitive and locomotor function in Aldh1l1-cre;Glut1 fl/+ mice compared with wild type mice. These symptoms in Aldh1l1-cre;Glut1 fl/+ mice were similar to those observed in Glut1 haploinsufficient mice.
**[Conclusions]** These results suggest that that Glut1 in astrocytes plays a crucial role in glucose uptake into CSF and ISF, and cognitive and locomotor functions. Glut1 in astrocytes, not only in vascular ECs, may be involved in the pathogenesis of Glut1 DS and a promising therapeutic target for Glut1 DS.

### [1P-133]

### Daily exercise training prevents cerebellar dysfunction in a rat model of heat stroke

*Kentaro Matsuzaki[1], Haruki Sakai[1], Naotoshi Sugimoto[1,2], Osamu Shido[1,3], Hiroko Kishi[1] ([1]*Shimane Univ.*, [2]*Kanazawa Univ.*, [3]*Shimane Rehabilitation college*)

Daily exercise training is beneficial in preventing heat stroke (HS), but the mechanism is unclear. In this study, we examined the preventive effect of exercise training on motor dysfunction in a rat model of HS. Male Wistar rats (10-week-old) were subjected in cages with running wheels for 30 days (training group: TR). The control group (CN) were kept in normal cages for 30 days. Rats in each group were then exposed to intensive heat (40°C) to induce HS, while the normal group (NT) was maintained at 25°C. The rats were then evaluated for motor function in a rotarod test, and cerebellum and plasma were collected for biochemical and histological analysis. Compared to CN-NT, CN-HS showed impaired rotarod performance, whereas TR-HS showed significant improvement. HS reduced calbindin protein expression, a marker of Purkinje cells in the cerebellum, which was ameliorated by exercise training. HS significantly induced neuronal degeneration and lipid peroxidation in the cerebellum, but these were suppressed by exercise training. Furthermore, inflammatory cytokine and p-NFkB levels in the cerebellum were enhanced in CN-HS, but significantly improved in TR-HS. Daily exercise training may prevent HS-induced motor dysfunction by inhibiting neurodegeneration, inflammatory responses, and oxidative stress in the cerebellum.

### [1P-134]

### Spatial pattern of brain activity involved in thermal comfort and discomfort

*Hironori Watanabe[1], Satoshi Shibuya[2], Taisuke Sugi[1], Kisyoshi Saito[1], Kei Nagashima[1] ([1]*Waseda University*, [2]*Kyorin University*)

Thermal comfort/discomfort (i.e., the hedonic component of thermal perception) is induced in the brain via afferent feedback from peripheral receptors, but it remains controversial which brain regions are relevant. The aim of the present study was to identify brain regions involved in thermal comfort and discomfort using electroencephalography (EEG). Fourteen participants received local thermal stimuli to both cervical regions of either 24.0°C or 40.0 °C using Peltier devices during whole-body thermal stimuli of 17.0°C or 47.0°C via a water-perfusion suit. To induce different thermal comfort and discomfort, the local thermal stimulus was consisted of a paired-thermal stimulus consisting of a 15-s reference stimulus (32°C) followed by a 10-s conditioned stimulus at either 24°C or 40°C. Using these combinations, the experiment comprised four thermal conditions: $Cold_{body}Cold_{local}$ and $Cold_{body}Hold_{local}$, which induced cold-discomfort and hot-comfort, and $Hot_{body}Cold_{local}$, and $Hot_{body}Hot_{local}$, which induced cold-comfort and hot-discomfort, respectively. Fifteen-channel EEG signals were continuously measured during each condition. For further analysis, 40 EEG responses were collected in each condition. To identify the brain regions involved in thermal comfort and discomfort, independent component (IC) analysis was applied to the preprocessed EEG data. The equivalent current dipole locations were then estimated, followed by clustering (k-means method) of the ICs with a dipole residual variance of < 15%. For the time-frequency analysis of each target cluster, event-related spectrum perturbations (ERSP) were compared between conditions. Clustering identified clusters involved in thermal comfort and discomfort, whose dipoles were located in the anterior and posterior cingulate cortices, middle temporal gyrus, and inferior frontal gyrus. However, the ERSP of the clusters did not differ between conditions. The present results suggest that cold-and hot-comfort/discomfort may be induced in common brain regions, regardless of different perceptions.

### [1P-135]

### Hypoactivation of viscerosensory brainstem nuclei responding to anorectic gut hormones in adolescent and middle-aged mouse with binge-like sugar consumption

*QILU GUAN[1], Yasunobu Yasoshima[1] ([1]*Physiological Laboratory, Graduate School of Frontier Biosciences, Osaka University*)

Gut-brain communication is one of regulatory mechanisms for feeding behavior. Normal nutrient consumption stimulates gastrointestinal tract, triggering the secretion of anorectic gut hormones through intestinal nutrient sensing., this process activates viscerosensory brainstem regions, the nucleus of tractus solitarius (NTS) and the external lateral regions of parabrachial nucleus (PBNel), through hormonal activation of the vagus nerve. It has been suggested that dysfunction in the viscerosensory brainstem pathway is one of the underlying mechanisms contributing to dysregulated binge-like behavior; we assumed that attenuated brainstem responses to anorectic gut hormones would be observed in mice showing binge-like sucrose consumption. Our unpublished data support the hypothesis; however, studies on binge-like behavior had limited only in adolescent mice. It remains unclear whether age differences result in distinct viscerosensory response patterns to the gut hormone after binge-like sucrose consumption. To address the issue, we compared binge-like consumption and brainstem activation between the adolescent and middle-aged mice with limited access procedure. Both groups showed gradual increase of sucrose consumption; however, the middle-aged group displayed significantly lower . After limited access training, the adolescent and middle-aged mouse received an intraperitoneal administration of saline or peptide YY (PYY3–36; 25 nmol/kg)) or cholecystokinin (CCK-8; 4 μg/kg). Number of c-Fos-like immuno-positive neurons in the NTS and PBNel were counted. It was observed that We also found age difference in the numbers of gut hormone-induced c-Fos positive neuronsbetween adolescent and middle-aged groups with binge-like behavior. Previous study suggested that catecholaminergic neurons in the NTS mediate feeding suppression by CCK-8; thus, we will compare the number of c-Fos and tyrosine hydroxylase (a catecholaminergic cellular marker) double-positive neurons in the NTS and PBNel after CCK-8 administration between adolescent and middle-aged mice.

### Poster

### [1P]
### Behavior, Biological rhythm, Sleep

March 28, 13:00 - 14:20, Poster Room

### [1P-136]

### Effects of hibernation-like hypothermic/hypometabolic state on sleep-wake behavior and neuronal activity in mice

*Yuma Takaishi[1], Tohru Takahashi[1], Takeshi Sakurai[1] ([1]*University of Tsukuba*)

Optogenetic or pharmacogenetic activation of a specific subpopulation of neurons in the anteroventral periventricular nucleus of the preoptic hypothalamus induces a hypothermic/hypometabolic state in mice that is similar to hibernation. These neurons, known as Q neurons, can trigger what we refer to as Q-neuron-induced hypothermic/hypometabolic state (QIH) [1]. Mice autonomously recover from QIH without any observed tissue damages, yet the underlying processes that occur in both the brain and peripheral tissues during QIH remain poorly understood.Therefore, we are investigating the effects of QIH on physiological functions involving the brain. We began by assessing sleep-wake behavior, which is indispensable for systemic homeostasis. We induced QIH via optogenetic excitation of Q neurons and examined alterations in sleep-wake behavior before, during and after QIH.

To determine quantitative changes in sleep-wake behavior before, during and after QIH, we examined the amounts and episode durations for each sleep stage. Compared to those before QIH, wakefulness increased for 2-4 hours and then decreased for 4-6 hours while NREM and REM sleep durations exhibited opposite changes in the 12 hours immediately following QIH. Although the total amounts per 12 hours did not change, the episode durations of wakefulness and NREM sleep were likely to shorten. These acute behavioral changes differed from those after 6 hours of sleep deprivation, which sleep transiently increased and fragmented. and those after daily torpor in which NREM sleep amount was comparable to baseline [2]. In the next 12 hours, the amounts returned to baseline, but the episode durations were significantly longer, revealing the consolidation of each stage. These changes were more pronounced with the 48-hour stimulation than with the 24-hour stimulation.

In sleep studies, each stage is generally distinguished by a characteristic EEG pattern. Therefore, EEG power spectrum analysis was performed to reveal qualitative changes in sleep-wake behavior before, during and after QIH. Similar to daily torpor [2], EEG amplitude was significantly attenuated during QIH. The EEG power spectrum analysis during NREM sleep showed that EEG amplitude recovered over several hours after QIH and remained elevated for at least 12 hours. Delta wave (0.5–4 Hz) during NREM sleep after daily torpor or sleep deprivation also increases [2], but the EEG pattern after QIH differed in terms of the presence of a recovery period, sustained enhancement, and enhancement in the higher frequency bands.

Takahashi, T.M. et al. *Nature* 583, 109–114 (2020)
V. V. Vyazovskiy et al. *Cerebral cortex* 27, 950-961 (2017)

### [1P-137]

### Sleep homeostasis in lizards and the role of cortex

*Sena Hatori[1], Sho Yamaguchi[1], Futaba Matsui[1], Zhiwen Zhou[1], Hiroaki Norimoto[1] ([1]*Department of Cellular Pharmacology, Graduate School of Medicine, Hokkaido University*)

Although their phenotypes are diverse, slow-wave sleep (SWS) and rapid eye movement sleep (REMS) are the two primary components of electrophysiological sleep (e-sleep) in mammals and birds. Slow waves in the cortex not only define SWS but are also used as biological markers for sleep homeostasis, given their rebound after sleep deprivation (SD). Recently, we discovered that the Australian dragon *Pogona vitticeps* exhibits two-stage sleep pattern in the dorsal ventricular ridge (DVR), which includes a homologue of the mammalian claustrum. It remains unclear whether reptilian e-sleep, which has been characterized by activity outside the cortex, compensates for sleep loss, as observed in mammals. We here report a significant rebound in the local field potential (LFP) after 7 hours of SD, during both SWS and REMS. Meanwhile, the duty cycle and mean bout length remained unaffected. We further investigated a possible role of the cortex in e-sleep regulation and homeostasis in *Pogona* and found that, although a corticotomy had no obvious effect on the LFP features investigated, corticotomy abolished LFP power rebound in the DVR after SD. These findings suggest that e-sleep homeostasis is a common feature in amniotes, and that cortex is involved in regulating activity rebounds in reptiles and mammals.

### [1P-138]

### Sleep inproving effect of Melinjo (Gnetum gnemon L.) seed extract in diet-induced obesity mice

*Akira Terao[1], Mao Sato[1], Chiaki Sugiura[1], Nobuaki Okumura[2] ([1]*Department of Biology, School of Biological Sciences, Tokai University,* [2]*Institute for Bee Products and Health Science, Yamada Bee Company Inc.*)

We investigated the effect of dietary melinjo (*Gnetum gnemon L.*) seed extract (MSE) on sleep architecture in high-fat diet (HFD)-induced obese mice. Fifty-six C57BL/6J male mice were fed different diets for 17 weeks: normal diet (ND), ND with 1% MSE (ND+MSE), HFD, and HFD with 1% MSE (HFD+MSE). Body weight and sleep architecture were examined in all mice after the study period. The body weight of HFD-fed mice increased by 50% compared to that of ND-fed mice. Although HFD did not affect the amount of non-Rapid Eye Movement (NREM) sleep, the average duration of NREM sleep bout was significantly shortened, and the number of NREM sleep bout was significantly increased. These findings indicate fragmented NREM sleep and altered sleep architecture resulted in impaired sleep quality in HFD-fed mice. Dietary MSE did not affect body weight or sleep architecture in the ND+MSE-fed mice. In contrast, the body weight and sleep architecture of HFD+MSE-fed mice were almost identical to those of ND-fed mice, indicating that dietary MSE completely blocked HFD-induced weight gain and sleep fragmentation. Our data provide compelling evidence that MSE is a promising dietary supplement that restores obesity-induced impaired NREM sleep quality in mice.

### [1P-139]

### The drinking behavior for sweet taste solutions affected by pre-exposure to them in water-deprived rats

Izumi Manabe[2], *Maho Yamazaki[1,2], Shinpei Takahashi[1], Shusuke Iwata[1], Toshiaki Yasuo[1], Takeshi Suwabe[1], Satoshi Kawano[2], Noritaka Sako[1] (*[1]Department of Oral Physiology, Asahi University School of Dentistry, [2]Department of Endodontics, Asahi University School of Dentistry*)

[Introduction]Do we choose more preferable taste solution even if we are in a state of water deprivation? In such situation, do we ignore taste information of drinking water to recover from thirsty? In the present study, we conducted behavioral studies using Wistar/ST rats to make clear this question. [Methods]Rats were divided into naive and pre-exposed groups. In naive group, water-deprived rats were trained to drink distilled water (DW) in two bottles for 10 min/a day in the training period (first 5 days). Following 5 days were test days, these rats underwent short-term (10 min) two bottle preference test for 5mM sodium saccharin (Sacc) vs DW. In pre-exposed group, rats also underwent same type of short-term (10 min) two bottle preference tests after 3 sets of long-term (48 hours) two bottle preference test for 5mM Sacc vs DW.This study was approved by "The Animal Care and Ethics Committee of Asahi University" (Approval No. 23-049).[Results and Summary] Naive rats drank DW more than Sacc without concern for its taste during the first 3 days of the short-term test period. But, on the following 2 test days, Sacc were consumed more than DW. On the other hands, in pre-exposed group, there was no significant difference between consumed volume of Sacc and DW during the all 5 test days of the short-term test period.These results suggest following speculations;(1) When naive rats are in a state of water deprivation, they choose drinking water without concern for its taste to recover from thirsty.(2) By experience of drinking Sacc on some days, the naive rats can choose more preferable drinking water. (3) Even if rats are pre-exposed of Sacc, they do not choose it. This fact may show that rats recognize Sacc dose not bring about post-ingestive effect, such as increasing of blood glucose and calorie.

### [1P-140]

### Understanding the Mechanisms of Microglial Dynamics and the ATP/Adenosine System in Controlling Aggressive Behavior Using the Zebrafish Model

*Ryohei Umeda[1,2], Akinobu Sohda[2], Shinichiro Kume[4], Hitoshi Teranishi[2], Kenshiro Shikano[2], Takatoshi Hikida[3], Reiko Hanada[2] (*[1]Department of Advanced Medical Science, Faculty of Medicine, Oita University, [2]Department of Neurophysiology, Faculty of Medicine, Oita University, [3]Laboratory for Advanced Brain Functions, Institute for Protein Research, Osaka University, [4]Department of Pathophysiology, Faculty of Medicine, Oita University*)

Aggressive behavior is a crucial evolutionary trait across diverse animal species, serving to protect oneself, offspring, and resources. However, heightened aggression has emerged as a societal concern, linked to symptoms of various mental disorders. The central role of the raphe nucleus in the serotonin circuit in modulating aggressive behavior has been reported due to the serotonin neurotransmitter's influence. In addition, microglia have been identified as inhibitors of aggressive behavior. Although microglia are known to migrate and induce inflammation in response to ATP as one of damage-associated molecular patterns (DAMPs), However, physiological mechanisms of ATP-mediated aggressive behavior were not clear. Studies in rats have indicated that adenosine (Ado), a metabolite of ATP, enhances aggressive behavior through Ado receptors, suggesting the potential significance of the microglia-mediated ATP/Ado system in aggressive behavior. Therefore, this study aiming to elucidate how microglial dynamics and the ATP/Ado system mechanistically regulate aggressive behavior to create and analyze an aggressive behavior zebrafish model.This study utilizes a zebrafish model to create and analyze a paradigm for aggressive behavior, aiming to unravel the mechanistic intricacies of how microglial dynamics and the ATP/Ado system regulate aggressive behavior. At first, we have performed the mirror test for evaluating and quantifying aggressive behavior, next we analyzed neurotransmitter dynamics during induced aggression at the sometime we confirmed neural activity in pivotal brain regions (especially the raphe nucleus and central nucleus of the hypothalamus) using transgenic zebrafish expressing neuron-specific GCaMP6f (ELAVL3:GCaMP6f), investigation of the spatial distribution of microglia using transgenic zebrafish expressing enhanced green fluorescent protein (EGFP) specifically in microglia (mpeg1:EGFP). These data elucidate the role of microglial dynamics through the ATP/Ado system in aggressive behavior regulation.

### [1P-141]

### Discovery and analyses of a novel mechanism of sleep regulation that depends on feeding condition

*Hibiki Okamura[1,2], Shimpei Taniguchi[1], Mitsuaki Kashiwagi[1,3], Shinnosuke Yasugaki[1,3], Mika Kanuka[1], Masashi Yanagisawa[1], Yu Hayashi[1,3] (*[1]WPI-IIIS, Univ. of Tsukuba, [2]Ph. D. Prog in Humanics, SIGMA, Univ. of Tsukuba, [3]Dept of Biol Sci, Grad Sch of Sci, Univ. of Tokyo*)

Our sleep comprises two states: rapid eye movement (REM) sleep and non-REM (NREM) sleep. NREM sleep is crucial for memory consolidation (Rasch et al., Science, 2007) and secretion of growth hormone (Takahashi et al., J Clin Invest, 1968). While the physiological function of REM sleep is less understood, the cerebral blood flow upsurge during REM sleep might promote the clearance of metabolites from brain (Tsai et al., Cell Rep, 2021). Furthermore, in humans, low amount of REM sleep is associated with a high risk of dementia (Pase et al., Neurology, 2017). NREM sleep is also reduced in patients with Alzheimer’s disease (Vitiello et al., J Gerontol, 1990). Thus, although the causal relationships are yet unclear, these human studies suggest that the amount of sleep is important for maintaining our health. However, it is currently difficult to control the sleep amount over a long term. Here, we serendipitously found that a certain type of diet can alter the amount of both NREM sleep and REM sleep in mice. In addition, switching back from this diet to a normal diet reversed the amount of sleep to normal levels. We also found that this feed condition-dependent change in sleep is largely enhanced by disrupting certain neuronal circuits. We are now trying to elucidate the underlying mechanisms. This study contributes to the understanding of a novel regulatory system of sleep that depends on the feeding condition. Moreover, it may provide clues for the development of methods to regulate the amount of sleep in humans.

### [1P-142]

### Strain differences in aggressive behavior induced by social isolation during the developmental period in rats

*Yu Nishimura[1], Dewi Mustika[1], Shinya Ueno[1], Naoki Tajiri[1], ChaGyun Jung[1], Hideki Hida[1] (*[1]Dept Neurophysiol & Brain Sci, Nagoya City Univ Grad Sch Med Sci*)

We previously reported that oral ingestion of monosodium glutamate (MSG), an umami substance, during development reduced aggression in the SHR strain, a rat model of attention deficit hyperactivity disorder. However, the previous methodology is not sufficiently reproducible to induce aggression constantly; it is crucial to reconfirm animal model conditioning. Therefore, we conducted a study to compare the aggressive behavior of different rat strains.Five different rat strains (Long-Evans, WKY/IZM, SHR/IZM, SHR-SP/IZM, and SHR-SP/EZO) were housed individually for 5 weeks from 25 days of age, immediately after weaning, to provide social isolation during development. Aggression was then assessed using the resident-intruder test; Wistar/ST rats weighing 0 – 30 g less than the resident rats were used as intruders. The frequency, duration, and latency of anogenital sniffing, aggressive grooming, and attack behavior for 10 min per day in the dark phase were analyzed for 3 consecutive days. Immunohistochemistry of c-Fos expression was then conducted on all strains to predict potential aggression-related brain areas.The resident-intruder test during the dark phase revealed that the intruder reliably induced aggressive behavior in the SHR/IZM. Among the five strains, SHR/IZM were the most aggressive in all aggression parameters: SHR/IZM exhibited a significant increase in both the number of attacks and the total attack time, and attack latency was significantly decreased in SHR/IZM compared to the other strains.SHR/IZM also showed increase in the number of c-Fos positive cells of aggression-related brain areas including the prefrontal cortex, lateral hypothalamus, and central amygdala. These data suggest that SHR/IZM are suitable for further examination of the effects of MSG on aggression.

### [1P-143]

### Kamikihito showed oxytocin receptor agonist action and improved social memory in oxytocin gene-deficient mice.

*Shizu Hidema[1], Katsuhiko Nishimori[1], Yuko Maejima[1], Keita Mizuno[2], Kenju Shimomura[1] ([1]*Fukushima Medical University,* [2]*Tsumura Kampo Research Laboratories*)

Nonapeptide oxytocin (Oxt) is known as neuromodulator and neurotransmitter, and has been revealed to play essential roles in various types of social behaviors, such as feeding behavior, anxiety, stress response and thermoregulation in CNS. Oxt neurons extend axons from PVN and SON to various parts of the nuclei expressing Oxt receptor (Oxtr) and release Oxt from their synaptic terminals. Oxt exerts various physiological functions via the Oxtr. Kamikihito (KKT) is a traditional Japanese medicine used to treat insomnia, anemia, anxiety and neurosis. Pharmacological studies have shown that KKT is effective for behavioral abnormalities and cognitive dysfunction, suggesting that KKT has effects on CNS. Recently we reported that KKT functions as oxtr agonist, and could directly activate OXT neurons via Oxtr in PVN and induced Oxt secretion In this study, we focused on the effect of KKT on social behavior of model animal. *Oxt gene*-deficient mice (Oxt KO) and *Oxtr gene* deficient mice (Oxtr KO) are known to Autism Spectrum Disorder (ASD) model mice with impaired social memory. The social memory of *both lines* was not affected by a single dose administration of KKT, but only Oxt KO improved by continuous administration of KKT for 14 days. These data suggest that KKT would have ability of agonist for Oxt. We're continuing to analyze the neural activation, especially in the regions related to social memory in the brain, to understand the pharmacological and physiological mechanisms about the amelioration of social memory observed after the continuous administration of KKT to the tested mice.

### [1P-144]

### Circadian rhythm of maternal behavior and pattern change of feeding behavior in dams raising pups in mice

*Atsumi Murakami[1], Hitoshi Okamura[3], Keiko Tominaga[1,2] ([1]*Graduate School of Science, Osaka university,* [2]*Graduate School of Frontier Biosciences, Osaka university,* [3]*Graduate School of Medicine, Kyoto university*)

Most organisms on the earth show circadian rhythms (about 24-hour rhythmicity) in their physiological processes and behaviors. Because mice are nocturnal, they are active during the dark phase in the 12-hour light: 12-hour dark (LD) cycle and rest in the light phase. However, dams raising pups exhibit decreased behavioral activity and an unclear activity rhythm. On the other hand, dams during raising pups must perform an important activity of maternal behavior for pups' survival, in addition to their food intake, drinking water, and spontaneous activities.

In this study, we focused on maternal behavior and analyzed its circadian rhythmicity. We observed the crouching behavior, which is the posture covering pups for lactating and maintaining pups' body temperature. Since dams show crouching posture several weeks from delivery until weaning, this is suitable for an index to observe the circadian rhythm of maternal behavior. Wildtype dam exhibited diurnal rhythm in crouching behavior under LD cycles and its circadian rhythm under constant darkness (DD). In contrast, *Per*-null ($Per1^{-/-}$, $Per2^{-/-}$, $Per3^{-/-}$) dam did not exhibit such rhythmicity in crouching behavior under the LD and DD, indicating that maternal behavior is regulated by the circadian clock. We also observed dams' feeding behavior during raising pups. Non-lactating female mice feed mostly in the dark phase because they are nocturnal. But dams ate half of the daily food in the light phase, therefore feeding pattern was altered. Since it is known that peripheral clocks are affected by feeding timing, we measured the expression level of clock genes in a dam's liver by Real-time quantitative PCR, and compared it with that of virgin mice. Some clock genes such as Per2 and Rev-erbα were drastically dampened. Moreover, the daily expression rhythm of metabolism enzymes, which are regulated by clock genes, was also significantly changed. On the other hand, in the SCN, no significant alterations in the expression rhythm of clock genes were observed.

From these results, it is clarified that crouching behavior in dams raising pups shows robust circadian rhythmicity, although spontaneous activity is decreased and feeding pattern is changed. Furthermore, significant alteration in the expression rhythm of clock genes and metabolism enzymes in the liver, but not in the SCN, indicates that the circadian rhythm of liver metabolism is disrupted in dams raising pups due to the change of the feeding pattern.

### Poster

### [1P]
### Pathophysiology

March 28, 13:00 - 14:20, Poster Room

### [1P-145]

### Overexpression of ROCK2 promotes Epithelial-to-Mesenchymal Transition (EMT) and metastasis in prostate cancer

*Alamgir Hossain, Aya Yamamura[1], Md Junayed Nayeem[2], Rie Takahashi[1], Motohiko Sato[1] ([1]*Department of Physiology, School of Medicine, Aichi Medical University, Aichi, Japan,* [2]*Department of Cardiac Physiology, National Cerebral and Cardiovascular Center Research Institute, Suita, Osaka, Japan*)

Rho-associated kinases (ROCKs) are serine-threonine protein kinases that exhibit two isoforms – ROCK1 and ROCK2 in humans. Aberrant expression of ROCKs has been reported in many human diseases like cardiovascular disease, neurological disorders, and several cancer types. However, clinical applications of ROCK inhibitors have been limited to only a few disease conditions. To evaluate their therapeutic potential in other diseases, extensive study is warranted . Overexpression of ROCKs in prostate cancer (PCa) has been reported in recent studies. However, their role in PCa pathogenesis has not been well investigated. We sought to investigate the role of ROCKs in PCa in our study. First, we checked expression of ROCK1 and ROCK2 mRNA and protein in normal prostate cancer cell line - PrEC and two prostate cancer cell lines, PC-3 and DU-145 by qRT-PCR and western blotting respectively. Both ROCK1 and ROCK2 expressions were found higher in cancer cell lines compared to normal cells. ROCK2 expression was notably higher in PC-3 cells than ROCK1. To understand the role of ROCKs in PCa pathogenesis, we knock down expression of ROCK1 and ROCK2 in PC-3 cells with small interfering RNAs (siRNAs) and looked for the changes in epithelial to mesenchymal transition (EMT) marker proteins. Knockdown of ROCK1 and ROCK2 resulted in decreased expression of EMT marker proteins - N-cadherin, Vimentin, and snail in PC-3 cells. Inhibition of ROCK2 by KD025 also resulted in decreased expression of N-cadherin and snail in PC-3 cells. To see the role of ROCK2 in PC-3 viability and migration, we performed cell viability and transwell migration assay. Both knockdown and inhibition of ROCK2 significantly reduced viability and migration of PC-3 cells. Together these results demonstrated the role of ROCK2 in PCa pathogenesis and suggested the potential of ROCK2 as a therapeutic target in PCa treatment.

### [1P-146]

### Central regulation of colorectal motility are altered in a rat model of Parkinson's disease

*Tomoya Sawamura[1], Kazuhiro Horii[1,2], Natsufu Yuki[1], Takahiko Shiina[1,3], Yasutake Shimizu[1,3] ([1]*Laboratory of Physiology, Joint Graduate School of Veterinary Sciences, Gifu University,* [2]*Division of Biological Principles, Department of Physiology, Graduate School of Medicine, Gifu University,* [3]*Laboratory of Physiology, Joint Department of Veterinary Medicine, Faculty of Applied Biological Sciences, Gifu University*)

We have previously shown that the central nervous system plays an important role in the regulation of colorectal motility. We hypothesized that disorders of central nervous system might be involved in defecation disorders due to disturbance of central regulation of colorectal motility. In this study, we focused on Parkinson's disease (PD). PD is defined by the loss of dopaminergic neurons in the substantia nigra pars compacta (SNc). Constipation is often associated with PD, but the causal relationship between the loss of dopaminergic neurons and constipation has not been elucidated. In this study, we aimed to elucidate whether the regulation of colorectal motility by the central nervous system is altered in the PD model rats. PD model rats were generated by injection of 6-hydroxydopamine (6-OHDA) into the right medial forebrain bundle to eliminate dopaminergic neurons in the unilateral SNc in male SD rats. The establishment of PD model was confirmed by rotational movements induced by intraperitoneal administration of apomorphine 2 weeks after administration of 6-OHDA. Colorectal motility was measured by in vivo in anesthetized PD rats 3 weeks after administration of 6-OHDA. In sham control rats, noxious stimuli in the colorectum enhanced colorectal motility. The enhanced motility was abolished by administration of a dopaminergic receptor inhibitor and serotonergic receptor inhibitors into the L6-S1 spinal cord, indicating that dopamine and serotonin released in the spinal defecation center plays an important role in the enhancement of colorectal motility. In contrast, noxious stimuli failed to enhance colorectal motility in PD rats. When $GABA_A$ receptor inhibitor, bicuculline, was administered into L6-S1 spinal cord, colorectal motility was enhanced in response to noxious stimuli even in PD rats. The noxious stimuli-induced colorectal motility under $GABA_A$ inhibition was abolished by administration of the dopaminergic receptor inhibitor, but not the serotonergic receptor inhibitors, into the L6-S1 spinal cord. These findings suggest that the disruption of nigral dopaminergic neurons alters component of the neurons related to regulation of colorectal motility; that is, serotonergic pathway becomes inoperative whereas GABAergic pathway becomes functional. Considering that GABA competes stimulatory action of monoamines on the spinal defecation center, the alteration of the neural components would be attributable to constipation in PD patients. The authors have no COI to disclose.

### [1P-147]

### Prolonged symptoms after COVID-19 infection and their physiological mechanisms

*Kanae Kobayashi[1], Takuya Yamazaki[2], Aiko Abe[1], Michiko Shoji[1], Itsuro Kazama[1] ([1]*Miyagi University, School of Nursing,* [2]*Miyagi University, School Affairs Division*)

In young adults with coronavirus disease 2019 (COVID-19), symptoms are usually mild and spontaneously disappear within a week. However, some patients experience long-term effects from their infection, commonly known as Long COVID or Post-COVID Conditions. According to some reports, people who were tested positive for COVID-19 usually develop common symptoms, such as high fever, sore throat, headache, cough and generalized fatigue. Additionally, some of them develop neuropsychiatric manifestations, such as short-term memory loss, inability to concentrate and depression. In most patients, fever, headache and sore throat subside within a week or so. However, the neuropsychiatric manifestations tend to persist much longer, lasting more than one month or two. Since the viral load decreases within two weeks after COVID-19 infection, the prolonged symptoms are considered to be attributable to chronic inflammation or enhanced immunological response. In our patch-clamp studies, the activity of lymphocytes was detected electro-physiologically by the delayed rectifier $K^+$-channel (Kv1.3) currents. Recent studies indicate that leukocytes, such as lymphocytes and microglia, are responsible for the pathogenesis of chronic inflammation. Therefore, the activity of these leukocytes was thought to be involved in the mechanisms of prolonged neuropsychiatric manifestations after COVID-19 infection.

### [1P-148]

### Effects sodium bicarbonate on hyperkalemia-induced ECG abnormalities in bullfrog hearts

*Saya KAZAMA Azuma[1], Ryo Kuwana[1], Ken Narisawa[1], Kanae Kobayashi[1], Itsuro Kazama[1] ([1]*Miyagi University, School of Nursing*)

Hyperkalemia is caused by excessive intake or ineffective elimination of potassium ions ($K^+$), or their excessive release from skeletal muscles. It is characterized by typical electrocardiogram (ECG) findings, such as peaked T waves and the widening of QRS complexes. In the present study, we injected potassium chloride (KCl) solutions (1, 10 and 100 mM) intravenously into bullfrogs, thus demonstrating the characteristic ECG abnormalities of hyperkalemia in frog hearts. The widened QRS complexes induced by 100 mM KCl injection were accompanied by an increase in the resting membrane potential in cardiomyocytes and a decreased slope of phase 0 in the action potential. Recording both ECG waveforms and the cardiac action potential enabled us to reveal the mechanisms of hyperkalemia-induced ECG abnormalities. Additionally, pre-treatment with sodium bicarbonate or salbutamol, a direct or indirect stimulator of $Na^+/K^+$-ATPase activity, significantly accelerated the recovery from the widened QRS complexes in the ECG, demonstrating a pronounced shift of extracellular $K^+$ ions into the intracellular space.

### [1P-149]

### Plasminogen activator inhibitor-1 is involved in the angiogenesis decreased by glucocorticoids during bone repair in mice

*Yuto Niwa[1], Kiyotaka Okada[2,1], Takashi Ohira[1], Yuya Mizukami[1], Naoyuki Kawao[1], Osamu Matsuo[1], Hiroshi Kaji[1] ([1]*Departments of Physiology and Regenerative Medicine,, Faculty of Medcine, Kindai University,* [2]*Departments of Arts and Sciences, Faculty of Medcine, Kindai University*)

Glucocorticoid excess induces osteoporosis and delayed fracture healing. Plasminogen activator inhibitor-1 (PAI-1), a principal inhibitor of plasminogen activators, is an adipocytokine that regulates bone metabolism. We previously showed that PAI-1 is involved in osteopenia and delayed bone repair induced by glucocorticoids in mice. However, roles of angiogenesis in glucocorticoid-induced delayed bone repair have not been clarified. It has been recently well recognized that angiogenesis of both CD31 and endomucin-positive type H vessels is crucial for bone repair after bone injury. We herein investigated the roles of PAI-1 and type H vessel formation in glucocorticoid-induced delayed bone repair after femoral bone injury using PAI-1-deficient female mice. Intraperitoneal administration of dexamethasone (Dex) significantly decreased the number and area of type H vessels as well as CD31-positive vessels at the damaged sites 4 days after femoral bone injury. PAI-1 deficiency significantly attenuated type H vessel formation as well as the mRNA and protein levels of vascular endothelial growth factor (VEGF) and hypoxia inducible factor-1α (HIF-1α) suppressed by Dex at the damaged sites 4 days after bone injury. It also significantly attenuated transforming growth factor-β (TGF-β) and bone morphogenetic protein-2 (BMP-2) mRNA levels decreased by Dex at the damaged sites. In conclusion, we herein demonstrated that Dex decreased the angiogenesis of type H vessels at the damaged sites during bone repair after femoral bone injury partly through PAI-1 in mice.

### [1P-150]

### Prevention of neuroinflammation by a dopamine D1-like receptor agonist ameliorates overall survival of sepsis model mice

*Rintaro Shinabe[1], Koichi Tanaka[2,3], Mohammed E Choudhury[1], Kanta Mikami[1], Jun Takeba[3], Junya Tanaka[1] ([1]*Department of Molecular and Cellular Physiology, Ehime University Graduate School of Medicine,* [2]*Advanced Emergency and Critical Care Center, Ehime Prefectural Central Hospital,* [3]*Department of Aeromedical Services for Emergency and Trauma Care, Ehime University Graduate School of Medicine*)

### Poster

### [1P]
### Sensory function

March 28, 13:00 - 14:20, Poster Room

### [1P-151]

### A recurrent cortical circuit triggers somatosensory perception

*Yasuhiro Oisi[1], Yusuke Atsumi[1], Yoshiki Ito[1], Saito Yoshihito[1], Hiroyuki Uwamori[1], Maya Odagawa[1], Takayuki Suzuki[1], Chie Matsubara[1], Shigeki Kato[2], Kazuto Kobayashi[2], Kenta Kobayashi[3], Midori Kobayashi[1], Atsushi Kobayashi[4], Kanako Ueno[1], Masanori Murayama[1] (*[1]RIKEN, [2]Fukushima medical Univ., [3]NIPS, [4]NI*)

We believe that our brain makes us perceive the truth of the external world as it is. But in fact, it does not. When a mosquito perches on your leg, you may sometimes perceive and chase it away, but sometimes you do not perceive and suffer from itch afterward. How does the brain perceive stimuli? has been the most fundamental question in the study of perception. Recent theoretical studies have emphasized the important role of long range projections, including feedforward(FF) and feedback(FB) inputs. These theories are being tested by many studies of the neural correlates of perception in monkeys and humans. However, it remains unclear how such hierarchical interactions contribute to perception due to methodological limitations in dissecting and manipulating circuits precisely in time and space in primate research. We have previously reported a recurrent hierarchical circuit consisting of cortical long-range projections between the secondary motor cortex (M2) and the primary somatosensory cortex (S1) in mice (Manita et al., Neuron 2015). Furthermore, somatosensory stimulation sequentially induced activity in S1, M2, and S1 on the recurrent circuit. M2 FB input can trigger dendritic spikes and burst firing in S1 neurons. Based on these results, we hypothesized that the M2 FB projection to S1 contributes to somatosensory perception. Here, we tested this hypothesis using optogenetic, chemogenetic, pharmacological, and lesions of the circuit during a somatosensory stimulus detection task. We defined a perceptual detection threshold in each mouse that performed the behavioral task and investigated how the threshold changes with circuit manipulations. First, we found that S1 and M2 lesions, pharmacological and optogenetic inhibition of each area significantly increased the threshold, indicating impaired perception. Pathway-specific optogenetic and chemogenetic inhibition of both the S1->M2 FF and M2->S1 FB projections also impaired perception. These results suggest that the S1-M2 recurrent circuit contributes to perception via FF and FB inputs. Next, we tested whether activation of either FF or FB projections is sufficient for somatosensory perception. Pathway-specific optogenetic activation of both S1 FF and M2 FB projections was able to induce illusory somatosensory perception. Finally, we investigated which pathway is closely correlated with perception. Pathway-specific activation of M2 FB inputs with pharmacological M2 inactivation was able to induce illusory perception. In contrast, activation of the S1 FB input with pharmacological S1 inactivation impaired perception. These results support our hypothesis that somatosensory perception requires S1 activity that is evoked by recurrent M2 FB inputs.

### Poster Presentation

Day 2
(March 29, 13:00 - 14:20)

[2P] Neurophysiology, Neuronal cell biology - Plasticity
[2P] Neurophysiology, Neuronal cell biology - Neural network
[2P] Neurophysiology, Neuronal cell biology - Neurons, Synapses
[2P] Neurophysiology, Neuronal cell biology - Higher brain function
[2P] Neurophysiology, Neuronal cell biology - Motor function
[2P] Neurophysiology, Neuronal cell biology - Sensory function, Sensory organ
[2P] Molecular physiology, Cell physiology - Ion channels, Receptors
[2P] Molecular physiology, Cell physiology - Others
[2P] Embryology, Regenerative Medicine, Development, Growth, Aging
[2P] Muscle
[2P] Digestion, Digestive system
[2P] Oral physiology
[2P] Blood, Lymph, Immunity
[2P] Circulation
[2P] Urinary organ, Renal function, Urination
[2P] Autonomic nervous system
[2P] Physical fitness and sports medicine
[2P] Nutritional and metabolic physiology, Thermoregulation
[2P] Behavior, Biological rhythm, Sleep
[2P] Stress
[2P] Pathophysiology
[2P] Drug Action, Pharmacology
[2P] Study Methodology

### Poster

### [2P]
### Neurophysiology, Neuronal cell biology
### Plasticity

March 29, 13:00 - 14:20, Poster Room

### [2P-001]

### Postnatal development of Perineuronal Nets in SAMP10 mice

*Hiroshi Ueno[1], Yu Takahashi[2], Motoi Okamoto[3], Takeshi Ishihara[2] ([1]*KawasakiUniversity of Medical Welfare,* [2]*Kawasaki Medical School,* [3]*Okayama University*)

Aging is the most potent risk factor for developing neurodegenerative diseases and has been implicated in important changes in brain tissue. We studied a complex aging model using the senescence-accelerated mouse (SAM) strain. We selected the SAMP10 strains, which reportedly have brain function abnormalities. The extracellular matrix (ECM) molecules in the central nervous system (CNS) are composed of hyaluronic acid, tenascin-R, glycoproteins, and chondroitin sulfate proteoglycans (CSPGs). In the mature CNS, ECM is distributed as neuronal granules or perineuronal nets (PNNs), which are dense mesh-like structures of specialized ECM, covering the soma, proximal dendrites, and axon beginnings of some neurons. Furthermore, PNNs are formed during postnatal development, and their formation in the sensory cortex is considered to abolish synaptic plasticity. A previous study reported a weakly expressed Cat-315-positive PNN in SAMP10 at 3 months of age. No expression of Cat-315-positive PNN was observed in the prefrontal cortex of SAMP10 mice at 12 months of age. However, details of the distribution and developmental processes of PNNs in the somatosensory cortex of SAMP10 mice have not been elucidated. Here, we investigated the development of PNNs in the SAMP10 mouse somatosensory cortex. We found that the density of Cat-315-positive PNNs in the somatosensory cortex of SAMP10 mice decreased during postnatal development compared to senescence-accelerated mouse resistance (SAMR1) mice.

### [2P-002]

### The anterior cingulate cortex plays the important role for stress induced mechanical hypersensitivity

*Ryo Kawabata[1,2], Ayumi Fujita[2], Kohei Koga[2], Ikuko Yao[1] ([1]*Kwansei Gakuin University,* [2]*Department of Neurophysiology, Facility of Medicine, Hyogo Medical University, Hyogo, Japan*)

Stress is categorized into various types such as physical, psychological and social. Almost of all stress can influence on animal and human behaviors. Exposure of stress alters sensation and leads to form negative emotion including anxiety and depression. Recent our study showed acute physical stress induced by elevated open platform (EOP) caused prolonged mechanical hypersensitivity. Furthermore, we showed the EOP stress induced synaptic plasticity in layer II/III pyramidal neurons from the anterior cingulate cortex (ACC) that brain region related to pain and negative emotions. However, it is still unclear whether the ACC is involved in the EOP induced mechanical hypersensitivity, and how the EOP alter both spontaneous and evoked excitatory synaptic transmission in layer V pyramidal neurons in the ACC. In this study, we first examined if the ACC was involved in the hypersensitivity by the EOP for 30 min. We injected ibotenic acid into the ACC to lesion the neurons of the ACC. We conducted von Frey test before and after the EOP to measure mechanical thresholds. Next, by using whole-cell patch-clamp recording from brain slice preparation, we analyzed spontaneous and evoked synaptic transmissions from layer V pyramidal neurons within the ACC. Furthermore, we investigated electrophysiological property such as action potentials (APs) in the ACC. The EOP altered spontaneous excitatory postsynaptic currents (sEPSCs) and evoked EPSCs (eEPSCs), but did not change intrinsic neuron properties. Intriguingly, the mice exposed in the EOP produced abnormal neurotransmitter release on excitatory synapses in the ACC. These results suggest that the ACC play a critical role to modulate stress-induced mechanical hypersensitivity, and the ACC represents synaptic plasticity mainly on excitatory transmission in the ACC.

### [2P-003]

### Forced running after stroke promotes dendritic spine density in the motor cortex and reach performance in mice.

*KAYO NAKAMURA[1] ([1]*Toyohashi sozo University*)

Dendritic structural plasticity involving morphological change and turnover of spines has been known an essential mechanism of the learning and memory. This plasticity can be manifested as altered synapse strengthening to the activity-dependent modification. On the other hand, in motor paralysis after stroke, the learning that regains damaged motor function has to be thought optimally modify surviving neuronal networks for activity-dependent. However, it is still unknown how to do training to optimally surviving neuronal networks that compensate for function lost after brain injury. In this study, we used the common method that mice were injected a photosensitive dye solution Rose Bengal (10mg/kg, in PBS), and then the green laser illumination (532nm) was turned on for 30 min at the motor area to induce localized ischemia. Mice learned reach-to-grasp task during 8 days before surgery of stroke. After stroke, mice lost their learned reaching performance before undergoing surgery. Training after stroke was performed by forced running in a motorized basket for 30 min of the speed of rotations at 1.5 to 3.0 m/min between day 3 to day 10. Our results showed that reach performance of forced running mice after stroke significantly increase compared with Non-training mice at day 14. In order to confirm the neuronal plasticity, we visualized the dendritic spines using immunostaining and Golgi-Cox staining. Its morphology was identified from layer 2/3 and layer 5 pyramidal neurons in motor cortex. In the result, dendritic spine density of pyramidal neurons in training mice tend to increase compared with Non-training mice. Interestingly, training mice showed increase activity neurons compared with control mice. As a result, forced running mice showed more rapid recovery of motor function of forelimb compared to Non-training mice. Our data indicates that increased spine density by running mice after stroke might enhance reach learning in mice.

### [2P-004]

### CRSIPR/Cas9 based screening of molecules underlying axon initial segment plasticity

*Yixuan Du[1], Ryo Egawa[1], Hiroshi Kuba[1] ([1]*Cell Physiology, Graduate School of Medicine, Nagoya University*)

The axon initial segment (AIS), the site of action potential initiation, modulates neuronal excitability through plastic changes in response to synaptic inputs. In nucleus magnocellularis (NM) of the chicken, an avian homologue of mammalian anteroventral cochlear nucleus, the characteristic frequency-dependent shortening of the AIS occurs during the development. Our group revealed that this kind of shortening involves the modulation of microtubule dynamics by CDK5/p35. However, the signaling pathways downstream of CDK5/p35 and molecules that regulate microtubule stability within the AIS compartment are still poorly understood.

In this study, we performed the knockout (KO) screening by using in-vivo genome editing to search for molecules involved in the AIS shortening in NM cells. A plasmid vector that cleaves three sites of a single target gene by CRISPR/Cas9 was generated and introduced into NM progenitor cells at embryonic day 1.5 (E1.5) by *in ovo* electroporation. Brainstems were isolated at E21, when the AIS is supposed to show significant shortening, and cryo-sectioned into 50μm-thick slices, immunostained and imaged under a confocal microscopy.

20 molecules have been screened and eight of them altered the AIS length by KO in NM cells at E21. Among those showing AIS elongation, the effects were remarkable in the KO of GSK3β, a substrate of CDK5, and MACF1, a substrate of GSK3β as well as a cross-linker between F-actin and microtubules. Furthermore, the overexpression of constitutively active mutant of GSK3β shortened the AIS. These results suggest that GSK3β and MACF1 would be promising candidates mediating the auditory-input-dependent AIS shortening.

### [2P-005]

### Contextual learning requires rapid Ser$^{408\text{-}409}$ phosphorylation of the GABA$_A$ receptor β$_3$ subunit and the plasticity at inhibitory CA1 synapses

*Yuya sakimoto Sakimoto[1], Yuheng Yang[1], Dai Mitsushima[1], Hiroyuki Kida[1]
([1]*yamaguchi university, department of medicine*)

Contextual learning requires plasticity at both AMPA receptor-mediated excitatory and GABA$_A$ receptor-mediated inhibitory synapses in CA1 neurons, but detailed mechanisms of the learning-induced plasticity at GABA$_A$ receptor-mediated synapses have been unclear. We previously reported that the training for inhibitory avoidance (IA) task increased post-synaptic number of GABA$_A$ receptors (Sakimoto et al. Cereb Cortex 2019) and rapid phosphorylation of intracellular loop (Ser$^{408\text{-}409}$) of GABA$_A$ receptor β$_3$ subunits (Sakimoto et al FASEB J 2019). To further examine the causal relationship between the Ser$^{408\text{-}409}$ phosphorylation, GABAergic plasticity, and learning, we synthesized a novel peptide-based phosphorylation inhibitor targeting the Ser$^{408\text{-}409}$ site using a cell-permeable HIV-tagged peptide (Tat pep β$_3$-SS). First, to confirm the cell-specific effects of the peptide, we microinjected Tat-pep β3-SS-FITC into the CA1 and successfully prevented training-induced increases in inhibitory synaptic currents in tagged CA1 neurons, whereas a site-specific mutation control (Tat-pep β3-AA-FITC) showed no effect. Moreover, the injection of Tat-pep β$_3$-SS but not Tat-pep β$_3$-AA into the CA1 attenuated the rapid phosphorylation of Ser$^{408\text{-}409}$, postsynaptic Cl- current of GABA$_A$ receptors, and the expression of the postsynaptic GABA$_A$ receptor β$_3$ subunit after the training. Finally, we bilaterally microinjected the peptide into the CA1 under the freely-moving condition 60 min before the IA training. Tat-pep β$_3$-SS but not Tat pep β$_3$-AA clearly impaired the learning performance without changing sensory or motor functions. These results suggest a causal link between Ser$^{408\text{-}409}$ phosphorylation, GABA$_A$ receptor-mediated synaptic plasticity, and contextual learning. While traditional Hebbian plasticity has been theorized based on excitatory synapses, this study provides new evidence that contextual learning requires GABA$_A$ receptor-mediated plasticity at inhibitory synapses.

### Poster

### [2P]
### Neurophysiology, Neuronal cell biology
### Neural network

March 29, 13:00 - 14:20, Poster Room

### [2P-006]

### Synaptically induced translocation of protein kinase C γ in cerebellar Purkinje cells

*Nobutake Hosoi[1], Yuuki Fukai[1], Ayumu Konno[1], Hirokazu Hirai[1] ([1]*Department of Neurophysiology and Neural Repair, Gunma University Graduate School of Medicie*)

Among the central nervous system, protein kinase C (PKC), which is activated by $Ca^{2+}$ and DAG, is expressed most highly in the cerebellum. The gamma isoform of PKC (PKCγ) is expressed dominantly and exclusively in Purkinje cells (PCs), accounting for over 95 % of classical PKC subtypes in adult PCs. Resting PKCγ is localized in the cytoplasm of PC dendrites and soma. After its activation, PKCγ translocates to the plasma membrane. However, the physiological conditions that induce PKCγ translocation remain largely unknown in cerebellar PCs. In the present study, we virally expressed GFP-tagged PKCγ (PKCγ-GFP) specifically in adult PCs using the PC-specific L7-6 promoter, and examined which pattern of synaptic inputs (parallel fiber input or climbing fiber input) induced its translocation at PC dendritic shafts by current clamp recording combined with confocal GFP fluorescence imaging. A single or repetitive (150 pulses at 5 Hz for 30 s) activation of a climbing fiber (CF), which produced the complex spike in PC, failed to induce translocation of PKCγ-GFP to the cell membrane. Direct current injection (+2 nA for 3 s) to PC did not induce the translocation either, although PCs generated simple spikes continuously at high rates. In contrast, high-frequency parallel fiber (PF) stimulation (50 pulses at 50 Hz for 1 s), which triggered action potentials followed by sustained depolarization (presumably mGluR1-mediated slow depolarization), caused translocation of cytoplasmic PKCγ-GFP to the cell membrane. Low-frequency PF stimulation (150 pulses at 5 Hz for 30 s) induced continuous simple spike firing but did not induce translocation. These results suggest that CF-triggered depolarization, which causes $Ca^{2+}$ influx through voltage-gated $Ca^{2+}$ channels throughout PC dendrites and somas, is insufficient to induce the translocation of PKCγ. Instead, high-frequency PF stimulation, which can activate mGluR1, can lead to PKCγ translocation at PC dendrites.

### [2P-007]

### P2X3 receptors modulate the transmission of visual information in the retina.

*Toshiyuki Ishii[1], Atsushi Shimohata[1], Tomomi Shimogori[2], Makoto Kaneda[1] ([1]*Nippon Medical School*, [2]*RIKEN*)

First, we investigated the localization of the P2X3 receptors. In the adult mouse retina, P2rx3 mRNA was expressed in the ganglion cell layer (GCL), and the immunoreactivity for P2X3 receptor was found in the inner plexiform layer and GCL. When we examined the cell type expressing P2X3 receptor, P2X3 receptor signals coincided with some GABA or RBPMS immunoreactive cells, suggesting that P2X3 receptors are expressed in amacrine cells or RGCs. We next examined the physiological function of P2X3 receptor in the retina. In electroretinogram, intravitreal injection of A317491, an antagonist of P2X3 receptor, significantly decreased the amplitude of oscillatory potentials, which reflects the activity of amacrine cells. In addition, the responses to a,b-MeATP, an agonist of P2X3 receptor, were found in some ON- RGCs but not in OFF-RGCs. Next, to examine whether P2X3 receptor contribute to the retinal output, we recorded the activity of RGCs using multielectrode array under light stimulation. Application of A317491 modulated the firing rate of ON- and OFF-RGCs in a different manner. In ON-RGCs, some cells showed increased or decreased firing rates, whereas in OFF-RGCs, most of the cells that changed their firing rate had a decreased firing rate. To elucidate the mechanism by which A317491 increased the firing rate of ON-RGCs and decreased the firing rate of OFF-RGCs, we recorded postsynaptic currents in RGCs during light stimulation. The action of A317491 was increased evoked EPSCs in ON-RGCs but decreased them in OFF-RGCs. Those actions of A317491 to the ON- and OFF-RGCs were occluded by GABA or glycine receptor antagonist. These results suggest that the P2X3 receptors physiologically work for the visual information processing through amacrine cells and RGCs in the mouse retina.

### [2P-008]

### To-and-fro oscillatory dynamics in salience network of rats

*Hiroshi Yoshimura[1], Yoko Tominaga[2], Yusaku Maeda[3], Takeru Matsuda[3], Fumiyasu Komaki[3], Takashi Tominaga[2] ([1]*Institute of Biomedical Sciences, Tokushima University Graduate School*, [2]*Institute of Neuroscience, Tokushima Bunri University*, [3]*Department of Mathematical Informatics, Graduate School of Information Science and Technology, The University of Tokyo*)

Salience network (SN), one of large-scale networks in the brain, is considered to be involved in dynamic switching between default mode network and central executive network. The SN is composed of two distributed nodes, one is anterior insular (AI) and the other is anterior cingulate cortex (ACC). The ACC is involved in attention allocation, reward anticipation, decision-making, and the AI is involved in interoceptive awareness, cognitive function, social emotion. The two nodes are intrinsically connected, and switched on by sensory input. However, intrinsic signal dynamics in the SN has been unclear. Recent studies demonstrated that rodents also have SN, and the SN play a similar function to the human SN. Therefore, in an attempt to clarify the SN dynamics, we visualized large-scale events in the SN of rat brain slices, using optical recording methods with voltage sensitive dye, and analyzed how waves behave spatiotemporally. In order to expose functional large-scale network, gabazine, GABA-A receptor antagonist, was applied to extracellular medium. Somatosensory cortex was activated by low frequency electrical stimulation. After a while, to-and-fro signal traveling between the AI and the ACC was induced, in which signal bounced backed for several times between the two nodes. Thus, signal input generated large-scale oscillation at 1.3 Hz in the SN. Interestingly, in the node of the ACC, non-damped synchronized oscillations with phase-locked several waves at alpha frequency band were generated in wide area of ventral part of the ACC (Cg2), and bounce-backed signal was originated in upper layer of the Cg2. In the node of the AI, the claustrum is the origin of the bounce-backed signals. These results revealed that, (1) the SN includes device that produce large-scale oscillations, (2) the claustrum and ACC may play a role as network hub during to-and-fro signal communications, (3) ventral part of the ACC may be composed of mesoscopic network that produce phase-locked oscillations, and (4) one of the roles of oscillations may make the output gate open and close.

### [2P-009]

### Addressing the James-Lange theory of emotions regarding the fear responses by selective manipulation of the pathway from superior colliculus toward amygdala

*Kaoru Isa[1], Thongchai Sooksawate[1,2], Kota Tokuoka[1,3], Masatoshi Kasai[1], Sara Karimi[4,1], Sakura Hiramatsu[1], Kenta Kobayashi[5], Tadashi Isa[1,6,7] (*[1]Department of Neuroscience, Graduate School of Medicine, Kyoto University, [2]Fac. Pharmaceutical Sci., Chulalongkorn University,, [3]National Institute of Genetics, [4]Institute for Basic Sciences, Physiology Research Center, Kashan University of Medical Sciences, [5]Section of Viral Vector Development, National Institute for Physiological Sciences, [6]Institute for the Advanced Study of Human Biology (WPI-ASHBi), Kyoto University, [7]Human Brain Research Center, Graduate School of Medicine, Kyoto University*)

The James-Lange theory of emotion in the 19$^{th}$ century proposed that our emotional states emerge in response to physiological reactions elicited by external stimuli. That is, our retrospective interpretation of physical responses to environmental stimuli results in an emotional experience. Recently, a new approach to reveal this theory with a fine technique was reported and this theory was getting a lot of attention (Hsueh et al., 2023). Previously we reported that the selective optogenetic activation of the motor command originating from the motor layers of superior colliculus directed to the cuneiform nucleus on the same side induces innate fear responses including flight behavior in mice (Isa et al. 2020). Furthermore we revealed that the same stimulationy. Here, we found that the stimulation of the pathway from superior colliculus was revealed to form the memory of fearful experiences in the passive avoidance paradigm in mice and it was mediated through the efference copy pathway to the amygdala via the posterior thalamic nucleus triangular. Our results posits that the immediate activation of the amygdala through the efference copy of motor commands is the linchpin behind the induction of retrospective fear, remarkably aligning with the very essence of the James-Lange theory of emotion.

### [2P-010]

### Role of the dorsal peduncular cortex and dorsal tenia tecta in acute-stress-induced pain hypersensitivity

*Takahiro Wakisaka[1,2], Makoto Takemoto[2], Wen-Jie Song[2] (*[1]School of Medicine, Kumamoto University, [2]Department of Sensory and Cognitive Physiology, Graduate School of Medical Sciences, Kumamoto University*)

The neural mechanisms by which stress modulates pain sensitivity are not well understood. A previous study has reported that social defeat stress elicits c-Fos expression in the dorsal peduncular cortex (DP) and dorsal tenia tecta (DTT), two areas not yet explored well in the medial prefrontal cortex (Kataoka et al., 2020). Thus, DP/DTT may be involved in the modulation of pain sensitivity caused by stress. In this study, we investigated the effect of DP/DTT activation on pain sensitivity after acute restraint stress (RS) in naive male C57BL/6J mice by using a chemogenetic approach. We found that 2 hours of RS decreased mechanical pain threshold and increased the number of c-Fos positive cells in DP/DTT. Moreover, chemogenetic activation of DP/DTT decreased mechanical pain threshold. These results suggest that DP/DTT may control the change in pain sensitivity caused by acute stress.

### [2P-011]

### Development of video-oculography for 3D eye rotation including torsional eye movements

*Dai Sakaeda[1], Yoshikazu Shinoda[1], Mayu Takahashi[1] (*[1]Tokyo medical and dental university, Systems Neurophysiology*)

### [2P-012]

### Cortical projections to “fixation zone” and “saccade zone” in monkey superior colliculus.

*Asahi Shimizu[1], Yuriko Sugiuchi[1], Yoshikazu Shinoda[1], Mayu Takahashi[1] (*[1]Department of System Neurophysiology, Graduate School, Tokyo Medical and Dental University*)

### Poster

[2P]
**Neurophysiology, Neuronal cell biology**
**Neurons, Synapses**

March 29, 13:00 - 14:20, Poster Room

### [2P-013]

### Orexin receptor activation induces a slow afterhyperpolarization that results from the calcium-dependent closure of cation channels in serotonergic dorsal raphe neurons

*Masaru Ishibashi[1,2], Atsuo Fukuda[1], Christopher S. Leonard[2] ([1]*Dept. of Neurophysiology, Hamamatsu University School of Medicine,* [2]*New York Medical College*)

Serotonergic (5-HT) dorsal raphe (DR) neurons regulate numerous brain functions including sleep/wake states, circadian phase, reward and mood. Moreover, orexin receptor signaling at 5-HT DR neurons appears critical in the sleep disorder narcolepsy, which emerges following the loss of orexin signaling. We recently reported that in addition to producing a slow depolarization, orexin-A enhances the post-spike afterhyperpolarization (oeAHP), which alters spike encoding by increasing spike frequency adaptation. Mechanistically we found that the oeAHP involved two distinct components that required $Ca^{2+}$ influx. The first was of medium-duration (tau ~ 0.5s) and involved apamin-sensitive SK $Ca^{2+}$-activated $K^{+}$ channels. The second was of longer duration (tau ~ 5s), was apamin-insensitive (termed the ai-oeAHP). Previously, we reported that the ai-oeAHP was mediated by a transient, $Ca^{2+}$-dependent closure of around 50% of the cation channels activated by orexin. However, it remains unclear which cation channels are involved in the ai-oeAHP. In this study we used whole-cell patch clamp recordings in mouse brain slices to investigate the mechanisms and function of this ai-oeAHP. We found that flufenamic acid (FFA), attenuated both the orexin-induced inward current and ai-oeAHP. Moreover, we found that application of M084 (30 uM), a TRPC4/5 selective inhibitor, attenuated the orexin-induced inward current amplitude, without changing the ai-oeAHP amplitude. These results suggest that ai-oeAHP is a novel type of $Ca^{2+}$-dependent slow AHP that is induced by closure of non-TRPC4/5 channels.

### [2P-014]

### The raison d'etre of classical synapses in taste buds.

*Ryusuke Yoshida[1], Kengo Horie[1], Mitoh Yoshihiro[1] ([1]*Dept of Oral Physiol, Fac of Med, Dent Pharmac Sci, Okayama Univ*)

Taste receptor cells exist in taste buds. These cells have been classified into 4 types according to their morphological properties. Among them, Type II cells are known to be sweet, umami, and bitter (and also salty) taste cells because these cells express sweet, umami, or bitter receptors. However, Type II cells do not possess classical synapses with gustatory afferent fibers, but they use "channel synapses" with CALHM1/3 channels. Type III cells are believed to be sour-sensitive taste cells and have a synaptic structure with gustatory afferent nerve fibers. Therefore, taste bud synapses may contribute to sour taste transduction between taste cells and gustatory nerve fibers, but their roles are not yet fully elucidated. In this study, we investigate the roles of taste bud synapses by using transgenic mice lacking the synapse-associated protein, SNAP25, in taste cells. In control mice, SNAP25 was expressed in Type III cells but not in other types of cells. Expression of SNAP25 was not observed in taste bud cells of SNAP25-cKO mice. In addition, the numbers of Type III cells in taste buds were significantly reduced in SNAP25-cKO mice compared to control mice. In SNAP25-cKO mice, 5-ethynil-2'-deoxyuridine (EdU) positive Type III cells were significantly reduced 14 days but not 7 days after EdU administration compared to control mice. In a short-term lick test to analyze the taste sensitivity of mice, SNAP25-cKO mice showed reduced sensitivities to sour tastants but not to other tastants. These results suggest that taste bud synapses are required for the maintenance of Type III cells and the transduction of sour taste information from taste cells to gustatory nerve fibers.

### [2P-015]

### Correlation of M-channel activity and trafficking dynamics in neurons

*Daisuke Yoshioka[1], Yasushi Okamura[1] ([1]*Grad. Sch. of Med., Osaka Univ.*)

M-channels (KCNQ2/3) is one of the most important voltage-gated $K^{+}$ channels which determine neural excitability. In neurons, M-channels are predominantly localized on the axon initial segment (AIS), and their spatial pattern is strongly associated with neural excitability. Therefore, defects in M-channel trafficking cause various neurological disorders including epilepsy. However, the trafficking regulatory mechanism is very complex and remains unclear. M-channels have numerous trafficking regulatory sites which are mainly concentrated in the C-terminal region, including helixes A-D. In contrast, the N-terminal region, including the S1-S6 helix, contains activity regulatory sites that are closely involved in voltage sensing and electro-mechanical (E-M) coupling of M-channels. However, it has not yet been fully verified whether the activity regulation is completely independent of trafficking regulation of M-channels in neurons. In this study, we investigated the AIS localization of low-activity M-channels with mutations in their activity regulatory sites in order to elucidate the relationship between the activity and trafficking of M-channels. As a result, we found that several mutations of the activity-regulatory sites decreased the AIS selectivity of M-channel in a channel activity-dependent manner. Furthermore, detailed analysis of the 3D spatial dynamics of M-channels by single-molecule imaging revealed that mutations mainly affect the lateral diffusion and exocytosis processes of M-channels. In summary, this study is the first to demonstrate that the activity regulatory site of M-channels also plays an important role in trafficking regulation.

### [2P-016]

### Effect of Dopamine on the Activity of Striatal Projection Neuron

*Mami Ando[1], Haruka Fujie[1], Tomoki Sueoka[1], Atsushi Tamura[1,2], Kazuto Kobayashi[3], Makoto Osanai[1,2,4,5] ([1]*Osaka Univ.*, [2]*Tohoku Univ.*, [3]*Fukushima Medical Univ.*, [4]*CiNet, NICT*, [5]*CBS, RIKEN CBS*)

The striatum receives the cortical input and sends the inhibitory output to the other nuclei of the basal ganglia. The projection neurons are divided into two types of neurons, one is the direct pathway neurons expressing the dopamine D1 receptor (D1-MSN), and the other is the indirect pathway neurons expressing the dopamine D2 receptor (D2-MSN). The effects of dopamine on the activity of both types of striatal projection neurons are controversial. We applied multicellular calcium imaging technique to the acute brain slice preparation, and recorded fluorescence changes as the activity of the projection neurons. The responses of D1- or D2-MSN to the stimulation to the cortico-striatal axon bundle were compared before and after administration of dopamine, and D1 or D2 receptor selective agonists. In addition, it is known that the beta wave (13~30 Hz ) oscillations increase at the onset of movement at the motor cortex. So, we recorded the responses of the MSNs to the various frequency stimulation. In the control condition, the activity of D1-MSN, evoked by the stimulation, was larger than that of D2-MSN, suggesting that D1-MSN is more sensitive to the input from cortex than D2-MSN. When dopamine was administered, the activities of D1-MSN was increased, though no significant change was observed in D2-MSN. These results suggested that DA decreased the activity of D1-MSN. Furthermore, with D1 agonist, the activity of D1-MSN was decreased significantly as well, suggesting that DA modification was mediated by D1 receptors. In addition, when applying D2 agonist, the activities of D1-MSN was significantly decreased, even though there was no significant change in D2-MSN. This result suggested that the activation of the D2 receptors on the neurons other than D2-MSN had the inhibitory effect on the D1-MSN. The mechanism is not clear, but it is possible that interneurons may affect, or that the activation of presynaptic D2 receptor may inhibit synaptic vesicle release.

### [2P-017]

### The modulation of functional and trafficking properties of $GABA_A$ receptor by phosphorylation of β3 subunit.

*Aogi Kobayashi[1], Tomonori Furukawa[1], Saki Hatakeyama[1], Shuji Shimoyama[1], Shinya Ueno[1] ([1]*Department of Neurophysiology, Hirosaki University Graduate School of Medicine*)

### [2P-018]

### Age-related downregulation of the LINC complex and nuclear structural abnormalities in neurons

*Mina Amemiya[1,2], Koichi Hasegawa[2], Noriyki Hama[2], Ken-ichiro Kuwako[2] ([1]*Faculty of Medicine, Shimane University*, [2]*Department of Neural and Muscular Physiology, School of Medicine, Shimane University*)

Aging is the most significant factor causing decline in brain functions such as memory and cognition. However, the mechanisms of age-related physiological brain aging remain largely unknown. Previous studies in non-neuronal cells have suggested that nuclear abnormalities may cause senescence of cellular function. In neurons, although it has been suggested that abnormalities in nuclear structure may be associated with reduced level of neuronal activity, the direct involvement of nuclear abnormality in neuronal aging has yet to be elusive. The LINC complex, a nuclear envelope protein complex, is composed of KASH domain proteins such as Nesprin-1 and Nesprin-2 on the outer nuclear membrane and Sun proteins such as Sun1 and Sun2 on the inner nuclear membrane. Sun proteins bind to the nuclear lamin in the nucleus, while Nesprins interact with the cytoskeleton, including actin and microtubules, in the cytoplasm, thus the LINC complex plays an important role in structurally linking the nucleus and cytoplasm. In this study, we first examined the expression changes of the LINC complex molecules (Sun1, Sun2, Nesprin-1 and Nesprin-2) in mouse neurons during brain aging by immunohistochemical method. We found that the expression of the four LINC complex molecules was dramatically decreased in aged neurons compared to young neuronsin most cerebral cortical regions we examined, including prefrontal, somatosensory and motor area. We also found that the roundness of the nucleus was reduced and the number of the infolded nuclei was significantly increased in neurons of the aged prefrontal cortex. To elucidate the causal relationship between the age-related decreased expression (i.e. dysfunction) of the LINC complex and structural abnormalities in the nucleus, we next inhibited LINC complex function in young cortical neurons in vivo. Forced expression of a dominant-negative mutant of the LINC complex induced the structural abnormalities of the nucleus in young cortical neurons, similar to those of aged neurons. These results suggest that the age-related decline in LINC complex expression in neurons may cause structural abnormalities in the nucleus that may lead to a loss of neuronal function.

### [2P-019]

### Analysis for molecular profiling in the LINC complex-mediated AIS regulation

*Keiichiro Nakamura[1,2], Koichi Hasegawa[2], Noriyuki Hama[2], Ken-ichiro Kuwako[2] ([1]*Faculty of Medicine, Shimane University*, [2]*Department of Neural and Muscular Physiology, School of Medicine, Shimane University*)

The axon initial segment (AIS), a highly specialized structure located in the proximal axon, is essential to the regulation of neural activity and the formation of molecular barriers between the cell body and the axon. The AIS specifically accumulates the scaffold protein Ankyrin G, a master molecule for AIS formation, and the cytoskeletal protein βIV spectrin, and also contains specific voltage-gated sodium channels (Nav)/potassium channels (Kv) and the microtubule-associated proteins such as Trim46 and Camsap2. The AIS plays a key role in the generation and regulation of action potentials through Nav and Kv. Previous studies have shown that the AIS has structural plasticity that allows it to fluctuate its own length and position in response to stimuli, thereby altering the molecular repertoire of voltage-gated channels to regulate neural activity. The linker of nucleoskeleton and cytoskeleton (LINC) complex is a nuclear envelope protein complex composed of Nesprin and Sun proteins. Nesprins on the outer nuclear membrane interact with the cytoskeleton including microtubules and actin, and thus the LINC complex serves an important function as the center of the cytoskeletal network. We recently demonstrate that functional inhibition of the LINC complex in cortical neurons shortens their AIS length and completely abolishes their structural plasticity induced by depolarizing stimuli. We also found that dysfunction of the LINC complex significantly reduced the neuronal activity in cortical neurons. These facts strongly suggest the existence of a new regulatory system of neural activity via AIS control. However, the molecular mechanism of the LINC complex-mediated AIS regulation remains completely unknown. In this study, we examined the effects of inhibition of the LINC complex on the localization and expression levels of the AIS molecules in primary cortical neurons. Expression of a dominant-negative mutant of the LINC complex significantly reduced the total signal intensities of Ankyrin G and βIV spectrin in the AIS, which may reflect a shortening of AIS length. Interestingly, the molecular density of phosphorylated myosin light chain, an essential component of actin motor myosin II that was recently reported to play an important role in the regulation of AIS plasticity, was largely reduced by inhibition of the LINC complex. We are currently investigating the profiling of other AIS molecules, which will shed light on the mechanism of the LINC complex-mediated AIS regulation.

### Poster

### [2P]
### Neurophysiology, Neuronal cell biology
### Higher brain function

March 29, 13:00 - 14:20, Poster Room

### [2P-020]

### Change in the diversity of ripple firings during notation and recall in contextual learning task

*Shohei Fukuda[1], Junko Ishikawa[1], Dai Mitsushima[1] ([1]*Yamaguchi University*)

### [2P-021]

### Measurement of brain activity during dual warning stimulus task

*Takatoshi Satake[1,2], Kazumi Kasahara[2], Daisuke Yoshimaru[4,2,3], Ai Taki[2,3], Tomokazu Tsurugizawa[2,3] ([1]*Graduate School of Comprehensive Human Science, University of Tsukuba, Japan,* [2]*National Institute of Advanced Industrial Science and Technology(AIST), Japan,* [3]*Faculty of Engineering, Information and Systems, Univ. of Tsukuba, Japan,* [4]*Division of Regenerative Medicine, The Jikei University School of Medicine, Japan*)

Several functional MRI (fMRI) studies have investigated the activated brain regions associated with reaction tasks. The warning stimulus task followed by a target stimulus evokes the activation in the inferior frontal cortex and the anterior supplementary motor area of the right brain are involved. However, the impact of dual warnings on brain activity unclear. In this study, we aimed to investigate the relationship between response performance and brain function when two warning signal tasks by visual target stimuli. Twenty-two healthy young adults (17 males and 5 females, ages 22 ± 4.03) participated in the fMRI experiment using a 3.0 T clinical MRI system. To investigate the effect of warning signals, we conducted the following button-pressing tasks with healthy volunteers in MRI bore. The subjects predicted the "go" stimulus as a target that appeared with constant intervals following the two sequential warning stimuli, "ready" and "set". Two task conditions were created, and each was performed three times alternately. Condition 1 was set up with unified intervals (1s) between "set" and "go," and condition 2 was set up with variable intervals (0.6 s,1 s, or 1.3 s) between "set" and "go" of three different types. In addition, to investigate the effect of feedback, the subjects received four types of feedback immediately after the button press; "false" for a response less than 200 ms following the "go" stimulus, "excellent" for a response of 200 to 300 ms, "good" for a response within 300 to 400 ms, and "bad" for more than 400 ms. The response performance was classified as the discrete score; “excellent” is 3, “good” is 2, “bad” is 1, and "false" is 0. The response time and performance score in the constant interval task was significantly better than that in the variable interval task. Furthermore, several brain regions including bilateral visual cortex and left motor cortex were significantly activated both in unified intervals and variable intervals. This activation by unified interval task was significantly larger than the variable interval task ($p<0.05$, FDR-corrected at cluster level). COI: I have no COI.

### [2P-022]

### Neuronal basis underlying multi-tasking: neurons in the posterior medial prefrontal cortex of primates changes their functions across different tasks.

*Yoshiya Matsuzaka[1], Muhammad Ali Haider Awan[2], Hajime Mushiake[3] ([1]*Tohoku Med Pharm Univ,* [2]*Inst Translat Neurosci, Sheffield Univ,* [3]*Tohoku Univ*)

Higher mammals are able to simultaneously learn and perform a wide array of complex behaviors, which raises questions about how the neural representations of multiple tasks coexist within the same neural network. Do neurons play invariant roles across different tasks? Alternatively, do the same neurons play different roles in different tasks? To address these questions, we examined neuronal activity in the posterior medial prefrontal cortex of primates while they were performing two versions of arm-reaching tasks that required the selection of multiple behavioral tactics (i.e., internal protocol of action selection), a critical requirement for the activation of this area. During the performance of these tasks, neurons in the pmPFC exhibited selective activity for the tactics, visuospatial information, action, or their combination. Surprisingly, in 82% of the tactics-selective neurons, the selective activity appeared in a particular task but not in both. Such task-specific neuronal representation appeared in 72% of the action-selective neurons. In addition, 95% of the neurons representing visuospatial information showed such activity exclusively in one task but not in both. Our findings indicate that the same neurons can play different roles across different tasks even though the tasks require common information, supporting the latter hypothesis.

### [2P-023]

### Simultaneous 24-hour Wireless EEG and EMG Measurements and Characteristics of Sleep EEG in Marmosets

*Masanori Sawamura[1], Chen Chih-Yang[3], Kaoru Isa[2], Masashi Nakamura[2], Tadashi Isa[2,3], Ryosuke Takahashi[1], Hirotaka Onoe[3] ([1]*Department of Neurology, Graduate school of Medicine, Kyoto University,* [2]*Department of Physiology and Neurobiology, Graduate School of Medicine, Kyoto University,* [3]*Human Brain Research Center, Kyoto University Graduate School of Medicine*)

**Objective:** Many sleep and electroencephalography (EEG) studies have been conducted using rodents. However, since the sleep pattern of rodents is nocturnal and polyphasic, their sleep patterns are very different from those of humans. Marmosets, one of small non-human primates, have diurnal and monophasic sleep patterns more similar to that of humans than that of rodents. In this study, we established a method for measuring EEG, electro-oculography (EOG), and electromyography (EMG) in marmosets continuously for 24 hours by using a small wireless EEG system that can be implanted on the head. Furthermore, we evaluate rapid eye movement (REM) sleep behavior disorder (RBD), a prodromal symptom of Lewy body disease (LBD), by measuring EEG, EOG, and EMG in a marmoset inoculated with α-synuclein fibrils into the olfactory bulb.**Methods:** Electrodes were implanted on the top of the skull for EEG and EOG measurements on three adult marmosets. EMG electrodes were inserted into the trapezius muscle and they were connected to a small plug, which was fixed with dental resin to the skull along with a protective chamber. EEG, EOG, and EMG data were recorded continuously for 24 hours using a wireless EEG system (emka technologies). EEG frequency was analyzed by Fast Fourier Transform (FFT analysis) to determine the power in the δ-waves (0.5–4 Hz), θ-waves (4–8 Hz), α-waves (8–13 Hz) and β-waves (13–30 Hz) was calculated. The sleep stage was automatically determined based on the power spectrum results and confirmed visually. Moreover, α-synuclein aggregates were administered to the stomach and duodenum of the same marmosets, and REM without atonia (RWA) was examined.**Results:** The EEG, EOG, and EMG successfully identified the REM and non-REM (NREM) sleep stages, and a clear diurnal sleep-wake rhythm was observed in the marmosets. During the night, a periodic increase in δ-wave power was observed, and a decrease in EOG and EMG power was observed, which was identified as slow wave sleep (SWS). We also found REM period, in which α- and β- wave power increased. In visual analysis of the EEG, the high amplitude waveform in the θ–δ waveband was observed in the SWS period. The low amplitude waveforms in the α- and β-wave bands were observed in the REM period, as previously reported, but many spindle-like waveforms with high amplitude around 10 Hz were also observed. We also confirmed that the frequency of RWA increased after inoculation of α-synuclein fibrils.**Conclusion:** We established a technique for wireless 24-hour continuous measurement of EEG, EOG and EMG in marmosets. We succeeded in clearly distinguishing the REM and NREM sleep stages, and detected the appearance of symptoms similar to RBD.

### [2P-025]

### Quantitative assessment of attention allocation in Virtual Reality using somatic P300 responses

*Shingo Murakami Murakami[1], Kageyama Kaito[1], Maki Rintaro[1], Nakatani Yasushi[2], Ono Yumie[3] ([1]*Department of Electrical, Electronics, and Communication Engineering, Faculty of Science and Engineering, Chuo University,* [2]*Faculty of Economics, Chuo University, Tokyo, Japan,* [3]*Department of Electronics and Bioinformatics, School of Science and Technology, Meiji University*)

Virtual reality (VR) is known for its deeply immersive experience. To improve this immersion, it is important to use objective and quantitative evaluation methods within VR technology. Immersion, interpreted as the shift of attention from the real world to the virtual space, plays a pivotal role in the immersive experience. In our previous research (Ogawa *et al.*, ABE, 2022), an EEG-based method was introduced, utilizing auditory P300 responses to measure attention allocation while viewing 2D or 3D videos. This approach quantitatively assessed the allocation of attention from reality to the virtual world by measuring P300 amplitudes triggered by sound stimuli. However, this method was restricted to evaluating immersion in VR content accompanied by audio, due to its reliance on sound as a probe stimulus. Hence, we adopted a somatic probe stimulus method to objectively quantify attention allocation in viewing VR videos with audio. We innovated a somatic P300 system that incorporates vibration stimuli applied to a user's fingers while viewing VR video. A somatic oddball task was performed by ten young adult participants while experiencing VR content. The amplitude of the event-related P300 wave during this task served as a measure of attention directed toward the VR content. Piezoelectric device vibrations were delivered randomly as probe stimuli to the thumb (standard stimulus, 70%), middle finger (target stimulus, 15%), and little finger (deviant stimulus, 15%). EEG signals were recorded at Cz and Pz, filtered from 0.5 to 30 Hz, with a sampling frequency of 1000 Hz. To reduce electromyogram signal interference, mental counting replaced the switch-pressing method used in the prior study. Analyzing the event-related potential waveforms obtained from ten subjects revealed distinct P300 responses for both the target and deviant stimuli. The peak amplitudes of the P300 wave for these stimuli during VR video viewing were notably smaller than those during periods of non-VR video viewing. This indicates that the proposed method allows for objective and quantitative assessment of immersion intensity, even within VR environments accompanied by audio.

### [2P-024]

### Exploring the impact of hypersonic herp music on alpha activity: A study of music tone, high-frequency component and psychological factors.

*Misako Matsui[1], Yuri Masaoka[1], Nobuyoshi Koiwa[2], Motoyasu Honma[1], Shota Kosuge[1], Miku Kosuge[1], Daiki Shoji[1], Shunsuke Sakakura[1], Katsunori Inagaki[3], Masahiko Izumizaki[1] ([1]*Department of Physiological Regulation, Faculty of Medicine, Showa University,* [2]*Department of Psychosomatic Health Science, Faculty of Human Sciences, Human Comprehensive Sciences University,* [3]*Department of Orthopedic Surgery, Faculty of Medicine, Showa University*)

In this study, we conducted electroencephalogram (EEG) recordings while exposing participants to two distinct types of herp music: one with high-frequency components (HFCs) above 20 kHz, and the other without HFCs, falling within the audible range (below 20 kHz). Our primary goal was to investigate the connection between the hypersonic effect and the alpha frequency components (8-12Hz) in spontaneous EEG. The participants in our study, with an average age of 33.8 ± 8.6 years, experienced four music conditions, each lasting 300 seconds. These conditions included minor key herp music with HFCs, minor key herp music without HFCs, major key herp music with HFCs, and major key herp music without HFCs. Our findings indicated a significant decrease in alpha EEG activity in response to major key herp music with HFCs, particularly in the frontal regions. The disappearance of alpha component, synchronization during exposure to major key herp music with HFCs suggested that the subjects were in an awake and active state. Furthermore, this desynchronization of alpha power was associated with the participants' levels of depression and trait anxiety. Subjects with higher levels of depression and trait anxiety exhibited a tendency toward alpha component desynchronization. These results imply that the psychological benefits of herp music are influenced by factors such as the musical tone, the presence or absence of hypersonic elements, and the individual's levels of depression and trait anxiety.

### Poster

[2P]

### Neurophysiology, Neuronal cell biology

**Motor function**

March 29, 13:00 - 14:20, Poster Room

### [2P-026]

### Additive effect of Ninjin'yoeito on forced limb after intracerebral hemorrhage in rats is mediated by prevention of fast-twitch muscle atrophy

*Naoki Tajiri[1], Shinya Ueno[1], Dewi Mustika[1], Keita Mizuno[2], Hideki Hida[1] ([1]*Department of Neurophysiology & Brain Science, Graduate School of Medical Sciences & Medical School, Nagoya City University,* [2]*Tsumura Kampo Research Laboratories, Tsumura and Co*)

The importance of Kampo medicine is gradually recognized, especially Ninjin'yoeito (NYT) has effects on the brain and the muscle. However, the mechanism how Kampo medicine such as NYT effects on the brain remain unknown. In this study, we investigate whether the combination of FLU and NYT can improve better the recovery after intracerebral hemorrhage (ICH). ICH model was made by injecting type IV collagenase (15 units/ml, 1.4μl) near the internal capsule of male rats. FLU was initiated from day 1 after the ICH surgery and continued for 7 days, with the rats consuming chow containing 1% NYT until day 56. Five groups were prepared: sham-operated group, ICH-only group, ICH+FLU group, ICH+NYT group, and ICH+FLU+NYT group. Gross motor dysfunction was assessed using a motor deficits score (MDS) conducted until day 56. We conducted additional behavioral evaluations, including horizontal ladder test, gait analysis, and open field test on day 28. We revealed that the FLU + NYT group exhibited significantly better functional recovery in MDS compared to the ICH-only group. In open field test, the FLU+NYT group showed the longest total walking distance and a significantly increased maximum locomotion speed compare to ICH-only group. Retrograde labeling of corticospinal neurons with FluoroGold (FG) revealed that no significant increase of FG-positive cells was shown in FLU+NYT group. Anterograde labeling with biotinylated dextran amine (BDA) revealed that BDA-positive bouton-like varicosities in the red nucleus was increased by FLU, although similar number of the positive cells in FLU+NYT groups. To investigate whether the FLU + NYT group affected muscle atrophy and changes in skeletal muscles types, gastrocnemius and soleus were stained with antibodies for MHC I (slow twitch marker), and MHC IIb (fast twitch marker). Although the ICH-only group led to a significant decrease in both muscle type on the ipsilateral side, the FLU+NYT group showed a tendency to preserve fast-twitch muscle in the gastrocnemius. Data suggest that the combination of FLU + NYT improves the function recovery after ICH, indicating that the additive effect of NYT on the recovery was probably due to atrophy prevention on fast-twitch muscle.

### [2P-027]

### Influence of neocortical states on cerebrocerebellar communication

*Misa Shimuta[1], Daichi Konno[2,3], Takuya Sasaki[2,4], Taro Ishikawa[1] ([1]*Dept. Pharmacology, Jikei Univ. Sch. of Med.,* [2]*Lab. Chemical Pharmacology, Grad. Sch. of Pharmaceutical Sciences, The Univ. of Tokyo,* [3]*Lab. Geriatric Medicine, Grad. Sch. of Medicine, The Univ. of Tokyo,* [4]*Dept. Pharmacology, Grad. Sch. of Pharmaceutical Sciences, Tohoku University*)

The relationship between sleep and cerebellar activity and its coordination with other brain areas remains understudied. This is particularly important given the essential role of sleep in memory formation and consolidation. Our previous studies have shown a substantial influence of the neocortex on cerebellar activity in conscious and anesthetized mice. In this study, we aimed to understand how sleep modulates the interaction between the neocortex and the cerebellum during natural sleep cycles. We performed simultaneous local field potential (LFP) recordings from somatosensory (S1) and retrosplenial (RSp) cortex, as well as from crus I and II of the cerebellar cortex, in addition to EMG recordings from the neck muscles of freely moving mice (n=4). Over 3-4 hours, these mice explored a sound-attenuated chamber and spontaneously fell asleep. After the session, anesthetics were administered intraperitoneally to collect data on the anesthetized state. Off-line analysis using EMG and RSp LFP identified five brain states: REM and non-REM sleep, active and quiet wakefulness, and anesthetized state. LFP waves, when Fourier transformed, showed variations in magnitude across frequency bands. In retrosplenial cortex, θ and δ magnitudes peaked during REM and non-REM sleep, respectively. Similar patterns were observed in cerebellar crus I and II, whereas S1 showed no clear magnitude shifts across states. To assess neocortical-cerebellar correlations, we focused on S1-crus II connectivity, which is known to be highly interconnected. Notably, correlations during REM sleep were reduced across all frequency bands compared to non-REM, quiet wakefulness, and anesthetized states, with higher frequency bands showing more pronounced differences. Our results suggest that non-REM sleep may be critical for cerebrocerebellar communication during sleep. (COI: NO)

### [2P-028]

### Reward history enhances action-related activity of nigral dopaminergic striatal output pathways

*ALAIN RIOS[1], Satoshi Nonomura[2], Shigeki Kato[3], Junichi Yoshida[4], Natsuki Matsushita[5], Atsushi Nambu[6], Masahiko Takada[2], Riichiro Hira[1], Kazuto Kobayashi[3], Yutaka Sakai[7], Minoru Kimura[7], Yoshikazu isomura[1] ([1]*Tokyo Medical and Dental University,* [2]*Center for the Evolutionary Origins of Human Behavior, Kyoto University.,* [3]*Department of Molecular Genetics, Institute of Biomedical Science, Fukushima Medical University.,* [4]*Dominick P. Purpura Department of Neuroscience, Albert Einstein College of Medicine.,* [5]*Division of Laboratory Animal Research, Aichi Medical University.,* [6]*Division of System Neurophysiology, National Institute of Physiological Sciences and Department of Physiological Sciences, SOKENDAI.,* [7]*Brain Science Institute, Tamagawa University.*)

The nigrostriatal system's neurons are pivotal in the action selection. Yet, how this system combines recent outcomeswith action and outcome information to achieve following actions remains elusive. We delved into how the neuronal activities in the substantia nigra pars compacta (SNc) and dorsal striatum correlate with reward anticipation stemming from past outcomes. This was done using rats in a reward-driven choice activity. The activity tied to movement in both direct and indirect striatal projection neurons (dSPNs and iSPNs) was enhanced by anticipated rewards. This mirrored the activity pattern in SNc dopaminergic neurons across both medial and lateral nigrostriatal pathways. Taking into account the traditional basal ganglia model, where dopamine stimulates dSPNs and restrains iSPNs through distinct dopamine receptors, it appears that the anticipation of rewards can override dopamine's effects. Conversely, the activity related to outcomes was influenced by reward expectation, aligning with the established model and the principles of reinforcement learning.

### [2P-029]

### A novel rotating ladder running task apparatus for assessment of motor paralysis and neural activity after motor cortex infarction of mice

*Hironobu Osaki[1], Daisuke Ishii[2], Yoshito Masamizu[1] ([1]*Doshisha University,* [2]*Ibaraki Prefectural University of Health Sciences*)

Infarction of the motor cortex induces plastic changes in the remaining brain regions, such as the contralesional motor cortex. These changes are believed to be related to neuronal compensation mechanisms for functional recovery. To explore neuronal compensation mechanisms, monitoring changes in neural activity of the remaining brain regions during behavioral tasks is quite helpful. The ladder rung walking test evaluates the skilled walking ability of rodents. The test involves placing the animal on a horizontal ladder with irregularly spaced rungs and counting the number of missteps as it crosses the ladder. Therefore, it is suitable for assessing sensory-motor deficits and functional recovery after motor cortex infarction. However, the limited length of the ladder makes it difficult to monitor neural activity to study neural compensation mechanisms. To overcome this difficulty, we developed a rotary ladder running task apparatus for head-restrained mice. This allowed us to simultaneously assess and manipulate neural activity and motor function over an extended period. DeepLabCut (Mathis et al., 2018), a deep learning-based analysis, combined with three cameras that monitor animal behavior from different angles, was used for an unbiased and automated analysis of motor paralysis after photothrombotic infarction using Rose Bengal. Three-dimensional joint position reconstruction successfully monitored symptoms of motor paralysis of the forelimb, such as increased elbow joint angle and increased missteps of the contralateral forelimb of the lesion. Furthermore, by combining it with a custom-made Ca2+ imaging system of the contralesional motor cortex, we successfully monitored changes in neural activity associated with skilled walking after motor cortex infarction. This system is useful for elucidating the neural mechanisms of recovery of motor function and for developing an effective therapeutic strategy of rehabilitation for motor deficits after motor cortex infarction. (COI: NO)

### [2P-030]

### Diagonal-sequence diagonal-couplet quadrupedal gait in Japanese macaque enables flexible temporal inter-limb coordination.

*Kei Mochizuki[1], Kazunori Morita[1], Takashi Suzuki[1], Masahiko Inase[2], Katsumi Nakajima[1] ([1]*Iwate Medical University,* [2]*Kindai University*)

Quadrupedal animals' walking can be categorized into many types of gait patterns. Most animals such as horses and cats walk with a footfall pattern called lateral sequence, in which forelimbs touch down after ipsilateral hindlimbs. On the other hand, non-human primates adopt a gait called diagonal sequence, in which forelimbs touch down just after the contralateral hindlimbs. Each pair of contralateral fore-hindlimb is called a diagonal couplet, since it works synchronously in phase. While this diagonal-sequence diagonal-couplet (DS DC) gait is unique to non-human primates' locomotion, its functional significance has long been unclear.

In this study, we addressed this issue by examining quadrupedal gait in Japanese monkey walking on a treadmill at various speed. We used a wide speed range from ~2.0 km/h to ~10.0 km/h. The monkey was rewarded with a piece of vegetable or fruit for every 10–15 steps. For each speed condition, the belt speed was kept constant and the monkey walked on the treadmill for several minutes.

We found that the monkey's body axis was almost never parallel to the traveling direction. One diagonal fore-hindlimb couplet touched down centrally, approximately straight to the traveling direction ("straight couplet"). The other couplet touched down leftward and rightward aside from it, protruding laterally ("oblique couplet"). This locational difference between two diagonal couplets was maintained throughout all speed conditions. Furthermore, we found a substantial difference between these couplets when the belt speed became fast. During fast walking, limbs' stance phases of the oblique couplet became more overlapped, synchronizing their touch-downs and lift-offs. On the other hand, stance phases of the limbs of the straight couplet started to desynchronize during fast walking, shortening their double-support period. This means that limbs of the straight couplet disbanded their temporal relationship to work in separate timings, presumably to produce more propulsion and longer stride. Such division of labor between limb couplets may not be easily achieved in essentially symmetrical lateral-sequence gait in other animals. These findings suggest that the significance of DS DC gait in primates may better be understood in terms of flexible temporal inter-limb coordination, enabling to walk at a wide range of speed with the same gait pattern.

### [2P-031]

### Trunk movements during monkey's locomotion based on adaptive utilization of gravity

*Takashi Suzuki[1], Kei Mochizuki[1], Kazunori Morita[1], Yoshiro Suzuki[1], Masahiko Inase[2], Katsumi Nakajima[1] ([1]*Iwate medical university,* [2]*Kindai university*)

Japanese monkeys can walk on a treadmill and volitionally transform the trunk posture from horizontal for quadrupedal gait to vertical for bipedal gait and vice versa. The transformation of the gait is achieved with proficient coordination between trunk movements and changes of limb's stepping pattern. To investigate neural mechanisms related to the control of trunk movement, we first analyzed kinematics and EMG activity of the trunk and limbs during treadmill locomotion. During quadrupedal gait, the trunk was laid over the four stepping limbs. We found that, for transformation to bipedal gait, the trunk started to be righted up following the touchdown of either foot. The trunk was, then, set in near vertical on the two hindlimbs within a few steps. In parallel, the forelimbs were dissociated from cyclic movement of the hindlimbs and released from weight bearing. There was a strong laterality of which forelimb was first dissociated. Compared to steady-state quadrupedal gait, step cycle frequency of the hindlimb became higher to rostrally shift support base which now came under the upright trunk. The activity of antigravity muscles in the trunk and hindlimbs gradually increased during righting-up movement.

For transformation from bipedal to quadrupedal gait, we found that the upright trunk also started to be laid down following the touchdown of either foot. The trunk was, then, put into a horizontal position over the four limbs within a few hindlimb steps. Compared to steady-state bipedal gait, step cycle frequency of the hindlimb became lower during the laying-down movement. The position of the hindlimbs relative to the trunk shifted caudally and support base was formed again by the fore and hind limbs right under the trunk. Contrary to the righting-up movement, there was no laterality bias of which forelimb first touched down for quadrupedal gait. The activity of antigravity muscles in the trunk and hindlimbs gradually decreased during laying-down movement. Our results suggest that, for volitional trunk movements during gait, the monkey CNS appropriately modifies stepping limb movements to generate reaction for controlling trunk movements and precisely adjusts the activity of antigravity muscles to cooperate with gravity. This study will bring new insights into CNS mechanisms for controlling whole body movements based on adaption to gravitational field.

### [2P-032]

### Behavioral and neurological effects of Vrk1 deficiency in zebrafish

*Magdeline Elizabeth Carrasco Apolinario[1], Ryohei Umeda[1], Hitoshi Teranishi[1], Mengting Shan[1], Phurpa Phurpa[1], Wulan Sebastian[1], Shaohong Lai[1], Nobuyuki Shimizu[1], Hiroshi Shiraishi[1], Kenshiro Shikano[1], Takatoshi Hikida[2], Toshikatsu Hanada[1], Keisuke Ohta[3], Reiko Hanada[1] ([1]*Oita University,* [2]*Osaka University,* [3]*Kurume University*)

VRK1 is a serine/threonine kinase whose mutations have been reported in several neurodegenerative diseases, including spinal muscular atrophy associated with microcephaly, impaired cognitive function, and motor dysfunction in humans. Functions of VRK1 are reported in cell cycle progression, nuclear assembly and disassembly, transcription regulation, and chromatin condensation. However, precise pathophysiological mechanisms or VRK1 in neurodegenerative disorders have not been fully investigated.

To address this issue we established vrk1-deficient ($vrk1^{-/-}$) zebrafish and evaluated the neurological phenotype resulting in mild microcephaly and impaired motor function with a low brain dopamine content. Vrk1 deficient zebrafish also exhibited a decrease in cell growth, nuclear envelope formation defects, and heterochromatin formation in the brain. This is the first report that demonstrates the significant role of VRK1 in microcephaly and motor dysfunction in zebrafish. These findings help us understand the pathophysiological mechanisms that cause VRK1-related neurodegenerative diseases associated with microcephaly.

### Poster

### [2P]
### Neurophysiology, Neuronal cell biology
### Sensory function, Sensory organ

March 29, 13:00 - 14:20, Poster Room

### [2P-033]

### The effect of acetylcholine on starburst amacrine cells in the mouse retina

*Mie Gangi[1], Takuma Maruyama[2], Toshiyuki Ishii[1], Makoto Kaneda[1] ([1]*Nippon Medical School,* [2]*Tokyo Women's Medical University*)

In the retina, ACh release is exclusive to starburst amacrine cells (SACs). SACs also release GABA, and the GABAergic signals originating from SACs play a key role in the formation of direction selectivity in the retina. However, the function of ACh remains controversial. SACs consist of two subgroups: ON and OFF SACs, with similar morphological characteristics, except for their localization in the retina. The mirror symmetry of their morphologies strongly suggests similarities in their physiological properties. However, recent studies demonstrated that ON and OFF SACs were different in gene expression patterns and receptors, implying the different functions of ON and OFF SACs.

Here, we compared the cholinergic signaling pathways between ON and OFF SACs in the mouse retina. ACh induced GABAergic feedback to SACs in both ON and OFF SACs. However, ACh receptors involved in this feedback in adult were different in ON and OFF SACs, which were originally same in the early developmental stage. This feedback remained even in the presence of TTX in both SACs, implying that the ACh-induced GABAergic feedback may originate from non-spiking amacrine cells. When mGluR2 receptor agonist LY354740 was used to inhibit the transmitter release from SACs, spontaneous GABAergic inputs decreased, but ACh induced GABAergic feedback remained in both SACs. These findings suggest that the release of ACh from ON and OFF SACs might be regulated by amacrine cells other than SACs. Finally, to investigate the timing of ACh release from SACs, we recorded light responses from ON SACs and examined the effects of ACh blockers on these responses. The application of ACh blockers during full-field light stimuli did not produce discernible effects, suggesting that more complex light stimuli or manipulation of adaptation levels may be necessary for a comprehensive understanding.

### [2P-034]

### Behavioral discrimination of the sour taste solutions in rats

*Shinpei Takahashi[1], Shusuke Iwata[1], Toshiaki Yasuo[1], Takeshi Suwabe[1], Noritaka Sako[1] ([1]*Department of Oral Physiology, Asahi University School of Dentistry*)

[Introduction]Our lab's colleagues have revealed that rats can recognize the components in binary taste mixtures containing different taste qualities (Katagawa *et al*., 2016; Yamamura *et al.*, 2020). In the last annual meeting, we also demonstrated that the rats could recognize the components in binary sweet taste mixtures containing glucose (Glc) and Fructose (Fru), and that the rats aversive-conditioned to Glc could discriminate it from Fru, even if they had same quality of sweet taste. In the present study, we investigated whether rats could discriminate between different sour taste substances. [Methods]Male Wistar/ST rats (7 weeks of the age) were divided into the conditioned (n=7) and the control groups (n=6). The first 5 days were a training period, and the rats deprived water were allowed presentation of distilled water (DW) for 10 min. On the 6th day, the rats were allowed presentation of 10mM HCl for 10 min just before the injection of either 0.15M lithium chloride (the conditioned groups) or physiological saline (the control groups). On the following 10 test days after a recovery day, the number of licks for test solutions were measured for 10 sec. As sour test stimuli, 10mM HCl, 10mM acetic acid, 10mM ascorbic acid, 10mM citric acid and their binary mixtures were used. As non-sour stimuli, 0.5M sucrose and 0.1mM quinine-HCl were also used. [Results, discussion and summary] When the licking patterns were compared to the control group, the following results were obtained in the conditioned one. On the first test day, 33% of conditioned rats avoided all tested taste stimuli, and 67% of them did all tested sour taste stimuli. Thus, all conditioned rats could not discriminate HCl and other sour stimuli on this test day. When the test was continued, the rats avoided only HCl. These results may suggest rats have an ability of discrimination of sour tastes as well as sweet ones, and that this ability for sour is not as high as that for sweet.

### [2P-035]

### Visualization of neurons involved in aversive olfactory memory using TRAP mice

*Yoshihiro Murata[1], Maho Asano[1], Wakana Okami[1], Mutsuo Taniguchi[1], Masahiro Yamaguchi[1] ([1]*Department pf Physiology, Kochi Medical School*)

Olfactory memories strongly trigger the recall of emotional experiences. To elucidate the neural mechanisms, we tried to visualize the active neurons during olfactory memory recall using transgenic mice known as Targeted Recombination of Active Population (TRAP). In this study, aversive odor conditioning with electrical foot shock was conducted for the formation of olfactory memories with emotional experiences in the TRAP2 mice (Allen et al., 2017; males, $\geq$ 8 weeks old). The odor conditioning was performed according to the protocol of a previous report (Murata K., et al, 2015). Immunohistochemical analyses indicated that expressions of reporter protein tdTomato in response to the conditioned odor of eugenol were observed in the mouse brain such as the olfactory bulb, the olfactory cortex, and the amygdala. The results in the olfactory tubercle (OT), a part of the olfactory cortex, were consistent with the previous report that the aversive odor conditioning accompanies the activation of OT neurons in the lateral domain. The present study suggests the availability of the TRAP2 mice for visualizing the neural circuits involved in aversive olfactory memory.

### [2P-036]

### Pathogenic mechanism of dry eye-induced chronic ocular pain focusing on neuron-satellite glial cell communication in trigeminal ganglion

*Yuto Tei[1,2], Yoshinori Mikami[1], Taichiro Tomida[1], Daisuke Ohshima[1], Ryuji Hisamura[3], Katsuhide Yamasaki[3], Yuichi Hori[2], Satomi Adachi-Akahane[1] ([1]*Dept Physiol, Faculty Med, Toho Univ.*, [2]*Dept Ophthalmology, Faculty Med, Toho Univ.*, [3]*Ophtecs co.*)

The number of dry eye patients has been increasing rapidly in recent years. The typical symptoms include hypersensitivity and hyperalgesia, which significantly reduce the quality of life, yet treatment options are limited. We found that chronic intractable dry eye causes neuropathic pain. We have previously reported that ocular neuropathic pain involves the activation of neurons and glial cells at the level of the trigeminal nucleus, and continuous treatment with pregabalin is effective. However, the upstream mechanism in the trigeminal ganglion remains unclear. The purpose of this study was to clarify a pathogenic mechanism of dry eye-induced chronic ocular pain for developing an effective treatment of ocular neuropathic pain with minimal side effects. We tested the hypothesis that dry eye induces hypersensitivity and hyperalgesia through the activation of neurons and satellite glial cells in the trigeminal ganglion. We created and tested a VDT user dry eye model using female Sprague-Dawley rats. A dry eye was induced by placing the rat on a swing to keep the eyes open and applying air to dry them, which reproduced the real clinical situation of dry eye patients. The left eye was protected from dryness and used as a control. Phenotypic analysis showed that dry eye caused corneal epithelial damage, hypersensitivity, and hyperalgesia. To clarify the pathological mechanism, gene expression related to the development of neuropathic pain was analyzed by qRT-PCR. The results indicated that the neuronal activity and satellite glial cell markers are significantly increased in the trigeminal ganglion in the acute phase. In the chronic phase, VGCC$\alpha_2\delta$-1 subunit, CGRP, satellite glial cell, IL-1β, and TNFα gene expression were increased. The chronic phase results suggested that chronic activation of primary afferent neurons by dry eye-induced corneal damage may have activated satellite glia in the trigeminal ganglion and activated each other via CGRP and inflammatory cytokines, thereby increasing the input of pain information and causing hypersensitivity and hyperalgesia. Neural hyperactivity in the trigeminal ganglia and increased expression of VGCC$\alpha_2\delta$-1 subunit and CGRP may also contribute to sensitization of the trigeminal nucleus.
COI:properly declared

### [2P-037]

### Burst-induced long-term potentiation at synapses in the olfactory tubercle

*Sajib Podder[1], Yoshihiro Murata[1], Mutsuo Taniguchi[1], Masahiro Yamaguchi[1]
([1]*Department of Physiology, Kochi Medical School*)

Olfactory tubercle (OT), a part of the olfactory cortex, has a crucial role for odor associative learning. Appetitive and aversive odor conditionings accompany the activation of OT neurons in a domain-specific manner. This characteristic has led us to hypothesize that the mechanism includes plastic changes at synapses in the OT during the conditionings. To test the hypothesis, we examined whether long-term potentiation (LTP) occurs at the excitatory synapses in the OT by using field potential recordings. We prepared coronal acute brain slices (300 µm thickness) of mice and applied field recordings of excitatory postsynaptic potential (EPSP) at synapses in the olfactory tubercle for analyzing changes in the synaptic efficacy. Field EPSP was recorded when electrical stimulation (> 0.10 mA, 50 µs duration) was applied to one of the OT layers including the input fibers. Pharmacological analyses showed that the EPSPs depended on glutamatergic transmission through non-NMDA receptors. In the layers I and III of the OT, a set of 2 Hz burst stimulation elicited short-term potentiation of the field EPSPs that decayed back to their baseline values, whereas 3 sets with 1-min interval induced LTP that remained potentiated for 2.5 h. These results indicate that LTP can occur at the excitatory synapses in the OT, suggesting its contribution to the odor conditioning-dependent activation.

### [2P-038]

### Characterizing respiratory patterns during sniffing in mice.

*Tomohiro Noguchi[1], Hitoshi Sasajima[1], Sadaharu Miyazono[1], Mirai Takahashi[1], Hajime Sato[3], Hideaki Shiga[2], Kaoru Takakusaki[1] ([1]*Asahikawa Med. Univ.*, [2]*Kanazawa Med. Univ.*, [3]*Meikai Univ.*)

Respiration cycles, comprised of inhalation and exhalation phases, during exploration in mice are shortening. This rapid short breathing is called sniffing. Such shorter-cycle breathing may make shorten cycles of oscillatory receptor potentials in olfactory sensory neurons through synchronization with changes in odorant concentration in nasal cavity. Previously, our patch-clamp study demonstrated that shorter cycles of oscillatory depolarization increase information amount carried by rate codes of firing in olfactory sensory neurons. Therefore, we hypothesized that short-cycle breathing in sniffing enhances information transfer of olfactory sensory neurons than long-cycle breathing in a resting state. It is speculated that the sniffing respiration that accompanies exploratory behavior not only quickly takes in odorants from the outside environment into the nasal cavity, but also improves the accuracy of odor identification itself. In order to further verify this hypothesis, we measured the respiratory flow in mice using an unrestrained whole-body plethysmograph. This device uses a differential pressure transducer to measure respiratory flow derived from breathing of a freely moving mouse in a subject chamber. First, we compared the respiratory flow under anesthesia and after awakening. A BALB/c male mouse (8 weeks old or older) lightly anesthetized by sevoflurane inhalation was placed in the subject chamber, and the respiratory flow was measured. Mice (n = 6) awoke from anesthesia in several tens seconds and began actively exploring inside the chamber immediately afterward. The exploratory behavior was accompanied with sniffing-like breathing. The waveform of respiratory flow clearly differs between anesthetized and awakened states. Under anesthesia, a constant waveform continues in a regular manner, while after awakening, the amplitude increases and the period shortens, and the waveform of each breath changes. In power spectrum analysis of the respiratory flow, significant peaks were observed at 2 Hz and 5 Hz under anesthesia, but at around 10 Hz after awakening. We also attempted to extract parameters characterizing the respiratory waveform of sniffing induced by odor stimulation. Clarifying the respiratory patterns of sniffing provides a basis for elucidating neural mechanisms of an olfactomotor system controlling respiration.

### [2P-039]

### Effects of Sleep Deprivation on Nociceptive Behavior and Neuronal Responses Elicited in the Mouse Anterior Cingulate Cortex

*Kosuke Nakano[1,2], Keisuke Koga[1], Hidenori Koyama[1,2], Hidemasa Furue[1] ([1]*Dept Neurophysiol, Hyogo Medical University*, [2]*Dept Diabetes, Endocrinol Clin Immunol, Hyogo Medical University*)

Sleep deprivation can be both a cause and a consequence of pain. Although acute and chronic sleep deprivations are known to induce pain hypersensitivity, it is not fully understood how sleep deprivation alters nociceptive neuronal activities in the brain. In this study, we investigated effects of sleep deprivation on nociceptive behavior and neuronal activity in the anterior cingulate cortex (ACC), which is thought to be important for acute pain perception as well as the development of chronic pain. Mice were received a chronic sleep deprivation for 4 weeks. In sleep-deprived mice, nociceptive mechanical thresholds were remarkably decreased, and the mechanical hypersensitivity was lasted longer more than several weeks. *In vivo* multi-unit recordings were made from ACC neurons of anesthetized control and sleep-deprived mice, and nociceptive stimulation was applied to the skin of hind limb. ACC neurons exhibited spontaneous firings, and cutaneous nociceptive stimulation increased their firing frequency. In ACC neurons of control mice, nociceptive stimulation-induced firings were elicited during the period of stimulation. Interestingly, in ACC neurons of sleep-deprived mice, long-lasting after discharges were detected after cessation of nociceptive stimulation. Chemogenetic inhibition of ACC pyramidal neurons ameliorated mechanical hypersensitivity in sleep deprived mice, and mirogabalin, a chronic pain medication, also alleviated sleep deprivation-induced mechanical hypersensitivity. These results suggest that nociceptive responses elicited in ACC neurons are facilitated following sleep deprivation, and this enhanced ACC neuronal activity is important for the induction of sleep deprivation-induced chronic pain.

### Poster

### [2P]
**Molecular physiology, Cell physiology**
**Ion channels, Receptors**

March 29, 13:00 - 14:20, Poster Room

### [2P-040]

### The Study of The TRPM4 Function in Keratinocytes

*Kaori Otsuka Saito[1,2], Puguh Indrasetiawan[2,3], Muthi Ikawati[3], Ratna Annisa Utami[4], Zhihan Guo[2], Hiroko Kato[2], Manami Toriyama[2,5], Takeshi Hara[1,2], Makoto Tominaga[6,7,8], Ken J Ishii[9], Fumitaka Fujita[1,2] ([1]*Advanced Technology Institute, Mandom Corp.,* [2]*Graduate School of Pharmaceutical Sciences, Osaka University,* [3]*Faculty of Pharmacy, Universitas Gadjah Mada,* [4]*School of Pharmacy, Institut Teknologi Bandung,* [5]*Graduate School of Science and Technology, Nara Institute of Science and Technology,* [6]*Exploratory Research Center on Life and Living Systems, National Institutes of Natural Sciences,* [7]*National Institute for Physiological Sciences, National Institutes of Natural Sciences,* [8]*Department of Physiological Sciences, SOKENDAI,* [9]*The Institute of Medical Science, The University of Tokyo*)

The skin is a protective interface between the internal organs and environment and functions not only as a physical barrier but also as an immune organ. Keratinocytes (KC) are one of the major skin components where TRPM4, a member of the thermo-sensitive transient receptor potential (TRP) channel family, was recently reported to be expressed. Previously we have revealed that activation of TRPM4 surpressed the cytokine production in KC and identified the new TRPM4 agonist aluminum potassium sulfate. However, the role of TRPM4 in KC function remaines unclear. In this study, we showed that TRPM4 activation significantly reduced MMP9 production in normal human epidermal keratinocytes and in immortalized human epidermal KC (HaCaT cells), in gene and protein levels. TRPM4 activation using BTP2, a known TRPM4 agonist, and aluminum potassium sulfate significantly promoted KC proliferation in HaCaT cells. We further showed that this proliferation-increasing effect of TRPM4 agonist was disrupted by combined treatment with glibenclamide, a TRPM4 inhibitor. In addition, proliferation of HaCaT cells decreased in lower temperatures of 35°C and 33°C as previoulsy reported, and activation of TRPM4 recovered the proliferation. Taken together, these results suggest TRMP4 activation regulates KC proliferation and aluminum potassium sulfate will be an effective agent improving skin condition by regulating the proliferation and cytokine production through TRPM4.

### [2P-041]

### Elucidation of the mechanism of 5-HT-mediated activity of parasympathetic-like PC12 cells and the effect of Hange-Shashin-To

*Kaori Sato-Numata[1], Ryo Takayama[1], Keitatsu Ishikawa[1], Hirono Sugawara[1], Ayako Sakai[1], Tomohiro Numata[1] ([1]*Akita University*)

Irritable Bowel Syndrome (IBS) is a gastrointestinal disorder characterized by abnormal bowel movements, often triggered by increased fluid secretion into the intestines and microinflammation in the intestinal tract resulting from mental stress. Despite the well-established effectiveness of Hange-Shashin-To in alleviating diarrhea symptoms associated with IBS, its precise mechanism of action still needs to be discovered. Recent findings have proposed a link between 5-HT (serotonin) secretion in the intestinal plexus during stressful episodes and the onset of diarrhea. This study sought to explore the impact of 5-HT on parasympathetic-like PC12 cells and investigate the potential modulatory effects of Hange-Shashin-To.

To evaluate the effect of 5-HT on PC12 cells, we analyzed time-dependent changes in the cell area using cross-sectional area measurements. The results showed that undifferentiated cells remained unresponsive to 5-HT, whereas differentiated cells induced by nerve growth factor (NGF) exhibited a decrease in cell area. Further experiments revealed that secretory volume decreases (SVD) was abolished without extracellular $Ca^{2+}$. RT-PCR was used to assess the expression of receptors associated with the 5-HT pathway in undifferentiated and differentiated cells. The results revealed that the expression of 5-HT3A receptors was upregulated in differentiated cells compared to undifferentiated cells. In response to this result, a decrease in SVD was observed when 5-HT inhibitors were administered and evaluated based on SVD. These findings established that 5-HT causes an increase in intracellular $Ca^{2+}$ and stimulates secretion in differentiated PC12 cells by binding to the 5-HT3A receptor. Finally, the effect of Hange-Shashin-To on 5-HT-induced SVD was quantified by monitoring the changes in the cell cross-sectional area, establishing that SVD was significantly suppressed.

These results suggest that Hange-Shashin-To inhibits PC12 cell secretion by suppressing the 5-HT receptor-intracellular $Ca^{2+}$ upregulation pathway.

### [2P-042]

### Analysis of the diversity of prokaryotic calcium channels and its calcium-selectivity determinants

*Yuki Maeda[1], Tomoe Nakamura-Nishitani[1], Katsumasa Irie[1] ([1]*Wakayama Medical University*)

Calcium channels (Cavs) are indispensable for various physiological functions, including memory formation, muscle contraction and cardiac rhythm. The main role of Cavs is the selective permeation of calcium ions ($Ca^{2+}$), and sodium channels are known to be derived from Cavs. However, its calcium-selectivity determinants are not fully identified. Therefore, CavMr, the prokaryotic Cav we have identified, is a useful tools for understanding the evolutionary process of ion channels. Here, we identified several homologs of CavMr and compared their ion selectivity and amino acid sequences to elucidate the origin of ion selectivity.

We cloned three ion channels from prokaryotes living in tidal flats and salt lakes and analyzed the ion selectivity of these channels by electrophysiology. Currents were measured by the whole-cell patch clamp method using insect cells. One channel showed high permeability ratios of calcium per sodium ($P_{Ca}/P_{Na}$) over 100, indicating it is a $Ca^{2+}$ selective channel. One of the other two showed moderate $Ca^{2+}$ selectivity, and the other showed non-selective ion permeation.

Prokaryotic ion channels work as homo-tetramers, forming an ion pore in the centre of the tetramer. Four S5-S6 loops face each other in the ion pore. Seven amino acid residues critical for ion selectivity in these loops are called selectivity filters (SF). The sequence of the five N-terminal residues of SF in the three ion channels we cloned is TLEGW, the same as that of CavMr, but the two C-terminal residues differ from each other. In addition, CavMr has the characteristic amino acid sequence in the S5-S6 loop compaired to mammalian Cavs, but these ion channels lack this sequence. By mutational analysis, we evaluated their effects on $Ca^{2+}$ selectivity. When the C-terminal residue of SF is a negatively charged amino acid or small-side chain amino acid, the $Ca^{2+}$ selectivity increases. The C-terminal residue of SF is expected to face the extracellular side and form an ion entrance. Therefore, the entrance width and high negative charge are essential for $Ca^{2+}$ selective permeation.

### [2P-043]

### The role of PI(4,5)$P_2$ in mouse $GABA_AR$

*Risa Marie Mori-Kreiner[1], Takafumi Kawai[1], Yasushi Okamura[1] ([1]*Osaka University Graduate School of Medicine*)

This study investigates the functional significance of PI(4,5)$P_2$ binding in mouse GABA A receptors (m$GABA_A$Rs). PI(4,5)$P_2$, a crucial phospholipid component of the plasma membrane, is known to regulate the functions of various ion channels. Notably, recent Cryo-EM structures of human and mouse $GABA_A$Rs revealed PI(4,5)$P_2$ bound at the intracellular base of the α1 subunits.

In our previous electrophysiological experiments, α1β3γ2L m$GABA_A$Rs were heterologously expressed in *Xenopus* oocytes with voltage-sensing phosphatase (Ci-VSP) to deplete endogenous PI(4,5)$P_2$. Our results demonstrated that m$GABA_A$Rs exhibit a high affinity for PI(4,5)$P_2$, which can be weakened by mutating K311 to neutralize the positive charge ($\alpha1^{K311N}$). In addition, mutations of other candidate residues did not lead to increased sensitivity to VSP-mediated PI(4,5)$P_2$ depletion. However, the precise mechanism underlying the PI(4,5)$P_2$ regulation of m$GABA_A$R channel activity remains elusive. To further investigate the influence of PI(4,5)$P_2$ on m$GABA_A$R channel activity, we performed patch-clamp experiments to observe channel activity at the single-channel level. Furthermore, inside-out patch-clamp experiments were performed to examine the effects of PI(4,5)$P_2$ dissociation from the channel, comparing the "rundown" properties between $\alpha1^{wild\text{-}type}$ m$GABA_A$Rs and $\alpha1^{K311N}$ m$GABA_A$Rs.

Finally, to elucidate the physiological significance of PI(4,5)$P_2$ binding in $GABA_A$Rs, we also studied the mouse α4 subunit, which is highly expressed in extrasynaptic $GABA_A$Rs. Sequence alignment of the PI(4,5)$P_2$-binding regions reveals that the residue equivalent to the $\alpha1^{K311}$ in the α4 subunit is an asparagine (N310). To test whether m$GABA_A$Rs containing the α4 subunit show sensitivity to PI(4,5)$P_2$ depletion and whether this sensitivity can be reduced by mutation of N310K, we performed two-electrode voltage-clamp experiments to assess m$GABA_A$Rs co-expressed with Ci-VSP.

In summary, our study sheds new light on the regulatory role of PI(4,5)$P_2$ in $GABA_A$R channel activities, providing valuable insights into the molecular underpinnings of ion channel modulation with potential implications for future therapeutic strategies.

### [2P-044]

### Nav1.9 amino acid substitutions cause painful or painless symptoms due to differences in structural stability.

*Hiroko Okuda[1], Chieko Nagao[2], Bekker Gert-Jan[2], Atsuko Noguchi[4], Tsutomu Takahashi[4], Akio Koizumi[5], Shohab Youssefian[3], Toru Tezuka[3], Shinji Akioka[1,3] ([1]*Kyoto prefectural university medicine,* [2]*Osaka University,* [3]*Kyoto University,* [4]*Akita University,* [5]*Kyoto-Hokenkai*)

Familial episodic pain syndrome (FEPS) is an autosomal dominant inherited disorder characterized by paroxysmal pain episodes. Several gain-of-function variants of *SCN9A, SCN10A,* and *SCN11A*, which encode the voltage-gated sodium channels (VGSCs) Nav1.7, Nav1.8 and Nav1.9, respectively are associated with this disease. These VGSCs are expressed in dorsal root ganglia (DRG) that transmit peripheral pain signals to the central nervous system, and several of these VGSC variants are known to cause painful or painless disorders. Nav1.9 contributes to the generation of a persistent inward current at subthreshold voltages, and gain-of-function variants of Nav1.9 are surprisingly associated with two contrasting pain related phenotypes, familial episodic pain syndrome (FEPS) or congenital insensitivity to pain (CIP).In this study, we report a novel heterozygous variant of *SCN11A*, p.L811F, in a Japanese family with FEPS. A single nucleotide mutation at the same position gives rise to the p.L811P variant that is known to cause CIP. The affected Leu811 residue is located within the DII/S6 helix of Nav1.9 and is important for signal transduction from the voltage-sensing domain and for pore opening. To evaluate the effects of the L811F variant on the molecular stability and conformation of the DII/S6 region, which constitutes the cytosolic pore obstructing the hydrophobic gate, we conducted molecular dynamic simulations of Nav1.9. Moreover, to obtain functional insights into the potential impact of the L811F variant, we compared its functional effects with those of the wild-type (WT) and the CIP-related L811P proteins. Here we show that p.L811F increases the structural stability of Nav1.9, prevents its necessary conformational changes, and thereby alters the dynamics required for its functionality. In contrast, the CIP-related p.L811P variant destabilizes Nav1.9. We thus speculate that p.L811F may lead to current leakage, while p.L811P increases the current through Nav1.9. Furthermore, in terms of clinical relevance, we consider that current leakage resulting from L811F may lead to increased cellular excitability and the observed gain-of-function phenotype observed in FEPS.

### [2P-045]

### The regulation of the THIK-1 channel activity by the distal C-terminal region

*Michihiro Tateyama[1,2], Yoshihiro Kubo[1,2] ([1]*Div. Biophysics and Neurobiology, NIPS,* [2]*Dept of Advanced Sciences, SOKENDAI*)

A two-pore domain $K^+$ (K2P) channel, THIK-1, has been reported to play important roles in microglia and macrophage. The conductance of THIK-1 is known to be increased by a cleavage of the middle of C-terminal tail by the caspase 8. We also found that the THIK-1 mutants whose distal C-tail are truncated showed the large current density. Here, we investigated the effects of distal C-tail on the channel activity, by introducing an unnatural cross-linking amino acid, 4 amido-phenylalanine (AzF), into sixteen residues at the distal C-terminal tail (THIK-1-tail-AzF). The L398AzF mutant showed the maximal increase in the current amplitude upon the UV-exposure among the THIK-1-tail-AzF mutants, suggesting that Leu398 closely couple to the channel activity. As the inner helices of K2P serve as a regulatory domain for the channel conductance, we investigated the effect of Leu398A mutation on the conformation of the inner helices of THIK-1 channel. The incorporation of AzF into the residues in the lower part of inner helices made the mutants (THIK-1-helix-AzF) to respond the UV-exposure, while the additional L398A mutation changed the extent and/or direction of the responses in several THIK-1-helix-AzF mutants. These results show that Leu398 at the distal C-tail of THIK-1 is involved in the regulatory mechanism of the channel activity.

### [2P-046]

### A unique extracellular S4-S5 coupling in HCN channels regulates the voltage-dependent gating

*Jiaying Liu[1], Go Kasuya[1], Koichi Nakajo[1] ([1]*Div. of Integrative Physiol., Dept. of Physiol, Jichi Med Univ, Tochigi, Japan*)

The hyperpolarization-activated cyclic nucleotide-gated (HCN) channels are pivotal in regulating neuronal excitability and cardiac pacemaker activity. Structurally, HCN channels are very similar to voltage-gated $K^+$ channels: They are tetrameric, and each subunit consists of a voltage-sensing domain (VSD: S1-S4) and a pore-forming domain (PD: S5-S6). Changes in membrane potential are sensed by the S4 segment, which contains several positively charged amino acids and moves across the membrane in response to changes in membrane potential. In particular, the HCN channel gate initiates an opening when S4 moves downward during periods of hyperpolarization.

HCN channels have a more extended S4 segment than other voltage-gated $K^+$ channels and have nine positively charged amino acids on S4. Positively charged amino acids in the lower part of the S4 segment are responsible for the gating charge as they are assumed to cross the charge transfer center of phenylalanine in S2. However, the function of the positive charges on the upper part of S4 remains unknown. According to the available structural information of the HCN channels, we found that the third positively charged arginine residue (R378) from the extracellular side of S4 forms a salt bridge with aspartic acid (D444) in S5, a part of the PD. Therefore, as S4 moves downward during hyperpolarization, at least three positive charges (R372, R375, and R378) could form a salt bridge with D444. To test a possible functional role of the salt bridges, we created double mutants by swapping R in S4 and D in S5. These mutants resulted in a stepwise shift in the G-V relationships, depending on the position of the mutation on the S4 side. This suggests that the three positively charged amino acids on the extracellular side of S4 and the negatively charged amino acids on S5 may form salt bridges and be involved in voltage-dependent gating. While these extracellular positive charges do not participate as canonical gating charges, they form a unique coupling with S5 and regulate the hyperpolarization gating of HCN channels.

### [2P-047]

### Probing the mechanism of sodium ion permeation in TRPV1

*Yuki Nagasato[1,2], Keisuke Sanematsu[1,3,4], Masato Yamamoto[1], Ayaka Hirayama[1], Emiko Imamura[1], Yuko Kawabata[1], Shingo Takai[1], Matsui Toshirou[2,4], Noriatsu Shigemura[1,4] ([1]*Department of Section of Oral Neuroscience, Graduate School of Dental Science, Kyushu UniversityBioresources and Biosciences, Faculty of Agriculture, Graduate School of Kyushu University,* [2]*Department of Bioresources and Biosciences, Faculty of Agriculture, Graduate School of Kyushu University,* [3]*Oral Health/Brain Health/Total Health Research Center, Graduate School of Dental Science, Kyushu University,* [4]*Research and Development Center for Five-Sense Devices, Kyushu University*)

Transient potential receptor vanilloid 1 (TRPV1) is a non-selective cation-permeable channel activated by various stimuli, including heat, acids, and compounds. Although previous research using molecular dynamics (MD) simulation reported that calcium ions permeate the channel by a knock-on mechanism in the channel pore of TRPV2, 3, 5, and 6 with the high membrane potential (>400 mV), less is known about dynamics of TRPV1 on the sodium ion permeation with membrane potential in vivo. In this study, we attempted to elucidate the permeation mechanism of sodium ions in TRPV1 by MD simulations. An open-structure human TRPV1 was generated by the homology modeling, using the squirrel TRPV1 (PDB 7LQZ) and the rat TRPV1 (PDB 7RQY) as templates, and inserted into a lipid bilayer (POPC). MD simulations were performed under 150 mM NaCl with 100 mV membrane potential close to that in vivo until 1.5 µs. We observed about 30 sodium ion permeations during simulations. In the channel pore, three binding sites of sodium ions were found, two in the selective filter (SF) and one near the gate, and two or three sodium ions bound to them were observed to permeate cooperatively. Next, to compare the energy required for ion permeation, umbrella sampling was performed and then the potential mean force was calculated by weighted histogram analysis. The free energy barrier for the sodium ion passing the gate decreased as other sodium ions were present in the SF. The results indicate that the permeation of sodium ions is likely to occur in a knock-on mechanism. Finally, to investigate the contribution of the binding site formed by N677 to permeation, three mutants, N677A, N677Q, and N677D, were constructed and 500 ns MD simulations were carried out. As a result, the average number of ions permeating the channel pore was reduced in all the mutants compared to that in the wild type, suggesting that N677 contributes to ion permeation efficiency. In conclusion, our results indicate that sodium ions permeate TRPV1 by knock-on of two or three sodium ions in the channel pore and its high permeation efficiency depends on the moderate interaction between a sodium ion and N677 in a binding site near the gate.

### [2P-048]

### Electrophysiological analysis of an anticancer drug gemcitabine on hERG channel

Mengyan Wei[1,3], *Shinichiro Kume[1], Pu Wang[1,3], Xiufang Zhu[1,3], Masaki Morishima[2], Yangong Liu[3], Mingqi Zheng[3], Gang Liu[3], Hiroki Osanai[1], Kenshi Yoshimura[1], Tatsuki Kurokawa[1], Katsushige Ono[1] ([1]*Oita University,* [2]*Kindai University,* [3]*The First Hospital of Hebei Medical University*)

Gemcitabine is an anticancer drug commonly used in the treatment of several types of cancers including pancreatic cancer, non–small cell lung cancer and breast cancer. It is widely recognized that gemcitabine induces cardiovascular adverse reactions including myocardial ischemia, pericardial diseases, heart failure, and arrhythmias. Furthermore, association of gemcitabine with QT interval prolongations in electrocardiogram was also reported, although the exact mechanism of cardiac dysfunction causing arrhythmias remains unclear. In this study, we aimed to electrophysiologically evaluate the proarrhythmic cardiotoxicity of gemcitabine focusing on the human rapid delayed rectifier potassium channel, hERG channel. To analyze effects of gemcitabine on hERG channel, we used the patch clamp technique in the human embryonic kidney 293 (HEK293) cells stably expressing the hERG channel (HEK293-hERG). The hERG channel current was reduced by gemcitabine when applied for 24 h, but not immediately after the application. Gemcitabine modified the activation gating properties of the hERG channel toward the hyperpolarization direction, while inactivation, deactivation or reactivation gating properties were unaffected by gemcitabine. When gemcitabine was applied to HEK293-hERG cells in combined with tunicamycin, an inhibitor of N-acetylglucosamine phosphotransferase, gemcitabine was unable to reduce hERG channel current or shift the activation properties toward the hyperpolarization direction. While kifunensine, a mannosidase I inhibitor, alone reduced hERG channel current and the reduction was even larger in combined with gemcitabine, kifunensine was without effect on hERG channel current when HEK293-hERG cells were pretreated with gemcitabine for 24 h. In addition, gemcitabine down-regulated fluorescence intensity for hERG potassium channel protein in rat neonatal cardiomyocyte, although hERG mRNA was unchanged. Our results suggest the possible mechanism of arrhythmias caused by gemcitabine revealing a downregulation of hERG channel current through the post-translational glycosylation disruption possibly at the early phase of hERG channel glycosylation in the endoplasmic reticulum that alters the electrical excitability of cells.

### [2P-049]

### Voltage-sensing phosphatase (VSP) in endocytosis: Insight from in vivo zebrafish model.

*Adisorn Ratanayotha[1,2], Takafumi Kawai[1], Yasushi Okamura[1] ([1]*Laboratory of Integrative Physiology, Department of Physiology, Graduate School of Medicine, Osaka University, Suita, Osaka, Japan,* [2]*Department of Anatomy, Faculty of Medicine Siriraj Hospital, Mahidol University, Bangkoknoi, Bangkok, Thailand*)

Endocytosis is a highly conserved cellular process for nutrient absorption in animal species. We recently discovered that the voltage-sensing phosphatase (VSP), a membrane protein that regulates phosphoinositide (PIP) homeostasis in biological membranes, facilitates endocytosis in lysosome-rich enterocytes (LREs) of larval zebrafish. However, the precise in vivo mechanism remains elusive. In this study, we extend our previous findings to elucidate the role of VSP in endocytosis and nutrient absorption within LREs using transgenic zebrafish that express fluorescent markers for specific endosomal membranes. We show that zebrafish VSP localizes to recycling endosomes, as demonstrated by co-localization with Rab11-GFP signals in the apical region of LREs. Additionally, VSP deficiency in LREs reduced Rab11 signal intensity and impaired endocytosis after initial uptake of dextran and mCherry. Our findings highlight the importance of VSP in endocytosis and the physiology of absorptive intestinal epithelium, shedding light on the intricate in vivo mechanisms governing nutrient absorption during development.

### Poster

### [2P]
### Molecular physiology, Cell physiology
**Others**

March 29, 13:00 - 14:20, Poster Room

### [2P-050]

### Effect of $Na^{+}$-$K^{+}$-$2Cl^{-}$cotranspoter (NKCC) expression level on $Cl^{-}$ sensitivity of cell proliferation in human esophageal squamous carcinoma cell line KYSE70

*Nodoka Nakubo[1], Hiroaki Miyazaki[1] ([1]*Department of Life Science, Faculty of Science and Engineering, Setsunan university*)

Our previous study demonstrated that effect of intracellular $Cl^{-}$ on cell proliferation of human breast cancer cell lines, KYSE70 and KYSE170. The proliferation of KYSE70 cells was not inhibited, whereas the proliferation of KYSE170 cells was significantly inhibited under low $Cl^{-}$ conditions. Since intracellular $Cl^{-}$ concentration is generally regulated by $Na^{+}$-$K^{+}$-$2Cl^{-}$ cotransporters (NKCC), which take $Cl^{-}$ into the cell, and $K^{+}$-$Cl^{-}$ cotransporters (KCC), which take $Cl^{-}$ out of the cell, we hypothesized that the differences in cell proliferative potential are due to the balances in $Cl^{-}$ transporter expression. Thus, we investigated the mRNA levels of the $Cl^{-}$ transporters in each of the cells as well as in the breast cancer cells. The results showed that KYSE70 had low NKCC1 and high KCC1 mRNA levels, whereas KYSE170 had high NKCC1 and low KCC1 mRNA levels, namely, cell growth of KYSE70 cells is less inhibited by low $Cl^{-}$ treatment than that of KYSE170 cells, even though KYSE70 cells are thought to express less NKCC1 and are less likely to maintain $Cl^{-}$ in the cell. Therefore, we investigated whether low $Cl^{-}$ treatment alters NKCC protein expression in KYSE70. The protein expression of NKCC in KYSE70 cells was detected by Western blotting. The results showed that NKCC1 expression tended to be higher in the low $Cl^{-}$ conditions than in the normal environment. In particular, the protein expression level of NKCC1 was significantly higher at 24 hours after the treatment with low $Cl^{-}$ medium. Thus, under low $Cl^{-}$ conditions, increased expression of NKCC1 may suppress the decrease in intracellular $Cl^{-}$ concentration and thus abolish the inhibitory effect on cell proliferation. We then identified the effect of $Cl^{-}$ on the expression levels of NKCC transcription factors, Six1 and NFAT5, in order to determine the mechanism of NKCC upregulation under low $Cl^{-}$ conditions. The results showed that the expression of Six1 tended to increase after 10 hours of low $Cl^{-}$ treatment.

### [2P-051]

### Measurement of activity of $Na^{+}$-$K^{+}$-$2Cl^{-}$ cotransporter in human breast cancer cell line MCF-7

*Risa Matsuda[1], Hiroaki Miyazaki[1] ([1]*Department of Life Science, Faculty of Science and Engineering, Setsunan University*)

In a previous study, we examined the effect of $Cl^{-}$ on cell proliferation in the human breast cancer cell lines MDA-MB231 and MCF-7. When MDA-MB231 and MCF-7 were cultured in normal $Cl^{-}$ concentration medium (normal $Cl^{-}$ medium) and low $Cl^{-}$ concentration medium (low $Cl^{-}$ medium) for 3 days, both cells showed reduced cell growth in the low $Cl^{-}$ medium than in the normal $Cl^{-}$ medium. When MDA-MB231 and MCF-7 cultured in low $Cl^{-}$ medium were compared, MDA-MB231 had a lower cell number than MCF-7, and cell proliferation was more inhibited than MCF-7. Further examination of NKCC and KCC mRNA levels in both cells revealed that MDA-MB231 has low NKCC expression and high KCC expression, while MCF-7 has high NKCC expression and low KCC expression. It is hypothesized that MCF-7 are able to internalize $Cl^{-}$ from NKCC even in low $Cl^{-}$ medium, whereas MDA-MB231 are unable to retain $Cl^{-}$ in their cells due to low NKCC expression, and thus cell proliferation is more suppressed. We proved this hypothesis by treating MDA-MB231 with quercetin and apigenin, which are flavonoids and promote NKCC function, and examined how cell proliferation is affected in low $Cl^{-}$ medium. We expected that treatment with quercetin or apigenin would activate the NKCC of MDA-MB231, allowing $Cl^{-}$ to be taken up by the cells even in low $Cl^{-}$ medium and increasing cell numbers compared to conditions without treatment with activators. However, treatment with quercetin and apigenin did not significantly differ from the control. This suggests that the activator may have had no effect on the cells used in this study, but the previous study only compared the mRNA levels of transporters, and it is unclear to what extent transporters are actually at work. Therefore, this study aims to confirm the effects of quercetin and apigenin, and is investigating a method to measure NKCC activity using the FluxOR potassium channel assay kit.

### [2P-052]

### The pathophysiological role of Equilibrative Nucleoside Transporters (ENTs) in esophageal cancer

*Chishou Mitsuura[1], Yu Nagayoshi[1], Hitomi Kaneko[1], Kayo Nishiguchi[1], Takeshi Chujo[1], Kazuhito Tomizawa[1] ([1]*Department of Molecular Physiology, Faculty of Life Sciences, Kumamoto University*)

RNAs have more than 100 chemical modifications. In a previous study, we found that modified nucleosides, the metabolite of modified RNAs, were finally secreted to extracellular spaces. We also found that Equilibrative Nucleoside Transporters (ENTs) are main transporters for the secretion of modified nucleosides. Moreover, modified nucleosides have many physiological activities including induction of abnormal cell proliferation. However, the pathophysiological importance of ENTs in esophageal cancer cells were almost unclear. We quantified the expression of each ENTs by RT-PCR and compared with tumor and normal areas. We found that ENT4 is significantly elevated in tumor area. It was reported that ENT4 elevated at acidic conditions. In cancer cells, the hypoxia conditions lead acidic conditions by production of lactate. To confirm the relationship between acidic conditions and ENT4, we cultured 9 types of human esophageal squamous cell carcinoma cell-lines under hypoxia conditions for 24 hours. We found that the expression levels of ENT4 were significantly elevated in 6 types of cell lines. On the other hand, chemical activation of the HIF pathway by HIF-PHI did not increase ENT4. From these results, we found that ENT4 in significantly elevated in esophageal cancer, and this elevation was induced by hypoxia conditions. These reactions may suggest that the physiological function of ENT4 regulates HIF activation.

### [2P-053]

### Role of mast cells in tissue noradrenaline distribution

*Atsushi Fujimura[1], Yusuke Otani[2] ([1]*Department of Cellular Physiology, Okayama University Graduate School of Medicine, Dentistry and Pharmaceutical Sciences,* [2]*Department of Pathology, Beth Israel Deaconess Medical Center, Harvard Medical School*)

Mast cells are granulocytes that are ubiquitous in mucosal and connective tissues throughout the body. Mast cells are known to be associated with allergic and anaphylactic reactions by synthesizing and releasing histamine and proteases in their own cell body. In mice, mast cells are classified into mucosal mast cells (MMCs) and connective tissue mast cells (CTMCs), each of which express proteases and proteoglycans in a different manner. In this study, we unexpectedly found that CTMCs store noradrenaline, a sympathetic neurotransmitter, in their cells. To examine the tissue gradient of noradrenaline, we performed immunostaining of individual organs with antibodies against noradrenaline and noticed that there was a population of cells rich in noradrenaline in the cytoplasm. By performing biochemical analyses of mast cells cultured in vitro, We confirmed that these cells do not express catecholamine synthase and that they take up and store noradrenaline externally and release it in response to ionomycin stimulation. These findings are important in considering how noradrenaline released from sympathetic nerve terminals is distributed to tissues and exerts its effects. In addition, since there have been a series of reports in recent years that noradrenaline enhances the stemness of cancer cells, we believe that our findings provide some insight into the pathophysiological role of the sympathetic nervous system involved.

### [2P-054]

### Mitohormesis reduce the neuroinflammation for attenuation of kaolin-induced hydrocephalus

*EUNGSEOK OH[4], Heonjong Yoo[1], Woosuk Chung[1], Jiebo Zhu[1,2,3], Min Joung Lee[1,2,3], Jong Hun An[1,2,3], Hyunjoo Choi[1,2,3], Changhee Pyo[1,2,3], Jun Young Heo[1,2,3] ([1]*Department of Medical science, Chungnam National University School of Medicine, Daejeon, Republic of Korea,* [2]*Department of Biochemistry, Chungnam National University School of Medicine, Daejeon, Republic of Korea,* [3]*Brain Korea 21 FOUR Project for Medical Science, Chungnam National University School of Medicine, Daejeon, Republic of Korea,* [4]*Department of Neurology, Chungnam National University Hospital, School of Medicine, Daejeon, South Korea;*)

Ventriculomegaly induced by the abnormal accumulation of cerebrospinal fluid (CSF) leads to hydrocephalus, which is accompanied by neuroinflammation and mitochondrial oxidative stress. The mitochondrial stress activates mitochondrial unfolded protein response (UPRmt), which is essential for mitochondrial protein homeostasis. However, the association of inflammatory response and UPRmt in the pathogenesis of hydrocephalus is still unclear. To assess their relevance in the pathogenesis of hydrocephalus, we established a kaolin-induced hydrocephalus model in 8-week-old male C57BL/6J mice and evaluated it over time. We found that kaolin-injected mice showed prominent ventricular dilation, motor behavior defects at the 3-day, followed by the activation of microglia and UPRmt in the motor cortex at the 5-day. In addition, PARP-1/NF-κB signaling and apoptotic cell death appeared at the 5-day. By the silencing of Atf5 in activated microglia which is an upstream molecule of UPRmt, we identify the production of pro-inflammatory cytokines interleukin-6 (IL-6), interleukin-1β (IL-1β), and tumor necrosis factor-α (TNF-α), but also decreased mitochondrial membrane potential (MMP). Our results suggest that ATF5-dependent UPRmt in the microglia acts as a protective mechanism during neuroinflammation, and may be a potential treatment target for reducing neuroinflammation, it provides a new sight for the pathogenic target of hydrocephalus.

### [2P-055]

### Pathomechanism of severe liver injury associated with X-linked myotubular myopathy (XLMTM)

*Nobuyuki Shimizu[1], Hiroshi Shiraishi[1], Masanori Inoue[2], Kyoko Kiyota[1,2], Kenji Ihara[2], Hanada Toshikatsu[1] ([1]*Oita Univ. Department of Cell Biology,* [2]*Oita Univ. Department of Pediatrics*)

X-linked myotubular myopathy (XLMTM) is primarily characterized by congenital skeletal muscle disease due to loss of function of the MTM1 gene, which encodes the protein myotubularin and regulates membrane remodeling dynamics. XLMTM is also complicated by severe liver disorders such as hepatic peliosis (a vascular disorder of the liver) and cholestasis (a disorder of the excretion of bile synthesized in the liver), which complicate the indication for AT132, an adeno-associated virus (AAV) vector-based MTM1 gene therapy. However, the pathomechanisms underlying liver injury in XLMTM remain unknown. In zebrafish, an excellent animal model for pathological imaging analysis, loss of MTM1 gene function resulted in abnormal sinusoid morphology and a cholestasis-like phenotype that mimicked XLMTM patients. Detailed analysis of liver tissue revealed that MTM1-deficient zebrafish had impaired "bile canaliculi", apical membrane domains of hepatocytes and involved in bile acid excretion, accumulation of defective mitochondria, and increased inflammation, suggesting a model in which impaired hepatocyte function induces damage to vascular endothelial cells. In this presentation, we will also review the pathological molecular mechanisms common to muscle and liver tissues in XLMTM.

### [2P-056]

### Inhibitory effect of cytoplasmic dynein inhibitors on axonemal dynein

*Kotoku Kawaguchi[1], Shiori Morimoto[1], Takatoshi Hirosawa[1], Boshi Zhao[1], Shinji Asano[1] ([1]*Dept. Mol. Physiol., Col. Pharm. Sci., Ritsumeikan Univ.*)

Dyneins are a group of the largest and most complex cytoskeletal motor proteins. Dynein heavy chain is classified as either cytoplasmic or axonemal according to its physiological function and cellular localization. Axonemal dynein (outer dynein arm (ODA) and inner dynein arm (IDA)) are localized in the motile cilia of multiciliated cells (MCCs) in the airways, brain ventricles, and oviducts, as well as in the flagella of spermatozoa, and causes ciliary and flagellar movement. The ODA regulates the ciliary beat frequency (CBF), and the IDA regulates the ciliary beat distance (CBD). In the present study, we examined the inhibitory effects of cytoplasmic dynein inhibitors (ciliobrevin D and A, and dynapyrazole A) on axonemal dynein-mediated ciliary beating in two types of MCCs: mouse ependymal cells and normal human airway epithelial cells (NHBE).Ciliobrevin D inhibits CBF with an $IC_{50}$ of 30 μM in both ependymal cells and NHBE. The CBF values were decreased by the treatment of 100 μM ciliobrevin D by 83% (ependymal cells) and 72% (NHBE), respectively. The treatment of 100 μM ciliobrevin A reduced CBF by only 38% and 41%, respectively. The treatment of 100 μM dynapyrazole A also reduced CBF by only 34% and 23%, respectively. None of the cytoplasmic dynein inhibitors inhibited IDA. These results suggests that ciliobrevin D is a useful inhibitor for ODA but not IDA, of axonal dynein. Introducing the Cl group at C7 of the A ring of ciliobrevin dramatically improves the inhibitory activity, and loss of the C2-C9 double bond can impair the inhibitory activity against ODA. The lack of inhibitory activity of ciliobrevin D in IDA may be because the most conserved amino acid sequence between ODA and IDA in AAA3 was as low as 39%. NHBE and mouse ventricular ependymal cells were used as MCCs with motile cilia, but no difference was observed in the inhibitory effects of each inhibitor on CBF and CBD between these cells. These results suggest that the inhibitory effect of cytoplasmic dynein inhibitors on axonemal dynein does not depend on species or tissue differences in MCCs. Ciliobrevin D may have the potential to be applied to the construction of a quantitative analytical model for the decrease in mucociliary clearance in the airways that mimics the pathology of primary ciliary dyskinesia (PCD) caused by axonemal dynein abnormalities.

### [2P-057]

### The relevance between the origins of vascular smooth muscle cells and $PGE_2$ receptor expression on dedifferentiation induction.

*Rihito Horiuchi[1], Takahiro Inoue[1], Hiroki Bochimoto[2], Jun Tanihata[2], Susumu Minamisawa[1,2] ([1]*The Jikei University School of Medicine, Department of Cell Physiology,* [2]*The Jikei University School of Medicine, Division of Aerospace Medicine, Department of Cell Physiology*)

### [2P-058]

### Effects of changes in intracellular $Cl^-$ concentration on the activity small G protein Ras

*Yuuri Yamashita[1], Hiroaki Miyazaki[1] ([1]*Department of Life Science,Faculty of Science and Engineering,Setsunan University*)

### [2P-059]

### In vivo analyses of hepatocytic $Ca^{2+}$ dynamics using transgenic mouse line expressing $Ca^{2+}$ sensor protein

*Kazuki Yatabe[1], Yuichi Hiraoka[2], Ino Masamitsu[1], Toshio Miki[1], Kazunori Kanemaru[1] ([1]*Department of Physiology, Nihon University School of Medicine,* [2]*Department of Neuroscience, Institute for Intractable Diseases, Tokyo Medical and Dental University*)

### Poster

### [2P]
### Embryology, Regenerative Medicine, Development, Growth, Aging

March 29, 13:00 - 14:20, Poster Room

### [2P-060]

### Regulation of RP58/ZBTB18 expression after maturation defines the integrity of cognitive function and mossy cells in the hippocampal dentate gyrus.

*Hiroko Shimbo[1,3], Shinobu Hirai[1], Kenji Tanaka[2], Haruo Okado[1] ([1]*Brain Metabolic Regulation Group, Department of Psychiatry and Behavioral Sciences, Tokyo Metropolitan Institute of Medical Science,* [2]*Division of Brain Sciences, Institute for Advanced Medical Research, School of Medicine, Keio University,* [3]*Clinical Research Institute, Kanagawa Children's Medical Center*)

**[Background]** The transcriptional repressor RP58/ZBTB18 is highly expressed in glutamatergic neurons in the cerebral cortex from embryonic development and regulates neuronal differentiation, migration, and maturation. Developmental disorders, including intellectual disability, have been reported in patients with RP58 haploinsufficiency. RP58 heterozygous mice, which mimic the above patients, show reduced working memory and synaptic abnormalities (Mol. Psychi. 2023). RP58 continues to be expressed after maturation, and its expression is reported to be markedly reduced in the human brain with aging. However, the phenotypic impact of RP58, which decreases after maturation, is unknown. Therefore, in this study, we used model mice in which RP58 expression was artificially reduced from early adulthood (2 months old), and histological analysis and cognitive tests were performed in late adulthood (4-5 months old). **[Methods]** Actin-tTS:Rp58 tetO/tetO or tetO/+ mice, in which Rp58 expression can be reversibly controlled by doxycycline (Dox). Dox was administered until 2 months of age to maintain RP58 expression at normal levels. Then, Dox was removed after 2 months of age, and RP58 expression was artificially decreased. At 4-5 months of age, cognitive function tests such as the object location test (OLT) and the novel object recognition test (NORT) were conducted, as well as histological analysis. **[Results]** OLT and NORT showed that control mice (Rp58 tetO/tetO or tetO/+) spent longer time to explore objects moved to novel locations or novel objects, similar to wild-type mice of the same age, which means normal cognitive function. On the other hand, mice with reduced expression of Rp58 from the early adult stage (Actin-tTS:Rp58 teto/+ and Actin-tTS:Rp58 teto/teto) had shorter time to explore novel-located objects and novel objects, similar to RP58 heterozygous mice at the same age and similar to wild-type old mice, which means impairment of cognitive function. Histological analysis revealed morphological abnormalities such as loss of dendrites and cytoplasmic vacuolation of mossy cells in the hippocampal dentate gyrus. **[Discussion]** In mice in which RP58 expression was reduced from early adulthood, marked abnormalities of mossy cells of the hippocampal dentate gyrus and impaired cognition were observed in late adulthood. This phenotype was similar to that of aging mice. Thus, RP58 is an important factor for maintaining brain function during adulthood, independent of development, suggesting that declined expression of RP58 during aging might be a cause of impaired cognitive function in aging.

### [2P-061]

### Fabrication of high-strength multi-layered cell sheets derived from vascular smooth muscle cells using hydrostatic pressure under hypoxia.

*Takashi Nakamura[1], Tomoyuki Kojima[1,2], Yuko Hidaka[1], Etsuko Miyagi[2], Yoshihiro Ishikawa[2], Utako Yokoyama[1] ([1]*Tokyo Medical University,* [2]*Yokohama City University*)

Prosthetic vascular grafts are used for the treatment of congenital cardiovascular disease. However, there are limitations to this therapeutic strategy because of thrombogenicity and the lack of growth potential. Thus, biologically compatible tissue-engineered vascular grafts (TEVGs) are desired. In this study, we investigated whether hydrostatic pressure under hypoxia facilitates the fabrication of biocompatible TEVGs using human vascular smooth muscle cells (VSMCs). We cultured human umbilical cord artery smooth muscle cells with hydrostatic pressure (110-180 kPa, 0.002 Hz) under hypoxia ($PO_2$ = 70 mmHg)(HP/HYP), and we performed RNA-sequencing, western blotting, and immunocytochemistry. We then fabricated VSMC grafts using HP/HYP during cell seeding and evaluated the mechanical properties and immunohistochemistry. Finally, we implanted the VSMC grafts in rat abdominal aorta, and the implantation site was assessed with the histological method. In the RNA-sequencing, the expression of 20 genes was increased more than 2-fold by HP/HYP compared with atmospheric pressure under normoxia (AP/NOR). In these genes, we mainly focused on N-myc downstream-regulated gene-1 (NDRG1), which has been reported to enhance membrane expression of the adherence junction components. In the immunocytochemistry, N-cadherin, which regulates primarily cell-cell adhesion of VSMCs, was localized at the cell-cell junctions by HP/HYP, and *NDRG-1*-targeted siRNA attenuated the HP/HYP-induced localization of N-cadherin. HP/HYP also increased integrin α5β1 expression and promoted fibronectin fibrillogenesis. Furthermore, HP/HYP significantly increased collagen production (3.72±0.08-fold, n=5-6, $p$<0.05) and the protein expression of lysyl oxidase (1.34±0.03-fold, n=5-6, $p$<0.05), which is an essential enzyme for cross-linking of collagens. The tensile rupture strength of VSMC grafts was 2132±227 mmHg (n=5). VSMC grafts were implanted in rat abdominal aorta and withstood arterial blood pressure. Implantation sites were patent 5 months after implantation without thrombosis and aneurysmal formation. These findings indicated that HP/HYP enables the fabrication of high-strength and biocompatible TEVGs with a spatial arrangement of VSMCs and extracellular matrices.

### [2P-062]

### Involvement of Myeloid-derived growth factor in zebrafish optic nerve regeneration process

*Yuya Omori[1], Takumi Mokuya[1], Kayo Sugitani[1] ([1]*Division Health Science, Graduate School Medical Science, Kanazawa University*)

In fish, even when the optic nerve is damaged, retinal neurons avoid apoptosis and the retinotectal connection can be re-established within one month after optic nerve injury. Thus, the zebrafish optic nerve is useful as a central nervous system regeneration model for analyzing various regeneration-related molecules.

This study focused on myeloid-derived growth factor (MYDGF) in the zebrafish optic nerve regeneration process, especially within 24 hours after optic nerve injury. MYDGF is a secreted protein composed of 173 amino acids in humans and was originally found as a molecule that protects cardiomyocytes from apoptosis during myocardial infarction. Real-time PCR analysis showed that the peak of mRNA expression of MYDGF in the zebrafish retina was 1 hour after optic nerve injury. Immunohistochemical studies showed that MYDGF protein expression localized in the outer nuclear layer, the outer plexiform layer, and the inner plexiform layer.

To investigate the function of the expression of MYDGF after optic nerve injury, we knocked it down using gene-specific morpholino (MO). As a result, there was a significant increase in apoptosis-positive cells in the MYDGF-knockdown group compared to the control morpholino-treated group. Further, the expressions of Bcl-2 mRNA were clearly suppressed 1 hour and 6 hours after optic nerve injury. These results suggested that MYDGF is involved in neuronal survival in the injured retina in the acute phase after optic nerve injury.

### [2P-063]

### Isolation of PRRX1-positive neural crest-derived cells in hair follicles

*Kaito Hosoda[1], Masashi Higuchi[1] ([1]*Laboratory of Veterinary Biochemistry, Joint Department of Veterinary Medicine, Faculty of Agriculture, Tottori University*)

### [2P-064]

### Involvement of Notch Signaling in Inducing Differentiation of Vasopressin Neurons

*Moeka Kuno[1], Tsukushi Suzuki[1], Rika Umemoto[1], Shunya Tsukamoto[1], Yoshinari Mera[1], Miho Kawata[1], Yu Kodani[1], Kanako Saito[1], Akira Nakashima[2], Toshiki Kameyama[1], Hiroshi Nagasaki[1] ([1]*Department of Physiology , School of Medicine , Fujita Health University,* [2]*Department of Physiological Chemistry, School of Medicine , Fujita Health University*)

### [2P-065]

### Induction of mouse ES cells into hypothalamic arcuate nucleus nerves by gene transfer of transcription factors

*Rika Umemoto[1], Shunya Tsukamoto[1], Yoshinari Mera[1], Moeka Kuno[1], Tsukushi Suzuki[1], Miho Kawata[1], Yu Kodani[1], Kanako Saito[1], Akira Nakashima[2], Toshiki Kameyama[1], Hiroshi Nagasaki[1] ([1]*Department of PhysiologyI, School of Medicine , Fujita Health University,* [2]*Department of Physiology Chemistry , School of Medicine , Fujita Health University*)

### Poster

### [2P]
### Muscle

March 29, 13:00 - 14:20, Poster Room

### [2P-066]

#### Effect of neurotransmitters, beta-agonists and calcitonin gene related peptide (CGRP), on expression of myosin heavy chain class $II_a$ ($MyHCII_a$) mRNA in mouse skeletal myocytes

*Junko Tashiro Yamaji[1], Yoshiaki Mori[1] ([1]*Dept. of Rehab., Kansai Univ. of Welf. Sci.*)

Neurotransmitters including catecholamine and calcitonin gene related peptide (CGRP) from motor nerves release to postsynaptic neuromuscular receptors on the myoneural junction and also activates adenylyl cyclase pathway in the skeletal muscle cells. However, the effects of these neurotransmitters and the cAMP pathway on mRNA levels of MyHCs in skeletal muscle still remain unknown. In this study, we examined that these ligands, catecholamine and CGRP, on mRNA expression of $MyHCII_a$ and IL-6 in mouse myocytes. Then our study yielded the following results: (1)The mRNA level of $MyHCII_a$ was significantly upregulated by calcineurin activators but not by IL-6, and was significantly decreased by calcineurin inhibitor. (2)The $MyHCII_a$ mRNA level was decreased by medium containing CGRP. (3)The $MyHCII_a$ mRNA level was significantly upregulated by medium containing cAMP and with beta-2 agonist but was not effected by that medium containing PKA inhibitor.These results indicated that cAMP pathway is affected for $MyHCII_a$ mRNA level.

### [2P-067]

#### $Ca^{2+}$ influx through slow muscle-type nicotinic AChR contributes to slow muscle contraction.

*Buntaro Zempo[1], Fumihito Ono[2], Koichi Nakajo[1] ([1]*Jichi medical university,* [2]*Osaka Medical and Pharmaceutical University*)

Skeletal muscle in vertebrates consists of two types of muscle fibers: slow muscle and fast muscle. Recent studies in zebrafish found that the subunit composition of nicotinic acetylcholine receptor (AChR) in the neuromuscular junction of slow muscle is different from that of fast muscle. In zebrafish as well as in mammals, AChR in fast muscle is composed of α, β, δ, and ε (or γ) subunits. On the other hand, AChR in the slow muscle of zebrafish lacks ε (or γ) and is composed of only α, β, and δ subunits. However, the physiological significance of slow muscle-type AChR has not been understood. In the present study, we compared the channel properties of slow and fast muscle-type AChRs expressed in *Xenopus* oocytes by two-electrode voltage clamp. We found that the slow muscle-type AChR shows much higher $Ca^{2+}$ permeability than the fast muscle-type one. To clarify the physiological functions of the $Ca^{2+}$ influx through the slow muscle-type AChR, we mutated the pore-lining Glu (E) of the d subunit, which is considered to be a key amino acid residue for $Ca^{2+}$ permeability, to Gln (Q). We confirmed that the AChR containing the mutant d subunit lost the $Ca^{2+}$ permeability in *Xenopus* oocytes. Next, we generated a transgenic zebrafish line that expresses the mutant d subunit in slow muscle. The slow muscle cells of this transgenic zebrafish lose the $Ca^{2+}$ permeability, allowing us to analyze the physiological roles of the $Ca^{2+}$ influx through slow muscle-type AChRs on locomotor activity. We recorded the startle response of the transgenic zebrafish using a high-speed camera. As a result, the turn angles of bodies during the startle response of the transgenic zebrafish were significantly smaller than those of the wild type. In addition, swimming speed was slower in the transgenic zebrafish than in the wild type. To more precisely analyze the effects of mutation on slow muscles, we generated the transgenic line lacking all muscle-type AChRs except the $Ca^{2+}$-impermeable slow muscle-type AChRs. In other words, this transgenic zebrafish swims only with the $Ca^{2+}$-impermeable slow muscle-type AChRs. We compared its locomotor activities with the γ/ε subunit double knockout line (DKO) that expresses only $Ca^{2+}$-permeable slow muscle-type AChR. We found that the turn angles of the bodies of the transgenic zebrafish were smaller than those of DKO. These results suggest that $Ca^{2+}$ influx through slow muscle-type AChR significantly contributes to contracting slow muscle cells.

### [2P-068]

#### Age-related structural deteriorations of muscle spindle in mice.

*Minako Takaishi[1], Ken Watanabe[1], Tohru Hosoyama[1] ([1]*Department of Musculoskeletal Disease, Research Institute, National Center for Geriatrics and Gerontology*)

**Background:** Proprioceptive information is essential for normal gait and the maintenance of a stable posture. The muscle spindle (MS), which is a crucial proprioceptor in skeletal muscle, conveys the information of changes in both length and stretching speed of muscles to the central nervous system. Although age-related alterations in the proprioceptive system are thought to be related to the risk of falling and low back pain in older adults, it is unclear how aging affects a structure and function of MS. In this study, we aimed to clarify age-related structural changes of MS particularly focusing on spindle capsule, intrafusal fibers (IFs), and annulospiral endings of afferent.
**Methods:** Soleus (SOL) and extensor digitorum longus (EDL) muscles were dissected from young (3-month-old) and aged (26-month-old) C57BL/6N mice (n=6). Cryo-sections of longitudinal direction (150-μm thickness) from both muscles were provided to immunofluorescent labelling with the appropriate antibodies to visualize MS structure. Using confocal z-stack images from the equatorial region of MS, the structural features were compared between young and aged mice.
**Results:** There were no significant differences in the number and diameter of both MSs and IFs between young and aged mice, indicating no age-related atrophy in MS and IF. Whereas annulospiral endings were well organized in young mice, the number of coils surrounding IFs were significantly decreased in aged mice. Accordingly, the percentage of unraveled-afferents (less than 10 coils in the IFs) were markedly increased in aged mice (SOL: young=14.0±3.9% vs. aged=52.2±11.9%, EDL: young=4.2±4.6% vs. aged=51.3±14.9%, P<0.01). In addition, an area of terminal endings of afferent nerve was significantly smaller in aged mice than that in young (SOL: young=964±97$\mu m^2$ vs. aged=626±87$\mu m^2$, P<0.01, EDL: young=1233±117 $\mu m^2$ vs. aged=798±308 $\mu m^2$, P<0.05). Taken together, it is suggested that denervation causes deterioration of MS in aged mice.
**Conclusion:** Our findings show that age-related structural deterioration of afferents without IF atrophy could contribute to disfunction of proprioception in aged-muscle.

### [2P-069]

### Development of EAD in heart cells involves reverse E-C coupling and reverse electrotonic conduction along T-tubules.

*Takao Shioya[1] ([1]*Department of Physiology, Faculty of Medicine, Saga University*)

Early after depolarization (EAD) underlies the development of life-threatening ventricular arrhythmias. Since EADs develop preferentially in damaged heart cells with abnormal $Ca^{2+}$-signaling, I studied the causal link between the development of EADs and aberrant intracellular $Ca^{2+}$ ($[Ca^{2+}]_i$) dynamics, using nystatin "superforated-patch" recording technique and $[Ca^{2+}]_i$ imaging by Fluo 4-AM in mouse heart cells. My results show: 1) The generation of EADs was preceded by the development of depolarizing membrane potential ($V_m$) fluctuation. 2) The depolarizing $V_m$ fluctuation occured concurrently with a local brief $[Ca^{2+}]_i$ elevation, and the $V_m$ fluctuation was eliminated when $Ni^{2+}$ was used to block the $Na^+/Ca^{2+}$ exchanger. 3) The generation of the $V_m$ fluctuation and EADs were suppressed when the T-tubule system of was detubulated. 4) Abbreviating the T-tubule's length constant by increasing the extracellular $K^+$ level suppressed the $V_m$ fluctuation and EADs accordingly. I conclude that EADs are caused by the depolarizing $V_m$ fluctuation, which is induced locally in the T-tubule membrane by aberrant $[Ca^{2+}]_i$ elevation and is conducted electoronically along the T-tubules back to the surface membrane.

### [2P-070]

### Upregulation of Myomaker in hypertrophic C2C12 myotubes following knockdown of ryanodine receptor 3

*Rika Ito[2,1], Huikai Zhang[1], Yusuke Takeo[1], Tatsuro Egawa[4,1], Atsushi Asakura[3], Katsumasa Goto[1] ([1]*Department of Physiology, Graduate School of Health Sciences, Toyohashi SOZO University,* [2]*Nagoya Sangyo University,* [3]*Stem Cell Institute, University of Minnesota School of Medicine,* [4]*Graduate School of Human and Environmentl Studies, Kyoto University*)

Recently, we have demonstrated that knockdown of ryanodine receptor3 (Ryr3) induces hypertrophic fusion of mouse myoblast-derived C2C12 cells. Muscle-specific membrane proteins Myomaker and Myomixer (also called Myomerger and Minion) are known as fusogenic regulators. Myomaker and Myomixer cooperatively drive myoblast fusion. It is generally considered that Myomaker functions at the hemi-fusion phase, whiles Myomixer facilitates the progression and completion of fusion. Thus, Myomaker and Myomixer may impact on myotubes size. However, there is no evidence showing the functional linking between Ryr3 and these fusogenic regulators. The purpose of this study was to investigate a potential role of muscle-specific fusogenic proteins. Therefore, we assessed expression level of Myomaker and Myomixer in Ryr3 knockdown-associated hypertrophic myotubes of C2C12 cells. Ryr3 in C2C12 cells was knocked down by siRNA 3 days after the initiation of differentiation. Myomaker was significantly upregulated by Ryr3 knockdown ($p<0.05$). On the other hand, Ryr3 knockdown suppressed the expression level of Myomixer ($p<0.05$). Results indicates that hypertrophic myotubes associated Ryr3 knockdown may be attributed to the upregulation of Myomaker and/or the uncoupling of Myomaker and Myomixer.
This study was partially supported by JSPS KAKENHI (18H03160, K.G.; 19K22825, K.G.; 19KK0254, K.G.; 22H03474, K.G.; 22K19722, K.G.; 22H03319, K.G.; 22K18413, K.G.), a grant from DAIKO FOUNDATION (K.G.), Graduate School of Health Science, Toyohashi SOZO University (K.G.), and a research grant from Toyohashi SOZO university (K.G.). All authors declare no COI associated with this study.

### [2P-071]

### Promoting effect of sigma-1 receptor on the skeletal muscle differentiation and regeneration

*Hiroshi Todaka[1], Mikihiko Arikawa[2], Tatsuya Noguchi[3], Atsushi Ichikawa[1], Takuma Higuchi[4], Shuji Sakamoto[4], Takayuki Sato[1] ([1]*Department of Cardiovascular Control, Kochi Medical School, Nankoku, Kochi, Japan,* [2]*Department of Biological Sciences, Faculty of Science and Technology, Kochi University, Akebono, Kochi, Japan,* [3]*Department of Cardiology and Geriatrics, Kochi Medical School, Nankoku, Kochi, Japan,* [4]*Laboratory of Molecular Biology, Science Research Center, Kochi Medical School, Nankoku, Kochi, Japan*)

The sigma-1 receptor (Sig1R), a chaperone protein, is known to play a crucial role in various cellular activities such as the suppression of inflammation and apoptosis by regulating endoplasmic reticulum (ER) stress. Genetic mutations in Sig1R cause distal neuropathy with muscle atrophy and weakness. However, the physiological function of Sig1R in skeletal muscle remains unclear. In this study, to elucidate the function of Sig1R in skeletal muscle, we examined the expression levels of PAX7, a marker protein of satellite cells (skeletal muscle stem cells), and myogenic regulatory factors such as myogenin and myosin heavy chain (MyHC) using a mouse model of skeletal muscle injury/regeneration and C2C12 cell line as a model of myoblast differentiation. As results, in the mouse model of skeletal muscle injury/regeneration, the expression level of Sig1R reached its peak on day 4 of muscle regeneration, followed by a gradual decrease. On day 4 of muscle regeneration, along with Sig1R, the expression levels of PAX7 and myogenin were increased. This suggests that Sig1R expression is increased in proliferated and differentiated satellite cells and myoblasts during skeletal muscle regeneration. To verify this, we measured the expression of Sig1R in differentiation-induced satellite cells and C2C12 myoblast cells. The result showed that the expression of Sig1R was increased in the early stage of muscle differentiation and decreased in the late stage. Next, we examined the effect of knocking down Sig1R on muscle differentiation in C2C12 cells. The result showed that the expression levels of myogenin and MyHC were suppressed and the fusion index level was decreased in Sig1R knockdown C2C12 cells, indicating the ability of Sig1R to promote muscle differentiation and myotube formation. Finally, we investigated the effect of Sig1R agonist in the mouse model of skeletal muscle injury/regeneration. The result showed that the expression levels of myogenin and MyHC were elevated, indicating that Sig1R regulates skeletal muscle differentiation and regeneration in vivo. These results suggest that the elevation of Sig1R promotes muscle differentiation and regeneration by increasing the expression levels of myogenin and MyHC in the early stage of the skeletal muscle differentiation process. This study further investigated the involvement of the ER stress pathway in the Sig1R-promoting skeletal muscle differentiation and regeneration.

### [2P-072]

### Effects of contraction mode and intensity on signal protein activity and sarcomere structure in mildly atrophic muscle

*Kazuhiro Hirano[1], Hideki Yamauchi[1], Naoya Nakahara[1], Maki Yamaguchi[1], Tomonori Hayashi[1], Shigeru Takemori[1] ([1]*The Jikei University School of Medicine*)

Sarcopenia is a current serious social issue, and safe and effective training prescriptions for the elderly are awaited. We consider training program with eccentric contraction (ECC) would effectively strain skeletal muscle sarcomere to trigger muscle strengthning effects without imposing excessive physical stress in the infirm elderly. However, intence strain in sarcomere would also damage atrophied muscle to exacerbate muscle weakening. Our previous study has shown that ECC of low intensity triggered protein signaling for muscle strengthening without inducing even a microscopic deterioration in sarcomere in healthy muscle. In the present study, we extend our precious study to evaluate the effects of low-intensity ECC in mildly atrophic muscle induce by tail suspension.

Plantaris muscles with maintained blood perfusion of 7-week rats after 3-days tail suspension were divided into control group (CON), isometric contraction group (ISO), low-intensity ECC group (L-ECC), and high-intensity ECC group (H-ECC). The stimulation frequencies were 50 Hz for L-ECC, 100 Hz for ISO and H-ECC. L-ECC muscles received tetanus stimulation of 0.3 sec duration iterated 30 times every 3 seconds. The iteration number of ISO and H-ECC were adjusted to match its tension-time integral to that of L-ECC.

After each contraction load, signal proteins were detected or skinned fibers were prepared and the sarcomere structure of the fibers was evaluated by X-ray diffraction.

After 3-days of tail suspension, we confirmed that the weight of the plantaris muscle was significantly decrease compared with healthy muscle. Amog signaling proteins, both mTORC1 and MAP kinase were significantly more active in L-ECC and H-ECC than CON and ISO. In X-ray diffraction, no macroscopic deterioration in sarcomere structure was detected, and both myosin and troponin reflection intensities were significantly decreased in H-ECC than CON.

In conclusion, L-ECC may be able to induce muscle hypertrophy signals preserving sarcomere structure.

### [2P-073]

### Trial to develop a rat model of muscle cramps/spasms using infusion of hypertonic saline

*Toru Taguchi[1,2], Ota Hiroki[1,2], Ueda Manami[3], Akagi Junji[3] ([1]*Dept. Phys. Ther., Fac. Rehabil., Niigata Univ. Health Welfare,* [2]*Inst. Hum. Mov. Med. Sci., Niigata Univ. Health Welfare,* [3]*Central R&D Lab., Kobayashi Pharmaceutical Co., Ltd.*)

Muscle cramps/spasms are characterized by involuntary muscle contractions that cause severe pain, and they occur in people during sleep or in athletes during competition. These are recurring phenomena, and there are no adequate treatment or prevention methods. To date, the pathological mechanism has not been elucidated, and no experimental model useful for basic research has been established. In this study, we attempted to create a novel experimental model that mimics clinical muscle cramps/spasms using electromyographic (EMG) activity induced by intramuscular infusion of hypertonic saline (HS). Under isoflurane anesthesia, an insulated needle-type recording electrode that was electrified only at the tip was inserted into the rat tibialis anterior (TA) muscle belly at a depth of 3 mm. Using a 30-gauge injection needle, HS was continuously administered to the TA about 1.5 mm distal from the recording electrode. In experiment 1, 5% HS or 0.9% physiological saline (PS) was administered at a volume of 100 µl for 1 min. In experiment 2, different amounts of HS (10, 30, 50, 100 µl/min) were administered intramuscularly in the same manner as experiment 1. In both experiments 1 and 2, EMG activity was observed and recorded for 10 min. In experiment 1, the net number and amplitude of EMG events were significantly higher in the HS group than in the PS group. In experiment 2, the net number and amplitude of EMG events increased roughly in a dose-dependent manner, and reached a maximum at 100 µl/min. Since previous studies have reported that pain induced by intramuscular injection of HS in humans is typically described as "cramping", and that C-fiber muscle nociceptors showed remarkable excitation in all cases when HS is injected into rat muscles. Taken together, it is considered that the HS-induced EMG activity observed in this study well reproduces the phenomenon of involuntary muscle contraction accompanied by pain in human muscle cramps/spasms, and that it is useful as a basic research model. This work was supported by the collaborative research grant provided by Kobayashi Pharmaceutical Co., Ltd.

### Poster

[2P]

**Digestion, Digestive system**

March 29, 13:00 - 14:20, Poster Room

### [2P-074]

### Identification of neural pathways affecting descending serotonergic neurons that regulate defecation.

*Natsufu Yuki[1], Tomoya Sawamura[1], Kazuhiro Horii[2], Hiroshi Yamaguchi[3], Akihiro Yamanaka[3], Takahiko Shiina[1,4], Yasutake Shimizu[1,4] (*[1]Department of Basic Veterinary Science, Laboratory of Physiology, Joint Graduate School of Veterinary Sciences, Gifu University., [2]Division of Biological Principles, Department of Physiology, Graduate School of Medicine, Gifu University, [3]Department of NeuroscienceII, Research Institute of Environmental Medicine, Nagoya University, [4]Laboratory of Physiology, Joint Department of Veterinary Medicine, Faculty of Applied Biological Sciences, Gifu University*)

<Background>The mechanism by which acute stress elicits defecation reflex is not well understood. It has been reported that descending serotonergic neurons projecting from the medullary raphe to the spinal defecation center regulate colorectal motility and are involved in stress-induced defecation. <Aim>In this study, we focused on the neurons projecting to the medullary raphe to identify the neural pathways that cause stress-induced defecation. <Methods>Rats were injected FluoroGold (FG), a retrograde tracer, into the medullary raphe to label neurons projecting to the medullary raphe nucleus. c-Fos expression was examined immunohistochemically to identify brain regions activated in response to water avoidance stress. To verify whether the identified regions regulate colorectal motility, we then microinjected of (S)-AMPA, a glutamate agonist, to the brain regions in urethane-anesthetized rats and recorded colorectal motility. Colorectal motility was assessed by inserting cannulas into the distal colon and anus and recording intraluminal pressure and expelled fluid volume in vivo. In addition, we used the chemogenetic technology DREADD (Designer Receptors Exclusively Activated by Designer Drugs) to control the activity of specific neurons. We expressed an inhibitory DREADD, hM4Di, in neurons projecting from the paraventricular hypothalamic nucleus (PVH) or the dorsomedial hypothalamus (DMH) to the medullary raphe. <Results>In the hypothalamus, FG-positive cells were observed in the PVH and the DMH. These FG-positive cells exhibited a significant increase in c-Fos expression by water avoidance stress. When (S)-AMPA was unilaterally administered to the PVH or the DMH, a marked increase in colorectal intraluminal pressure that was associated with increased expelled fluid was observed. The injection-induced increase in colorectal motility was suppressed by prior administration of serotonergic inhibitors into the lumbosacral spinal cord. Specific inhibition of the PVH→medullary raphe pathway or the DMH→medullary raphe pathway suppressed the injection-induced increase in colorectal motility in anesthetized rats and the water avoidance stress-induced defecation in conscious rats. <Conclusions>Stress-responsive neurons projecting from the PVH and the DMH to the medullary raphe activate descending serotonergic neurons that project to the spinal defecation center and enhance colorectal motility. These pathways are suggested to be novel neural pathways inducing stress-induced defecation.

### [2P-075]

### Actions of purple sweet potato extracts on amylolysis and glucose absorption in mice isolated small intestine

*Tomoo Homma[1], Mika Kobayashi[1], Yuka Suzuki[2], Ryujiro Shiina[2] (*[1]Maebashi Institute of Technology, [2]Nichino Kagaku Kogyo Co.,Ltd.*)

Purple sweet potato extracts (PSPE) made by Nichino Kagaku Kogyo include much quantity of polyphenols (main component is anthocyanidin). We reported that transmural potential generated by glucose (Glu) application in isolated everted sacs of small intestine of mice became temporarily small by PSPE application. That is, PSPE induced temporary inhibition of Glu absorption. In this case, we observed effects of PSPE on Glu absorption at early stage (within 5 min) since Glu application. When we examined absorbed Glu quantities under 0.8% or 1.2% PSPE condition for 20 min in the present study, PSPE didn't inhibit Glu absorption. This result may suggest that PSPE has a prolonged effect on Glu absorption. Moreover, we examined effects of PSPE on amylolysis. Everted sac specimens were dipped in Ringer solution including 0.5% soluble starch for 20 min under 0.6% or 1.2% PSPE, and Glu formation and its absorbed quantities were investigated. Both 0.6% and 1.2% PSPE condition inhibited amylolysis about 75%. Inhibitory effect of amylolysis became weak by 1.2% PSPE treated with polyvinylpolypyrrolidone (for removing polyphenol partially). These results showed polyphenols in PSPE were related with inhibition of amylolysis. Research about inhibitory mechanism and related components in PSPE has been studied.

### [2P-076]

### Ratio-metric fluorescent $Ca^{2+}$ sensor revealed the spatial gradient of basal $Ca^{2+}$ concentration in the stomach of mice

*XIN ZHANG[1], Shinsuke Nakayama[1], Naoko Iwata[1], Chiho Takai[1] (*[1]Cell Physiology Graduate School of Medicine Nagoya University*)

Spontaneous electric rhythms drive gastrointestinal movement. Network-forming pacemaker cells, referred to as interstitial cells of Cajal (ICCs) are distributed in the myenteric region of the gastrointestinal tract. In the stomach, pacemaker potentials known as slow waves propagate from the oral to the anal end at regular intervals, thereby mixing, and roughly digesting food. However, the mechanism underlying the oral-to-anal propagation of pacemaker potentials is still unknown. Calcium ions ($Ca^{2+}$) play a crucial role in regulating cellular excitation in numerous organs, including the gastrointestinal tract. In this study, we utilized transgenic mice selectively expressing a genetically encoded ratio-metric $Ca^{2+}$ indicator (YC-Nano50) in skeletal and smooth muscles to measure $[Ca^{2+}]_i$. CFP and YFP fluorescence images of YC-Nano50 were acquired separately to evaluate fluorescence resonance energy transfer (FRET), which reflects $[Ca^{2+}]_i$. This enabled the YFP/CFP ratio to be compared as a $[Ca^{2+}]_i$ index between different regions. We found a gradient of the YFP/CFP ratio. In our optical set-up of FRET measurement (W-VIEW Gemini and ORCA-ER, Hamamatsu Photonics), the basal YFP/CFP ratio was ~ 1 near the esophagus, 0.6-0.8 in the corpus and 0.3-0.5 in the antrum and pylorus regions. Atropine and TTX almost changed the basal YFP/CFP ratio within the range of variation. From the results, it was considered that the regional difference of $[Ca^{2+}]_i$ between the corpus and antrum/pylorus is not under neural control. Future investigation is required to elucidate the spatial control mechanism. It is known that spontaneous $[Ca^{2+}]_i$ oscillations evoke inward currents by activating $Ca^{2+}$-activated $Cl^-$ channels in pacemaker cells. Since pacemaker ICCs and smooth muscle cells form functional syncytia, pacemaker cells connected to smooth muscle cells with higher basal $[Ca^{2+}]_i$ in the corpus are likely more frequently activated than those in the antrum/pylorus. The gradient of basal $Ca^{2+}$ concentration may have a crucial effect on the propagation of the stomach.

### [2P-077]

### The sex differences in the enhancement of the colorectal motility caused by a ghrelin receptor agonist

*Shumpei Tsukamoto[1], Tomoya Sawamura[1], Natufu Yuki[1], Kazuhiro Horii[2], Takahiko Shiina[1], Yasutake Shimizu[1,3] ([1]*Laboratory of Physiology, Joint Graduate School of Veterinary Sciences, Gifu university,* [2]*Division of Biological Principles, Department of Physiology, Graduate School of Medicine, Gifu University,* [3]*Center for One Medicine Innovative Translational Research (COMIT), Gifu University*)

We have previously reported that there are sex differences in the central control mechanism of colorectal motility. A ghrelin receptor agonist (GA), which is expected to be a new constipation reliever, acts on the spinal defecation center, enhancing colorectal motility in male rats. However, it is unclear whether it exerts the same effect in females. In this study, we aimed to clarify sex differences in the enhancement of the colorectal motility caused by GA and elucidate its mechanism. Eight-week-old Sprague-Dawley rats were anesthetized, cannulas were placed in the distal colon and anus, and colorectal motility was evaluated in vivo. GA (RaQualia Pharmaceutical Co., Ltd.) dissolved in saline was administered intravenously or intrathecally to the L6-S1 level of the spinal cord, and changes in intraluminal pressure and expelled volume from the anus were measured. Intravenous administration of GA enhanced colorectal motility in male rats. In female rats, higher dose of GA was required to induce the same level of enhancement of colorectal motility in males. The results shows that there are sex differences in effect of GA on colorectal motility. The sex differences in effects of GA were observed even when GA was administered intrathecally to the lumbosacral spinal cord, suggesting that there is no sex difference in the process by which GA is transferred into the spinal cord. Furthermore, when ghrelin peptide was administered intrathecally, similar sex difference was observed. Previous studies have revealed that the GABAergic inhibitory transmission to the preganglionic neurons of the sacral parasympathetic nucleus in the spinal cord operated remarkably in female rats. Therefore, we examined the possible involvement of GABAergic neurons in the sex differences in the prokinetic effect of GA. When a GABA receptor inhibitor was intrathecally administered into the lumbosacral spinal cord, lower doses of GA effectively enhanced colorectal motility in females as well. These results suggest that GABA suppresses the spinal defecation center constitutively in females and the GABAergic inhibition may attenuate the action of GA. The sex differences found in this study may provide valuable information for the use of GA as a constipation reliever in future.

### [2P-078]

### Effects of short-chain fatty acids on transepithelial ion transport and ion permeability in the mice small intetine

*Shin-Ichiro Karaki[1] ([1]*Laboratory of Physiology, Department of Environmental Life Sciences, University of Shizuoka*)

Short-chain fatty acids (SCFAs), which are predominantly 2-6 carbon normal carboxylates produced by enterobacterial fermentations of dietary fiber and oligosaccharides, have been known to induce the fluid secretion in the mucosa and to affect the smooth muscle motility by stimulating from large intestinal lumen. In small intestine, amount of the microbiota keeps extremely low level, so that the concentrations of SCFAs also keeps extremely low. However, the condition of small intestinal bacterial overgrowth (SIBO) has been reported to occur, e.g., by such as excess intake of FODMAP diet, and to induce abdominal symptoms including like abdominal bloating, diarrhea, and constipation. These symptoms might be induced by stimuli of bacterial metabolites, such as SCFAs. However, there has been few reports investigating the effects of SCFAs on the functions of small intestine. Therefore, in the present study, effects of SCFAs on small intestinal fluid secretion and mucosal ion permeability were investigated by using mice small intestinal mucosa-submucosa preparations mounted on Ussing chambers. As results, acetate, which has been reported to induce no secretory response in the large intestine, induced a transepithelial electrogenic anion secretion (as an index of fluid secretion) as much as propionate. Moreover, SCFA induced an increase in transepithelial ion permeability in the small intestine, except for the jejunum, the same as in the large intestine. Interestingly in the jejunum, SCFAs decreased the transepithelial ion permeability. The present results showed that the mode of effects of SCFAs on the small intestine was different from its on the large intestine. Therefore, further studies of the effects of SCFAs on small intestinal mucosal functions should be performed. These results may reveal the abdominal symptoms indued by SIBO.

### [2P-079]

### Alteration of gut microbiota diversity by proteinase-activated receptor 1 antagonist in the experimental model of colitis-associated tumorigenesis

*XIAODONG LI[1], Lin-Hai Kurahara[1], Zhixin Zhao[3], Feiyan Zhao[3], Ryo Ishikawa[2], Gaopeng Li[1], Zhihong Sun[3], Katsuya Hirano[1] ([1]*Dept. Cardiovasc. Physiol., Fac. Med., Kagawa univ., Japan,* [2]*Dept. Diagnostic Pathology, Kagawa Univ Hospital, Kagawa univ., Japan,* [3]*Inner Mongolia Key Laboratory of Dairy Biotechnology and Engineering; Ministry of Education, Inner Mongolia Agricultural University, Hohhot, Inner Mongolia, China*)

**Abstract**

**Objectives:** The diversity of gut microbiota contributes to the pathogenesis of inflammatory bowel disease (IBD) and the associated tumorigenesis. The expression of thrombin receptor proteinase-activated receptor 1 (PAR1) has been reported to be upregulated in inflammatory bowel disease and contributes to inflammation. However, the effect of PAR1 on gut microbiota diversity is unknown. The present study investigated the effects of the PAR1 antagonist atopaxar on the gut microbiota diversity in the experimental model of colitis-associated tumorigenesis.

**Main findings:** A colitis-associated tumorigenesis mouse model was prepared by azoxymethane (AOM)/dextran sulfate sodium (DSS) treatment. The atopaxar treatment was started after completing the AOM/DSS treatment. AOM/DSS model exhibited weight loss and diarrhea, tumor development, inflammation, fibrosis, production of inflammatory cytokines (IL6, TNF-α, INF-γ, TGF-β1 mRNA), and increased proliferative activity of epithelial cells in the colon. Atopaxar treatment significantly mitigated all these pathological changes. The metagenome analysis of stool sample revealed a significant difference in β-diversity, but not α-diversity, between AOM/DSS and control mice. Atopaxar treatment induced a significant difference in the β-diversity, but not α-diversity, by affecting the abundance of specific bacteria in AOM/DSS mice.

**Conclusion:** $PAR_1$ antagonist mitigated the inflammation and inhibited the associated tumorigenesis in the experimental colitis. The effect of atopaxar on gut microbiota diversity may contribute, at least in part, to the therapeutic effect of $PAR_1$ antagonist.

### [2P-080]

### Preferential Neurogenesis of Nitrergic Neurons in the Myenteric Plexus of the DSS-induced Colitis Mouse Colon Causes Colonic Dysmotility in Colitis

*Makoto Kadowaki[1], Kana Miyata[1], Takeshi Yamamoto[1], Shusaku Hayashi[1] ([1]*Laboratory of Gastrointestinal Pathophysiology, University of Toyama*)

The enteric nervous system (ENS) continues to undergo various disturbances throughout life, which causes neurodegeneration in the ENS. Therefore, it is assumed that neurogenesis is induced to maintain the neuronal network in the adult ENS. However, these underlying mechanisms are largely unknown. We aimed to investigate adult neurogenesis in the DSS-induced colitis mouse colon. Methods: male C57BL/6N mice (12-week-old) were administered 2% DSS in their drinking water for 8 days. After DSS treatment, cross-sections and longitudinal muscle and myenteric plexus preparations from the colon were used for immunohistochemistry. The segments of colons were mounted in organ baths and then exposed to the neuroactivator veratridine. Results: in the motility study, veratridine-induced colonic contractions were significantly suppressed in DSS-induced colitis mice compared to normal mice. Immunohistochemical analyses revealed that the proportion of nitrergic neurons per ganglion was significantly increased in the colons of DSS-induced colitis mice compared to normal mice. Furthermore, the proportion of Sox2 (newly born neuron marker)-positive neurons per ganglion was not significantly different between normal mice and DSS-induced colitis mice, whereas the proportion of Sox2-positive nitrergic neurons to Sox2-positive neurons per ganglion was significantly increased in the colons of DSS-induced colitis mice compared to normal mice. In addition, NOS inhibitor significantly enhanced veratridine-induced colonic contractions in DSS-induced colitis mice compared with normal mice. Conclusions: these findings suggested that colitis caused an imbalance in the enteric neural circuit composed of excitatory neurons and inhibitory neurons in the myenteric plexus of the colon, which resulted in colonic dysmotility.

### Poster

### [2P]
### Oral physiology

March 29, 13:00 - 14:20, Poster Room

### [2P-081]

**Role of BMP2 expressed by excretory duct ligation of mouse parotid gland**

*Megumi Yokoyama[1], Osamu Katsumata-Kato[1], Miyuki Toda[1], Junko Fujita-Yoshigaki[1]
([1]*Department of Physiology, Nihon University School of Dentistry at Matsudo*)

Although BMP2 (bone morphogenetic protein 2) is known to induce differentiation of bone tissue and cartilage. It has been reported that pancreatic duct ligation increases BMP2 expression. The structure of the pancreas as an exocrine gland shows a similar histological structure to that of the parotid gland. Therefore, we predicted that a similar phenomenon would occur with duct ligation in the parotid gland, and searched for the expression of BMP2 and investigated the role of BMP2.Using the parotid gland of a mouse whose unilateral parotid gland excretory duct was ligated with a microclip for 7 days, the expression level of BMP2 was investigated by real-time PCR, and immunohistochemical staining with anti-Ki67 antibody were performed. The control was a sham-operated parotid gland. Both BMP2 gene expression level and Ki67-positive cell rate were significantly increased in parotid glands performed duct ligation. Next, to examine the role of BMP2, we harvested the mouse parotid gland and treated it with collagenase and hyaluronidase to prepare primary cultured parotid gland cells. The cells were cultured for 48 hours in the presence of BMP2 and in the presence of BMP2 and BMP2 inhibitor, LDN193189. The cell proliferation ability of primary cultured cells supplemented with BMP2 was significantly increased compared to the cells not supplemented with BMP2. Furthermore, in order to confirm that the primary cultured cells maintained epithelial function, the expression levels of E-cadherin and Vimentin were confirmed by Western blotting, and it was found that the expression of E-cadherin was maintained. Vimentin was not detected. To validated the BMP2 signal pathway, phospho-smad1 was detected in the presence of BMP2, but not by addition of inhibitor.From the above, the expression level of BMP2 increases in the parotid gland due to duct ligation, and its role is to increase cell proliferation ability, suggesting that it is involved in the regeneration and recovery of the salivary gland.

### [2P-082]

**Intracellular cAMP level increase induced $Ca^{2+}$ influx in odontoblasts and inhibited mineralization**

*Maki Kimura[1], Takehito Ouchi[1], Ryuya Kurashima[1], Hidetaka Kuroda[1,2], Masayuki Ando[1], Kyosuke Kono[1], Sachie Nomura[1], Yoshiyuki Shibukawa[1] ([1]*Department of Physiology, Tokyo Dental College*, [2]*Department of Dental Anesthesiology, Kanagawa Dental University*)

Odontoblasts are capable to detect various stimuli applied to the dentin surface. The stimuli elicit $Ca^{2+}$ influx from extracellular medium through mechanosensitive ion channels in odontoblasts. The intracellular $Ca^{2+}$ signaling is essential for reactionary dentin formation and/or generating dentinal pain. Previously, we revealed the functional coupling showing that intracellular cAMP level increase by activation of cannabinoid 1 receptors induced $Ca^{2+}$ influx through transient receptor potential vanilloid subfamily member 1 in odontoblasts. These findings suggest that intracellular cAMP mediates intracellular $Ca^{2+}$ signaling in odontoblasts. In this study, we examined the crosstalk between intracellular $Ca^{2+}$ and cAMP signaling in human odontoblasts and the effect of intracellular cAMP level on mineralization. Intracellular cAMP level and intracellular free $Ca^{2+}$ concentration ($[Ca^{2+}]_i$) were measured with mNeonGreen-based cAMP sensor and fura 2-AM in human odontoblast (HOB) cells. For mineralization assay, HOB cells were cultured for 21 days in mineralization medium. We then performed alizarin red and von Kossa staining. In the presence of extracellular $Ca^{2+}$, application of an adenylyl cyclase (AC) activator, forskolin (FSK) or a beta-2 adrenergic ($\beta_2$) receptor agonist (isoproterenol) increased intracellular cAMP level and $[Ca^{2+}]_i$ in HOB cells. The $[Ca^{2+}]_i$ increase could not be observed by removing extracellular $Ca^{2+}$. FSK-evoked $[Ca^{2+}]_i$ increase was inhibited by application of a protein kinase A (PKA) inhibitor in HOB cells. The $[Ca^{2+}]_i$ increase was also inhibited by application of a non-selective $Ca^{2+}$ channel blockers, $Zn^{2+}$ or $Gd^{3+}$, or non-specific TRP channel antagonist, 2APB. However, application of blockers of L- or T-type voltage gated $Ca^{2+}$ channels (VGCC) had no effect on the $[Ca^{2+}]_i$ increase. In mineralization assay, addition of FSK to mineralization medium inhibited mineralization level by HOB cells. We showed that activation of AC and $\beta_2$ receptors induced intracellular cAMP level increase and then cAMP-mediated PKA activation elicited $Ca^{2+}$ influx from extracellular medium in odontoblasts. $\beta_2$ receptor activation increased intracellular cAMP level by AC stimulation in odontoblasts. The $Ca^{2+}$ influx evoked by increase in intracellular cAMP level was occurred by $Zn^{2+}$-, $Gd^{3+}$-, and 2APB-sensitive $Ca^{2+}$ channel activities, except L- and T-type VGCC. In addition, we suggest that AC activation suppressed dentin formation and mineralization.

### [2P-083]

**The functional analysis of GPRC5C as a saccharide sensor**

*Shingo Takai[1], Yuko Kawabata[1], Keisuke Sanematsu[1,2,3], Syusuke Iwata[4], Fuminori Kawabata[5], Takashi Kanematsu[6], Eijiro Jimi[2,7], Noriatsu Shigemura[1,3] ([1]*Sect. of Oral Neurosci., Grad. Sch. Dental Sci., Kyushu Univ.*, [2]*Oral Health/Brain Health/Total Health Res. Center, Kyushu Uni.*, [3]*R and D Center for Five-Sense Devices Taste and Odor Sensing, Kyushu Univ.*, [4]*Dept. of Oral Physiol., Asahi Univ. Sch. of Dentistry*, [5]*Physiol. of Domestic Anim., Faculty of Agric. and Life Sci., Hirosaki Univ.*, [6]*Dept. of Cell Biol. Aging Sci. and Pharmacol. Div. of Oral Biological Sci. Faculty of Dental Sci. Kyushu Univ.*, [7]*Lab. of Molecular and Cellular Biochem. Grad. Sch. of Dental Sci. Kyushu Univ.*)

GPRC5 family belongs to the class C G protein-coupled receptors (GPCRs), the same as taste receptor type 1 member (TAS1Rs). TAS1Rs are involved in the sensing of nutrients in the various organs, including the peripheral taste system and gastrointestinal tract, however expression and function of GPRC5s have not been well studied, and its specific ligands are still largely unclear. In this study, we focused GPRC5C and explored the expression of GPRC5C, one of the GPRC5 family genes, and found characteristic expression patterns in a subset of intestinal cells, pancreatic α-cells and type II taste cells. Next, we cloned mouse *Gprc5c* and performed an intracellular calcium concentration assay in transgenic HEK293 cells together with a chimeric G protein (*Gα16-gust44*). GPRC5C + Gα16-gust44 expressing HEK293 cells showed robust increases in intracellular calcium concentration in response to monosaccharide (glucose, fructose and galactose), disaccharide (sucrose and maltose) and sugar alcohol (sorbitol) stimulation, whereas a sweet-tasting amino acid (D-phenylalanine) and an artificial sweetener (SC-45647) could not generate responses. The response to glucose was increased in a concentration-dependent manner and its EC50 was 75.37 mM in this experimental setting. Notably, there were time lags between stimulation and the intracellular calcium increase for all natural sugars and the sugar alcohol we tested. The duration from the end of 100 mM glucose stimulation to the beginning of the response averaged 46.6 seconds. Even a long glucose application (3 min) produced no response during the stimulation itself. These results suggest the GPRC5C may provide “off” responses for drops in sugar or sugar alcohol concentrations, representing the first demonstration of this novel function in nutrient detection.

### [2P-084]

### Development of newly evaluation system for hardness and springiness perceptions in rats

*Takutoshi Wakao[1,2], Chihiro Nakatomi[2], Chia-Chien Hsu[2], Tatuo Kawamoto[1], Kentaro Ono[2] (*[1]Division of Orofacial Functions and Orthodontics, Department of Health Promotion, Kyushu Dental University, [2]Division of Physiology, Department of Health promotion, Kyushu Dental University*)

During mastication, hardness and springiness sensations play important roles for formation of food bolus in the mouth. However, due to the lack of established animal experimental system the mechanism underlying hardness and springiness perceptions remain unclear. Therefore, we established a newly animal experimental system for evaluating hardness and springiness perceptions. Test gel materials were prepared with agar (carbohydrate-based) at 1-3% and gelatin (protein-based) at 4-16%. Physical properties of the materials were measured by stress-rupture test. In human sensory test, subjects were introduced to chew the test materials and answered intensities of hardness and springiness. In animal experiments, we used male Wistar rats and conducted conditioned aversion test by using the administration of 0.15M LiCl (unconditioned stimulus: US). In human sensory test, the initial elastic modulus (IEM) and fracture energy (FE) were positively correlated with hardness and springiness perception, respectively. In the two-bottle test of gelatin gels at 4% and 16%, agar gel at 2.2% was used as conditioning stimulus (CS) because IEM of the agar gel was equivalent to 16% gelatin gel. After one time of CS-US training, preference to 16% gelatin gel was significantly decreased. In the two-bottle test of agar gels at 1% and 3%, gelatin gel at 8.5% was used as CS because FE of the gelatin gel was equivalent to 3% agar gel. After two times of CS-US training, preference to 3% agar gel was significantly decreased. These results suggested that rats perceive the hardness and springiness of the gels, based on IEM and FE, respectively. By using the methodology employed in this study, it becomes possible to elucidate the receptors and neural circuits involved in the perception of the hardness and springiness.

### [2P-085]

### Preventive roles of agmatine on anxiety- and hindpaw pain-like responses under persistent inflammatory conditions of the masseter muscle in male mice.

*Yuya Iwamoto[1,2], Kajita Piriyaprasath[1,3], Mana Hasegawa[1,2], Yoshito Kakihara[4,5], Noritaka Fujii[2], Kensuke Yamamura[1], Keiichiro Okamoto[1,5] (*[1]Division of Oral Physiology, Faculty of Dentistry and Graduate School of Medical and Dental Sciences, Niigata University, Niigata City, Japan., [2]Division of General Dentistry and Dental Clinical Education Unit, Faculty of Dentistry and Graduate School of Medical and Dental Sciences, Niigata University, Niigata City, Japan., [3]Department of Restorative Dentistry, Faculty of Dentistry, Naresuan University, Phitsanulok, Thailand., [4]Division of Dental Pharmacology, Faculty of Dentistry and Graduate School of Medical and Dental Sciences, Niigata University, Niigata City, Japan., [5]Sakeology Center, Niigata University, Niigata City, Japan.*)

Agmatine (AGM) is an endogenous polyamine, contained in rice-fermented foods, like Japanese Sake and Sake Lees, and could have a role in alleviating psychological illnesses. However, less attention has been paid to the AGM effect on them associated with chronic orofacial pain. This study aims to determine the inhibitory effects of AGM on anxiety- and hindpaw pain-like responses with neural responses in the brain in the masseter muscle (MM) pain model. MM pain model was developed by MM injection of Complete Freund's Adjuvant (CFA) in male mice (C57BL/6J). Anxiety-like behaviors were assessed by multiple procedures, using the Dark/Light box, elevated plus maze, social interaction, open field, and novel object recognition tests on Day +1, +3, +7, and +11 after CFA. Thermal and mechanical sensitivities in the hindpaw were also assessed to quantify pain-like behaviors outside the affected region. Immunohistochemical experiments (IHC) were conducted to assess the effects of inflammatory pain in the MM on epigenetic (acetylated histone H3) and neural responses (c-Fos and FosB) in the anterior cingulate cortex (ACC), insular cortex (IC), rostral ventromedial medulla (RVM), and trigeminal caudalis (Vc) at Day +11. AGM (30 mg/kg/day) was systemically administered daily from Day -10 to 0, which could allow us to elucidate the preventive effects of AGM.CFA mice increased anxiety-like behaviors and impaired memory functions, while hindpaw pain-like behaviors were also increased. The AGM improved those behaviors in CFA mice, but not in sham mice. IHC revealed that CFA mice showed increased acetylated histone H3-positive cells in several regions. Further, in CFA mice, FosB expressions in the ACC and IC, and c-Fos expressions in the RVM and Vc regions were affected on Day +11 compared with sham mice. These findings indicated that AGM had long-term modulatory effects on neural responses even after the AGM administration was stopped on Day 0. In conclusion, daily intakes of AGM for 10 days before the induction of MM inflammation could regulate neural functions in the brain, which can prevent anxiety and nociception in the remote areas associated with orofacial pain conditions.

### [2P-086]

### Piezo1 channel activates TRPV1/TRPA1 channels in rat odontoblast

*Ryuya Kurashima[1], Maki Kimura[1], Takehito Ouchi[1], Yoshiyuki Shibukawa[1] (*[1]Tokyo Dental College*)

As sensory receptor cells, odontoblasts play important roles in dentin formation and the mechano-sensory transduction pathway. We have previously demonstrated that direct mechanical stimulation activates the mechano/thermosensitive ion channels, transient receptor potential (TRP) channel subfamilies (TRPV1, TRPV2, TRPV4, and TRPA1) and mechanosensitive ion channel, Piezo1 channel (Piezo1), in odontoblasts. Direct mechanical stimulation-induced intracellular $Ca^{2+}$ signal was almost completely abolished in the Piezo1 gene-silenced odontoblasts compared to the cells not subjected to the silencing. Thus, Piezo1 seems to provide the most upstream intracellular signal of the mechanosensitive processes in odontoblasts. However, the signaling mechanism of the crosstalk between Piezo1 and TRP channel activities has not been clarified. This study investigated the crosstalking machinery between these channels in acutely isolated rat odontoblasts obtained from neonatal Wistar rats. We observed Piezo1, TRPV1, TRPV4, and TRPA1 immunoreactivities in the primary cultured acutely isolated rat odontoblasts. Piezo1 and TRPV4 immunoreactivities showed polarized localization, while Piezo1 and TRPV1/TRPA1 represented colocalization. In the presence of extracellular $Ca^{2+}$, 1.5 min application of a pharmacological Piezo1 activator, Yoda1, increased intracellular free $Ca^{2+}$ concentration ($[Ca^{2+}]_i$) measured by fura-2, showing transient biphasic responses composed of initial and second phases. We also observed triphasic $[Ca^{2+}]_i$ increase composed of transient biphasic followed by a steady phase in response to 10 min application of Yoda1. Application of a selective Piezo1 antagonist, Dooku1, completely inhibited Yoda1-induced triphasic $[Ca^{2+}]_i$ increases. When we applied a selective TRPV1 antagonist, A784168, we could not observe any effects on the initial phase but observed inhibitory effects on the second and steady phases of Yoda1-induced triphasic $[Ca^{2+}]_i$ increases. A selective TRPA1 antagonist, HC030031, did not inhibit the initial and steady phases but inhibited the second of the Yoda1-induced triphasic $[Ca^{2+}]_i$ phases. We could not observe any significant effects of a selective TRPV4 antagonist, RN1734, on the Yoda1-induced triphasic responses. We also observed transient $[Ca^{2+}]_i$ increase followed by steady $[Ca^{2+}]_i$ increase in response to 2 min application of direct mechanical stimulation to odontoblasts. Direct mechanical stimulation-induced $[Ca^{2+}]_i$ increases were completely inhibited by treatments with non-selective mechanosensitive ion channel inhibitors, $GdCl_3$, A784168, and HC030031. The results suggest structural and functional crosstalks between Piezo1 and TRPV1/TRPA1 in rat odontoblasts.

### [2P-087]

### Elucidation of relationship between EP4 receptor and IL-6 and IL-24 in oral cancer cells

*Wakana Fukae[1], Soichiro Ishikawa[1,2], Masanari Umemura[1], Rina Nakakaji[1,2], Eriko Yamashita[1,2], Yoshihiro Ishikawa[1] (*[1]Cardiovascular Research Institute (CVRI), Yokohama City University, [2]Department of Oral and Maxillofacial Surgery, Yokohama City University*)

### Poster

### [2P]
### Blood, Lymph, Immunity

March 29, 13:00 - 14:20, Poster Room

### [2P-088]

**Flavacitropone A from *Glycosmis citrifolia* (Rutaceae) induces autophagic cell death via p53 pathways in human pre-B cell leukemia cells**

*Takuya Matsui[1] ([1]*Department of Physiology, School of Medicine, Aichi medical university*)

Precursor B-cell acute lymphoblastic leukemia is the most prevalent pediatric cancer and its pathogenic mechanism is not known. Although conventional chemotherapy can now cure this disease, various problems remain, including adverse effects of anti-tumor drugs, bone marrow and central nervous system relapses, and decreased quality of life. Thus, we are exploring seed compounds from natural products. We have reported that flavacitropone A, a homoacridone-flavanone dimer isolated from *Glycosmis citrifolia* (Willd.) Lindl., induces cell death in a human pre-B cell leukemia cell line (NALM6). Here, we aimed to elucidate the detailed pharmacological mechanism of flavacitropone A in NALM6. Microarray analysis revealed that flavacitropone A dramatically upregulated DDIT3 and ATF3 genes, and functional enrichment analysis by GSEA software predicted that this biological activity of flavacitropone A was involved in MYC, MTOR and FOXO3 signaling. DDIT3, ATF3 and p21 mRNA was upregulated and cMyc mRNA was downregulated, consistent with the microarray results. Immunoblotting was performed to examine the protein expression of signaling molecules in NALM6 at 12 and 24 h after flavacitropone A treatment: CHOP, ATF3, phosphorylated p53, p21 and LC3A/B were upregulated, while phosphorylated Akt, phosphorylated FoxO1/3 and cMyc were downregulated. Akt, cMyc and p53 are known to be involved in multiple functions in tumor cells, including cell proliferation, cell survival and apoptosis. The changes in CHOP, LC3A/B and phosphorylated FoxO1/3 suggest that flavacitropone A induced autophagic cell death. We investigated the relationship between flavacitropone A-induced cell death and these molecules using inhibitors (p53 inhibitor: pifithrin b; Akt inhibitor: MK2206; cMyc inhibitor: 10058-F4). Treatment of NALM6 with flavacitropone A plus pifithrin b dramatically decreased annexin V-positive cells and downregulated DDIT3 mRNA and p21 mRNA compared with flavacitropone A alone. By contrast, cells treated with flavacitropone A plus MK2206 or 10058-F4 showed slightly increased annexin V-positive cells compared with flavacitropone A alone. These results show that cell death and G0/G1 arrest of flavacitropone A-treated cells are closely linked to p53 expression. Thus, flavacitropone A appears to induce autophagic cell death via upregulation of p53 expression in NALM6.

### [2P-089]

**Src promotes IFNγ-induced PD-L1 expression through PYK2 activation**

*Chihiro Hayashi[1], Yuto Mizuno[1,2], Masanari Umemura[1], Fumina Suzuki[1], Mio Mochiduki[1], Akane Nagasako[1], Kagemichi Nagao[1,4], Rina Nakakaji[1,3], Soichiro Ishikawa[1,3], Wakana Fukae[1], Yoshihiro Ishikawa[1] ([1]*Cardiovascular Research Institute (CVRI), Yokohama City University Graduate School of Medicine,* [2]*Department of Environmental Immuno-Dermatology, Yokohama City University Graduate School of Medicine,* [3]*Department of Oral and Maxillofacial Surgery, Yokohama City University Graduate School of Medicine,* [4]*Department of Neurosurgery, Yokohama City University Graduate School of Medicine*)

### Poster

### [2P]
### Circulation

March 29, 13:00 - 14:20, Poster Room

### [2P-090]

### Alterations in ketone body metabolism and its cardioprotective effects in the early stage of diabetic cardiomyopathy

*Yoshinori Mikami[1], Daisuke Ohshima[1], Taichiro Tomida[1], Yuto Tei[1], Satomi Adachi-Akahane[1] ([1]*Department of Physiology, Faculty of Medicine, Toho University*)

Diabetic cardiomyopathy (DMCM) is defined as diabetic myocardial dysfunction without coronary artery disease. It is characterized in its early stage by left ventricular (LV) diastolic dysfunction and later by systolic dysfunction. However, the mechanisms that induce diastolic dysfunction in the early stage of DMCM have not been understood. We aimed to elucidate the mechanism that precedes diastolic dysfunction caused by metabolic dysregulation. Streptozotocin (STZ) was administered to mice to generate a model of type 1 diabetes mellitus. LV diastolic function, but not systolic function, was significantly impaired 4 weeks after STZ administration (STZ-4W). In the isolated ventricular myocytes from the STZ-4W mice, the $Ca^{2+}$ transient decay rate was slower than in control mice. These results suggest that the capacity for $Ca^{2+}$ uptake into the sarcoplasmic reticulum via sarcoplasmic/endoplasmic reticulum $Ca^{2+}$-ATPase (SERCA) is downregulated. Thus, we examined the hypothesis that diastolic dysfunction is caused by a reduction in SERCA activity associated with a decrease in energy substrates. Metabolomic analysis of ventricles by CE-TOFMS revealed that ATP concentration was not decreased but rather slightly increased in the STZ-4W group. Interestingly, the concentration of β-hydroxybutyrate (βOHB) was markedly higher in the ventricles of the STZ-4W group than those of the control group. βOHB is a ketone body used as an energy source during starvation. Blood levels of βOHB were elevated in the STZ-4W group compared to the control group, suggesting that they were taken up into the myocyte. To investigate the possibility of impaired ketone body metabolism, we examined the expression of metabolic enzymes by RNA-seq and qRT-PCR. In the ventricles of the STZ-4W group, the expression levels of 3-hydroxybutyrate dehydrogenase 1 (BDH1), which uses βOHB as a substrate, and the rate-limiting enzyme succinyl-CoA:3-ketoacid CoA transferase (SCOT) were significantly lower than those of the control group, suggesting defective βOHB metabolism. The expression levels of several antioxidant genes were elevated, indicating that the accumulated βOHB exerts cardioprotective effects through histone deacetylase inhibition. These results suggest that βOHB accumulated in ventricular myocytes maintains ATP levels and plays a cardioprotective role in the early stage of DMCM. (COI : NO)

### [2P-091]

### Creation of TAC model rats according to stenosis diameter--Changes in cardiac function according to stenosis intensity--

*Tkakuya Akashi[1], Mina Matumoto[1], Yoshizo Fukuda[1], Hironobu Ikeda[1] ([1]*nissei bailis*)

Purpose: Using a rat cardiac hypertrophy model, the effect of the stenosis strength on the cardiac function was examined by measuring the changes of cardiac function due to stenosis strength up to 24 weeks after stenosis. Methods: Transverse aortic constriction (TAC) was performed on 46 male Slc:Wistar rats. The rats were divided into four groups according to the difference in stenosis diameter (group 1: 0.8 mm, group 2: 0.6 mm, group 3: 0.55 mm, group 4: 0.5 mm). Changes in cardiac function and survival rate were evaluated 2, 4, 8, 12, 16, 20 and 24 weeks after stenosis. For the evaluation of cardiac function, the values of each test item obtained by a general-purpose ultrasonic echo diagnostic device were used. Results: The left ventricular Diastolic anterior ventricular wall thickness (LVAWd) was 2.3 ± 0.1 mm in group 1, 2.4 ± 0.1 mm in group 2, 2.8 ± 0.1 mm in group 3, 2.6 ± 0.2 in group4 at increased over time in until eight weeks after stenosis, which remained constant until 24 weeks thereafter. The left ventricular end-diastolic diameter (LVIDd) and left ventricular end-systolic diameter (LVIDs) remained constant in group 1, but a tendency toward dilatation was observed in groups 2, 3, and 4. The left ventricular ejection fraction (EF) was 96.6 ± 1.2 % in group 1, 98.1 ± 0.5 % in group 2, 94.5 ± 2.4 % in group 3, and 94.3 ± 1.9 % in group 4 at 2 weeks after stenosis, but it remained constant until 24 weeks after stenosis. Survival rate remained constant in group 1 after acute phase death. Groups 2, 3 and 4 decreased over time. Conclusion: In this study, we investigated changes in cardiac function depending on stenosis intensity up to 24 weeks after stenosis. In all groups, myocardial hypertrophy reached its maximum 8 weeks after stenosis. Furthermore, EF remained within the normal range until the end of the study. On the other hand, changes in cardiac lumen diameter expansion were shown to differ depending on stenosis strength.

### [2P-092]

### Blood dilution by a routine intravenous infusion caused a unique increase in mean corpuscular volume (MCV) during and after the orthopedic fracture surgery

*Katsumasa Kawahara[1,2,3], Mitsufumi Nakawaki[1], Yukiko Yasuoka[3], Takao Shimohama[3], Mariko Nishikitani[4], Shoko Matsuda[5], Hiroshi Nonoguchi[6], Daisuke Kobayashi[2], Akihiro Hazama[2] ([1]*Zama General Hospital,* [2]*Fukushima Medical University,* [3]*Kitasato University,* [4]*Kyushu University Hospital,* [5]*Health Science Co., Ltd.,* [6]*Kitasato University Medical Center*)

At the 100th meeting of JPS in Kyoto (2023), we showed that a relationship between human erythrocyte count (RBCC) and mean corpuscular volume (MCV) was negative in the **pre**operative orthopedic patients, and that in a subgroup of the patients with the higher counts of **platelet** (PLT) after the surgery, their slopes remained nearly unchanged for 2 weeks. Interestingly, similar negative (inverse) relationships were observed beyond the species barrier of animals on ground (Hawkey CM et al., 1991). In another point of view, severe training of world-class endurance athletes caused MCV-increase as well as hemodilution by expanding plasma volume (Smith JA et al.,1999). Thus, we hypothesized that MCV (erythrocyte size) might be regulated and physiologically adjusted for optimization of peripheral circulation at the normal condition and upon blood dilution by intravenous infusion of isotonic fluid during and after the surgery, especially in the orthopedic *fracture* surgery patients. **Materials & Methods**: We analyzed the electric medical record test results of 2021-22 for the orthopedic patients with traumatic injury in Zama General Hospital of JMA, according to the guideline for patients' rights. The subjects were sub-grouped into (a) *fracture* patients with open surgery and/or arthroplasty surgery; **Fx** (n=7) and (b) non-fracture patients; control (n=7). **Results**: (1) There were no significant differences between the two groups in ages and **pre**operative major blood data, such as plasma protein, total bilirubin, RBCC, hemoglobin concentration (Hb), MCV, MCHC, PLT count, and so on. (2) In the **post**operative period of 2 weeks, PLT counts (mean values) of both **Fx**-group and control significantly (p=0.00001 and p=0.035, respectively) increased from 26.9 (**pre**operative) to 44.5 (peak) and from 24.8 to 28.7. (3) Through the **peri**operative period, the values of red blood cell distribution width (RDW) in both groups were almost unchanged and within the normal ranges. (4) Surprisingly, the negative slope and large negative **r** (correlation coefficient) were well maintained in **Fx** group (n = 7 of Days1~7 and 5 of Day14): **-0.063** and -0.760 (**pre**operative), **-0.061** and -0.904 (Day1), **-0.036** and -0.864 (Day3), **-0.052** and -0.917 (Day7), **-0.079** and -0.908 (Day14), while they were not in control (n=7). (5) Finally, MCV was slightly, but significantly (p = 0.006~0.025) increased through the **post**operative periods in **Fx** group (values of Days1, 3, 7, 14 vs. preoperative value: 1.025, 1.021, 1.021, 1.041, in sequence), but not in control. **Conclusion and Perspective**: In the orthopedic *fracture* surgery patients, blood dilution-induced increase in MCV may be a key to optimize a peripheral blood flow and prevent unexpected blood clot formation.

### [2P-093]

### The dominant-negative effect of Nav 1.5 variants does not play a crucial role in determining phenotype severity.

*Ayami Tano[1], Koichi Kato[2], Hideyuki Jinzai[2], Daisuke Fukumoto[2], Toru Jubo[3], Seiko Ohno[4], Yoshihisa Nakagawa[2], Minoru Horie[2] ([1]*Department of Neurology, Shiga University of Medical Science,* [2]*Department of Cardiovascular Medicine, Shiga University of Medical Science,* [3]*Department of Cardiology and Geriatrics, Kochi Medical School, Kochi University,* [4]*Department of Bioscience and Genetics, National Cerebral and Cardiovascular Center*)

**Objectives:** Recent studies have demonstrated that some loss of function (LOF) -SCN5A variants can exert a dominant-negative (DN) effect through channel dimerization, though this newly emerging concept is still controversial. Additionally, the association between DN effect and phenotype severity has not been well understood. In this study, we identified a proband with compound heterozygous SCN5A variants, p.G833R and p.T1396P, in a family with bradyarrhythmia. Through the clinical and *in vitro* findings, we studied 1) the electrophysiological consequences led by the identified variants, 2) the existence of the channel-dimerization and DN-effect, and 3) the association of the DN-effect and phenotype severity.
**Methods:** We performed functional analysis of mutant Nav1.5s in HEK cells by using a whole-cell patch-clamp method. The DN-effect was assessed by co-expressing WT and mutants in each heterozygous combinations, with or without difopein, a peptide cutting 14-3-3 interaction between two sodium channels. Cell surface expression of mutated channels was measured by flowcytometry. We also conducted co-immunoprecipitation (CO-IP) of HA- or FLAG- tagged variants in the same condition with patch-clamp to assess the channel connections.
**Results:** HEK cells expressing T1396P only did not show any measurable sodium current, while those with G833R had comparative current with that of WT channel. Both variants were not different in cell-surface expression in flowcytometry. Cells expressing WT/T1396P showed significant reduction and delay of inactivation decay, which was normalized by co-expression with difopein. The results implied that T1396P manifested DN-effect via channel dimerization, which could be canceled by difopein. Interestingly, heterozygote T1396P/G833R did not demonstrate DN-effect by the LOF-T1396P variant. Notably, the proband, heterozygote T1396P/G833R showed relatively severe bradycardia than her mother carrying only T1396P, suggesting that DN-cancelation by G833R did not moderate the phenotype severity. CO-IP study proved a channel-channel connection in all conditions regardless of DN-effect.
**Conclusion:** Our electrophysiological study supported the concept of Nav1.5-dimerization and consequent DN-effect by LOF-SCN5A variants although phenotype severity was not simply explainable by these mechanisms. Both difopein and the G833R variant canceled the DN-effect by the LOF-T1396P variant. However, a firm connection between channels remained regardless of the absence of DN-effect. Further studies are necessary to elucidate mechanisms underlying unknown interaction between channels, which possibly associate with the phenotype characteristics.

### [2P-094]

### High glucose induced irregular rhythm of mouse sinoatrial node

*Yukari Takeda[1,2], Satoshi Matsuoka[1,2] ([1]*Department of Integrative and Systems Physiology, Faculty of Medical Sciences, University of Fukui.,* [2]*Life Science Innovation Center, University of Fukui.*)

Higher prevalence of arrhythmia, including sinus node disfunction (SND), has been reported in patients with diabetes mellitus. Although diabetes mellitus is a complex multifactorial disease, diabetic hyperglycemia has been indicated as a key factor to induce arrhythmias. It has been suggested that enhanced spontaneous sarcoplasmic reticulum (SR) $Ca^{2+}$ release contributes to increase in the occurrence of premature ventricular complex (PVC) in Langendorff-perfused rat heart during acute high glucose challenge. However, the causal association between SND and hyperglycemia has not been reported. Since local calcium release (LCR) from SR in sinoatrial nodal cells (SNC)s contributes to automaticity, we investigated the effects of high glucose (25 mM) on SA node function. Electrocardiogram recording of Langendorff-perfused mouse heart demonstrated increased variability of R-R interval due to irregular sinus rhythm along with PVC during high glucose perfusion. In isolated SNCs, high glucose increased the variability of contraction interval. The amplitude of LCR and the occurrence of early LCR were increased during high glucose challenge. ROS levels were elevated in SNCs under the high glucose condition. Our findings suggest that acute hyperglycemic condition induces sinoatrial node dysfunction, which is associated with increased ROS levels and impaired SR $Ca^{2+}$ handling.

### [2P-095]

### Reconstructing heart functions on a microfluidic chip

*Mengxue Wang[1], Yun Liu[1], Rumaisa Kamran[1], Xiaoxia Han[1,2], Keiji Naruse[1], Ken Takahashi[1] ([1]*Okayama University,* [2]*Harbin Medical University*)

We present a bioengineered organ model designed to recapitulate the vital function of the human heart. This innovative system incorporates cardiomyocytes derived from human induced pluripotent stem cells (iPS cells), fibroblasts, and vascular endothelial cells cultured on a two-channel microfluidic chip. Notably, the endothelial cell layer, influenced by the culture medium flow, aligns itself in the direction of the medium flow, closely resembling the behavior of blood vessels in vivo. Concurrently, the medium flow promotes the formation of cell-cell junctions, effectively reducing vascular permeability. Under conditions simulating high blood pressure, such as pressure and vascular stretching observed during hypertension, the endothelial cell layer responds by releasing nitric oxide. Moreover, the endothelial cell layer within the "vascular" channel substantially facilitates the differentiation of iPS cells into cardiomyocytes in the neighboring "cardiac" channel. This co-culture not only promotes the expression of myocardial-specific markers but also facilitates sarcomere maturation, ultimately leading to increased myocardial contractility. Remarkably, this cardiac tissue exhibits a heightened heart rate in response to the administration of noradrenaline. Additionally, the reconstituted heart tissue successfully reproduces arrhythmia-like asynchronous myocardial excitation after the introduction of a calcium channel blocker. This "heart-on-a-chip" authentically replicates the structure and function of the heart, utilizing human cells. It offers a promising avenue to extend the capabilities of cell culture models while reducing the need for experimental animals in pharmacology/toxicology tests and disease model development.

### [2P-096]

### Relationship between cerebral hypercapnic vasodilatation and dynamic cerebral autoregulation

*Kei Ishii[1], Hidehiko Komine[1] ([1]*AIST*)

Decreased cerebral blood flow (CBF) during hypotension may lead to an increase in partial pressure of $CO_2$ ($PCO_2$) in the brain, which may cause vasodilation throughout cerebral vasculature. The degree of hypercapnic vasodilatation would differ between each brain region. We hypothesized that in certain brain regions, cerebral hypercapnic vasodilatation would assist dynamic cerebral autoregulation during hypotension. CBF was measured in the motor cortex (MC) and thalamus using laser-Doppler flowmetry in anesthetized rats. Dynamic cerebral autoregulation was examined during ventricular pacing for 30 s at 550–800 beats/min. Cerebrovascular response time and reactivity to 5% $CO_2$ exposure for 70 s were also examined. Ventricular pacing immediately decreased arterial blood pressure (AP) with no or little changes in in central venous pressure and intracranial pressure. The rapid AP reduction accompanied the decrease in CBF which was greater in the MC than that of the thalamus ($P < 0.05$). The differential CBF responses between the MC and thalamus were observed in the case of moderate hypotension (-34 mmHg $\leq \Delta MAP \leq$ -15 mmHg), but not severe hypotension (-54 mmHg $\leq \Delta MAP \leq$ -35 mmHg). Cerebrovascular response time and reactivity to $CO_2$ exposure also differed between the brain regions. The cerebrovascular response time and reactivity to $CO_2$ seemed to be correlated with the dynamic cerebrovascular response to moderate and severe hypotension only in the thalamus. These results suggest that dynamic cerebral autoregulation to tachyarrhythmia–induced hypotension may be caused at least partly by the $CO_2$-related vasodilatation in the thalamus.

### [2P-097]

### Dynamical Mechanisms of Sinoatrial Node Pacemaking: Roles of membrane and $Ca^{2+}$ clocks determined by bifurcation analyses of a mathematical model

*Yasutaka Kurata[1], Kunichika Tsumoto[1], Yuhichi Kuda[1], Mamoru Tanida[1] ([1]*Department of Physiology II, Kanazawa Medical University*)

To provide insights into the roles of membrane and $Ca^{2+}$ clocks in sinoatrial node (SAN) pacemaking, we theoretically investigated the roles of hyperpolarization-activated cation channel current ($I_f$) and sarcoplasmic reticulum (SR) $Ca^{2+}$ handling in SAN pacemaking. Bifurcation analyses as well as numerical simulations were performed using a mathematical model for rabbit SAN cells developed by Severi et al (J Physiol 595, 2012): stabilities of equilibrium points (EPs) and limit cycles (LCs) and bifurcation points were determined as functions of the maximum ion channel conductance and other parameters. Pacemaker activity of the Severi model was transiently abolished by an abrupt reduction of the maximum $I_f$ conductance ($g_f$) to 1% of the control value; pacemaker activity resumed with gradual decreases in intracellular $Na^+$ concentrations ($[Na^+]_i$). In contrast, gradual reductions in $g_f$ did not lead to cessation of pacemaker activity, which was due to gradual decreases in $[Na^+]_i$ during $g_f$ reductions. Stabilities of EPs and LCs were not significantly affected by modulating $I_f$ or by enhancing SR $Ca^{2+}$ uptake/release. Robustness against hyperpolarizing loads (ACh application) was lower in the $I_f$-reduced system than in the normal system; however, incorporating background $Na^+$ current ($I_{bNa}$) enhanced robustness against hyperpolarizing loads of the $I_f$-reduced system. SR $Ca^{2+}$ cycling ($Ca^{2+}$ uptake rate) did not significantly affect stability or robustness against hyperpolarizing loads of the model cells. Spontaneous intracellular $Ca^{2+}$ oscillations around unstable EPs did not occur under voltage-clamped conditions in the model cell. Simulated behaviors of sarcolemmal ionic currents during spontaneous pacemaking and the slow-fast decomposition analysis revealed that phase 4 depolarization is driven chiefly by slow activation of the delayed-rectifier $K^+$ channel current with additional contribution of $I_f$ activation. We conclude that neither $I_f$ as the membrane clock nor the SR $Ca^{2+}$ clock is essential for SAN pacemaking. SAN cell stability and pacemaking largely depend on the L-type $Ca^{2+}$ channel current.

### [2P-098]

### Effect of prolonged sitting on dynamic cerebral autoregulation

*Shotaro Saito[1], Marino Karaki[1], Narumi Kunimatsu[1], Hayato Tsukamoto[2], Shigehiko Ogoh[1] ([1]*Toyo University,* [2]*Waseda University*)

Prolonged sitting is known to increase the risk of cerebrovascular disease, independent of physical inactivity. However, the exact physiological mechanism behind how prolonged sitting increases the risk of cerebral disease remains unclear, and effective countermeasures have yet to be established. Since prolonged sitting has been linked to gradual decreases in cerebral blood flow (CBF) and induces cognitive dysfunction, we hypothesized that prolonged sitting also leads to attenuation in cerebrovascular regulation. To test this hypothesis, we examined the effect of 4 hours of continuous sitting on dynamic cerebral autoregulation (dCA) of the anterior and posterior circulation, which is a crucial component of CBF regulation, in healthy young adults. Thirteen young healthy participants were instructed to remain seated for 4 hours without moving their lower limbs. Mean arterial pressure (MAP), and mean blood velocities of the middle and posterior cerebral arteries (MCA Vm and PCA Vm, respectively) were measured before and after 4 hours of continuous sitting. dCA of the anterior and posterior circulation was evaluated using transfer function analysis (TFA) with MAP and either MCA Vm (anterior circulation) or PCA Vm (posterior circulation). After 4 hours of sitting, MCA Vm exhibited a significant decrease ($P = 0.011$), while MAP and PCA Vm remained unchanged compared to the baseline (MAP and PCA Vm; $P = 0.376$ and $P = 0.914$). In addition, the evaluation of dCA in the anterior and posterior circulation, as indicated by TFA low-frequency phase and normalized gain, revealed no significant change following a 4-hour sitting period (anterior circulation: $P = 0.652$ and $P = 0.290$, posterior circulation: $P = 0.481$ and $P = 0.751$). Despite the decrease in anterior CBF due to prolonged sitting, it is notable that dCA remained consistent in both the anterior and posterior circulation. This finding suggested that the decrease in CBF due to prolonged sitting may not be attributed to alterations in dCA.

### [2P-099]

### Understanding the role of epidermal growth factor (EGF) pathway ligands in exacerbation of pulmonary hypertension due to insulin resistance

*MD JUNAYED NAYEEM[1], Mark T. Waddingham[1], Hirotsugu Tsuchimochi[1], James T. Pearson[1] ([1]*Department of Cardiac Physiology, National Cerebral and Cardiovascular Center Research Institute, Suita.*)

**Introduction:** Insulin resistance (IR) is prevalent in pulmonary hypertension (PH) patients, exhibiting poorer pulmonary vascular function and increased mortality. Data from our group shows that IR exacerbates pulmonary vascular remodeling, accelerating the progression of PH in Goto-Kakizaki (GK) rats. Various ligands for the EGF pathway have been implicated in PH development. In this study, we investigated the role of EGF family ligands in the development of severe early pulmonary vascular remodeling in GK rats with PH. **Methods:** PH was induced in GK ( n=14) and Wistar (n=14) rats with Sugen 5416 (20mg/kg) and exposure to chronic hypoxia (10% $O_2$ ) for 1 week and 2 weeks. Right ventricular pressure (RVP) was measured after each time point by closed-chest right heart catheterization. After the hemodynamic measurements, heart and lung tissue were collected. The Fulton index was calculated from the ratio of RV and left ventricle plus septum weight. Expression of specific EGF ligands was determined by qRT-PCR and western blotting. **Results:** Mortality was only observed in GK rats (37.5%) at 2-week time point. Interestingly, RV systolic pressure (RVSP) did not increase in GK, over the time course of the study, whereas a progressive increase in RVSP was observed in Wistar. A higher Fulton index and increased hematocrit were observed in GK at both 1 and 2-week time points. In the ongoing study, we have found that the mRNA expression of EGF family ligands, amphiregulin, and HB-EGF, is greater in GK lung at both the 1-week (~1.3-fold) and 2-week (~3-fold) time point when compared to Wistar. HB-EGF protein level was also significantly increased in the GK lung (strain effect ; P<0.05) at both 1 and 2-week time points. A trend for greater EGF receptor protein expression in the lung was observed in normoxic GK than in the Wistar, which was depressed after hypoxia. These observations suggest that elevated EGF ligands may accelerate the progression of PH in the setting of combined IR and PH, possibly through immune cell activation and vascular smooth muscle remodeling. Currently, we are investigating the extent of pulmonary vascular remodeling at both time points. We are now also investigating the EGF pathway in the lung endothelial cells. **Conclusion** : Elevated EGF ligands in the lung may contribute to early pulmonary vascular remodeling due to combined IR and PH.

### Poster

### [2P]
### Urinary organ, Renal function, Urination

March 29, 13:00 - 14:20, Poster Room

### [2P-100]

### Effects of peritoneal dialysis fluids on AVP dynamics in humans and transgenic rats

*Hiromichi ueno[1,2], Tetsu Miyamoto[1], Mitsuhiro Yoshimura[2], Takashi Maruyama[2], Junichiro Koga[1], Masaharu Kataoka[1], Ueta Yoichi[2] (*[1]The Second Department of Internal Medicine, School of Medicine, University of Occupational and Environmental Health, [2]Department of Physiology, School of Medicine, University of Occupational and Environmental Health*)

**Background:** Although peritoneal dialysis (PD) is recommended as the first-line treatment for end-stage renal disease (ESRD), it often could not continue because of fluid retention. Arginine vasopressin (AVP), which is well known to be an antidiuretic hormone, cause fluid retention and stimulate thirst. Therefore, AVP is thought to be one of the important factors related to fluid management. Several studies have reported that an increase of AVP level in hemodialysis. On the other hand, the effect of peritoneal dialysis fluid (PDF) on AVP kinetics in humans and transgenic rats remains unclear. **Methods:** In this study, we studied 20 PD patients ranging age from 48-79 years and PD duration for 2-145 months. We examined plasma AVP level on admission. AVP synthesis and release are mainly controlled by plasma osmolality and blood volume. We have generated transgenic rats expressing an AVP-enhanced green fluorescent protein (eGFP) fusion gene. In this study, we investigated AVP-eGFP synthesis in the hypothalamus after peripheral administration of saline, Glucose-PDF or Icodextrin-PDF, using the transgenic rats.**Results:** In PD patients, a significant increase in plasma AVP level and osmolality was observed. We obtained a positive correlation between plasma AVP level and osmolality. In the glucose-PDF and icodextrin-PDF groups, eGFP fluorescent intensities in the SON and the PVN after administration were significantly increased in comparison with saline group. **Discussion:** Several studies have reported that PD solutions increase plasma osmolarity. Therefore, we presumed that PD-induced hyperosmolarity could cause an increase in AVP levels. To our knowledge, this is the first report demonstrating elevated plasma AVP levels in PD patients and upregulation of hypothalamic AVP after peritoneal dialysis fluid administration in transgenic rats. These findings provide new insights into fluid management in PD patients.

### [2P-101]

### Dopamine receptor-mediated urinary continence mechanisms in a sneeze-induced rat model

*Kota Kawase[1,2], Takuya Koie[2], Minoru Miyazato[1] (*[1]University of the Ryukyus, [2]Gifu University*)

**[Object]** Neurotransmitter mechanisms in the urinary continence reflex are complicated, and the involvement of dopaminergic urethra continence mechanisms remains unclear. Therefore, we aimed to elucidate the role of dopamine receptors in the urinary continence reflex using a sneeze-induced rat model.
**[Methods]** Ten-week-old normal female Sprague-Dawley rats (n=24) were administered cumulative doses of a nonselective dopamine agonist (apomorphine) at 0.01-1 mg/kg during sneezing with a rat whisker using a 3.5-Fr microtip-transducer urethral catheter. Then, rats were divided into four groups according to the presence or absence of dopamine $D_1$ receptor antagonist (SCH-23390: 0.1 mg/kg) and dopamine $D_2$ receptor antagonist (remoxipride: 1 mg/kg). A: apomorphine only, A+S: apomorphine + SCH-23390, A+R: apomorphine + remoxipride, A+S+R: apomorphine + SCH-23390 + remoxipride. Urethral baseline pressure (Pu) at rest and urethral reflex pressure (ΔPu) and abdominal pressure (ΔPa) during sneeze induction were measured.
**[Results]** Resting Pu was not significantly different in either group. Most of A and A+S ($D_1$ receptor antagonist) groups showed continuous urethral contractions (bursting activity) at 1 mg/kg apomorphine, but group of A+R ($D_2$ receptor antagonist) or A+S+R did not. Sneeze-induced ΔPu/ΔPa ratio was significantly lower both in groups A and A+S when apomorphine 1 mg/kg was administered (pre-drug vs. A only, p = 0.03, pre-drug vs. A+S, p = 0.02), but not in the group of A+R or A+S+R.
**[Conclusions]** Dopamine reduces urethral reflex pressure, primarily through the activation of dopamine $D_2$ receptors. It was suggested that dopamine $D_1$ receptors may contribute to maintain the urethral pressure by assisting external urethral sphincter contractile stimulation in the spinal cord. Thus, activation of $D_1$ receptors may represent a new approach in the treatment of urinary incontinence, especially in Parkinson's disease with depleted dopaminergic neurons.

### [2P-102]

### Effects of PHD inhibitor on Erythropoietin Production in the Rat Body

*Yukiko Yasuoka[1], Yuichiro Izumi[2], Tomomi Oshima[1], Katsumasa Kawahara[3], Noriko Takahashi[1], Hitoshi Nonoguchi[4] (*[1]Dept of Physiol. Kitasato Univ. School of Med., [2]Dept of Nephrol. Kumamoto Univ. Graduate School of Medical Sci., [3]Dept of Rehabilitation, Zama General Hospital, [4]Internal Med. Kitasato Univ. KMC Hospital*)

Hypoxia stimulates erythropoietin (Epo) production in the kidney by inhibiting the Prolyl Hydroxylase Domain (PHD), which then stimulates hypoxia-inducible factor (HIF) 1α/2α. PHD inhibitors mimic severe hypoxia, thereby, reducing HIF1α/2α degradation, which increases HIF1α/2α activity and Epo production. Although PHD inhibitors rapidly stimulate Epo production, the precise sites of Epo production following the administration of these drugs have not been identified. We examined the effects of the PHD inhibitor, Roxadustat (ROX), and severe hypoxia on Epo production in various tissues in rats. Methods: Male Sprague Dawley rats (170-200g) were divided into four groups: 1, control (n=6); 2, ROX-treatment (n=9); 3, control to hypoxia (n=6); and 4, severe hypoxia (n=6). The ROX-group rats were given an intraperitoneal injection of ROX (FG-4592, 50 or 100 mg/kg body weight) for 6 hours. The hypoxia-group rats were placed in 7% $O_2$ for 4 hours. After implementing each treatment, rats were injected with mixed anesthetic, and blood was taken from the heart. Organs were taken after perfusing 20 mL of PBS from the heart. Expression of Epo mRNA and protein were estimated by using Real Time PCR, western blotting and immunohistochemistry. Results: ROX (50 mg/kg) significantly increased the plasma Epo concentration from 1.2 ± 0.1 to 1072 ± 333 mIU/mL ($p < 0.001$). ROX increased Epo mRNA expression in both the kidneys and liver. However, Epo protein was detected in the kidneys but not in the liver. Epo protein was also detected in the salivary glands, spleen, epididymis and ovaries. However, both PHD inhibitor (ROX) and severe hypoxia increased the Epo protein abundance only in the kidneys. Conclusion: Epo is produced in many tissues. PHD inhibitors as well as severe hypoxia regulate Epo production only in the kidneys.

### [2P-103]

### Comparison between urinary fatty acid binding protein 1 and neutrophil gelatinase-associated lipocalin in acetaminophen-induced acute kidney injury in mice

*Tatsuya Iso[1], Rina Tanaka[2], Hiroki Matsui[2], Tomoyuki Yokoyama[2] ([1]*Gunma University of Health and Welfare, Faculty of Medical Technology and Clinical Engineering,* [2]*Gunma University Graduate School of Health Sciences*)

Acute kidney injury (AKI) is a life-threatening condition under diverse etiologies. Urinary fatty acid binding protein 1 (FABP1, 14 kDa) and neutrophil gelatinase-associated lipocalin (NGAL, 25 kDa) are useful biomarkers that enable a more accurate and earlier diagnosis of AKI in clinical settings. We have recently reported that urinary FABP1 is mostly derived from liver and synergistically enhanced by tubular and liver injury. Urinary NGAL is induced by tubular epithelial cell damage and released into urine. Urinary NGAL, which can be derived from serum, is also elevated by impaired protein reabsorption by proximal tubule injury. Acetaminophen, an analgetic drug, has hepatotoxicity as well as nephrotoxicity (acute tubular necrosis) when excess amount is used. In this study, we aimed to study the differential response of two biomarkers to acetaminophen-induced AKI in mice. Intraperitoneal administration of high dose acetaminophen (200 mg/kg) induced strong hepatotoxicity with eighty-fold elevation of serum FABP1 (control 3.6 vs. 292.3 ng/ml). Surprisingly, urinary FABP1 levels were exponentially increased seventy-seven thousand-fold (control 1.5 ng/day vs. 117.2 mg/day). Low dose administration (80 mg/kg) increased neither serum or urine FABP1. In contrast, high dose acetaminophen remarkably increased urinary NGAL twenty-sixfold (control 45.1 ng/day vs. 1198.6 ng/day) while low dose also modestly elevated it eightfold (390.3 ng/day). There was strong correlation between urinary FABP1 and NGAL ($r = 0.796$, $p < 0.0001$), suggesting common mechanisms by which both urinary markers are elevated. Thus, urinary FABP1 is a liver-derived biomarker for impaired protein reabsorption in AKI while urinary NGAL is superior to urinary FABP1 in the case of modest tubular damage.

### [2P-104]

### Protective Effects of House Cricket in Diabetic Nephropathy

*Nobuo Nagai[1], Yasuki Matano[1], Kenta Nomura[1], Atsushi Ogura[1] ([1]*Nagahama Institute of Bio-Science and Technology, Faculty of Bioscience*)

Diabetic nephropathy (DN) is a major complication of diabetes mellitus (DM). For developing DN, prolonged hyperglycemic exposure is necessary. and Therefore, its prevention is preferable not by drug but by continuous food consumption. Recently, insects have attracted attention as a source of protein because they grow efficiently with little food. In addition it has been reported that they have a variety of bioactive substances that are beneficial to humans and are expected to inhibit the development and progression of human diseases. Here we studied the effects of house cricket (*Acheta Domesticus*) powder (HCP) on renal dysfunction associated with diabetes in murine model. Naive C57BL/6J mice (Normal) or mice with treatment of streptozotocin (250 mg/kg/week) for DM induction were followed by continuous oral administration of Saline (SA) or HCP (3000 mg/kg) for 60 days, and their renal function was assessed by blood biochemical and histological analysis. 3000 mg/kg is equivalent to the protein intake of a male adult. It was found that blood glucose concentration significantly elevated in the DM+SA and DM+HCP groups compared to the Normal+SA group. Renal weight was significantly higher in the DM+SA and DM+HCP groups compared to the Normal+SA group. Both blood urea nitrogen (BUN) and blood creatinine (CRE) concentrations were also significantly higher in DM+SA group compared to the Normal+SA group. While both BUN and CRE concentration were comparable in DM+HCP group with Normal+SA group. Furthermore, it was also found that the marked glomerular atrophy and degeneration observed in the DM+SA group were alleviated in the DM+HCP group. These results suggested that house cricket consumption may improve renal dysfunction associating with renal glomerular degeneration in diabetes.

### Poster

### [2P]
### Autonomic nervous system

March 29, 13:00 - 14:20, Poster Room

### [2P-105]

### Involvement of the neural pathway from the lateral habenula to midbrain dopaminergic areas underlying the stress-related cardiovascular response

*Yuma Sato[1,2], Masayuki Matsumoto[3,4], Tadachika Koganezawa[1,4] (*[1]Dept Physiol, Inst Med, Univ Tsukuba, Tsukuba, Ibaraki, Japan, [2]Dr Prog Med Sci, Grad Sch Comp Human Sci, Univ Tsukuba, Ibaraki, Japan, [3]Dept Cogn Behav Neurosci, Inst Med, Univ Tsukuba, Tsukuba, Ibaraki, Japan, [4]Transborder Med Res Ctr, Univ Tsukuba, Ibaraki, Japan*)

The cardiovascular response is essential for stress-related behavior, such as freezing and fight or flight response. The lateral habenula (LHb), a small epithalamus nucleus, has been implicated in the coping stress. The LHb quickly responds to stressful event and send aversive information to other brain regions. The LHb neurons regulate the midbrain dopaminergic areas in stress processing. Thus, the LHb-dopaminergic system potentially has an important role in the stress-related cardiovascular response. However, the neural substrate in the cardiovascular response induced by stress events remains unclear. In this study, we examined the neural circuits of the LHb-dopaminergic system in the stress-related cardiovascular response.All experiments were performed with Wistar male rats (250~350 g) anesthetized by urethane (1 ~ 1.25 g/kg, *i.p.*). Mean arterial pressure (MAP) was measured from a catheter inserted into the femoral artery. Heart rate (HR) was calculated from the electrocardiography. The femoral vein was also canulated to administer the non-selective dopaminergic antagonist (clozapine, 1 mg/kg, *i.v.*). We electrically stimulated (300 µA, 0.5 ms duration, 100 Hz, for 10 s) the unilateral LHb and observed the cardiovascular response. The activation of the LHb phasically caused a pressor response and bradycardia. The administration of the clozapine attenuated the LHb-induced pressor response and bradycardia. Furthermore, we conducted the inactivation of the midbrain dopaminergic areas with activation of the LHb to investigate the dopaminergic areas' involvement in the LHb-originated cardiovascular response. We injected muscimol, a $GABA_A$ receptor agonist, into the ventral tegmental area (VTA) or the ventrolateral periaqueductal area (vlPAG) to inactivate these areas. We found that inactivation of the VTA attenuated the LHb-induced pressor response and bradycardia. On the other hand, inactivation of the vlPAG attenuated the LHb-induced pressor response and enhanced the bradycardia. These results indicate that dopaminergic neurons in the VTA mediate the excitation of the sympathetic and parasympathetic nervous system in the cardiovascular response originating from the LHb-stimulation, and these in the vlPAG mediate the sympathoexcitation.

### [2P-106]

### Histological analysis of DSS-induced colonic inflammation in transgenic mice with enhanced non-neuronal cardiac cholinergic system.

*Asuka Mano[1], Yoshihiko Kakinuma[1] (*[1]Department Bioregulatory Science, Nippon MedicalSchool*)

Introduction

Inflammatory bowel diseases (IBD) are chronic inflammatory disorders of the gastrointestinal tract and the pathogenesis of IBD has not been fully elucidated.DSS-induced colitis, which is the model of IBD, manifests as body weight loss, rectal bleeding, and diarrhea. To investigate the functions mediated by the non-neuronal cardiac cholinergic system (NNCCS), we established a murine model overexpressing choline acetyl transferase gene in the heart (ChAT-Tg). ChAT-Tg mice shows vagal nerve stimulatory effects in non-cardiac organs. Taken together, we induced DSS-induced colitis in ChAT-Tg mice to evaluate the effect of non-neuronal cardiac cholinergic system on colonic inflammation.

Methods

For acute DSS-induced colitis, mice received 1.5% DSS via the drinking water ad libitum for 7 days. Alterations of daily body weight, food intake, drinking and disease activity index (DAI) were measured. After 7days, the colon was transected and rolled from proximal to distal end according to swiss-roll method to evaluate histological damage of colitis severity. Extents of inflammatory cell infiltration and goblet cell depletion were evaluated by hematoxylin-eosin and Alcian blue staining, respectively.

Result

DSS-induced loss of body weight gain, food intake, drinking but there were comparable between WT mice and ChAT-Tg mice. DAI score was significantly increased in WT mice, in contrast, it was suppressed in ChAT-Tg. Histological analysis revealed that area of inflammatory cell infiltration was significantly increased and depletion of goblet cells were evident in WT mice. However, the depletional extent in ChAT-Tg mice was milder than that in WT mice.

Discussion

Exacerbation of colitis induced by DSS in WT mice was alleviated in ChAT-Tg mice. We previously reported that enhancement of NNCCS suppresses responses to systemic inflammation, so activation of NNCCS may restrain development of colitis.

Conclusion

Enhancement of the non-neuronal cardiac cholinergic system may suppress DSS-induced colonic inflammation in mice.

### [2P-107]

### Responses of sympathetic and vagal nerve activity to glucagon-like peptide-1 (GLP-1) receptor stimulation in conscious rats.

*Shizuka Ikegame[1], Kenju Miki[1], Misa Yoshimoto[1] (*[1]Nara Women's University*)

Glucagon-like peptide-1 (GLP-1) is a peptide hormone that plays an integral role in glucose homeostasis. Since GLP-1 receptors are expressed throughout the body, we hypothesized that responses of autonomic activity to GLP-1 receptor stimulation might be different depending on the stimulation site. However, the details of the effects of GLP-1 receptor stimulation on autonomic nervous system activity have not been reported. Therefore, this study investigated the effects of different sites of GLP-1 receptor stimulation on autonomic nervous system activity by administering GLP-1 receptor agonists via three routes: intravenous (iv) , intraportal vein (iport), and intraperitoneal (iperi). Male Wistar rats were chronically implanted with electrodes, catheters for measuring cervical vagal activity, renal and lumbar sympathetic nerve activity, electroencephalogram, electromyogram, electrocardiogram, arterial pressure, sensors for tissue fluid glucose concentration, and catheters for drug administration in the vein, portal vein, and abdominal cavity. Exendin-4, a GLP-1 receptor agonist, was administered to conscious rats via one of the following routes: venous, intra-abdominal, or portal vein. Renal sympathetic nerve activity was rapidly decreased by Exendin-4 administration and gradually recovered to pre-administration levels. There were no significant differences in renal sympathetic nerve activity among the different routes of Exendin-4 administration. On the other hand, lumbar sympathetic nerve activity increased slowly after Exendin-4 administration through iperi and iport routes while it decreased initially then gradually increased following the administration through iv route. Cervical vagal activity was increased in all routes of Exendin-4 administration. These data suggest that the responses of sympathetic nerve activity induced by GLP-1 receptor stimulation were found to have regional differences and stimulation site specificity.

### [2P-108]

### Distribution of the superior salivatory nucleus neurons innervating the salivary glands, blood vessels of the tongue, and lacrimal glands and the responsivenesses to orexin

*Yoshihiro Mitoh[1], Kengo Horie[1], Tadasu Sato[2], Takehiro Yajima[2], Ichikawa Ichikawa[2], Ryusuke Yoshida[1] ([1]*Dept. Oral Physiol., Okayama Univ. Grad. Sch. Med. Dent. Pharm. Sci.,* [2]*Div. Oral Craniofacial Anat., Tohoku Univ. Grad. Sch. Dent*)

The superior salivatory nucleus (SSN) is the primary parasympathetic center, which projects to the submandibular and sublingual glands, blood vessels of anterior tongue, and lacrimal glands via the submandibular ganglion, intralingual ganglion, and pterygopalatine ganglion, respectively. In this study, we examined the distribution of cell bodies for each target tissue in SSN, and investigated whether tongue neurons are excited by orexin A (OXA), an orexigenic peptide, since previous our study showed that salivary gland neurons are activated by OXA. In anatomical study, salivary gland neurons and lacrimal gland or tongue neurons were retrogradely labeled with Dextran-Texas Red and Dextran-fluorescein, respectively in adult rats. In electrophysiological study, whole-cell patch-clamp recordings were made from salivary gland or tongue neurons retrogradely labeled with Dextran-Texas Red in neonatal rat brain slices. The area of distributions for salivary gland, lacrimal gland, and tongue neurons were similar to previous results, but salivary gland and tongue neurons were intermingled, and lacrimal gland neurons were distributed separately in the ventral part of area for salivary gland neurons. Both salivary gland and tongue neurons generated inward currents with dose-dependent manner in response to OXA at a holding potential of -70 mV, and their curves were similar. However, the average frequency of mEPSCs for salivary gland neurons was 3.81±0.71 Hz (n=12), which was significantly faster than that for tongue neurons, 0.85±0.1 Hz (n=21). The frequency of mEPSC for both types of neurons did not change in the presence of OXA. Therefore, the effect of OXA was considered to be mainly mediated via OX receptors in the postsynaptic membrane rather than the presynaptic membrane.

### [2P-109]

### Autonomic responses to emotional changes during sports video viewing

*Kirari Wada[1], Tsugutake Yoneda[1], Hidefumi Waki[1], Ko Yamanaka[1] ([1]*Physiology, Health and Sports Science, Juntendo University, Japan*)

The viewing of sports, music, and arts often induces transitory pleasurable feelings and autonomic responses such as goosebumps. Accordingly, previous studies have examined physical reactions, autonomic responses (goosebumps and chills), and brain activity. In these studies, it has been shown that heart rate, respiratory dynamics, and electric skin conductance are changed by listening to emotional music. It has been reported that the prefrontal cortex and superior temporal gyrus are activated during emotional music listening. However, the physiological mechanisms underlying sports video viewing-induced emotional changes remain unclear. In this study, we recorded autonomic responses (blood pressure, heart rate, respiratory rate, and electric skin conductance) and brain activity (electroencephalogram) during emotional sports viewing in 30 healthy participants (15 men and 15 women). In addition, the appearance of goosebumps was captured by a camera. Participants were instructed to press a button when they subjectively felt "moved" by watching a sports video (SPO; sports video condition), or during an instructed time of viewing an upside-down, left-right, and reverse-playback video (CON; control condition) to exclude button-pressing motion-related activities. The results revealed that, in the SPO condition, the average number of button presses per participant was 35.3 ± 13.5 times. The heart rate increased immediately before the button press of the emotional report, and the response of electric skin conductance changed immediately thereafter in the SPO condition compared to the CON condition. Furthermore, alterations in blood pressure were noted following button presses during the SPO task. These results suggest that emotion during sports video viewing may trigger sympathetic responses at different times.

### [2P-110]

### Analysis of immune responses of macrophage cells of the adrenal gland in response to acute stress via sympathetic nervous system pathway

*Hiromasa Higuchi[1,3], Shuei Sugama[2] ([1]*International University of Health and Welfare Graduate School, Health and Welfare Sciences,* [2]*International University of Health and Welfare, Center for Basic Research,* [3]*Dokkyo Medical University, Nikko Medical Center*)

Stress is known to have various effects on behavioral and immune responses. In this study, we investigated the effects of stress on macrophages (MΦ) in the adrenal gland (AG). This study was focused on the hypothalamus-sympathetic-adrenal medulla pathway (SAM system). Wistar rats were divided into several groups, such as control (CTRL), acute restraint stress (aRS, restraint for 2 hours), phenylephrine (PE, 50 ug/kg), isoproterenol (ISO, 50 ug/kg), and propranolol (PROP, 10 mg/kg), respectively. After the perfusion with 4% paraformaldehyde, the AG was investigated with immunohistochemical procedures. The intensity of Iba1 immunoreactivity in a certain yet fixed area was taken as an indicator of MΦ activation. The fluorescence intensity of tyrosine hydroxylase and dopamine-beta-hydroxylase immunoreactivity were also used to indicate if the animal perceived each treatment as stressful stimuli. The result demonstrated that Iba1 immunoreactive cells altered their morphology in aRS, PE, and ISO, respectively, with their responses varying depending on the treatment. In the PROP group, however, Iba1 immunoreactive cells did not show any change. As a whole, this study demonstrated that Iba1 immunoreactive cells, presumably peripheral macrophage, immediately responded to those stressful stimuli, suggesting that the noradrenergic sympathetic nervous system may be involved in the modulation of macrophage cells' activity.

### [2P-111]

### A study to investigate the effect of sympathetic nervous system stimulation on food intake as well as behavioral activity of rats

*Soshi Nishina[1], Shuei Sugama[1] ([1]*International University of Health and Welfare*)

### [2P-112]

### Changes in renal and lumbar sympathetic and cervical vagal nerve activity patterns induced by the development of type 1 diabetes mellitus

*Haruka Hagi[1], Fuka Iwama[1], Shizuka Ikegame[1], Kenju Miki[1], Misa Yoshimoto[1]
([1]*Laboratory of Autonomic Physiology Faculty of Human life and Environment Nara Women's University*)

### [2P-113]

### Activation of afferent vagal nerves in the gastro-intestinal organs by irisin, one of exercise hormones, in mice

*Yuichiro Kimoto[1], Mamoru Tanida[1], Yasutaka Kurata[1] ([1]*Department of physiology 2, Kanazawa Medical University*)

### Poster

### [2P]
### Physical fitness and sports medicine

March 29, 13:00 - 14:20, Poster Room

### [2P-114]

### Landing position of trampoline gymnasts with stable occlusal balance reflects postural control function

*Mutsumi Takahashi[1], Yogetsu Bando[2], Takuya Fukui[3,4], Akiko Maruyama[3,5], Masaaki Sugita[6], Yoshihide Satoh[1] ([1]*Dept Physiol, Nippon Dent Univ, Niigata, Japan,* [2]*BANDO Dental Clinic, Ishikawa, Japan,* [3]*Dept Sports Sci, Kanazawa Gakuin Univ, Ishikawa, Japan,* [4]*JGA, Tra, Commit, Tokyo, Japan,* [5]*JGA, Tra, Reinforce, Tokyo, Japan,* [6]*Facul Sport Sci, Nippon Sport Sci Univ, Tokyo, Japan*)

The aim of this study was to clarify the relationship between the landing position during consecutive straight jumps and standing posture stability of trampoline gymnasts with stable occlusal balance. Participants were 10 trampoline gymnasts (competition experience: ≥12 years), all of whom had stable occlusal balance. To assess postural control function, the displacements in the forward–backward (COP-FB) and the left–right directions (COP-LR) of the center of foot pressure were recorded under eyes-open and eyes-closed conditions. Landing positions during 10 consecutive straight jumps were recorded. The horizontal displacements from the center of the bed in the forward–backward (H-FB) and the left–right directions (H-LR) directions were recorded. Differences in COP displacement between visual conditions were analyzed, along with the correlations between COP displacement and landing position. No significant difference in COP displacement was observed between visual conditions. Significant strong positive correlations were observed between COP-FB and H-FB, and between COP-LR and H-LR ($p<0.05$). This study clarified that the landing position during consecutive straight jumps of trampoline gymnasts with stable occlusal balance reflects standing postural control function. This work was supported by JSPS KAKENHI Grant Number JP23K10617.

### [2P-115]

### Vocalization of "*Men*" with Nasal Sounds in *Kendo* Increases Exhaled Nitric Oxide Levels

*Hajime Arikawa[1], Taichi Sakamoto[1] ([1]*Chubu Gakuin University*)

[Aims] Nitric Oxide (NO) is a compound generated within the body that exhibits various physiological effects, including vasodilation by relaxing vascular endothelial cells. NO is abundantly produced in the paranasal sinuses. Previous research has indicated that humming, a form of vocalization that involves the nasal cavity, elevates the fractional exhaled nitric oxide (FeNO) level. Unlike oral exhalation, vocalizing nasal sounds may also increase FeNO. In *kendo*, the term "*Men* (/meN/)" is used to denote a specific striking area and involves such nasal vocalization. Therefore, it is conceivable that FeNO could increase when vocalizing "*Men*," which might contribute to enhanced arterial oxygenation in the lungs. To clarify the physiological characteristics unique to *kendo*, this study aims to verify whether FeNO levels increase upon the vocalization of "*Men*," which incorporates nasal sounds.

[Methods] Nine healthy male university students with experience in *kendo* and without any respiratory diseases were selected. Their FeNO levels were measured using a NObreath device (manufactured by Bedfont Scientific Ltd) under various conditions: regular oral breathing, regular nasal breathing, humming, and both single and continuous vocalization of "*Men*." An exhalation gas collection mask that covered the nose was fitted to the device for the measurement. Statistical analysis employed Steel's non-parametric test, using the humming condition as a control for multiple comparisons. The significance level was set at 5%, and effect sizes (ES) were also calculated.

[Results] FeNO levels registered at 151.4 ± 30.1 ppb during humming. In comparison, levels were 13.9 ± 4.0 ppb during regular oral breathing, 65.1 ± 6.7 ppb during regular nasal breathing, 41.6 ± 11.0 ppb during a single vocalization of "*Men*," and 141.9 ± 25.2 ppb during continuous vocalizations of "*Men*." Significant reductions in FeNO were observed during regular oral and nasal breathing, as well as during a single vocalization of "*Men*," when compared to humming (P=0.001 (ES=0.63), P=0.028 (ES=0.63), and P=0.005 (ES=0.63) respectively). No statistically significant difference was observed between humming and continuous vocalizations of "*Men*" (P=0.977 (ES=0.03)).

[Conclusion] Continuous vocalizations of "*Men* (/meN/)," which incorporate the nasal sounds of "*Me* (/me/)" and "*N* (/N/)," produced FeNO levels comparable to humming. These results suggest that FeNO levels can increase when vocalizing "*Men*" continuously in *kendo*, which could possibly enhance arterial oxygenation upon inhalation. Future research will further explore the impact of this phenomenon on arterial oxygenation.

### [2P-116]

### Acute effect of moderate aerobic exercise on the cerebral neural activity during task switching in healthy young and older adults: A randomized crossover study

*Ryota Asahara[1], Marina Fukuie[1], Daisuke Hoshi[1], Jun Sugawara[1], Takshi Tarumi[1] ([1]*National Institute of Advanced Industrial Science & Technology*)

**Background:** Although it has long been thought that physical exercise improves brain health, current evidence is inconsistent regarding its acute effect. **Objective:** To determine the acute effect of moderate aerobic exercise on task switching performance and its associated cerebral neural activity in healthy young and older adults**Methods:** This study used a crossover design enrolling 17 healthy young (8 women and 9 men, 25 ± 2 yr) and 19 older (10 women and 9 men, 62 ± 5 yr) adults. Each participant underwent two experimental conditions (i.e., exercise and control) on separate days in random order. In the exercise condition, participants completed a 30-min moderate-intensity aerobic exercise (60-70% of the age-predicted maximal heart rate) on a motor-driven treadmill. In the control condition, they sat quietly on a chair for 30 min. In both conditions, functional magnetic resonance imaging (fMRI) data were collected during task switching at 3 time points: baseline (before exercise or control condition) and 10 min (post-1) and 30 min (post-2) after both conditions. The main outcomes were task switching performance (error rate and reaction times) and the task-related cerebral neural activity.**Results:** Error rates and reaction times during task switching improved over time ($p < 0.05$) in both exercise and control conditions, although no significant effect of condition or condition-by-time interaction was observed. The improvements in task performance were similarly ($p > 0.05$) observed in young and older groups. The fMRI data showed significant decrease in the task-related cerebral activity over time ($p < 0.001$) in both conditions regardless of age, although no significant condition-by-time interaction was observed. No brain region showed increase in the task-related cerebral activity ($p > 0.05$ for time effect) in both conditions. **Conclusion:** Acute moderate-intensity aerobic exercise did not improve task switching performance and the task-related cerebral neural activity in healthy young and older adults. It is possible that the acute effect of exercise may be masked by practice or learning effect.

### [2P-117]

### Polyamine metabolism in rat soleus muscle atrophied with unloading

*Hideki Yamauchi[1], Shigeru Takemori[1] ([1]*The Jikei University School of Medicine*)

Aim: Small polycationic compounds, polyamines are ubiquitous cellular metabolites. Among polyamines, spermidine and spermine were documented to affect various cellular function including cell growth. We recently observed drastic changes in polyamine metabolism in atrophied skeletal muscle. In the present study, we aimed to evaluate correlation between atrophy and polyamine metabolism. Atrophy was induced by hind-limb unloading with tail-suspension with and without intermittent resistance exercise. Methods: F344 17-week female rats fed ad libitum were used. Rats were tail-suspended for 3 weeks to induce significant atrophy (n=7). The degree of atrophy with the tail-suspension was lowered by intermittent resistance exercise to induce moderate atrophy (n=7). In the resistance exercise, rats were placed on an upright 45-cm-long wire mesh tube of 6.5 cm diameter with a load of 50~70% of body weight attached to their tails. Each training bout lasted 10 min, 3 times per day every 4 hours in the dark period for 3 weeks. Rats for control muscle (n=7) were kept without tail-suspension and exercise. At the end of the intervention, soleus muscle was excised to examine protein expressions with western blotting. Results: Continuous 3-week unloading significantly decreased soleus mass to 53% of control. Western blotting indicated increase in the expression level of muscle specific ubiquitin ligase, MuRF1 to 143% of control. With intermittent exercise to induce moderate atrophy, decrease in soleus mass was ameliorated to 69% of control, and expression level of MuRF1 was suppressed to 120% of control. As for polyamine synthesis, s-adenosylmethionine decarboxylase, a key enzyme for the synthesis of spermidine and spermine, decreased significantly with continuous tail-suspension. On the other hand, spermidine/spermine $N^1$-acetyltransferase, which plays a key role in polyamine catabolism, increased with the continuous tail-suspension. Both changes in enzyme contents were suppressed by intermittent exercise. Concomitant increase in ornithine decarboxylase, spermidine synthase, and spermine synthase and decrease in spermine oxidase were affected by the intermittent exercise to smaller extents.  Discussion: Atrophy would correlate closely with polyamine metabolism. It is now more strongly suggested that polyamines might regulate anabolism and catabolism in muscle cells.

### [2P-118]

### Effects of voluntary exercise on cognitive dysfunction and anxiety-like behaviors in a mouse model of psychiatric disorders.

*Jonghyuk Park[1], Hiroko Shimbo[1,2], Shoko Tamura[1], Takatoshi Hikida[3], Haruo Okado[1], Shinobu Hirai[1] ([1]*Brain Metabolic Regulation Group, Department of Psychiatry and Behavioral Sciences, Tokyo Metropolitan Institute of Medical Science,* [2]*Clinical Research Institute, Kanagawa Children's Medical Center,* [3]*Laboratory for Advanced Brain Functions, Institute for Protein Research, Osaka University*)

**[Background]** One of the characteristics of psychiatric disorders (PD), including schizophrenia and bipolar disorder, is cognitive decline. Disrupted-in-Schizophrenia-1 (Disc1) is a gene associated with developing PD. Previous studies have reported that environmental stress during adolescence to Disc1 transgenic mice resulted in the severity of schizophrenia-like behavioral phenotypes. Physical exercise improves brain energy metabolism, which contributes to ameliorating cognitive dysfunction induced by stress. However, the effects of voluntary exercise on the PD are unknown. Therefore, we aimed to determine whether voluntary exercise during adolescence could ameliorate cognitive dysfunction observed in PD model mice in adulthood. **[Methods]** Male wild-type (WT) and model mice that mimic patient genome mutation of PD, Disc1 heterozygote (Disc1 het) mice, were used. Experiments were conducted in six groups. The 3-4 mice were housed, and grouped-house mice were used as controls (WT- or Disc1-Ctr). The other mice were housed alone (isolation stress) from four weeks of age (WT- or Disc1-Iso). Simultaneously, the other isolation mice were allowed a voluntary wheel running in their cages (WT- or Disc1-Iso+WR). After six weeks, all mice performed behavior tests as follows: open field test (OFT), object location test (OLT), elevated plus maze (EPM), and prepulse inhibition test (PPI). After a battery of behavior tests, these mice's brain sections were used for immunohistochemical analysis. **[Results]** Total traveled distances and time in the center in the OFT did not differ significantly among the six groups; however, total traveled distances tended to be higher for the Disc1-Iso mice than for Disc1-Ctr and -Iso+WR mice. OLT scores significantly decreased in both Iso groups compared to Ctr mice. A marked decrease in OLT score was observed in the Disc1-Iso mice, while voluntary exercise prevented stress-induced cognitive dysfunction. In the EPM test, anxiety-like behavior was observed in Disc1-Iso mice, which was prevented by exercise. **[Conclusion]** Environmental stress of isolation during adolescence is a significant aggravating factor for cognitive decline and anxiety-like behavior in PD, while voluntary physical exercise induces a protective role against these abnormal behaviors in a mouse model of PD.

### [2P-119]

### Physical activity of mice after denervation – application of deep learning-based detection system

*Naomi Teranishi[1], Naoya Nakahara[1], Shigeru Takeru[1] ([1]*The Jikei University School of Medicine*)

[Background] Unilateral sciatic nerve denervation is a common method to induce experimental muscle atrophy. After denervation, paralyzed muscles secondarily receive passive extension concomitant with physical activity supported by unaffected muscles. Monitoring detailed mouse activity is crucial for assessing these secondary effects. Deep learning-based image recognition can provide valuable assistance in tracking mouse activity. As a starting point, we developed an auto-tracking system of a mouse in low-contrast environments and applied the system to evaluate post-operative activity of denervated mice. [Animals] Four white mice (ICR; 8W) were individually housed in cages of white bedding, and continuously video recorded with infrared surveillance cameras (ATOM Cam Swing). Unilateral denervation of sciatic nerve was performed under deep anesthesia. Hindlimb muscle mass was quantified with an X-ray CT device. [System] We further trained a pre-trained image recognition system (Detectron2 + Mask R-CNN-R50-FPN) using 1,200 manually labeled images of the mice in cages.[Validation] Comparison with manual assessment of 60-second video images during the pre-denervation dark period validated the system's accuracy in detecting mice. Observed small differences (<5-mm) were mainly due to manual assessment errors. Automatically tracked mice activity was significantly high during the dark period compared with the light period. This result is consistent with the nocturnal nature of mice and validate little effect of surveillance cameras on mice activity during the dark period. [Denervation] Judging from the moving distance of mice, denervation caused limited effects on the overall activity of mice. A significant drop was observed only on the day of denervation procedure. Mice activity gradually recovered in a week, despite 35% loss of paralyzed muscle mass in 4 weeks. [Conclusion] Our automatic system satisfactorily tracked mice even in a low-contrast dark condition and indicated limited effects of denervation on overall activity of mice. Therefore, physical activity of paralyzed and compensating muscle should be carefully assessed to evaluate denervation effects on the muscle. We are planning to improve our image recognition system to categorize mice activity to estimate passive and active activities of paralyzed and compensating muscles after a denervation procedure.

### Poster

### [2P]
### Nutritional and metabolic physiology, Thermoregulation

March 29, 13:00 - 14:20, Poster Room

### [2P-120]

### Effect of estrogen and leptin on palatable sucrose solution intake and energy balance in ovariectomized rats

*Natsumi Kosugi[1], Sayaka Eguchi[1], Nanako Sakagawa[1], Aoi Takahashi[1], Mio Nishimaki[2], Akira Takamata[2] ([1]*Graduate school of Humanities and Sciences, Nara Women's University,* [2]*Department of Environmental health, Nara Women's University*)

Estrogens are known to exert hypophagic and antiobesity actions. We have reported that estrogens also enhance palatable 10% sucrose solution (10%-SS) intake. Leptin attenuates food intake and body fat accumulation. In the present study, we examined the effect of estradiol and leptin on 10%-SS and energy intakes.
Female Wistar rats were ovariectomized and implanted subcutaneously with a silicon capsule containing either estradiol (E2 group) or cholesterol (Veh group). In experiment 1, rats received chronic systemic leptin (38.4 µg/day) or saline administration using a subcutaneously implanted osmotic pump for 14 days. In experiment 2, rats received a bolus leptin (10 µg/10 µL) or saline administration in the lateral ventricle. All rats in these experiments were provided access to 10%-SS, water, and standard rodent chow, and intakes of these were measured.
Food intake was less and 10%-SS intake was greater in the E2 group than those in the Veh group without leptin administration. In the Veh group, chronic systemic leptin administration attenuated food intake, while the leptin administration increased 10%-SS intake. By contrast, in the E2 group, chronic systemic leptin administration did not affect food intake or 10%-SS intake. Intracerebroventricular administration of leptin attenuated food intake and 10%-SS intake in the Veh and E2 group. In the Veh group, both chronic systemic and intracerebroventricular administration of leptin enhanced the sucrose preference, defined as the fraction of energy intake from the 10%-SS to total energy intake, but had no effect in the E2 group. As a result, the sucrose preference in the leptin administered Veh group became comparable to that in the E2 group.
These results suggest that leptin, like estrogens, may enhance the preference of sweet solution in addition to decreasing energy intake, and leptin affects the preference of sweet solution in an estrogen level-dependent manner.

### [2P-121]

### Sex differences and effects of sex hormones on high-fat diet preference and body weight regulation

*Aoi Takahashi[1], Natsumi Kosugi[1], Sayaka Eguchi[1], Nanako Sakagawa[1], Mio Nishimaki[2], Akira Takamata[2] ([1]*Graduate School of Humanities and Sciences, Nara Women's University,* [2]*Department of Environmental Health, Nara Women's University*)

Excessive consumption of high-fat diets (HFD) is thought to induce obesity, and sex hormones are known to be involved in the regulation of eating behavior. However, our previous studies failed to find that exclusive feeding with HFD increases food intake and body weight in rats of both sexes. To clarify the hypothesis that overeating and obesity may occur when rats were given free access to both HFD and normal diets (ND) simultaneously, we examined food/energy intake, body weight and preference for HFD in rats that had free access to both HFD and ND in the present study. We also evaluated the effects of sex differences and sex hormones on the regulations of energy intake, body weight and the preference for HFD. Rats were assigned to the following six groups: intact male (M group), intact female (F group), ovariectomized and estradiol replaced female (E2 group) or vehicle treated female (Veh group), orchiectomized male (ORX group), and sham-operated male (Sham-M group). Each group was divided into two subgroups: one had free choice of ND and ND (ND group) and the other had free choice of HFD and ND (HFD group). We measured daily food/energy intake, water consumption, and body weight for 30 days.The preference for HFD, calculated as the fraction of energy intake from HFD to total energy intake, was significantly lower in the F group than in the M group. The preference was similar between the E2 group and the Veh group. On the other hand, the preference for HFD was significantly higher in the Sham-M group than that in the ORX group. These suggest that endogenous androgens are possibly involved in the sex differences in the preference for HFD. Furthermore, both food/energy intake and body weight were significantly greater in the HFD group than the ND group in the M and Sham-M groups, but not in the F, E2, or ORX groups. These results indicate that endogenous androgens cause excessive food/energy intake and body weight gain by elevating preference for HFD in male rats given free access to HFD and ND.

### [2P-122]

### The effect of serotonin depletion in the periventricular area and the perifornical area of the lateral hypothalamus on the estrogen-induced modulation of feeding behavior

*Nanako Sakagawa[1], Natsumi Kosugi[1], Sayaka Eguchi[1], Aoi Takahashi[1], Mio Nishimaki[2], Akira Takamata[2] ([1]*Graduate School of Humanites and Sciences, Nara Women's University,* [2]*Department of Environmental Health, Nara Women's University*)

Both estrogens and serotonin (5-HT) are known to have anorectic effects. Estrogens reportedly act on the serotonin neurons in the dorsal raphe nucleus and increases serotonin levels in the central nervous system. In the present study, we examined the interaction between estrogens and 5-HT in the regulation of feeding behavior. Female Wistar rats were ovariectomized and implanted subcutaneously with a silicone capsule containing either estradiol (E2 group) or cholesterol (Veh group). Each group was injected with 5,7-Dihydroxytryptamine (5,7-DHT) or saline into the lateral ventricle or into the bilateral perifornical area of the lateral hypothalamus (PF-LH). Feeding behavior, including circadian feeding rhythm and feeding patterns, were measured.Food intake during the light phase, food intake and c-Fos expression of the orexin neuros in the PF-LH in response to intraperitoneal 2-deoxy-D-glucose (2DG) were less and c-Fos expression in the suprachiasmatic nucleus (SCN) during the light phase was greater in the E2 group than in the Veh group. Intracerebroventricular 5,7-DHT reversed these E2 effects in the E2 group. Depletion of 5-HT in the PF-LH reduced the anorectic effects of E2 by eliminating the anorectic effect during the light phase, while depletion of 5-HT in the PF-LH had no impact on the food intake and the neural activity of orexin A neurons in response to 2DG. Our findings suggest that serotonin is possibly involved in the estrogen's anorectic effect. However, serotonin is not likely to be directly involved in the regulation of the orexin neurons in the PF-LH, which is attenuated by estrogens.

### [2P-123]

### Neuronal subpopulation of nucleus accumbens medial shell suppresses food intake during sickness

*Ryo Hara[1], Yuka Terakoshi[2], Takeshi Sakurai[2], Katsuyasu Sakurai[2] ([1]*Graduate School of Comprehensive Human Sciences, University of Tsukuba,* [2]*International Institute for Integrative Sleep Medicine (WPI-IIIS), University of Tsukuba*)

Animals change behaviors and adapt to various physiological challenges. Food intake generally decreases during sickness, however, the neuronal mechanism underlying this phenomenon has not been fully understood. First, to determine the brain regions that regulate feeding suppression during sickness, we performed whole-brain neuronal activity mapping. We found that cFos expression in the nucleus accumbens medial shell (NAcSh) significantly increased in LPS-induced sickness conditions, and then we hypothesized that this neuronal population suppresses food intake during sickness. To test this hypothesis, we adopted the CANE method to specifically reactivate neurons that were activated in the LPS condition. CANE makes any genes expressed in neurons that express cFos in an activity-dependent manner. By using CANE and DREADD, we artificially reactivated neurons and found that food intake significantly decreased. Finally, we investigated the projection sites of these neurons, and axons of these neurons were found in some brain regions, such as the lateral hypothalamus.

### [2P-124]

### Role of proteinase-activated receptor 1 in the development of age-related insulin resistance

*Takeshi Hashimoto[1], Natsuko Tsurudome[1], Katsuya Hirano[1] ([1]*Dept Cardiovasc Physiol, Fac Med, Kagawa Univ*)

**Background**: Insulin resistance develops with aging, owing to chronic inflammation in the adipose tissues. It is also known that the coagulation activity increases with aging, in both systemically and locally in the adipose tissues. The coagulation factors with proteinase activity cause not only blood coagulation, but also chronic inflammation by activating G protein-coupled receptor, referred to as proteinase-activated receptor (PAR). However, the relationship between hyper-coagulability and insulin resistance in the elderly remains elusive. We investigated the role of $PAR_1$ in the development of age-related insulin resistance using $PAR_1$-knockout mice (KO).
**Main results**: The body weight gain with aging in KO mice was significantly lower than that of WT mice, although there was no significant difference in food intake between two groups. The locomotor activity of KO mice during the nighttime was significantly higher than that of wild-type mice (WT) until 28 weeks of age. The younger hair condition was maintained in the aged KO mice compared to the aged WT mice. The insulin sensitivity decreased with aging in WT mice. At 50 weeks of age, the insulin sensitivity of KO mice was significantly higher than that of WT mice. The total weight of epididymal, perirenal, interscapular, and gluteofemoral adipose tissues increased with aging in WT mice, while this increase was suppressed in KO mice. When fed a high-fat diet from 8 to 20 weeks of age, the insulin resistance developed similarly between WT and KO mice, with similar increase in the body weight. Treatment of WT mice with $PAR_1$ antagonist from 8 to 80 weeks of age or from 50 to 80 weeks of age had no significant effect on the development of insulin resistance at 80 weeks of age.
**Conclusions**: The genetic deletion, but not pharmacological inhibition, of $PAR_1$ specifically prevented the development of the age-related insulin resistance in mice. $PAR_1$ is suggested to be a novel therapeutic target for prevention and treatment of the age-related diabetes mellitus.

### [2P-125]

### The activity of the medial and dorsolateral prefrontal cortex during udon eating is influenced by food preferences.

*Coco Yamazaki[1], Maruhashi Karen[1], Yoshida Keisuke[1], Takatsuru Yusuke[1] ([1]*Dept. Nutr. Health Sci., Toyo Univ.*)

### Poster

### [2P]
### Behavior, Biological rhythm, Sleep

March 29, 13:00 - 14:20, Poster Room

### [2P-126]

### Nicotinic acetylcholine receptors in the suprachiasmatic nucleus modulate photic phase resetting of circadian rhythms in mice

*Sae Nakamura[1], Hitoshi Okamura[3], Keiko Tominaga[1,2] ([1]*Graduate School of Science, Osaka University*, [2]*Graduate School of Frontier Biosciences, Osaka University*, [3]*Graduate School of Medicine, Kyoto University*)

Many physiological phenomena and behavioral activities in living organisms exhibit circadian rhythmicity (approximately 24 hours), driven by a circadian clock. In mammals, the suprachiasmatic nucleus (SCN) of the hypothalamus contains the central circadian clock responsible for generating endogenous rhythms and synchronizing these rhythms to the environmental light-dark cycles. A light pulse, the strong zeitgeber, shifts the phase of the clock in a phase-dependent manner. Light in the early subjective night elicits phase delay, and light in the late subjective night produces phase advance, whereas, in the subjective day, light pulse shows no effect on circadian rhythms. Internal signaling, such as sleep/wake states, may also affect the SCN circadian clock. The cholinergic system in the brain is one of the essential systems regulating sleep/wake states. It is known that the SCN receives some cholinergic projections from the basal forebrain and brainstem, suggesting that ACh signaling in the SCN may play a role in modulating circadian rhythms. However, the function of the nicotinic acetylcholine receptors (nAChRs), but not muscarinic acetylcholine receptors, in the SCN is not well understood. In this study, to elucidate interactions between nAChRs signals and light information in the SCN, we examined the effects of nAChRs antagonist (mecamylamine, MEC) on the light-induced phase shifts in the behavioral activity rhythms and the expression of light-induced factors in the SCN involving in the phase shifts. MEC was microinjected into the third ventricle just above the SCN. MEC did not affect the endogenous behavioral rhythms under DD conditions, showing that nAChRs signal may not be involved in the modulating phase and free-running period of endogenous rhythms. However, MEC significantly attenuated the light-induced phase delay in the early subjective night but not the phase advance in the late subjective night. MEC administration also significantly reduced the light-induced c-Fos and *Per1* expressions and phosphorylation of MAPK in the SCN, suggesting that the inhibitory effects of MEC on the light-induced phase delay in behavioral rhythms produced by attenuation of light-activated intracellular signals in the SCN. These results indicate that nAChRs in the SCN may not affect the endogenous rhythms but potentiate the phase-shifting effects of light in the early subjective night and that the interaction between photic information and cholinergic inputs via nAChRs exists in the SCN.

### [2P-127]

### Positive effects of mindfulness meditation and reduced screen light emission on sleep quality in nocturnal smartphone user

Prabha Gurung[1], Pui Lam Tang[1], *Chuan LI[1] ([1]*Tung Wah College*)

Nocturnal smartphone exposure is one of the most important environmental factors that induces sleep disturbance in the young people. The screen light and the stress associated with the bedtime phone use induce the late sleep, the increased sleep latency, the wake-up in the night and the poor alertness in the morning. This study aimed to measure the effects of mindfulness meditation (MM) and the reduced screen light exposure on the sleep in the young adults experiencing the excessive bedtime phone use. Karolinska Sleepiness scale (KSS) questionnaire, sleep onset latency and sleep efficiency were recorded for evaluating the subjective and objective effects on sleep quality.In the first experiment, the effects of mindfulness meditation (MM) on sleep quality were measured. It is reported that the stress and arousal induced by bedtime smartphone use is closely associated with the poor sleep of young adults. The mindfulness practice may improve the emotional regulation and reduce stress of subjects, and hence to be a possible tool to improve the sleep of nocturnal smartphone user. In current study, young adults were randomly assigned to two groups, MM group and control group. Participants in both groups were required to use smartphone at least 3 hours before sleep at 12 am. MM group was required to join an online breathing practice via zoom for 15 minutes before going to bed while control group was required to stop using phone for 15 minutes in the same period of time. After MM practice for four days, the subjective and objective sleep quality data of participants were collected and analysed. When compared with control group, the average sleep onset latency in MM group was significantly reduced. However, no significant differences were found in sleep efficiency and KSS score. In the second experiment, the effects of reduced screen light emission, both white lightand short-wavelength light, on the sleep quality were investigated. Young adults were exposure to three different light conditions in three non-consecutive nights in three weeks, including white light screen emission (control), low white screen light emission (low-WL) and a reduced short-wavelength condition (low-SW). Sleep onset latencies were shortened significantly in low-SW night but not in low-WL nights when compared with control nights. KSS questionnaire indicated that the subject felt more alert in the morning after low-WL night than control nights. Our results suggest that both mindfulness meditation and the lower light screen emission decreases negative effect of bedtime smartphone use and promotes sleep quality in young adults.

### [2P-128]

### Oral Ingestion of Monosodium Glutamate Decreases Strong Aggression Related to Nucleus of Solitary Tract Activation via Vagus Nerve

*Dewi Mustika[1], Yu Nishimura[1], Shinya Ueno[1], Naoki Tajiri[1], Cha-Gyun Jung[1], Hideki Hida[1] ([1]*Department of Neurophysiology and Brain Science, Nagoya City University Graduate School of Medical Sciences, Nagoya, Japan*)

Monosodium glutamate (MSG), an umami substance, stimulates the gut-brain axis via gut umami receptors and subsequent vagus nerve activation. However, the brain mechanism underlying the effect of MSG ingestion during the developmental period has not been clarified yet. To answer this question, we used an aggressive rat strain, SHR/IZM, a model of attention-deficit hyperactivity disorder (ADHD). SHR/IZM rats were housed individually after weaning (postnatal day 25$^{th}$: P25) to escalate the aggressive behavior, and 60 mM MSG was orally ingested for five weeks. Aggression was then assessed using the resident-intruder test at P60: Wistar/ST rat weighing 0 to 30 g less than the resident was used as an intruder. The frequency, duration, and latency of anogenital sniffing (weak aggression), aggressive grooming (moderate aggression), and attack behavior (strong aggression) for 10 min a day in the dark phase were analyzed for three consecutive days. We revealed that MSG ingestion significantly decreased the frequency and duration of aggressive grooming and attack behavior and increased the latency of attack behavior compared with the control group ($p<0.05$). Furthermore, c-Fos immunostaining was performed in the nucleus of the solitary tract (NTS) and the aggression-related areas. Oral MSG ingestion increased the number of c-Fos$^+$ cells in the intermediate NTS (iNTS) ($p=0.0094$) and decreased its in the central amygdala ($p=0.0084$). However, the number of c-Fos$^+$ cells was comparable in the prefrontal cortex and lateral hypothalamus between the MSG-treated and control groups. Total vagotomy at the subdiaphragmatic level successfully diminished the MSG effect on strong aggression: the frequency and the duration of the attack were recovered to the control group, showing that the vagus nerve activation has an important role in MSG effect on aggression ($p<0.05$). Our results suggest that oral ingestion of MSG decreases isolation-induced aggression in a rat model of ADHD mediated by the vagus nerve relating to c-Fos activation in the iNTS and its inactivation in the CeA.

### [2P-129]

### Effect of patterns of RR intervals during sleep onset on sleep parameters and subjective evaluation of sleep in perimenopausal women

*Michiko Tanaka[1], Aki Nozue[2], Mou Nagasaka[1], Miyuki Matsuyama[3], Chiyomi Egami[3] ([1]*Miyazaki Prefectural Nursing University,* [2]*Miyazaki University,* [3]*Fukuoka Prefectural University*)

This study aimed to classify patterns of RR intervals during sleep onset in perimenopausal women, determine how each pattern affected sleep status, and subjectively evaluate sleep. Thirteen working women aged 45–55 years were included. Measurements of sleep status and the RR intervals during sleep were conducted at home for five days. During the five-day period, sleep measurements were conducted on at least two working days and two holidays; the first day could either be a working day or a holiday. Sleep status was measured using a daily sleep diary and a sheet-shaped device (Nemuri SCAN, Paramount Bed). The RR interval data was obtained using a heart rate monitor (myBeat, Union Tool). High frequency (HF) and low frequency (LF)/HF ratio, indicators of the autonomic nervous system, were calculated from RR intervals. The sleep onset of the subjects was classified into three patterns (A–C) based on the RR intervals. The Kruskal–Wallis test was used to examine sleep parameters and subjective sleep comparisons between each pattern. The differences between workdays and holidays were analyzed using the Mann–Whitney U test. The relationship between subjective evaluation of sleep and sleep parameters in the three patterns was deduced using Spearman's rank correlation coefficient. All analyses were conducted using SPSS ver. 27.0. Among the 72 nights of sleep available for analysis in 13 subjects, the distribution of nights was 41, 12, and 19 nights for patterns A, B, and C respectively. Pattern A showed a prolongation of the RR interval after falling asleep, followed by a shortening of the interval, and then a further prolongation. Pattern B was similar to pattern A; however, the amplitude of the changes was smaller. Pattern C demonstrated repeated prolongations and shortenings of the RR interval for a short time immediately after falling asleep, followed by prolongation. Differences were also observed with respect to working days and holidays. With regards to the relationship between subjective evaluation and sleep parameters in overall sleep onset, subjective evaluation was found to have a positive correlation with the total score of the OSA sleep evaluation form and negative correlation was observed with menopausal symptoms. In pattern A, subjective evaluation of sleep was positively correlated with sleep duration, bedtime, and total score of the OSA sleep evaluation form, and negatively correlated with daily activity, sleep efficiency, and menopausal symptoms. In patterns B and C, there was a positive correlation with the total score on the OSA sleep evaluation form and a negative correlation with menopausal symptoms. We have been observing differences between items that correlated with subjective evaluation of sleep in each pattern of RR intervals at sleep onset. This suggests that autonomic nervous system responses during sleep may influence sleep quality. (Supported by JSPS KAKENHI 23K10064).

### [2P-130]

### Relationship between structural-LTP and NREM sleep-inducing SIK3 pathway

*Motoki Juichi[1], Chika Shimizu[1], Miyo Kakizaki[1], Kei Nishida[1], Shinnosuke Nomura[1,2], Takeshi Kanda[1], Takeshi Sawada[2], Hiromasa Funato[1,3], Yusuke Iino[1], Shoi Shi[1], Masashi Yanagisawa[1] ([1]*WPI-IIIWPI-IIIS, University of Tsukuba, Ibaraki, Japan,* [2]*Faculty of Medicine, The University of Tokyo,Tokyo,Japan,* [3]*Department of Anatomy, Toho University, Tokyo, Japan*)

Daily sleepiness, or increased sleep need, is prominent after an all night awaking. To counteract this, one might sleep longer and deeper than usual the following day. This balance of sleep amount and depth, termed sleep homeostasis, compensates for sleep loss. However, the underlying mechanisms and substances regulating sleep need are still under investigation . The "SHY hypothesis" suggests that increased synaptic strength during wakefulness induces sleep, which is then offset during sleep. Studies have indicated a correlation between sleep need intensity and dendritic spine size, which is correlated with cortical excitatory synaptic strength. In our research, we observed spine enlargement in the prefrontal cortex (PFC) of wild-type mice during the early light period, when daily sleep need is high. Furthermore, we found that an induction of structural long-term potentiation (sLTP) via spine enlargement in the PFC of wild-type mice increases the amount and depth of NREM sleep. Yet, the molecular mechanism is elusive. To investigate it, we focused on the SIK3 signaling pathway, where the kinase activity of SIK3 might encode sleep need, subsequently inducing NREM sleep. Single-cell RNA sequencing suggests that the SIK3 signaling pathway may regulate synaptic plasticity factors, such as BDNF and Nptx. Moreover, phosphorylation of synaptic proteins increases with sleep need. Thus, we explored the relationship between the SIK3 pathway and sLTP. Through EEG-/EMG-based sleep/wake analysis on whole-brain excitatory neuron SIK3-deficient (SIK3 cKO) mice, we found that SIK3 cKO mice lacked the increase of amount and depth of NREM sleep following sLTP induction in PFC. This suggests that the SIK3 signaling pathway modulates NREM sleep in response to sLTP. We are further investigating the relationship between the SIK3 signaling pathway and sLTP by assessing the spine size of SIK3 mutant mice and investigating sLTP alternations when manipulating SIK3 activity with drugs in primary neurons.

### [2P-131]

### Effects of mild chronic stress on sleep and pain thresholds in mice.

*Sachiko Chikahisa[1,2], Junhel Dalanon[3], Parimal Chavan[3], Tetsuya Shiuchi[2], Noriyuki Shimizu[2], Kazuo Okura[3], Yoshitaka Suzuki[3], Yoshizo Matsuka[3], Hiroyoshi Séi[2] ([1]*Dept Health and Nutrition, Shikoku Univ.,* [2]*Dept Physiology, Tokushima Univ Grad Sch of Biomed Sci.,* [3]*Dept Stomatognathic Function and Occlusal Reconstruction, Tokushima Univ Grad Sch of Biomed Sci.*)

Stress is known to cause the onset of sleep disturbances and increased pain sensitivity, but the mechanism is unknown. The purpose of this study was to investigate the effects of predictable mild chronic stress (PCMS) on sleep and pain sensitivity. Eight-week-old C57BL/6 J male mice were divided into three PCMS groups: a control group with sawdust on the floor of the breeding cage (C), a group with wire mesh on the floor (M), and a group with water directly under the wire mesh (W). The animals were divided into three PCMS groups and kept for 21 days for sleep recording and pain sensitivity evaluation. Pain sensitivity was evaluated by tail immersion and hot plate tests for sensitivity to thermal stimuli, and by tail pinch test for sensitivity to mechanical stimuli. Experiments were conducted under ethical considerations after approval by the University of Tokushima Animal Experiment Committee. Sleep measurements revealed that mice in the M and W groups showed a decrease in the amount of non-rapid eye movement (NREM) sleep and a decrease in slow-wave activity (SWA) during NREM sleep. These mice reared in the PCMS environment showed enhanced pain sensitivity to thermal and mechanical stimuli. Therefore, we examined the relationship between SWA strength and pain sensitivity in each mouse and found a significant correlation between the two, indicating that the greater the SWA during NREM sleep, the lower the pain sensitivity. Furthermore, double immunostaining of orexin and c-Fos in brains from the lateral hypothalamus revealed that the number of activated orexin neurons was significantly increased in the M and W groups compared to the C group. We next performed the same experiment with suvorexant, an orexin receptor antagonist, and found that the increased sensitivity to thermal and mechanical pain observed in groups M and W was alleviated. In summary, our results suggest that rearing in a PCMS environment may reduce sleep quality and cause hyperalgesia to mechanical and thermal stimuli, and that the orexin system is involved in this mechanism.

### [2P-132]

### Role of estrogen receptor β-expressing neurons in the lateral septum in modulation of social anxiety in male mice.

*Kansuke Hasunuma[1,2], Mariko Nakata[1], Sonoko Ogawa[1] ([1]*Laboratory of Behavioral Neuroendocrinology, University of Tsukuba,* [2]*JSPS Research Fellow DC*)

Testosterone, after being converted to estradiol, regulates a variety of social behaviors, including social anxiety, by acting on estrogen receptors (ER) α and ERβ in male mice. The lateral septum (LS) is one of the key brain areas responsible for the regulation of male social behaviors. We have previously reported that site-specific knockdown of ERβ, but not ERα, in the LS enhanced levels of social anxiety in male mice measured in a social interaction test. Furthermore, we have found that ERβ-expressing LS neurons send dense projections to the anterior hypothalamus (AHA), which is known to be a part of the neural network for anxiety-like behavior in general. Thus, in the present study, we investigated the effects of chemogenetic manipulations of ERβ-expressing LS neurons on anxiety-like behavior in both social and non-social contexts, as well as on neuronal activation in various hypothalamic regions, including the AHA. ERβ-iCre male mice were injected with adeno-associated viral vectors either Cre-dependently transduced chemogenetic receptors for excitation (hM3Dq) or inhibition (hM4Di) of neuronal activity, or control viruses in the LS. They were subsequently subjected to light-dark transition (LDT) and social interaction (SI) tests to assess anxiety-like. All mice were treated (i.p.) with clozapine N-oxide (CNO) 30 minutes before each test. Excitation of ERβ-expressing LS neurons significantly reduced social anxiety-like behaviors in SI tests. Anxiety-like behaviors were not affected by suppression of ERβ-expressing LS neuronal activity in both tests. These results suggest that ERβ-expressing LS neurons influence anxiety levels, particularly in social situations. After all behavioral tests were completed, brain tissues were collected 100 minutes after CNO injection for immunohistochemical detection of c-Fos, as a marker of neuronal activity. We found that in the AHA, the number of c-Fos-positive cells was significantly higher in mice treated with hM3Dq viral vectors compared with those treated with control virus. These findings collectively suggest that the excitatory neuronal connection between ERβ-expressing LS neurons and the AHA is specifically implicated in the reduction of anxiety levels in social contexts in male mice. (Supported by KAKENHI 21J20176 to KH and 15H05724 and 22H02941 to SO.)

### [2P-133]

### Wistar rats raised in an affectionate environment display lifesaving-like behaviors while distinguishing life from death

*Kanta Mikami[1], Tomomi Doi[1], Yuka Kigami[1], Mohammed E Choudhury[1], Junya Tanaka[1] ([1]*Department of Molecular and Cellular Physiology, Ehime University Graduate School of Medicine*)

### [2P-134]

### Development of behavioral research methods to elucidate family/social relationships in rats

*Tomomi Doi[1], Kanta Mikami[1], Rintaro Shinabe[1], Chisato Yajima[1], Mohammed E Choudhury[1], Junya Tanaka[1] ([1]*Department of Molecular and Cellular Physiology, Ehime University Graduate School of Medicine*)

### Poster

[2P]
**Stress**

March 29, 13:00 - 14:20, Poster Room

### [2P-135]

### Prostaglandin $E_2$ induces sustained suppression of noradrenergic neurons in the locus coeruleus to moderate stress response

*Yasutaka Mukai[1,2,3], Tatsuo Okubo[4], Michael Lazarus[5], Daisuke Ono[3], Kenji Tanaka[6], Akihiro Yamanaka[4] ([1]*Hokkaido University,* [2]*Postdoctoral Research Fellow of JSPS,* [3]*Nagoya University,* [4]*The Chinese Institute for Brain Research, Beijing,* [5]*University of Tsukuba,* [6]*Keio University*)

Noradrenergic neurons in the locus coeruleus (LC-NA neurons) are involved in modulation of various behaviors, such as stress responses and sleep/wakefulness. Preceding electrophysiology and transcriptomics studies have identified several substances that modulate LC-NA neuronal activity. However, these studies predominantly focused on major substances with anticipated physiological outcomes, potentially overlooking the significance of less studied substances. In this study, we established a method to screen substances that affect the activity of LC-NA neurons using acute brain slices from transgenic mice expressing calcium indicator, yellow Cameleon-Nano50. We screened 53 substances and found 11 that increased and 13 that decreased intracellular calcium concentration ($[Ca^{2+}]_i$) of LC-NA neurons. Among them, gastrin-releasing peptide, neuromedin U (NMU), and angiotensin II increased $[Ca^{2+}]_i$, while pancreatic polypeptide and prostaglandin $D_2$ decreased $[Ca^{2+}]_i$. Remarkably, these five substances had not been previously reported. This result showed the high sensitivity and potential of our screening method. Furthermore, we observed that 2 min application of prostaglandin $E_2$ ($PGE_2$) induced a sustained decrease of $[Ca^{2+}]_i$ for over an hour via the $EP_3$ receptor (EP3R). To examine the physiological role of $PGE_2$, we generated a conditional knockout (cKO) mouse strain in which the EP3R was exclusively knocked out in noradrenergic neurons. Following 30 min of restraint stress in the latter half of the light period, cKO animals showed prolonged depression-like behavior (immobility) in the tail-suspension test (TST), along with longer wakefulness during the subsequent dark period. Additionally, cKO animals showed a larger calcium signal during TST, as measured by fiber photometric recording of calcium indicator (G-CaMP6) signal in LC-NA neurons. It has been reported that LC-NA neurons are activated by various stressors, and increased LC-NA neuronal activity can be correlated with depression-like states. Furthermore, exposure to stressors induce longer sleep and thereby shorter wakefulness, potentially serving an adaptive function in coping with stressors. Consequently, our findings suggest that stress-induced $PGE_2$ suppresses LC-NA neuronal activity to moderate behavioral responses to stressors.

### [2P-136]

### Alterations in microbiome-gut-brain axis and contribution of microbiota to the brain dysfunction in chronic unpredictable mild stress (CUMS) mice.

*TAOLEI HAN[1], Kenjiro Sato[2], Nobuhiko Kojima[1,2,3] ([1]*Graduate School of Life Sciences, Toyo University,* [2]*Institute of Life Innovation Studies, Toyo University,* [3]*Department of Life Sciences, Toyo University*)

Depression is a complex mental health disorder, and increasing evidence suggests that depressive and anxious behaviors are linked to changes in the composition of gut microbiota. However, the specific pathways and causal relationship between gut microbiome and brain function have not been fully elucidated. In this study, we investigated the interrelationships of the microbiota-brain-gut axis by exposing mice to chronic unpredictable mild stress (CUMS). After assessing depression-like and anxiety-like behaviors in individual animals through behavioral tests, such as open field test and forced swimming test, we comprehensively screened for changes in gene expression in the brain and the composition of intestinal bacteria by genome-wide RNA-seq analysis and meta 16s analysis, respectively, to explore their potential connections. RNA-seq analysis indicated that 480 genes were differentially expressed in the cerebral cortex of CUMS mice (P- Value < 0.05). Pathway enrichment analysis revealed significant down-regulation of the MAPK signaling pathway, which is associated with inflammation and neurogenesis, consistent with the dysregulation of MAP kinase pathway in human depression patients. In mouse cecal contents significant differences in bacterial composition and diversity were found between the CUMS and control groups. Among several bacterial species that altered in their abundance, we noted following two species of *Lactobacillus*; *Lactobacillus murinus* became extinct, while *Lactobacillus reuteri* was robustly increased in the CUMS group. We cultivated and fed these bacteria to healthy mice to explore their potential roles in affecting brain function and behavior. We hypothesized that these intestinal bacterial communities may influence depressive behavior by directly or indirectly modulating signaling pathways such as MAPK signaling pathway in brain cells. and we are currently analyzing gene expression and phosphorylation of ERK and p38 in the cerebral cortex, hippocampus, and prefrontal cortex of mice transplanted either *Lactobacillus murinus* and *reuteri*. These studies will demonstrate the interconnections between microbiota and host physiology, and understanding these mechanisms should provide us to gain insights for new diagnostic and therapeutic strategies for depression.

### [2P-137]

### The role of orexin neurons in the regulation of chronic itch processing

*Asuka Oura[1], Tatsuroh Kaneko[1], Takuro Kanekura[2], Tomoyuki Kuwaki[1], Hideki Kashiwadani[1] ([1]*Department of Physiology, Graduate School of Medical and Dental Sciences, Kagoshima University,* [2]*Department of Dermatology, Kagoshima University Graduate School of Medical and Dental Sciences*)

Chronic itch is linked with elevated stress, anxiety, and other mood-related disorders. These conditions aggravate itch symptoms, establishing a harmful cycle that deteriorates the prognosis of the condition and impairs the quality of life. While most previous studies have concentrated on peripheral and spinal mechanisms of chronic itch, our understanding remains limited regarding the mechanism in the central nervous system.

Previously, we demonstrated that orexin (ORX) producing neurons in the lateral hypothalamus are instrumental in the neural processing of acute itch, as performed through the chloroquine-induced acute pruritus model.

Here, to investigate whether orexin (ORX) neurons also participate in chronic itch processing, we utilized ORX neuron ablation mice (ORX-abl mice) and created a model of contact dermatitis-associated chronic itch by repeatedly applying diphenylcyclopropenone (DCP) to the nape skin of mice.

In the DCP-induced chronic itch model, ORX-abl mice exhibited significantly reduced scratching behavior compared to their wild-type (WT) counterparts during 60 minutes of the observation period. These findings imply that ORX neurons also have a crucial role in chronic itch processing, not just in acute pruritus as described in our previous report.

We also measured transepidermal water loss (TEWL), a well-established metric for assessing skin barrier function. Notably, ORX-abl mice demonstrated significantly lower TEWL compared to the WT control group. This suggests that inhibiting ORX neurons may mitigate scratching behavior and consequently prevent skin barrier dysfunction induced by chronic itch.

Thus, our findings indicate that suppressing ORX neurons may reduce scratching behavior and subsequently prevent skin barrier dysfunction caused by chronic itch. This highlights the potential therapeutic relevance of targeting ORX neurons in managing chronic itch conditions such as contact dermatitis and atopic dermatitis.

### [2P-138]

### Changes in sucrose preference in mice undergoing chronic corticosterone administration and liquid restriction

*Yu Wakaumi[1], Kensaku Nomoto[1], Kenji Kansaku[1] ([1]*Department of Physiology, Dokkyo Medical University School of Medicine*)

### [2P-139]

### pH-inducible transcription factors controlling pH stress adaptation

*Haruki Omatsu[1], Tado Yuki[1], Kagami Ryoko[1], Mori Yasuo[1], Takahashi Nobuaki[1]
([1]*Department of Synthetic Chemistry and Biological Chemistry, Graduate School of Engineering, Kyoto University*)

### Poster

### [2P]
### Pathophysiology

March 29, 13:00 - 14:20, Poster Room

### [2P-140]

**TRPV4 expression in atopic dermatitis and its response to osmotic stress**

*Atsuko Kamo[1], Mitsutoshi Tominaga[2], Kenji Takamori[2,3] ([1]*Faculty of Health Care and Nursing, Juntendo University, [2]Juntendo Itch Research Center (JIRC), Institute for Environmental and Gender-Specific Medicine, Juntendo University Graduate School of Medicine, [3]Department of Dermatology, Juntendo University Urayasu Hospital*)

The skin serves as the interface between the body's internal environment and its external surroundings, playing a vital role in maintaining homeostasis by regulating water movement. Conditions like dry skin and pruritic dermatological diseases such as atopic dermatitis (AD), are often associated with impaired skin barrier function and itch. Increased transepidermal water evaporation due to impaired skin barrier function can alter the epidermal environment, including osmolality, which may contribute to the sensation of itch inducing scratching behavior that further changes the cutaneous environment. Transient receptor potential vanilloid 4 (TRPV4) is expressed in epidermal keratinocytes and is activated by factors such as temperature (27~35℃) and hypoosmolality. A recent study demonstrated that TRPV4 expression increased in dry skin model mice, and its antagonists inhibited epidermal thickness and reduced scratching behavior. However, the detailed mechanisms underlying pruritus and the involvement of TRPV4 in AD remain unclear.
In this study, we examined TRPV4 expression in AD and the effects of extracellular osmolality on TRPV4 expression. Immunofluorescence analysis was performed on the skin of six healthy participants as controls and 10 AD patients. Some AD patients exhibited intensive TRPV4 expression compared to healthy controls. Semiquantitative analysis revealed a significant increase in TRPV4 fluorescence intensity in AD patients compared to healthy participants, although it did not correlate with itch visual analog scale scores. Next, normal human epidermal keratinocytes were cultured in a medium containing 1.4 mM calcium to induce differentiation, and osmotic stimuli (200 mOsm/kg, 300 mOsm/kg, and 400 mOsm/kg) were applied. Real-time reverse transcription-polymerase chain reaction assays showed that TRPV4 and filaggrin mRNAs increased in normal human epidermal keratinocytes cultured in a hyperosmotic medium. In summary, the epidermal osmotic environment in AD patients may fluctuate, which could affect TRPV4 expression and skin barrier function.

### [2P-141]

**Role of neutrophil extracellular traps in pathological progression after ischemic stroke**

*Kana Sugimoto[1], Chihpin Yang[1], Ryuichi Katada[1], Hiroshi Matsumoto[1] ([1]*Department of Legal Medicine, Osaka University Graduate School of Medicine*)

Excessively activated neutrophils release extracellular reticular structures called neutrophil extracellular traps (NETs). NETs are composed mainly of extracellular decondensed chromatin affixed with histones, neutrophil elastase (NE), and myeloperoxidase (MPO), which can trap and destroy infectious microorganisms. However, an uncontrolled or excessive NETs formation causes cellular damages and adjacent tissue injuries. NETs have been reported to be present in the brain of stroke patients, but the biological function and underlying mechanism of NETs in pathological progression after ischemic stroke remain poorly understood. In this study, we examined the effects of NETs on post-infarction edema formation using transient middle cerebral artery occlusion (tMCAO) model rats, rat neutrophil, and astrocyte primary cultures. To examine the spatiotemporal profile of neutrophil infiltration in the brain after tMCAO, Ly6g-positive cells were observed in leptomeninges, cerebral cortices, and striata in the ipsilateral hemisphere of the ischemic brain. Infiltration of those cells occurred in the leptomeninges after 6 hours and was then also observed in the cortex and striatum after 24 hours of tMCAO. In addition, several NE and MPO-positive cells were also recognized, indicating the formation of NETs. Notably, no Ly6g-positive cells were detected in contralateral hemispheres. Furthermore, there was a positive correlation between the number of neutrophil infiltrates and the level of brain edema. To determine the relationship of activated neutrophils to brain edema, isolated neutrophils activated with LPS or HMGB1 were co-cultured with astrocytes. The results showed an increase in AQP4 expression in astrocytes. These findings suggest that activated neutrophils induce astrocytic AQP4 expression in the peri-infarct and ischemic core tissues, thereby exacerbating brain edema. Modulation of neutrophil activities, therefore, represents a promising therapeutic strategy for stroke therapy. In conclusion, NETs were observed in the peri-infarct and ischemic core tissues of rat stroke brain, which activates AQP4 expression in astrocytes and then exacerbates brain edema.

### [2P-142]

**Lithium treatment rescued mania-like behavior in a synaptically modified mouse model of bipolar disorder.**

*Wataru Ota[1], Takuya Takahashi[1] ([1]*Dept. Physiol., Yokohama City Univ. Sch. of Med.*)

Bipolar disorder (BD) is a mental health condition that causes large mood swings, including mania and depression. However, the biological basis, such as synaptic and circuit alterations, underlying BD remains unclear.
The excitatory glutamate α-amino-3-hydroxy-5-methyl-4-isoxazole propionic acid receptor (AMPAR) is a fundamental component of neurotransmission. We have developed a positron emission tomography (PET) tracer for AMPARs, [$^{11}$C]K-2, which is the first technology used to visualize and quantify the density of AMPARs in the living human brain (Miyazaki *et al.*, *Nat. Med.*, 2020). Importantly, PET imaging with [$^{11}$C]K-2 (AMPAR PET imaging) depicts cell-surface AMPARs, a functionally crucial fraction of AMPARs (Arisawa *et al.*, *Neurosci. Res.*, 2021). This tracer exhibited the decrease of AMPARs in the cerebellum of bipolar patients was significantly correlated with the symptomatology score of manic state (Young Mania Rating Scale; YMRS). Based on this clinical data, we have generated a novel synaptically modified mouse model of BD (manic state), *i.e.*, knockdown of AMPAR expression in the mouse cerebellum using short hairpin RNA targeting to AMPAR. This model (Cb-shAMPAR mouse) shows several manic phenotypes, such as reduced immobility time in forced swim and tail suspension tests, hedonia-like high sucrose preference, and circadian disruption, as we reported at the last annual meeting of the Physiological Society of Japan. In this presentation, we will show some additional data that reinforce the validity of this model.
Lithium is a classical anti-manic state drug (mood stabilizer) used in patients with BD. We administered lithium to Cb-shAMPAR mice (LiCl in drinking water at 300 mg/L). Although the administration of lithium did not affect the locomotor activity or motor coordination, lithium treatment prolonged the immobility time of the Cb-shAMPAR mice to the normal range (same level as non-treated control mice) in the forced swim and tail suspension tests. Cb-shAMPAR mice treated with water (another control) showed mania-like behavior, as observed in the previous experiment. Thus, administration of lithium rescued mania-like behavior in animals with reduced cerebellar AMPAR expression, as observed in patients with bipolar mania. These results suggest that our animal model of the manic state shares biological mechanisms that have been detected in human patients with BD and satisfies the three key validities required for an ideal model of psychiatric disorders: face validity, construct validity, and predictive validity. Taken together, our approach using AMPAR PET imaging in combination with animal experiments is expected to lead to further understanding of the neurobiology and synaptic physiology of BD and other psychiatric disorders.

### [2P-143]

### Relation between severity of headache and amygdala volume in migraine patients.

*Shota Kosuge[1,2], Yuri Masaoka[1], Hideyo Kasai[2], Motoyasu Honma[1], Miku Kosuge[1,3], Daiki Shoji[1,2], Kouzou Murakami[4], Takaaki Naito[5], Misako Matsui[1], Hidetomo Murakami[2], Masahiko Izumizaki[1] (*[1]1)Department of Physiology, Showa University School of Medicine, [2]2)Department of Neurology, Showa University School of Medicine, [3]3)Department of Respiratory Medicine, Showa University Fujigaoka Hospital, [4]4)Division of Radiation Oncology, Department of Radiology, Showa University School of Medicine, [5]5)Department of Radiological Technology, Showa University Hospital*)

Migraine (MG) is a complex disorder of the brain that involves multi-sensory disturbances. Symptoms of MG include the primary headache accompanied with disordered perceptions of light, sound and smell. Patients often complain of hypersensitivities toward normal light or sound and these induce headache as well as nausea. In this study, we measured the structural volume of brain regions in patients with MG, and investigated a relationship between structural volumes and headache severity. 33 migraine patients (aged 15-64 years) and 27 healthy subjects (aged 19-60 years) measured whole brain T1-weighted magnetic resonance imaging (MRI) and measured brain volume with Freesurfer software. MG and healthy subjects (controls) were measured Migraine Disability Assessment Questionnaire (MIDAS) to assess headache severity. Analysis of covariance showed decreased volumes in the bilateral amygdala and globus pallidus, in MG compared with those of controls. Correlation analysis showed a negative correlation between the volume of the right amygdala and MIDAS scores in MG, indicating that individuals with smaller right amygdala volume have more severe migraine symptoms. The amygdala is responsible for emotions of anxiety, and pain sensation. We assume that our findings will not only advance our understanding of migraine pathophysiology but also pave the way for potential therapeutic interventions targeting the amygdala and its role in emotion regulation for enhanced migraine management.

### [2P-144]

### Paxillin tyrosine 31 phosphorylation regulates breast cancer cell migration and invasion

*Ying Zhang[1], Huanyu Zhen[2], Ming Xu[2], Noriko Maeda[2], Ryouichi Tsunedomi[2], Hiroko Kishi[3], Hiroaki Nagano[2], Sei Kobayashi[1] (*[1]Yamaguchi University, [2]Yamaguchi University, Department of Gastroenterological, Breast and Endocrine Surgery, Yamaguchi University, [3]Shimane University*)

Metastasis remains the primary cause of death in breast cancer patients. Elevated levels of paxillin expression have been observed in various types of cancer, with its tyrosine phosphorylation playing a pivotal role in driving cancer cell migration. However, the specific impact of distinct tyrosine phosphorylation events of paxillin in the progression of breast cancer remains to be fully understood. In this study, we discovered that overexpression of paxillin in breast cancer tissue is associated with a poorer prognosis for patients. Knocking down paxillin inhibited the migration and invasion of breast cancer cells. Additionally, the phosphorylation of paxillin at tyrosine residue 31 (Tyr31) showed a significant increase during TGF-β1-induced migration and invasion of breast cancer cells. Inhibiting Fyn activity or silencing Fyn resulted in decreased paxillin Tyr31 phosphorylation. Both wild-type and constitutively active Fyn directly phosphorylated paxillin Tyr31 in an in vitro system, indicating that Fyn is a direct mediator of paxillin Tyr31 phosphorylation. Furthermore, the non-phosphorylatable mutant of paxillin at Tyr31 led to a reduction in actin stress fiber formation, migration, and invasion of breast cancer cells. Collectively, our results provide direct evidence that Fyn-mediated paxillin Tyr31 phosphorylation is essential for breast cancer migration and invasion. This suggests that targeting paxillin Tyr31 phosphorylation could be a potential therapeutic strategy for mitigating breast cancer metastasis.

### [2P-145]

### Evaluation of Damage Associated Molecular Patterns (DAMPs) in a Zebrafish model of Heart Failure

*Phurpa Phurpa[1], Ryohei Umeda[1,2], Schinichiro Kume[3], Magdeline Elizabeth Carrasco Apolinario[1], Menting Shan[1], Kenshiro Shikano[1], Hitoshi Teranishi[1], Takatoshi Hikida[4], Yulong Li[5], Reiko Hanada[1] (*[1]Department of Neurophysiology, Faculty of Medicine, Oita University, [2]Department of Advanced Medical Sciences, Faculty of Medicine, Oita University, [3]Department of Cardiovascular Sciences, Faculty of Medicine, Oita University, [4]Laboratory for Advanced Brain Functions, Institute for Protein Research, Osaka University, [5]State Key Laboratory of Membrane Biology, Peking University School of Life Sciences*)

**Background**
Heart failure (HF) has poor prognosis due to its therapeutic challenges. Thus, clear understanding of its etiopathophysiology beyond what is known till date remains to be very crucial to enhance HF management. HF is associated with sterile inflammation caused by DAMPs. Extracellular Adenosine triphosphate (eATP) and adenosine (eADO) are two of the main DAMPs with multiple roles in HF. However, in-vivo ATP/ADO dynamics in heart failure (and its different subgroups) have not been studied till date. Therefore, this study will be assessing the eATP/eADO in live zebrafish (ZF) HF models with different underlying etiopathogenesis to provide insight on the link between purinergic signals (ATP/ADO) and HF. Thereby, enabling development of drugs that could block purinergic signals.
**Methods**
This study comprises the establishment of drug induced HF models in ZF and transgenic (Tg) ZF expressing fluorescent ATP/ADO. First of all, zebrafish models of HF have been established. Terfenadine and adrenaline have been tested to induce HF in wild type (WT) and cmlc2: eGFP ZF via atrioventricular block and b-receptor desensitization, respectively. HF was evaluated by assessing cardiac functions: heart rate (HR), fractional shortening, ejection fraction, stroke volume, cardiac output (CO), and end-diastolic volume (EDV). Simultaneously, a plasmid containing Tol2-cmlc2-$GRAB_{ATP}$/$GRAB_{ADO}$ fused with eGFP was developed with the subsequent microinjection of this plasmid with Tol2mRNA into a single cell ZF egg. $GRAB_{ATP}$/$GRAB_{ADO}$ is expected to fluoresce in presence of eATP/eADO in the heart. The intensity of fluorescence is directly proportional to the quantity of eATP/eADO. The established $GRAB_{ATP}$/$GRAB_{ADO}$ will be treated with terfenadine, adrenaline and doxorubicin to induce HF - simulating, dilated cardiomyopathy, pressure overload and oxidative stress associated HF, respectively, followed by assessment of eATP/eADO in HF compared to those without HF. **Results**
WT and Tg ZF (Cmlc2-eGFP) treated with terfenadine showed dilatation of ventricle and atrium with pericardial edema. HR was significantly lower in terfenadine and adrenaline treated ZF compared to control. In addition, compared to control, CO was significantly lower and EDV was significantly higher in terfenadine treated ZF.

### Poster

### [2P]
### Drug Action, Pharmacology

March 29, 13:00 - 14:20, Poster Room

### [2P-146]

#### Anti-stress effect of Hangekobokuto - Study of stress model mice

*Emi Nakamura-Maruyama[1], Naoyuki Himi[1], Takehiro Nakamura[1] ([1]*Department of Physiology2, Kawasaki Medical School*)

Purpose: Hangekobokuto is a Kampo medicine known to be effective in nervous gastritis and improving the experience feeling of a foreign body in the throat and esophagus caused by stress. In recent years, its therapeutic effect on depression-like symptoms has attracted attention, but its mechanism of action is unknown. In this study, we first investigated the anti-stress effects of Hangekobokuto using stress model mice.
Methods: C57BL/6J mice were subjected to water immersion with restraint stress (3-7 h/day, 15 consecutive days) to create a model mouse. Hangekobokuto (2.0 mg/ml, original powder provided by TSUMURA & CO.) was administered orally using a measuring water bottle (4-6 ml/day). Two weeks after completing the model, depression-like symptoms were evaluated behaviorally (Novelty suppressed feeding test, Locomotor activity, Forced swimming test) and histologically (Neurogenesis in hippocampal dentate gyrus). Depression-like symptoms were also compared between the group that was treated with Hangekobokuto before the model was created and the group that was treated for two weeks after the model was completed.
Results: Compared to stress and distilled water group, stress and Hangekobokuto group showed improvement in behavioral tests, and a reduction in stress-induced depressive-like symptoms. Hangekobokuto promoted neurogenesis in the hippocampal dentate gyrus in both normal and stress groups. The effect of improving depression-like symptoms was lower in the group administered after the model was completed than in the group administered before the depression-like model was created. There was also no difference in neurogenesis.
Conclusion: The promotion of neurogenesis by Hangekobokuto may be involved in the improvement of depression-like symptoms. The results also suggest that Hangekobokuto may be a prophylactic agent for depression when used before stress is applied.

### [2P-147]

#### Novel Approaches to Alleviate Heart Failure: A Focus on Moku-boi-to and Angiotensin II Receptor Blockade

*Fumiha Abe[1], Hideaki Tagashira[1], Tomohiro Numata[1] ([1]*Akita Univ.*)

Heart failure (HF) is a critical health concern in Japan, ranking among the top three leading causes of mortality. The current therapeutic strategies primarily focus on mitigating cardiac hypertrophy, yet their efficacy remains limited. Consequently, there is an urgent imperative to develop more potent therapeutic agents capable of enhancing patients' quality of life while simultaneously alleviating the substantial medical cost burden associated with HF. Recent attention has turned towards incorporating Japanese herbal medicine alongside existing treatments, although the mechanism of action is largely unknown and clinical evidence is lacking, so the medical community is cautious about this.This study explored six Kampo medicines with known cardiovascular benefits, utilizing an angiotensin II (AngII)-induced neonatal rat ventricular myocyte (NRVM) hypertrophy model. The outcomes unveiled Moku-boi-to (MBT) as a noteworthy candidate for suppressing the hypertrophic response induced by AngII.AngII elicited an array of adverse effects in NRVM, including increased cell volume increases, reduced cell viability, diminished ATP production, and elevated levels of reactive oxygen species (ROS). MBT treatment demonstrated a remarkable capacity to ameliorate these symptoms. Furthermore, it effectively addressed the issues of mitochondrial fragmentation and heightened intracellular $Ca^{2+}$ levels linked to cardiomyocyte hypertrophy.Regarding the mechanism of action of MBT, when combined with losartan, an angiotensin II type 1 ($AT_1$) receptor antagonist, the efficacy of losartan and MBT in alleviating AngII-induced myocardial hypertrophy was demonstrated by a combination index > 1., the $AT_1$ receptor was found to be the target. Finally, MBT has shown efficacy in attenuating isoproterenol-induced cardiac hypertrophy and associated dysfunction, correlating with preventing cardiac fibrosis. Collectively, it underscores MBT's utility in addressing myocardial hypertrophy and heart failure. Its mechanism of action involves the enhancement of mitochondrial function through $AT_1$ receptor blockade, shedding light on a promising avenue for future therapeutic development.

### [2P-148]

#### Long-term oral administration of L-ornithine alleviates allergic rhinitis symptoms in mice

Madoka Tsujimoto[1], Maki Inokuchi[1], *Yasushi Hayashi[1] ([1]*Department of Food and Human Nutrition, Faculty of Human Life Sciences, Notre Dame Seishin University*)

**Introduction**: We have demonstrated that long-term oral administration of L-ornithine effectively reduces psychological stress in mice. There is a well-established association between stress exacerbation and allergic diseases, and reducing psychological stress alleviates the symptoms of allergic diseases. Based on the correlation between psychological stress and allergic diseases, we assessed the effectiveness of ornithine to treat the symptoms of allergic diseases. We used an ovalbumin (OVA)-induced allergic rhinitis mouse model to evaluate the preventive effect of long-term ornithine administration on rhinitis symptoms. In addition, we analyzed the mechanism underlying the anti-rhinitis effects of ornithine, with a particular focus on helper T (Th) cells in the mouse spleen.
**Methods**: Female BALB/c mice aged 7 weeks were used in this study. Rhinitis symptoms were induced through repeated nasal administration of OVA. L-ornithine was dissolved in tap water and administered orally via a water bottle for 5 weeks. We monitored rhinitis symptoms weekly and tracked the concentration of OVA-specific IgE antibody (OVA-IgE) in plasma collected from the tail vein over time. The mice were deeply anesthetized, and their spleens were removed. Subsequently, mRNA was extracted from the spleen, and real-time PCR was used to analyze the master transcription factor and cytokine genes of Th cells.
**Results**: Repeated OVA sensitization resulted in an exacerbation of rhinitis symptoms and a significant increase in OVA-IgE levels. Conversely, L-ornithine led to a reduction in sneezing and nasal rubbing at 3 weeks post-administration, emphasizing its preventive effect against rhinitis. Moreover, the increase in OVA-IgE was significantly suppressed. PCR analysis revealed that ornithine administration suppressed the activity of Th2 and follicular Th cells, whereas it enhanced the activity of Th1 and regulatory T cells.
**Conclusions**: These findings emphasize that ornithine can alleviate rhinitis by restoring the composition ratio of Th cell subsets. (COI:No)

### Poster

### [2P]
### Study Methodology

March 29, 13:00 - 14:20, Poster Room

### [2P-149]

**Streamlining Vascular and Lymphatic Staining: A Rapid and Cost-Efficient Protocol for Large Tissue Sections**

*Makoto Matsuyama[1], Sebastian Sjoeqvist[1,3], Takahiro Iwamiya[1,2,3] ([1]*Metcela Inc.*, [2]*Institute for Advanced Biosciences, Keio University,* [3]*Nagoya University Graduate School of Medicine*)

Histological evaluation of vascular and lymphatic vessels traditionally involves labor-intensive techniques that necessitate dozens of sectioning and complex software reconstruction, often spanning months of effort. Here, we introduce a rapid protocol that streamlines this process, enabling the comprehensive examination of thick tissue samples, including centimeter-sized organs or multi-hundred-micrometer sections.
This novel method employs conventional fluorescence microscopy setups and standard antibodies to visualize vascular and lymphatic vessel branches, eliminating the need for costly specialized equipment or reagents. The protocol hinges on a thick section stained with fluorescent probes, which immediately reveals the vascular vessel branching, condensing the entire workflow of sectioning, staining, imaging, and reconstruction into a one-week timeframe. Moreover, the technique minimizes tissue damage, rendering it adaptable to challenging targets like membrane proteins.
Our approach is both user-friendly and compatible with any lab setups, making it accessible to researchers with few antibody options against their targets or limited access to expensive optical equipment. We demonstrate the protocol's efficacy through antibody-free labeling of vascular vessels in whole rat hearts, and antibody staining of lymphatic vessels in rat lungs, showcasing its versatility in advancing histological studies.
COI: No

### [2P-150]

**A non-invasive gene transfection technology using transcranial focused ultrasound irradiation**

*Toshimasa Mimura[1], Michele Chan[1], Masabumi Minami[1], Nobuki Kudo[1,2], Yuichi Takeuchi[1] ([1]*Department of Biopharmaceutical Sciences and Pharmacy, Faculty of Pharmaceutical Sciences, Hokkaido University,* [2]*Department of Bioengineering and Bioinformatics, Faculty of Information Science and Technology, Hokkaido University*)

The blood-brain barrier (BBB) regulates entries of molecules except those essential for normal brain functions; this limitation is a significant obstacle for delivery of gene and water-soluble drugs to the brain. Here we developed a non-invasive and brain region-specific gene transfection technology with microbubble-mediated temporal opening of BBB followed by transient increase of plasma membrane permeability via sonoporation with transcranial ultrasound irradiation in adult male mice. The right hemisphere of the brain of each anesthetized mouse was irradiated by 0.894 MHz ultrasound using a non-focused transducer for three minutes following the intravenous administration of microbubble and pCMV-GFP plasmid solutions. After recovery, the animals were normally reared for seven days and sacrificed by transcardial perfusion. The brain was removed, sectioned, and immunohistochemically stained with an anti-NeuN, GFAP, or Iba1 antibody with nuclear counter staining. The sections were then observed with a laser-scanning confocal microscope. As a result, approximately 80% of GFP expressing cells co-localized with NeuN-positive cells whereas those co-localized with GFAP or Iba1 signals were minor. Because the GFP signals were observed to the superficial brain regions (e.g., cerebral cortex and hippocampus), a focused ultrasound transducer with higher frequency was implemented to achieve effective BBB-opening of deeper brain regions located at the focus. Spherical 5 and 10 MHz transducers successfully achieved a miniature BBB-opening in approximately <1 $mm^3$ volume in the striatum with the same acoustic pressure. Our transcranial sonoporation technology allows a non-invasive and brain region specific gene or drug delivery with transcranial focused ultrasound irradiation.

### Poster Presentation

Day 3
(March 30, 13:00 - 14:20)

[3P] Neurophysiology, Neuronal cell biology - Plasticity
[3P] Neurophysiology, Neuronal cell biology - Neural network
[3P] Neurophysiology, Neuronal cell biology - Neurons, Synapses
[3P] Neurophysiology, Neuronal cell biology - Sensory function, Sensory organ
[3P] Molecular physiology, Cell physiology - Ion channels, Receptors
[3P] Embryology, Regenerative Medicine, Development, Growth, Aging
[3P] Muscle
[3P] Oral physiology
[3P] Circulation
[3P] Respiration
[3P] Physical fitness and sports medicine
[3P] Nutritional and metabolic physiology, Thermoregulation
[3P] Behavior, Biological rhythm, Sleep
[3P] Stress
[3P] Pathophysiology
[3P] Drug Action, Pharmacology
[3P] Medical education, Medical histology
[3P] Study Methodology
[3P] Others

### Poster

### [3P]
### Neurophysiology, Neuronal cell biology
**Plasticity**

March 30, 13:00 - 14:20, Poster Room

### [3P-001]
### Length abnormalities of the axon initial segment in mouse models of attention-deficit hyperactivity disorder

*Takeshi Yoshimura[1], Taiichi Katayama[1] ([1]*United Graduate School of Child Development, Osaka University*)

The axon initial segment (AIS) is a structural neuronal compartment of the proximal axon that plays key roles in sodium channel clustering, action potential initiation, and signal propagation for neuronal outputs. Mutations in constitutive genes of the AIS, such as *ANK3*, have been identified in patients with neurodevelopmental disorders. Nevertheless, morphological changes in the AIS in neurodevelopmental disorders have not been characterized. In this study, we investigated the AIS length in animal models of attention-deficit hyperactivity disorder (ADHD). We observed abnormalities in AIS length in ADHD model mice and rats. These results demonstrate that AIS length is altered in specific brain regions in ADHD rodent models, and AIS abnormalities may be conserved across species. In addition, we found that repeated treatments of atomoxetine, an ADHD drug, significantly improved AIS abnormality along with hyperactivity in ADHD model mice. Our findings provide novel insight into the potential contribution of the AIS to the pathophysiology and pathogenesis of neurodevelopmental disorders.

### [3P-002]
### Plant-derived catechin modulates olfactory nerve activity in the land slug *Limax valentianus*

*Yoshimasa Komatsuzaki[1], Aya Nagata[1], Ken Lukowiak[2] ([1]*CST, Nihon Univ.*, [2]*Hotchkiss Inst., Cumm. Sch. Med., Univ. of Calgary*)

Epicatechin (EpiC), a flavonoid abundant in green tea, improves cognitive function in various animals, including invertebrates. Our previous studies have shown that EpiC enhances memory formation through two types of association learning in the pond snail. However, it is unclear how EpiC affects intercellular signaling mechanisms that play an important role in memory formation. In this study, we focused on the neuronal synaptic connections from the olfactory organ to the olfactory center in the terrestrial slug *Limax valentianus*, an animal model for learning and memory with a highly developed olfactory system. The procerebral (PC) lobe, an olfactory center of the slug, receives olfactory input from the inferior and superior tentacular noses, and is represented as spatial and temporal activity patterns observed as oscillatory local field potentials of approximately 0.7 Hz. Here, we investigate the effects of EpiC on the synaptic plasticity between the olfactory organ and the PC lobe in the land slug. To measure the excitatory postsynaptic potential (EPSP) around the parietal-ventral synapse in the PC lobe, a recording electrode was lightly inserted into the dorsal surface of the PC lobe. The superior tentacular nerve was stimulated with a suction electrode. The stimulation intensity of the tentacular nerve was determined to be about half of the maximum value of the synaptic response (amplitude of the EPSP). The results showed that the amplitude of the EPSPs in the PC increased within ten minutes after administration of EpiC. High-frequency stimulation of the olfactory nerve (100 Hz, 1 s, 4 trains) induced long-term depression in the presence of EpiC but did not in the absence of EpiC. Furthermore, the administration of EpiC reduced the frequency of spontaneous oscillatory activity in the PC lobes of slugs. These results suggest that EpiC directly affects a synaptic connection between the olfactory nerve and the PC lobe to cause memory enhancement. The authors declare no conflicts of interest related to this abstract.

### [3P-003]
### Supra-spinal plasticity of the corticospinal projections after motor recovery from spinal cord injury in macaque monkeys

*Satoko Ueno[1,2], Reona Yamaguchi[2], Kaoru Isa[1], Toshinari Kawasaki[1], Masahiro Mitsuhashi[1], Tadashi Isa[1,2,3] ([1]*Department of Neuroscience, Grad Sch of Med., Kyoto Univ.*, [2]*Institute for the Advanced Study of Human Biology (WPI-ASHBi), Kyoto Univ.*, [3]*Human Brain Research Center, Grad Sch of Med., Kyoto Univ.*)

Lesion to the corticospinal tract (CST) is one of the critical components caused by spinal cord injury (SCI) and recovery of motor functions would depend on how to rebuild similar neuronal circuits as before the injury. Our recent studies showed that the macaque monkeys with subhemisection of the middle cervical spinal cord (C4/C5) appreciably recovered their hand movements following intensive behavioral tests and repeated cortical electrical stimulation. In this model, we found that the CST of lesion-affected side originating from the contralesional motor cortex re-routed through the long distance of the spinal cord and terminated in the ipsilesional gray matter caudal to the lesion, where the motoneurons innervating the hand muscles were located. Here, we aimed at further exploring the projection of CST axons at the supraspinal level in this recovery model. We investigated the axonal distribution originating from the ipsilesional and contralesional primary motor cortex (M1) labeled by anterograde viral tracers injected into M1 on each hemisphere. In this study, we focused on the projection to the putamen. Compared with the unaffected pathway which projects mainly to the putamen on the same side as the injection site, the labeled axons of the lesion-affected pathway to the putamen on the opposite side increased. Because the putamen is known to receive the inputs from the motor cortex and send the feedback signals to the same hemisphere through the cortico-basal ganglia loop, the present result might suggest that the putamen on the ipsilesional side receives the input from the contralesional M1 and activate the ipsilesional M1 through the cortico-basal ganglia on the ipsilesional side. These results suggest that the plasticity of the motor control system occurred in the cortico-basal ganglia as well as the spinal cord and might contribute to the activation of the ipsilesional motor cortex in this model. Such plastic change of CST projections to a wide variety of brain areas should compose critical components of motor functional recovery after SCI.

### [3P-004]

### Gadolinium induces mGluR-, eCB-, and P2R-dependent long-term depression of evoked synaptic transmission

Odgerel Zorigt[1], *Hiroki Yasuda[1,2], Takahito Nakajima[1], Yoshito Tsushima[1] ([1]*Gunma University,* [2]*Saga University*)

Gadolinium-based contrast agents (GBCAs) are commonly used in magnetic resonance imaging (MRI) examination. GBCAs remain in some brain regions after MRI examinations and they could damage brain tissues. Here, we report that high concentration of gadolinium induces metabotropic glutamate receptor (mGluR)-, endocannabinoid (eCB)-, and purinergic receptor-depepndent long-term depression (LTD) of synaptic transmission in the CA1 region of the mouse hippocampus. A low concentration of gadolinium (100 μM) potentiated field excitatory postsynaptic potentials (fEPSPs) with a decrease in paired-pulse ratio (PPR), indicating that low concentration of gadolinium enhances glutamate release at presynaptic sites. On the other hand, high concentrations of gadolinium (1000 μM) induced group 1 mGluR-, eCB, and P2 receptor (P2R)-dependent presynaptically-expressed LTD and P2R-dependent postsynaptically-expressed LTD. DHPG (30 μM), a group 1 mGluR agonist, induced LTD with an increase in paired-pulse ratio (PPR). AM251, a cannabinoid receptor 1 (CB1R) antagonist, and PPADS (50 μM), a P2R antagonist, inhibited DHPG-induced LTD, indicating that mGluR-dependent DHPG-induced LTD is also CB1R- and P2R-dependent. WIN55212-2 (WIN), a CBR agonist, induced LTD wit an increase in PPR and WIN-induced LTD was inhibited by PPADS. These results suggest that P2R is required for CB1R to depress evoked synaptic transmission.

### [3P-005]

### Learning-induced $GABA_A$ receptor phosphorylation and the blockade in the hippocampal CA1 neurons: quantification of membrane receptors using super-resolution microscopy.

*Yuzuki Teramoto[1], Yukihiko Osaki[1], Yuya Sakimoto[2], Dai Mitsushima[2], Yuheng Yang[2] ([1]*Yamaguchi University Faculty of Medicine and Health Sciences,* [2]*Department of Physiology, Yamaguchi University Graduate School of Medicine*)

### [3P-006]

### Expression pattern of immediate early genes observed on re-exposure to stimuli after memory consolidation in filial imprinting in the domestic chick

*Rie Suge[1] ([1]*Saitama Medical Univ.*)

Filial imprinting is a type of early learning, whereby social preference becomes restricted to an object following exposure to that object. In domestic chicks, chicks learn characteristics of the object (the imprinting stimulus) and its preference for the object is maintained by consolidation process with neural plasticity during a perinatal sensitive period. Imprinting leads to an increase in the proportion of neurons in the intermediate and medical mesopallium (IMM) that are selectively responsive to a visual imprinting stimulus. This increase is dependent on undisturbed sleep 5-12 h after the exposure. In early stage and sleep period of this consolidation process, transient expression of Fos-like immunoreactivity was observed in the IMM. During the sensitive period, chicks can learn characteristics of stimuli other than the imprinting stimulus. Effect of new stimulus and re-exposure of learned stimulus on the neural activity in the IMM was investigated using immunocytochemistry. Dark-reared chicks (24 h old) were divided into five groups, 1) RR: Day 1 exposed to Red box, Day 2 exposed to Red box again, 2)RD: Day 1 exposed to Red box, Day 2 kept in the dark, 3) DR: Day 1 kept in the dark, Day 2 exposed to Red box, 4)RB: Day1 exposed to Red box, Day 2 exposed to Blue cylinder (novel stimulus), 5) DD: Day1, 2 kept in the dark. Subjects were imprinted in running wheels by exposure to a movie of rotated and moving red box or striped blue cylinder (displayed on liquid crystal display) for 30 min. Expression pattern of immediate early genes, *c-fos*, *Ark* and *egr-1* in the IMM were examined 2 h after the Day 2 exposure with immunocytochemistry. Exposure of Day 1 make difference of the expression patterns after Day 2. This work was supported by JSPS KAKENHI Grant Number 19K03369 and 23K03016.

### Poster

[3P]
**Neurophysiology, Neuronal cell biology**
**Neural network**

March 30, 13:00 - 14:20, Poster Room

### [3P-007]

### Automatic extraction of hippocampal ripples using cluster analysis

*Yuki Kawauchi[1], Junko Ishikawa[2], Dai Mitsushima[2], Jun Nishii[1] ([1]*Biological Cybernetics Laboratory, Yamaguchi University Graduate School of Sciences and Technology for Innovation,* [2]*Department of Physiology, Yamaguchi University Graduate School of Medicine*)

In the hippocampus, neural activity called ripple waves are frequently observed during and after novel experiences and are thought to represent memory information. In this study, we developed an algorithm to automatically extract ripple waves from recorded neural signals, to analyze ripple waves more efficiently. In the proposed method, the power spectrum of neural signals is clustered into three classes by the *k-means* method. The data in the cluster with the smallest average power of the cluster data is classified as the base waves, and the remaining clusters are classified as ripple waves. The classified data are then used as teacher data to train a Support Vector Machine (SVM), and this SVM is used to classify novel neural signals. To evaluate the performance of the algorithm, we used neural signals recorded multiple-unit firing activity of CA1 neurons at a sampling frequency of 25,000 Hz from 10 male rats. Under free-moving condition, we initiated the recordings in their home cage and exposed to four novel episodic experiences for 10 min. Recording was then continued in the home cage for 40 min. From the data for each individual and each experience, approximately 90 ripple and base waves were obtained by the visual judgment of an expert based on the waveform. They were split with a time window of 20 ms to obtain a power spectrum of approximately 500 waveforms. Forty datasets were prepared by the above preprocessing. A frequency band of 100 to 2,000 Hz was used for the clustering. Clustering results for each individual and each experience dataset showed that the classification performance of the supervised data was 92% Accuracy, 92% Precision, 88% Recall, and 88% F1. Next, the classification performance of SVM on novel data was evaluated by cross-validation. The SVM was trained in two ways: using the clustering results as a teacher signal and using signals visually classified by an expert. No significant differences were found in any of the four performance measures. Furthermore, the classification performance of these SVM was almost the same as that obtained using clustering alone. Classification by clustering for long-time neural signals requires high computational cost. The proposed method, which uses the results of clustering a subset of neural signals as the SVM's teacher data, solves this problem.

### [3P-008]

### Mu-opioid receptor-dependent modulatory mechanisms of projections from the medial prefrontal cortex to ventrolateral periaqueductal gray

*Yuka Nakaya[1], Masayuki Kobayashi[1], Hiroyuki Neyama[2], Shigeki Kato[3], Kazuto Kobayashi[3], Yi Long Cui[2] ([1]*Department of Pharmacology Nihon University School of Dentistry,* [2]*Laboratory for Biofunction Dynamics Imaging, RIKEN Center for Biosystems Dynamics Research,* [3]*3 Department of Molecular Genetics, Fukushima Medical University Institute of Biomedical Sciences*)

The medial prefrontal cortex (mPFC) plays a major role in both sensory and affective aspects of pain. mPFC neurons project to the midbrain region ventrolateral periaqueductal gray (vlPAG). Descending projections from the vlPAG to the locus coeruleus and raphe magnus are considered to suppress neural activities of the spinal cord and the trigeminal subnucleus caudalis. Mu-opioid receptors (MOR) are principally expressed in GABAergic interneurons in the mPFC and the endogenous μ-opioid system in the mPFC is reported to introduce placebo analgesia. However, it has been unknown whether inhibitory neurons expressing MOR modulate neural activities of pyramidal neurons projecting to the vlPAG. Here, we examined how MOR-expressing neurons in the mPFC modulate the activities of pyramidal neurons that project to the vlPAG. Whole-cell patch-clamp recording was performed using MOR-Cre-KI rats that received AAV-Flex-hChR2 (H134R)-mCherry injection into the mPFC and Alexa 647-conjugated cholera toxin subunit B (CTB) injection into the vlPAG. First, we recorded from mCherry-positive cells and found that most mCherry-positive cells showed repetitive spike firing with extremely high frequency, suggesting that they were fast-spiking GABAergic neurons. Next, we recorded responses to blue light stimulation from pyramidal neurons that were labeled with Alexa 647. We found that optical stimulation induced inhibitory postsynaptic potentials (IPSPs) in pyramidal neurons. These inhibitory responses obtained from pyramidal neurons projecting to the vlPAG were diminished by the administration of DAMGO, a selective MOR agonist, which was recovered by additional administration of CTAP, a selective MOR antagonist. These results suggest that MOR-expressing neurons in the mPFC are mostly GABAergic neurons and activation of MOR expressed in these inhibitory neurons is likely to suppress the inhibition of pyramidal neurons that increase vlPAG neuron activities under the situation when endogenous MOR ligands are released.

### [3P-009]

### Configuration of fronto-subcortical projections controlling mood and emotion: a comparative anatomical study in rodents and primates

Kei Kimura[1], Rintaro Yoshino[1], Yuki Soga[1], Madoka Hirose[1], Paola Alemán-Andrade[2], Taichi Kawamura[1], Soshi Tanabe[3], Andi Zheng[3], Shinya Nakamura[1,4], Shinya Ohara[1], Ken-ichi Inoue[3], Masahiko Takada[3], *Ken-Ichiro Tsutsui[1,2] ([1]*Tohoku Unviersity Graduate School of Life Sceinces,* [2]*Tohoku Unviersity Graduate School of Medicine,* [3]*Center for the Evolutionary Origins of Human Behavior, Kyoto University,* [4]*National Institute for Physiological Sciences*)

The medial frontal cortex (MFC), which includes the anterior cingulate cortex (ACC), is the main source of cortical projections to the amygdala. The interaction between MFC and the amygdala is of special interest, because many clinical studies have reported functional impairments in these structures in patients with depression. It is widely thought that MFC is involved in the regulation of negative emotion and mood, as well as in autonomic responses, via its projections to the amygdala. Based on the cytoarchitectural criteria, MFC and the amygdala can be divided into subregions and subnuclei, respectively. Clarifying the topographic projections from MFC to the amygdala may be a key to understand the function of MFC and the pathogenesis of psychiatric disorders related to the dysregulation of negative emotion and mood. Previous studies using conventional neural tracers reported that the projections from MFC to the amygdala originate mainly from the ventral and dorsal regions of ACC (sgACC and dACC, respectively) and terminate within the basal nucleus. While these studies have examined the distribution of cells projecting to the basomedial amygdala (BMA) within the basal nucleus, the cells of origin of projections to the basolateral amygdala (BLA) remain unknown. The present study aimed to comprehensively investigate the distribution patterns of MFC neurons projecting to BMA and BLA by using both adeno-associated virus vectors and biotin dextran amine (BDA), which permit anterograde and retrograde tracings. We found that MFC subregions projecting to BMA and BLA were clearly segregated except for sgACC that projects to both BMA and BLA. Notably, dACC was observed to be separated into the rostral part projecting to BLA and the caudal part projecting to BMA. Furthermore, the results of anterograde tracing from subregions of ACC revealed the topographic projection patterns into the periaqueductal gray (PAG). Additionally, we examined the fronto-limbic projection patterns in rodents and found that they also have a similar projecting pattern from amigdalar nucleus to MFC. Our results suggest that these ACC regions may play different roles in the regulation of emotion and mood via the projections to different subnuclei of the basal amygdalar nucleus and PAG.The authors declare no conflicts of interest associated with this study.

### [3P-010]

### Analysis of hippocampal ripple firings using convolutional neural network: AI learns training-dependent and experience-specific changes in the diversity

*Yuta Ishihara[1], Yuto Tomohara[1], Ken'ichi Fujimoto[1], Hiroshi Murai[2], Junko Ishikawa[2], Dai Mitsushima[2] ([1]*Kagawa Univ.*, [2]*Yamaguchi Univ.*)

The hippocampus is known to play an important role in memory by processing spatiotemporal information of episodic experiences. We previously recorded multiple-unit firing of hippocampal CA1 neurons during learning in freely behaving male rats. By extracting thousands of multiple-units short-term synchronized firing events (ripple firings: approximately 50 msec, frequency 300 Hz to 10 kHz), we found a diversity of the ripple firings specific to the preceding experience. In this study, we hypothesized that changes in this diversity might depend on the type of preceding experience. If this hypothesis holds, we can distinguish specific waveforms of ripple firings associated with each episode. To test this, we first tried to classify the thousands of ripple firings measured from rats into five categories: those recorded after experiencing one of the four episodes and those recorded before experiencing any of the four episodes. Preliminary analyses calculated the similarity of two ripple firings in each category using a cross-correlation function and tested for significant differences between categories using one-way analysis of variance (ANOVA) and Scheffé's method. Although ANOVA showed significant differences between any two categories, Scheffé's post-hoc analysis failed to show the difference between certain categories. Here we constructed a convolutional neural network (CNN) to classify the current stocks of ripple firings into these five categories. We also showed that the CNN can classify the ripple firings by finding episode-specific features that cannot be identified by conventional statistical methods. Since the findings suggest that almost all the ripple firings have certain features related to each episodic experience and its pre-experience, we would like to visualize the specific part of the firings to which the CNN pays attention. Once visualization is achieved, it is possible to identify the portion of experiential information contained in the ripple firings and contribute to the deciphering of experience-specific brain codes.

### [3P-011]

### Nicotinic effects on thalamocortical induced neuronal circuit activities in mouse primary auditory cortex.

*Makoto Nakanishi[1], Hideki Kawai[1] ([1]*Soka University, Department of Science and Engineering for Sustainable Innovation, Faculty of Science and Engineering*)

Systemic nicotine exposure is known to improve sensory cognitive function and attentional behavior in mammals including humans. In the rodent primary sensory system, acute nicotine exposure enhances thalamocortical (TC) axon excitability and increases synchronous synaptic inputs to primary auditory cortex (A1). However, how nicotinic activation regulates the intracortical circuit impinging upon thalamorecipient cells remains unclear.

We investigated the nicotinic regulation of thalamocortically evoked neuronal activities in excitatory neurons and inhibitory fast spiking (FS) interneurons in the layers 3/4 of A1.We prepared auditory TC slices, which maintain TC connections from the ventral division of medial geniculate body (MGv) to A1, using adolescent female mice (postnatal days 26-30, C57BL/6J and GAD67-eGFP knock-in mice). To elicit synaptic responses and/or action potentials in patched neurons, a parallel bipolar stimulation electrode was placed on the superior thalamic radiation (STR), the white matter in the auditory TC axon pathway. Neuronal activities in excitatory neurons and FS interneurons were recorded using the whole-cell patch-clamp method. Nicotine was applied locally to A1 via a perfusion needle or in the bath.

Local application of 0.3 mM nicotine in A1 mostly reduced the number of TC axonal stimulation-evoked action potentials in the excitatory neurons of A1. The amplitude of monosynaptic TC EPSCs and inhibitory synaptic inputs from FS interneurons were unaffected. However, in FS neurons, the probability of TC EPSC induction was reduced while the amplitude was unaffected. Nicotinic modulation of thalamocortically evoked polysynaptic EPSCs and IPSCs were then analyzed in excitatory neurons. Nicotine either enhanced or suppressed both EPSCs and IPSCs (i.e., co-tuned), depending on patched neurons. In the case of enhancement, the excitatory and inhibitory synaptic balance shifted towards inhibition. In the case of suppression, the decreases in EPSCs and IPSCs were nearly equally affected. These results may indicate that TC stimulation recruits (at least) two separate neural circuits in vitro, where nicotine enhances one circuit and suppresses the other. Future studies will reveal precise cellular and circuit mechanisms of nicotinic regulation in the thalamorecipient layers.

### [3P-012]

### Multiple-unit firing activity of hippocampal CA1 neurons expresses recent preceding experience

*Takashi Kuremoto[1], Junko Ishikawa[2], Shingo Mabu[2], Dai Mitsushima[2] ([1]*Nippon Institute of Technology*, [2]*Yamaguchi University*)

The hippocampus plays an important role in the formation of episodic memory. To identify patterns of hippocampal firing activity specific to episodic memory, we performed EEG recognition using deep learning methods. Briefly, adult male rats habituated to the home cage experienced one of four experimental episodic stimuli (restraint stress, contact with a female rat, contact with a male rat, or contact with a novel object) for 10 minutes. Recorded brain spike signals (300–10 kHz) in hippocampal CA1 were classified using machine learning methods such as convolutional neural networks (CNN), support vector machines (SVM), deep learning model VGG16, and combination models composed by CNN with SVM or VGG16 with SVM. As a result, VGG16 with SVM successfully detected multiple unit activity (MUA) with ripple firings corresponding to specific episodes, achieving a validation accuracy of 96.5%. The results suggest that the EEG containing ripple firings correspond to specific episodic memories. By capturing ripple firings, EEG analysis can assess and diagnose memory function, that may help detect various cognitive disorders.

### [3P-013]

### Axonal trajectories of individual commissural vestibular nucleus neurons

*Takahiro Ando[1], Izumi Sugihara[1], Mayu Takahashi[1] ([1]*Tokyo Medical and Dental University, Systems Neurophysiology*)

The vestibular nuclei receive input from the peripheral vestibular organ, and sends signals to various region in the central nervous system, such as the extraocular motor nuclei, spinal cord, the contralateral vestibular nucleus, and the cerebellum. Projection of vestibular nucleus neurons to the contralateral vestibular nuclei is well known as a path of the commissural inhibition system between the bilateral vestibular nuclei. Many physiological studies have revealed that it increases the sensitivity for angular head acceleration-related signals. However, its anatomical aspect has been less clarified. In this study, we examined the anatomical characteristics of this commissural projection by labeling individual vestibular nucleus neurons with biotinylated dextran amine (BDA) injected into the medial vestibular nucleus (MVN) and reconstructing the axonal trajectory of each labeled axons from serial sections in the mouse.Many labeled axon terminals were observed in the contralateral vestibular nucleus, predominantly in the medial (MVN), and superior vestibular nucleus (SVN), in addition to the nucleus prepositus hypoglossi (NPH). Labeled terminals were also distributed on both sides in the extraocular motor nuclei, and in the cervical cord, particularly in neck motor neuron areas.We reconstructed 28 axons nearly completely, identifying cell bodies in the MVN in 14 axons. Among these 28 axons, 7 axons arose from the MVN, crossed the midline, entered the medial longitudinal fasciculus (MLF), and then projected to the contralateral abducens nucleus. These axons gave rise to some collaterals terminating in the contralateral NPH and MVN, where many labeled terminals were observed mainly in the ventral part of the NPH, and magnocellular portion of the MVN. Three axons projected to the contralateral oculomotor or trochlear nucleus, and they had axon collaterals that terminated in the contralateral NPH, and MVN. Terminal distribution of these axons in NPH and MVN was similar to that of the axons that projected to the abducens nucleus.The remaining 18 axons arose from the MVN, crossed the midline, ran laterally and then projected to the contralateral vestibular nuclei. Labeled terminals of these axons were mainly observed in the parvocellular portion of the MVN, and peripheral part of the SVN.We examined axonal projection patterns of individual vestibular nucleus neurons in the contralateral vestibular nuclei. The results indicated that there are two types of commissural projection pattern of vestibular nucleus neurons; one group projects contralaterally to the extraocular motor nuclei with axon collaterals terminating in the vestibular nuclei and NPH, and the other group projects only to the vestibular nuclei on the contralateral side.

### Poster

[3P]
### Neurophysiology, Neuronal cell biology
**Neurons, Synapses**

March 30, 13:00 - 14:20, Poster Room

### [3P-014]
### Neuroprotective effects of Sinapic acid on Scopolamine-induced learning and memory impairment model mice

*In-Seo Lee[1,3], Ga-Young Choi[2], Inturu Sreelatha[1], Sungho Maeng[1], Ji-Ho Park[1,3]
(*[1]KYUNG HEE UNIVERSITY, [2]Korea Basic Science Institute, [3]Dept. of Gerontology(AgeTech-Service Convergence Major)*)

The seriousness of the diseases caused by aging have recently gained attention. Alzheimer's disease (AD), a chronic neurodegenerative disease, accounts for 60–80% of senile dementia cases. Continuous research is being conducted on the cause of Alzheimer's disease, and it is believed to include complex factors, such as genetic factors, the accumulation of amyloid beta plaques, a tangle of tau protein, oxidative stress, cholinergic dysfunction, neuroinflammation, and cell death. Sinapic acid is a hydroxycinnamic acid found in plant families, such as oranges, grapefruit, cranberry, mustard seeds, and rapeseeds. It exhibits various biological activities, including anti-inflammatory, anti-oxidant, anti-cancer, and anti-depressant effects. Sinapic acid is an acetylcholine esterase inhibitor that can be applied to the treatment of dementia caused by Alzheimer's disease and Parkinson's disease. However, electrophysiological studies on the effects of sinapic acid on memory and learning must still be conducted. Therefore, it was confirmed that sinapic acid was effective in long-term potentiation (LTP) using organotypic hippocampal segment tissue. In addition, the effect on scopolamine-induced learning and memory impairment was measured by oral administration of sinapic acid 10 mg/kg/day for 14 days, and behavioral experiments related to short-term and long-term spatial memory and avoidance memory were conducted. Sinapic acid increased the activity of the field excitatory postsynaptic potential (fEPSP) in a dose-dependent manner after TBS, and restored fEPSP activity in the CA1 region suppressed by scopolamine. The scopolamine-induced learning and memory impairment group showed lower results than the control group in the Y-maze, Passive avoidance (PA), and Morris water maze (MWM) experiments. Sinapic acid improved avoidance memory, short and long-term spatial recognition learning, and memory. In addition, sinapic acid weakened the inhibition of the brain-derived neurotrophic factor (BDNF), tropomyosin receptor kinase B (TrkB) and the activation of prostaglandin-endoperoxide synthase 2 (COX-2) and interleukin 1 beta (IL-1β) induced by scopolamine in the hippocampus. These results show that sinapic acid is effective in restoring LTP and cognitive impairment induced by the cholinergic receptor blockade. Moreover, it showed the effect of alleviating the reduction in scopolamine-induced BDNF and TrkB, and alleviated neuroinflammatory effects by inhibiting the increase in COX-2 and IL-1β. Therefore, we showed that sinapic acid has potential as a treatment for neurodegenerative cognitive impairment.

### [3P-015]
### Ghrelin-induced electrophysiological responses of neurons in the deep cerebellar nuclei

*Moritoshi Hirono[1], Boyang Zhang[1], Hiroshi Hosoda[2], Masanori Nakata[1] (*[1]Dept Physiol, Wakayama Med Univ, [2]Dept Mol Pathophysiol, Shinshu Univ*)

The cerebellum is involved in motor coordination and motor learning, and instrumental in cognition, emotion and reward. Yet, the relationship between cerebellar functions and feeding behavior remains unclear. Ghrelin, an endogenous orexigenic peptide, is secreted into the circulation from the stomach during fasting and transfers through the blood-brain barrier and exist in the brain as well. Previously we reported that the peptide facilitates spontaneous firing of cerebellar Purkinje cells. However, little is known about its physiological effects on neurons in the deep cerebellar nuclei (DCN). Immunohistochemically, we investigated expression patterns of ghrelin and its growth hormone secretagogue receptor 1a (GHS-R1a) in the DCN of mice. We further examined the effects of ghrelin on spontaneous firing of DCN neurons using patch clamp recordings. Ghrelin and GHS-R1a were strongly expressed in DCN neurons. Bath-application of ghrelin to mouse cerebellar slices reduced the spontaneous firing rate of DCN neurons, whereas the peptide caused inward currents in the neurons. This reduction did not change with blockers for excitatory and inhibitory synaptic transmissions, suggesting that ghrelin suppresses the excitability of DCN neurons directly through their GHS-R1a activation. Thus, ghrelin attenuates cerebellar output signals directly and indirectly, thereby controlling the reward system and subsequently adjusting the feeding behavior.

### [3P-016]
### Adverse effects of A$\beta_{1\text{-}42}$ oligomers: impaired contextual memory and altered intrinsic properties of CA1 pyramidal neurons

*MIN KAUNG WINT MON[1], Kida H[1], Kanehisa I[1], Kurose M[1], Sakimoto Y[1], Ishikawa J[1], Kimura R[2], Mitsushima D[1] (*[1]Department of Physiology, Yamaguchi University Graduate School of Medicine, [2]Center for Liberal Arts and Sciences, Sanyo-Onoda City University*)

Alzheimer's disease is characterized by synaptic dysfunction, increased excitatory/inhibitory balance and neuronal hyperactivity with A$\beta_{1\text{-}42}$ (amyloid beta) oligomers as the major culprit. Although much of the synaptotoxic effects have been explored in the *in vitro* application of A$\beta_{1\text{-}42}$ oligomers, we found the effects of *in vivo* injection of A$\beta_{1\text{-}42}$ oligomers on contextual memory and intrinsic/synaptic properties of CA1 pyramidal neurons. A$\beta_{1\text{-}42}$ oligomers were bilaterally injected into the dorsal CA1 region of 4-week-old male rats for behavioral tests and electrophysiological studies. After 1 week, the A$\beta_{1\text{-}42}$-injected rats showed reduced latency in the inhibitory avoidance (IA) task and displayed less freezing in the contextual freezing (CF) test without affecting spontaneous movement, pain sensitivity and emotional state. Congo red that has been used to identify amyloid fibrils clearly stained membranes of CA1 pyramidal neurons in the A$\beta_{1\text{-}42}$-injected group, but not in the control. In current clamp analysis, we discovered an increase in membrane resistance and spike numbers as well as a reduction in threshold current after learning, highlighting neuronal hyperexcitability in the A$\beta_{1\text{-}42}$ group. Further, bath application of riluzole, a reversible voltage-gated sodium channel (Nav) antagonist, successfully reduced spikes in a concentration-dependent manner, inhibiting the toxic effect of A$\beta_{1\text{-}42}$ oligomers. In contrast, TTX (tetrodotoxin), an irreversible antagonist, completely blocked the generation of action potential spikes and thus failed to maintain normal firing pattern as found in the control. These data provide new clues as to how A$\beta_{1\text{-}42}$ oligomers could cause memory impairment and neuronal hyperexcitation, leading to the development of new therapeutic strategies.

### [3P-017]

### Regular voluntary exercise prevents decreased NAD+ levels and cognitive function in aged mice.

*Jimmy Kim[1], Jonghyuk Park[2], Ko Yamanaka[1], Hidefumi Waki[1,3] ([1]*Department of physiology, Graduate School Health and Sports Science, Juntendo Univ.*, [2]*Brain Metabolic Regulation Group, Department of Psychiatry and Behavioral Sciences, Tokyo Metropolitan Institute of Medical Science*, [3]*Institute of Health and Sports Science & Medicine, Juntendo Univ.*)

Nicotinamide adenine dinucleotide ($NAD^+$) is an essential coenzyme in all living cells involved in fundamental biological processes, namely metabolism, cell signaling, gene expression, and DNA repair, among others. However, the amount of intracellular $NAD^+$ has been reported to decrease with age. This systemic decrease in $NAD^+$ levels during aging is partly due to decreased nicotinamide phosphoribosyltransferase (NAMPT), the rate-limiting enzyme in a major $NAD^+$ biosynthetic pathway in mammals. NAMPT is the critical enzyme in the salvaging pathway for the biosynthesis of $NAD^+$. In particular, it plays an essential role in aging, inflammation, energy metabolism, and other age-associated functions. Also, Brain-specific NAMPT knockdown in young and aged mice has been reported to reduce hippocampal $NAD^+$ levels and contribute to the development of cognitive impairment. Further, the preventive effect of regular exercise against age-related cognitive decline is widely known. However, no research has revealed the relationship between hippocampal $NAD^+$, especially NAMPT levels, and improved cognitive function due to exercise. We investigated the preventive effect of regular exercise on age-related cognitive decline and $NAD^+$ levels from the perspective of changes in hippocampal NAMPT levels. Twenty-month-old C57BL/6J female mice were divided into a control group (non-exercise) and an Exercise group (regular voluntary exercise). The exercise group was allowed three times a week and only did spontaneous exercise at night. Behavioral experiments were conducted after the animals were kept under these conditions for four weeks, and blood, hippocampus, and skeletal muscle were collected under anesthesia. Regular exercise improved working and long-term memory and anxiety-like behaviors in aged mice. In addition, regular exercise decreased age-related inflammatory responses and increased NAMPT, BDNF mRNA expression, and $NAD^+$ levels in the hippocampus. Our results suggest that the prevention of age-related cognitive decline and the improvement of cognitive function through regular exercise are associated with an increase in $NAD^+$ levels via NAMPT in the hippocampus.

### [3P-018]

### P2X receptor- and postsynaptic NMDA receptor-mediated long-lasting facilitation of GABAergic synapses in the rat insular cortex

*Kiyofumi Yamamoto[1], Masayuki Kobayashi[1] ([1]*Department of Pharmacology Nihon University School of Dentistry*)

Adenosine triphosphate (ATP) changes the efficacy of synaptic transmission. Despite recent progress in terms of the roles of purinergic receptors in cerebrocortical excitatory synaptic transmission, their contribution to inhibitory synaptic transmission is unknown. To elucidate the effects of α,β-methylene ATP (αβ-mATP), a selective agonist of P2X receptors (P2XRs), on inhibitory synaptic transmission in the insular cortex (IC), we performed whole-cell patch-clamp recording from IC pyramidal neurons (PNs) and fast-spiking neurons (FSNs) in either sex of VGAT-Venus transgenic rats. αβ-mATP increased the amplitude of miniature IPSCs (mIPSCs) under conditions in which NMDA receptors (NMDARs) are recruitable. αβ-mATP-induced facilitation of mIPSCs was sustained even after the washout of αβ-mATP, which was blocked by preincubation with fluorocitrate. The preapplication of a $P2X_1$ receptor antagonist or a $P2X_3$ receptor antagonist blocked αβ-mATP-induced mIPSC facilitation. Intracellular application of the NMDAR antagonist MK801 blocked the facilitation. D-serine, which is an intrinsic agonist of NMDARs, mimicked αβ-mATP-induced mIPSC facilitation. The intracellular application of BAPTA, or the bath application of a CaMKII inhibitor blocked αβ-mATP-induced mIPSC facilitation, thus indicating that mIPSC facilitation by αβ-mATP required postsynaptic $[Ca^{2+}]_i$ elevation through NMDAR activation. Paired whole-cell patch-clamp recordings from FSNs and PNs demonstrated that αβ-mATP increased the amplitude of unitary IPSCs without changing the paired-pulse ratio. These results suggest that αβ-mATP-induced IPSC facilitation is mediated by postsynaptic NMDAR activations through D-serine released from astrocytes. Subsequent $[Ca^{2+}]_i$ increase and postsynaptic CaMKII activation may release retrograde messengers that upregulate GABA release from presynaptic inhibitory neurons, including FSNs.

### [3P-019]

### Physiological significance of the protein complex formed by delta type glutamate receptor and Cbln family in hippocampus.

*Itaru Arai[1], Keiko Matsuda[1], Kotaro Konno[2], Shigetomo Suyama[1], Ayumi Hayashi[1], Masahiko Watanabe[2], Michisuke Yuzaki[1] ([1]*Keio Univ. Sch. Med.*, [2]*Hokkaido Univ.*)

The delta glutamate receptor (GluD), consisting of GluD1 and GluD2, belongs to the ionotropic glutamate receptor family based on amino acid sequence similarity. However, while D-Ser binds to the ligand binding domain of GluD1 and GluD2, it has been unclear whether and how GluD receptors mediate synaptic transmission as ion channels. Interestingly, postsynaptic GluD2 regulates synapse formation and maintenance and synaptic plasticity independently of channel function by binding to presynaptic neurexins (NRXs) via cerebellin1 (Cbln1), forming a tripartite complex (GluD2 - Cbln1 - NRX) at the parallel fiber-Purkinje cell synapse in the cerebellar cortex.

It is now widely accepted that GluDs and Cblns are also expressed in various brain regions outside the cerebellum. In the hippocampus, for example, GluD1 and Cbln1 and 4 have been reported to be highly expressed. However, it is still unclear with which Cbln GluD1 forms complexes and what their physiological roles are in different regions within the hippocampus. In the present study, we investigated the detailed localization of GluD1 and Cbln1,4 in the mouse hippocampus and their physiological roles.

Immunohistochemistry revealed that GluD1 is highly expressed in the middle part of the molecular layer in DG (DG-mML) and in the stratum lacunosum moleculare in CA1 (CA1-LM). To identify the endogenous ligand for GluD1 in these regions, in situ hybridization (ISH) was first performed in the entorhinal cortex (EC), since it is known that granule cells (GCs) of EC layers 2 and 3 project to the DG-ML and CA1-LM, respectively. ISH revealed that Cbln1 mRNA was highly expressed in layer 2 GCs. On the other hand, Cbln4 mRNA was expressed in both layer 2 and layer 3 GCs. Next, immunohistochemistry was performed in the hippocampus and showed that Cbln1 was mainly expressed in DG-mML and Cbln4 was expressed in both DG-mML and CA1-LM, supporting the result of ISH. In addition, Cbln1 and 4 were co-localized with GluD1 in both DG-mML and CA1-LM, suggesting that they bind to each other to form a complex. We will further discuss how and what physiological roles the GluD1-Cbln1 and 4 complex plays in distinct hippocampal sub-regions.

### [3P-020]

### Prenatal exposure to valproic acid in mice leads to abnormalities in synaptic pruning and microglia activation.

*Tomoki Tamura[1], Kenjiro Sato[2], Nobuhiko Kojima[1,2] ([1]*Graduate School of Life Science, Toyo University*, [2]*Center for Biomedical Engineering Research, Toyo University*)

Prenatal exposure to the anti-epileptic drug valproic acid (VPA) is known to increase the risk of developing autism spectrum disorder (ASD). Because prenatal VPA exposure causes behaviors similar to human ASD in rodents, postnatal VPA-exposed mice have been widely studied as ASD model animals. It has been reported that prenatal VPA-exposed mice have significantly reduced dendritic spine density in brain regions including the cerebral cortex and the hippocampus (Yamaguchi et al., 2017; Hara et al., 2017). It has also been reported that activated microglia are enhanced in these brain regions in VPA-exposed mice (Gassowska et al., 2020; Luo et al., 2023). Microglia are known to be activated in response to inflammation and function in normal synaptic pruning and removal of damaged neurons. However, current knowledge is insufficient to prove a causal relationship between enhanced microglial activation and reduced dendritic spine density. Therefore, we then hypothesized that in the developing brain prenatal VPA exposure enhances microglial activation, leading to abnormal synaptic pruning and a gross reduction in dendritic spine density and tried to prove this hypothesis. To determine the precise time course of glial cell activation and synaptic pruning, we analyzed the brains of prenatal VPA-exposed mice longitudinally. The cerebral cortex and hippocampus of VPA-exposed mice on postnatal days 7, 14, 21, 28, and 30 were histologically analyzed to detect temporal changes in dendritic spine density, dendritic length and dendritic complexity. We also investigated in parallel microglial activation by immunostaining with anti-Iba-1 antibody. As a result, we confirmed that the time course of microglial activation in the cerebral cortex is consistent with that of the reduction of dendritic spine density. Finally, to investigate changes in gut microbiota in this ASD mouse model, we performed meta 16S analysis of DNA extracted from the cecum contents of VPA-exposed mice. Since microglial activation is known to be bidirectionally related to changes in the gut microbiota, comparing the diversity of gut microbiota in VPA-exposed mice and control mice gut microbiota may help us understand the interrelationship between gut microbiota and microglial activation in the brain. These studies are designed to determine the relationship between changes in dendritic spine in the brain of VPA-exposed mice, the regulatory function of microglia in synaptic pruning, and the gut microbiota. This study will contribute to the elucidation of the molecular mechanism involved in the etiology of ASD and to the identification of factors that ameliorate problematic behaviors in ASD patients.

### [3P-021]

### Knockout of *DEPDC5*, the gene associated with drug-resistant focal epilepsy, causes abnormal $Ca^{2+}$-waves in human iPS cell-induced neurons

*Yuna Horiuchi[1], Haruka Inaba[2], Tetsuya Hando[2,3], Tomohiko Ai[2,4], Takashi Miida[2,3]
(*[1]Department of Clinical Laboratory Technology, Juntendo University, Faculty of Medical Science, [2]Department of Clinical Laboratory Medicine, Juntendo University Graduate School of Medicine, [3]Department of Clinical Laboratory, Juntendo University Hospital, [4]Department of Clinical Laboratory Medicine, Juntendo University Urayasu Hospital*)

Genetic variants in the DEP domain containing 5 (DEPDC5) are one of the most common causes of inheritable drug-resistant temporal focal epilepsy. Epilepsies caused by *DEPDC5* variants are often associated with brain malformations and sudden unexplained death in epilepsy (SUDEP). It has been reported that the *DEPDC5* genes regulates cell growth via rapamycin complex 1 (mTORC1) pathways. Though several animal models showed knockout of the *DEPDC5* causes epilepsy, the underlying mechanisms by which *DEPDC5* variants cause neuronal excitations remains unelucidated in human cells.
To examine whether knockout of *DEPDC5* causes abnormal excitations in human iPS cell (iPSC)-induced neurons, *DEPDC5* was knocked out using CRISPR-Cas9 gene editing methods. Expression of DEPDC5 peptides was confirmed by a mass spectroscopy. Then, the cells were differentiated into neurons by Dual Smad Inhibition. Spontaneous cellular excitation was evaluated by $Ca^{2+}$-imaging (AquaCosmos/Ratio imaging system, Hamamatsu Photonics). DNA sequencing of the *DEPDC5*-knockout cells showed a truncated change, c.251_258delCTTTGGGG:(p.Gly15Glyfs*5), in one allele. The DEPDC5 peptides encoded by exon 7 were decreased to less than half in the *DEPDC5*-knockout cells compared to the wild type (WT) cells. $Ca^{2+}$-imaging showed abnormal $Ca^{2+}$ bursts in neurons derived from the *DEPDC5*-knockout cells, while the WT cells did not show any abnormal $Ca^{2+}$ waves. These results indicate that haploinsufficiency of *DEPDC5* can cause abnormal excitation in human iPSC-induced neurons. Currently, we are planning to further investigate cellular excitations in iPSC-induced cardiomyocytes to dissect underlying mechanisms of SUDEP.

This work was supported in part by a Grant-in-Aid for Scientific Research (C) [grant number 18K08900 and 21K08135 (to T.A.)] and a Grant-in-Aid for Young Scientists [grant number 22K15694 (to Y.H.)] from the Japan Society for the Promotion of Science.

### [3P-022]

### Synaptic molecular alterations in the striatum of obsessive-compulsive disorder model mice induced by chronic administration of a dopamine D2-like receptor agonist quinpirole

*Qile Yang[1], Hirokazu Sakamoto[1], Kenzo Hirose[1] (*[1]Department of Pharmacology, Graduate School of Medicine, University of Tokyo*)

Obsessive-compulsive disorder (OCD) is a chronic psychiatric disorder with a prevalence of 1 to 2%, which is also stated as one of the top ten disabling diseases by WHO. According to previous research, the hyperactivation of cortico-striatal-thalamic-cortical loop could be one of the causes of OCD, and alterations in glutamatergic transmission may play key roles in its pathology. However, the molecular mechanisms have not been clarified yet. In this research, we generated OCD model mice induced by chronic administration of a dopamine D2-like receptor agonist quinpirole, which is a classical method used to generate OCD model animals (Szechtman H et al. 1998), and performed quantitative high-resolution immunofluorescence imaging of synaptic proteins in the brain to investigate the molecular alterations in glutamatergic synapses of the model mice. An open field test confirmed that quinpirole administrated C57B6/J mice show increased locomotion and repetitive behaviors, which are hallmarks of OCD-like behavior. We found that the NMDA receptor subunit NR2A was specifically decreased in the glutamatergic synapses in the striatum of the OCD model mice, while NR2B subunit, glutamatergic postsynaptic scaffold proteins (PSD95 and SAPAP), and dopamine receptors (D1 and D2 receptor) showed little change. Furthermore, super-resolution imaging revealed an alteration in the sub-synaptic distribution of NMDA receptors in the striatum synapses of the OCD model mice. These results suggest that biased alterations in postsynaptic proteins of glutamatergic synapses in the striatum might involve OCD pathology, and that detailed analysis of the alterations in different time points may help to define the onset and maintenance mechanisms of OCD.

### Poster

### [3P]
### Neurophysiology, Neuronal cell biology
**Sensory function, Sensory organ**

March 30, 13:00 - 14:20, Poster Room

### [3P-023]

### Reversal potential of GABA responses in starburst amacrine cells changes during developmental period in the mouse retina

*chengzhu yin[1], toshiyuki ishii[1], kaneda makoto[1] ([1]*Dept. Physiol., Nippon Medical School*)

In the mammalian nervous system, GABAergic inputs switch from an excitatory input to an inhibitory input during developmental period. The underlying mechanism for this polarity change of GABA responses is attributed to the switching of subtypes of chloride transporter (NKCC1 and KCC2), which results in the change of the intracellular $Cl^-$ concentration. In early stage of development, since NKCC1, which transports $Cl^-$ into cells, is dominant, the intracellular $Cl^-$ concentration is high. When KCC2, which transports $Cl^-$ out of cells, starts to contribute, the intracellular $Cl^-$ concentration starts to decrease and reaches the adult level. In the cells of the ganglion layer in the mouse retina, similar change of the intracellular $Cl^-$ concentration has been reported. In addition, we have previously reported that the difference of the intracellular $Cl^-$ concentration between ON- and OFF-bipolar cells is generated by the difference of the activity between NKCC1 and KCC2 in the mouse retina. However, such a study has not been carried out in the starburst amacrine cells, a key neuron for the formation of neural circuits of direction selectivity in the retina. In the present study, therefore, we examined whether such a switching of chloride transporter occurs in the starburst amacrine cells using patch clamp technique in mGluR2-GFP transgenic mouse, which selectively express GFP signals in starburst amacrine cells. We also examined whether intracellular $Cl^-$ concentration is different between ON- and OFF-starburst amacrine cells like bipolar cells. The reversal potential of GABA responses shifted to the hyperpolarized side during development (-29.5 ± 16.1 mV at P2-P4, -49.1 ± 4.4 mV at P9-P13, and -53.8 ± 0.9 mV at P20-P30). The similar shift of reversal potential was observed in both ON- and OFF-starburst amacrine cells. Our results support the hypothesis that switching of chloride transporter during developmental stage contributes the polarity of GABA responses in the developing ON- and OFF-starburst amacrine cells in the mouse retina.

### [3P-024]

### Role of tachykinin peptides in nociceptive- and pruriceptive-processing

*Rumi Nakayama-Naono[1], Akihiro Ambo[1], Kazufumi Ikuta[1,2], Ryuichi Harada[1], Tadaho Nakamura[1], Hideki Funahashi[3], Nobuyuki Okamura[1] ([1]*Tohoku Medical and Pharmaceutical Univ.*, [2]*Kanazawa Univ.*, [3]*Univ. of Miyazaki*)

Tachykinin peptide, represented by substance P (SP) are one of the neuropeptides, and share a common amino acid sequence. Tachykinin peptides include hemokinin-1 (HK-1) and four other endokinin peptides (EKs). EKs are classified into four peptides; endokinin A (EKA), B (EKB), C (EKC) and D (EKD), respectively. The physiological functions of these peptides in nociception and pruritic stimulus processing are not fully elucidated. Previous studies have shown that among the EKs, the EKA and EKB (EKA/B) have a role as excitatory peptides, like as SP. On the other hand, the EKC and EKD (EKC/D) play a role in suppressing EKA/B and SP function. In addition, EKC/D has been shown to suppress nociceptive behavior when administered intrathecally to rodents, and inhibit inflammation when administered to peripheral tissues. It is expected that the difference in the function of the EKs may be due to the difference in the C-terminal amino acid. In the analysis of the role of tachykinin peptides in pruritus, our results suggest that these peptides are involved in itch behavior, and the differences in the C-terminal amino acid sequence may lead to different functions in itch transmission system. These results indicate that EKA/B and EKC/D are important role in nociceptive and pruriceptive processing.

### [3P-025]

### Investigating the aftereffect in tactile temporal order judgement in individuals with autism spectrum disorder and attention-deficit hyperactivity disorder

*Kevin Widjaja[1,2], Aya Sato[1], Naomi Ishii[1], Yukie Nagai[3], Makoto Miyazaki[2,4], Makoto Wada[1] ([1]*Department of Rehabilitation for Brain Functions, Research Institute of National Rehabilitation Center for Persons with Disabilities, Tokorozawa, Saitama, Japan*, [2]*Graduate School of Science and Technology, Shizuoka University, Hamamatsu, Shizuoka, Japan*, [3]*International Research Center for Neurointelligence, The University of Tokyo, Bunkyo-ku, Tokyo, Japan*, [4]*Faculty of Informatics, Shizuoka University, Hamamatsu, Shizuoka, Japan*)

Individuals with autism spectrum disorders (ASD) have atypical time perception/cognition and are not able to adapt to prior experience effectively, leading to difficulties in real life. A previous psychophysical study using tactile temporal order judgement (TOJ) suggests a small or no positive aftereffect ('Bayesian calibration') in individuals with ASD (Wada et al., 2023). The result is consistent with the 'hypo-priors' hypothesis for autistic perception (Pellicano & Burr, 2012). In this study, however, one individual who was diagnosed with ASD and attention-deficit hyperactivity disorder (ADHD) displayed a large adaptation aftereffect contrast with the hypothesis. The co-occurrence of ASD and ADHD might contribute to the contrasting result. In the present study, we further investigated the adaptation aftereffect in individuals ASD and ADHD. Twelve diagnosed participants (6 individuals with ASD, 6 individuals with ASD and ADHD) engaged in a tactile TOJ task. In each trial, participants received two tactile stimuli to both their hands and judged the order of the stimuli, as either right-first or left-first. Each participants completed two sessions (248 trials/session) of the task. In each session, we presented the participants with either a right-first or left-first biased tactile stimuli. Autism quotient (AQ), empathy quotient (EQ), systemizing quotient (SQ), and Conners' Adult ADHD Rating Scales (CAARS) were used to measure participants' autistic and ADHD traits, respectively. As a result, we observed a Bayesian calibration in four of the six participants with ASD and ADHD, whereas five of the six participants with ASD showed no adaptation aftereffect. Notably, two participants, one individual with ASD and the other with ASD and ADHD, who showed contrasting results are taking antipsychotic and psychostimulant medications, respectively. Altogether, our results so far suggest that ASD and ADHD conditions might have their relative contributions to individuals' learning ability. Nonetheless, further statistical confirmation is needed to provide any valid conclusion.

### [3P-026]

### The chemotherapeutic agent irinotecan impairs amiloride-sensitive sodium taste responses in mice

*Makoto Sugita[1], Nobuyuki Sakayori[1], Hiroyuki Kawaguchi[2], Nami Obayashi[1,2]
([1]*Department of Physiology and Oral Physiology, Graduate School of Biomedical and Health Sciences, Hiroshima University,* [2]*Department of General Dentistry, Hiroshima University Hospital*)

Taste alteration is a side effect observed in cancer patients receiving chemotherapy. Patients receiving irinotecan appear to report significantly more taste alterations than patients in other treatment groups. However, it remains elusive how irinotecan administration causes taste disturbances, and which type of taste dysfunction is elicited by irinotecan. Here we combined the two-bottle preference tests for examining taste responses and immunohistochemical analyses in mice to clarify the types and mechanisms of taste alteration induced by irinotecan administration. Irinotecan administration reduced the intake of solution with sodium taste, but had no effect on sweet taste responses. In the presence of the epithelial sodium channel (ENaC) blocker amiloride, the intake of sodium taste solution was comparable between the irinotecan-treated and control groups. Irinotecan administration decreased α-ENaC immunoreactivity detected in taste bud cells without affecting the number of the cellular proliferation marker Ki67-positive cells within or around taste buds. Our results suggest that behavioral sodium taste responses originating from ENaC function may be altered by irinotecan administration.

### [3P-027]

### A role for vasopressin in reciprocal synaptic transmission in the mouse accessory olfactory bulb: effects on the voltage-activated $Ca^{2+}$ currents recorded from mitral cells

*Mutsuo Taniguchi[1], Yoshihiro Murata[1], Masahiro Yamaguchi[1], Hideto Kaba[1] ([1]*Dept. of Physiol., Kochi med. sch., Kochi univ.*)

Central vasopressin (AVP) facilitates social recognition and modulates many complex social behaviors in mammals. Vasopressin neurons were reported to exist in the accessory olfactory bulb (AOB), the first relay in the vomeronasal system. The AOB has been demonstrated to be a critical site for mating-induced mate recognition (olfactory memory) in female mice. The effect of AVP, however, on the synaptic transmission between dendrites in the AOB of female mice is largely unknown.
By measuring the reciprocal synaptic currents (IPSCs) from mitral cells in the AOB, we have demonstrated that AVP significantly reduced the IPSCs via V1a receptors. The reciprocal transmission contains both glutamatergic transmission from mitral cells to granule cells and GABAergic one from granule to mitral cells. Concerning to GABAergic transmission, we have also reported that extracellular application of AVP significantly suppressed voltage-activated $Ca^{2+}$ currents in the granule cells without affecting the magnitude of the response of mitral cells to GABA, suggesting that AVP reduces the GABAergic transmission through a presynaptic mechanism.
In the present study, we have examined the effect of V1a receptor activation on presynaptic properties in the glutamatergic transmission (that is, mitral cell activities). AOB slices were prepared from 23- to 35-day-old Balb/c mice. Using the whole-cell voltage clamp, the current response of mitral cells was recorded in the presence of antagonist for GABAergic transmission, picrotoxin. Recording from mitral cells, an extracellular application of AVP diminished the $Ca^{2+}$ currents, suggesting that AVP reduces the glutamatergic transmission to some extent through the inhibition of $Ca^{2+}$ channels.

### [3P-028]

### The relationship between brain-wide acetylcholine dynamics and behavior in the mouse visual detection task.

*Akinori Y Sato[1], Ryosuke Takeuchi[1], Kei Ito[1], Masahito Yamaguchi[1], Fumitaka Osakada[1,2,3] ([1]*Graduate School of Pharmaceutical Sciences, Nagoya University,* [2]*Laboratory of Neural Information Processing, Institute for Advanced Research, Nagoya University,* [3]*Institute of Nano-Life-Systems, Institutes for Innovation for Future Society, Nagoya University*)

Sensory processing dynamically changes depending on behavioral contexts, psychological states, and movements of animals. These changes are regulated primarily by neuromodulators, which are molecules released from specific neurons to a wide area of the brain and modulate the activities of neurons throughout the brain. Acetylcholine is one of the neuromodulators regulating various brain functions, including visual perception. However, the neural mechanism of the modulation remains unclear because few studies have focused on brain regions other than the primary visual cortex (Pinto et al., 2013). To investigate the relationship between neuromodulation and visual perception with a focus on the brain-wide effects of acetylcholine, we recorded acetylcholine dynamics across the mouse brain using wide-field imaging of a genetically encoded fluorescent sensor of acetylcholine, iAChSnFR (Borden et al., 2020). We assessed the visual perception using a visual detection task for head-fixed mice. In this task, a visual stimulus was presented on either the left or right display in front of a mouse. The mouse was rewarded with a water drop by licking a right or left spout corresponding to the stimulus. iAChSnFR imaging during the task demonstrated that acetylcholine levels changed in response to stimulus presentation and licking behavior to report the stimulus detection. To evaluate the contribution of the task events and the behavior to the acetylcholine dynamics, we constructed a generalized linear model. The task events and the mouse movements tended to contribute to the dynamics in the anterior and posterior regions of the brain, respectively. These results suggest modulation of visual information processing by acetylcholine in different brain regions in response to both task events and behavioral movements, highlighting the relationship between brain-wide acetylcholine and visual perception.

### [3P-029]

### Anti-pruritic effects of linalool odor exposure in mice

Sho Ueno[1], Tatsuroh Kaneko[1], Tomoyuki Kuwaki[1], *Hideki Kashiwadani[1] ([1]*Department of Physiology, Graduate School of Medical and Dental Sciences, Kagoshima University*)

*Background:* Pruritus is one of the uncomfortable feelings on the skin and sometimes impairs our quality of life. Though antihistamines are the initial drug of choice for the treatment of pruritus, antihistamines-resistant prurituses evoked by various pruritogens via histamine-independent pathways are reported. Thus, the development of treatment for antihistamines-resistance pruritus is one of the pressing issues. Previously we have shown that odor of linalool, one of the monoterpene alcohols in lavender extracts, induced analgesic effects triggered by olfactory input. The linalool analgesia significantly attenuates formalin-induced pain, raising the hypothesis that the linalool odor may also attenuate antihistamine-resistance chemical pruritus. To address the hypothesis, we observed the scratching behaviors evoked by intradermal administration of pruritogens in mice under linalool odor exposure. *Methods:* Male C57/BL6 and CD1 (ICR) mice were used. We injected pruritogens intradermally (chroloquine (200 mg / 50 mL / site) or serotonin (50 mg / 50 mL / site)) at the nape of the neck of mice. Immediately after the injection, mice were placed in observation chamber ventilated with linalool odor or odorless air (as control). Then scratching behaviors were video-recorded for 30 minutes and scratching bouts were counted. To examine the contribution of the olfactory input to the anti-pruritic effects, we deprived olfaction through bilateral olfactory bulb suctioning (olfactory bulbectomy). Two weeks after the bulbectomy, we assessed the anti-pruritic effect of linalool odor using chroloquine-induced scratching model. *Results:* In chroloquine and serotonin induced scratching models, linalool odor exposure significantly reduced scratching behaviors for the first 6 minutes after pruritogen injection. After bulbectomy, the anti-pruritic effects of linalool odor were disappeared, indicating that olfactory sensory input triggered the anti-pruritic effects. The anti-pruritic effects were observed in both C57BL/6 and CD1 (ICR) mice. *Conclusion:* Linalool odor exposure induced anti-pruritic effects to antihistamines-resistant pruritus. Furthermore, the effects were triggered by olfactory input evoked by linalool odor. These results suggest the potential benefit of linalool odor on the control of pruritus in clinical situation.

### Poster

### [3P]
### Molecular physiology, Cell physiology
### Ion channels, Receptors

March 30, 13:00 - 14:20, Poster Room

### [3P-030]

### Single-channel ion-permeation of the Aquaporin 6 has a large unitary conductance and tunable anion and cation selectivity depending on pH

*Takahisa Maki[1], Oiki Shigetoshi[1], Iwamoto Masayuki[1] ([1]*University of Fukui*)

Homotetrameric Aquaporins (AQPs) possess a water selective pore (hereafter aqua pore) in each monomer. The water selectivity of an aqua pore is strictly defined by two conserved structural filters, the aromatic-arginine (ar/R) filter and the Asn-Pro-Ala (NPA) motifs (water selectivity filter). Among AQPs, Aquaporin 6 (AQP6) is a unique AQP that is ion-permeable. However, whether the aqua pore shares the ion permeation route remains controversial. The structural information is useful for the identification of the ion pore, but is unavailable for AQP6. Alternatively, the subunit structure of human AQP6 (hAQP6), rather than the tetrameric structure, is registered in the AlphaFold Protein Structure Databases. The structure of the ar/R filter and NPA motifs is indistinguishable from hAQP1, which is highly water selective. hAQP1 is ion-permeable upon cGMP stimuli. A central pore in the tetrameric structure of hAQP1 has been proposed as a candidate for an ion permeation route. Here, we examined the ion permeation route of the hAQP6 by single-channel current recording in the contact bubble bilayer to which purified hAQP6 is reconstituted. hAQP6 had a large unitary conductance of ~330 pA at acidic pH and ~630 pS at neutral pH with a weak rectification in the symmetrical 100 mM NaCl, suggesting that the ion permeable pore is wide. The channel exhibits unusual bell-shaped voltage-dependent gating, opens fully below ±50 mV, and nearly closed over ±200 mV, while exhibiting transitions among multiple sub-conductance levels in the transition voltages. The reversal potential revealed slight anion selectivity at acidic pH ($P_{Na}/P_{Cl}$ = 0.14). Surprisingly, the selectivity changed from anionic to cationic at neutral pH ($P_{Na}/P_{Cl}$ = 9.8) when the solution pH changed from acidic to neutral, further supporting the wide pore. Mercury ion also modified the selectivity. One-side pH perfusion from acidic to neutral pH revealed the effective side for increasing the single-channel conductance relevant to the channel orientation. The homotetrameric structure of hAQP6 constructed with AlphaFold2 revealed that the central pore of the homotetramer has the largest radius among AQPs, making it a candidate for the ion permeation route of hAQP6.

### [3P-031]

### PI(4,5)$P_2$ binding to α subunit regulates channel activities of mammalian $GABA_A$ receptors as revealed by the method of photocaged amino acid

*Rizki Tsari Andriani[1,2], Risa Mori-Kreiner[1], Natsuki Mizutani[1], Daisuke Yoshioka[1], Takafumi Kawai[1], Yasushi Okamura[1] ([1]*Integrative Physiology, Graduate School of Medicine, Osaka University.*, [2]*Graduate School of Medicine, Osaka University JSPS International Research Fellow*)

PI(4,5)$P_2$ is the major signaling lipid in the plasma membrane known to modulate numerous cellular functions by interacting with ion channels, receptors, and transporters. Notably, recent cryo-EM reconstructions of $GABA_A$ receptors ($GABA_A$Rs) revealed PI(4,5)$P_2$ bound to the α1 subunits.

In previous two-electrode voltage-clamp (TEVC) experiments, α1β3γ2L mouse $GABA_A$Rs (m$GABA_A$Rs) were heterologously expressed in *Xenopus* oocytes with a voltage-sensing phosphatase (VSP) to observe the effect of enzymatically depleting endogenous PI(4,5)$P_2$ on the channel activity. Our findings suggested that α1β3γ2L m$GABA_A$Rs exhibit resistance to the depletion of PI(4,5)$P_2$ by VSP, whereas the introduction of a neutralizing mutation into K311, one of the lysine residues indicated in PI(4,5)$P_2$ binding, led to a rapid decay of current ($I_{GABA}$) upon VSP activation. This result suggested that the wild-type channels have extremely high affinity to PI(4,5)$P_2$, and this depended on residue K311. However, it still remains unclear whether activities of α1β3γ2L m$GABA_A$Rs require PI(4,5)$P_2$.

To address this point, we introduced an approach that uses optogenetic manipulation of the PI(4,5)$P_2$ binding residue. This technique, called caged lysine method, enables us to perform precise photochemical control of PI(4,5)$P_2$ binding at the single amino acid level over ion channel activities without depleting PI(4,5)$P_2$ in the same cells.

Here, we show our results from incorporating photocaged lysine into the K311 of the α1 subunits in α1β3γ2L m$GABA_A$Rs. Uncaging the photocaged lysine introduced into K311 showed a robust increase of $I_{GABA}$ immediately after UV irradiation. To verify that uncaging the channel complex restores the nature of PI(4,5)$P_2$ binding of wild-type channels, we used VSP to deplete endogenous PI(4,5)$P_2$ before and after UV irradiation. Before UV irradiation, m$GABA_A$Rs were sensitive to VSP-mediated PI(4,5)$P_2$ depletion, whereas after UV irradiation, the uncaged channels were no longer sensitive to the PI(4,5)$P_2$ depletion. These findings highlight the crucial role of residue K311 in PI(4,5)$P_2$ binding in m$GABA_A$Rs, suggesting PI(4,5)$P_2$ plays an important role in modulating the channel activities of m$GABA_A$Rs. Furthermore, our results provide the first example of utilizing caged amino acid as a useful tool to study ligand-regulation of ion channels, in particular, with high ligand binding affinity.

### [3P-032]

### Significance of Glu/Gln in the sugar-binding pocket on $H^+$ transport in $Na^+$-glucose cotransporters

*Satoko Ichihara[1], Kazuyo Kamitori[1,2], Yuichiro Fujiwara[1,2,3] ([1]*Molecular Physiology and Biophysics, Faculty of Medicine, Kagawa University, Kagawa, Japan,* [2]*International institute of Rare Sugar Research and Education, Kagawa University, Kagawa, Japan,* [3]*Physiology and Biophysics, Graduate School of Biomedical and Health Sciences, Hiroshima University*)

Sodium-glucose cotransporters (SGLTs) are expressed in the intestine and kidney and are responsible for sugar absorption. Human SGLT1 (hSGLT1) is responsible for membrane transport of glucose and galactose in small intestinal epithelial cells and its genetic mutation results in malabsorption. hSGLT1 is also known to transport capacity of H+. hSGLT3 is expressed in neurons of the small intestine and is reported to function as a glucose sensor that does not transport sugar but causes membrane depolarization in response to glucose. Moreover, it was reported that in hSGLT3, mutation of Glu457, which constitutes the sugar binding pocket, to Gln corresponding to hSGLT1 results in the acquisition of sugar transport capacity. In this study, we focused on this residue and compared sugar transport, pH sensitivity, and H+ transport in hSGLT1 and hSGLT3.Each hSGLTs was expressed in Xenopus oocytes and sugar-induced Na+ current were measured in neutral and acidic conditions. In the presence of Na+, hSGLT1 type (the residue in its sugar-binding pocket is Gln457) showed only a slight change in current amplitude in response to changes in external pH, whereas hSGLT3 type (Glu457) exhibited an increase in current under acidic conditions. Next sugar-dependent H+ currents under Na+-free conditions were analyzed. In hSGLT1 type, H+ currents could be observed in neutral conditions and its amplitude increased under acidic conditions. In contrast, in hSGLT3 type, no or only weak sugar-dependent H+ current could be observed in neutral conditions, and sugar-induced H+ current were increased under acidic conditions. These results suggest that hSGLT3 type is more sensitive than hSGLT1 type, to the pH of the external fluid, and dramatically increase the H+ transports under acidic conditions. Taken together, hSGLT1 and hSGLT3 types are H+-permeable and that the amino acid residues that make up the sugar binding pocket affect pH sensitivity and H+ transport capacity, producing functional differences among the SGLT family. Although hSGLT3 has been reported to be a possible glucose sensor, its molecular mechanism and physiological role are still unclear. The results of this study revealed a relationship between the glucose-sensing function of SGLT and pH. This suggests that SGLT may play an important role in glucose transport and glucose reception during acidosis, such as ketoacidosis.

### [3P-033]

### Structural determinants of the direct inhibition of GIRK channels by Sigma-1 receptor antagonist

*Chang LIU[1,2], I-Shan Chen[1,2,3], Michihiro Tateyama[1,2], Yoshihiro Kubo[1,2] ([1]*Div. Biophys and Neurobiol, NIPS, Okazaki, Japan,* [2]*Physiol Sci, SOKENDAI, Hayama, Japan,* [3]*Dept. Pharmacol, Wakayama Med Univ, Wakayama, Japan*)

G-protein-gated inward rectifier $K^+$ (GIRK) channel is known to be activated by the $G_{\beta\gamma}$ subunit released from the stimulated $G_i$-coupled receptors. There are four subfamily members (GIRK1, GIRK2, GIRK3, GIRK4) and they can form functional homotetramers or heterotetramers which are involved in various physiological functions, such as the regulation of excitability of cardiomyocytes and neurons. BD1047 is one of the representative antagonists of multi-functional sigma-1 receptor (S1R). In the analysis of the effect of BD1047 on the inhibition of Gi-coupled receptors by S1R using GIRK channel as an effector, we observed that BD1047 directly inhibits the GIRK channel current even in the absence of S1R. Thus, we aimed to clarify the effect of BD1047 on GIRK channels as well as the structural determinants and observed the following results: (1) BD1047 directly inhibits the current of GIRK channels in *Xenopus* oocytes. It has a remarkable inhibition effect on GIRK4 channel and a weak inhibition effect on GIRK2 channel. (2) BD1047 also inhibits the ACh-induced native GIRK current in isolated rat atrial myocytes. (3) A GIRK4-GIRK2 chimera which contains only the proximal cytoplasmic N-terminal of GIRK4 showed a strong inhibition effect by BD1047. The Leu77 residue on the N-terminal of GIRK4 is critical for the inhibition effect. (4) Molecular docking analysis indicated the importance of the Leu77 for the BD1047 docking to GIRK4. (5) The activator of GIRK channel, ivermectin, competes with BD1047 at Leu77 on GIRK4. This study provides us with a novel inhibitor of GIRK channel and information for the development of pharmacological treatment to GIRK4-associated diseases.

### [3P-034]

### Interaction sites required for the KCNQ1 channel inhibition by KCNE4

*Minami Nakamura[1], Go Kasuya[1], Koichi Nakajo[1] ([1]*Div. of Integrative Physiol., Jichi Med Univ, Tochigi, Japan*)

KCNQ1 is a voltage-gated potassium (Kv) channel. Calmodulin (CaM) and KCNE proteins are known to be its auxiliary subunits and modulate its function. Human has five types of KCNE genes, as KCNE1-KCNE5. KCNE4 inhibits KCNQ1 currents in expression system such as *Xenopus* oocytes. Previous reports suggest that the interaction between the intracellular tetraleucine (LLLL) motif of KCNE4 and CaM is critical for KCNQ1 channel inhibition. However, it is not known where KCNE4 interacts with KCNQ1. Since the S1 segment in KCNQ1 is one of the transmembrane binding sites for KCNE3 in the Cryo-EM KCNQ1-KCNE3 complex, we evaluated whether those binding sites were also critical for inhibition by KCNE4. We mutated amino acids residues of the S1 segment to smaller alanine (A) or larger tryptophan (W). We also examined some intracellular residues, such as F364, I368, and P369, which are close to KCNE3 in the KCNQ1-KCNE3 structure. Nevertheless, all those KCNQ1 alanine or tryptophan mutants did not attenuate the current inhibition by KCNE4, suggesting that these amino acids may not be critical for the inhibition by KCNE4. Therefore, we next focused on the C-terminus region of KCNE4. Some deletion mutants attenuated the inhibition, suggesting that the C-terminus region may be more critical for the current inhibition than the transmembrane region. Therefore, the binding of KCNE4 might differ from the cryo-EM structure of the KCNQ1-KCNE3 complex.

### [3P-035]

### HCN channel activation induced by Hydrogen sulfide ($H_2S$)-evoked Nitric oxide (NO) production in cultured rat dorsal root ganglion neurons.

*You Komagiri[1] ([1]*Department of Physiology, School of Medicine, Iwate Medical University, Japan*)

In animal models of neuropathic pain, it has been reported that the expression and activity of HCN channels and $H_2S$ synthases increase. We previously reported that an $H_2S$ donor (NaHS) shifted the voltage-dependence of HCN channel current (*Ih*) toward positive potential via NO synthase and guanylate cyclase activity. In this study, we confirmed whether NO production is indeed involved in the effects of the $H_2S$ donor on *Ih* activation. NO fluorescence imaging using the fluorescent dye DAF-2 demonstrated a significant enhancement of intracellular NO production after exposure to the $H_2S$ donor in cultured DRG neurons. In addition, we investigated the effect of L-cysteine, a substrate for the $H_2S$ producing enzymes, on the activation of HCN channels. The application of L-cysteine also shifted the voltage dependence of *Ih* in a positive direction by about 10 mv, as did the addition of the $H_2S$ donor. These results suggest that not only exogenous $H_2S$ but also endogenously produced $H_2S$ activate HCN channels in the DRG neurons. We will further examine the contribution of endogenously produced $H_2S$ and NO production to HCN channel regulatory mechanisms.

### [3P-036]

### The role of astrocytic TRPA1 as an oxygen sensor in higher brain functions

*Akito Nakao[1], Hideto Oota[1], Yasuo Mori[1] ([1]*Department of Synthetic Chemistry and Biological Chemistry, Graduate School of Engineering, Kyoto University*)

Acute hypoxia sensors are essential for maintaining oxygen homeostasis within aerobic organisms. This is especially important for the brain, which is particularly vulnerable to oxygen deprivation due to high energetic demand. Our group have previously reported that astrocytic TRPA1 channels in the brainstem respiratory center selectively monitor moderate hypoxia, which can occur under physiological conditions, and partly contribute to respiratory plasticity. To examine the role of astrocytic TRPA1 as an oxygen sensor in higher brain functions, we conducted behavioral screening of astrocyte-specific *Trpa1* knockout mice. Altered anxiety- and depression-like behaviors were observed in astrocyte-specific *Trpa1* knockout mice through several tests. Fear conditioning and Barnes maze tests showed altered memory in the absence of astrocytic TRPA1. These results suggest that astrocytic TRPA1 channels as a physiological hypoxia sensor are involved in emotionality and memory. This presentation will focus on the behavioral phenotypes of astrocyte-specific *Trpa1* knockout mice and discuss the role of astrocytic TRPA1 as an acute sensor for physiological hypoxia in higher brain functions.

### [3P-037]

### Quantitative analysis of biocytin-labeled taste cell numbers in response to sweet substances

*Katsuhiro Kaetsu[1,2], Yoshiki Nakao[1], Hiroyuki Nakagawa[3], Yoshitaka Ohtubo[1] ([1]*Grad. School of Life Sci. and Syst. Eng., Kyusyu Inst. of Tech.*, [2]*Dept. of Chem. and Biol. Eng., Natl. Inst. Tech., Sasebo Col.*, [3]*Div. of Biol., Earth Syst. Sci., Faculty of Sci., Fukuoka Univ.*)

Taste cells express G-protein-coupled taste receptors and generate oscillating receptor potentials with action potentials in response to taste substances applied to their apical membranes, whereas they exhibit membrane depolarization when their basolateral membranes are exposed to high-$K^+$ stimuli. This membrane depolarization induces the openings of adenosine triphosphate (ATP)-permeable channels, such as calcium homeostasis modulator 1 and 3 channels and connexin hemichannels, to transfer taste information from the taste cells to the taste nerves. Because ATP-permeable channels form large pore that allow the transport extracellular biocytin into the cytosol, we investigated the numbers of biocytin-labeled cells per fungiform taste bud following apical membrane exposure to 30 mM saccharin (Sac). Compared to deionized water stimuli on the apical membrane, significant increases in biocytin-labeled cells were observed following the application of Sac. No significant differences in the number of phospholipase Cβ2 (PLCβ2)-immunoreactive type II cells were observed between the Sac and deionized water stimuli. After exposure to Sac, biocytin-labeled cells were rarely observed in PLCβ2-immunoreactive cells. In contrast, almost all PLCβ2-immunoreactive cells were labeled with biocytin after exposing the basolateral membrane to 150 mM KCl solution. Although a number of biocytin-labeled cells increased after exposure to Sac, these cells were not immunoreactive to PLCβ2 and synaptosomal-associating protein-25 (SNAP25), suggesting that Sac-induced transmitter release from type II cells may cause membrane depolarization on the PLCβ2 and SNAP25 non-immunoreactive cells that open biocytin-permeable hemichannels. Furthermore, these results suggest that oscillating receptor potentials limit the opening of biocytin-permeable (i.e., ATP-permeable) channels in PLCβ2-immunoreactive type II cells. By controlling the voltage-dependent opening of ATP-permeable channel, type II cells may maintain cellular homeostasis when transmitting afferent taste information.

### [3P-038]

### Exploration of novel TRPV1 inhibitors with a focus on the mechanism of skin irritation

*Masayuki Takaishi[1,2], Maori Kono[1,2], Makoto Tominaga[3] ([1]*mandom corp.*, [2]*Laboratory of Advanced Cosmetic Science, Graduate School of Pharmaceutical Sciences, Osaka University*, [3]*Exploratory Research Center on Life and Living Systems, National Institutes of Natural Sciences*)

TRPV1 is known to be one of the factors to mediate inflammatory pain, considered to play important roles in itch signaling and reported to be one of the factors to induce skin irritation. However, it is not clear how TRPV1 acts as a molecular sensor for skin irritation through signaling pathways activated by intracellular calcium increases or thereby depolarizing the membrane potentials. In addition, it is necessary to design a new TRPV1 evaluation system considering epithelial barrier functions since TRPV1 is located in the lower layers of epithelial cells. Furthermore, although many studies have focused on TRPV1 as a target for analgesics and many TRPV1 antagonists have been found, there are many safety issues remained to be solved such as side effects. Finding a new way searching for safe TRPV1 antagonists must be a powerful tool to develop new analgesic components with a new concept. In our study, we constructed a co-culture system of HEK293 cells stably expressing human TRPV1 (hTRPV1) with a three-dimensional skin model having a barrier function. First, we selected appropriate scaffold proteins to keep appropriate cell proliferation and functions. hTRPV1 cells embedded in a collagen-gel maintained original cell proliferation and functions. Next, we screened new indicators for TRPV1-dependent downstream releasing factors. As a result, we found a MCP protein which is a member of the CC chemokine family proteins and released in a hTRPV1-activity-dependent manner, and the MCP family proteins are known to be released in the excisional biopsy of human skin.

Then, we established a functional screening system searching for hTRPV1 antagonists with around 20,000 kinds of naturally-derived compounds. First, changes in intracellular $Ca^{2+}$ levels were examined for the functional screening, and MCP family protein concentrations in the medium were measured in the second screening. The first screening identified nine compounds some of whose $IC_{50}$ values showed lower values compared to $IC_{50}$ values for menthol, which is a naturally-derived hTRPV1 antagonist. Moreover, their inhibitory activities for TRPV1 were confirmed using a patch-clamp method. Additionally, these compounds inhibited the MCP family proteins as well. Thus, we identified several compounds, which might be strong candidates for novel analgesics.

### [3P-039]

### Development of membrane tension-clamp method in contact bubble bilayer

*Yuka Matsuki[1], Masako Takashima[2], Toshiyuki Yoshida[3], Masayuki Iwamoto[2], Shigetoshi Oiki[4] ([1]*Dept. Anesth. Reanimatol., Univ. Fukui Fac. Med. Sci.*, [2]*Dept. Mol. Neurosci., Univ. Fukui Facult. Med. Sci.*, [3]*Div. Engin. Info. Sci., Univ. Fukui. Facult. Eng.*, [4]*Biomed. Imag. Res. Cent., Univ. Fukui*)

Membrane tension varies substantially in biological membranes, affecting the activity of membrane proteins, including ion channels. However, measurement and control of membrane tension by the patch-clamp technique remain semi-quantitative. We have developed the contact bubble bilayer method (CBB), allowing quantitative tension evaluation for investigating the effects of membrane tension on ion channels. Here, we developed a membrane tension-clamp method in which the membrane tension is stably maintained via a feedback control system, permitting stable single-channel current recordings under constant tension. In the CBB method, a lipid bilayer is formed by contacting two bubbles whose surface is covered with a phospholipid monolayer. The bilayer tension ($\gamma_{bi}$) is evaluated from the bubble pressure and the bubble image, involving the bubble radius (R) and the contact angle (θ) between two bubbles. While the bubble pressure is continuously monitored via a fine pressure gauge, the geometrical (R and θ) values must be extracted from the bubble image in real-time using an image analysis program. Accordingly, to establish the tension-clamp system, we first established a real-time monitoring system of the bubble geometry by encoding an online program, which integrated the bubble pressure to evaluate the $\gamma_{bi}$ value automatically. In parallel, the bubble pressure was finely controlled by a stepping motor. Then, the measured bilayer tension was served for the feedback controller, yielding an output to fine-tune the stepping motor. To examine the stability of the tension-clamping, the membrane tension was maintained at an arbitrary tension value (2-10mN/m) for minutes and then changed to another tension value. We revealed that the bilayer tension was stable for more than ten minutes. Furthermore, we examined the system in the presence of a tension-sensitive KcsA potassium channel, recording single-channel currents simultaneously. We demonstrated that the open probability of the channel exhibited stepwise changes in parallel to the bilayer tension.

### [3P-040]

### Multiple pathways regulating TRPM2 activity at body temperature.

*Makiko Kashio[1], Satoru Masubuchi[2], Makoto Tominaga[1,3] ([1]*Div. Cell Signaling, NIPS*, [2]*Dep Physiol, Aichi Med Univ*, [3]*Thermal Biology Group, ExCELLS*)

Transient receptor potential (TRP) ion channels constitute a superfamily of large ion channels that are activated by a wide range of chemical, mechanical and thermal stimuli. TRP channels having temperature sensitivity are called thermo-TRPs, and each shows a characteristic temperature threshold for its activation. Therefore, regulation of temperature thresholds is thought to be crucial for their functional regulation.TRPM2 is one of thermo-TRPs working as a non-selective cation channel. TRPM2 is expressed in various tissues including brain, immunocytes and pancreatic β-cells where TRPM2 is continuously exposed to core body temperature. TRPM2 activity at body temperature could be regulated by endogenous factors such as $NAD^+$ metabolites and $Ca^{2+}$ in the cytosol. We have clarified that these endogenous TRPM2 regulators affect temperature thresholds for TRPM2 activation. TRPM2 expressed in HEK293T cells showed temperature thresholds of around 47°C in the absence of any endogenous cytosolic factors mentioned above. Cytosolic adenosine diphosphate ribose (ADPR) and $Ca^{2+}$ lowered temperature thresholds for TRPM2 activation in a concentration dependent manner. TRPM2 phosphorylation by protein kinase C (PKC) counteracted the effect of cytosolic $Ca^{2+}$, and completely abolished $Ca^{2+}$-dependent reduction of TRPM2 thresholds. Surprisingly, TRPM2 activation with another $NAD^+$ metabolite o-acetyl ADPR (OAADPR) lacked the threshold regulation by cytosolic $Ca^{2+}$. These results suggest that multiple environmental factors coordinately determine temperature thresholds for TRPM2 activation reflecting cellular metabolism. We'd like to discuss possible physiological roles of each regulatory pathway.

### [3P-041]

### Molecular mechanisms of therapeutic drugs for the expression defects of the CFTR mutant most common among Caucasian Cystic Fibrosis patients also rescuing different CFTR mutants found in Japanese patients

*Hikaru Sohma[1], Rio Kimishima[1], Hiroshi Nakagawa[2], Tadaomi Furuta[3], Hiroshi Ishiguro[4], Kanako Nakao[1], Yoshiro Sohma[1] ([1]*IUHW*, [2]*Chubu Univ.*, [3]*Tokyo Inst. Tech.*, [4]*Nagoya Univ.*)

Cystic Fibrosis (CF) is the most popular, life-shorten, inheritance disease in Caucasians, which is caused by function-loss mutations in Cystic Fibrosis Transmembrane conductance Regulator (CFTR). The most frequent mutation in Caucasians is a deletion of Phenylalanine508 (*Δ*F508) classified into the class II (trafficking defect). Recently a few chemical chaperons (correctors) for rescuing the *Δ*F508 mutant from the trafficking defect were developed by Vertex Inc and approved by FDA in USA. Especially the FDA granted Vertex Inc. approval for a game-changer drug "Trikafta", a combination of "C1" corrector, tezacaftor and "C2" corrector, elexacaftor and a channel function potentiator, ivacaftor in 2019.

CF is very rare in Japanese and its CFTR mutation profiles are different from those in Caucasians. At present, twenty-two mutations were identified from twenty-four Japanese CF patients definitely diagnosed. We have investigated the potential therapeutic effects of the Vertex drugs on the top-three frequent class II Japanese mutations, H1085R located in Intra-Cellular Loop 4 (ICL4), L441P in Nucleotide Binding Domain 1 (NBD1) and Q98R in Membrane Spawning Domain 1 (MSD1).

We found that a combined application of C1 and C2 correctors rescued H1085R and L441P from their trafficking defects and ivacaftor also significantly potentiated their channel function. Interestingly Q98R could be rescued by a relative low concentration sole application of C1 correctors whereas ivacaftor failed to potentiate it channel function.

Recent Cryo-EM studies suggest that the Vertex correctors bound at the interface between MSDs and ICLs, which is far from even their original target F508 position as well as all the three Japanese mutation points. Based on these findings, it was suggested that the Vertex drugs reduced the molecular fluctuation of the whole CFTR molecule, not in the mutation specific way. Our preliminary molecular dynamics simulation study supports this idea.

### [3P-042]

### A comparative study of the human and mouse GluD2 *Lurcher* mutants reveals the role for upper part the M4 transmembrane helix on the activity of ionotropic glutamate receptors

*Masayuki Itoh[1], Wataru Kakegawa[1], Michisuke Yuzaki[1] ([1]*Department of Neurophysiology, Keio University School of Medicine*)

Ionotropic glutamate receptors (iGluRs), consisting of GluAs, GluKs, GluNs and GluDs, play vital roles at excitatory synapses in vertebrates. Unlike other iGluRs, GluDs do not form functional ion channels in heterologous cells *in vitro*, but play a role in synaptic formation by forming trans-synaptic complex with neurexin and cerebellins. However, a single amino acid mutation (A654T) in the third transmembrane region (M3), originally found in *Lurcher* (*Lc*) mice, caused GluD2 channels to open spontaneously. Previously, we reported that HEK293 cells expressing human $GluD2^{Lc}$ showed less than one-tenth of the leak current of those expressing mouse $GluD2^{Lc}$. Furthermore, our mutant study successfully identified a single amino acid (F831 in human, L831 in mouse) at the upper region of fourth transmembrane region (M4) as the responsible for the differential effect of the Lc mutation. In this study, we further investigated the structural basis for the differences in channel activity between human and mouse isoforms. First, we examined the $Ca^{2+}$-sensitivity of hGluD2Lc, as the current of mGluD2Lc has been reported to be potentiated by extracellular $Ca^{2+}$ ions. Unlike $mGluD2^{Lc}$, $hGluD2^{Lc}$ lacks $Ca^{2+}$-sensitivity, and introduction of a mouse-type mutation (F831L) into hGluD2Lc restored $Ca^{2+}$-sensitivity. We then examined the effects of analogous mutations in three other iGluRs (GluD1, GluK2, and GluA2). In the case of GluD1, the mutation at the corresponding site (GluD1F831L) enhanced the constitutively-active current of the *Lurcher*-like mutation in GluD1 (GluD1C645I/A654T). Similarly, in the GluK2 homomer, the mutation (GluK2I820F) produced a reduction in glutamate-gated current and changes in gating kinetics. In contrast, a similar mutation (GluA2V813F) had little effect on the current activated by glutamate in the GluA2 homomer. However, when GluA2 was co-expressed with CNIH2, an auxiliary subunit of AMPAR, this mutation significantly attenuated the gating modulation of GluA2 by CNIH2. The upper part of the M4 helix, together with pre-M1 and M3 forming the gate, constitutes a structure called the "gating-triad", which has been proposed to be a crucial site that links ligand sensing to the opening and closing of iGluR. Therefore, our findings indicate that GluD2 shares a common channel-gating machinery with other iGluRs, but hGluD2 may be difficult to gate.

### [3P-043]

### Analysis of molecular mechanism underlying STIM1-mediated suppression of $Ca_V1.2$

*Takuro Numaga-Tomita[1] ([1]*Shinshu University School of medicine, Department of Molecular Pharmacology*)

In smooth muscle cells, vasoconstrictor stimulation activates $G_q$-PLC pathway and induces $Ca^{2+}$ influx. These are mediated by several $Ca^{2+}$-permeable channels including store-operated $Ca^{2+}$ channels (SOCC) and voltage-dependent L-type $Ca^{2+}$ channel $Ca_V1.2$. It has been reported that ER membrane protein STIM1, the ER $Ca^{2+}$ sensor of SOCC, interact and inhibit $Ca_V1.2$ channel. However, the molecular mechanism of STIM1-mediated suppression of $Ca_V1.2$ remains elusive. In this study, we have analyzed STIM1-dependent $Ca_V1.2$ suppression by the patch clamp technique. It has been demonstrated that $Ca_V1.2$ is expressed as both full length and C-terminally truncated isoforms. C-terminally truncated $Ca_V1.2$ still interacts with distal C-terminus (DCT) which negatively regulates $Ca_V1.2$. STIM1 dependent suppression was recapitulated with reconstituted $Ca_V1.2$ ($Ca_V1.2$ delta1821+DCT), indicating that STIM1-dependent suppression requires $Ca_V1.2$ DCT sequence. According to the analysis of gating currents, membrane surface expression of $Ca_V1.2$ was severely suppressed by STIM1 overexpression. Although endogenous STIM1 also significantly suppressed $Ca_V1.2$ channel, the steady-state inactivation or activation was unaffected. Furthermore, $Ca_V1.2$ interacts physically with STIM1. The reason why STIM1 interact with $Ca_V1.2$ in the absence of TG is currently under investigation. These data suggest that STIM1 movement to the plasma membrane upon store-depeletion somehow induces the endocytosis of $Ca_V1.2$, which reduces the number of channels and whole cell $Ca_V1.2$ currents.

### [3P-044]

### Prostaglandin E2 receptor 4-mediated TRPV4 upregulation may be involved in RANTES production in abdominal aortic aneurysm

*Hana Inoue[1], Tashiro Michiko[1], Yokoyama Utako[1] ([1]*Department of Physiology, Tokyo Medical University*)

We previously reported that prostaglandin E2 receptor 4 (EP4) is overexpressed in vascular smooth muscle cells (VSMCs) in patients with abdominal aortic aneurysm (AAA) and that VSMC-specific EP4-overexpressing transgenic (EP4tg) mice are highly susceptible to AAA. Hypertension is a major risk factor for AAA, however, the effect of EP4 signal on mechano-sensing ion channel signaling has not yet been investigated. In the present study, we found that a mechano-sensitive ion channel, *Trpv4* expression is significantly increased in EP4tg-VSMC compared to nontransgenic-VSMC (5.3±1.4 folds, n = 5-6, p<0.05, Mann-Whiteney U-test). To investigate the pathophysiological consequences of TRPV4 activation, we performed transcriptome analysis in VSMC treated with a TRPV4 agonist, GSK1016790A (10 nM). We found that a transcriptional factor, *Nfatc2*, was significantly increased over 100-fold by TRPV4 activation. Western blotting confirmed that TRPV4 agonist stimulated nuclear translocation of NFATc2, suggesting that TRPV4 activation stimulated NFATc2-mediated transcription. To clarify downstream of the TRPV4-NFATc2 pathway, enrichment analysis was performed using the NFAT-related gene set (NFAT_Q4_01). *Ccl5* (RANTES), a chemokine that was reportedly increased in AAA patients' aortic walls, was markedly increased by TRPV4 activation in VSMCs. A Nfatc2 inhibitor, cyclosporine A (10 μM), completely inhibited the upregulation of *Ccl5* (n = 6, p<0.05, ANOVA with Bonferroni post-hoc comparisons), indicating that TRPV4-NFATc2 pathway was essential for the *Ccl5* increase. These results suggest that EP4 enhances TRPV4 activation by upregulation of TRPV4, thereby increasing NFATc2-mediated RANTES production in AAA.

### Poster

### [3P]
### Embryology, Regenerative Medicine, Development, Growth, Aging

March 30, 13:00 - 14:20, Poster Room

### [3P-045]

### Roles of mesenchymal stem cell derived extracellular vesicles on retinal cells and corneal neovascularization

*Hisaki Hayashi[1], Motohiko Sato[1] ([1]*Department of Physiology, Aichi Medical University*)

Mesenchymal stem cells (MSC) have been intensively investigated as a cell–based therapy and regenerative medicine. Extracellular vesicle (EVs), derived from MSC has been reported to have anti-oxidant potential, however, roles of EVs in age related macular degeneration, induced by oxidative stress in eyes, has not been reported yet. Purpose of this study is to analyze the effect of bone marrow MSC derived EVs on recovery from oxidative stress induced cellular damage in eyes. First, EVs from bone marrow derived MSC was isolated by combination of filtration and sucrose gradient ultracentrifuge method, and the quality was evaluated. Immunocytochemistry and flow cytometry indicated 100% of cultured endothelial cells incorporated EVs labeled with fluorescent labeling reagent. Next, purified MSC-EV's biological activity in ocular cells and tissue were analyze. Hydrogen peroxide induced cytotoxicity in cultured retinal cells were attenuated by MSC-EV treatment via in-vitro LDH assay. MSC-EV injection in the vitreous of experimental mouse CNV models demonstrated change of microglial localization in the retinas of lase-induced CNV model. We will discuss the role of MSC-EVs on retina and induced CNV in the session.

### [3P-046]

### Effect of ZFHX3 on oligodendrocyte differentiation

*Cha-Gyun Jung[1], Shiori Tominaga[1], Yu Nishimura[1], Mustika Dewi[1], Shinya Ueno[1], Naoki Tajiri[1], Hideki Hida[1] ([1]*Department of Neurophysiology and Brain Science, Nagoya City University Graduate School of Medical Sciences*)

Oligodendrocytes (OLGs) are generated from oligodendrocyte precursor cells (OPCs) that exit the cell cycle and differentiate into OLGs throughout the central nervous system at predictable developmental ages. However, the signal transduction mediators that underlie these events remain poorly understood. We previously demonstrated that the transcription factor ZFHX3 (also known as ATBF1) induces cell cycle arrest associated with neuronal differentiation; however, its role in OLG differentiation remains unknown. In this study, we investigated the role of ZFHX3 in OPC cell cycle arrest and its impact on OLG differentiation. ZFHX3 was predominantly expressed in the cytoplasm of proliferating OPCs. In contrast, it was primarily localized in the nuclei of differentiated OLGs, where ZFHX3 expression was higher than that in OPCs. ZFHX3 knockdown in OPCs by siRNA resulted in an increase in the number of BrdU- and PDGFR-α-positive cells, along with increased levels of PDGFR-α mRNA and protein. Furthermore, ZFHX3 knockdown in OPCs decreased the expression of myelin-related markers, including CNPase and MBP, and reduced the outgrowth of myelin membrane sheets without affecting cell death. ZFHX3 knockdown in OPCs also decreased the CDK inhibitors (p21 and p57) while increasing Id4 levels at both mRNA and protein levels. We propose that nuclear ZFHX3 plays an important role in OLG differentiation and maturation by arresting OPC cell cycle.

### [3P-047]

### Calcium-induced reorganization of cortical actin cytoskeleton in mammalian oocytes at fertilization

*Hideki Shirakawa[1], Fumiya Kato[1], Ryo Yonekura[1] ([1]*The University of Electro-Communications*)

Actin filaments (F-actin) are densely localized beneath the plasma membrane of mammalian oocytes and involved in various cellular events at fertilization. We investigated the dynamics of cortical F-actin organizations visualized in mouse oocytes with F-actin-binding fluorescent probes, in relation to the changes in intracellular $Ca^{2+}$ concentration induced by sperm or sperm-borne egg-activating protein PLCζ. Repetitive transient $Ca^{2+}$ increases induced by PLCζ caused significant increases in fluorescence intensity of F-actin probe at the oocyte cortex. These increases nearly corresponded to the changes in $PIP_2$, both spatially and temporally, and were suppressed by inhibitors of actin polymerization, suggesting that they were due to, at least in part, $PIP_2$-dependendent facilitation of F-actin polymerization. When a fertilizing sperm attached to the oocyte surface, the cortical F-actin around the attachment site started gathering toward the sperm head. Interestingly, rapid accumulation of F-actin around the sperm head was observed upon the first sperm-induced $Ca^{2+}$ increase, and then the fused sperm head began to move deeper into the cytoplasm of the oocyte, suggesting that the $Ca^{2+}$-induced local reorganization of cortical F-actin drives the initial step of sperm incorporation. In the presentation, the mechanism of F-actin reorganization around the fertilizing sperm will be discussed, based on some experiments with F-actin inhibitors.

### [3P-048]

### Mechanism of regionalization of vasopressin・oxytocin neuronal nuclei in the hypothalamus.

*kanako saito[1], toshiki kameyama[1], yu kodani[1], miho kawada[1], akira nakasima[2], hiroshi nagasaki[1] ([1]*Department of Physiology I, Fujita Health University,* [2]*Department of Physiological Chemistry, Fujita Health University*)

In the hypothalamus, which is responsible for endocrine and autonomic regulation, neurons arise in the vicinity of the third ventricle surface during embryonic development to form a diverse neuronal nucleus by regionalization of homologous cells. The neuronal migration and the interaction with neighboring cells that may occur during migration are essential for the construction of functional neural networks in each of these neuronal nuclei. However, it remains unclear how neurons move, interact with neighboring cells, and subdivide regions. In this study, we focus on the mechanism that arginine vasopressin (AVP)/oxytocin (OXT) neurons projecting to the posterior pituitary are deliberately separated and localized in the paraventricular nucleus (PVN) and supraoptic nucleus (SON). First, we observed whether AVP/OXT neurons date and place of birth differed between PVN and SON, using EdU administration and in utero electroporation in pregnant mice. We found that both AVP/OXT neurons were born at 10-12 days of gestation(E10-12) in the hypothalamic ventricular surface, where neural stem cells divide and proliferate, and then migrate and localize in the PVN and SON regions. These result the PVN and SON, composed of AVP/OXT neurons, segregate into two different neuronal nuclei despite no significant difference in their birth situation, suggests that some molecular signal may be involved that promotes segregation between the PVN and SON regions. Therefore, we focused on the mechanism at the molecular level. One of the candidate gene, Reelin, is known to regulate neuronal migration and layer formation in various regions of brain, and it is expressed in the vicinity of the PVN in the hypothalamus. We found Reelin expressed from the embryonic period when the hypothalamic nuclei begin to form in mouse. In Reelin-deficient mice, however, we did not recognize clear inhibition of segregation of PVN/SON, except that some cells migrating from PVN to SON showed stagnation in migration. These results suggested that Reelin may act on AVP/OXT neurons to regulate certain parts of the PVN/SON separation.

### [3P-049]

### Postnatal changes of $GABA_A$ receptor-mediated currents and glycine receptor-mediated currents in CA3 pyramidal cells of the cultured hippocampal slice

*Kirika Takenaka[1], Masahiro Mori[1] ([1]*Kobe Univ. Facul. Health Sciences*)

Gamma-aminobutyric acid (GABA) and glycine are major inhibitory neurotransmitters in the central nervous system. Their ligand-gated chloride channel receptors, $GABA_A$ receptors and glycine receptors are expressed in the hippocampus, where fast GABAergic synaptic transmission but fast glycinergic synaptic transmission has been reported. We studied if functional $GABA_A$ receptors or glycine receptors might be involved in the development of hippocampal neurons. Outward current responses to pressure application of GABA or glycine (0.3 mM, 0.5-1 s) were recorded under voltage-clamp at 0 mV in CA3 pyramidal cells of the cultured hippocampal slices made from newborn rats (postnatal day 0: P0). At 7 days in vitro (DIV 7), the peak amplitude of $GABA_A$ receptor-mediated currents ($I_{GABA}$) was 2758 ± 473 pA (n=5) and the peak amplitude of glycine receptor-mediated currents ($I_{GLY}$) was 1146 ± 244 pA (n=9). We cultured the hippocampal slices with a $GABA_A$ receptor blocker, gabazine (10 µM) or a glycine receptor blocker, strychnine (10 µM) for 14 days. The CA3 pyramidal cells of those slices were labeled with biocytin to analyze morphological parameters such as total dendritic length and number of branching points. The mechanisms, by which $GABA_A$ receptors or glycine receptors could affect the development of hippocampal neurons will be discussed.

### [3P-050]

### The defect in nonsense-mediated mRNA decay pathway induces premature aging in zebrafish

*Hiroshi SHIRAISHI[1], Shaohong LAI[1], Wulan SEBASTIAN[1], Hongxia WANG[1], Nobuyuki SHIMIZU[1], Reiko HANADA[2], Toshikatsu HANADA[1] ([1]*Oita University Faculty of Medicine, Department of Cell Biology,* [2]*Oita University Faculty of Medicine, Department of Neurophysiology*)

The nonsense-mediated mRNA decay (NMD) is one of the quality control mechanisms for mRNA, recognizing and degrading mRNA containing a premature termination codon (PTC) resulting from mutations. SMG9 forms a complex with SMG1 and control this pathway. Patients with mutations in *SMG9* have been reported to develop heart and brain malformation syndromes (HBMS). However, the pathogenesis of HBMS and the physiological role of SMG9 in vivo are not fully understood. We generated *smg9*-deficient zebrafish using the CRISPR/Cas9 system. In *smg9*-deficient zebrafish, a decrease in phosphorylation levels of UPF1, a target of the SMG1 complex, and reduced expression of NMD endogenous target genes were observed, suggesting the positive regulatory role of SMG9 in NMD. Moreover, *smg9*-deficient zebrafish displayed abnormalities in brain formation and cardiac function. Hence, the *smg9*-deficient zebrafish was considered a good animal model for HBMS. Furthermore, intriguingly, the *smg9*-deficient zebrafish gradually exhibited an aging-like phenotype after three months of age, resulting in significantly decreased survival rates compared to wild-type zebrafish. Upon further analysis of aging-like phenotype, increased senescence-associated beta-galactosidase activity and elevated expression of cellular senescence-related genes were confirmed in the *smg9*-deficient zebrafish. In order to investigate how Smg9 deficiency causes premature aging phenotypes, we focused on the increased expression of smox (spermine oxidase), one of the NMD endogenous target genes. The premature aging phenotype observed in *smg9*-deficient zebrafish was also observed by smox mRNA injection to wild-type zebrafish, and smox inhibitors alleviated the *smg9*-deficient phenotype, suggesting that the *smg9*-deficient phenotype is partly due to elevated smox expression caused by NMD dysfunction.

### Poster

### [3P]
### Muscle

March 30, 13:00 - 14:20, Poster Room

### [3P-051]

### Ketogenic diet feeding shifts fast muscle fiber composites to slow in extensor digitorum longus muscle of sedentary sema3A-floxed female mice

*hiroko hagiwara[1], chiaki kakehashi[1], toshiya funabashi[1] ([1]*St.Marianna University School of Medicine. Department of physiology*)

Ketogenic diet (KD), an extremely high-fat diet with extremely low carbohydrates, reportedly changes the energy metabolism properties of skeletal muscle. We described recently that 4-week KD consumption improves extensor digitorum longus (EDL) muscle aerobic capacity without obstructing muscle contractile function, as reflected by a myosin heavy chain (MyHC) composition shift from IIb to IIx in male rats by increasing Sema3A. This study examines whether KD alters the composition of MyHCs in female Sema3A-floxed mice. After being fed a control diet (CON, 10% fat, 10% protein, 80% carbohydrate) or a KD (90% fat, 10% protein, 0% carbohydrate) for 4 weeks, we analyzed the soleus, EDL, and tibialis anterior (TA). These muscles' MyHCs compositions were found using immunocytochemical method as types I, IIa, and IIb, respectively, using BA-D5, SC71, and BF-F3 antibodies. No staining were presumed to be Type IIx myofibers. Results show that KD significantly raised blood β-hydroxybutyric acid (CON 0.69±0.11 SEM, KD 1.9±0.22 mM). Between CON and KD, no significance was found for type I or type II MyHCs proportions in soleus or TA. However, the proportion expression of MyHC type IIx in KD (33.5%±5.8 SEM) was significantly higher than in CON (18.7%±3.2). Expression of MyHC type IIb in KD (54.4%±7.3) was lower than that in CON (68.5%±2.6), but it was not significant. The findings demonstrate that the four-week KD induced a fast-to-slow shift among type II MyHC isoforms in EDL muscle of Sema3A-floxed female mice. We are examining the effects of Sema3A knocked down by adeno-associated virus on the composition of MHCs in the EDL.

### [3P-052]

### Cast immobilization in adult rats suppresses satellite cell proliferation *in vitro*

*Yung-Li HUNG[1,2], Shuichi Machida[2] ([1]*Japan Society for the Promotion of Science*, [2]*Juntendo University*)

Skeletal muscle undergoes rapid and profound atrophy in response to decreased mechanical loading, e.g., in limb immobilization, hindlimb suspension, bed rest, and spaceflight. However, skeletal muscle also exhibits a remarkable ability for regrowth. In mature or old rats, muscle atrophy occurs mainly through a loss in existing mass, including a reduction in a loss of myonuclei. It is likely that muscle satellite cells play essential role for successful regrowth from inactivity-induced muscle atrophy, since skeletal muscle fibers are terminally differentiated (postmitotic) and require the proliferation of muscle satellite cells to provide new myonuclei for increasing regrowth. However, to the best of our knowledge, there is no information on how the proliferative function of muscle satellite cells is affected in skeletal muscle atrophied by cast immobilization. The aim of this study was to examine the effects of cast immobilization on the proliferative function of muscle satellite cells *in vitro*. Female F344 rats (12-18 weeks old) were divided into two experimental groups: control (CON) and immobilized (IM). Rats in the IM group were subjected to cast immobilization of both lower extremities using casting-tape for 10 days. After casting immobilization, primary satellite cells were isolated from the quadriceps, gastrocnemius, soleus, and plantaris of the rats. The effect of cast immobilization on isolated satellite cell proliferation was examined using ki-67, BrdU, Pax7 and MyoD immunostaining. The ki-67-positive cell ratios were not significantly different between the CON and IM groups. The BrdU-positive cell ratio was significantly decreased in the IM group compared with the CON group. The Pax7-positive/MyoD-positive cell ratio was significantly decreased in the IM group compared with the CON group, while the Pax7-negative/MyoD-positive cell ratio was dramatically increased in the IM group. These results suggest that cast immobilization in adult rats may reduce proliferative function of muscle satellite cells and could induce myogenic differentiation progression.

### [3P-053]

### Cesium impact on C2C12 murine skeletal muscle cells: Proliferation and its morphology

*Daisuke Kobayashi[1], Tooru Funyu[1], Youichiro Takahashi[1], Akihiro Hazama[1]
([1]*Fukushima Medical University, Sch.Med.Dept.Cell. Integrat. Physiol.*)

An alkali metal cesium is thought to be distributed uniformly throughout the body rather than accumulating in specific organs when taken into the body. Skeletal muscle accounts for about 40% of the human body, and it is known that cesium taken into the body is also distributed in skeletal muscle, but there is little information on the effects of cesium on skeletal muscle cells. The aim of this study is to evaluate an effect of cesium on skeletal muscle cells. As a first step, we evaluated the effects of cesium on skeletal muscle cells proliferation. Cesium was added to murine C2C12 skeletal muscle cells in culture, and we measured cell proliferation and performed morphological observations. Cell proliferation was suppressed in a dose-dependent manner, and microscopic observation revealed cell volume swelling and cell nuclei increasing, as well as multinucleated cells. C2C12 myoblasts are able to fuse and to differentiate into myotubular cells in low nutrition condition. Cesium treatment conditions may mimic low nutrient conditions similar to differentiated cultures.

### [3P-054]

### CGRP-cAMP-dependent signal transduction pathways upregulate MyHC I mRNA through the activation of PKA and Epac1 in C2C12 cells.

*Yoshiaki Mori[1], Junko Yamaji[1], Reiko Hiroshima[1] ([1]*Dept of Rehabil Sci, Kansai Univ. of Welf Sci, Kashiwara, Japan*)

Our previous study using differentiated C2C12 cells indicated that myosin heavy chain type I (MyHC I) mRNA expression level was significantly increased by the application of calcitonin gene-related peptide (CGRP). Effects of CGRP have been studied in two skeletal muscle cell lines, rat L6 cells and mouse C2C12 cells. These cell lines appear to express CGRP receptors coupled to adenylyl cyclase activity. CGRP has also been identified in spinal motoneurons of several species and in the nerve terminals of the rodent neuromuscular junction. Therefore, we examined the contribution of cAMP dependent pathways on the upregulation of MyHC I mRNA levels in C2C12 cells. C2C12 cells were induced to differentiate to myotubes by medium exchange to D-MEM containing 2%FBS. The cells were incubated in D-MEM containing 2%FBS with chemical compounds at the beginning of differentiation and removed after 24hr, and were maintained in differentiation medium for 3 days. MyHC I mRNA expression levels were measured by the quantitative PCR method. MyHC I mRNA levels were significantly increased by the administration of isoprotelenole, forskolin, or 8-Br-cAMP. The effects of forskolin on MyHC I mRNA expression level were significantly inhibited by the co-administration of PKA inhibitor. Although the effects of forskolin on MyHC I mRNA expression level were not affected by the co-administration of exchange protein activated by cAMP 2 (Epac2) inhibitor, administration of exchange protein activated by cAMP 1 (Epac1) inhibitor significantly suppressed the increase in MyHC I mRNA caused by the administration of forskolin. Furthermore, MyHC I mRNA expression level was not affected by the application of cAMP response element binding protein (CREB) inhibitor. These results suggested that the upregulation of MyHC I mRNA level by the activation of CGRP-cAMP-dependent signal transduction pathways was involved in PKA and Epac1 mediated processes in C2C12 cells.

### [3P-055]

### Effects of Addition of MG132 and Chloroquine to C2C12 Myotubes on Signals Related to Muscle Protein Synthesis and Degradation

*Ryoto Iwai[1], Takanaga Shirai[2,3], Kazuki Uemichi[1,3], Tohru Takemasa[4] ([1]*Graduate School of Comprehensive Human Science, University of Tsukuba,* [2]*Faculty of Human Science, Kanagawa University,* [3]*Japan Society for the Promotion of Science,* [4]*Faclty of Health and Sports Sciences, University of Tsukuba*)

Skeletal muscle exhibits remarkable quantitative plasticity and is intricately regulated by the balance between muscle protein synthesis and breakdown. Akt/mTOR signaling cascade is well known to protein synthesis regulation, and its activation markedly increases muscle protein synthesis. Conversely, both the ubiquitin-proteasome system and the autophagy-lysosome pathway are among the most important intracellular proteolysis. The ubiquitin-proteasome system explicitly targets abnormal intracellular proteins for degradation, whereas the autophagy-lysosome pathway selectively and indiscriminately degrades intracellular proteins. Previous study suggests that MG132, an inhibitor of proteasome activity, into C2C12 myoblasts results in decreased protein synthesis. Our study aimed to elucidate the effects of these pathways on protein synthesis and associated degradation signaling in C2C12 myotubes.We used 0.1% DMSO or MG132 (10 μM), an inhibitor of proteasome activity, or chloroquine (10 μM), an inhibitor of lysosome activity, dissolved in this solvent in the medium of C2C12 myotubes on day 6 of differentiation induction (n=3 per group) and collected cells after 3 hours incubation, Western blot was used to quantify proteins related to muscle protein synthesis and degradation.

Compared to the DMSO group, the addition of MG132 and chloroquine significantly decreased the expression of puromycin labeled protein. On the other hand, the addition of MG132 and chloroquine did not affect the expression of proteins related to muscle protein synthesis and degradation, although the expression level of phosphorylated Akt was significantly lower with the addition of chloroquine. Furthermore, the addition of MG132 tended to increase ubiquitinated protein expression and significantly increased p62 expression, while chloroquine did not affect the expression levels of degradation substrates. More than 80% of intracellular protein degradation is dependent on the ubiquitin-proteasome pathway, suggesting that the labeled degradation substrates accumulate intracellularly due to inhibition of proteasome activity. In addition, muscle protein synthesis was significantly reduced by the addition of MG132 compared to chloroquine, indicating that the ubiquitin-proteasome pathway plays an important role in protein metabolism in muscle cells.

### [3P-056]

### Sarcomere structure analysis of in vivo slow-twitch muscle with retained blood supply: an x-ray diffraction study

*Naoya Nakahara[1], Hideki Yamauchi[1], Maki Yamaguchi[1], Tomonori Hayashi[1], Kazuhiro Hirano[1], Shigeru Takemori[1] ([1]*The Jikei University School of Medicine*)

We have developed an x-ray diffraction technique to analyze fine sarcomere structure of in vivo muscle with retained blood supply, which enabled analysis of contracting muscle without a sign of fatigue. Our first attempt was succeeded in fast-twitch muscle, extensor digitorum longus. Here, we report our recent effort to apply the technique to analyze sarcomere structure of slow-twitch muscle, soleus.

In general, slow-twitch muscle, such as soleus, resides close to the bone covered with layers of fast-twitch muscle of considerable thickness. Therefore, it is difficult to pull slow-twitch muscle retaining blood supply out from the layers of fast-twitch muscle on the path of x-ray.

X-ray diffraction patterns from soleus muscle of anesthetized 6-month female ICR mice were obtained at BL-6A in KEK, Tsukuba. Obtained diffraction patterns were superior quality showing high orders of reflections and layer lines. The myosin 1st layer line profile from soleus muscle with retained blood supply showed bimodal distribution as reported earlier researchers (Iwamoto et al., 2003, Ma et al. 2019) supporting the myosin heads are relaxed enough in the in vivo muscles. Equatorial 1,1/1,0 intensity ratios of in vivo muscle were lower than those of relaxing skinned muscle fibers as in the case of fast-twitch muscle suggesting myosin heads are more detached in the in vivo muscle.

In conclusion, we succeeded in obtaining fine x-ray diffraction patterns from in vivo skeletal muscle with retained blood supply, even in slow-twitch muscle. Slow-twitch muscle has been the focus of much attention in health sciences: for instance, disuse atrophy predominantly affects slow-twitch muscle. Course of atrophic change in sarcomere detected with x-ray diffraction would serve to find counter measures against muscle atrophy. As our next research step, we will elucidate atrophy process in a mouse model of disuse atrophy induced by cast immobilization.

### [3P-057]

### Characteristic features of abnormal nuclear shape in skeletal muscle from a mouse model of nuclear envelopathy

*Eiji Wada[1], Kotaro Ariga[1], Nao Susumu[1], Motoshi Kaya[2], Yukiko Hayashi[1] ([1]*Tokyo Medical University,* [2]*The University of Tokyo*)

Presence of abnormal shaped nuclei is a hallmark of nuclear envelopathies, which are the group of diseases caused by mutations in the genes encoding nuclear envelope proteins. Mutations in the lamin A/C gene (*LMNA*) cause several diseases including Emery-Dreifuss muscular dystrophy. A mouse model carrying H222P-*Lmna* mutation develop severe cardiomyopathy and mild skeletal myopathy, and abnormal nuclei were present in both cardiac and skeletal muscles. Primary cultured skeletal muscle cells from H222P mice proliferate and efficiently differentiate into myotubes, similarly to those from wild type mice. During proliferation, abnormal shaped nuclei are barely detectable. However, markedly deformed myonuclei are observed around 12.5% of total myonuclei on myotubes from H222P mice. Among them, 70% of deformed myonuclei has a bleb and string structure, and 30% of abnormal myonuclei show elongation form. We evaluate the expression of nuclear membrane proteins in these abnormal myounuclei. Localizations of lamin A/C and emerin are maintained in the bleb, string, and elongated part of myonuclei; however, lamin B1/B2, nesprin 1, and a nuclear pore complex protein are disappeared in these abnormal compartments. Although myonuclei are markedly deformed, deposition of γH2AX or apoptosis is rarely observed. These results represent that myonuclei of H222P mice have resistance to the DNA damage despite of marked morphological changes.

### [3P-058]

### Inhinitory effects of phorbol-12,13-dibutyrate on relaxation of skinned carotid artery

*Masaru Watanabe[1], Toshio Kitazawa[2] ([1]*Graduate School of Human Health Sciences, Tokyo Metropolitan University,* [2]*Graduate School of Agricaltural and Life Sciences*)

Background: Phorbol 12,13-dibutyrate (PDBu), a potent activator of protein kinase (PKC), is known to have various effects on smooth muscle contraction and relaxation cycle. A previous study presented that PDBu caused force development through activation of very slow cycling latch-bridge independent on phosphorylation level of myosin regulatory light chain (MLC20) phosphorylation (Hai and Kim. J Appl Physiol. 2005; 98:1356-65). Since relaxation process of skinned smooth muscle reflects "latch like state" (eg. Mihashi et al. J Smooth Musc Res. 2020; 56: 19-28), we examined whether PDBu affects relaxation process of beta escin skinned (cell membrane permeabilized) phasic taenia cecum and tonic carotid artery from guinea pig.
Results and Discussion: In skinned carotid artery, PDBu significantly inhibited force decay after Ca ion removal from the artificial intracellular solution at 1 micro M and higher. On the other hand, little effects of PDBu on relaxation process in skinned taenia cecum were observed. Regression analysis of the relaxation process (Mihashi et al., 2020) indicates that, in skinned carotid artery, PDBu suppressed the relaxation process through increasing the rate of formation of slow-cycling bridge after detachment of fast cycling-cross bridge, and also elongation of detachment of slow-cycling bridge. The present results strongly support the idea that protein kinase C contributes latch bridge formation in tonic smooth muscle.
COI; No

### Poster

### [3P]
### Oral physiology

March 30, 13:00 - 14:20, Poster Room

### [3P-059]

### Depolarization induced $Ca^{2+}$ mobilization in rat odontoblasts

*Madoka Seki[1], Maki kimura[2], Takehito Ouchi[2], Ryuya Kurashima[2], Yoshiyuki Shibukawa[2] (*[1]Tokyo dental college anesthesiology, [2]Tokyo dental college physiology*)

Stimulation applied to the dentin surface elicits activation of mechanosensitive ion channels and intracellular $Ca^{2+}$ signaling in odontoblasts, resulting in the release of ATP from pannexin-1 channel to the extracellular space. The released ATP acts as an intercellular transmitter from odontoblasts to trigeminal ganglion neurons, as well as from odontoblast to odontoblast. The intercellular communication is involved in generation of dentinal sensation and dentin formation. The cation influx via an activation of mechanosensitive ion channels may induce plasma membrane depolarization in odontoblasts. However, intracellular $Ca^{2+}$ signaling elicited by the plasma membrane depolarization in odontoblasts, and its participation to their cellular functions have remained unclear. In this study, we investigated intracellular $Ca^{2+}$ signaling pathway induced by plasma membrane depolarization in acutely isolated rat odontoblasts. Dental pulp slices were obtained from the incisors of newborn (3-9 days) Wistar rats. After enzymatic treatment, the primary cultured odontoblasts in the dental pulp slices were used for experiments within 24 hr culture period. Intracellular free $Ca^{2+}$ concentration ($[Ca^{2+}]_i$) was measured with fura-2 AM fluorecence imaging and expressed as the fluorecence ratio ($R_{F340/F380}$) at two excitation wavelengths of 380 nm and 340 nm. In the presence of extracellular $Ca^{2+}$, high $K^+$ (50 mM) solution, that induced depolarization, transiently increased $[Ca^{2+}]_i$ in odontoblasts, and did not show any desensitizing effects on the $[Ca^{2+}]_i$ increase by the third application. In the absence of extracellular $Ca^{2+}$, high $K^+$ solution also increased $[Ca^{2+}]_i$. The amplitude of the increase was significantly smaller than that in the presence of extracellular $Ca^{2+}$. In the absence of extracellular $Ca^{2+}$, the $[Ca^{2+}]_i$ increase was significantly inhibited by pretreatment of 1 µM dantrolene, a ryanodine receptor inhibitor. These results suggests that plasma membrane depolarization induced $Ca^{2+}$ influx and $Ca^{2+}$ release from the intracellular stores through ryanodine receptors in rat odontoblasts.

### [3P-060]

### Odontoblasts expressed glucocorticoid receptors

*Yuya Kuboyama[1], Maki Kimura[2], Takehito Ouchi[2], Ryuya Kurashima[2], Seikou Shintani[1], Yoshiyuki Shibukawa[2] (*[1]Department of Pediatric Dentistry, Tokyo Dental College, [2]Department of Physiology, Tokyo Dental College*)

**Objectives**
Recently, it has been reported that several patients subjected to the long-term steroid administration suffer severe dentin hypersensitivity-like tooth pain (steroid-derived tooth pain). The intensity of pain seems to be positively correlated with administrated dose of steroid, and is reversibly relieved by dose reduction or withdrawal. However, the occurrence mechanism of steroid-derived tooth pain has remained to be clarified. In addition, in not only patients undergoing long-term administration of steroids but also experimental animals treated by hydrocortisone/cortisone steroids have reported to increase the reactionary dentin formation, resulting in the narrowing of dental pulp cavity. Ritchie et al (2004) have reported that dexamethasone promotes extracellular matrix synthesis and mineralization in rat tooth organ culture, while Wang et al (2000) have reported that enlarged dental pulp cavity could be observed in rats undergoing methylprednisolone administration. However, the mechanism in which steroids mediated reactionary dentin formation has remained to be clarified. Dentin forming odontoblasts are sensory receptor cells. Nociceptive stimulation to the dentin surface activates TRP and Piezo channels in odontoblasts, increasing intracellular $Ca^{2+}$, which is then extruded by the $Na^{+}$-$Ca^{2+}$ exchanger to the dentin mineralizing front. In the present study, to elucidate the effects of steroids on odontoblast cellular function, we investigated expression patterns of glucocorticoid receptors and steroid-induced intracellular $Ca^{2+}$ signaling in mature human odontoblasts (HOB cells).
**Methods**
HOB cells were cultured in αMEM containing FBS. After loading calcium fluorescent indicator (fura 2-AM) for 60 min, we measured intracellular free $Ca^{2+}$ concentration ($[Ca^{2+}]_i$) in HOB cells during steroid application.
**Results**
In the presence of extracellular $Ca^{2+}$, application of 5 µM dexamethasone, a glucocorticoid receptor agonist, increased $[Ca^{2+}]_i$ in HOB cells.
**Conclusions**
This result suggests that glucocorticoid receptors was functionally expressed in odontoblasts.

### [3P-061]

### Effects of systematic yohimbine administration on masticatory muscle activities during sleep-wake states in freely moving rats

*Yiwen Zhu[1], Ayano Katagiri[1], Hiroki Toyoda[1], Masaharu Yamada[1,2], Makoto Higashiyama[1], Takafumi Kato[1,3,4] (*[1]Department of Oral Physiology, Graduate School of Dentistry, Osaka University, [2]Department of Dental Anesthesiology, Graduate School of Dentistry, Osaka University, [3]United Graduate School of Child Development, Osaka University, [4]Sleep Medicine Center, Osaka University Hospital*)

[Background] Masticatory muscle activities fluctuate in relation to sleep-wake states and arousals. Yohimbine (YOH), an alpha2-adrenoreceptor antagonist, has been used as a pharmacological stressor and shown to induce arousal activity and disruption of the sleep-wake cycles. This study aimed to investigate the effects of systemic yohimbine on sleep distribution and masticatory muscle activity during sleep.

[Method] In eighteen Sprague-Dawley rats, Electrodes for electroencephalogram and electromyogram of neck muscle were surgically implanted for sleep recording. Electrodes for left masseter muscle were surgically implanted to observe masticatory muscle activity. Sleep recordings were performed for continuous 2 days. On the first day, control data was collected from ZT 4 to ZT 18. Next day, baseline data from ZT 4 to ZT 6 were collected. Following injection at ZT6, the recording was continued until ZT 18. Rats were divided into three groups and intraperitoneally injected with YOH at three different doses (1.0, 2.0, and 3.0 mg/kg). Vigilance states (wakefulness, non-rapid eye movement [NREM] sleep, and rapid eye movement [REM] sleep) were scored for every 10-s epoch. To assess masseter activity level, electromyographic (EMG) activity was integrated for every 10-s epoch. After minimum value was subtracted, integrated EMG activity for each epoch was normalized by that during chewing.

[Results] After YOH injection, the percentage of wakefulness in the light phase (ZT7 - ZT12) increased dose-dependently while those of NREM and REM sleep decreased compared to control condition. The number of NREM sleep episodes also reduced dose-dependently in the light phase. Sleep variables did not change in the dark phase (ZT12 – ZT18). The median EMG activity level of masseter muscle was significantly decreased from wakefulness (5.5 %) to NREM (1.1 %) and REM (1.5 %) sleep in light phase of the control condition. However, the EMG activity level of masseter muscle did not significantly increase during all vigilance states after YOH injection in the light and dark phases.

[Conclusion] Acute systematic YOH can alter sleep-wake states by increasing wakefulness while masticatory muscle activity did not significantly increase during sleep and waking states.

COI: Properly Declared.

### [3P-062]

### The analysis for expression of ATP releasing channels which is related to water-evoked swallowing in laryngopharyngeal region of rats.

Chia-Chien Hsu[1], Chihiro Nakatomi[1], *Kentaro Ono[1] ([1]*Division of Physiology, Kyushu Dental University*)

Water-evoked swallowing is an essential function for water intake to maintain body fluid homeostasis. However, the receptive mechanism of water-evoked swallowing reflex in the pharyngeal-laryngeal region is still unclear. Recent findings report that the swallowing reflex-related nerves were activated by the releasing of ATP from laryngeal taste bud-like structures. We have found the swallowing reflex was inhibited by the administration of an ATP receptor inhibitor (AF353) to the rat laryngeal region, and taste bud-like structures were mainly located in the arytenoids. For these above findings, we focused on the ATP release pathway associated with water-evoked swallowing in arytenoids which contain taste bud-like structures. To investigate the localization of ATP releasing channels (Calhm1, Pannexin 1, Connexin 43) in laryngeal region, we collected arytenoids from six male Wistar rats (250~350 g) and performed RT-PCR to examine the mRNA expression levels of ATP releasing channels. In addition, the larynxes were obtained from five male Wistar rats (250-350 g) for preparing frozen sections. We excised rat larynxes transversely at a thickness of 10 μm, and then performed fluorescence immunostaining to investigate the localization of Calhm1, Pannexin 1, Connexin 43 in arytenoids. CK8 was used as a taste bud-like cell marker. In RT-PCR analysis, the expression of Calhm1, Pannexin 1, Connexin 43 were identified in rat arytenoids. Taste bud-like cells (CK-8 immuopositive) revealed immunopositive images for Calhm1, Pannexin 1, and Connexin 43. The arytenoid mucosal epithelium also revealed Calhm1 and Pannexin 1 immunopositive images, especially Connexin 43 showing strong immunopositivity. These results suggest that Calhm1, Pannexin 1, Connexin 43 expressed in the arytenoid mucosal epithelium including taste bud-like structures may be involved in the ATP release pathway which triggers water swallowing reflex.

### [3P-063]

### Effects of cervical vagal nerve stimulation on respiration and blood pressure variability and tissue blood flow in salivary gland

*Hirotaka Ueda[1], Yoshihiro Mitoh[2], Hiroshi Kamioka[1], Ryusuke Yoshida[2] ([1]*Department of Orthodontics, Okayama University Graduate School of Medicine, Dentistry and Pharmaceutical Sciences,* [2]*Department of Oral Physiology, Okayama University Graduate School of Medicine, Dentistry and Pharmaceutical Sciences*)

**[Aim]** The effects of cervical vagal nerve stimulation (VNS) on respiratory and blood pressure variability and salivary gland tissue blood flow are not well known. The aim of this study was to investigate the effects of afferent or efferent VNS on respiratory and blood pressure variability and salivary gland tissue blood flow. **[Materials and Methods]** Wistar/ST male rats were anesthetized with isoflurane, then the left cervical vagal nerves were cut, and bipolar stimulation electrodes were attached to the afferent and efferent bundle and connected to an electrical stimulator. A blood pressure transducer was inserted into the right-sided carotid artery to record blood pressure. A laser Doppler blood flow meter was attached to the left submandibular gland to record salivary gland tissue blood flow. A piezo transducer was attached to the chest to record respiratory variability. VNS were performed at 5 V, 40 Hz, for 10 seconds, and respiration, blood pressure, and tissue blood flow on the left submandibular gland were recorded. **[Results]** Afferent VNS induced transient respiratory depression. On the contrary, efferent VNS induced no respiratory depression. Blood pressure variations were not observed after both afferent and efferent VNS. Regarding tissue blood flow in salivary glands, afferent VNS enhanced tissue blood flow in salivary gland. On the other hand, efferent VNS had a limited effect on salivary tissue blood flow. **[Discussion]** The results suggest afferent VNS on the left side induced respiratory depression. The results also suggest that VNS on the left side has a limited effect on blood pressure variability. Regarding the effect of VNS on tissue blood flow in the salivary gland, it is suggested afferent VNS indicated a marked increase in tissue blood flow during afferent stimulation compared to the efferent VNS. It is possible that afferent VNS on the left side affected the respiratory center, however, further investigation will be needed.

### [3P-064]

### Differences in the effects of gustatory inputs on the hemodynamics of the three major salivary glands in rats and their relationship to salivation

*Toshiya Sato[1], Hisayoshi Ishii[1] ([1]*Div. of Physiol., Dept. of Oral Biol., Sch. of Dent., Health Sci. Univ. Hokkaido*)

Previously, we demonstrated that trigeminal sensory input reflexly induces increases in blood flow and salivation in the rat major salivary glands via parasympathetic nerves. This suggests the importance of parasympathetic nerve activation due to orofacial sensory input, not only in salivation but also in glandular hemodynamics. Furthermore, the relative salivary secretion ratio of the three major salivary glands is known to differ depending on the types of sensory input, such as gustatory and somatosensory stimulation, suggesting that glandular hemodynamics are also regulated according to differences in sensory input. However, no details are available. Thus, we analyzed the glandular hemodynamics and salivation in major salivary glands during electrical stimulation of the taste nerve, which induces particularly pronounced salivation in rats.Rats were anesthetized with urethane and artificially ventilated. The femoral artery and vein were cannulated to allow the injection of the drugs and the monitoring of blood pressure, respectively. A central cut end of the lingual nerve was electrically stimulated. While the somatosensory afferent fibers of the lingual nerve were cut, the gustatory afferent fibers via the chorda tympani nerve remained intact. The cervical sympathetic trunk was cut in the neck. The glandular hemodynamics were recorded using a laser speckle flow meter. Salivary gland ducts were cannulated to collect secreted saliva.Electrical stimulation of the taste nerve induced increases in blood flow and salivation in the submandibular and sublingual glands, but not in the parotid glands. These increases in blood flow and salivation were completely inhibited by intravenous administration of an autonomic ganglion blocker (hexamethonium) and significantly reduced by administration of NO synthesis inhibitor (L-NAME), which has only an inhibitory effect on vasodilation.Our results indicate that gustatory input from the tongue was involved in parasympathetic increases in blood flow and salivation in the submandibular and sublingual glands, but not in the parotid glands. Furthermore, inhibition of parasympathetic increases in blood flow significantly decreases salivation. These results suggest that differences in the effects of gustatory inputs on the hemodynamics of the three major salivary glands may account for the differences in the relative secretion ratio of saliva, and glandular hemodynamics are closely related to salivation.

### [3P-065]

### Tooth loss upregulated the expression of AD-related molecules in hippocampus and suppressed cognitive ability in Alzheimer's model mice.

*Maeshiba Munehisa[1,2], Hiroshi Kajiya[2,3], Keisuke Migita[4], Kazuko Goto[5], Kei Egashira[2], Tsugumi Fujita[3], Takashi Tsuzuki[1] ([1]*Department of Rehabilitation, Fukuoka Dental College,* [2]*Oral Medical Center, Fukuoka Dental College,* [3]*Department of Physiological Science and Molecular Biology, Fukuoka Dental College,* [4]*Department of Drug Informatics, Faculty of Pharmaceutical Sciences, Fukuoka University,* [5]*Department of Dental Hygienist, Fukuoka College of Health Science*)

[Backgroud] There have been some reports on the relationship between lesions in periodontal tissue and cognitive ability. We have also reported that hyperocclusion in occlusal disharmony induced decline in cognitive ability. Tooth loss have also been reported to be associated with oral dysfunction such as social skills and oral health QOL, as well as decline in cognitive ability. Although occlusal supporting after tooth loss using dentures may be reduced the risk of dementia, it is not yet clear about the mechanism as evidence-based dentistry. The present experiments are aimed to clarify the correlation between tooth loss, its recovery of occlusal supporting and maintenance of cognitive ability using Alzheimer's disease model mice.

[Methods] Alzheimer's disease (AD) model mice (familial Alzheimer's disease variant arctic triple mutant knock-in mouse (C57BL/6-App(tm3 (NL-G-F); AppKI(3)) and its control (C57BL/6-App(tm1 (NL); AppKI(1) ) mice were used in present experiments. The model mice were divided into three groups: a control group, a group in 1 month after extraction of mandibular molars, and a group in occlusal support on 1 month after tooth extraction. The expression in AD-related molecules was evaluated using mouse brain slices. Two types of behavior test were evaluated by video recording and visual inspection in present experiments. The social memory test employed an eight-arm radial maze test (EARMT). The object recognition test (ORT) was used as various aspects of learning and memory.

[Results&Discussion] The tooth loss declined the cognitive abilities at 2 and 4 months after extraction in control (APPKI(1)) mice and Alzheimer model mice (APPKI(3)) using eight-arm radial test (ERMT), but not at 6months in APPKI(3) mice. The tooth loss upregulated the positive cells of AD-related molecules (amyloid-beta, p-tau in trigeminal mesencephalic nucleus (Vmes) in APPKI(3) mice , but not in locus coeruleus (LC). The extraction also upregulated the remarked expression of AD-related proteins in hippocampus at 2 and 4 months after extraction compared with control in APPKI(3) mice. Furthermore, the tooth loss dominantly upregulated the positive cells of p-Tau in CA3 of hippocampus at 4 month after extraction compared with control (non-extraction) in APPKI (3) mice. These results suggest that the expression of phosphorylated tau in the hippocampal region after tooth extraction may be associated with a risk factor of cognitive decline in occlusal disharmony.

### Poster

[3P]
**Circulation**

March 30, 13:00 - 14:20, Poster Room

### [3P-066]

### Nuclear connectin novex-3 contributes to fetal cardiomyocyte proliferation by softening nuclei.

*Ken Hashimoto[1], Momoko Ohira[1], Aya Kodama[1], Misaki Kimoto[1], Mariko Inoue[2], Yuu Usui[1], Akira Hanashima[1], Yoshihiro Ujihara[3], Satoshi Mohri[1] ([1]*First Department of Physiology, Kawasaki Medical School,* [2]*Central Research Institute, Kawasaki Medical School,* [3]*Department of Electrical and Mechanical Engineering, Nagoya Institute of Technology*)

Coordinated regulation of proliferation and differentiation of cardiomyocytes at the appropriate timing are required for normal cardiac development. Novex-3, the short splice variant of the giant sarcomeric protein connectin (titin), is an ill-defined protein, which was first identified as a component of an elastic Z-disk to I-band linking system, and was recently suggested to be expressed in cardiomyocyte nuclei and to promote proliferation of these cells during fetal life. Here we analyzed novex-3 knock-out mice to better understand the pathophysiological role of this protein. These mice showed an impaired cardiomyocyte proliferation during early development before birth, and demonstrated a trend toward cardiac hypertrophy at the neonatal stage. In adults, hypertrophy deteriorated and progressed to left ventricular dilation and contractile dysfunction with impaired $Ca^{2+}$ handling, resulting in poor survival. Mechanistic analysis revealed that stiffer nuclei with stabilized circumnuclear microtubules in knock-out cardiomyocytes could be associated with impaired proliferation at the perinatal stage, whilst the distributions of nuclear lamins and cytoskeletal proteins such as alpha-actinin and desmin intermediate filaments remained unchanged. These data suggest that novex-3 has a non-sarcomeric function, and plays a pivotal role as an early contributor for cardiomyocyte proliferation which constitutes a part of the coordinated regulation program required for normal cardiac development in mice. Improving our understanding of novex-3 functions could pave the way for possible applications in cardiac regeneration in post-mitotic adult cardiomyocytes. Possibly, re-expression of nuclear novex-3 could restore compliant nuclei, thereby driving gene expression program toward cardiomyocyte proliferation.

### [3P-067]

### Deciphering the Potential Mechanisms of *Alternanthera sessilis* Red against Atherosclerosis using Network Pharmacology and Molecular Docking Approaches

*OMILLA RAGAVAN[1], YOKE KEONG YONG[1], MUHAMMAD NAZRUL HAKIM ABDULLAH[2], LAI YEN FONG[3] ([1]*DEPARTMENT OF HUMAN ANATOMY, FACULTY OF MEDICINE AND HEALTH SCIENCES, UNIVERSITY PUTRA MALAYSIA.,* [2]*DEPARTMENT OF BIOMEDICAL SCIENCE, FACULTY OF MEDICINE AND HEALTH SCIENCES, UNIVERSITI PUTRA MALAYSIA.,* [3]*DEPARTMENT OF PRE-CLINICAL SCIENCES, FACULTY OF MEDICINE AND HEALTH SCIENCES, UNIVERSITI TUNKU ABDUL RAHMAN*)

**Objective:** The red cultivar of *Alternanthera sessilis* (ASR), a medicinal weed traditionally consumed by Asians, reduces the risk of cardiovascular disease with established athero-protective effects from previous studies. However, the underlying mechanism of ASR against atherosclerosis remains unknown. This investigation aims to discover the potential mechanisms of ASR in treating atherosclerosis using network pharmacology and molecular docking. **Methodology:** Phytochemicals of ASR were collected via literature search. Screening of their pharmacokinetic properties was conducted in accordance with the criteria of the Traditional Chinese Medicine System Pharmacology (TCMSP) database, which required a minimum of 30% oral bioavailability and a drug-likeness value greater than 0.18. Potential targets of ASR bioactive compounds were sourced from SwissTargetPrediction and DrugBank databases; atherosclerosis targets from OMIM, DisGeNet, and GeneCards databases. The Venn diagram demonstrated overlapping genes. STRING database and Cytoscape software were used to construct protein-protein interaction (PPI) and compound-target networks respectively. Hub genes were retrieved based on three topological parameters: Degree, Betweenness, and Closeness. DAVID bioinformatics tool was employed for Gene Ontology (GO) and Kyoto Encyclopedia of Genes and Genomes (KEGG) pathway enrichment analyses. Molecular docking validated binding affinities between key bioactive compounds and the hub genes. **Results:** Among 179 compounds identified from ASR, only 4 (Dehydrodieugenol, Luteolin, Quercetin and Xanthosine) emerged as key bioactive compounds meeting the criteria. PPI network between ASR and atherosclerosis targets revealed 192 intersecting genes, from which 4 hub genes were discovered namely AKT1, HSP90AA1, SRC and ESR1. These targets were associated with positive regulation of MAPK cascade according to GO analysis. KEGG pathway analysis showed that lipid and atherosclerosis were among the top 10 significantly enriched pathways in the list preceded by the PI3K-Akt signaling pathway. This finding suggests the potential impact of ASR key compounds on enhancing the protective role of Akt-dependent eNOS activation, leading to physiological nitric oxide generation and a subsequent reduction in the prolonged inflammation. Besides, the molecular docking results displayed Dehydrodieugenol-ESR1 complex with the highest binding affinity at -8.56 kcal/mol. **Conclusion:** Our study uncovers potential molecular pathways for treating atherosclerosis with ASR through network pharmacology and molecular docking. Experimental validation *in-vitro* and *in-vivo* is necessary to confirm these computational findings.

### [3P-068]

### Vascular endothelial dysfunction in emerin deficient mice

*Megumi Kato[1], Takafumi Iesaki[2], Yukiko Hayashi[1] ([1]*Department of Pathophysiology, Tokyo Medical University,* [2]*Department of Radiological Technology, Faculty of Health Science, Juntendo University*)

Purpose: Nuclear envelopathy is known to cause various diseases such as muscular dystrophy, cardiomyopathy, progeria, and lipodystrophy by genetic mutations of nuclear membrane proteins such as lamin A/C and emerin. Members of the Linker of Nucleoskeleton and Cytoskeleton (LINC) complex, nesprins, which were deeply associated with nuclear envelope proteins, were reported to have important roles of mechanotransdution and morphology in the endothelial cells. In this study, we investigated the vascular endothelial function using emerin-deficient mice (EMD). Methods: We used male EMD and C57BL/6J mice as control (WT) at 12 weeks of age. We examined glucose tolerance and lipid levels in serum. Endothelial function was analyzed by wire myography using thoracic aorta. We performed qRT-PCR to check gene expressions and histological analyses of the aortic endothelial cells by TEM (Transmission electron microscopy). Results: EMD mice had glucose intolerance and hyperlipidemia. Endothelial dysfunction was also observed from 12 weeks of age. There were morphological abnormalities of aortic endothelial cells in EMD by TEM. Conclusion: These results suggested the lack of emerin is related to endothelial dysfunction.

### [3P-069]

### Effect of Inhaled Nitric Oxide (iNO) Therapy on Myocardial Reverse Remodeling in a Rat Model of Cardiac Hypertrophy: Exploring New Potential for iNO

*Hirotsugu Tsuchimochi[1], James Pearson[1] ([1]*National Cerebral and Cardiovascular Center*)

***Purpose:*** Currently, insurance coverage for inhaled nitric oxide (iNO) therapy using a dedicated inhalation device is limited to improving hypoxic respiratory failure with pulmonary hypertension in neonates and pulmonary hypertension in the perioperative period of cardiac surgery. Therefore, new applications of iNO for cardiovascular disease need to be explored.
***Method:*** First, to examine the effects of iNO on systemic hemodynamics in the unanesthetized state, male Sprague-Dawley rats with an indwelling blood pressure transmitter were exposed to 20-80 ppm of NO and the effects on arterial pressure (AP) and heart rate (HR) were examined. Next, NO electrodes were implanted in the ascending aorta of rats under propofol anesthesia, and arterial blood NO concentration, plasma nitrite and nitrate, AP and HR were measured in response to NO gas inhalation. Furthermore, using a recovery model from ascending aortic constriction (AAC) induced cardiac hypertrophy, the effects of chronic exposure to 20 ppm NO for 2 weeks during the recovery period on both left and right ventricular function were examined.
***Results:*** Inhalation of NO gas at low concentrations used in a clinical setting did not affect AP or HR in unanesthetized rats. Arterial blood NO concentrations did not change significantly in the range of 20-80 ppm of iNO, nor were they affected by L-NAME (50 mg/kg iv) in anesthetized rats. On the other hand, plasma nitrite and nitrate concentrations increased immediately after iNO. Under isoflurane anesthesia, 20 ppm of iNO had no significant effect on cardiac function, whereas at 80 ppm a decrease in LVSP, AP, PAP, and LV dP/dt was observed. Lastly, in the recovery from AAC-induced cardiac hypertrophy, 2 weeks of chronic exposure to 20 ppm NO had no significant effect on cardiac function. Low concentration of iNO therapy used in a clinical setting was suggested to increase NO storage by increasing blood nitrite and nitrate concentrations without significantly affecting circulating blood NO gas concentrations or systemic hemodynamics.
***Conclusion:*** The potential for acute iNO to reduce cardiac afterload has been demonstrated, but the impact of iNO in more severe cardiovascular disease models, including NO storage capacity as nitrite or nitrate and the side effects of iNO, should be investigated hereafter.

### [3P-070]

### Atrioventricular hearts and molecular spring connectin in the mollusc oyster

*Akira Hanashima[1], Misaki Kimoto[1], Momoko Ohira[1], Yuu Usui[1], Ken Hashimoto[1], Satoshi Mohri[1] ([1]*First Department of Physiology, Kawasaki Medical school*)

Cardiomyocytes contract through the molecular movement of myosin and actin within their sarcomeres, but require other systems for extension. While vertebrates have developed the ability to fill their ventricles with blood from their atria during evolution, the link between atrial size and ventricular function is not well known. We examined the heart of the oyster, a mollusk that has an atrial/ventricular heart similar to vertebrates, at different levels of organization, from organ to molecule, and compared their evolutionary history since 600 million years ago. The oyster heart was situated in a cavity of equal volume that was enclosed by a scallop adductor muscle or another structure. The oyster heart resembled the mammalian heart in having two atria and two ventricles. The atria and ventricles contracted in a sequential manner, despite the absence of atrioventricular conduction. During ventricular contraction, the cavity became negatively pressurized, allowing blood to flow into the atria. However, X-ray CT and pressure measurement revealed that oysters have open circulatory systems with low atrial and ventricular pressures, despite having vascular systems, unlike vertebrates with closed circulatory systems. The ventricular tissue of oysters had a spongy texture, composed of elongated cardiomyocytes that resembled those of amphibian ventricles with large atria. The electron microscopy revealed a wide spacing of the myosin filaments that mediated the contraction of the cardiomyocytes. Gel electrophoresis and RT-PCR experiments revealed very long elastic regions of the connectin molecules that determined the extensibility of the cardiomyocytes. These features suggested a high capacity for dilatation similar to that of amphibian ventricles with large atria. Therefore, we propose that the long elastic region of connectin and the high ventricular dilatability in hearts that rely mainly on atrial filling are a universal principle common to all animal phyla.

### [3P-071]

### Elevation of modified nucleosides in blood by chronic kidney disease affect vascular endothelial cell proliferations

*Hitomi Kaneko[1], Yu Nagayoshi[1,2], Ryosuke Yamamura[1,2], Takeshi Chujo[1], Kazuhito Tomizawa[1] ([1]*Department of Molecular Physiology, Faculty of Life Sciences, Kumamoto University,* [2]*Department of Nephrology, Faculty of Life Sciences, Kumamoto University*)

Various post-transcriptional chemical modifications of RNA contribute to protein translation efficiency and stability of RNA structure. We have previously found that modified RNA is degraded to nucleosides and that the nucleosides at the site of modification (modified nucleosides) are excreted extracellular space, especially in urine. In addition, we have reported that these modified nucleosides have physiological functions via some receptors like adenosine reseptors. In epidemiologic studies, renal failure is known as a major risk factor of cardiovascular disease. In this study, we hypothesize that the excretion of modified nucleosides from urine is decreased by renal failure. Thus the abnormal accumulation of modified nucleosides in the blood may damage cardiac myocytes or vascular endothelial cells. First, we collected serum from 230 patients with chronic kidney disease and compared the correlation between the concentration of modified nucleosides in the blood and eGFR, an index of renal function. We found that several modified nucleosides were increased in the serum and significantly inversely correlated with eGFR. Especially, 1-methylinosine ($m^1I$) was most significantly inversely correlated with eGFR. Next, we performed the addition of $m^1I$ to HAoECs, a vascular endothelial cell line. As a result, HAoECs significantly increased cell proliferation by administration of $m^1I$, and the same reaction was not observed in other cell lines. We explored the extracellular receptors of $m^1I$. We found that the expression level of adenosine $A_{2A}$ receptor is significantly elevated in HAoECs and $m^1I$ activates Erk phosphorylation through adenosine $A_{2A}$ receptor. Additionally, RNA-seq analysis indicated that $m^1I$ causes activation of MAPK signaling and increases in inflammation-associated markers. Therefore, it was suggested that signaling by $m^1I$ induces inflammation-related pathological proliferation, and leading to angiostenosis and finally related to the pathogenesis of vascular disease.

### [3P-072]

### The contribution of large-diameter afferent fibers to cerebral blood flow response to sciatic nerve stimulation in urethane-anesthetized mice

*Nobuhiro Watanabe[1], Masamichi Moriya[1], Harumi Hotta[1] ([1]*Department of Autonomic Neuroscience, Tokyo Metropolitan Institute for Geriatrics and Gerontology*)

Cardiovascular function is regulated during exercise and its mechanisms involve both central command and peripheral afferent inputs. Cerebral blood flow (CBF) is also modulated during exercise. As to its mechanism, the contribution of central commands has been reported, whereas the involvement of peripheral afferent inputs in CBF regulation during exercise is yet to be fully understood. To imitate exercise, electrical stimulation is used for skeletal muscle contraction. We aimed to investigate the influence of sciatic nerve stimulation on CBF in anesthetized mice. Animals were anesthetized using urethane and artificially ventilated. Rectal temperature was maintained. CBF was measured using a laser-speckle contrast imager. A bipolar wire electrode was placed on the left sciatic nerve kept intact. A tetanic contraction of left hindlimb was induced by burst electrical stimulation, every second, 10 times in total (rectangular electrical pulses with a duration of 0.2 ms at twice the motor threshold, which mainly activate group I nerve fibers). CBF increased during the stimulation and peaked immediately after the stimulation ended. The CBF increase in the parietal cortex was by approximately 20 % and the increase was significantly larger than those in frontal and occipital cortex. The CBF increase did not differ between the right and left hemispheres. In contrast, blood flow in the temporal muscle was not significantly affected by the stimulation. Transection of the sciatic nerve at the distal side of the electrode abolished muscle contraction, however, the CBF response to the stimulation was remained. The present study suggests that CBF increases induced by sciatic nerve stimulation are attributed to afferent inputs possibly conveyed by the group I fibers of the hindlimb.

### [3P-073]

### Mechanisms of automaticity in HL-1 mouse atrial myocytes: roles of the membrane and $Ca^{2+}$ clocks

*Yuhichi Kuda[1], Yasutaka Kurata[1], Takayuki Ikeda[2], Mamoru Tanida[1], Kunichika Tsumoto[1], Toshishige Shibamoto[1], Hideto Yonekura[2] ([1]*Department of Physiology II, Kanazawa Medical University, Japan,* [2]*Department of BiochemistryII, Kanazawa Medical University, Japan*)

**Methods**: Kir2.1 was transfected into HL-1 cells to reduce the occurrence of automaticity. Fluorescence imaging using Cal-520 and FluoVolt was performed to measure intracellular $Ca^{2+}$ dynamics and membrane potentials, respectively, under the following conditions: 1) administration of 1 mM $Ba^{2+}$ ($I_{K1}$ blocker) alone, and 2) administration of 10 µM BAPTA-AM with and without 1-Hz electrical pacing. $I_f$ dynamics in normal (untransfected) HL-1 cells was determined by the superforated-patch recording using nystatin (Shioya, 9th FAOPS, 2019).**Results**: 1) $Ba^{2+}$ administration to Kir2.1-overexpressing quiescent HL-1 cells induced automaticity, following oscillatory membrane potential depolarization that shows resting state instability. 2) $I_f$ was detected in some normal HL-1 cells but the cells with $I_f$ did not have automaticity, whereas the normal HL-1 cells that had no $I_f$ showed automaticity. 3) 10 µM BAPTA-AM did not affect automaticity of Kir2.1-overexpressing HL-1 cells in the absence of $Ba^{2+}$, while abolishing $Ca^{2+}$ transients.**Conclusions**: HL-1 cells showed $I_{K1}$ block (depolarization)-induced automaticity. $I_f$ was not required for the generation of spontaneous activity. Automaticity occurred even when $Ca^{2+}$ transients were eliminated. These findings suggest that HL-1 cells exhibit automaticity by the membrane clock independent of the $I_f$ channel. SR $Ca^{2+}$ clock may not be essential for HL-1 cell automaticity.

### [3P-074]

### Theoretical and experimental analysis of the relationship between ventricular size and stiffness properties of trabecular ventricles of Anura

*Megumi Ito[1], Shukei Sugita[1], Masanori Nakamura[1], Yoshihiro Ujihara[1] ([1]*Nagoya Inst. Tech.*)

The diastolic mechanical property of the ventricle is assessed by examining the ventricular diastolic pressure-volume (P-V) relationship, which is generally quantified by the stiffness or the stiffness constant. To exclude the effect of size on ventricular stiffness, the P-V relationship of humans and other mammals is usually normalized with the ventricular lumen volume. However, in amphibians and reptiles with trabecular ventricles, the ventricular mass is used for normalization instead of ventricular lumen volume[1, 2], since it is difficult to define the ventricular lumen accurately. Nevertheless, whether normalizing the P-V relationship by ventricular mass is a favorable method for attenuating the ventricular size effect when evaluating the mechanical properties of trabecular ventricles of different sizes is unclear. In this study, we analyzed theoretically and experimentally the relationship between the size of the trabecular ventricle and the passive mechanical property[3]. Assuming that the trabecular ventricle is a thin-walled sphere, the theoretical analysis showed that the stiffness parameters obtained directly from the P-V relationship were inversely proportional to the natural ventricular lumen volume. We then estimated the natural ventricular lumen volume from the lumen area of ventricular cross sections of three species of anurans (frogs and toads) with different ventricular size and habitats, *X. laevis* (aquatic), *P. nigromaculatus* (semiaquatic), and *B. j. formosus* (terrestrial). The natural ventricular lumen volume was proportional to the ventricular mass, suggesting that the stiffness parameters were inversely proportional to ventricular mass. We performed pressure-loading tests on the trabecular ventricles of three species of anurans and obtained P-V relationships, and the stiffness parameters calculated from P-V relationships were inversely proportional to ventricular mass. These combined results suggest that the stiffness parameters of the trabecular ventricles of Anura are essentially dependent on the ventricular size. Normalization of the volume change during a pressure-loading test with the ventricular mass helped to attenuate the size effect of the ventricles, and this suggests that it would be a valuable method for comparing the diastolic mechanical functions of different-sized trabecular ventricles.

1) Honda et al., Kawasaki Med J, 2018.
2) Ito et al., J Biol Phys, 2021.
3) Ito et al., J Biorheol, In press.

### [3P-075]

### Devised method of wearing a portable brain activity measuring device for measuring cerebral blood flow in bathing

*Takeshi Yamaguchi[1], Hideki Yano[2], Fumiyo Miki[3], Yasuko Okuda[4] ([1]*Laboratory of anatomy, physiology and cardiovascular sciences, Faculty of Nursing, Shikoku university,* [2]*Adult health nursing, Faculty of Nursing, Shikoku university,* [3]*Women's health nursing, Faculty of Nursing, Shikoku university,* [4]*Graduate school of nursing, Hiroshima bunka gakuen university*)

[Background] Suzuki et al. have reported that bathing accidents are associated with transient disturbance of consciousness and fatigue. [Aim] The aim of this study was to clarify how cerebral blood flow is related to circulatory function and whether it can be used as an index of criteria for safe bathing by measuring and quantifying cerebral blood flow. [Methods] The subjects were nine healthy adult males (21 ± 1 years old) with no physical problems. At the same time as measuring vital signs such as blood pressure and pulse rate from before bathing to 10 minutes after bathing, hemoglobin (Hb) levels were measured using HOT -2000 (NeU Inc.) by near-infrared spectroscopy. The integral value of Hb levels before, during and after bathing was used as cerebral blood flow. [Results and discussion] At the beginning of the experiment, the left and right cerebral blood flow was significantly misaligned due to sweating and body movements, but was further fixed with a rubber band as an improvement measure. As a result, the deviation between the left and right cerebral blood flow was almost eliminated and the left and right cerebral blood flow changed synchronously. Cerebral blood flow increased during the bath and for 10 minutes after the bath compared to before the bath.

### [3P-076]

### Frequency and details of voluntary exercise in Dilated Cardiomyopathy model mice

*Masami Sugihara[1], Ryo Kakigi[2], Takashi Murayama[3], Sachio Morimoto[4], Nagami Kurebayashi[3] ([1]*Dept. Clin. Lab. Med., Juntendo Univ. Fac. Med.,* [2]*Fac. of Health & Sports Sci., Juntendo Univ.,* [3]*Dept. Pharmacol., Juntendo Univ. Fac. Med.,* [4]*Dept. Health Sci. at Fukuoka, IUHW*)

Dilated cardiomyopathy (DCM) is one of major causes of heart failure (HF) and characterized by dilatation of the left ventricle and markedly reduced contractility. The effects of exercise on patients with inherited DCM have not been established because DCM is associated with high risk of worsening HF and sudden death(SD) due to lethal arrythmia. A knock-in mouse model of human inherited DCM, TNNT2 ΔK210, shows similar dispositions to DCM patients and is useful model for evaluating therapeutic effects. In this study, we observed locomotion in more detail and examined the effects of different frequencies of wheel running on cardiac function and lower limb muscles.Homozygous ΔK210 (DCM) mice showed enlarged heart and frequent SD with t1/2 of ~70 days. DCM mice were divided into 3 groups based on the frequency of voluntary exercise: no exercise control (CONT), a group was kept on wheels for 2 days and kept without wheels for 2 days, repeated for 4 days (2D) and daily exercise (ED). The 2D and ED groups started running at 1 month of age. At the 2 months of age, mice were sacrificed after an investigation with echocardiography and ECG, and their heart, lung, lower extremity muscles (soleus, plantaris and gastrocnemius) and body weights were measured. Gene expressions associated with HF in the myocardium were measured by qPCR analysis. We also examined lower extremity muscle composition. About 60% of the running in both the 2D and ED groups was at night. In echocardiography, while the ejection fraction was significantly improved in ED group, E/e' increased predominantly in 2D group compared with ED group and CONT groups, suggesting worsening diastolic function. The weights of soleus muscles were significantly and similarly increased in 2D and ED groups. We will further investigate the relationship between cardiac function and changes in lower extremity muscle strength.

### [3P-077]

### Donepezil Markedly Prevents Cardiac Remodeling in Obesity-induced Hypertensive Rats

*Meihua Li[1], Toru Kawada[1], Masafumi Fukumitsu[1], Takuya Nishikawa[2], Keita Saku[1]
(*[1]Department of Cardiovascular Dynamics, National Cerebral and Cardiovascular Center, [2]Department of Research Promotion and Management, National Cerebral and Cardiovascular Center*)

Acetylcholinesterase inhibition by donepezil has shown anti-inflammatory effects, such as suppression of cardiac production and release of the cytokine in reperfused myocardial infarction. This study investigated whether donepezil applies to the treatment of obesity-induced hypertension. Four-week-old rats were fed a high-fat diet (57% kcal as fat) throughout the study period. After 4 weeks, we implanted a blood pressure transmitter for monitoring conscious hemodynamics. After one week of recovery, all animals were randomly assigned to an untreated (UT, n = 15) or donepezil-treated (DT, n = 11, 3 mg/kg/day) group. After the treatment of 10 weeks, the effects of donepezil were evaluated by hemodynamics, blood biomarkers, immunohistochemistry, and morphology. Compared with UT, DT significantly suppressed hypertension. DT prevented the progression of cardiac remodeling and dysfunction. DT prevented obesity-induced hypertension-related soleus muscle atrophy and maintained the activity of rats. DT also decreased plasma levels of insulin, leptin, and CRP. The results suggest that donepezil may be used as a potential therapeutic candidate for patients with obesity-induced hypertension.

### [3P-078]

### Effects of arterial baroreflex on aortic impedance and renal arterial impedance

*Toru Kawada[1], Yukiko Fukuda[2], Meihua Li[1], Masafumi Fukumitsu[1], Takuya Nishikawa[3], Keita Saku[1] (*[1]Department of Cardiovascular Dynamics, National Cerebral and Cardiovascular Center, [2]Medical and Health Informatics Laboratories, NTT Research, Inc, [3]Department of Research Promotion and Management, National Cerebral and Cardiovascular Center*)

Knowledge about arterial impedance is important to develop precise cardiovascular simulators. We examined the effects of the arterial baroreflex on aortic impedance ($Z_{AO}$) and renal arterial impedance ($Z_{RA}$) in anesthetized rats (n = 6). Carotid sinus baroreceptor regions were isolated from the systemic circulation, and carotid sinus pressure (CSP) was controlled to either 60 mmHg or 140 mmHg. Under each CSP condition, the heart was randomly paced for 60 s. We calculated $Z_{AO}$ from aortic flow and aortic pressure data, and $Z_{RA}$ from renal blood flow and renal arterial pressure data. A three-element Windkessel model was used to quantify $Z_{AO}$ (R1: characteristic impedance, R2: peripheral resistance, and C: aortic compliance) and $Z_{RA}$ (R1: proximal impedance, R2: distal impedance, and C: renal arterial compliance). As expected, R1 and R2 were significantly larger, and C was significantly smaller in $Z_{RA}$ than in $Z_{AO}$. The increase in CSP from 60 mmHg to 140 mmHg did not affect R1 in $Z_{AO}$ (0.052 ± 0.007 vs. 0.070 ± 0.013 mmHg·min/mL) or in $Z_{RA}$ (0.920 ± 0.139 vs. 1.314 ± 0.255 mmHg·min/mL), but it significantly reduced R2 in $Z_{AO}$ (0.952 ± 0.099 vs. 0.644 ± 0.102 mmHg·min/mL, $P < 0.05$) and in $Z_{RA}$ (9.363 ± 1.584 vs. 5.931 ± 1.102 mmHg·min/mL, $P < 0.05$). The increase in CSP did not affect C in $Z_{AO}$ [5.46 ± 0.57 vs. 5.86 ± 0.80 ($\times 10^{-3}$) mL/mmHg] but significantly increased C in $Z_{RA}$ [0.035 ± 0.005 vs. 0.190 ± 0.070 ($\times 10^{-3}$) mL/mmHg, $P < 0.05$]. These results suggest differential effects of the carotid sinus baroreflex on $Z_{AO}$ and $Z_{RA}$. The renal circulation has local autoregulation that increases renal vascular resistance in response to an increase in renal arterial pressure. In the present experimental settings, an increase in CSP reduced efferent sympathetic nerve activity and arterial pressure. The presence of the renal autoregulation might have contributed to the differential effects of the carotid sinus baroreflex on $Z_{AO}$ and $Z_{RA}$.

### [3P-079]

### Mavacamten effects on sarcomere contractile dysfunction in transgenic mouse myosin binding protein-C mutant models of hypertrophic cardiomyopathy

*James T Pearson[1], Mark Waddingham[1], Hirotsugu Tsuchimochi[1] (*[1]NCVC*)

Cardiac myosin binding protein-C (cMyBP-C) is an important negative regulator of muscle contractility inhibiting myosin filament sliding within the sarcomere. Mutations in Mybpc3 are one of the most common causes of familial hypertrophic cardiomyopathy characterised by hypercontractility and impaired muscle relaxation. Utilising synchrotron X-ray scattering simultaneous with pressure-volumetry we examined in situ cross-bridge cycling and sarcomere shortening in the left ventricle (LV) to understand the progression of contractile and diastolic dysfunction in rodent models deficient in cMyBP-C (truncation and global deletion models) and how myofibrillar ATPase inhibition (mavacamten 2.5mg/kg ip bolus) affects actin-myosin dynamics. Beat-to-beat myosin mass transfer to actin filaments and sarcomere shortening were heterogeneous across the LV free wall, but greatest in the epicardium (hypercontractility relative to WT) in Mybpc3 mutants (del/del and del/+). On the other hand, weakly-binding myosin S1 cross-bridges remaining in close proximity to actin in diastole in the subendocardium was pronounced in del/del mice and some del/+ mice (with diastolic dysfunction), which was associated with reduced shortening and delayed sarcomere lateral expansion. Both impairments were partially corrected by acute mavacamten treatment (15min) with a small reduction in sarcomere shortening. Conversely, in some cases, severely hypercontracted subendocardial muscle lost contractile function following 2-3h mavacamten exposure while evoking exaggerated muscle shortening in the epicardium of the same hearts. In such dysfunctional hearts subsequent dobutamine stimulation re-awakened subendocardial contractile function. These findings suggest acute ATPase inhibition does not improve subendocardial function in advanced hypertrophic cardiomyopathy states. Currently, the effects of chronic mavacamten treatment on sarcomere and LV function and remodeling are being investigated.

### [3P-080]

### Effect of high hydrostatic pressure on cellular contractility in mouse cardiomyocytes

*Yohei Yamaguchi[1], Toshiyuki Kaneko[2], Kachi Cho[1], Keisei Murata[1], Gentaro Iribe[2], Masayoshi Nishiyama[3], Susumu Ohya[1] (*[1]Department of Pharmacology, Graduate School of Medical Sciences, Nagoya City Univ., [2]Department of Physiology, Asahikawa Medical Univ., [3]Department of Physics, Kindai Univ.*)

The heart contracts continuously throughout its life, and myocardial cells are subjected to varying hemodynamic stresses. An increase in preload stretches the ventricular walls, initiating the Frank-Starling mechanism, which enhances myocardial cellular contractility. Meanwhile, an increase in afterload raises ventricular pressure, leading to higher hydrostatic pressure on myocardial cells. However, the precise effect of high hydrostatic pressure on cardiac contraction remains elusive due to the lack of a suitable measurement system under such hydrostatic pressure conditions. In this study, we investigated the effect of high hydrostatic pressure on cardiomyocyte contraction and $Ca^{2+}$ handling using our developed hydrostatic pressure microscope. Isolated ventricular myocytes were electrically stimulated at 1 Hz and exposed to high hydrostatic pressure in a normal Tyrode's solution-filled hydrostatic pressure chamber. Our observations revealed a significant increase in myocardial cellular contractility, as measured by changes in sarcomere length, and $Ca^{2+}$ transient, as measured using the $Ca^{2+}$ indicator (Fura-4F), during exposure to high hydrostatic pressure at 200 mmHg, compared to ambient conditions. Furthermore, the genetic deletion of TRPC6, mechanosensitive channels, suppressed these increases. Our findings suggest that high hydrostatic pressure induces an increase in myocardial cellular contractility by enhancing $Ca^{2+}$ transient via TRPC6 channels.

### [3P-081]

### Impact of increased left atrial pressure on pulmonary arterial impedance estimation in rats

*Masafumi Fukumitsu[1], Naoshi Hotta[2], Toru Kawada[1], Meihua Li[1], Takuya Nishikawa[1], Keita Saku[1] ([1]*National Cerebral and Cardiovascular Center,* [2]*Yokohama city university hospital*)

Pulmonary arterial impedance (PAZ) provides the pulsatile properties of pulmonary vasculature. In pulmonary circulation, the ratio of downstream pressure [left atrial pressure (LAP)] to upstream pressure [pulmonary arterial pressure (PAP)] is relatively large compared to systemic circulation. We have reported that LAP little affected PAZ estimation in normal physiological condition, however, it remains unclear to what extent LAP affects the PAZ estimation when LAP excessively increases in the pathological status. To address this issue, we used six Sprague-Dawley rats to examine the PAZ as a reciprocal change of hydraulic admittance using the two methods: 1) a one-input, one-output system (I1O1 analysis) that does not take into LAP account, and 2) a two-input, one-output system (I2O1 analysis) that included LAP. PA flow, PAP and LAP waves were measured simultaneously under irregular pacing. Transverse aortic ligation followed by blood transfusion (TAL/BT) was performed to increase LAP >15mmHg. TAL/BT increased PAP from 17 to 33 mmHg, and LAP from 3 to 20 mmHg (P=0.03 for both), whereas PA flow did not change significantly. The I2O1 analysis yielded an accurate estimation of PAZ at baseline and TAL/BT, judging from high coherence function >0.9 over the wide range of frequency. In contrast, the I1O1 analysis estimated PAZ reasonably well only at baseline. The coherence function associated with TAL/BT decreased to 0.5 in the low frequency range (below 1.0Hz), and PVZ estimated by the I1O1 analysis showed large errors in the low frequency range: modulus expressed in common logarithm 0.154 ± 0.04 (corresponds to 74% of PVZ modulus estimated by the I2O1 analysis at 0.12Hz), and phase 0.386 ± 0.136 radians (568% of PVZ phase estimated by the I2O1 analysis at 0.12Hz), respectively. In conclusion, increased LAP waves can affect PAZ estimation in the low range of frequency.

### [3P-082]

### Cordycepin inhibits CDK1/TERT signaling and the augmented proliferation of pulmonary artery smooth muscle in pulmonary hypertension

*GAOPENG LI[1], XIAODONG LI[1], Lin-Hai Kurahara[1], Kaori Ishikawa[2], Ryo Ishikawa[3], Kazufumi Nakamura[4], Katsuya Hirano[1] ([1]*Department of Cardiovascular Physiology, Faculty of Medicine, Kagawa University,* [2]*Department of General Internal Medicine, Kagawa University Hospital,* [3]*Department of Diagnostic Pathology, Kagawa University Hospital,* [4]*Department of Cardiovascular Medicine, Faculty of Medicine, Okayama University*)

Purpose: Pulmonary hypertension (PH) is characterized by pulmonary vasoconstriction and vascular remodeling due to excessive growth of the pulmonary artery smooth muscle cells (PASMCs). Telomerase reverse transcriptase (TERT), a catalytic subunit of telomerase complex, possess RNA-dependent RNA polymerase (RdRP) activity, which augments cell proliferation, when phosphorylated and thereby activated by cyclin-dependent kinase 1 (CDK1). Cordycepin, an active ingredient of Cordyceps militaris, inhibits the viral RdRP. The present study explored the effect of cordycepin on CDK1/TERT signaling and cell proliferation of pulmonary arterial smooth muscle in PH.
Methods: A rat PH model was prepared by a single subcutaneous injection of 60 mg/kg monocrotaline (MCT) in SD rats. Cordycepin treatment (15 mg/kg/day) in drinking water was started 10 days after MCT injection. Echocardiography and histopathological analysis were performed 21 days after MCT injection. Immunoblotting and immunofluorescence staining were employed to evaluate the expression of CDK1 and TERT phosphorylated at T249 (P-TERT) in lung tissues and PASMCs both derived from patients with PH. MTT assay was used to analyze the proliferation of PASMCs.
Results: MCT rats exhibited right ventricular (RV) hypertrophy, reduced RV fractional shortening, and medial wall thickening of pulmonary artery. Cordycepin ameliorated RV hypertrophy, RV dysfunction, and medial wall thickening, as well as prolonged the survival of MCT rats. Cordycepin reduced a fraction of P-TERT-positive cells in vascular area of the MCT rats. The lung tissues of the PH patients showed a significantly higher fraction of CDK1/P-TERT double-positive cells than in non-PH lung. RO-3306 (5 μM), a CDK1 inhibitor, significantly reduced P-TERT levels, while RO-3306 and cordycepin significantly inhibited the proliferation in PASMCs derived from PH patients.
Conclusion: Cordycepin inhibits CDK1/TERT signaling and proliferation of PASMCs in PH. Cordycepin holds a potential as a novel remedy for the treatment of PH.

### [3P-083]

### The effect of left ventricular venting on the coronary circulation during VA-ECMO support in normal and failing heart

*Yuki Yoshida[1], Kei Sato[1], Shohei Yokota[1], Hiroki Matsushita[1], Hidetaka Morita[1], Akitsugu Nishiura[1], Masafumi Fukumitsu[1], Kazunori Uemura[1], Toru Kawada[1], Keita Saku[1] ([1]*National Cerebral and Cardiovascular Center*)

PURPOSE
Veno-arterial extracorporeal membrane oxygenation (VA-ECMO) is an important intervention in the management of cardiogenic shock (CS). However, in cases of VA-ECMO induced pulmonary oedema, left ventricular (LV) venting is prerequisite. Although the hemodynamic effects of VA-ECMO and LV venting are well understood in the clinical, it remains unknown how those combination impacts on the coronary circulation. We investigated that the effect of LV venting for VA-ECMO on coronary artery characteristics and coronary blood flow (CBF) in normal and failing heart.
METHODS
Eight dogs were used under general anesthesia. VA-ECMO was established with a centrifugal pump. An LV cannula was inserted from the left atrium into the left ventricle and connected to the outflow cannula. CS was induced by ligation of the left ascending coronary artery. We simultaneously recorded arterial pressure (AP), LV pressure (LVP) and CBF, and adjusted VA-ECMO flow by 1-1.5 L/min and the degree of LV venting to keep peak LVP below 10 mmHg. We compared hemodynamics and CBF between VA-ECMO and VA-ECMO with LV venting under normal and CS conditions.
RESULTS
Nineteen data sets were obtained (two dogs died during preparation). In both normal and CS conditions, VA-ECMO increased mean AP and peak LVP. LV venting markedly decreased peak LVP in both the normal (99±19 vs. 11±12 mmHg) and the CS (85±18 vs. 4±4 mmHg), indicating the establishment of LV unloading. Despite the significant increase in coronary perfusion pressure, LV venting slightly decreased CBF in the normal heart (54±38 vs. 39±24 ml/min, p<0.05), whereas did not change it in the CS (43±21 vs. 38±17 ml/min, ns). LV venting increased coronary vascular resistance in both the normal (1.1±0.6 vs. 3.3±1.9 mmHg/ml/min, p<0.01) and the CS heart (0.7±0.4 vs. 2.7±1.4 mmHg/ml/min, p<0.01).
CONCLUSIONS
In the ECMO-supported condition, the increase of coronary perfusion pressure by LV venting did not increase the coronary flow, especially in the normal condition. Coronary autoregulation may determine CBF during LV unloading.

### [3P-084]

### Effect of individual different exercise pressor on cerebral circulation and function.

*Narumi Kunimatsu[1], Shotaro Saito[1], Marino Karaki[1], Hayato Tsukamoto[2], Shigehiko Ogoh[1] ([1]*Toyo University,* [2]*Waseda University*)

Isometric exercise, especially, results in a notable elevation of arterial blood pressure (ABP), which could potentially be problematic for individuals with cerebral vascular disease. However, the influence of this heightened ABP on cerebral circulation and its subsequent impact on cognitive function remains insufficiently comprehended. The aim of the current study was to explore the repercussions of exercise induced ABP elevation on cerebral circulation and cognitive function in young, healthy individuals. Thirty young participants (aged 21.4±1.2 years) completed the interval handgrip (IHG) exercise protocol, which involved four 2-minute handgrip (HG) exercise trials at 25% of their maximum voluntary contraction, with a 3-minute recovery period between each trial. We measured various parameters, including the mean blood velocity (MCA Vm) of the right middle cerebral artery, mean arterial pressure (MAP), and partial pressure of end-tidal carbon dioxide ($P_{ET}CO_2$), both before and during the left-hand IHG exercise. Furthermore, a cognitive task (Go/No-Go task) was administered to assess cognitive function before and after the HG exercise protocol. To investigate the impact of varying responses in ABP increase to the HG exercise protocol, we categorized 16 out of 30 participants into two groups: the high ABP response group (n=8, +35.9±3.4 mmHg) and the low ABP response group (n=8, +8.6±4.9 mmHg, p=0.001). Significant differences were observed in MCA Vm, but not in MCA CVCi and $P_{ET}CO_2$, between the two groups (p=0.028, p=0.938, and p=0.616, respectively). Cognitive function improved in the low ABP response (P=0.008), while there was no significant improvement in the high ABP response group (P=0.424). These findings suggest the variances in individual pressor responses to exercise may influence exercise-induced cognitive function improvement in young individuals, independent of cerebral circulation.

### Poster

### [3P]
### Respiration

March 30, 13:00 - 14:20, Poster Room

### [3P-085]

#### Respiratory rhythm is associated with a recalibration of body perception.

*Miku Kosuge[1], Motoyasu Honma[1], Yuri Masaoka[1], Shota Kosuge[1], Momoka Nakayama[2], Shotaro Kamijo[1], Yusuke Shikama[1], Masahiko Izumizaki[1] ([1]*Showa University*, [2]*Dentsu Lab Tokyo*)

Change in body perception requires recalibration of various sensory inputs. In the rubber hand illusion (RHI), an artificial hand is touched synchronously and tactile stimuli of one's own hand are perceived as if the artificial hand were a part of one's body when the observer's hand is not in view. The RHI causes a recalibration of the spatial location of visual and somatosensory cues, resulting in a distorted perception of the spatial location of the observer's hand and imbues a sense of ownership to the rubber hand. However, it is less known how information other than sensations relates to the recalibration of body perception. Here, we focused on the relationship between respiration and cognition and investigated whether respiratory rhythms are related to the recalibration of hand perception. To examine the effect of respiratory rhythm on hand perception (ownership and location sense) in the RHI, we built a visual feedback environment, in which a mannequin hand moved in conjunction with its own respiratory rhythm, and participants performed an experiment under conditions in congruency/incongruency for spatial and temporal factors. Thirty-eight healthy persons participated in the experiments.　The temporal and spatial congruency between own respiratory rhythm and the mannequin hand markedly facilitated the phenomenon of hand ownership sense transfer to the mannequin hand, while incongruency had little effect on the change in hand ownership. In contrast, the congruency had no effect on location sense.　The findings showed that recalibration of hand ownership in the RHI is enhanced by satisfying the spatial similarity and temporal synchrony conditions between the respiratory rhythm and mannequin hand. This suggests that hand ownership transfers to a real object by the integration of visual information and respiratory rhythm without somatosensory input. The current study proposes that an internal model in the brain allows respiratory rhythms to be involved in the adaptation of the body's neural representations.

### [3P-086]

#### Direct observation and characterization of coughing in mice

*Shogo Soma[1], Akiyuki Taruno[1,2] ([1]*Kyoto Prefectural University of Medicine*, [2]*JST-CREST*)

Despite growing indirect evidence by sound and whole-body plethysmography, skepticism regarding the ability of mice to cough persists. The lack of a robust animal model for cough research has limited the application of advanced technologies and impeded progress in cough medicine. In this study, we directly assessed coughing in mice using a multifaceted approach involving measurements of respiration dynamics, electromyographic activities, and vocal fold movements. Our key observations include that (i) typical tussive stimuli, such as citric acid, consistently elicit a respiratory reflexive response consisting of a deep inspiration with a dilated glottis and a subsequent strong expiration with a narrowed glottis, which is a phenotypic hallmark of a cough in other species; (ii) the reflex involves a characteristic temporal activation pattern of the diaphragm (inspiratory muscle), external oblique abdominis (expiratory muscle) and thyroarytenoid muscle (a vocal fold adductor muscle), which is commonly observed in other species' coughs; and (iii) the reflexive response disappears after bilateral transection of the superior laryngeal nerve, which mediates the cough reflex in other species. Collectively, when compared to coughs in other species, the reflex in mice exhibits substantial similarities in terms of receptors, afferent pathways, efferent pathways, and physiological responses. These shared characteristics exclude other respiratory reflexes. Thus, we conclude that the mouse reflex represents a cough or, at the very least, is equivalent to coughs in other species. This study establishes an experimental model for cough research in mice, which will serve as a strong driving force towards enhancing our understanding of cough mechanisms and fostering novel advancements in diagnosing and treating cough-related diseases.

### [3P-087]

#### Involvement of the serotonergic system in the neural circuits for the stress-related respiratory response from the lateral habenula.

*Riko Mizukami[1,2], Masayuki Matsumoto[3,4], Tadachika Koganezawa[1,4] ([1]*Department of Neurophysiology, Institute of Medicine, University of Tsukuba, Tsukuba, Ibaraki, Japan*, [2]*Doctoral Program in Neurosciences, Graduate School of Comprehensive Human Sciences, University of Tsukuba, Tsukuba, Ibaraki, Japan*, [3]*Department of Cognitive and Behavioral Neuroscience, Institute of Medicine, University of Tsukuba, Tsukuba, Ibaraki, Japan*, [4]*Transborder Medical Research Center, University of Tsukuba, Tsukuba, Ibaraki, Japan*)

Stress stimuli cause a fight or flight response and freezing response in behavior, as well as specific involuntary responses of blood circulation and respiration. The cardiovascular and respiratory centers in the brainstem regulate stress-related physiological responses. However, the details of the neural circuits underlying these responses are still unclear. Previously, we have reported that the lateral habenula (LHb) modulates stress-induced cardiovascular responses. The LHb is originally known to be involved in stress-induced behavior and learning by sending indirect projections to the monoaminergic system. Our previous report also indicated that the serotonergic system partly mediates the LHb-induced cardiovascular responses. Therefore, this study investigated whether the serotonergic system mediates the LHb-originating respiratory response. Thus, We observed the effect of blocking the serotonergic receptors on the LHb-induced respiratory response. We used urethan-anesthetized Wistar male rats. Blood pressure was measured through a catheter inserted into the femoral artery, and heart rate was counted from the electrocardiogram. Respiratory movements were measured by the isotonic transducer attached to the dorsal neck of the rats. Electrical stimulation was applied by the electrode inserted into the LHb. An antagonist of serotonergic receptors, methysergide, was administered via the femoral vein. We observed the effect of blocking the serotonergic receptors on the LHb-induced respiratory response. As a result, the LHb activation increased the respiratory frequency and the minute ventilation but did not significantly change the tidal volume. The administration of methysergide enhanced the LHb-induced increase in respiratory frequency. However, the blockade of serotonergic receptors did not affect the LHb-induced increase in the minute ventilation because the blockade negatively changed the response in the tidal volume. These results suggested that the serotonergic system may be involved in the neural circuits for the respiratory responses from the LHb, and suppress the excessive elevation of respiratory frequency without changing the minutes ventilation.

### [3P-088]

### Donepezil (Aricept(R)) affect ventilation in mice

*Isato Fukushi[1,2], Kotaro Takeda[2,3], Akira Umeda[4], Yasumasa Okada[2] ([1]*Aomori University of Health and Welfare,* [2]*Murayama Medical Center,* [3]*Fujita Health University,* [4]*International University of Health and Welfare Shioya Hospital*)

Donepezil (Aricept(R)), an acetylcholinesterase inhibitor, has been widely used for the treatment of dementia. Because acetylcholine is a crucial neurotransmitter in the brainstem respiratory $CO_2$ sensing mechanism, concerns could be raised whether donepezil affects hypercapnic ventilatory responses. However, the effects of donepezil on ventilation have not been well clarified. We investigated the hypothesis that donepezil could augment hypercapnic ventilatory responses. We measured ventilation by whole body plethysmograghy in conscious, spontaneously breathing, male mice, consisting of young (7 week-old, n=10) and geriatric (65 week-old, n=10) groups. We analyzed ventilatory parameters sequentially in room air, 100% $O_2$, 2% $CO_2$, 100% $O_2$, 8% $CO_2$ and 100% $O_2$ conditions before and after injection of two doses of donepezil (0.2 mg/kg and 2.0 mg/kg). Respiratory parameters (tidal volume, respiratory rate, and minute ventilation) were calculated from the measured respiratory flow. The carbon dioxide concentration in the chamber was monitored with a carbon dioxide concentration analyzer. In results, both age groups showed a moderate ventilatory increase at 2% $CO_2$ and a pronounced increase at 8% $CO_2$. Either dose of donepezil did not affect ventilation under 100% $O_2$ or 2% $CO_2$ gas condition. On the other hand, hypercapnic ventilatory augmentation during 8% $CO_2$ was attenuated in low dose of the donepezil-administered conditions as compared to before donepezil-administered condition. Additionally, hypercapnic ventilatory augmentation of geriatric mice was smaller than that of young mice. We conclude that donepezil may have to be carefully used in elderly patients with hypercapnic respiratory failure.

### [3P-089]

### Acute hypoxia has region-specific effects on the ECoGs

*Masashi Kawamura[1], Airi Yoshimoto[1], Nobuyoshi Matsumoto[1,2], Yuji Ikegaya[1,2] ([1]*The University of Tokyo,* [2]*The Institute for AI and Beyond*)

Oxygen is pivotal for animal survival, and reduced oxygen levels in the air can significantly impact peripheral organs. Previous studies have extensively explored hypoxic effects on the respiratory rates and heart rates (Arias-Reyes et al., 2021; Masao H. & Tetsuo N., 1982), as well as cognitive performance (Wang et al., 2022). Additionally, research simulating high-altitude conditions demonstrated electroencephalogram changes, specifically a shift from fast to slow delta oscillations (Akopyan et al., 1984). However, limited knowledge exists regarding the effects of hypoxia on electrocorticograms (ECoGs), particularly concerning oxygen concentration changes during hypoxic conditions.

To fill this knowledge gap, we designed an experimental setup allowing precise control of oxygen concentration while maintaining standard atmospheric pressure (1 atm). This setup enabled us to exclusively examine the impact of oxygen concentration changes on neural activity. ECoGs were recorded simultaneously from the primary motor cortex, primary somatosensory cortex, and anterior cingulate cortex in freely behaving rats exposed to acute hypoxia, while respiratory signals and electrocardiograms (ECGs) were recorded using implanted wire electrodes. Oxygen concentration was manipulated by regulating the flow of oxygen and nitrogen gases into the experimental chamber. We analyzed the power spectrum of ECoGs using fast Fourier transform, focusing on frequency bands (0.3-4 Hz (delta), 4-8 Hz (theta), 8-15 Hz (alpha), 15-30 Hz (beta), and 30-90 Hz (gamma)). The normalized power of each frequency band was calculated to identify specific responses to hypoxia.

Consistent with previous findings, we found increased respiratory and heart rates during hypoxic exposure. Remarkably, we also found a distinct decrease in the power of the alpha frequency band in the primary motor cortex, while the primary somatosensory and anterior cingulate cortex remained unaffected. This alpha-specific change of ECoG power reflects increased excitability in the primary motor cortex (Ros et al., 2010) and may help escape from hypoxic environments. These results also emphasize diverse hypoxic responses across different neocortical regions, indicating the presence of region-specific mechanisms that warrant further investigation.

### [3P-090]

### Simultaneous and comprehensive recording of respiratory neurons using a high-density probe.

*Minako Okazaki[1,2], Tadachika Koganezawa[1,3] ([1]*Dept Neurophysiol, Inst Med, Univ Tsukuba, Tsukuba, Ibaraki, Japan,* [2]*Dr. Prog Neurosci, Grad Sch Comp Human Sci, Univ Tsukuba, Ibaraki, Japan,* [3]*Transborder Med Res Ctr, Univ Tsukuba, Ibaraki, Japan*)

The respiratory center consists of some compartments among the ventral respiratory column. Although the neural network of various neurons generates respiratory patterns and rhythms, the neural properties and the dynamics of the neural network remain unclear. In this study, we aimed to record various types of respiratory neurons in the ventral respiratory column simultaneously. We performed *in situ* arterially perfused preparation of rats and the central respiratory outputs were recorded from the phrenic and vagus nerves. We inserted high-density Neuropixels probes from the dorsal or caudal surface of the brainstem. When the probe was inserted from the dorsal surface of the medulla, the neural activities of a single respiratory compartment were recorded. On the other hand, when the probe was inserted from the caudal end of the brainstem, about 100 single neurons from the ventral respiratory column were simultaneously recorded. These neurons showed various firing patterns according to respiratory phases such as augmenting inspiratory, augmenting expiratory, post-inspiratory, and pre-inspiratory activities. Therefore, the caudal approach using Neuropixels probes can record various respiratory neurons from all respiratory compartments simultaneously. This method will help to unravel the basal composition and dynamics of neural networks according to the respiratory changes.

### [3P-091]

### Elucidation of EMT mechanism in fibrosis of asthmatic airway remodeling focusing on TMEM16A

*Susumu Yoshie[1], Toru Funyu[1], Akihiro Hazama[1] ([1]*Department of Cellular and Integrative Physiology, Graduate School of Medicine, Fukushima Medical University*)

[Background] Airway remodeling caused by asthma triggers, such as allergens, is characterized by structural changes of subepithelial fibrosis, goblet cell metaplasia, submucosal gland hyperplasia, smooth muscle cell hyperplasia, and angiogenesis, leading symptoms such as dyspnea, which cause marked quality of life deterioration. In particular, fibrosis exacerbated by asthma progression is reportedly mediated by epithelial-mesenchymal transition (EMT). However, the molecular mechanism of EMT in fibrosis of asthmatic airway remodeling has not yet been fully clarified.[Methods] BEAS-2B, which is a human airway epithelial cell line, was treated with serum-free medium containing TGF-β1 as an inducer of EMT and/or T16Ainh-A01 as a TMEM16A inhibitor. Morphological change, gene expression, migration, and invasion of those cells were evaluated.[Results] TGF-β1-treated BEAS-2B showed typical phenotypes of mesenchymal cells, judging from morphological change, gene expression, migration, and invasion. Interestingly, T16-inhA01 inhibited TGF-β1-induced EMT in BEAS-2B.[Conclusions] This study suggests that TMEM16A is involved in EMT in fibrosis of asthmatic airway remodeling.

### Poster

[3P]
**Physical fitness and sports medicine**

March 30, 13:00 - 14:20, Poster Room

### [3P-092]

### Acute effects of moderate-high-intensity interval exercise (MHIIE) on immune cell markers and stress hormonal responses: exploring the possible effects of systemic inflammation levels

*Yi-Hung Liao[1], Chun-Chung Chou[2], Hsiao-Kuan Wu[1], Li-Wei Yeh[1], Yi-Yuan Lin[1]
([1]*National Taipei University of Nursing and Health Sciences, Taipei City, Taiwan,* [2]*National Taipei University of Technology, Taipei City, Taiwan*)

Background: The aim of this study was to investigate the acute effects of different levels of systemic inflammation on immune markers and markers of stress during a single bout of moderate to high intensity interval exercise (MHIIE). Methods: A total of 17 healthy, physically active young men participated in this study. Participants underwent resting baseline neutrophil lymphocyte ratio (NLR) levels and were categorized into a low NLR group (NLR = 1.40 ± 0.25; age: 22.5 ± 3.1 years; n = 8) and a high NLR group (NLR = 2.14 ± 0.55; age: 23.2 ± 5.5 years; n = 9) based on NLR median. Next, participants underwent a single session of acute moderate-high-intensity interval exercise (MHIIE; 75%-60% VO2 peak; 7 minutes per cycle; 5 cycles), and blood samples were collected before (EX0), immediately after (EX40), and 30 minutes after (EX70) exercise for cortisol testing and immune cell index analyses. RESULTS: Our results showed that after MHIIE, there were no significant differences between the low and high NLR groups in terms of leukocyte counts, lymphocyte counts, T-cells (CD4 and CD8), immune indices (CD4/CD8 ratio), and natural killer cell indices (CD16+CD56). However, cortisol levels in the low NLR group showed a higher trend of increase after intermittent exercise, compared to a lower increase in the high NLR group. This suggests that high levels of systemic inflammation may attenuate the response to a single acute exercise-evoked NLR elevation, and although the difference between these two groups was not statistically significant (approaching significance, p = 0.064), it warrants further investigation. Conclusion: Our study provides some insight into the complex interactions between moderate- and high-intensity interval exercise and systemic inflammatory status reflecting by NLR. Although no significant changes in immune markers were observed, our study provides an interesting starting point for future in-depth investigations into the relationship between the immune system and stress markers. Further studies may be needed to elucidate the physiological and clinical significance of these changes and to understand the acute response to exercise in different NLR backgrounds (or systemic levels of inflammation).

COI: No

### [3P-093]

### Effects of 12 Weeks of Somatosensory Games on Heart Rate Variability and Sleep-Related Biomarkers in Middle-age women with Poor Sleep Quality

*Yi-Yuan Lin[1], Hsiao-Kuan Wu[1], Chun-Chung Chou[2], Ching Huang[1], Chi Chen[1], Yi-Hung Liao[1] ([1]*National Taipei University of Nursing and Health Sciences, Taipei City, Taiwan,* [2]*National Taipei University of Technology, Taipei City, Taiwan*)

**Background**: The aim of this study was to investigate the effects of 12-week somatosensory games on heart rate variability and sleep-related biomarkers in middle-aged women with poor sleep quality. **Methods:** Twenty-nine women with poor sleep quality (aged 58±6 years old) were recruited as participants assigned into experimental group receiving ring fit adventure exergame (RAF, n=15) and control group without somatosensory games intervention (CON, n =14). The RAF group received ring fit adventure exergame for 60 minutes each time, 2 times a week, for 12 weeks. The control group was not allowed to participate in intervention activities during the study period. The heart rate variability, sleep quality and related hormone indicators included serotonin and cortisol, and high-sensitivity C-reactive protein were measured before and after the 12-week intervention. **Results:** After the 12-week somatosensory games intervention, the total score of Pittsburgh Sleep Quality Index in the RAF group was significantly lower than that in CON group. In time domain variables, standard deviation of all RR intervals (SDNN) and the square root of the mean of the sum of the squares of differences between adjacent RR intervals (RMSSD) were significantly increased in the RAF group, when compared with the CON group. In frequency domain variables, change in the high frequency (HFnu) was significantly higher while change in the low frequency (LFnu) and LF/HF ratio were significantly lower in the RAF group, when compared with the CON group. The change level of serotonin in the RAF group was significantly higher than that in the CON group. There were no significant differences between the two groups in the changes of high-sensitivity C-reactive protein and cortisol. **Conclusion:** The results of this study suggest that somatosensory games might improve sleep quality, as well as increasing serotonin and reducing autonomic nerves in middle-aged women with poor sleep quality.

COI: NO

### [3P-094]

### Effects of Sugar-Sweetened Soy Milk Supplementation and Meridian Massage on Exercise Recovery and Muscle Soreness Status

*Hsiao-Kuan Wu[1], Yi-Yuan Lin[1], Ting-Heng Chou[1], Cheng-Chieh Yang[1], Yi-Hung Liao[1], Chun-Chung Chou[2] ([1]*National Taipei University of Nursing and Health Sciences, Taipei City, Taiwan,* [2]*National Taipei University of Technology, Taipei City, Taiwan*)

Purpose: Sugar-sweetened soy milk contains carbohydrates and proteins, yet it remains unconfirmed whether meridian massage can enhance soy milk's nutritional recovery benefits. This study aims to investigate the combined effects of soy milk nutritional supplementation and meridian massage on exercise performance recovery and muscle repair. Methods: Nine healthy individuals with regular exercise habits participated in a crossover experimental design. Participants underwent two different recovery interventions: (1) Sugar substitute placebo + Chinese meridian massage (CMM/P) and (2) Soy milk supplementation + Chinese meridian massage (CMM/S) after high-intensity interval exercise. Blood markers were measured before and 24 hours post-exercise, and a 5-kilometer time trial was conducted on the first and second days. Results: There were no interaction effects on time trial completion time, power-to-weight ratio, torque, and force between the two groups on both days. However, CMM/S resulted in significantly lower creatine kinase (p = 0.03) and uric acid (p = 0.039) levels compared to CMM/P. Subjective muscle soreness did not differ between the interventions. Conclusion: This study suggests that supplementing commercially available sugar-sweetened soy milk along with Chinese meridian acupoint massage can reduce muscle damage indicators (creatine kinase) and inflammation-related factors (uric acid) but not cycling performance-related parameters on the day following high-intensity interval exercise.

### [3P-095]

### The Serum Biomarkers for Brain Injury during an Annual Periodization in Taekwondo Athletes

*Chun-Chung Chou[1], Yi-Zhen Yang[2], Yu-Chi Sung[3], Yi-Hung Liao[4] ([1]*National Taipei University of Technology,* [2]*University of Taipei,* [3]*Chinese Culture University,* [4]*National Taipei University of Nursing and Health Sciences*)

**Purpose:** The main aim of this study was to investigate the response of serum biomarkers for brain injury (S100-B) during an annual periodization in taekwondo athletes. **Methods:** Nine male elite taekwondo athletes were collected and measured in each period of annual periodization training: pre-season (the week before the general preparation period), general preparation period (the last 2-3 weeks of the general preparation period), specialized preparation period (the last 2-3 weeks of the specialized preparation period), and competition period 1 (the week of the pre-tournament reduction period), and competition period 2 (the last week of competition period). Blood samples were collected in the morning following an overnight fast, and S100-B was measured at each time point. In addition, the present study also recorded body and brain impacts or hits in taekwondo athletes during each match in competition period. **Results:** There were no significant differences between different periods in S100-B levels in taekwondo athletes. During the match, the brain was subjected to an average of 1.11 hits (including both active and passive hits), the body 60.33 times, the hands 9.22 times, and the lower limbs 63.22 times in each match. **Conclusions:** Despite repetitive head impacts during matches, no significant fluctuations in S100B levels were found between periodization phases, suggesting that head impacts were below the threshold required to alter systemic S100B levels in elite taekwondo athletes.

### [3P-096]

### Relationship between changes in lower limb muscle activity and weight bearing during a squat exercise under optokinetic stimulation with a head-mounted display

*Junya Komagata[1], Atsushi Sugiura[2], Atsuya Otsuka[3], Yuki Komatsu[4], Toshihiro Kitama[2] ([1]*Dept of Physical Therapy, Nagoya Women's Univ.,* [2]*Center for Life Science Research, Yamanashi Univ.,* [3]*Dept of Physical Therapy, Health Science Univ.,* [4]*Kamiina Seikyo Hospital*)

Muscle weakness is a major cause of the reduced daily life activity performance in stroke patients. This muscle weakness can be attributed, firstly, to asymmetric weight bearing (WB). In this study, we examined the effects of optokinetic stimulation (OKS) through a head-mounted display (HMD) on postural stability, weight-bearing (WB), and electromyography (EMG) in the lower limbs of healthy subjects. Fifteen healthy students wearing the HMD were instructed to perform squats involving approximately 60 degrees of knee flexion and extension at the pace of a metronome. For OKS, a random dots pattern in a virtual 3D space were presented in horizontal or torsional directions (HOKS or TOKS). In the WB assessment, the sway mean position of the center of pressure (SM) and mean foot pressure (FP) were measured. EMG was recorded from four leg muscles: the vastus lateralis (VL) and vastus medialis (VM), semitendinosus (ST), and femoral biceps (FB). During TOKS, SM shifted significantly in the direction of stimulation, and FP on the stimulated side increased. The mean EMG activity in the same direction showed a clear increase compared to stationary OKS condition. In contrast, no clear changes were observed in both WB and EMG during HOKS. SM had a positive correlation with the mean EMG activity of each muscle ($p < 0.05$). FP had a positive correlation only with the mean EMG activity of VM ($p < 0.05$). These results suggest that OKS during squats effectively increases lower limb muscle activity in the direction of the stimulation and has an impact on asymmetric WB. The combination of squats and OKS may be beneficial for muscle training in stroke patients.

### [3P-097]

### Dynamics of respiratory and energy metabolism during incremental and decremental exercise followed by endurance exercise

*Tadashi Saitoh[1], Tatsuro Hakozaki[1], Rena Ishida[1] ([1]*Yamagata University*)

The aim of this study was to investigate dynamics of respiratory and changes in energy metabolism and concentrations of blood lactate during endurance with incremental and decremental pre-exercise. Eight subjects participated in this study. The protocol of incremental exercise constituted of 5 sets of cycling exercise for 3 min between anaerobic threshold (AT) – Δ60% and AT + Δ60% on a cycling ergometer, with an increase of Δ30% of AT after each set. The decremental exercise protocol was the reverse of the incremental exercise protocol. Subjects performed endurance exercise at intensity of AT for 10 min followed by incremental or decremental exercise. There was also a 2 min rest period before and after the exercise test and between the sets. During the rests, concentrations of blood lactate and blood glucose levels were measured using blood obtained from pricking the subject's fingertip. During the tests, ventilation level, pulmonary gas exchange rates, and electrocardiography were continuously measured. Heart rate was calculated using electrocardiography. Endurance exercise with incremental pre-exercise resulted in more hyperventilation than with decremental one. The respiratory quotient during endurance exercise was higher in the decremental pre-exercise group than in the incremental pre-exercise group. After endurance exercise, the concentrations of blood lactate in the decremental pre-exercise group were significantly lower than that in the incremental pre-exercise group. These results suggest that high-intensity exercise prior to endurance exercise causes hyperventilation during endurance exercise, and that hyperventilation may suppress the function of monocarboxylate transporter.

### [3P-098]

### High post-exercise glucose intake attenuates the hypotensive and arterial destiffening effects in young prehypertensive males

*Hsin-Fu Lin[1], Chih-Yuan Hsueh[1], Kang Tung[2], Ho-Seng Wang[2] ([1]*National Taiwan University,* [2]*National Taiwan Normal University*)

**Background**: A diet with high glucose intake may contribute to arterial stiffening and an increase in cardiovascular risks; a single bout of aerobic exercise may favor reduced blood pressure and arterial stiffness. However, it remains unknown whether glucose intake would attenuate the hypotensive and arterial de-stiffening effects induced by acute aerobic exercise in a dose-dependent manner. **Purpose**: To investigate the effects of different doses of glucose supplementation after a single session of aerobic exercise on postprandial blood pressure, arterial stiffening, and cardiac sympathetic activity in prehypertensive men. **Methods**: By using a single-blind study design, twelve prehypertensive men (28 ± 4 years) were randomly cross-assigned to a control group (placebo, Con), a low glucose group (25 g of glucose, 25 g), and a high glucose group (75 g of glucose, 75 g) at least one week apart. After obtaining the hemodynamic indices (heart rate, blood pressure, and waveforms, heart-brachial pulse wave velocity, systolic time intervals) and blood samples for biomarkers (blood glucose, insulin) before exercise, a 30-minute treadmill running at 65% heart rate reserve (HRR) was performed, and 250 g of glucose supplement was consumed immediately after exercise. The hemodynamic indices and blood samples were also measured at 30, 60, 90, and 120 minutes after exercise. **Results:** An acute bout of aerobic exercise elicited a reduction of brachial and carotid blood pressure 60 min post-exercise after the placebo treatment, whereas 25g and 75g both did not introduce reduced hypotensive responses; the heart-brachial pulse wave velocity (hbPWV) at 60 minutes post-exercise was significantly higher in the 75g group than that in the placebo (75g: 3.35±0.48 m/s; placebo: 3.14±0.40 m/s). Systole time intervals such as calibrated ventricular ejection time (ETc), pre-ejection period (PEP), and total power from heart rate variability analyses decreased significantly 30 minutes after exercise. The changes of insulin in area under the curve (ΔAUC) significantly correlated with the changes of $PEP_{AUC}$ ($r = -0.42$), $PEP/ETc_{AUC}$($r=-0.39$) that were also correlated with the changes of haPWV ($r=-0.5$ and $r= -0.4$) respectively. However, there was no significant correlation between insulin and hbPWV ($r = -0.13$). **Conclusions:** Post-exercise glucose intake attenuates the hypotensive effects induced by acute aerobic exercise in prehypertensive males. The higher dose of glucose intake could elicit an arterial stiffening effect compared with the placebo, suggesting the importance of post-exercise glucose intake control in diet. The increased central arterial stiffness may result from the increased cardiac sympathetic activity induced by the hyperinsulinemic response.

### Poster

### [3P]
### Nutritional and metabolic physiology, Thermoregulation

March 30, 13:00 - 14:20, Poster Room

### [3P-099]

### Effect of loss of claudin-15 on peritoneal mesothelium membrane

*Noriko Ishizuka Takeshita[1,2], Yuyu Yazaki[2], Wendy Hempstock[2,3], Hisayoshi Hayashi[2]
([1]*University of Shizuoka junior college,* [2]*Lab of Physiol, Sch Food and Nutr Sci, Univ of Shizuoka,* [3]*School of Nursing, University of Shizuoka*)

The peritoneum, a membrane enveloping the inner abdominal cavity, comprises a single layer of mesothelial cells on its surface and deeper layers of connective tissue.
In peritoneal dialysis treatment for renal failure, this peritoneum serves as the dialysis membrane, facilitating the extraction of waste products into the dialysate by a presumed process through the mesothelial cells into the peritoneal cavity. Although tight junctions are recognized between mesothelial cells, their specific properties and physiological significance remain unknown. This investigation aims to explore the ion permeability of the peritoneal membrane in mice lacking claudin-15, a protein believed to influence the ion permeability of tight junctions, as seen in the intestine. The study involved isolating the diaphragm and removing its muscle layer to create a one-layer mesocutaneous membrane specimen. Real-time RT-PCR was employed to measure mRNA expression levels. Claudin-2, 3, and 15 expression was evident in the diaphragm, with claudin-15 being the most highly expressed. Fluorescence immunostaining revealed the localization of claudin-15 at cell junctions between mesothelial cells. A comparable staining pattern was observed in the mesentery, indicating uniform claudin-15 expression in the peritoneum within the abdominal cavity. To assess ion permeability, transepithelial electrical conductance was measured using the Ussing chamber method in both wild-type and claudin-15 deficient mice. Additionally, intraperitoneal administration of glucose, subsequent blood glucose level measurements, and an evaluation of peritoneal permeability were conducted. Moreover, besides its barrier function, the peritoneal mesothelium potentially acts as a free-surface mechanical sensor, akin to proposed functions in renal tubules and gallbladder epithelium. This hypothesis suggests the importance of glandular hair-like structures in this function. Therefore, given the presence of such structures in the peritoneal mesothelium, we examined whether claudin-15 deficiency alters this structure using scanning electron microscopy.

### [3P-100]

### Chronic sugar sweetened alcohol overconsumption causes memory impairment in mice

*Yasunobu Yasoshima[1], Shuuta Takahashi[1] ([1]*Div Behavioral Physiology, Grad Sch of Human Sciences, Osaka University*)

Overconsumption of sweetened alcohol is widely prevalent. Excessive alcohol consumption causes memory impairment in human and rodents. However, little is known about underlying cellular and neural mechanisms by which memory is impaired by chronic sugar-sweetened alcohol consumption in mice. To approach the issue, we examined the memory tests in mice with voluntary drinking of sucrose-sweetened 5% alcohol solution, or saccharin-sweetened 5% alcohol solution with limited access procedure under 20-h food deprivation for 4 weeks. Effects of chronic binge-like sucrose or saccharin consumption without alcohol were also assessed. Mice allows to ingest chow during the 4-h of sweet solution access. An open field test, a light/dark box test, an object recognition test, a food location memory test, and a passive avoidance test were examined. After behavioral tests, number of Neuronal N-immunopositive cells as neurons and ionized calcium-binding adapter molecule 1 (Iba-1)-immunopositive cells as microglia were counted. Pure alcohol consumption in the two groups with sweetened alcohol were greater than 10 g/kg/4 h.
We found memory deficit in object recognition memory and spatial memory in mice with sucrose-, but not saccharin-sweetened, alcohol consumption. No alteration of anxiety-like behavior and avoidance learning was found; spatial memory retrieval after alcohol withdrawal for 7 days was also impaired in sugar-sweetened alcohol group. In mouse groups with chronic sucrose- and saccharin-sweetened alcohol consumption, number of Iba-1-positive microglial cells in the dorsal hippocampus significantly decreased in comparison to mice without alcohol consumption. Present results suggest that chronic sugar-sweetened alcohol impairs microglial regulation in the hippocampal memory function, leading to spatial memory impairment, while chronic alcohol consumption mixed with an artificial sweetener on memory has a relatively lower effect on memory.

### [3P-101]

### HMIT-1.3 is essential for 2-deoxy-D-glucose toxicity in *C.elegans*.

*Luna Izuhara[1], Sawako Yoshina[1], Eriko Kage-Nakadai[2], Shohei Mitani[1] ([1]*Tokyo Women's Medical University,* [2]*Osaka Metropolitan University*)

Animals need energy sources from outside as food. Mammalian animals have glucose transporters on the intestinal membrane and absorb digested glucose from food carbohydrates.
*C. elegans is known* as a powerful model organism for genetic analysis of complex biological processes such as development, behavior, metabolism, and aging.
In the laboratory, *C. elegans* is raised on bacterial monocultures, traditionally an *E. coli* strain OP50. Also, *C. elegans* has 20 glucose (hexose) transporter family genes, which are categorized into two; transporters by facilitated diffusion mechanisms (17 genes, collectively named as GLUT) and proton-coupled inositol transporters (3 genes, collectively named as HMIT), but does not have any sodium-dependent glucose transporter (SGLT) homologues.
Additionally, E. coli can use glucose as an energy source by absorbing it with a glucose transporter.
Therefore we asked which transporters are involved in the absorption of glucose in *C. elegans*. To address this issue, we investigated the toxicity of 2-deoxy-D-glucose (2DG) to wild-type and transporter mutant worms because 2DG is imported through glucose transporters.
Result, we show that HMIT-1.3 is essential for 2-deoxy-D-glucose toxicity in *C.elegans*. Also, we found that only *hmit-1.3* mutants are resistant to 2-deoxy-D-glucose among 20 putative glucose transporter homologs in *C. elegans*.

### [3P-102]

### Adipocyte EID1 promotes adaptive thermogenesis

*Takahashi Itsuki[1], Watanabe Yuusuke[1], Sato Tomohiko[2], Miyazaki Mitsue[3], Amano Izuki[4], Nakanishi Takeo[5], Koibuchi Noriyuki[4], Shimokawa Noriaki[1,4] ([1]*Takasaki University Graduate School of Health and Welfare,* [2]*Ota College of Medical Technology,* [3]*Hirosaki University Graduate School of Medicine,* [4]*Gunma University Graduate School of Medicine,* [5]*Faculty of Pharmacy, Takasaki University Graduate School of Health and Welfare*)

In mammals, white adipose tissue (WAT) and brown adipose tissue (BAT) contribute to energy metabolism and thermogenesis, respectively. We recently showed that the EP300-interacting inhibitor of differentiation 1 (EID1) inhibits triglyceride accumulation and increases the expression of thermogenic proteins in human adipose-derived mesenchymal stem cells. However, the in vivo function of EID1 in thermogenesis in adipose tissues remains unknown. In this study, we investigated the crosstalk between EID1 and thermogenesis in adipose tissues in vivo. We generated EID1 transgenic mice (Tg mice) overexpressing EID1 in adipose tissue. Interestingly, when Tg mice were exposed to a cold environment (4°C, 1 h), the expression of thermogenic genes, such as uncoupling protein-1 (Ucp-1) and peroxisome proliferator-activated receptor γ coactivator 1-α (Pgc-1α) increased in BAT. To assess whether these changes were actually linked to an increase in body temperature in Tg mice, we monitored the core body temperature during cold exposure using the Anipill system, with telemetry capsules implanted in the peritoneal cavity. Tg mice maintained a high body temperature during cold exposure. We are currently studying the gene expression mechanism of EID1-mediated thermogenesis after cold exposure.

### [3P-103]

### Metabolic Effects of Skeletal Muscle Glucocorticoid Receptors

*Nan Wang[1], Tetsuya Shiuchi[1], Hiroyoshi Sei[1] ([1]*Department of Integrative Physiology, Tokushima University Graduate School of Biomedical Sciences*)

Skeletal muscle plays an important role in exercise and postural maintenance, but also in the regulation of systemic energy metabolism. Glucocorticoids are important regulators of skeletal muscle mass, and long-term exposure causes muscle atrophy. However, the role of muscle glucocorticoids in the regulation of energy metabolism remains unclear. Therefore, to better understand the role of muscle glucocorticoid signaling, this study examined the effects of chronic glucocorticoid administration on skeletal muscle in mice that are acquired by doxycycline-induced skeletal muscle-specific glucocorticoid receptors knockout (mGRKO), and in wild-type mice (WT). Chronic corticosterone (CORT) drinking induced atrophy of the gastrocnemius muscle, but such CORT-induced muscle atrophy was not seen in mGRKO. In addition, WT and mGRKO mice fed CORT showed decreased locomotor activity and increased scapular adipose tissue and inguinal adipose tissue weights. On the other hand, glucose tolerance improved in WT mice fed CORT, but no change was observed in mGRKO. These findings suggest that corticosterone causes muscle atrophy via muscle glucocorticoid receptors as well as contributes to obesity-related metabolic changes.

### [3P-104]

### Acute Food Intake Induced Thermogenesis in Brown Adipose Tissue in Mice

*Bong Soo Seok[1], Mira Kato-Suzuki[1], Anju Tsukada[1], Zibo Yang[1], Yukino Tamakoshi[1], Yuko Okamatsu-Ogura[1], Kazuhiro Kimura[1] ([1]*Hokkaido University*)

Food consumption generates heat as a byproduct from digestion, absorption, and processing nutrients. An additional heat is produced as a diet-induced thermogenesis (DIT), and the role of brown adipose tissue (BAT) has been suggested. DIT has been reported to be preferentially activated by chronic intake of Western-style diet; however, it is still under debate whether the BAT thermogenesis is specifically induced by Western-style diet. In addition, it is poorly documented whether DIT is caused by a single meal intake or not, especially in experimental animals. The aim of this study is to examine the effect of acute intake of different type of food on DIT and BAT thermogenesis in mice.Thirteen-week-old C57BL/6 male mice were fasted overnight and then fed with a normal diet (ND) or a high-fat diet (HFD) for an hour. When the temperature in the interscapular region, where BAT is located, was monitored via thermal camera before and after the refeeding, the HFD group, but not ND group, showed significantly higher temperature at 30-45 minutes postprandial compared to the fasted groups. Then, we measured the oxygen consumption (VO2), an index of thermogenesis, using indirect calorimetry before and after the refeeding. At 3 hr postprandial, the HFD group showed significantly higher VO2 than the fasted group, while VO2 of the ND group was not significantly different from that of the fasted groups. To measure the gene expression of Uncoupling protein 1 (*Ucp1*), a mitochondrial protein responsible for thermogenesis and the index of the activation of BAT thermogenesis, BAT samples were collected at fasted state or 1, 2, 3, or 4 hr postprandial after ND or HFD refeeding. The *Ucp1* expression in BAT of both the ND and HFD groups significantly increased at 3 hr postprandial compared to the fasting state, and the HFD group maintained the expression level until 4 hr postprandial. The acute HFD, but not ND, intake increased the body temperature and oxygen consumption postprandially. However, *Ucp1* expression significantly increased after the intake of both ND and HFD, although it lasted longer in the HFD group. These results suggest that food intake, regardless of the quality of the meal, activates BAT thermogenesis, but the effect is more potent in HFD than ND.

### Poster

### [3P]
### Behavior, Biological rhythm, Sleep

March 30, 13:00 - 14:20, Poster Room

### [3P-105]

### Effects of ketogenic diet and noradrenergic agents on abnormal behaviors induced by neonatal dopamine depletion.

*Masanori Ogata[1], Kei Eto[1], Hitoshi Ishibashi[1] ([1]*Department of Physiology, School of Allied health Sciences, Kitasato University*)

The ketogenic diet (KD) is a high fat, low carbohydrate and adequate-protein diet, and has shown ameliorative effects on several neurological disorders, such as epilepsy and Parkinson's disease. Recently, we reported that the KD improved some of the abnormal behaviors induced by neonatal dopamine (DA) depletion (The 99$^{th}$ and 100$^{th}$ Annual Meeting of the physiological Society of Japan). The rats with neonatal DA depletion have been used as animal models for attention deficit hyperactivity disorder. Important role of the noradrenergic neural system in KD treatment has been also reported. In the present study, effects of KD, atomoxetine (a selective noradrenaline reuptake inhibitor) and propranolol (beta-adrenergic receptor antagonist) on the abnormal behaviors in rats with neonatal DA depletion were investigated using open field (OF), elevated plus maze (EPM) and 24-hour home cage (24-h) tests. On postnatal day 4, treatments with 6-hydroxydopamine (i.c.v) were performed to induce neonatal DA depletion. The rats were fed normal diet (ND) or KD for 5 weeks after weaning. In the OF and/or EPM tests, ND-fed rats with DA depletion showed increases in locomotor activity, and decreases in anxiety-related and exploratory behaviors. The ND-fed rats with DA depletion also showed a decrease in locomotor activity in the 24-h test. Treatment of KD or atomoxetine, but not propranolol, improved the hyperlocomotor activity of the rats with neonatal DA depletion in the OF test. The ameliorative effects of KD or atomoxetine treatment were not enhanced by the combination of these treatments. The other hand, a combination of KD and propranolol treatments induced ameliorative effects on the decreased anxiety-related behaviors of the rats with neonatal DA depletion in the EPM test. There was no significant effect of propranolol alone on the abnormal anxiety-related behaviors of the rats with neonatal DA depletion in the EPM. Any treatments of them did not improve the decreased exploratory behaviors of rats with neonatal DA depletion in the OF test. These results suggest that several neuronal systems are involved in both the abnormal behaviors induced by neonatal DA depletion and ameliorative effects of KD treatment on the abnormal behaviors, and the noradrenergic neural system appears to be one of them.

### [3P-106]

### Roles of suprachiasmatic AVP neurons on female reproductive functions

*Mizuki Sugiyama[1,2], Jiaxu Chen[1], Michihiro Mieda[3], Takahiro Nakamura[1] ([1]*Laboratory of Animal Physiology, School of Agriculture, Meiji University,* [2]*Major in life science, Graduate school of Agriculture, Meiji university,* [3]*Department of Integrative Neurophysiology, Faculty of Medicine, Kanazawa University*)

The estrous cycle is 4-5 days in female mice, and it is divided into four stages: metestrus, diestrus, proestrus, and estrus. During the late afternoon of proestrus, ovulation is caused by a luteinizing hormone (LH) surge following a gonadotropin-releasing hormone (GnRH) surge. It has been reported that the LH surge did not occur in rodents when the suprachiasmatic nucleus (SCN) was lesioned. It suggests that the preovulatory GnRH/LH surge requires not only high levels of estrogen but also timing signals from the SCN. Although it has been suggested that peptides-producing neurons such as arginine vasopressin (AVP) and vasoactive intestinal peptide are related to the GnRH/LH surge system, the details of the mechanism are still unclear.Our previous study showed *Avp-Vgat -/-* (*Avp-Cre; Vgat flox/flox*) mice, the vesicular GABA transporter (*Vgat*) gene is specifically deleted in AVP neurons, exhibited disrupted estrous cycle and this cycle was restored by injection of adeno-associated virus (AAV- *EF1α-DIO-Vgat-mcherry*) to the SCN. Based on these results, we further investigated the role of suprachiasmatic AVP neurons on female reproductive functions. In the present study, *Avp-Cre* mice whose AVP neurons expressed the light-activated protein (Jaws: red-shifted cruxhalorhodopsin) by injection of AAV (AAV-*CAG-FLEX-Jaws-GFP*) were used, and then using optogenetics technology, the neural activity of suprachiasmatic AVP neurons of the mice was time-dependently suppressed, and its effects on the estrous cycle were investigated. As a result, when AVP neurons were suppressed during the critical window of the LH surge (ZT 9-11 in proestrus), the estrous cycle was prolonged. These results suggest that the timing signal from AVP neurons in the SCN plays an important role in female reproductive functions.

### [3P-107]

### Loss of non-canonical open reading frame within lncRNA *TUNAR* increased pre-pulse inhibition and depression-related behavior in mice

Kazuki Fujii[1,2,3], Yusuke Moriwaki[4], Yumie Koshidaka[2], Mayumi Adachi[2], Yuko Yanagibashi[2], Shoko Hongo[2], Yasunori Aizawa[5], *Keizo Takao[1,2,3,4] ([1]*Department of Behavioral Physiology, Faculty of Medicine, University of Toyama,* [2]*Life Science Research Center, University of Toyama,* [3]*Research Center for Idling Brain Science, University of Toyama, Toyama, Japan,* [4]*Department of Behavioral Physiology, Medicine and Pharmaceutical Sciences, University of Toyama,* [5]*Graduate School of Bioscience and Biotechnology, Tokyo Institute of Technology*)

By definition, long noncoding RNAs (lncRNAs) do not contain protein-coding open reading frames (ORFs). Recent bioinformatics and high-throughput sequencing studies, however, reported that many lncRNAs possess short "non-canonical" ORFs (sORFs) encoding microproteins. These newly identified microproteins may help to elucidate the mechanisms underlying vital phenomena and are potential targets for drug discovery. The Tcl1 upstream neuron-associated RNA (*TUNAR*) was initially discovered as a lncRNA. The *TUNAR* sequence is remarkably conserved across vertebrates and is highly expressed in neural tissues. By in silico screening, we identified that *TUNAR* has an sORF region encoding a 48-amino acid polypeptide. The lncRNA TUNAR plays a vital role in pluripotency and neural differentiation of mouse embryonic stem cells. Recently, it was reported that *TUNAR* and the microprotein regulates neural differentiation in mice. Whether or not and how *TUNAR* and the microprotein affect brain function and behaviors, however, remain unclear. Here, we generated *TUNAR*-microprotein reporter mice and *TUNAR* sORF deletion mice on the C57BL/6J background using the CRISPR-Cas9 genome editing system. Utilizing *TUNAR*-microprotein reporter mice in which an epitope-tag coding sequence was inserted before the stop codon, we detected the epitope tag-specific stained cells in the mouse central nervous system (e.g., thalamus, inferior colliculus, pons). To investigate the function of *TUNAR* microprotein in the brain, we subjected *TUNAR* sORF deletion mice to a comprehensive behavioral test battery. *TUNAR* sORF deletion mice had significantly lower body weight than their wild-type (WT) littermates. In the rotarod test, *TUNAR* sORF deletion mice exhibited better motor coordination compared with WT mice. In the startle response/pre-pulse inhibition (PPI) test, while there was no significant difference in the acoustic startle response, *TUNAR* sORF deletion mice showed increased PPI compared with WT mice. In both the Porsolt forced swim test and tail suspension test, *TUNAR* sORF deletion mice exhibited increased depression-related behavior. These results suggest that the *TUNAR* sORF deletion induces a depressive effect. These findings together indicate that the non-canonical microprotein from *TUNAR* is translated in the brain and has a critical role in sensory-motor gating and depression-related behavior.

### [3P-108]

### The suprachiasmatic nucleus is required for light-induced behavioral rhythms in mice lacking circadian rhythms

*Shota Miyazaki[1], Kazuto Watanabe[1], Nana N. Takasu[2], Takahiro J. Nakamura[1], Wataru Nakamura[2] ([1]*Laboratory of Animal Physiology, School of Agriculture, Meiji University,* [2]*Department of Oral-Chrono Physiology, Graduate School of Biomedical Sciences, Nagasaki University*)

The circadian rhythm exists in almost all organisms on the Earth, controlling behavioral and physiological functions to align with the 24-hour cycle associated with the planet's rotation. *Period* (*Per*) genes are the major clock genes responsible for generating the circadian clock. Three homologs of *Per* genes, *Per1*, *Per2*, and *Per3*, have been identified in mammals. The locomotor activity rhythm of mice lacking all homologs (*Per1/2/3* KO mice) entrains with a 12-hour light /12-hour dark cycle but does not exhibit circadian rhythm under constant darkness. However, light pulses in constant darkness can induce the expression of short-period (15-20-hour) locomotor activity rhythms. The mechanisms underlying the generation of the light-induced behavioral rhythm remain unknown. In the present study, we investigated whether the suprachiasmatic nucleus (SCN) is involved in the light-induced rhythms observed in *Per1/2/3* KO mice. We conducted experiments to lesion the SCN and measure the neuronal firing activity rhythms *in vivo* and *in vitro*. In the experiment where the SCN was unilaterally or bilaterally lesioned in *Per1/2/3* KO mice, the bilateral lesioned group under constant darkness did not show the light-induced rhythm. In contrast, the unilateral lesioned group exhibited a light-induced rhythm similar to the sham-operated group. *In vivo* multi-unit neuronal firing activity rhythm recordings, neuronal firing activity rhythms in the SCN were observed under light-dark conditions but disappeared under constant darkness. However, after a 6-hour light pulse, a rhythm of approximately 19.5 hours, similar to the locomotor activity rhythm, was observed. *In vitro* experiments, the SCN was dissected from newborn *Per1/2/3* KO mice, and dispersed cultures were performed using multi-electrode dishes to evaluate the presence of neuronal firing activity in each SCN neuron. As a result, circadian rhythms in the spontaneous firing frequency of some neurons were detected, even in the absence of stimulation. Additionally, to explore the physiological significance of the approximately 19.5-hour rhythm, female *Per1/2/3* KO mice were housed under a light-dark cycle with a period of 19.5 hours, which resulted in an increased number of individuals showing a stable estrous cycle of 4 to 5 days, similar to wild-type mice. These results suggest that the SCN, as the central circadian clock, is essential for the expression of light-induced rhythms in *Per1/2/3* KO mice, and SCN neurons have the ability to generate short-period circadian rhythms even in the absence of major clock genes.

### [3P-109]

### Participation of the glutamatergic system in the median preoptic nucleus in the drinking response elicited by hypovolemia in rats

*Junichi Tanaka[1], Akihiko Ushigome[2], Makoto Takahashi[1], Mayu Takahashi[3], Yasushi Hayashi[4] ([1]*Naruto Univ. of Education,* [2]*Teikyo Heisei Univ.,* [3]*Osaka Kyoiku Univ.,* [4]*Notre Dame Seishin Univ.*)

The glutamatergic system in the median preoptic nucleus (MnPO) has been suggested to participate in the control of drinking behavior. The present study was designed to investigate whether water ingestion causes changes in the release of glutamate (Glu) in the MnPO elicited by hypovolemia, and whether local application of the *N*-methyl-$_D$-aspartate (NMDA) and non-NMDA antagonists in the MnPO alters the hypovolemia-induced water intake, in freely moving rats. Normotensive hypovolemia was evoked by subcutaneous injection of polyethylene glycol (PEG), extracellular concentrations of Glu were measured using in vivo microdialysis methods. Subcutaneous application of PEG (30%, 5 ml) significantly increased the Glu release in the MnPO. Water intake significantly reduced the increase in the Glu release in the MnPO caused by the PEG treatment. Perfusion of either the NMDA antagonist dizocilpine (MK801, 50 μM) or the non-NMDA antagonist 6-cyano-7-nitroquinoxaline-2,3-dion (CNQX, 50 μM) through the microdialysis probe reduced the amount of water intake induced by the PEG treatment. Neither MK801 nor CNQX administered locally produced a significant alteration in the Glu release in the MnPO. These results provide evidence that the glutamatergic mechanisms in the MnPO may play important roles in the modulation of water intake caused by hypovolemia, and imply that the modulation may be mediated through both NMDA and non-NMDA receptors.

### [3P-110]

### Genetic Blueprint vs. Environmental Imprints: Dissecting the Determinants of Male Attractiveness

*Yoshinori N Ohnishi[1], Yukie Kawahara[1], Yoko Ohnishi[1], Akinori Nishi[1] ([1]*Kurume Univ. School of Medicine, Dept. of Pharmacol.*)

Understanding what constitutes male attractiveness has long been a subject of interest, with debates often centered around the relative contributions of genetics and environment. This study utilizes male mice as a model organism to probe the underpinnings of attractiveness, specifically examining the role of inherent traits versus external influences. Initially, our investigation revealed that female mice's preferences were significantly influenced by visual cues, as indicated by their disinterest in males when visual access was obstructed. However, this preference shifted when females with impaired vision were introduced, suggesting that factors beyond appearance are at play in mate selection. Further observations indicated that social dynamics could adversely affect the attractiveness of a male observed with less attractive counterparts, underscoring the influence of social context on mating behavior. Subsequently, we challenged the heritability of attractiveness through controlled mating experiments. Despite preconceived notions of attractive males achieving greater reproductive success, our results showed a higher mating success rate for males not initially deemed attractive. Further investigation into the offspring of these matings revealed no significant disparity in attractiveness, suggesting that the trait may not be as heritable as once thought. Moreover, the study sought to understand if social dominance, as determined by competitive encounters in a controlled environment, correlated with attractiveness. Intriguingly, no consistent pattern emerged from these interactions, and attempts to alter perceived attractiveness through pharmacological means (specifically, the administration of cocaine) did not yield uniform changes in either competitive outcomes or female preference. In summary, our findings challenge the notion that male attractiveness is solely a product of genetic factors. Instead, they highlight the complex interplay between genetic predisposition, environmental context, social interaction, and individual behavior. This complexity suggests that attractiveness is not a static trait passed down through generations but a dynamic quality influenced by a myriad of factors. These insights not only deepen our understanding of mating behaviors in rodents but may also offer broader implications for other species, including humans.

### [3P-111]

### Synchronization of exercise timing in a social wheel cage requires physical contact between individuals

*Ko Yamanaka[1], Jimmy Kim[1], Hidefumi Waki[1] ([1]*Dept.Physiol. Health and Sports Sci., Juntendo Univ.*)

Although engaging in exercise and sports activities can be effective in improving physical and mental health and preventing disease, it is difficult to maintain motivation for them. A way of effectively maintaining and improving exercise motivation is to exercise with others who are highly motivated to exercise. However, the physiological mechanisms by which exercise motivation is transmitted by others remain unclear. In this study, we aimed to examine how differences in social interactions affect exercise motivation between individuals. Thirty-two male Long-Evans rats (4 weeks old), in 16 separate pairs, were kept in a social wheel cage for 4 weeks. The partition between the cages was replaced weekly as follows: 1) eight pairs of rats were separated either with a wire mesh (“Pair” condition) or black acrylic panels (“Single” condition) and 2) the other eight pairs of rats were separated either with a transparent acrylic (“Transparent” condition) or black acrylic panels (“Single” condition). The number of wheel rotations was measured as the locomotor activity index. Cross-correlation analysis was conducted on the temporal relationship between the timing of exercise in the paired rats. No significant changes were observed in the wheel rotation between the Pair and Single conditions and between the Transparent and Single conditions. Interestingly, the variation in the locomotor indices of individual rats tended to be smaller in the Pair condition than in the Single condition. Furthermore, the cross-correlation analysis revealed higher synchronization in timing of exercise between the rats in the Pair condition compared to those in the Single condition ($p < 0.05$) but not in the rats in the Transparent condition compared to those in the Single condition ($p > 0.05$). These results suggest that the synchronization in the timing of exercise between rat-pairs in a social wheel cage may require social interaction involving physical contact, rather than visual influence.

### [3P-112]

### Evaluation of activity, body temperature and sleep fluctuation in shift work model mice

*Hiroaki Fujihara[1], Takeshi Ebara[1] ([1]*Department of Ergonomics, Institute of Industrial Ecological Science, University of Occupational and Environmental Health*)

We created an animal model of shift work by using mouse with feed restriction and exercise restriction by running wheel (RW). We used C57BL/6 mice and evaluated electroencephalogram (EEG) and electromyogram (EMG) for sleep stage, ambulatory activity, RW activity and body temperature. The mouse was surgically implanted a radio transmitter into the peritoneal cavity for measuring ambulatory activity and body temperature, and the electrodes for measuring EEG and EMG under sevoflurane anesthesia. After the one week of recovery period, one week of baseline was recorded. After the baseline period, in shift work model group, the mouse was permitted to run on the RW and to eat the food during only light period and limited both of them in the dark period. In control group, we permitted them during the dark period only. Throughout the experiment, amount of ambulatory activity, RW activity, body temperature, EEG and EMG were recorded. In shift work model group, peaks of ambulatory activity shifted from the dark period to the light period in the first day of the feeding and RW restriction. Body temperature decreased in the latter half of the dark period and increased in the first half of the light period. NREM sleep increased in the dark period and arousal increased in the light period in the shift work model group. These results suggested that the biological changes in this mouse model might be equivalent to those of human in shift work condition.

### [3P-113]

### Molecular regulatory mechanisms of after-effect in the mammalian circadian rhythm

*Keisuke Ikegami[1], Masubuchi Satoru[2], Shigeyoshi Yasufumi[3] ([1]*Laboratory of Regulation in Metabolism and Behavior, Faculty of Agriculture, Kyushu University,* [2]*Department of Physiology, School of Medicine, Aichi Medical University,* [3]*Department of Anatomy and Neurobiology, Kindai University Faculty of Medicine*)

The suprachiasmatic nucleus (SCN) in the anterior hypothalamus acts as a circadian pacemaker in mammals, which senses ambient light information from the retina, to manage daily body rhythms. This light information not only has a short-term effect resetting locomotor activity rhythms such as sleep-wake in a time-dependent manner, but also a “after-effect” in which light pulse of just a few minutes changes period of locomotor activity rhythm for a long time (several weeks or more). Although the correlation between phase shifts and periodic changes can help quickly adapt to irregular light stimuli during jet lag or at night, the mechanisms, one of the great mysteries in biological clock research, remain unknown.

We have discovered CBA/N mice after-effect-deficient, but C57BL/6J mice sufficient. Since CBA/N can produce melatonin from the pineal gland, when we performed pinealectomy in CBA, the arrested after-effect was rescued, indicating pineal humoral factor regulating after-effect. SCN ex vivo analysis after nocturnal light pulse to both mice strains revealed longer period in C57BL/6 but no change in CBA/N, suggesting semi-irreversible changes in the SCN. Furthermore, RNA-seq analysis of the SCN in these mice showed the possibility that myelination of oligodendrocytes in the SCN nerve axonal myelin sheath can trigger after-effect and alter the SCN neural network leading to changes in the response to light stimulation. Since demyelination by cuprizone feeding in C57BL/6 mice tend to suppress the after-effect of locomotor activity by nocturnal light pulse, myelination may be involved in the nighttime photo-responsiveness in after-effect. Now we are investigating the effect of myelination on the persistence of after-effect.

### Poster

### [3P]
### Stress

March 30, 13:00 - 14:20, Poster Room

### [3P-114]

### The role of $5HT_{1A}$ receptors on the cardiovascular response and central neural excitability during social defeat stress in the rat.

*Mio Matsuyama[1], Joji Horiuchi[1] ([1]*Department of Biomedical Engineering, Toyo University*)

Sympathetic cardiovascular responses such as hypertension and tachycardia induced by psychological stressors are mediated by the rostroventral medulla (RVM) and medullary raphe (MR) from the dorsomedial hypothalamic area (DMH), one of the stress centers. A previous study has shown that central administration of serotonin (5HT) receptor agonist inhibits the stress-like cardiovascular responses evoked by the DMH stimulation. This indicates that 5HT and 5HT receptors may play an important role in stress-induced cardiovascular responses. However, the action sites of 5HT in the brain remain unclear, and the role of central serotonin on the actual stress-induced cardiovascular response is poorly understood. In the present study, conscious rats were administered $5HT_{1A}$ receptor blocker intraventricularly, and we compared their cardiovascular responses and central neural excitability to those of the control group during the social defeat stress (SDS) challenge. Before implantation of the telemetry probe for measuring arterial pressure (AP) and heart rate (HR), a pipe was inserted and fixed in the brain for administering the drug intraventricularly. The intraventricular injection of WAY-100635 (0.5 mg/ml, 4 µl), the $5HT_{1A}$ receptor antagonist, was made before the SDS challenge. In the control group, initial increases in AP and HR were observed just after starting the SDS challenge, and the AP and HR decreased gradually but still kept higher levels of the pre-SDS challenge. In contrast, central administration of WAY-100635 suppressed the cardiovascular responses to the SDS throughout the stress period. In terms of neural excitability, the distribution of c-Fos-expressed neurons in the DMH and PeF exhibited a significant increase. In contrast, the number of c-Fos expressed neurons in the MR, which is the descending pathway for stress-induced tachycardic responses, was significantly suppressed, while there was no clear difference observed in c-Fos expression in the RVM, which constitutes the descending pathway for stress-induced hypertensive responses. These results suggested that central $5HT_{1A}$ receptors modified an excitatory role in cardiac function induced by acute psychological stress such as the SDS, possibly by mediating the enhancement of the MR neural excitability, whereas they played the suppressive role of neuro-excitability of the hypothalamic stress centers.

### [3P-115]

### Evaluation of stress tolerance in depressive state in rats: using neural excitability and cardiovascular response as indicators

*Shouta Ushikubo[1], Mio Matsuyama[1], Joji Horiuchi[1] ([1]*Department of Biomedical Engineering, Toyo University, Japan*)

It is known that prolonged or excessive stress can cause psychiatric symptoms such as depression and/or physical symptoms such as hypertension. It is also known that depression and hypertension are highly related because many patients suffer from both. The relationship between stress-related hypertension and depression is not clear, but it is possible that depressed patients overreact to everyday stressors. In this study, we induced depression in conscious rats by chronic restraint stress and observed changes in stress-induced cardiovascular responses and neural excitability in the brain, especially in the hypothalamus and medulla, before and after acute psychological stress, air-puff stress, to evaluate stress sensitivity in the depressed state. Wistar rats were implanted with a telemetry probe to measure blood pressure (BP) and heart rate (HR) under freely moving conditions. After a week-long recovery period, the rats were subjected to an open-field behavioral test to evaluate their depressed states and to assess stress sensitivity by exposing air-puff stress. The following day, rats in the experimental group were subjected to restraint stress for 2 hours a day for one week, and as a control group, rats were allowed to move freely in their cages for a week instead of restraint stress. The day after the last day of restraint stress, the open-field behavioral test and air-puff stress were performed again. The rats were then deeply anesthetized, and the brains were fixed and removed. The sectioned brain was subjected to immunohistochemical staining of c-Fos, a neuroexcitatory marker. In the cardiovascular parameters, significant suppression was observed in the stress-induced tachycardic response, indicating that cardiac function may be less sensitive to stress, although no difference was observed in BP to air-puff stress challenge in the depressed rats. Moreover, neural excitability in the dorsomedial hypothalamic area (DMH) was decreased compared to the control group. However, neural excitability was increased in the medullary raphe (MR), the sympathetic descending terminal of the tachycardic response during stress. These results suggested that the discrepancy between the tachycardic response and the neural excitabilities of DMH and MR during depression was not due to the neural activity of MR itself, but the parasympathetic system and another brain region might be involved in the response.

### [3P-116]

### Elucidating the usefulness of mastication in depression

*MIE KAMATE[1,2], Hitoshi Teranishi[1], Kenshiro Shikano[1], Ryohei Umeda[1], Kenji Kawano[2], Reiko Hanada[1] ([1]*Oita University Faculty of Medicine Department of Neurophysiology*, [2]*Oita University Faculty of Medicine Department of Oral and Maxillofacial Surgery*)

Modern society is a "stress society", and it is known that excessive stress destroys the homeostasis in health and progresses to mental and neurological disorders such as depression. It has been reported that the " mastication "  decreases stress levels, however the details of this phenomena and molecular mechanisms are not fully clarified yet. Groups of mice with different chewing intensity (solid feed group and powder feed group) were subjected to “Repeated Social Defeat Stress (R-SDS)”, and behavioral analysis was performed. To address examine, a social interaction test (SIT) was performed after 10 days of R-SDS. As a result, in SIT, the time spent in the stress avoidance zone was significantly increased in the powder-fed group of the defeat group mice compared to the solid-fed group.Furthermore, we examined neurotransmitter dynamics such as catecholamine and serotonin, acetylcholine, excitatory glutamic acid, inhibitory GABA in R-SDS using LC/MS/MS systems. In Defeat group mice, it was revealed that serotonin in the dorsal hippocampus was significantly decreased in the powder-fed group compared to the solid-fed group.In addition, analysis using quantitative PCR revealed that in the Defeat group of mice, Iba-1, a microglial marker, was significantly increased in the ventral hippocampus of the powder-fed group compared to the solid-fed group.From the above, it was found that differences in food texture (differences in mastication intensity) affected stress resilience, and that a decrease in masticatory strength reduces resilience to stress. We plan to elucidate even more detailed molecular mechanisms in the future.

### [3P-117]

### Serum leptin contributes to development and persistent of muscular hyperalgesia induced by Repeated Cold Stress

*Teruaki Nasu[1], Hotta Norio, Kazue Mizumura[2], Kimiaki Katanosaka[1] ([1]*College of Life and Health Sciences, Chubu University,* [2]*Department of Physiology, Nihon University School of Dentistry*)

<Introduction>Chronic musculoskeletal pain is one of the symptoms of patients with overeating. Leptin, has physiological roles in control of feeding and autonomic nervous system . Furthermore, there are several reports that leptin makes neuropathic pain worse. Therefore, it is possible that leptin contributes to chronic musculoskeletal pain, but has not been examined. whether leptin contributes or not. Therefore, we measured the serum leptin levels and its effect on muscle pain in repeated cold stress (RCS) rats, which is characterized with chronic musculoskeletal pain and used as a model of fibromyalgia.<Method and Result> We measured leptin concentration in the control group and RCS group by ELISA (Enzyme-Linked Immuno Sorbent Assay) .We evaluated muscular mechanical withdrawal threshold with the Randall-Selitto method to investigate change of serum leptin concentration was involved in muscle pain. Serum leptin concentrations in the RCS group were significantly lower than those in the healthy group. Subcutaneous administration of leptin for 7 days reversed the mechanical hyperalgesia caused by RCS exposure. Furthermore, this analgesic effect of leptin was inhibited by co-administration of Compound C, an AMPK (AMP-activated protein kinase) inhibitor.<Conclusion> It is suggested that leptin has analgesic effect on muscular hyperalgesia induced by RCS, and it is mediated by AMPK. Considering the results, decrease of serum leptin level in RCS rats contributes to chronic muscle pain.

### [3P-118]

### Relationship between increased intestinal motility and the parasympathetic nervous system by stimulation of the hypothalamic stress center

*Naoya Kikuchi[1], Mio Mathuyama[1], Joji Horiuchi[1] ([1]*Department of Bioengineering. Toyo University*)

Psychological stress is known to cause stress-induced cardiovascular reactions such as hypertension and tachycardia. On the other hand, irritable bowel syndrome (IBS), one of the stress-related diseases, is classified into diarrhea, constipation, and mixed types according to its symptoms. Still, its detailed mechanism has not yet been elucidated. In particular, diarrhea symptom is thought to be caused by increased intestinal motility due to parasympathetic dominance, which is inconsistent with a stress-induced cardiovascular response. We hypothesized that acute psychological stress may upset the balance of the autonomic nervous system and affect intestinal motility. In this experiment, we activated the neurons in the dorsomedial hypothalamic area (DMH; a candidate of the stress center) and investigated mechanisms of intestinal motility during DMH stimulation. Stimulation of neurons in the DMH caused cases in which intestinal motility was enhanced along with stress-like cardiovascular response. In addition, when Atropine, a cholinergic receptor blocker, was administered intravenously in the case of an animal with increased intestinal motility by stimulation of the DMH, a decrease in intestinal motility was observed. These results suggest that the increase in intestinal motility induced by DMH stimulation was caused by enhancing the parasympathetic nerve activity. However, the discrepancy between DMH stimulation-induced parasympathetic intestinal hypermotility and stress-induced cardiovascular responses has not been elucidated. Therefore, we investigated the participation of the dorsal motor nucleus of the vagus nerve in the medulla (DMN) as a descending tract from the DMH on the intestinal movements during DMH stimulation. Blocking the DMN did not affect the enhanced intestinal motility induced by DMH stimulation. This indicated that the intestinal hypermotility caused by DMH may not be mediated via the DMN. The parasympathetic nervous system, which has a connection between the intestinal tract and the central nervous system, includes the hypogastric nerve that controls the lower intestinal tract, and the result suggested that the hypogastric nerve might involve in the enhancement of intestinal motility by DMH stimulation.

### Poster

### [3P]
### Pathophysiology

March 30, 13:00 - 14:20, Poster Room

### [3P-119]

### Evaluation of danger-associated molecular patterns (DAMPs) dynamics in a zebrafish model of gut inflammation

*Mengting Shan[1], Ryohei Umeda[1,2], Shinichiro Kume[3], Phurpa Phurpa[1], Magdeline Elizabeth Carrasco Apolinario[1], Kenshiro Shikano[1], Hitoshi Teranishi[1], Takatoshi Hikida[4], Yulong Li[5], Reiko Hanada[1] ([1]*Department of Neurophysiology, Faculty of Medicine, Oita University,* [2]*Department of Advanced Medical Sciences, Faculty of Medicine, Oita University,* [3]*Department of Cardiovascular Sciences, Faculty of Medicine, Oita University,* [4]*Laboratory for Advanced Brain Functions, Institute for Protein Research, Osaka University,* [5]*State Key Laboratory of Membrane Biology, Peking University School of Life Sciences*)

**Background** Recently, changes in dietary habits have led to an increase in the number of people suffering from inflammatory bowel disease (IBD) and irritable bowel syndrome (IBS). These diseases are intractable, have a wide variety of causes, and have not yet developed treatments. The gut and immune systems are closely integrated and functionally interdependent. DAMPs, particularly adenosine triphosphate (ATP) and its metabolite adenosine (Ado), play an important role in detecting endogenous stress signals. But the pathogenesis of ATP/Ado in gut inflammation is unclear. Therefore, this study aims to assess the pathophysiological role of ATP and Ado in a zebrafish model of IBD or IBS. **Methods** This study has two main experiments: one is development of drug-induced IBD or IBS model in zebrafish, and the second is to establish transgenic zebrafish with fluorescent ATP/Ado sensor, which is $GRAB_{ATP}$ or $GRAB_{Ado}$, to examine in vivo dynamics ATP/Ado related to disease progression. Lipopolysaccharides (LPS) was used to induce IBD, and 2,4,6-Trinitrobenzenesulfonic acid (TNBS) was used to induce IBS in wild type zebrafish (WT). IBD or IBS is evaluated with some morphological change such as the number of goblet cells, proliferating villi, cell infiltration, and examined proinflammatory cytokine levels. At the same time, plasmid containing Fabp2-$GRAB_{ATP}$ and Fabp2-$GRAB_{Ado}$ were developed. And each plasmid was co-injected with Tol2 transposase mRNA into one-cell stage of zebrafish eggs. We will treat $GRAB_{ATP}$ or $GRAB_{Ado}$ zebrafish to develop IBD or IBS as same as we did on WT to evaluate ATP/Ado extracellular levels in gut. **Result** LPS or TNBS treatment showed significantly higher number of goblet cells and proliferating villi compared to control group. Intestinal lumen was significantly narrower in LPS treated WT compared to control group. And $GRAB_{ATP}$ or $GRAB_{Ado}$ zebrafish was established and will introduce our recent data about extracellular ATP/Ado dynamic using these zebrafish.

### [3P-120]

### Discovery of a new factor that promotes the formation of translation initiation factor complexes and its role in cancer

*Takuto Itano[1], Rongsheng Huang[2,3], Eiji Nakata[1], Haruyoshi Katayama[1], Toshifumi Ozaki[1], Kazuhito Tomizawa[4], Atsushi Fujimura[2] ([1]*Department of Orthopaedic Surgery, Okayama University Gradeate school of Medicine, Dentistry, and Pharmaceutical sciences,* [2]*Department of Cellular Physiology, Okayama University Graduate School of Medicine, Dentistry, and Pharmaceutical Sciences,* [3]*Department of Trauma Orthopedics, The Second Hospital of Dalian Medical University,* [4]*Department of Molecular Physiology, Kumamoto University Faculty of Life Sciences, Kumamoto*)

The formation of the translation initiation factor complex is the first step required for translation of mRNAs with complex 5'UTRs. The translation initiation factor complex eIF4F is thought to be composed of three pairs: eIF4E, which binds to the m7G cap of mRNA, eIF4A, which has RNA helicase activity, and eIF4G, which acts as a scaffolding protein. Various molecular pathways that promote or inhibit the formation of this complex have been identified, including phosphorylation of 4E-BP by mTOR, especially in the field of cancer research. In this study, we newly identified CDKAL1 as a factor that promotes eIF4F complex formation. In rhabdomyosarcoma, a tumor derived from skeletal muscle cells, CDKAL1 was required to maintain stemness. As a molecular mechanism for this, we found that a group of mRNAs under CDKAL1-dependent translational control contain abundant CG-rich 5'UTRs. As one of them, we found SALL2, a core cancer stemness factor, and confirmed that SALL2 actually defines the stemness of rhabdomyosarcoma under CDKAL1-dependent translational control. Intriguingly, although CDKAL1 is classically a tRNA-modifying enzyme, our mechanism is independent of its enzymatic activity. Furthermore, we have found molecular pathways that regulate CDKAL1-dependent eIF4F complex formation, and confirmed that their inhibition actually induces a decrease in translation and cancer stemness of SALL2. Our findings are expected to contribute to the explanation of physiological phenomena related to translational regulation, since the molecular mechanisms involved in eIF4F are not limited to cancer biology.

### [3P-121]

### Global analysis of specific gene expression in Brain of AQP11 null mice

*Yasuko Tanaka Mochizuki[1], Shoichi Fukada, Takumi Abe, Kenichi Ishibashi([1]*Meiji Pharmaceutical University*)

Aquaporin11 (AQP11) is one of aquaglyceroporins which are permeable to water, glycerol and small molecules. The representative expression patterns are kidney, brain, testis and thymus. Especially, AQP11 null mice have characteristics that they are dead from polycystic kidney disease within one month and their phenotype of thymic involution is began from juvenile. The purpose of this study is to elucidate the role of AQP11 in brain. Therefore, microarray analysis was employed to determine alteration of gene expression. Brain samples were isolated from AQP11 null mice and wild type mice. Gene expression was available to calculate quantitatively as the fold change (FC). Database searches were performed using the David Bioinformatics Resources 6.8 (beta). The annotation analysis was performed 1.2 or more and 0.8 or less for each FC. Representative gene was analyzed by RT-qPCR quantitatively. By this analysis, we identified 24 up-regulated genes which were mainly participate in inorganic cation transmembrane transporter activity. Moreover, 48 gene were identified as down-regulated gene which were related to zinc ion binding, transition metal ion binding, cation binding, ion binding and endoplasmic reticulum. RT-qPCR analyses revealed the enhanced expression of Atp2c2 and the diminished expression of AQP6, Pgap2 and Trim12a. These observations suggest that AQP11 null brain was influenced not only the molecules to participated in material transportation, but also the ion transport gene in endoplasmic reticulum. Furthermore, the brain in AQP11 null mice might have the relation to AQP6 interactions.

### [3P-122]

### Identification and elucidation of liver metastasis-associated miRNAs in primary colorectal cancer

*MING XU[1,2], Ryouichi Tsunedomi[1], Michihisa Iida[1], Nakagami Yuki[1,2], Hiroto Matsui[1], Yoshitaro Shindo[1], Yukio Tokumitsu[1], Yusaku Watanabe[1], Shinobu Tomochika[2,1], Shigeru Takeda[1], Tatsuya Ioka[1], Hiroaki Nagano[1] ([1]*Yamaguchi University*, [2]*Shimonoseki City University*)

**Background:** We previously reported that overexpression of miR-221 and miR-222 in the cancer stroma is associated with liver metastasis in colorectal cancer (CRC). Here, we identified the aberrant miRNA expressions in primary CRC tissues which are associated with liver metastasis and inestigated its function. **Method:** The primary CRC specimens was obtained from patients without liver metastasis (stage II) and with liver metastases (stage IV) by Laser-capture microdissection. The miRNAs expression profile was analysis by microarray (n=6). The candidate miRNAs were validated with q-PCR (stage II, n=39; stage IV, n=42). Tissue from liver metastases was also used. Cell migration and invasion of miRNA mimic and inhibitor transfected cells were assessed by wound-healing assay and transwell assay, respectively. **Result:** miR-30b and miR-106b were identified as overexpressed miRNAs in the primary and liver metastases of stage IV CRC. Inhibition of miR-30b or miR-106b reduced cell migration and invasion ability. On the other hand, increased miR-30b or miR-106b levels promoted cell migration and invasion. **Conclusion:** miR-30b and miR-106b may associated with metastasis of CRC.

### [3P-123]

### Investigating the Efficacy of tDCS in a Rat Model of Motor Cortex Infarction Using the PIT Method

*Tatsuro Kumada[1], Saho Morishita[2], Satoshi Tanaka[3] ([1]*Tokoha Univ. Fac. Hlth. Med. Sci.*, [2]*Tokoha Univ. Fac. Prom. Sci.*, [3]*Hamamatsu. Univ. Sch. Med. Dept. Psych.*)

Rehabilitation plays an important role post-cerebral infarction, as aftereffects often persist post-onset and interfere with daily life. It has been suggested that methods such as electrical brain stimulation are effective as rehabilitation intervention methods. In recent years, transcranial direct current stimulation (tDCS), which is applied by placing electrodes on the scalp and applying a weak direct current, has been proposed. tDCS is a simple method of electrical brain stimulation anticipated for application in the treatment of cerebrovascular disorders, psychiatric disorders, and neurological conditions. However, the underlying neurological mechanisms are largely unknown. Therefore, we have investigated on the efficacy and mechanisms of tDCS after cerebral infarction using cerebral infarction model rats. In this study, the effectiveness of tDCS in restoring motor function in a focal motor cortex rat model with photochemically induced thrombosis (PIT) was investigated. Whereas, rats with the PIT showed low severity of impairment in general neurological evaluations, motor evaluations using the beam walk test and three-dimensional motion analysis revealed significant hindlimb movement impairment. When these rats underwent daily electrical stimulation using the tDCS method, there was an improvement in motor function recovery with low-intensity electrical stimulation, suggesting tDCS's effectiveness.

### [3P-124]

### The cause of cytotoxicity in the Vapor phase of heated tobacco products and rolled cigarettes is reactive oxygen species, and this is regulated by CAMKK2

*Kagemichi Nagao[1], Masanari Umemura[1], Yu Iida[1], Shota Takayasu[1], Soichiro Ishikawa[1], Akane Nagasako[1], Rina Nakakaji[1], Takahiro Horinouchi[2], Yoshihiro Ishikawa[1] ([1]*Yokohama City University Graduate School of Medicine, Cardiovascular Research Institute (CVRI),*, [2]*Hokkaido University Graduate School of Medicine, Department of Cellular Pharmacology*)

[Background] In recent years, due to the increasing usage of heated tobacco products (HTPs), there have been concerns about potential health risks. The substance derived from tobacco smoke after removing nicotine and tar is termed the Vapor phase (VP). Toxicity of the VP from traditional tobacco has been highlighted. Although research on HTPs is still limited, similar concerns about their toxicity have been indicated. We investigated whether the VP from HTPs also possesses cytotoxicity with a particular emphasis on reactive oxygen species (ROS) and intracellular $Ca^{2+}$ concentration. [Materials and Methods] HSC-3 cells (human oral cancer cells) were used. Cigarette Smoke Extract (CSE) from traditional tobacco products (1R6F, University of Kentucky) and HTPs (IQOS, Phillip Morris International) had tar and nicotine removed using a Cambridge filter, and was then suspended in a culture medium (Dulbecco's Modified Eagle Medium or Hanks' Balanced Salt Solution) to create the VP extract. We utilized the Health Canada Intense (HCI) protocol for tobacco extraction, achieving 80 puffs/5ml. Cell Counting Kit-8 assay was performed to investigate toxicity. Western blotting was conducted to examine protein changes. We conducted apoptosis analysis using FlowCytoMeter. Changes in ROS due to VP stimulation were measured by Electron Spin Resonance (ESR) and the high-sensitive-DCFH assay. N-acetylcysteine(NAC) was used to remove ROS. $Ca^{2+}$ concentration was measured by Fluo-4 assay. We focused on one of the $Ca^{2+}$-related signals, CAMKK2 ($Ca^{2+}$/calmodulin-dependent protein kinase kinase). CAMKK2 knockdown of HSC-3 cells was established by shRNA lentiviral system with or without VP stimulation. [Result] Similar to traditional cigarettes, the VP of HTPs also exhibited cytotoxicity. For both VPs, we observed an enhancement in late apoptosis and an increase in phosphorylation of p38. VP stimulation significantly enhanced ROS production. When neutralized with NAC, the cytotoxicity from the VP of traditional cigarettes was negated. Interestingly, HTPs stimulation promoted cell proliferation. Knockdown of CAMKK2 in HSC3 cells reduced the cytotoxicity compared to HSC-3 infected by scramble control shRNA. Additionally, NAC negated the production of ROS and the phosphorylation. [Conclusion] The VP of HTPs, similar to traditional cigarettes, exhibits cellular toxicity through CAMKK2, leading to ROS production.

### Poster

### [3P]
### Drug Action, Pharmacology

March 30, 13:00 - 14:20, Poster Room

### [3P-125]

**Network pharmacology prediction and molecular docking-based strategy to discover the potential pharmacological mechanism of Malaysian Tualang Honey (MTH) against atherosclerosis.**

*Ain Nabila Syahira Shamsol Azman[1], Yoke Keong Yong[1] ([1]*Department of Human Anatomy, Faculty of Medicine and Health Sciences, Universiti Putra Malaysia*)

Atherosclerosis, a prevalent cardiovascular disorder characterized by the accumulation of lipids and fibrous components within the arterial wall, is commonly managed through pharmacological interventions. However, the associated adverse effects of such therapies necessitate the exploration of alternative treatment approaches. Malaysian Tualang Honey (MTH) has shown promising cardioprotective effects in previous research, making it a potential candidate for addressing atherosclerosis. However, the main components and potential mechanisms of MTH remain unclear. This study aims to initially clarify the potential mechanism of MTH in the treatment of atherosclerosis based on network pharmacology and molecular docking techniques. The study involved searching and screening for the primary bioactive compounds and their associated protein targets in MTH using TCMSP, Swiss Target Prediction, and SuperPred databases. To identify atherosclerosis-related targets, OMIM, GeneCards and DisGeNET databases were employed. The overlap between the compound and disease targets was determined, and the common targets were used to construct a protein-protein interaction (PPI) network via the STRING database. This comprehensive process led to the identification of the top 10 central genes. Additionally, we conducted Gene Ontology (GO) and KEGG enrichment analyses on these targets. Finally, molecular docking analyses were carried out for the top 10 core target and the bioactive compounds of MTH. Among the 103 compounds investigated in MTH, only six met the specified screening criteria, leading to the identification of 336 potential gene targets. Notably, 238 of these targets were found to overlap with those associated with atherosclerosis. The identified targets were associated with GO related to biological processes associated with inflammation, which are particularly relevant in the context of atherosclerosis. Additionally, these compounds were found to be directly linked to pathways related to lipids and atherosclerosis. The molecular docking studies revealed that the bioactive compounds in MTH could efficiently bind to the binding sites of the top 10 target proteins. Notably, a majority of these active ingredients exhibited a robust binding affinity with the target gene PIK3CA. In conclusion, this study offers valuable insights into how MTH might be beneficial in treating atherosclerosis, indicating its potential as a promising treatment option. However, it is crucial to conduct further in vitro and in vivo studies to validate these findings and enhance our understanding of the therapeutic mechanisms of MTH.

### [3P-126]

**Evaluation of the Analgesic Effect of the Kampo Formula "Yokukansan" through Inhibition of Substance P Secretion in a Rat Model of Hunner-Type Interstitial Cystitis**

*MANA Tsukada[1,2], Takehiko Sambe[1], Tadashi Hisamitsu[2], Masataka Sunagawa[2] ([1]*Showa Univ. Research Administration Center (SURAC)*, [2]*Dept. Physiol., Showa Univ. Grad. Sch. Med.*)

[Purpose] Originally, Kampo formula “Yokusansan (YKS)” was traditionally administered to patients with symptoms of emotional irritability, neurosis, and insomnia and to infants suffering from night crying and convulsions. The crude drug components of YKS , such as Glycyrrhizae radix, Bupleuri radix, Uncariae uncis cum ramulus, and Cnidii rhizome, have demonstrated analgesic properties. YKS is also employed in the treatment of various pain disorders, including fibromyalgia, post-herpetic neuralgia, phantom-limb pain, headache, and trigeminal neuralgia. This study aimed to investigate the analgesic effects of YKS on Hunner-type interstitial cystitis (HIC) and elucidate its underlying mechanisms using an animal model. [Methods] A Hunner-type interstitial cystitis (HIC) rat model was induced by administering a Toll-like receptor-7 agonist (Loxoribine). Eight-week-old female Wistar rats were divided into three groups: Control group, HIC group, and HIC group treated with YKS (YKS+HIC group). Bladder pain was assessed through von Frey tests, which measured escape behavior. Three days after inducing HIC, bladder and spinal cord tissues were extracted, and the expression of Substance P (SP) was analyzed. [Results] The von Frey tests revealed a significant reduction in escape thresholds in the HIC group when compared to the Control group. However, this reduction was further alleviated by YKS treatment. Additionally, the examination of SP expression in the bladder wall and spinal cord showed a noteworthy increase in the HIC group compared to the Control group, yet this increase was further mitigated by YKS treatment. [Discussion] It has been reported that SP in bladder tissue is involved in the expression of bladder pain through neurokinin-1 receptors. Kampo formulas are composed of multiple crude drugs, suggesting that multiple mechanisms of action work concurrently to produce an effect. We have previously reported the contribution of antioxidant effects as one of the mechanisms of YKS. YKS proves to be beneficial in alleviating HIC pain, and one of its mechanisms of action is likely the suppression of SP secretion. Although YKS treatment for HIC is not yet widespread, it holds promise for future clinical applications. COI:NO

### [3P-127]

**The effect of the ketogenic diet on inflammatory pain and neuropathic pain**

*Kei Eto[1], Masanori Ogata[1], Hitoshi Ishibashi[1] ([1]*Department of Physiology, School of Allied Health Sciences, Kitasato University*)

Chronic pain is pain that lasts for a long time, and there are two types of it: chronic inflammatory pain and chronic neuropathic pain. Because inflammation is involved in the induction of both types of chronic pain, inhibition of inflammation can be a good target for the treatment of chronic pain. The ketogenic diet, which contains high fat, low carbohydrate, and adequate protein, is used for the treatment of epilepsy. Since the treatment of the ketogenic diet generates ketone bodies, which have an antioxidant effect, the ketogenic diet can attenuate inflammation. Thus, the ketogenic diet inhibits not only epilepsy but also other diseases, such as Parkinson’s disease. In addition, a previous report demonstrated that the ketogenic diet alleviated complete Freund adjuvant-induced inflammatory pain. However, little is known about whether this diet inhibits chronic inflammatory pain induced by other inflammatory compounds and chronic neuropathic pain. Thus, in the present study, we examined the effect of the ketogenic diet on formalin-induced chronic inflammatory pain and sciatic nerve injury-induced chronic neuropathic pain. The injection of formalin into the dorsal surface of the hind paw induced mechanical allodynia in both hind paws ipsilateral and contralateral to the formalin injection one week after the injection. Treatment of the ketogenic diet alleviated the mechanical allodynia in both hind paws. Next, we examined the effect of the ketogenic diet on paw edema induced by formalin injection. Formalin injection induced edema on the hind paw, while the ketogenic diet inhibited the edema. Furthermore, we performed immunohistochemical staining of microglia in the spinal dorsal horn and examined whether the ketogenic diet can inhibit formalin-induced microglia activation in the spinal cord. We found that formalin injection into the mouse hind paw increased the number of microglia in the spinal dorsal horn ipsilateral to the injected hind paw one week after the injection. On the other hand, ketogenic diet treatment significantly reduced the number of microglia, but the number did not return to the baseline level. Finally, we examined the effect of the ketogenic diet on mechanical allodynia induced by partial sciatic nerve ligation. Nerve injury-induced mechanical allodynia and ketogenic diet treatment alleviated the allodynia. Thus, these results suggest that the ketogenic diet may reduce formalin-induced inflammation and inhibit microglia activation in the spinal dorsal horn, which in turn alleviates mechanical allodynia. Since inflammation is critical for the induction of neuropathic chronic pain, this anti-inflammatory effect of ketogenic may contribute to attenuation of mechanical allodynia induced by nerve injury.

### [3P-128]

### Subunit-specific inhibition of GIRK channel activity by a steroid alkaloid conessine and its region of action

*Misaki Yoshimasu[1], Ayaki Tsuda[1], I-Shan Chen[1], Tomoe Y. Nakamura-Nishitani[1]
([1]*Wakayama Medical University*)

### Poster

### [3P]
### Medical education, Medical histology

March 30, 13:00 - 14:20, Poster Room

### [3P-129]
### Conditions for proper textbooks of physiology for students who wish to be clinical engineers.

*Takayoshi Hosono[1] ([1]*Osaka Electro-Communication University*)

Clinical engineer is one of co-medical professions who operate life support management equipment such as respirators, heart-lung machines, and dialyzer. The national qualification examination for clinical engineer consists of 180 questions, and about 60 questions of the exam relates to physiology and medicine, and the rest problems relate to engineering, physics, chemistry and information science. Therefore students needs compact and easy to understand. In present study, we prepared 12 textbooks which seemed suitable for students of students at early stages. We made photocopies consisted of 10 – 12 pages of the texts, and let read the photocopies for 20 students. After reading the photocopies, the students estimated each textbook for scores between 0 to 10 on the viwe points of (1) easy to understand, (2)suitable for preparation, (3)easy to review, (4) easy to read, (5) simple figures, (6) easy explanation, (7) satisfied with contents, (8) appropriate quantity, (9) suitable for preparation and measure for national examinations by 5 levels. The averaged scores distributed between 3.6 and 2.5. In free descriptions needs of exercise problems were frequently suggested. Conclusion. Students' estimations of textbooks of physiology widely distributed.

### Poster

### [3P]
### Study Methodology

March 30, 13:00 - 14:20, Poster Room

### [3P-130]

### Quick and easy method for gramicidin-perforated patch-clamp recordings.

*Kakeru Miura[1], Sota Ueda[1], Kosuke Kazashi[2], Kazuhito Utsunomiya[1], Mari Kiriyama[2], Toshihide Tabata[3] (*[1]Intellectual Information Engineering, Department of Engineering, University of Toyama, [2]Medical Design Program, Graduate School of Pharma-Medical Sciences, University of Toyama, [3]Faculty of Engineering, University ofToyama*)

A perforated patch-clamp technique with the chloride ion-impermeable perforating agent gramicidin (gPPCT) is indispensable for investigating the electrical activities of excitable cells highly dependent on the chloride ion gradient. The major applications of a gPPCT include the analysis of the excitability of the central neurons involved in circadian rhythm regulation. Despite its importance, a gPPCT has been rarely used because gramicidin leaked from the recording pipette often hampers giga-seal formation, and perforation progresses only slowly. Here we devised a modified gPPCT in which a high concentration (250 µg/ml) of gramicidin was delivered to the patched membrane after the completion of giga-seal formation. Gramicidin was puffed from the fine quartz capillary (outer diameter, 100 µm; ALA QT, ALA Scientific Instruments, NY, USA) that was inserted in a recording pipette (inner diameter, 0.86 mm; outer diameter, 1.5 mm) through the 30° fork of a 2PK+ pipette holder (ALA Scientific Instruments). We examined the efficacy of the modified gPPCT using cultured HEK293 cells as a model preparation. When the modified gPPCT was combined with narrow-orifice recording pipettes typically used in brain slice recordings (tip resistance, ~8.5 MΩ), the success rate of giga-seal formation (~80%) and the perforation speed (time for the series resistance to decrease to 200 MΩ, ~19 min; the series resistance achieved 20 min after gramicidin delivery, ~200 MΩ) were improved as compared with those of a conventional gPPCT (~50%, ~37 min, and ~360 MΩ) in which the tip and rest of the recording pipette were filled with gramicidin-free and -containing pipette solutions, respectively. When the modified gPPCT was combined with wide-orifice pipettes (~2.5 MΩ), the perforation speed was further improved (~18 min and ~150 MΩ). Moreover, we could use the modified gPPCT to record the potential and current responses from mammalian central neurons. Our modification would allow many researchers to perform gPPCT recordings with ease and thereby promote the investigations of chloride ion gradient-dependent cellular activities.

### [3P-131]

### Methods to selectively activate neurons and astrocytes in a calcium imaging study

*Hiroshi Onimaru[1], Isato Fukushi[2], Shunya Yoda[1], Keiko Ikeda[3], Itaru Yazawa[4], Kotaro Takeda[5], Yasumasa Okada[6], Masahiko Izumizaki[1] (*[1]Department of Physiology, Showa University School of Medicine, [2]Faculty of Health Sci., Aomori Univ. of Health and Welfare, Aomori, Japan, [3]Dept. Oral Physiol, Showa Univ. Sch. of Dent., Tokyo, Japan, [4]Dept. Food & Nutrition, Kyushu Nutrition Welfare Univ, Fukuoka, Japan, [5]School of Health Sciences, Fujita Health Univ., Toyoake, Aichi, Japan, [6]Clinical Res. Ctr., Murayama Med. Ctr., Musashimurayama, Tokyo Japan*)

In calcium imaging experiments, it is essential to classify cells displaying increased calcium signals as either neurons or astrocytes. We aimed to develop methods to differentiate neurons and astrocytes by specifically activating each of them in a calcium imaging study. Experiments were performed in brainstem-spinal cord preparations that were isolated from newborn rats (P0-P4) under deep isoflurane anesthesia. Preparations were transversely cut at the rostral level of the medulla, and superfused with the artificial cerebrospinal fluid (25-26˚C). The C4 ventral root activity was recorded to assess the inspiratory output of the preparation. The cellular activities in the ventrolateral medulla close to the cut surface where a calcium indicator Oregon Green was locally injected were analyzed by calcium imaging. We confirmed that the application of low K+ (0.2 mM) or proteinase-activated receptor-1 (PAR1) agonist TFLLR (10 µM) could predominantly activate astrocytes in the medulla. In addition, immunohistochemical examination demonstrated that PAR1 was expressed on many astrocytes in the ventral medulla. To develop effective methods for selective activation of neurons, we tested several compounds that were expected to specifically excite neurons, e.g. glutamic acid (100-400 µM), excitatory peptides such as substance P (50-100 nM) and voltage dependent Na+ channel opener, aconitine (5 µM). We found that aconitine and substance P were useful for the identification of neurons, although a limited number of astrocytes might also be activated. We propose that the combination of aconitine (or substance P) and low K+ (or TFLLR) stimulation would be helpful for the classification of neurons and astrocytes in a calcium imaging study.

### [3P-132]

### Improved gradual acceptor photobleaching reveals the interplay of distinct GPCRs in living cells.

*Takuya Mori[1], Taito Takahashi[1], Hakushun Sakairi[2], Yuji Kamikubo[2], Takashi Sakurai[2], Toshihide Tabata[3] (*[1]Graduate School of Pharma-Medical Sciences, University of Toyama, [2]Department of Pharmacology, Juntendo University School of Medicine, [3]Faculty of Engineering, University of Toyama*)

We have previously shown that B-type gamma-aminobutyric acid receptor (GABAbR) and type-1 metabotropic glutamate receptor (mGluR1) interplay with each other on the postsynaptic membrane of cerebellar Purkinje neurons, and this may facilitate mGluR1-mediated synaptic plasticity crucial for cerebellar motor learning. To elucidate the extent of the interplay between these G protein-coupled receptors, we devised an improved gradual acceptor photobleaching. Cultured HEK293 cells co-expressing GABAbR1 subunit with a Halo-tagged extracellular domain, GABAbR2 subunit, and mGluR1 subunit with a SNAP-tagged extracellular domain were alternatively illuminated with a weak 488 nm laser and an intense 552 nm laser using a confocal microscope. The time-course of donor fluorescence of each fluorescent punctum was fitted by a model equation incorporating an exponential rise reflecting the release of the donor fluorophore (HaloTag Alexa Fluor 488 Ligand, Promega) from donor-acceptor Förster resonance energy transfer (FRET), and an exponential decay starting after a short pause reflecting the fading of donor fluorescence following a donor-donor trap. When the mGluR1 subunit was solely labeled with the donor and acceptor fluorophores (SNAP-Surface 488 and SNAP-Surface 594, respectively, New England BioLabs) (i.e., a fraction of mGluR1 was expected to contain both donor and acceptor fluorophore-labeled subunits), the initial FRET efficiency ($E_0$) was 0.493 (median) and an application of 10 µM L-glutamate decreased the $E_0$ to 0.187. This result is consistent with the previous report that ligand-binding increases the distance between the extracellular domains of the subunits constituting a functional mGluR1. When the GABAbR1 subunit was solely labeled with the donor fluorophore (theoretically, $E_0 = 0$), the $E_0$ was as low as 0.025. When the GABAbR1 and mGluR1 subunits were labeled with the donor and acceptor fluorophores, respectively, the $E_0$ reached 0.489. This result demonstrates the intrinsic property of GABAbR to interplay with mGluR1. Our method would provide a powerful tool for quantitative analysis of inter-GPCR interplay in living cell preparations.

### Poster

### [3P]
### Others

March 30, 13:00 - 14:20, Poster Room

### [3P-133]

### Suppressive Activity of Boiogito, a Japanese Traditional Kampo Medicine, on Periostin Secretion in Human Fibroblast-like Synoviocytes *In Vitro*.

*Takayuki Okumo[1,3], Midori Mochizuki[2,4], Haruka Takemura[2,3], Kanako Izukashi[2,3], Hideshi Ikemoto[1], Naoki Adachi[1], Yasunori Takayama[1], Nobuyuki Kawate[4], Koji Kanzaki[3], Masataka Sunagawa[1] ([1]*Department of Physiology, Showa University School of Medicine, Tokyo, Japan.*, [2]*Department of Physioilogy, Showa University Graduate School of medicine, Tokyo, Japan.*, [3]*Department of Orthopedic Surgery, Showa University Fujigaoka Hospital, Yokohama, Japan.*, [4]*Department of Rehabilitation Medicine, Showa University School of Medicine, Tokyo, Japan.*)

Background: Osteoarthritis (OA) is a degenerative joint disease mainly affecting the elderly and characterized by symptoms such as pain, swelling, limited range of motion, and locomotive dysfunction. Our previous report has revealed that synovial fluid aspirated from knee OA patients has higher levels of periostin depending on disease progression, which suggests that periostin is one of the important biomarkers associated with development and progression of OA. The present *in vitro* study was investigated whether periostin production and secretion from interleukin-13 (IL-13)-stimulated human fibroblast-like synoviocyte (hFLS) could be inhibited by administration of boiogito (BOT), a kampo medicine.
Methods: Initially, the dose- and time-dependent manner of periostin production in hFLS stimulated by IL-13 was assessed by ELISA. Following these preliminary experiments, it was planned to stimulate hFLS with IL-13 at a concentration of 20 ng/ml for 72 hours. Subsequently, BOT was applied 2 hours prior to IL-13 stimulation at a concentration of 100, 500, 1000 ug/ml, respectively. After 72 hours, Cell proliferation was assessed by MTT assay and cell culture medium was evaluated for periostin levels with ELISA.
Results: IL-13 stimulation led to increased cell proliferation in hFLS and preapplication of BOT made no difference in MTT assay. Notably, periostin levels were significantly higher in the IL-13 group compared to the control group, which was significantly reduced in the IL-13 + BOT 1000 ug/ml group.
Discussion: Our previous *in vivo* study demonstrated that oral administration of BOT can prevents the progression of OA in a knee OA rat model. This study suggests that one of the therapeutic mechanisms of BOT in treating OA may involve the inhibition of periostin secretion within the inflamed synovium. However, the specific signaling pathway through which BOT inhibits periostin production remains unclear and further research is needed to identify the essential components in BOT responsible for these effects.

### [3P-134]

### The involvement of acetylcholine in the initial phases of sexual reproduction in unicellular organisms

Kaho Komatsu[1], Yuto Shimada[2], Yuya Hasegawa[1], Rikiya Nakamura[1], *Mikihiko Arikawa[1] ([1]*Kochi Univ.*, [2]*National Institutes of Natural Sciences*)

Ciliated protozoa exhibit an intriguing process known as conjugation for sexual reproduction. It has been shown that an extracellular factor is involved in cell-cell interactions during the initial phases of conjugation in *Tetrahymena thermophila*. However, this factor remains unidentified. Actually, when cells were washed during the conjugation induction, the number of paired cells was significantly decreased. This result indicates that the extracellular factor involved in the pair formation exists in extracellular liquid of the conjugation-induced cells. In the present study, building on prior experiments conducted on the ciliate *Paramecium caudatum*, we investigated the effect of acetylcholine (ACh), a widely recognized neurotransmitter in the nervous systems of higher organisms, on pair formation during *Tetrahymena* conjugation. When carbamylcholine, a stable analogue of ACh, or tacrine, an inhibitor of ACh-degrading enzymes, was added to *Tetrahymena* cells during conjugation induction, the pair formation was accelerated. Conversely, when acetylcholinesterase, an ACh-degrading enzyme, or atropine, an inhibitor of muscarinic ACh receptors, was added, the pair formation was attenuated. On the other hand, tubocurarine, an inhibitor of nicotinic ACh receptors, did not show any noticeable effects. These results indicate that 1) ACh is secreted from the conjugation-induced cells, 2) ACh is rapidly degraded by acetylcholinesterase, 3) ACh binds to not nicotinic but muscarinic ACh receptors, and 4) ACh somehow promotes pair formation. On the basis of these results, ACh can be considered as the extracellular factor involved in cell-cell interactions in *Tetrahymena* conjugation. In the conjugation process, it is thought that *Tetrahymena* cells secrete ACh, and cells that receive it through muscarinic ACh receptors are more likely to form pairs. In other words, it is likely that cells actively promote pairing on their own via secretion and reception of ACh. Although the mechanism behind this self-promotion system in the sexual reproduction in *Tetrahymena* is not yet understood, ACh may play a pivotal role in cell-cell communication in ciliated protozoa. Our study contributes to the growing understanding of this fascinating biological phenomenon.

### [3P-135]

### Therapeutic Effects of Platelet-Rich Plasma on Knee Osteoarthritis Progression in Rats

*Haruka Takemura[1], Takayuki Okumo[2], Taro Kimura[2,3], Kanako Izukasi[1], Midori Mochizuki[2], Hideshi Ikemoto[2], Yasunori Takayama[2], Naoki Adachi[2], Koji Kanzaki[3], Masataka Sunagawa[2] ([1]*Department of Bioregulation, Showa University Graduate School of Medicine*, [2]*Department of Physiology, Showa University School of Medicine, Division of Bioregulation*, [3]*Department of Orthopedic Surgery, Showa University Fujigaoka Hospital*)

Background: In recent years, intra-articular administration of Platelet-rich Plasma (PRP) has garnered attention as a novel therapeutic strategy for knee osteoarthritis (KOA). However, whether PRP therapy suppresses the degenerative changes in the joint remains uncertain. In this study, PRP was intra-articularly injected to rats with induced KOA, and its therapeutic effects were investigated.
Methods: PRP was prepared from the whole blood of nine-week-old male Wistar rats by centrifugation at 25°C, 200×g, for seven minutes.We conducted a previous study to determine the method of adjusting PRP.We defined good PRP as a sample with a low erythrocyte count and a high platelet count. 8 ml of whole blood was aspirated from five 9-week-old male Wistar rats and centrifuged at 25°C, 200 × g, for 7 minutes to prepare PRP. The white blood cell count, red blood cell count, and platelet count were measured, and the differences in blood cell components between whole blood and PRP were compared.The white blood cell count tended to be higher in whole blood than in PRP, but the difference was not significant. The red blood cell count was significantly lower and the platelet count was significantly higher in PRP than in whole blood .
KOA was induced in the right knees of 9-week-old male Wistar rats through medial meniscus destabilization (DMM) surgery. The animals were divided into four groups: control, sham, DMM, and DMM + PRP (n = 5 each). Rats in the DMM + PRP group received 50 µl of intra-articular PRP injection in the right knee joint at 4 weeks post-surgery. During the study, periodic rotarod test was conducted to assess locomotive function. Eight weeks after DMM surgery, the degree of medial meniscus extrusion was measured by CT imaging in the right knee, followed by histological analysis of the harvested knees. The progression of KOA was assessed using OARSI score, and the number of multi-nucleolus TRAP-positive osteoclasts in the subchondral bone was counted in the histological analysis.
Results: No significant differences were observed in the degree of medial meniscus extrusion between the DMM group and the DMM + PRP group. Likewise, there were no significant differences in the walking time in the rotarod test between the DMM group and the DMM + PRP group. However, OARSI score was significantly higher in the DMM group, which was significantly in the DMM + PRP group. TRAP-positive osteoclasts in the subchondral bone in the DMM group increased over time, peaking at 4 weeks post-surgery. While the DMM + PRP group showed higher TRAP-positive osteoclasts in the subchondral bone compared to the Control group, no significant difference was found compared to the DMM group.
Conclusion: Intra-articular administration of PRP in KOA model rats suggests the potential to inhibit KOA progression.

### [3P-136]

### The cytoskeletal remodeling is significantly inhibited by substance derived from Chinese herbal medicine, demonstrating its potent tumor-inhibiting effect

*Haibo Wang[1], Zewen Chu[1,2], Shiyu Guo[2], Masataka Sunagawa[2], Yanqing Liu[1,2]
([1]*Yangzhou University,* [2]*Showa University*)

**Aims of this study:** This study was to explore the effect and mechanism of diterpene compound Triptonoterpene from *C. orbiculatus* on apoptosis of GC cells, to provide experimental basis for clarifying the anti-tumor components of *C. orbiculatus* and lay the foundation for the development and application of *C. orbiculatus*.**Methods:** BGC-823 cells and MKN-28 cells will be treated with Triptonoterpene. We first detect cell proliferation through cell viability detection assay and colony formation assay. Anexin V-FITC staining assay, Hoechst staining and Mitochondrial transmembrane potential test were used to observe the apoptosis of cells. The distribution of F-actin can be observed using TRITC-Phalloidin staining. Western blot assay was used to detect the expression of apoptosis related proteins and actin cytoskeleton related proteins. **Results:** After Triptonoterpene intervention in GC cells, we observed apoptosis events in the cells. After TRITC-Phalloidin staining, it was observed that F-actin gathered around the nucleus under fluorescence microscope, and actin remodeling was inhibited. The ROCK/LIMK/Cofilin pathway in GC cells is also blocked by Triptonoterpene, which destroys the dynamics of actin. We further explored the relationship between the dynamic changes of actin and apoptosis of GC cells under the influence of Triptonoterpene. We suspect that this event may be due to the imbalance of actin dynamics, which leads to the mitochondrial membrane permeabilization (MMP) event and cell apoptosis. As we can see, in response to Triptonoterpene treatment, the mitochondrial transmembrane potential ($\Delta\Psi m$) of GC cells is significantly reduced through fluorescence microscopy, and the expression of Bcl-2 family related proteins has undergone significant changes. When we pretreated with Latrunculin A (LAT-A, an inhibitor of actin polymerization), we can see that under the combined action of Triptonoterpene and LAT-A, the $\Delta\Psi m$ is lower. which may affect actin dynamics through ROCK/LIMK/Cofilin pathway, leading to the occurrence of MMP events and eventually apoptosis. **Conclusions:** Triptonoterpene induces apoptosis of GC cells by inhibiting actin remodeling, it may be that the change of actin dynamics causes the change of MMP.

### [3P-137]

### Electroacupuncture effects on motor coordination and ADAMTS5 expression in a rat model of knee osteoarthritis

*Oyunchimeg Chuluunbat[1], Hideshi Ikemoto[1], Naoki Adachi[1], Takayuki Okumo[1], Zewen Chu[2], Shiyu Guo[1], Yangqing Liu[2], Tadashi Hisamitsu[1], Sunagawa Masataka[1]
([1]*Department of Physiology, Showa University Graduate School of Medicine,* [2]*Institute of Traditional Medicine, Medical College, Yangzhou University*)

Knee osteoarthritis (KOA) is an age-associated disorder that leads to the weakening of articular cartilage, affecting the quality of life due to symptoms such as pain and limited range of motion. Electroacupuncture (EA) therapy is a therapeutic approach in which a mild electric current is administered through the acupuncture needles at acupoints. EA has shown to have potential benefit for KOA; however, its effect on articular cartilage remains unclear. In this study, we investigated the effects of EA on the expression of a disintegrin and metalloproteinase with thrombospondin motifs subtype 5 (ADAMTS5), a key enzyme associated with cartilage degradation, in a KOA model rat. Wistar rats were divided into four groups: a control, a Sham, an OA induced by surgically destabilizing the medial meniscus (DMM), and an EA-treated after OA induction (DMM+EA) groups, respectively. DMM surgery was performed on the right knee. EA treatment (square-wave pulse current, 15 mA, 4 Hz) was administered by passing between ST36 (Zusanli) and Ex-LE2 (Heiding) acupoints, three times a week, for 30 minutes each time, for four weeks after DMM operation. The rotarod test was carried out before surgery and on days 7, 14, 21, and 28 after surgery to measure a loss of motor coordination. In addition, histological assessment was performed to evaluate degenerative changes in articular cartilage, and western blot analysis was conducted to investigate the expression of ADAMTS5 in the right knee joint tissue four weeks after the surgery. Rats in the DMM group exhibited a marked decrease in the time spent on the rod on days 14, 21, and 28, compared to the control group; however, the reduction was significantly inhibited in the DMM+EA group. The histological assessment revealed the degeneration of knee articular cartilage in the DMM group, which was less severe in the DMM+EA group. DMM treatment increased the expression of ADAMTS5 in knee joint tissue, which was reduced by EA treatment. These results indicate that EA's ability to suppress the expression of ADAMTS5 in knee joint tissue plays a role in improving the activity limitation induced by OA.

### [3P-138]

### New concept for the creation of organic compounds that enable life form on primitive Earth

*Yuki Tado[1], Takuro Fujiwara[1], Yuki Okubo, Nobuaki Takahashi[1] ([1]*Kyoto University*)

The Earth is a watery place and the sole planet where life has been confirmed. While the presence of water is in general believed to be essential in the establishment of life, a large quantity of $CO_2$ was also necessary for the birth and flourishing of life on Earth. The existence of both $CO_2$ and water led to the formation of organic compounds that constitute living organisms. However, it remains elusive how organic compounds were generated from inorganic $CO_2$ on primitive Earth. In particular, most previous studies have focused on $CO_2$ fixation chemistry utilizing $H_2$ as a stoichiometric reductant under extremely high pressures and temperatures, and the relevance of the reaction products to biology is basically low. Here we show that ultraviolet (UV) photoredox chemistry between $CO_2$ and compound X, a non-metallic substance found in a certain type of meteorite, generates a wide variety of organic compounds, such as acetate, lactate, succinate, and malic acid. On primitive Earth, shorter wavelengths of UV (> 200 nm) reached the Earth's surface without being blocked by ozone layer. Given this, together with the fact that $CO_2$ was the major component of antient atmosphere, it is plausible that the combination of UV and $CO_2$ was responsible for the creation of organic organisms on primitive Earth. We are currently investigating whether some archaea and bacteria can survive and proliferate in a glucose-free minimal media supplemented with the products generated by our UV chemistry as carbon sources.

### [3P-139]

### Study on the mechanism of Traditional Chinese medicine *Celastrus orbiculatus* extract alternative splicing ACTN4 by PTBP1 to inhibit gastric cancer metastasis

*Zewen Chu[1,2], Haibo Wang[1], Shiyu Guo[2], Masataka Sunagawa[2], Yanqing Liu[1,2]
([1]*Yangzhou University,* [2]*Showa University*)

Actin cytoskeleton remodeling is not only the structural basis of tumor cell morphology change and invasion and metastasis movement, but also the power source of cell movement, which is an important biological process of tumor cell invasion and metastasis. According to the theory of Traditional Chinese medicine, deficiency of vital qi is the root of gastric cancer, and stasis of collaterals is the key to gastric cancer. Therefore, rattan drugs are often used clinically to prevent shape, promoting blood circulation for removing obstruction in collaterals, use rattan drugs to activating collaterals to treat the syndrome of collateral stasis. It was found that Traditional Chinese medicine *Celastrus orbiculatus* extract could inhibit the invasion and metastasis of gastric cancer by inhibiting the actin remodeling of gastric cancer cells. Proteomic studies have confirmed that polypyrimidine region binding protein 1 (PTBP1) is involved in *Celastrus orbiculatus* extract to inhibit actin cytoskeleton remodeling in gastric cancer cells, but the molecular mechanism remains unclear. This project intends to study the molecular mechanism of *Celastrus orbiculatus* extract inhibiting cytoskeletal remodeling and invasion and metastasis of gastric cancer by PTBP1 mediated alternative splicing based on the pathogenesis characteristics of "stagnation to phlegm stasis obstruct the collaterals" of gastric cancer by cell experiments and the construction of PTBP1 Cas9-KO mouse model. To clarify the relevant mechanism of inhibiting invasion and metastasis of gastric cancer by *Celastrus orbiculatus* extract, and to lay theoretical and experimental basis for the clinical application and development of *Celastrus orbiculatus* Thunb. as anti-cancer drugs.

### Late Breaking Abstracts

Day 1
(March 28, 13:00 - 14:20)

[1LBA] Autonomic nervous system
[1LBA] Environmental physiology
[1LBA] Nutritional and metabolic physiology, Thermoregulation
[1LBA] Behavior, Biological rhythm, Sleep
[1LBA] Study Methodology
[1LBA] Others

### Late Breaking Abstracts

### [1LBA]
### Autonomic nervous system

March 28, 13:00 - 14:20, Poster Room

### [1LBA-001]

### Heart rate response to autonomic nervous system blockers in Japanese house bats

*Kazuki YOSHINO-HASHIZAWA[1], Kohta I KOBAYASI[1], Shizuko HIRYU[1] ([1]*Doshisha Univ.*)

Bats are fascinating animals for cardiac control mechanisms because they are the only mammals capable of flight, hibernation, and daily torpor. Bats are also great model animals for vocal communication because they rely on vocalizations to make decisions even in the dark. However, there is currently no established method to assess their anxiety and stress levels associated with their behavior. Therefore, there is a need to develop an assessment system for autonomic nervous system (ANS) activity. In this study, we administered ANS blockers (atropine and propranolol) to Japanese house bats (*Pipistrellus abramus*) and analyzed their heart rate (HR) response to the dose. The results showed that HR tended to decrease after administration of a low dose of atropine and PBS as a control, whereas a high level of HR (approximately 400 bpm) was maintained after administration of a high dose of atropine. In addition, high doses of propranolol decreased HR. These results show that atropine and propranolol act on their ANS and have dose-dependent effects on bats. Furthermore, these blockers have the potential to evaluate the internal states of bats through intervention in behavioral experiments.

### [1LBA-002]

### Responses of splenic sympathetic nerve activities induced by electrical stimulation of the sciatic nerve

*Masamichi Moriya[1], Nobuhiro Watanabe[1], Harumi Hotta[1] ([1]*Tokyo Metropolitan Institute for Geriatrics and Gerontology*)

Exercise is known to modulate immune functions possibly through the autonomic and endocrine systems. This study investigated whether the nerve stimulation-induced contraction of hindlimb muscles affects the splenic sympathetic nerve efferent activity. In male rats anesthetized with isoflurane, splenic sympathetic nerve activity was recorded while stimulating the intact sciatic nerve with a pulse of 0.2 ms duration at 2 or 4 times the motor threshold to induce muscle contraction. Train stimuli (10 pulses at 100 Hz) was applied to produce tetanic contraction, and was repeated 30 times (10 trains at 1 Hz was repeated 3 times at an interval of 1 minute). For analysis, the reflex potentials associated with the train stimuli were averaged for the 30 times. An excitatory potential at a latency of approx. 80 ms was consistently observed. To eliminate the influence of muscle contractions, we either cutting the sciatic nerve at a site peripherally to the stimulation, or administering a muscle relaxant rocuronium. However, the magnitude of the reflex potential was not altered by the presence or absence of the muscle contractions. The results indicate that afferent stimulation of the sciatic nerve excite splenic sympathetic nerve, independently of muscle contractions.

### Late Breaking Abstracts

[1LBA]

**Environmental physiology**

March 28, 13:00 - 14:20, Poster Room

**[1LBA-003]**

### Elucidation of the Regulatory Mechanisms of *Fgf21* Expression by Ethanol

*Hazuki Ioroi[1], Sho Matsui[1], Yasuo Oguri[1], Satoshi Tsuzuki[1], Tsutomu Sasaki[1]
([1]*Department of Food Science and Biotechnology, Graduate School of Agriculture, Kyoto University*)

Fibroblast growth factor 21 (FGF21) is a peptide hormone predominantly produced by the liver, which is induced by nutritional signals such as fasting, high-carbohydrate diets, and amino acid restriction. It has been reported that ethanol consumption increases circulating FGF21 levels and hepatic expression of *Fgf21* mRNA in mice. While various transcriptional factors have been identified in the regulation of *Fgf21* expression, the precise mechanisms by which ethanol controls *Fgf21* expression remains unknown. In this study, we investigated the regulatory mechanisms of *Fgf21* expression by ethanol.
To investigate what ethanol metabolite is responsible for induction of *Fgf21*, AML12 cells, murine hepatocyte cell line, were co-treated with 200 mM ethanol and 2 mM pyrazole, an inhibitor of alcohol dehydrogenase. The *Fgf21* mRNA levels and the concentration of acetaldehyde in the medium were measured by quantitative PCR and acetaldehyde assay kit, respectively. We found that *Fgf21* mRNA levels were significantly increased by ethanol treatment, even when production of acetaldehyde from ethanol was blocked. Furthermore, to investigate whether ethanol cause changes in signal transduction pathway, AML12 cells were treated with 200 mM ethanol and 2 mM pyrazole, and the phosphorylation of Akt, JNK, ERK, and p38 MAPK were measured by western blotting. We found that phosphorylation of ERK was reduced under co-treatment with ethanol and pyrazole. Moreover, *Fgf21* mRNA levels were increased under treatment with 10 µM U0126, a MEK inhibitor.
These data indicate that ethanol, not acetaldehyde, is responsible for *Fgf21* induction and that the pathway is mediated through reduction of ERK phosphorylation.
It is still unclear whether *Fgf21* induction by ethanol is caused by transcriptional activation or increased stability of mRNA. Now we are trying to investigate the effect of ethanol on the stability of *Fgf21* mRNA. We treated AML12 cells with ethanol and actinomycin D (ActD), an inhibitor of transcription, and measured *Fgf21* mRNA levels by quantitative PCR. We found that *Fgf21* mRNA levels tended to be higher when co-treated with ethanol and ActD than when treated with ActD alone, indicating that ethanol might inhibit *Fgf21* mRNA decay. We will investigate the relationship between mRNA stability and ERK phosphorylation and clarify the mechanisms of *Fgf21* induction by ethanol in more detail.

### Late Breaking Abstracts

### [1LBA]
### Nutritional and metabolic physiology, Thermoregulation

March 28, 13:00 - 14:20, Poster Room

### [1LBA-004]
### Regulation of BHMT by LRH-1 in methionine cycle of liver

*Sulagna Mukherjee[1], Jae-Ho Lee[1], Hee-Kyung Han[1], Soo-Young Park[1], Hye Ji Jang[1], Dae-Kyu Song[1], Jae-Hoon Bae[1], Seung-Soon Im[1] ([1]*Keimyung University*)

Betaine-homocysteine S-methyltransferase (BHMT), one of the most abundant proteins in the liver, is involved in the regulation of homocysteine metabolism. Generally, decreased BHMT gene expression leads to homocysteine accumulation in the liver, which can induce mitochondrial stress. However, the molecular mechanism of *Bhmt* transcription has not been elucidated. This study outlines the mechanism of *Bhmt* is mediated by liver receptor homolog-1 (LRH-1), and the effect of BHMT deficiency in liver causes methionine disorder. During fasting, both *Bhmt* and *Lrh-1* expressions increased in the liver of normal mice, but *Bhmt* expression was decreased in LRH-1 liver specific knockout (LKO) mice. In addition, the lipid peroxide content in the liver tissues of LRH-1 LKO mice was increased. Promoter activity analysis confirmed the binding of LRH-1 to a specific site at +131/+137 bp of the mouse *Bhmt* promoter. LRH-1 deficiency is associated with elevated reactive oxygen species (ROS) production, lipid peroxidation, and mitochondrial stress in hepatocytes. In conclusion, this study suggests that lack of LRH-1-mediated decrease in *Bhmt* expression promotes triglyceride accumulation by increasing ROS levels and inducing mitochondrial stress via disrupted methionine cycle. Understanding these regulatory pathways may pave the way for novel therapeutic interventions in metabolic disorders associated with hepatic lipid accumulation.This work was supported by the Ministry of Education of the Republic of Korea and the National Research Foundation of Korea(NRF-2023R1A2C3003717) and Main Research Program of the Korea Food Research Institute funded by the Ministry of Science and ICT and the Ministry of Health and Welfare, Republic of Korea(HI14C1324).

### [1LBA-005]
### Loss of SREBP-1c Ameliorates Hepatic Steatosis and Liver injury in NASH through Lipocalin-2

Eun-Ho Lee[1], *Min-Hee Seo[1], Dae-Kyu Song[1], Jae-Hoon Bae[1], Seung-Soon Im[1] ([1]*Keimyung University*)

Roles of sterol regulatory element-binding proteins (SREBPs) have been established as lipid synthetic transcription factors especially for cholesterol and fatty acid synthesis. SREBP-1c isoform, which constitutes more than 90% of the in vivo SREBP-1, is a key regulator of early events in the liver's response to insulin and is a major determinant of lipogenic gene transcription. In this study, we explored the role of SREBP-1c on NASH and LCN2 gene expression regulation. Wild-type and SREBP-1c knockout (KO) mice fed with a high-fat/high-sucrose diet, carbon tetrachloride ($CCl_4$)-treated, and with lipocalin-2 (LCN2) overexpression. LCN2 gene expression and secretion increased in $CCl_4$-induced liver fibrosis mice models, and SREBP-1c regulated LCN2 gene transcription. Moreover, treatment with holo-LCN2 stimulated intracellular iron accumulation and fibrosis gene expression in mouse HSCs, but this effect was not observed in SREBP-1cKO HSCs, indicating that SREBP-1c-induced LCN2 expression and secretion stimulate HSCs activation through iron accumulation. Further, LCN2 expression was strongly correlated with inflammation and fibrosis in patients with NASH. Our findings indicate that SREBP-1c regulates Lcn2 gene expression, contributing to diet-induced NASH. Reduced *Lcn2* expression in SREBP-1cKO mice protects against NASH development. Therefore, the activation of *Lcn2* by SREBP-1c establishes new connection between iron and lipid metabolism, affecting inflammation. These findings may lead to new therapeutic strategies for NASH.This work was supported by the Technology development Program (RS-2022-00167190) funded by the Ministry of SMEs and Startups(MSS, Korea) and Basic Science Research Program through the National Research Foundation of Korea (NRF) funded by the Ministry of Education (2021R1A6A3A01088315).

### [1LBA-006]
### Interrogation of the Neural Circuits Controlling Daily Torpor in Mice

*HIROSHI YAMAGUCHI[1] ([1]*Research Institute of Environmental Medicine, Nagoya University*)

Endotherms have the ability to endure low temperatures and periods of scarce food availability by actively entering a state of reduced metabolism known as torpor. While it's known that the brain orchestrates the reduction in both metabolic rate and body temperature (Tb) during torpor, the exact neural pathways involved in these mechanisms remain largely unexplored. In this study, we pinpoint the neural pathways that govern torpor regulation. This was achieved through a combination of whole-brain mapping of neurons activated during torpor, targeted manipulation of specific neural cell types, and circuit mapping using viral tracing techniques. Our findings reveal that multiple groups of neurons in the hypothalamus become active both prior to and during the torpor state. Through genetic silencing experiments, we have established that the activity of these neuronal groups plays a crucial role in torpor control. This study highlights the vital function of hypothalamic neurons in controlling Tb and metabolic rate during torpor and delineates key components of the neural network that regulates torpor.

### [1LBA-007]

### Regulatory mechanism of glucagon-like peptide-1 secretion by taurine in enteroendocrine cells

*Yuri Osuga[1], Kazuki Harada[1], Tetsuya Kitaguchi[2], Masami Hirai[3], Mitsuharu Matsumoto[4], Takashi Tsuboi[1] ([1]*Department of Life Sciences, Graduate School of Arts and Sciences, The University of Tokyo,* [2]*Laboratory for Chemistry and Life Science, Institute of Innovative Research, Tokyo Institute of Technology,* [3]*RIKEN Center for Sustainable Resource Science,* [4]*Dairy Science and Technology Institute, Kyodo Milk Industry Co., Ltd.*)

Glucagon like peptide-1 (GLP-1) is a type of gastrointestinal hormone secreted by enteroendocine L-cells in the small intestine. These cells secrete GLP-1 in response to the presence of nutrients including such as glucose, amino acids, and fatty acids. GLP-1 stimulates insulin secretion from pancreatic beta cells in a glucose-dependent manner and activates the afferent vagus nerve to suppress appetite.

Taurine is a sulphur-containing amino acid and is taken up by the body through dietary intake. Free taurine is present in the lower lumen of the small intestine and is reconjugated from taurocholic acid by the intestinal microbiota. However, the relationship between taurine in the gastrointestinal tract and the regulation of GLP-1 secretion is unclear.

In this study, we found that taurine promotes GLP-1 secretion from enteroendocrine L-cells in the small intestine. We administered taurine to a $Ca^{2+}$-sensitive fluorescent dye-loaded enteroendocrine cell line and performed intracellular $Ca^{2+}$ imaging. The results suggested that taurine treatment increased intracellular $Ca^{2+}$ levels through membrane depolarization mediated by closure of ATP-sensitive potassium channels.

In addition, we expressed a fluorescent protein-based ATP sensor, MaLionR, in an enteroendocrine cell line and performed intracellular ATP imaging. Taurine treatment increased the intracellular ATP levels. Taken together, it is suggested that taurine increases the intracellular ATP levels, and closes ATP-sensitive potassium channels, resulting in an increase in intracellular $Ca^{2+}$ levels and ultimately promoting GLP-1 secretion.

### [1LBA-008]

### Comprehensive lipidomics of lipid metabolic changes toward hibernation in the Syrian hamster.

*Akari Yamauchi[1,2], Junpei Yamashita[1], Yuki Sugiura[3], Yuta Matsuoka[3], Masamitsu Sone[1,2], Yoshifumi Yamaguchi[1,2] ([1]*Hibernation metabolism, physiology, and development Group, Inst. Low Temp. Sci., Hokkaido Univ.,* [2]*Biosphere Science, Grad. Sch. Env. Sci., Hokkaido Univ.,* [3]*Grad. Sch. Med., Kyoto Univ.*)

Lipids play crucial roles in homeostasis as storable sources for energy production and thermogenesis under starvation, flexible components of cell structures such as plasma membranes for thermal adaptation, and signaling molecules to change cellular and body status in response to extrinsic stimulus. To survive harsh seasons with starvation and cold, some mammals opt for hibernation, a strategy to suppress the basal metabolism and thermogenesis, thereby being lowering body temperature. Although importance of lipid storages for hibernation is well known, it remains to be elucidated how the changes of lipid profiles occur during hibernation and what roles it has in hibernation. In this study, we conducted lipidomic analysis to identify the lipid species whose amount changes during hibernation, with the final goal of determining important lipids for hibernation.
Syrian hamster (*Mesocricetus auratus*), a small mammalian hibernator, converts their bodies from the summer-active mode (Non-HIB, non-hibernation) to winter-mode by short photoperiod and cold temperature (SD-Cold) and begins to hibernation after several months of SD-Cold. Hibernation of small mammalian hibernators is characterized by multiday low body temperature state (deep torpor; HIB-DT) and normothermic state (periodic arousal; HIB-PA). Liver, brown adipose tissue (BAT), inguinal white adipose tissue (WAT) and plasma were collected from different states of animals; young adult at Non-HIB (warm), adult at Non-HIB (warm), adult at HIB-DT, and adult unresponsive hamsters (Un-HIB) , which are hamsters that did not hibernate under more than 7months of SD-Cold. The comprehensive lipid profile was obtained by non-targeted analysis using Orbitrap-mass spectrometry. This lipidomic analysis identified 1340 lipids in liver, 1620 in BAT, 1242 in WAT, and 1193 in plasma in total. Principal component analysis revealed HIB-DT differed from other groups of hamsters, particularly in liver. The lipids largely contributed to the HIB-DT were acylcarnitine (AcCa) species. The lipid species accumulated in various tissues only in HIB-DT (e.g., PI(22:6_18:1), PC(40:6)) were also identified. In addition, some lipids exhibited higher amounts only in un-HIB. Thus, we identified lipid species that specifically increased or decreased during HIB-DT or un-HIB. Regulation of these lipids may play important roles in hibernation.

### Late Breaking Abstracts

### [1LBA]
### Behavior, Biological rhythm, Sleep

March 28, 13:00 - 14:20, Poster Room

### [1LBA-009]

**The superior colliculus is critical for virtual head turns and eye movements during REM sleep**

*Yuta Senzai[1,2], Massimo Scanziani[1,2] ([1]*University of California, San Francisco,* [2]*Howard Hughes Medical Institute*)

Since the discovery of REM sleep, the nature of the rapid eye movements that characterize this sleep phase has remained elusive. Do they reveal gaze shifts in the virtual world of dreams or simply reflect random brainstem activity? In a previous study, we harnessed the head direction (HD) system of the mouse thalamus, a neuronal population whose activity reports, in awake mice, their actual HD as they explore their environment and, in sleeping mice, their virtual HD. We discovered that the direction and amplitude of rapid eye movements during REM sleep reveal the direction and amplitude of the ongoing changes in virtual HD, i.e. virtual head turns.

What coordinates the direction of rapid eye movements with that of virtual head turns during REM sleep? We have tested the role of the superior colliculus (SC) because, in awake animals, the SC coordinates eye and head movements to generate gaze shifts. We have discovered that the SC activity can predict the direction of rapid eye movements and virtual head turns during REM sleep. Furthermore, we have also discovered that silencing the SC has a major impact on virtual head turns during REM sleep. These discoveries suggest that the SC, by orchestrating sensorimotor representation in the sleeping brain, may mediate gaze shifts in the virtual world of REM sleep.

### [1LBA-010]

**Voluntary exercise prevents and alleviates pain-induced anxiety, potentially correlating with parvalbumin-positive neurons and perineuronal net in corticolimbic regions of rats**

*Thu Nguyen Dang[1], Tomoyuki Kurose[1], Kyo Nishio[1], Naoto Fujita[1], Hisao Nishijo[2], Norimitsu Morioka[3], Susumu Urakawa[1] ([1]*Department of Musculoskeletal Functional Research and Regeneration, Graduate School of Biomedical and Health Sciences, Hiroshima University,* [2]*Faculty of Human Sciences, University of East Asia, Ichinomiya Gakuen-cho, Shimonoseki City, Yamaguchi,* [3]*Department of Pharmacology, Graduate School of Biomedical and Health Sciences, Hiroshima University*)

Anxiety disorder emerged in the late phase of neuropathic pain. Whether this emotional disorder could be alleviated by exercise interventions and the neurobiological mechanisms underlying this ameliorative effect were unknown. This study was conducted on male Wistar rats with partial sciatic nerve ligation (PSL). Hyperalgesia following the PSL was assessed through the von Frey and Acetone tests. Anxiety-like behaviors were evaluated using open field (OF) and elevated plus maze (EPM) tests. Voluntary exercise in a running wheel was introduced either 3 days after PSL surgery (early voluntary exercise) or 4 weeks post-surgery (late voluntary exercise) when anxiety is already induced by pain. While PSL rats subjected to early exercise exhibited a reduction in hyperalgesia and did not show anxiety-like behaviors. Those subjected to late exercise presented an improvement in the manifestations of anxiety disorders, as indicated in the OF (measured by time spent and rearing activity in the central zone) and the EPM (evaluated through time spent, number of entries, and head dips in the open arm). Both early and late voluntary exercises not only ameliorated the mechanical and thermo threshold stimuli but also the level and duration of responses during the von Frey and Acetone tests. Furthermore, voluntary exercises were found to impact the changes of parvalbumin (PV)-positive neurons and their perineuronal net (PNN) in specific corticolimbic regions. These findings suggest that voluntary exercise holds therapeutic potential for both preventing the onset and alleviating pain-induced anxiety, potentially correlating with PV-positive neurons and PNN in specific corticolimbic regions.

### Late Breaking Abstracts

[1LBA]
**Study Methodology**

March 28, 13:00 - 14:20, Poster Room

### [1LBA-011]

### *In vivo* volumetric imaging in mouse brain using multibeam scanning two-photon microscopy

*Mitsutoshi Ataka[1], Kohei Otomo[2,3], Tomomi Nemoto[1,2] ([1]*National Institute for Physiological Sciences, National Institutes of Natural Sciences,* [2]*Exploratory Research Center on Life and Living Systems (ExCELLS), National Institutes of Natural Sciences,* [3]*Graduate School of Medicine, Juntendo University*)

This study presents an alternative approach for two-photon volumetric imaging that combines multibeam lateral scanning with continuous axial scanning using a confocal spinning-disk scanner and an electrically focus tunable lens. Using this proposed system, the brain of a living mouse could be imaged at a penetration depth of over 450 µm from the surface. *In vivo* volumetric $Ca^{2+}$ imaging at a volume rate of 1.5 Hz within a depth range of 130–200 µm, was segmented with an axial pitch of approximately 5-µm and revealed spontaneous activity of neurons with their 3D positions. This study offers a practical microscope design equipped with compact scanners, a simple control system, and readily adjustable imaging parameters, which is crucial for the widespread adoption of two-photon volumetric imaging.

### Late Breaking Abstracts

[1LBA]
**Others**

March 28, 13:00 - 14:20, Poster Room

### [1LBA-012]

### Involvement of delta opioid receptors in the preemptive analgesic effect of high-frequency transcutaneous electrical nerve stimulation in rats with acute inflammatory pain

*Hideshi Ikemoto[1], Naoki Adachi[1], Takayuki Okumo[1], Oyunchimeg Chuluunbat[1], Tadashi Hisamitsi[1], Masataka Sunagawa[1] ([1]*Department of Physiology, Showa University Graduate School of Medicine*)

Transcutaneous electrical nerve stimulation (TENS) is a treatment that employs electrical stimulation on the skin to induce analgesic effects. It is suggested that the effect and mechanism of TENS analgesia vary depending on the frequency. In this experiment, we investigated the involvement of delta opioid receptors (DOR) in the preemptive analgesic effects, which is the analgesic intervention before a painful stimulus to achieve better pain relief, of high-frequency TENS (HT: 100 Hz) using a rat model of acute inflammatory pain. Rats were randomly divided into four groups: a control group, a formalin-administered group, a formalin-administered group treated with HT, and an HT + formalin group treated with naltrindole (NTI), a DOR inhibitor, before HT. In the formalin-treated groups, formalin (1%, 50 µL) was subcutaneously injected into the plantar on the right hind paw. HT was applied for 30 min before formalin injection. NTI (0.1 µg/rat) was intracerebroventricularly administered 10 min before HT to investigate the involvement of DOR, whereas rats in the other groups were injected with Ringer's solution instead of NTI. The total time spent in pain-related behaviors such as licking, flinching, and lifting, was quantified for 60 min immediately after the formalin injections. We also observed the expression of phosphorylated extracellular signal-regulated kinase (pERK) and c-fos, which are used as markers of neural activation, in the spinal dorsal horn via immunofluorescence. As a result, the duration of pain-related behavior and the number of pERK- and c-fos-positive cells in spinal dorsal horn were significantly increased following the injection of formalin; however, the increase was significantly inhibited by HT. Moreover, the effects of HT were partially antagonized by NTI. These results suggested that the DOR plays a role in the preemptive analgesic effect of HT.

### [1LBA-013]

### COMBINED EFFICACY OF LY294002 AND OTS964 IN SUPPRESSING SELF-RENEWAL OF POWER-LAW CODED GLIOMA STEM CELL POPULATIONS

*MICHIYA SUGIMORI[1] ([1]*University of Toyama Faculty of Medicine*)

Glioblastoma, a primary brain tumor, is resistant to chemotherapy and can develop into a fatal space-occupying lesion. Glioma stem cells (GSCs) are thought to be responsible for tumor growth, chemo-resistance, and recurrence. Clonal glioma sphere (GS) culture, in which GSCs are enriched and self-renew as GS clone populations, provides us with quantitative details regarding GS clone survivability and changes in growth during GS/GSC population self-renewal. Previously, we proposed a novel chemotherapeutic paradigm, temozolomide (TMZ) and OTS964 in combination (T&O), and showed that T&O efficiently eliminated self-renewing GS clones and significantly suppressed the regrowth of TMZ-sensitive GS clones. However, it remained unclear whether T&O would be effective in treatment of TMZ-resistant GSC populations. T&O did not suppress T98-GS clone growth during population self-renewal, suggesting that TMZ-like growth suppression is necessary for the long-term control of GSC population size. In this study, we tested the PI3K inhibitor LY294002, which is thought to suppress GSC self-renewal, alone and in combination with OTS964 (L&O) against T98G-GS populations. LY294002 alone suppressed T98G-GS clone growth for 2–3 weeks, while allowing the clones to survive. By contrast, L&O efficiently eliminated two-thirds of the T98G-GS clones and continuously suppressed T98G-GS clone regrowth for 2–3 times longer than LY294002 alone, suggesting that L&O represents an alternative to T&O. Moreover, T98G-GS clones pre-treated with L&O exhibited a half survival rate in the following generations, suggesting that L&O treatment perturb the GSC self-renewal capacity. The L&O in combination led significant increase in the un-spliced form of p27 via synergistic splicing suppression; LY294002 strongly contributed spliced p27 up-regulation, suggesting that L&O suppresses GSC proliferation via up-regulation of un-spliced and spliced forms of p27 in combination. Our findings indicate that this quantitatively validated combination paradigm could control growth of TMZ-resistant GSC populations through immediate and sustained shrinkage of GSC populations exhibiting power-law governed growth diversity.

### Late Breaking Abstracts

Day 2
(March 29, 13:00 - 14:20)

[2LBA] Molecular physiology, Cell physiology
[2LBA] Embryology, Regenerative Medicine, Development, Growth, Aging
[2LBA] Blood, Lymph, Immunity
[2LBA] Urinary organ, Renal function, Urination
[2LBA] Stress
[2LBA] Pathophysiology

### Late Breaking Abstracts

[2LBA]

### Molecular physiology, Cell physiology

**Others**

March 29, 13:00 - 14:20, Poster Room

### [2LBA-001]

**Development of AAV vectors targeting various cell types in the mouse and marmoset brains**

*Hirokazu Hirai[1,2], Yuki Fukai[1], Hayato Kawabata[1], Ryo Aoki[1], Yasunori Matsuzaki[1,2], Ayumu Konno[1,2] ([1]*Gunma Univ Grad Sch Med,* [2]*Gunma Univ Viral Vector Core*)

In many central nervous system diseases, specific cell types are selectively damaged. For example, amyotrophic lateral sclerosis impairs spinal motor neurons, spinocerebellar ataxia damages cerebellar Purkinje cells, and multiple sclerosis affects oligodendrocytes. Additionally, recent studies indicate that neuroinflammation activates microglia and astrocytes, which deteriorate neurological diseases such as Alzheimer's disease, cerebral infarction, cerebral hemorrhage, and brain trauma, and accelerate brain aging. Therefore, functional regulation of specific cell types in the brain seems an effective therapeutic intervention to treat the above brain diseases and brain aging. We have been developing AAV vectors that enable efficient transgene expression in specific cell types in the brain. Here we will introduce AAV vectors that allow transgene expression specifically in excitatory neurons, inhibitory neurons, Purkinje cells, astrocytes, microglia, and oligodendrocytes in mice. Furthermore, we will present our recent data on whether those cell type-specific AAV vectors in mice function as cell type-specific vectors also in the common marmoset brain.

### [2LBA-002]

**Membrane Potential Modulates ERK Activity via Phosphatidylserine Dynamics**

*Mari Sasaki[1], Masanobu Nakahara[1], Takuya Hashiguchi[1], Fumihito Ono[1] ([1]*Osaka Medical and Pharmaceutical University*)

The plasma membrane potential has been associated with cell proliferation for more than 40 years. However, it has not been clear whether cell proliferation is directly regulated by membrane potential. Here we show that ERK (extracellular signal-regulated kinase) activity is regulated by the membrane potential. Membrane depolarization induced ERK activity in a voltage dependent fashion. Depolarization to 0 mV without any growth factor induced ERK signaling comparable to that with the EGF stimulation. This voltage-dependent ERK activity was regulated by phosphatidylserine dynamics of the membrane and not by the extracellular calcium entry. This study suggests the possibility that membrane potential may have diverse physiological functions beyond the action potential well-established in the neural system.

### [2LBA-003]

**M20 subunit inhibits auto-dephosphorylation of MYPT1 subunit in myosin light chain phosphatase**

*Tetsuo Yamashita[1], Masumi Eto[2], Katsuya Hirano[1] ([1]*Department of Cardiovascular Physiology, Faculty of Medicine, Kagawa University,* [2]*Biochemistry Unit, Faculty of Veterinary Medicine, Okayama University of Science*)

**Background**: Myosin light chain phosphatase (MLCP) dephosphorylates myosin light chain and suppresses vascular smooth muscle contraction. MLCP is a heterotrimer consisting of a catalytic subunit PP1c and two non-catalytic subunits MYPT1 and M20. The MYPT1 subunit suppresses the catalytic activity of PP1c when phosphorylated at either threonine-696 or -853 (T696 and T853) by protein kinases such as ROCK1/2 and ZIPK. However, the role of the other non-catalytic subunit M20 remains unclear.
**Main results**: The present study investigated the effect of M20 on the ROCK-dependent MYPT1 phosphorylation. MLCP trimer (PP1c + MYPT1 + M20) and dimer (PP1c + MYPT1) were expressed as recombinant proteins in *Saccharomyces cerevisiae* and purified by affinity chromatography. The activities of the purified MLCP dimer and trimer to dephosphorylate the phosphorylated MLC were 1,970 and 3,190 nmol/min/mg, respectively. The phosphorylation of MYPT1 was started by adding 1 mM ATP to 200 nM purified MLCP and 1 nM active ROCK2. The level of phosphorylation at T696 and T853 were evaluated by a dot blot analysis using the corresponding specific antibodies. In the presence of a phosphatase inhibitors (microcystin, orthovanadate and NaF), the phosphorylation at T696 and T853 rapidly increased in both MLCP dimer and trimer, reaching a plateau within 10 minutes, and stayed unchanged until 90 min. Thereafter, Y27632, a ROCK1/2 inhibitor was added; however, the levels of phosphorylation at T696 and T853 both remained fairly unchanged in the MLCP trimer. In the MLCP dimer, the levels of phosphorylation at T696 and T853 significantly decreased. In the absence of phosphatase inhibitors, the phosphorylation of T696 and T853 rapidly increased in MYPT1 trimer, but to the lower maximal levels of 82% and 53% of those obtained in the presence of phosphatase inhibitors. In the MYPT1 dimer, the phosphorylation of T696 and T853 gradually and linearly increased to the maximum levels of 53% and 44%, respectively.
**Conclusion**: The presence of M20 was related to the higher level of MYPT1 phosphorylation. M20 is suggested to inhibit the auto-dephosphorylation of MYPT1 in MLCP.

### [2LBA-004]

### Simulated Microgravity Affects Functional Morphology Involving the Prostaglandin Transporter SLCO2A1 in Human Umbilical Vein Endothelial Cells

*Saki Ishikawa[1], Taiga Nakayama[1], Reo Takizawa[1,3,4], Hideki Saito[1,3], Susumu Minamisawa[1], Hiroki Bochimoto[1,2] ([1]*Division of Aerospace Medicine, Department of Cell Physiology, The Jikei University School of Medicine*, [2]*Japan Aerospace Exploration Agency*, [3]*INAMI Space Laboratory Ltd*, [4]*Ushiku Aiwa General Hospital*)

### Late Breaking Abstracts

[2LBA]
**Embryology, Regenerative Medicine, Development, Growth, Aging**

March 29, 13:00 - 14:20, Poster Room

### [2LBA-005]

### Investigation of the frequency-regulated micro-vibration (FRMV)-mediated osteoblast differentiation mechanism in MC3T3-E1 Cells

*Ayumu Matsushita[1], Tada-aki Kudo[1], Yohei Hayashi[2,3], Kanako Tominami[1], Satoshi Izumi[1], Takakuni Tanaka[4], You-Ran Luo[4,5], Keiko Gengyo-Ando[1], Takuya Noguchi[6], Atsushi Matsuzawa[6], Guang Hong[4], Junichi Nakai[1] ([1]*Division of Oral Physiology, Graduate School of Dentistry, Tohoku University, [2]Cell Resourse Center for Biomedical Research, IDAC, Tohoku University, [3]Graduate School of Life Sciences, Tohoku University, [4]Division for Globalization Initiative, Tohoku University, [5]College & Hospital of Stomatology, Guangxi Medical University, [6]Laboratory of Health Chemistry, Graduate School of Phamaceutial Sciences, Tohoku University*)

Physical stimulation is a crucial factor influencing the metabolism of osteoblasts and their precursors, playing a pivotal role in bone remodeling. However, the role of micro-vibration, a type of physical stimulation that may efficiently induce osteoblastic differentiation, remains largely unclear. This study aimed to assess the mechanism of frequency-regulated micro-vibration (FRMV)-induced osteoblastic differentiation in the mouse pre-osteoblastic MC3T3-E1 cell line, a widely used experimental model in bone biology, osteogenesis, and skeletal development research. Before initiating FRMV stimulation, MC3T3-E1 cells were cultured for 2 or 3 days at 37˚C in an atmosphere of 5% $CO_2$/95% air in a growth medium. To induce differentiation, a waterproof micro-vibrator set in a $CO_2$ incubator was used to apply FRMV to cells incubated on culture plates. The cells in newly exchanged differentiating medium on the vibrator were exposed to FRMV (50 sec/hour) with a frequency either every day or four days per week for a maximum of 21 days. As a positive control, BMP2 was also used to induce osteoblast differentiation in the absence of FRMV exposure. The reagents for Alkaline phosphatase (ALP) activity assay and a BMP receptor inhibitor, LDN193189, were used to investigate the role of FRMV in osteoblastic differentiation. The results indicated that FRMV gradually increased the ALP activity of MC3T3-E1 cells by day 7, and the upregulated ALP activity level was maintained from day 7 to day 21 at almost the same level. Additionally, LDN193189 substantially inhibited the FRMV-mediated upregulation of ALP activity. Furthermore, a significantly different ability of FRMV to upregulate ALP activity was observed by comparing the two types of FRMV programs. These results suggest that FRMV can gradually upregulate the ALP activity of MC3T3-E1 cells within 7 days of FRMV stimulation, and the activity remains at the same level after day 7 during FRMV-induced osteoblastic differentiation in the cells with an unknown mechanism. Moreover, we also found that the mechanism of FRMV-mediated osteoblastic differentiation may be involved in the BMP signaling pathway, and the efficiency of FRMV on osteoblastic differentiation can change in an FRMV program-dependent manner. Taken together, FRMV may offer an effective bone regeneration technique and contribute to future regenerative medicine.

### [2LBA-006]

### Effects of ethanol exposure on cardiac development in chick embryos imaged with swept source OCT

*Ryuichiro Yamazaki[1], Tomoya Tanaka[1], Takashi Yamaoka[1], Yuuta Moriyama[1,2], Toshiyuki Mitsui[1] ([1]*Aogaku Univ. Dept of Phys.*, [2]*PREST JST*)

### [2LBA-007]

### Changes in Reinforcement Learning Ability with Aging

*Hiroyuki Ohta[1], Takashi Nozawa[2], Takashi Nakano[3], Yuji Morimoto[1], Toshiaki Ishizuka[1] ([1]*National Defense Medical College*, [2]*Mejiro University*, [3]*Fujita Health University*)

Does aging hinder learning from failure experiences or learning from success experiences? In this study, we quantified changes in learning ability due to aging in mice using the 5-armed bandit task (5-ABT) and a computational Q-learning model. As a result, we found that aged mice (18 months old) had a reduced ability to learn from failures compared to adult mice (12 months old) and young mice (3 months old). Also, the ability to utilize what has been learned to choose actions efficiently increases with adulthood but decreases with aging. These findings prompt a rethinking of rehabilitation methods in aging and dementia. Furthermore, the methodology presented in this study provides new evaluation criteria for dementia model mice.

### [2LBA-008]

### A viral vector strategy for labeling and analyzing human cortical organoids

*Shota Adachi[1], Masatoshi Nishimura[1], NGOMai Thuy[1], Akinori Sato[1], Ryosuke F. Takeuchi[1], Fumitaka Osakada[1,2] ([1]*Laboratory of Cellular Pharmacology, Graduate School of Pharmaceutical Sciences, Nagoya University, Japan,* [2]*Laboratory of Neural Information Processing, Institute for Advanced Research, Nagoya University, Japan*)

The major goals of neuroscience are to understand the development, structure, and function of the human brain and to treat neurological and psychiatric disorders. However, research on the human brain has predominantly relied on postmortem analysis and non-invasive imaging. Animal models have been extensively used *in vitro* and *in vivo*, but it is uncertain whether they are sufficient to elucidate the human brain, which differs in structure and function from the animal model brain. Limited access to the human brain faces a challenge in studying normal and abnormal neural processes. However, recent advances in induced pluripotent stem cell (iPSC) and organoid technologies have provided unprecedented opportunities to study the human brain *in vitro* and *ex vivo*.Neural organoids, 3D brain-like structures differentiated *in vitro* from pluripotent stem cells, hold considerable potential for advancing human brain research. Nevertheless, the complexity of human neural organoids limits in-depth analysis. To analyze human neural organoids at the molecular, cellular, and circuit levels, we employed viral vectors, such as adeno-associated virus vectors (AAVs), enabling efficient and conventional labeling and cell-type-specific manipulation.AAVs can express transgenes using promoters and enhancers for extended periods with low toxicity, making them valuable for gene transfer in the brain. However, the efficiency of gene transfer by AAVs varies among cell types and species depending on their serotypes, necessitating optimization for effective labeling in human neural organoids. In this study, to establish viral approaches in human neural organoids, we generated human cortical organoids (hCOs) by re-aggregating completely dissociated iPSCs and evaluated the infection efficiency of different capsid AAVs. We generated various AAVs, including the naturally occurring AAV capsids AAV-2, AAV-6, and AAV-9, and the newly engineered AAV capsids AAV-DJ, AAV-PHP.eB, AAV-Cap-Mac, and AAV-2-retro. Titer-matched AAVs-CAG-EYFP-WPRE3 were infected into hCOs on day 72 and analyzed on day 89. Immunostaining of frozen sections of the infected organoids determined the percentage of virus-infected, EYFP-positive cell area. FACS analysis quantified the percentage of EYFP-positive cells in the organoids. Furthermore, EYFP-positive cells were compared qualitatively and quantitatively by immunostaining of CUBIC-treated transparent hCOs. Finally, we performed two-photon calcium imaging to determine the function of AAV-infected neurons in hCOs. This poster presents comprehensive data comparing AAVs in human neural organoids. The AAV-based approaches will facilitate molecular, cellular, and circuit-level analyses in human neural organoids.

### Late Breaking Abstracts

[2LBA]
**Blood, Lymph, Immunity**

March 29, 13:00 - 14:20, Poster Room

### [2LBA-009]

### Analysis of the anti-inflammatory effects of plasma from a mammal hibernator, the Syrian hamster.

*Fuuka Okutsu[1,2], Yoshifumi Yamaguchi[1,2] ([1]*Hokkaido university,* [2]*Institute of Low Temperature Science*)

Hibernation, a survival strategy employed by some mammals and birds facing starvation and cold, involves a state of low metabolism and body temperature (Tb). Syrian hamsters, small mammal hibernator, do not hibernate in a summer-like, warm long-day conditions (non-hibernating periods; non-HIB), but when exposed to a winter-like, short-day cold conditions (SD-cold), they begin to hibernate after several months (pre-hibernation period; pre-HIB). During the hibernation period (HIB), they repeat two states; deep torpor (HIB-DT) with a low Tb and immobile state, and periodic arousal where body temperature quickly rises to 37°C and hamster resume activity. Several studies suggest that blood humoral factors are involved in the dynamic metabolic changes associated with hibernation. However, few studies have examined the effects of these factors on the immune system of hibernating animals.In this study, we focused on macrophages, key players in the innate immune system, and investigated the influence of hamster plasma on their inflammatory responses. Bone marrow-derived macrophages (BMDM) from hamsters were cultured in media containing hamster plasma from animals at non-HIB, pre-HIB, HIB-DT, or unHIB, which did not hibernate under long-term SD-cold. As a comparison group, groups treated with fetal bovine serum (FBS) or horse serum (HS), or mouse plasma were also prepared. After 1 day of culture, the inflammatory response was induced by addition of Lipopolysaccharide (LPS), a component of Gram-negative bacteria, and the gene expression levels of inflammatory cytokines were examined by qPCR. The expression of Cxcl9 and IL-1β was significantly suppressed in all four groups cultured in medium containing hamster plasma compared to the groups treated with FBS, HS or mouse plasma. This anti-inflammatory effect was also observed to a slight extent in mouse BMDM, indicating a potential cross-species impact. Furthermore, the anti-inflammatory effects depend on diet given to the hamsters, suggesting the contribution of metabolites derived from diets.

### Late Breaking Abstracts

[2LBA]
**Urinary organ, Renal function, Urination**

March 29, 13:00 - 14:20, Poster Room

### [2LBA-010]

### The Effects of cGMP on mechanosensitivity of TRPC6 channel and the filtration barrier function reinforcement in cultured glomerular podocytes

*Jun Ichikawa[1], Midori Nakagawa[2], Ryuji Inoue[2] ([1]*Sano Nihon Univ. Col.*, [2]*Dept. Physiol., Fukuoka Univ. Sch. Med.*)

Glomerular podocytes express the canonical transient receptor potential 6 (TRPC6) channel, the activity of which is subject to both agonistic and mechanical stimuli such as neurohumoral factors and blood flow/pressure, respectively. We recently found that mechanical stimulation rather suppressed the receptor-mediated activation of TRPC6 channel in cultured murine podocytes by using the intracellular $Ca^{2+}$ imaging and whole-cell patch clamp techniques. And this was accompanied by the inhibition of FITC-labelled albumin leak across the cell-culture insert membrane. Meanwhile, receptor-activated TRPC6 channels heterologously expressed in HEK293 cells underwent upregulation by mechanical stimuli while negative regulation by cGMP through protein kinase G-mediated phosphorylation (Takahashi *et al., J.Physiol.*, 586, p4209-4223, 2008; Inoue R. *et al.*).
In this study, we therefore addressed the question of how cyclic GMP (cGMP) affects the above-described effects of mechanical stimulation. We prepared immortalized mouse podocytes stably expressing wild-type TRPC6. Pretreatment of these cells with 8Br-cGMP, an analogue of cGMP, moderated the suppression of $Ca^{2+}$ influx by mechanical stimulation, and partially reversed the concomitant inhibition of the albumin leakage. These results suggest that albeit the positive correlation between the $Ca^{2+}$ influx and albumin leak, the actions of cGMP on TRPC6 channel would depend on the cellular context with respect to its receptor-mediated activation and mechanical modulation. COI:NO

### Late Breaking Abstracts

[2LBA]
**Stress**

March 29, 13:00 - 14:20, Poster Room

### [2LBA-011]

### Depression state enhances emotional contagion for pain

*Satoshi Kitazume[1], Kisho Obi-Nagata[1,2], Noriyuki Koibuchi[1,2] ([1]*Gunma University School of Medicine*, [2]*Gunma University Graduate School of Medicine*)

Depressed patients often show personality changes such as excessive empathy, loss of objectivity, and blurred boundaries between self and others, which often exacerbate depressive symptoms and interfere with treatment. However, it is unclear how the depressive state affects empathy and self-identification. To elucidate the neurobiological basis for the effects of depression on empathy and emotional contagion, we examined how depression model mice respond to the pain of other mice using social pain transfer, a behavior that is a primitive form of empathy toward others.

We found that both the chronic corticosterone-treated model and the chronic social defeat stress model exhibited prolonged empathy-like behavior, as indicated by longer-lasting mechanical hypersensitivity induced by the interaction with a cagemate experiencing inflammatory hyperalgesia. Furthermore, we found that the prolonged empathy-like behavior in the depression model mice was mainly generated by visual input. This finding suggests that the state of depression affects emotional contagion in mice models and is an important step in understanding the physiological background of depression-related altered empathy. Our next step is to clarify the brain regions associated with this prolonged emotional contagion in the depression model by circuit manipulation using optogenetic and pharmacogenetic tools.

### Late Breaking Abstracts

[2LBA]
**Pathophysiology**

March 29, 13:00 - 14:20, Poster Room

### [2LBA-012]

### Reconsidering the mechanism of S-T segment elevation that appears on electrocardiograms in acute myocardial infarction

*Noritaka Okamura[1] ([1]*Ehime Prefectural University of Health Sciences*)

This study was conducted to confirm the injury current theory regarding S-T segment elevation in the electrocardiogram during acute myocardial infarction. When the ventricular muscle is at rest, the injury current flows from the damaged part to the healthy part, causing the baseline of the electrocardiogram to drop.

In this study, electrocardiograms were recorded using Xenopus laevis while an electric current was passed from the outside of the ventricular muscle to the contralateral ventricular muscle lateral during its resting phase.

When a current was passed from the left side of the ventricle to the right side during the resting phase of the ventricular muscle, S-T segment shifted downward in leads I and II. When current was applied from the lower side to the upper side of the ventricular muscle, S-T segment shifted downward in leads II and III. On the other hand, when the left lateral part of the ventricular muscle was ablated, S-T segment elevation occurred in leads I and II. Moreover, when the outside of the apex of the heart was ablated, S-T segment elevation occurred in leads II and III.

These results refute the conventional explanation of injury current as the mechanism of S-T segment shift in electrocardiograms during acute myocardial infarction. We propose that S-T segment elevation during acute myocardial infarction should be discussed in terms of changes in the intrathoracic electric field depending on the region where the resting membrane potential disappears.

Authors have no COI to disclose in relation to the presentation.

### [2LBA-013]

### Simultaneous disturbance of NHE1 and LOXL2 decreases tumorigenicity of head and neck squamous cell carcinoma

*Kodai Nagashio[1], Yuji Hayashi[2], Itaru Watanabe[1], Shoko MIyoshi[1], Mohammed E Choudhury[1], Hajime Yano[1], Naohito Hato[2], Junya Tanaka[1] ([1]*Department of Molecular and Cellular Physiology, Graduate School of Medicine, Ehime University,* [2]*Department of Otorhinolaryngology, Head and Neck Surgery, Ehime University Medical School, Graduate School of Medicine, Ehime University*)

Objective: Although there have been brilliant advancements in the practical application of therapies targeting immune checkpoints, achieving success in targeting the microenvironment remains elusive. In this study, we aimed to address this gap by focusing on NHE1 and LOXL2, which are upregulated in head and neck squamous cell carcinoma (HNSCC) cells.Methods: The malignancy of a metastatic human HNSCC cell line was assessed in a mouse tongue cancer xenograft model by knocking down (KD) NHE1, responsible for regulating intracellular pH, and LOXL2, responsible for extracellular matrix (ECM) reorganization via cross-linking of ECM proteins. In addition to assessing changes in PD-L1 levels and collagen accumulation following knockdown, the functional status of the PD-L1 / PD-1 immune checkpoint was examined through co-culture with NK92MI, a PD-1 positive phagocytic human Natural Killer (NK) cell line.Results: The tumorigenic potential of each KD cell line was similar to that of the control cells, whereas the potential was attenuated in cells with simultaneous KD of both factors (double knockdown [dKD]). Additionally, we observed decreased PD-L1 levels in NHE1 KD cells and compromised collagen accumulation in LOXL2 KD and dKD cells. NK92MI cells exhibited phagocytic activity toward HNSCC cells in co-culture, and the number of remaining dKD cells after co-culture was the lowest in comparison to the control and single KD cells.Conclusion: This study demonstrated the possibility of achieving efficient antitumor effects by simultaneously disturbing multiple factors involved in the modification of the tumor microenvironment.

### Late Breaking Abstracts

Day 3
(March 30, 13:00 - 14:20)

[3LBA] Neurophysiology, Neuronal cell biology - Plasticity
[3LBA] Neurophysiology, Neuronal cell biology - Neural network
[3LBA] Neurophysiology, Neuronal cell biology - Glia
[3LBA] Neurophysiology, Neuronal cell biology - Sensory function, Sensory organ
[3LBA] Molecular physiology, Cell physiology - Membrane transport
[3LBA] Molecular physiology, Cell physiology - Ion channels, Receptors
[3LBA] Muscle
[3LBA] Oral physiology
[3LBA] Drug Action, Pharmacology

### Late Breaking Abstracts

[3LBA]
### Neurophysiology, Neuronal cell biology
**Plasticity**

March 30, 13:00 - 14:20, Poster Room

### [3LBA-001]

**Study on the alleviating effect of Moutan Radicis Cortex on neuronal plasticity decline caused by inflammation**

Young-Ki Ham[1], *In-Seo Lee[1], Gyo-Ha Hwang[1], Kyun-Seob Park[1], Su-Bin Jung[1], Ji-Won Yoon[1], Jun-Seok Suh[1], Ji-Ho Park[1] ([1]*Kyung Hee University*)

Inflammation is a response triggered by harmful stimuli and conditions, such as infection and tissue damage, and is responsible for a variety of physiological and pathological processes. In this paper, it was investigated at the relationship between inflammation and long-term potentiation (LTP) in hippocampal slices caused by inflammation-inducing substance through electrophysiological methods and protein expression level evaluation. Accordingly, it was confirmed that in the LTP of hippocampus, the treatment of Moutan Radicis Cortex (MRC) extract protects LTP from the decrease in LTP caused by inflammation-inducing substance. In the protein expression level evaluation, the expression level of inflammatory cytokines and neurotrophic factors showed that the group treated simultaneously with inflammatory cytokines and inflammatory inducers decreased the expression of inflammatory cytokines and increased the expression of neurotrophic factors compared to the group treated only with inflammatory inducers. showed. However, since there was excessive expression, which was thought to be caused by toxicity, in the group treated only with the extract, it was believed that the efficacy of the extract was not in the aspect of suppressing inflammation, but rather in protecting against inhibition of LTP by increasing the expression of neurotrophic factors. Therefore, considering that the results of this study showed that MRC was toxic at high concentrations and also had significant bioactive effects at low concentrations, it seemed that an appropriate dose of MRC might be useful for use as a defense agent against neuroplasticity.

### [3LBA-002]

**Paired associative premotor-cerebellar stimulation modulates cerebellar function depending on interstimulus intervals in healthy subjects**

*Satoko Koganemaru[1], Kazuki Tanaka[1], Atsushi Shima[1], Cao Yedi[1], Tomoaki Miyake[2], Tatsuya Mima[3] ([1]*Department of Regenerative Systems Neuroscience, Human Brain Research Center, Graduate school of Medicine Kyoto University,* [2]*Department of Neurology, Graduate school of Medicine Kyoto University,* [3]*The Graduate School of Core Ethics and Frontier Sciences, Ritsumeikan University*)

**[Background]** Paired associative stimulation (PAS) using two stimulator machines of transcranial magnetic stimulation (TMS) on different cortical areas, has developed to induce brain plastic changes in human. Based on the mechanism of spike-timing dependent plasticity (STDP), the interval between the two stimuli over pre- and post-synaptic sites determines which plasticity is induced, potentiation or depression. Recently, we have found that the PAS of the cerebellum and the contralateral premotor area (PMA) could enhance cerebellar function in healthy participants. However, it is unknown whether cerebellar function can be affected by altering the inter-stimulus interval of the premotor-cerebellar PAS method.
**[Methods]** Twenty-three healthy adults (11 females) were given the PAS in which 120 times of paired stimuli of the first stimulus on the right PMC (intensity: 90% rMT) followed by the second stimulus on the left cerebellum (intensity: SI 1mV) with interstimulus interval (ISI) of 5 (PAS-5), 20 (PAS-20) and 40 msec (PAS-40) every six seconds, 120 times in total in three different days (PAS-5, PAS-20 and PAS-40, respectively). We evaluated the cerebellar brain inhibition (CBI) in the left cerebellum.
**[Results]** After PAS-5 (ISI of 5 msec), CBI was inhibited, while CBI was enhanced after PAS-20 (ISI of 20 msec). CBI was not changed after PAS-40 (ISI of 40 msec).
**[Discussion]** The change of CBI was affected by the time interval between the two stimuli in the premotor-cerebellar PAS. It suggests that the cerebellar function can be modulated based on the STDP mechanism.

### Late Breaking Abstracts

[3LBA]
**Neurophysiology, Neuronal cell biology**
**Neural network**

March 30, 13:00 - 14:20, Poster Room

### [3LBA-003]

### Parabrachial *Cck* neurons involved in the feedback control of thirst

*Takashi Matsuda[1], Kenta Kobayashi[2], Kazuto Kobayashi[3], Masaharu Noda[1] ([1]*Tokyo Institute of Technology, Institute of Innovative Research, Homeostatic Mechanism Research Unit,* [2]*National Institute for Physiological Sciences, Section of Viral Vector Development,* [3]*Fukushima Medical University School of Medicine, Institute of Biomedical Sciences, Department of Molecular Genetics*)

In terrestrial animals, including humans, central mechanisms controlling thirst are important for maintaining the volume of body fluids and the concentration of sodium ([$Na^+$]) at physiological levels. Thirst is controlled by multiple factors reflecting body fluid conditions, such as the level of [$Na^+$], osmolality, and angiotensin II (Ang II). We previously identified and characterized Ang II receptor (AT1a)-positive glutamatergic neurons in the subfornical organ (SFO) that drive thirst (water neurons) based on body fluid conditions (Matsuda et al., *Nat. Neurosci*., 2017): Water neurons projected to the preoptic area, such as the median preoptic nucleus (MnPO) and organum vasculosum of the lamina terminalis. On the other hand, animals also rapidly and temporarily stop water intake after the ingestion of water based on signals originating from digestive organs. We have already revealed that water neurons in the SFO were temporarily suppressed in response to water intake (Matsuda et al., *Nat. Neurosci*., 2017; Matsuda et al., *Nat. Communi*., 2020). The parabrachial nucleus (PBN) is the relay center of ingestion signals from the digestive organs. We here identified neuronal populations expressing *cholecystokinin* (*Cck*) mRNA in the lateral PBN (LPBN) that are activated in response to water intake. The *Cck* neurons in the dorsal lateral compartment of the LPBN projected to GABAergic neurons in the MnPO. The optogenetic stimulation of these *Cck*-positive projection neurons suppressed thirst under water-depleted conditions. The combination of optogenetics and *in vivo* $Ca^{2+}$ imaging during ingestion revealed that the *Cck* neurons control the activity of GABAergic neurons in the target nucleus. Consistently, the optogenetic activation of GABAergic neurons in the MnPO suppressed water intake to the same extent as the optogenetic activation of *Cck* neurons. Therefore, the MnPO appears to be a control center integrating multiple signals for the promotion and suppression of thirst. These findings provide the feedback mechanisms for the suppression of thirst after ingestion.

### [3LBA-004]

### Effects of Visually Induced Backward Self-Motion Sensation on Memory Retrieval

*Kyoko O'Neill[1], Shigeru Kitazawa[1,2,3] ([1]*Graduate school of Frontier Biosciences, Osaka University,* [2]*Graduate School of Medicine, Osaka University,* [3]*Center for Information and Neural Networks (CiNet)*)

Background: Spatial and temporal concepts in the human mind are interconnected. It has been reported that forward body movements are associated with future-oriented thinking, while backward movements are linked to past-oriented thinking (Miles et al., 2010). In this study, we conducted two psychological experiments and functional imaging based on the hypothesis that memory recall, a key example of past-oriented thinking, is facilitated by the sensation of backward body movement (vection). Methods: Participants memorized 72 images of objects. Then, in the recall task, participants were shown 144 images (including 72 previously seen and 72 new images) one by one at the screen center (visual angle: 23° x 23°). To induce a sensation of self-motion (vection), we played either a video simulating forward or reverse movements as if seen from a car's driver seat (Exp.1), or a video of expanding/contracting random dots (Exp.2) in the peripheral field of view (72° x 105°). Participants responded to each image with three choices: "definitely seen", "probably seen", or "never seen". Brain activity was also measured using a 3T MRI while four types of random-dot videos (expansion, contraction, random, and static) were presented with a viewing angle of 100° x 100°. Results: We found no significant overall difference in memory recall between forward and backward movement sensations. However, detailed analysis revealed that memory recall continuously decreased from the first to the last image with forward movement, and improved in the second half of the experiment with backward movement. Brain imaging indicated increased activity in the bilateral primary visual cortex and the lingual gyrus (a brain region connected to the hippocampus, key for memory) during backward movement, but no significant change in the hippocampus itself. Conclusion: These results suggest that the sensation of moving backward may enhance memory recall by indirectly influencing the hippocampus through increased activity in the lingual gyrus.

### Late Breaking Abstracts

[3LBA]
**Neurophysiology, Neuronal cell biology**
**Glia**

March 30, 13:00 - 14:20, Poster Room

### [3LBA-005]

### Association between Microglia and ADHD-like Behaviors: Analysis Using a Novel Animal Model of ADHD, the Lister-Hooded Rat

*Chisato Yajima[1], Junya Tanaka[1], Hajime Yano[1] ([1]*Department of Molecular and Cellular Physiology, Ehime University Graduate School of Medicine*)

Microglia are the only mesodermal cells in the brain parenchyma and have been implicated in various psychiatric disorders. However, their association with ADHD(attention deficit hyperactivity disorder) is unknown. We focused on microglia in the brain of a newly discovered rat model of ADHD, the Lister Hooded Rat (LHR).
LHRs are non-albino outbred rats with black hair from head to back, and are characterized by hyperactivity and persistent hyperactivity in the same environment compared to spontaneously hypertensive rats (SHRs), which have long been used as a model of ADHD. Inattention is also prominent, with a higher frequency of falls in the drop from height test. Hyperactivity and inattention improved with treatment with the ADHD drug atomoxetine, and similar down-regulation was observed in the frontal lobe for eight genes whose expression variation has been reported in human ADHD. In addition, compared to Wistar rats, a common albino rat used as a control, there was a characteristic increase in expression of the Fos gene and cFos protein, which indicate increased neuronal activity in the posterior region of the medial aspect of the frontal lobe (Prelimbic region; PrL) (Jogamoto et al. 2020; Neurochem Int). It has also been shown that axonal primordia are shortened in LHR, such as in frontal lobe neurons (Usui et al. 2022; Neurochem Int).
In the present study, we found that the number of microglia was significantly reduced in the frontal lobe, especially in the orbitofrontal cortex, in LHR compared with Wistar rats, CD11b, which is involved in synaptic phagocytosis, was less expressed. We are currently investigating the relationship between behavioral changes and microglial activity by administering clenbuterol, an adrenergic β2 receptor agonist that increases microglial activity.

### [3LBA-006]

### Cortical-wide energy dynamics in REM sleep

*Yusuke Takahashi[1], Yoko Ikoma[1], Ko Matsui[1] ([1]*Super-network Brain Physiology, Graduate School of Life Sciences, Tohoku University*)

Glucose consumption in the brain reaches 20% of the total, although it only occupies 2% of the total body mass in humans. On-demand energy delivery and smart consumption systems are equipped in the innate brain. Depending on the cognitive task, sudden increases or decreases in local energy demand occur. Cerebral blood flow and glucose metabolism rapidly adapt in response to changes in these demands through the 'neuro-metabolic coupling' mechanisms. However, high energy-demanding computation may topple the balance of cell energy constancy. Dreams during REM sleep are assumed to be the representations of the optimization of past experience database. Vast amount of energy may be required for such information processing. The energy dynamics associated with the transition from non-REM sleep to REM sleep were studied. Here, a wide-field fluorescence imaging technique was employed to measure the dynamics of cerebellar blood flow, energy biomolecule pyruvate in astrocytes, and ATP in neurons through the intact skull of transgenic mice. Fluorescence from the albumin-mScarlet expressed in the bloodstream increased during REM sleep indicating an increase in the cerebral blood vessel diameter, consistent with the assumption that brain activity during REM sleep requires high energy. FRET-based fluorescence sensor for pyruvate was selectively expressed in astrocytes. Intracellular pyruvate level dropped in astrocytes with REM sleep. Apparently, the consumption of pyruvate overwhelmed the enhanced supply of glucose and oxygen from the blood vessels. The pyruvate could have been used as an energy substrate for ATP production in the mitochondria in astrocytes, or it could have been released extracellularly to be handed over to neurons. Using another ATP sensor expressed in neurons, it was found that neuronal ATP level also drops with REM sleep. Either 1) pyruvate/lactate supply from astrocytes was reduced, 2) ATP production in neuronal mitochondria was reduced, or 3) ATP consumption was greatly increased in neurons. Interestingly, the decline in neuronal ATP began in the posterior cortex and propagated throughout the entire cortex over approximately 10 seconds. This suggests that the entire cortex neurons do not transition into REM sleep synchronously; instead, specific cellular activities in the posterior cortex may trigger the transition to REM sleep.

### Late Breaking Abstracts

[3LBA]

### Neurophysiology, Neuronal cell biology

**Sensory function, Sensory organ**

March 30, 13:00 - 14:20, Poster Room

### [3LBA-007]

### Optogenetic gamma-band entrainment of parvalbumin-positive neurons in the primary sensory cortex facilitates the activity of spinal dorsal horn neurons and withdrawal response in mice

*Tomofumi Otsuki[1,2], Daisuke Uta[3], Karin Yamada[3], Mayuka Tateno[1], Yuto Koide[1,5], Jumpei Matsumoto[1,4], Tsuyoshi Setogawa[1,4], Hiroshi Nishimaru[1,4] (*[1]System Emotional Science, Faculty of Medicine, University of Toyama, [2]Graduate School of Innovative Life Science, University of Toyama, [3]Department of Applied Pharmacology, Faculty of Pharmaceutical Sciences, University of Toyama, [4]Research Center for Idling Brain Science (RCIBS), University of Toyama, [5]Department of Engineering, School of Engineering,University of Toyama*)

The primary somatosensory cortex (S1) is the main region in the cerebral cortex that receives, and processes pain information conveyed from the periphery. Previous studies have shown that cortical oscillations in the gamma frequency band (30-100 Hz) in the S1 are associated with pain in humans and rodents. In a recent study, it has been demonstrated that 40 Hz optogenetic entrainment of parvalbumin-positive (PV) neurons localized in S1 (S1-PV-gamma-activation) enhanced nociceptive sensitivity (Tan et al., Nat Commun. 2019). It was indicated that the effects were mediated via the descending serotonergic pain modulation system, which modulates the neuronal activity in the spinal dorsal horn (SDH) neurons. However, it remains unclear which side of the body is affected and whether the neural activity of SDH neurons on the affected side is altered. In this study, we investigated the nociceptive behavior in bilateral hind paw and the temporal changes of neuronal activity in SDH neurons during and after the S1-PV-gamma-activation in transgenic mice expressing channelrhodopsin (ChR2) in PV neurons (PV-ChR2 mice). To examine the nociceptive behavior, we performed the von Frey test in freely moving mice up to 30 minutes after the S1-PV-gamma-activation (40 Hz, 5-s stimulation, 20-s interval, 5 cycles) in the unilateral S1. Non-noxious filament (0.07 g) was applied to the planter surface of the hind paw on both sides. During activation, withdrawal response in the contralateral hind paw significantly increased but not in the ipsilateral one in PV-ChR2 mice. Furthermore, we confirmed the effect of the S1-PV-gamma-activation on contralateral SDH neurons, extracellular single-unit recording *in vivo* was performed in urethan-anesthetized PV-ChR2 mice. There was no significant change in spontaneous firing in all the neurons examined (n = 7). However, the firing rate significantly increased (approximately 2-fold) in response to punctate mechanical force (0.4/4.0 g) to the plantar surface of the hind paw. The effect was transient, and the response returned to baseline after 30 min. Our findings suggest that the S1-PV-gamma-activation transiently induces allodynia and hyperalgesia in the contralateral hind paw by increasing the firing rate of SDH neurons in response to mechanical stimulation.

### [3LBA-008]

### Screening of lipid regulatory genes involved in temperature sensation

*Hinata Yamanaka[1,2,3], Takuto Suito[2,3], Takaaki Sokabe[1,2,3] (*[1]Physiological Sciences Program, SOKENDAI, [2]Division of Cell Signaling, National Institute for Physiological Sciences, [3]Thermal Biology Group, Exploratory Research Center on Life and Living Systems*)

Transient Receptor Potential (TRP) channels are membrane receptors that sense a wide range of temperature changes in animals. The regulatory mechanisms of TRP channels are one of the most exciting questions in sensory processes. Recent studies have indicated a dual role for plasma membrane lipids: they provide a protective barrier between the inside and outside of the cell and behave as regulators of membrane protein function. This regulation is mediated by lipid-protein interactions and is influenced by the physicochemical properties of the membrane. However, previous studies have focused only on specific lipids, and comprehensive analyses are needed to elucidate the relationship between lipids and membrane proteins.

To seek lipids that are potentially important for channel functions, we performed an RNA-sequencing analysis of isolated sensory neurons expressing either thermos-sensitive TRPA1 or TRPL in *Drosophila* larvae. We identified multiple genes involved in lipid metabolism that were highly expressed in TRPA1- and TRPL-expressing sensory neurons compared to whole body. We then knocked down these candidate genes specifically in neurons to evaluate their physiological roles in temperature sensation by analyzing thermotaxis of larvae.

We will discuss the potential contribution of lipid regulatory genes and lipid products to sensory functions, which could provide new insights into the regulatory mechanisms of sensory molecules.

### [3LBA-009]

### Loss of MAP2 causes a disruption of the signaling process in the auditory peripheral system

*Kazuki Shin'ya[1], Tomohiro Miyasaka[2], Kohta Kobayasi[1] (*[1]Doshisha University, [2]Nihon University*)

MAP2 is one of the major neural microtubule-associated proteins (MAPs) and evolutionally conserved from *C. elegans* to mammals. This suggests its importance in neuronal function. However, the importance of MAP2 for the development and maintenance of nervous system functions remains unclear. To elucidate the function of MAP2 *in vivo*, we observed the behavior of MAP2 knockout (MKO) mice. We found that MKO mice normally develop into adult without any apparent abnormal bodily movement but show a reduced surprise response or avoidance to loud noise. Therefore, we hypothesized that the loss of MAP2 causes hearing loss, and measured the auditory brainstem response (ABR) to 2, 4, 8, 16, 32, 50, 60, 64, 68, and 72 kHz sound stimuli to verify the function of MAP2 in the auditory signal processing. Response thresholds of MKO mice were higher than those of wild type (WT) mice at 4 kHz -32 kHz, with a maximum increase of 40 dB at 16 kHz. ABR above 50 kHz was not observed in MKO mice even with the loudest stimulus (90 dB SPL). In addition, there was an increase in response latency and a decrease in amplitude, which are characteristic of sensorineural hearing loss. To clarify the site causing the hearing loss, we observed the localization of MAP2 in the cochlea using wholemount staining. MAP2 was expressed in the cochlear outer hair cells (OHC), inner hair cells (IHC), and cochlear neurons. The amount of MAP2 expression was higher in the OHC than IHC. In addition, the number of OHCs of base turn of the cochlear decrease in MKO. The number of IHCs did not change in any turn. To identify the location of damage in OHCs, we observed the length of stereocilia of OHC at apex, middle and base turn using scanning electron microscopy. Loss of MAP2 gene did not change stereocilia length in OHCs. These results suggest that MAP2 contributes to the maintenance of hearing sensitivity to sound, especially in the high frequency range. The possible function of microtubules for converting mechanical to electrical stimuli in OHC will be discussed.

### [3LBA-010]

### Calculation of the neural activity timing of the primary somatosensory cortex

*Tatsuya Umeda[1], Kazuhiko Seki[2], Takashi Hanakawa[1] ([1]*Department of Integrated Neuroanatomy & Neuroimaging, Kyoto University Graduate School of Medicine,* [2]*Department of Neurophysiology, National Center of Neurology and Psychiatry*)

The development of Brain-Machine Interfaces (BMI) is highly anticipated for enhancing the daily lives of patients who have lost somatic sensation due to brain or spinal cord injuries. Previously, somatosensory interfaces have transmitted tactile information through direct electrical stimulation to the primary somatosensory cortex (S1) to evoke the tactile sensation. However, a somatosensory interface capable of replicating the perception of hand movements has not been realized yet. This study, therefore, aimed to develop a stimulation method to replicate the perception of movement. We used marmosets, whose S1 is exposed on the brain surface, to devise a method for estimating the firing patterns of S1 neural activity based on information about the size and shape of objects manipulated by the hand. Initially, we created an experimental system with a lever attached to a haptic feedback device. This device can generate forces of varying sizes and orientations in three-dimensional space. The lever, which moves in two axes (forward and backward, up and down), allows an animal to feel a virtual object through counterforce. We had two marmosets operate the lever and recorded their S1 neural activity using a 32-channel multi-electrode array as they perceived a virtual object with a sloped shape. Initially, we analyzed whether S1 neural activity encodes information about virtual objects of two sizes (1mm and 7mm) using sparse logistic regression. The results indicated that the S1 encoded the size information of the virtual object during the slope-climbing process. Next, using hand trajectory data, we accurately estimated the timing of neural firing in the S1 by combining sparse linear regression with a firing model. Compared to shuffled control data, the estimation accuracy was significantly high. Thus, we successfully estimated the neural activity pattern of the S1 based on the spatial information of the touched objects.

### Late Breaking Abstracts

### [3LBA]
### Molecular physiology, Cell physiology
### Membrane transport

March 30, 13:00 - 14:20, Poster Room

### [3LBA-011]

### Identification of urate transport by sodium-dependent vitamin C transporters

*Yu Toyoda[1,2], Hiroshi Miyata[2], Hirotaka Matsuo[1], Tappei Takada[2] ([1]*National Defense Medical College,* [2]*The University of Tokyo Hospital*)

Uric acid is crucial because of its anti-oxidant activity and a causal relationship with hyperuricemia and gout. Like vitamin C, another water-soluble anti-oxidant, uric acid mainly exists as its anion form (urate) under physiological conditions; thus, it cannot passively penetrate the plasma membrane. Hence, active transport plays a pivotal role in regulating urate handling in humans. However, previously-identified urate transporters do not thoroughly explain such handling systems, suggesting the presence of latent machineries with physiological significance. To address this issue, we herein focused on SLC23A proteins that have been identified as sodium-dependent vitamin C transporters (SVCTs), because we previously identified SLC2A12 as a physiologically important urate and VC transporter (***PNAS***, 2020; ***iScience***, 2022) and a homology search revealed that SLC23A1/SVCT1 and SLC23A2/SVCT2 are the closest to YgfU (a urate transporter in *E. coli*) in amino acid sequence. To investigate the urate transport ability of SVCT1 and SVCT2, we conducted cell-based analyses using each transporter-expressing mammalian cells. The results demonstrated that SVCT1 [1] and SVCT2 [2] are novel urate transporters characterized by their lower affinity for urate compared with already-identified urate importers. Moreover, we generated a hyperuricemic mice model with *Svct1* knockout; in this model, serum urate levels were lower than controls, suggesting that Svct1 disruption could reduce serum urate [1]. Given that Svct1 physiologically functions as a renal vitamin C re-absorber, it could also be involved in urate re-uptake in the kidney. Regarding SVCT2, focusing on its molecular properties as a urate importer, we established a convenient cell-based urate efflux assay using SVCT2-expressing cells for urate exporter hunting [2].
On the other hand, the physiological role of SVCT2 as a urate transporter remains to be investigated; as *Svct2* knockout mice die soon after birth, conditional knockout approach will be required for this purpose. While further studies are needed to obtain deeper insights into the underlying mechanisms, our findings regarding the dual-substrate specificity of SVCTs expand the understanding of handling systems for the water-soluble antioxidants in our body.

[1] Toyoda *et al.*, ***Pflugers Arch.*** 2023, PMID: 36749388
[2] Toyoda *et al.*, ***J Biol Chem.*** 2023, PMID: 37390985

### Late Breaking Abstracts

[3LBA]

**Molecular physiology, Cell physiology**

**Ion channels, Receptors**

March 30, 13:00 - 14:20, Poster Room

### [3LBA-012]

### The $Na^+$ leak channel NALCN is inhibited by the activation of α2-adrenergic receptors in auditory neurons of mice

*Tomohiko IRIE[1], Tenzin Ngodup[2], Seán Elkins[2], Laurence Trussell[2] ([1]*Department of Physiology, Kitasato University School of Medicine,* [2]*Oregon Hearing Research Center and Vollum Institute, Oregon Health & Science University*)

Cartwheel inhibitory interneurons of the dorsal cochlear nucleus of mammals potently suppress multisensory signals that converge with primary auditory afferent input, and thus regulate auditory processing. Noradrenergic fibers from locus coeruleus project to the dorsal cochlear nucleus, and α2-adrenergic receptors inhibit spontaneous spike activity but simultaneously enhance synaptic strength in cartwheel cells, a dual effect leading to enhanced signal-to-noise for inhibition. However, the ionic mechanism of this striking modulation is unknown. Recently, it has been reported that some G protein-coupled receptors modulate neuronal activity through $Na^+$ leak channel NALCN. Therefore, we generated a glycinergic neuron-specific knockout of NALCN in mice and found that its presence was required for spontaneous firing in cartwheel cells. Activation of α2-adrenergic receptors inhibited both NALCN and spike generation, and this modulation was absent in the NALCN knockout. Moreover, α2-dependent enhancement of synaptic strength was also absent in the knockout. $GABA_B$ receptors mediated inhibition through NALCN as well, acting on the same population of channels as α2 receptors, suggesting close apposition of both receptor subtypes with NALCN. Thus, multiple neuromodulatory systems determine the impact of synaptic inhibition by suppressing the excitatory leak channel, NALCN.

### [3LBA-013]

### IL-1β and CK1α-Mediated Regulation of THIK-1 Channel Expression in Macrophages: Implications for Inflammation and Therapeutic Potential in Acute Lung Injury

Marie Merci Nyiramana[1,2], Eun-Jin Kim[1], Min-Seok Woo[1], Dang Long Cao[1,2], Ji Hyeon Ryu[3], Dong Kun Lee[1], Dong Woon Kim[4], *Dawon Kang[1,2] ([1]*Department of Physiology, College of Medicine and Institute of Medical Sciences, Gyeongsang National University,* [2]*Department of Convergence Medical Science, Gyeongsang National University,* [3]*Pusan National University Yangsan Hospital,* [4]*Chungnam National University School of Medicine*)

The tandem-pore domain halothane inhibited potassium (THIK)-1 channel is predominantly responsible for $K^+$ efflux in microglia. While blocking this channel inhibits IL-1β release, its expression is reduced by lipopolysaccharide (LPS). In this study, we investigated the mechanism and role of LPS-induced THIK-1 reduction in macrophages and an acute lung injury mouse model. We found that LPS treatment led to a concentration-dependent decline in THIK-1 mRNA and protein levels. Mechanistic insights revealed IL-1β as a pivotal mediator in this LPS-triggered THIK-1 downregulation. Further investigation into the underlying mechanisms implicated the involvement of casein kinase 1 (CK1) and the NF-κB signaling. Furthermore, the THIK-1 C-terminus, rich in CK1α phosphorylation sites, was required for its LPS-mediated downregulation and macrophage activation. In our acute lung injury mouse model, THIK-1 suppression downregulated inflammatory signals. Our results show that CK1 α activation mediates LPS-induced decrease of THIK-1 in macrophages. We suggest that THIK-1 could be a potential therapeutic target for inflammatory diseases.

### [3LBA-014]

### Therapeutic potential of fermented aged mountain cultivated ginseng and compound K in models of allergic asthma and acute lung injury through modulation of macrophage polarization

Long Dang Cao[1,2], Min-Seok Woo[1], Eun-Jin Kim[1], Ji Hyeon Ryu[3], Dong Kun Lee[1], Kye Man Cho[4], Sang Soo Kang[2,5], *Dawon Kang[1,2] ([1]*Department of Physiology and Institute of Medical Sciences, College of Medicine, Gyeongsang National University, Jinju, Republic of Korea,* [2]*Department of Convergence Medical Science, Gyeongsang National University, Jinju, Republic of Korea,* [3]*Research Institute for Convergence of Biomedical Science and Technology, Pusan National University Yangsan Hospital, Yangsan, Republic of Korea,* [4]*Department of GreenBio Science and Agri-Food Bio Convergence Institute, Gyeongsang National University, Jinju, Republic of Korea,* [5]*Department of Anatomy and Institute of Health Sciences, College of Medicine, Gyeongsang National University, Jinju, Republic of Korea*)

Macrophage polarization is key in the pathophysiology of asthma and acute lung injury (ALI), characterized by inflammation and oxidative stress. The effects of fermented aged mountain cultivated ginseng sprout (FAMCGS) and its main component, compound K (CK), on macrophage polarization in asthma and ALI are not yet known. FAMCGS is a specially processed form of ginseng, designed to enhance its bioactive components while shortening the cultivation period. This study aims to investigate the therapeutic potential of FAMCGS extracts and CK in modulating inflammatory responses and oxidative stress in models of allergic asthma and ALI, with a focus on their effects on macrophage regulation. FAMCGS, created by steaming, aging, and fermenting mountain-cultivated ginseng sprouts (MCGS), was analyzed for ginsenoside content using high-performance liquid chromatography. In vivo studies on mouse models of ovalbumin-induced allergic asthma and LPS-induced ALI involved oral administration of FAMCGS and CK. In vitro studies used the MH-S mouse alveolar macrophage cell line. Inflammatory and oxidative stress markers were assessed in lung tissue, bronchoalveolar lavage fluid, and MH-S cells through PCR, Western blotting, histological assays, and ELISA.FAMCGS showed higher antioxidant and anti-inflammatory activities than MCGS, with notably higher concentrations of CK. In OVA-induced asthma models, parameters like Th2 cytokines, IgE production, and mast cell activation were significantly elevated, as were ALI-related inflammatory cytokines and neutrophil counts in the LPS group. Treatment with FAMCGS and CK in these models notably reduced these elevated parameters and altered macrophage polarization. Additionally, FAMCGS and CK decreased both LPS-induced M1 and IL-4-induced M2 polarization in MH-S cells and lowered reactive oxygen species levels in lung tissue and MH-S cells.FAMCGS and CK showed therapeutic effectiveness in mouse models of LPS-induced ALI and OVA-induced asthma by mitigating inflammatory parameters and reducing free radicals through the downregulation of macrophage polarization. These findings suggest their potential as therapeutic agents for respiratory inflammatory conditions.

### [3LBA-015]

### Estimation of Probability Distributions of Ion Channel Gating Parameters using a Markov Chain Monte Carlo Method

*Takao Shimayoshi[1], Toru Kojima[2], Daisuke Sakurai[2] ([1]*Okayama University,* [2]*Kyushu University*)

The properties of ion channels measured in experiments often exhibit wide variations, as evidenced by high variances in measurements. These variations can even occur among clones of the same genotype due to the instability and fragility of ion channels.
To explain the gating mechanisms of ion channels, a common method is the continuous-time aggregated Markov model. This model represents each observable open and closed state with multiple states. To determine parameter values for such a Markov model, a widely used conventional method is point estimation from experimental measurements. However, point-estimated parameter values can be subject to uncertainty due to the high variances in measurements. Since the properties of an ion channel are not identical among individual clones in a series of experiments, a parameter of a Markov model could not be identified as a certain value but rather a probability distribution.
This study introduces a method for estimating probability distributions of model parameters from experimental measurements based on Bayesian statistics. The probability of a set of model parameter values for the observed data is computed as the posterior probability using the likelihood of the parameters through Bayesian inference. Parameter sets are sampled according to the posterior probability using a Markov chain Monte Carlo (MCMC) method. Global sampling in the parameter space is achieved using replica exchange MCMC sampling.
This presentation illustrates an estimation of parameter probability distributions of a three-state gating model for ryanodine receptor 2 channels to demonstrate the potential of the developing method.

### Late Breaking Abstracts

[3LBA]
**Muscle**

March 30, 13:00 - 14:20, Poster Room

### [3LBA-016]

### Myosin and tropomyosin–troponin complementarily regulate thermal activation of skeletal and cardiac muscles

*Shuya Ishii[1,2], Kotaro Oyama[1,2], Fuyu Kobirumaki-Shimozawa[2], Tomohiro Nakanishi[2,3], Naoya Nakahara[4], Madoka Suzuki[5], Shin'ichi Ishiwata[6], Norio Fukuda[2]
(*[1]QST, [2]Dept Cell Physiol, Sch Med, Jikei Univ, [3]Dept Anesthesiology, Sch Med, Jikei Univ, [4]Dept Mol Physiol, Sch Med, Jikei Univ, [5]IPR, Osaka Univ, [6]Fac Sci Engn, Waseda Univ*)

We previously explored how cardiac muscle contractions were affected by a change in temperature, and demonstrated that the heart can efficiently function within the body temperature range (Ishii et al., *J Gen Physiol*, 2019). In the present study, we investigated the differences in the temperature sensitivity of contraction in skeletal vs. cardiac muscles under optical heating microscopy. First, rapid heating (25 to 41.5°C) within 2 s induced reversible sarcomere shortening along isolated fast skeletal myofibrils in relaxing solution. Next, we investigated the temperature-dependence of the sliding velocity of reconstituted fast skeletal or cardiac thin filaments on fast skeletal or β-cardiac myosin in an *in vitro* motility assay within the body temperature range (up to 40°C). We found that (1) the temperature dependence with fast skeletal thin filaments on fast skeletal myosin was comparable to that obtained for sarcomere shortening in fast skeletal myofibrils ($Q_{10}$ ~8), (2) both types of thin filaments started to slide at lower temperatures on fast skeletal myosin than on β-cardiac myosin, and (3) cardiac thin filaments slid at lower temperatures compared with fast skeletal thin filaments on either type of myosin. Therefore, the mammalian striated muscle may be fine-tuned to contract efficiently via complementary regulation of myosin and tropomyosin–troponin within the body temperature range, depending on the physiological demands of various circumstances (Ishii et al., *J Gen Physiol*, 2023).

### [3LBA-017]

### Different gene expression profile in the skeletal muscle and fascia

*Hiroki Ota[1,2], Kimiaki Katanosaka[3], Toru Taguchi[1,2] (*[1]Dept. Phys. Ther., Fac. Rehabil., Niigata Univ. Health Welfare, Niigata, Japan., [2]Inst. Human Mov. Med. Sci., Niigata Univ. Health Welfare, Niigata, Japan., [3]Dept. Biomed. Sci., Col. Life Health Sci., Chubu Univ., Kasugai, Japan.*)

Fascia covering skeletal muscle is physiologically important as a supportive tissue. Several lines of evidence in recent studies have revealed that the fascia played roles as a nociceptive sensory tissue and that nociception in the fascia was more facilitated compared to that in the muscle in pathological conditions of myofascial structures. These observations indicate that the muscle and fascia, which are closely located from anatomical and functional points of view, have different molecular background with different genetic characteristics. In the present study, we investigated differences in gene expression profiles in the skeletal muscle and fascia using RNA-seq and subsequent pathway analyses. The rat tibialis anterior muscle and crural fascia covering the muscle were separately harvested immediately after euthanasia with inhalation of $CO_2$ gas and snap frozen for later analysis. After extracting total RNA from the tissues, quality control was performed against the purified RNA. Gene profiling was conducted using an mRatBN7.2 database. Gene expression levels were compared to identify differentially expressed genes (DEGs) between the muscle and fascia. The results showed that 4,888 DEGs were upregulated in fascia compared to muscle. Subsequent pathway analysis revealed that 33 reactome pathways were significantly increased in fascia compared to muscle. These pathways are categorized into “extracellular matrix organization, metabolism, hemostasis, immune system, signal translation, developmental biology, cell to cell communication, cellular responses to stimuli, and programmed cell death”. These results demonstrated that the gene expression profiles in fascia were different from those in neighboring skeletal muscle. This work was funded by JSPS KAKENHI (JP23K10542). There were no conflicts of interest related to this study.

### [3LBA-018]

### Women's Life Science: Proposal for a Research "Kata" Based on Body-Mind Integrative Science Linking Cells and Body through Metabolism

*Yoriko Atomi[1] (*[1]Teikyo University, ACRO*)

In the 1960s, I was assigned to the laboratory of Dr. Toshio Watanabe, a physiologist from Jikei Medical University, for my undergraduation research in physical education at Ochanomizu University, which I happened to enter as my second choice. I learned that there was a field of research that focused on the science of human. My project was a study of the brain. Later, while conducting research on human exercise, I decided to study "adaptation" in the field of exercise biochemistry. I realized that there were strategies for "genetic" engineering in molecular biology, and I adopted those strategies as well. I began to work mainly with "cells" because it is impossible to find interacting proteins in muscle rhabdomeres. From the beginning, I did not always say "cells, cells, cells. It is also significant that I came to take cells and genes for granted after visiting the laboratory of Dr. Yoshiaki Nonomura. While studying adaptation, I came across the molecular chaperone αB-crystallin, the caretaker of tubulin/microtubules, one of the three cytoskeletal proteins. This research links proteostasis and mechanical stress, areas that have been disconnected and unconnected until now. It was not until much later that I seriously read the book "The Physiology of Being Alive" by Dr. Toshio Watanabe, and upon closer reading, I found that it contains what I consider to be important. There is growing evidence, both nationally and internationally, that the cellular activity that assures our being alive is, at bottom, the dynamics of cytoskeletal protein fibers. In this presentation, I will introduce the academic book, "Body-Mind Integrative Science: Concepts and Methods: The αB-Crystallin Adaptation Theory Connecting Cells, Body Axis, and Energy Metabolism with Consciousness," published in March 2024.

### Late Breaking Abstracts

[3LBA]
**Oral physiology**

March 30, 13:00 - 14:20, Poster Room

**[3LBA-019]**

**Short-term effects of radiation on salivary glands**

*Hitoshi Uchida[1,2] ([1]*University of Toyama,* [2]*University of Rochester*)

Saliva is essential for the maintenance of oral health. A decrease in saliva secretion, known as dry mouth, has often developed in oral diseases including dental caries, gingivitis, and periodontitis, as well as eating and speaking impairments. Reduced salivation frequently results from multiple medications, autoimmune diseases such as Sjögren's syndrome, and radiation therapy for head and neck cancers. While medication-induced dry mouth has been considered "reversible" if the medications are stopped or so, a permanent loss of saliva secretion is caused by radiation therapy, which is commonly used to treat head and neck cancer patients worldwide. Although a consequence of radiotherapy for head and neck cancer results in a significant reduction in saliva flow and permanent loss of the secretory acinar cells, available treatments are only temporary and palliative. So, efforts to uncover the basis for salivary gland radiosensitivity are important to establish new methods for radioprotection and regenerative therapy. In this study, I examined the molecular mechanisms underlying the acute loss of secretory function that occurs in the first 48 hours post irradiation (IR). A $^{137}Cs$ gamma ray irradiator was utilized, with anatomical targeting (achieved using a slit collimator) to deliver IR bilaterally to murine submandibular glands (SMGs). Whole stimulated saliva was collected at 3, 24, and 48 hours, and weekly for 12 weeks following IR. Saliva volume was reduced rapidly by 3 hours and showed temporal recovery by 1 week post-IR. From 1 to 12 weeks, saliva volume decreased in a time-dependent manner. Histological analysis showed that miner changes in cell size and interstitial spaces were observed, and those changes correlated with altered expression and localization of epithelial barrier proteins. By 48 hours, IR also disrupted the expression of saliva functional molecules, including Aquaporin5 (water channel) and Mist1 (one of the acinar markers). The immunobiological staining for γH2AX, which labels IR-induced double-stranded DNA breaks, revealed that the number of its foci was significantly increased in both acinar and duct cells at 3 hours following IR. After that, its numbers were decreased in a time-dependent manner. Consistent with their DNA repair activity, mRNA expression levels of *Tgfb1*, *Foxo3a*, and *Gadd45a* were rapidly, but transiently, increased by 3 hours post-IR. For the long-term (3 months) effects of IR, acinar cells were found in isolated clusters. And those clusters can respond to stimulation with carbachol, which may still secrete saliva. In conclusion, the short-term effects of IR showed increased DNA damage with rapid alteration of expression for saliva functional molecules. By 3 months post-IR, clustered acinar cells surviving retain secretory function.

### Late Breaking Abstracts

[3LBA]
**Drug Action, Pharmacology**

March 30, 13:00 - 14:20, Poster Room

**[3LBA-020]**

### In vivo real-time imaging of protein therapeutics pharmacodynamics in nerve and muscle through intravital microscopy

*JIWON YOUN[1], Hyungjin Kwon[1], Hyunseok Kim[1] ([1]*IVIM Technology*)

Protein therapeutics are protein-based drugs that can be widely used for pharmacological purposes, offering endless possibilities in the field of medicine. In this research, we investigated protein drugs commonly used in medical applications such as treating diseases arising from various muscle spasticity uses. They are generated by bacteria and are known to have several types. These proteins inhibit the release of acetylcholine, a neurotransmitter released from the axon terminals at neuromuscular junctions. To investigate its mechanism of action on acetylcholine receptors, we monitored the pharmacodynamics of this protein drug in real-time, in vivo. To imaging of delivery and mode of action the protein drug, we performed surgery and opened the thigh skin of the mouse to uncover the nerve and muscle below after injected the protein drug intramuscularly into the right thigh of the mouse and observed the area 30 minutes and 4 hours after injection. We used for more definitely imaging Thy1-YFP16 mouse model which expresses endogenous EGFP (enhanced green fluorescent protein) in nerve (green), SHG (Second Harmonic Generation) in muscle (white). For analyzing the protein drug mechanism as imaging, we conjugated acetylcholine antibody with fluorophore FSD555 (red), and protein drug with fluorophore FSD647 (blue). Through this experiment, we were able to see the drug precisely positioning itself in the muscles and nerves over time. This allowed us to take a step forward in understanding the mechanism and dynamics of this protein drug. Like this way, methods that allow real-time imaging of the pharmacodynamics of protein drugs in vivo is expected to be effective for monitoring the pharmacodynamics of other drugs, and it will likely lead to various fields such as the development of new drugs. We expect that this will lead to future pharmaceutical development and disease treatment.

**Open Access** This article is licensed under a Creative Commons Attribution 4.0 International License, which permits use, sharing, adaptation, distribution and reproduction in any medium or format, as long as you give appropriate credit to the original author(s) and the source, provide a link to the Creative Commons licence, and indicate if changes were made. The images or other third party material in this article are included in the article's Creative Commons licence, unless indicated otherwise in a credit line to the material. If material is not included in the article's Creative Commons licence and your intended use is not permitted by statutory regulation or exceeds the permitted use, you will need to obtain permission directly from the copyright holder. To view a copy of this licence, visit http://creativecommons.org/licenses/by/4.0/.
